# Mediating Mendelian randomization in the proteome identified potential drug targets for obesity-related allergic asthma

**DOI:** 10.1186/s41065-025-00376-w

**Published:** 2025-02-01

**Authors:** Jiannan Lin, Shuwen Lu, Xiaoyu Zhao

**Affiliations:** Department of Pediatrics, Jiaxing Second Hospital, Jiaxing, 314000 China

**Keywords:** Allergic asthma, Obesity-related asthma, Plasma proteins, Mendelian randomization analysis

## Abstract

**Background:**

With the development of the economy, the number of obese patients has been increasing annually worldwide. The proportion of asthma patients associated with obesity is also gradually rising. However, the pathogenesis of obesity-related asthma remains incompletely understood, and conventional pharmacological treatments generally show limited efficacy.

**Objective:**

This study aims to explore the causal relationship between obesity and allergic asthma, elucidate the pathogenesis of obesity-related asthma, and identify the plasma proteins involved in its development, providing new insights for clinical interventions.

**Methods:**

In this study, we employed a two-step approach for mediation Mendelian randomization (MR) analysis, utilizing stringent selection criteria to identify instrumental variables (IVs). This approach was used to assess the causal impact of obesity on allergic asthma and to validate the plasma proteins identified as mediating factors. We further explored the functions and enriched pathways of the mediating proteins using Gene Ontology (GO) and Kyoto Encyclopedia of Genes and Genomes (KEGG) analyses. Finally, we conducted drug-targeted MR analysis to evaluate the potential of each mediator plasma proteins as a drug target gene. If significant heterogeneity remained among the IVs, we applied the weighted median method as the primary analytical tool. Otherwise, we utilized the inverse variance weighted (IVW) method as the main analytical approach. Additionally, we conducted various sensitivity analyses and statistical tests to further illustrate the robustness of the observed associations.

**Results:**

The research findings indicate a causal relationship between obesity and allergic asthma. Plasma proteins such as TPST1, ROR1, and DAPK1 mediate this relationship, with TPST1 accounting for over 10% of the mediation effect. GO and KEGG analyses show that the genes corresponding to these mediator proteins are primarily enriched in pathways related to responses to stimuli, carbohydrate synthesis and metabolism, regulation of certain protein activities, and synaptic connections. The drug-targeted MR analysis suggests that SIGLEC12, BOLA1, HOMER2, and TPST1 all have the potential to be drug target genes.

**Conclusion:**

This study suggests that obese patients defined by BMI may promote the development of allergic asthma by influencing the expression of plasma proteins such as TPST1, ROR1, and DAPK1. Furthermore, some of these plasma proteins, including TPST1, could potentially serve as therapeutic targets for treating allergic asthma in these patients. However, further research is needed to explore their therapeutic potential and the mechanisms underlying their effects.

**Clinical trial number:**

Not applicable.

**Supplementary Information:**

The online version contains supplementary material available at 10.1186/s41065-025-00376-w.

## Introduction

Asthma is a chronic inflammatory disease of the airways, characterized by reversible airflow obstruction and airway hyperresponsiveness. It typically worsens and becomes more symptomatic at night and in the early morning hours, often triggered by infections. Clinically, the main symptoms include wheezing, chest tightness, and coughing. Severe asthma attacks can be life-threatening [1]. In recent years, the prevalence of asthma has been steadily increasing. The global prevalence of asthma is approximately 10%, with significant differences in prevalence rates across countries with varying income levels [2].This rising incidence places a considerable burden on both families and society. Asthma manifests in various phenotypes, generally classified as allergic and non-allergic asthma, with allergic asthma accounting for the majority of cases [3].

Obesity has become an increasingly pressing social issue, with its prevalence rising steadily in tandem with economic development. In the United States, the incidence of obesity among children and adolescents surged from 3.6% in 1980 to 19.3% in 2018 [4]. Recent studies have demonstrated a significant link between obesity and both the onset and exacerbation of asthma [5]. Obese children are more likely to be diagnosed with asthma, experience more frequent attacks, and present with more severe symptoms during these episodes. Furthermore, they tend to exhibit a reduced response to systemic corticosteroids [6]. However, the exact mechanisms driving this association remain poorly understood.

Current research suggests that obesity-related asthma is primarily characterized by neutrophilic inflammation and is typically classified as a non-allergic type of asthma [7]. However, it has been frequently observed in clinical practice that some obese children, particularly among pediatric patients, present with features typical of allergic asthma [8]. Furthermore, conventional corticosteroid treatments often prove less effective, making asthma management even more challenging in these cases [9]. Therefore, this study aims to further clarify the pathogenesis of obesity-related asthma, focusing on exploring biomarkers that may act as intermediaries. The ultimate goal is to provide a scientific basis for more effective clinical interventions and management strategies for obesity-related asthma.

However, in practice, numerous confounding factors and reverse causality often make it challenging to derive definitive conclusions from observational studies. The MR is a research method used to explore potential causal relationships between exposure and outcome by selecting appropriate instrumental variables to substitute for the exposure and then analyzing their association with the outcome. Due to the random nature of genetic inheritance, this method closely mirrors the design of randomized controlled trials [10]. As such, it offers distinct advantages in investigating causal links between two traits.

## Materials and methods

### Study design

In this study, we employed a two-step approach for mediation MR analysis. First, we investigated the causal relationship between plasma proteins and allergic asthma. Next, we explored the causal relationship between body mass index (BMI), used as a proxy for obesity, and plasma proteins that have potential mediating roles. After identifying the mediating plasma proteins, we first calculated the causal effect of BMI on these mediators (β₁). In the second step, we assessed the causal effect of the mediating plasma proteins on allergic asthma (β₂). We applied the delta method to evaluate the significance of the mediation effect (β₁ × β₂) and determined the proportion of the mediation effect within the total effect. Next, we conducted GO and KEGG analyses on the mediating plasma proteins to explore their functional roles. Finally, after identifying the mediator proteins, we conducted drug-targeted MR analysis to evaluate the potential of each mediator plasma proteins as a drug target gene.

**Fig. 1:**
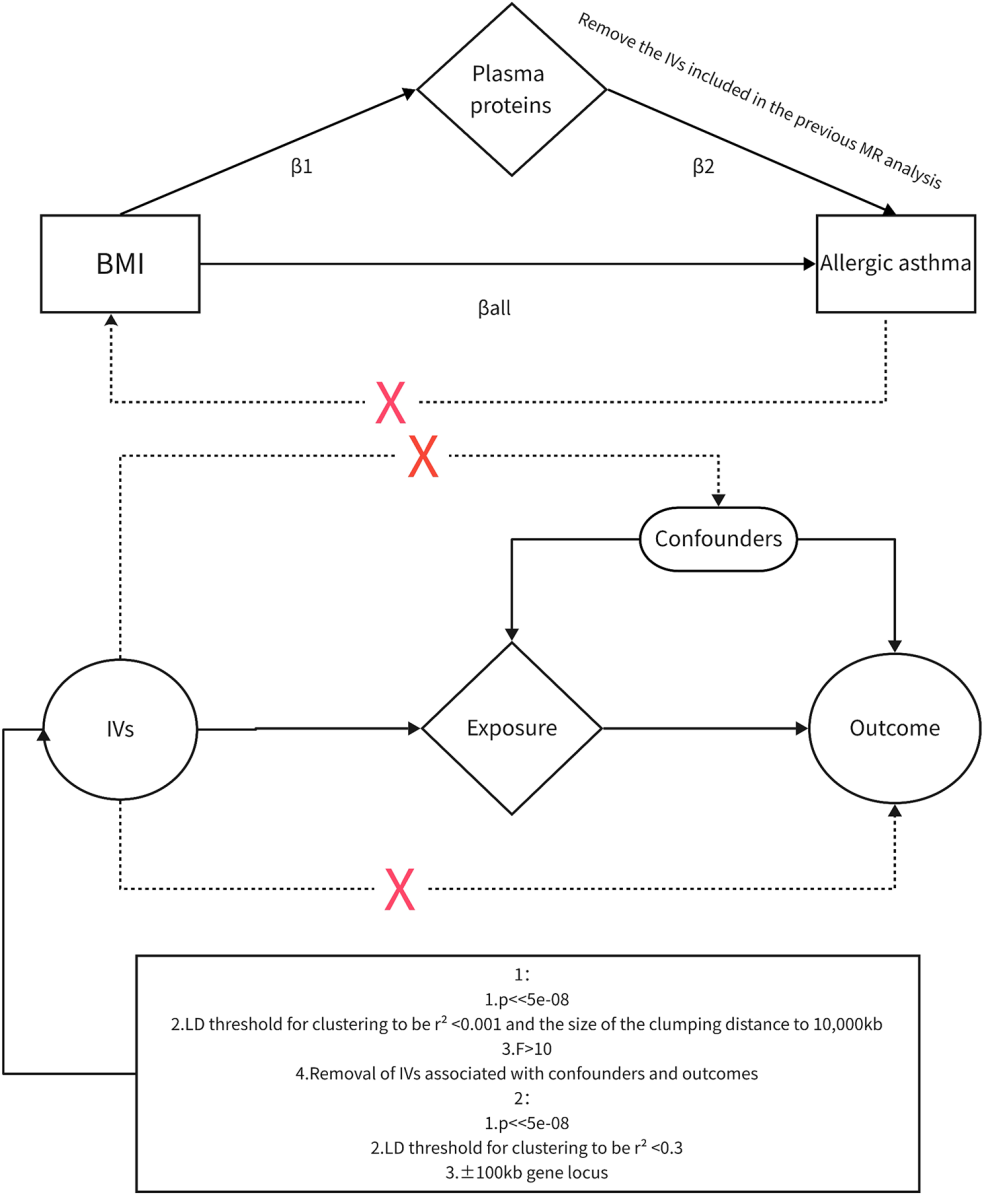
Mendelian randomization flowchart

The MR employs genetic variations as proxies for risk factors; therefore, effective instrumental variables in causal inference must satisfy three key assumptions: (1) The relevance assumption: genetic variations are directly associated with the exposure. (2) The independence assumption: genetic variations are independent of any confounding factors that may affect both the exposure and the outcome. (3) The exclusion assumption: genetic variations influence the outcome solely through the exposure, without any alternative pathways (Fig. 1).

### Data sources

In this study, the exposure data were sourced from the research findings of Barton AR et al.(accession numbers: ebi-a-GCST90025994) [11]. The plasma protein data were obtained from the aggregated dataset provided by Ferkingstad et al. in the Decode database (https://www.decode.com/summarydata/) [12]. The outcome data were derived from summary statistics related to allergic asthma in the FinnGen database (version r11; https://www.finngen.fi/en) [13]. Detailed information about the data can be found in Table 1.

**Table 1:** Details of exposure and outcome GWAS datasets

Dataset type	Consortium	Population	Sample size	PMID
BMI	Barton AR et al.	European	457,756	34,226,706
Plasma protein	Ferkingstad et al.	European	35,559	34,857,953
Allergic asthma	FinnGen	European	260,128	-

### Instrumental variables selection

The selected IVs had to meet the following criteria: (1) To ensure robust results, a p-value threshold of < 5 × 10⁻⁸ was used. (2) To maintain the independence of each IV, a linkage disequilibrium (LD) threshold of r² < 0.001 was applied for clustering, with a clustering distance set at 10,000 kb. (3) The F-statistic for each IV was calculated to assess weak instrument bias, with IVs selected if F > 10. (4) The GWAS Catalog database (https://www.ebi.ac.uk/gwas/) was utilized to exclude instrumental variables associated with confounding factors and outcomes.

### GO and KEGG analysis

We utilized the “clusterProfiler” and “enrichplot” packages to conduct GO enrichment analysis and KEGG pathway enrichment analysis on the genes corresponding to the identified intermediate proteins. In this analysis, gene annotation was performed using “org.Hs.eg.db” packages.

### The drug-targeted MR

After identifying the mediator proteins, we conducted drug-targeted MR analysis to evaluate the potential of each mediator protein as a drug target gene. The selection of instrumental variables met the following criteria: (1) *p* < 5 × 10^-8; (2) IVWs were located within ± 100 kb of the target gene locus; (3) LD threshold (r² < 0.3).

### Statistical analyses

To assess the stability of causal relationships and the validity of the hypotheses, we conducted various sensitivity analyses and statistical tests. To evaluate the heterogeneity and pleiotropy among the IVs, we employed Cochran’s Q statistic and the MR-Egger intercept test. The “leave-one-out” method, along with scatter plots and funnel plots, can be used to ensure the robustness of the results. We removed any unstable results from our analysis.

If significant heterogeneity among the instrumental variables was detected, we adopted the weighted median method as the primary analytical approach. Conversely, if no heterogeneity was observed among the IVs, we applied the IVW method as the main analysis technique. All analyses were performed using R software, along with the MR (Version 4.4.1). Given the exploratory nature of the study, we did not apply the FDR correction, setting the significance threshold at *p* < 0.05 [14, 15].

## Results

Based on the selection criteria established for our IVs, we first identified IVs closely related to BMI, followed by those associated with plasma proteins, and finally, those linked to allergic asthma. Using the GWAS Catalog database (https://www.ebi.ac.uk/gwas/), we excluded IVs that were related to confounding factors and outcomes in their respective MR analyses.

### The impact of BMI on allergic asthma

**Fig. 2:**
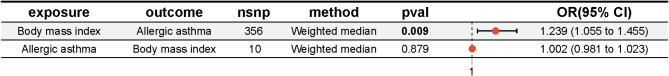
The results of Mendelian randomization for BMI and allergic asthma

Initially, we performed a MR analysis to examine the causal relationship between BMI and allergic asthma. The findings revealed a significant causal link, with an OR of 1.239 (95% CI: 1.055–1.455, *p* = 0.009) (Fig. 2). In contrast, reverse MR analysis indicated no causal relationship between allergic asthma and BMI (*P* > 0.05) (Fig. 2). Furthermore, the Cochran’s Q statistic indicated significant heterogeneity in the results (Supplementary Material 1). Therefore, we used the weighted median method as the primary analytical approach for this study. Additionally, the MR-Egger intercept test indicated that our MR analysis was not affected by horizontal pleiotropy (Supplementary Material 1). Scatter plots, funnel plots, and leave-one-out analysis further illustrated the robustness of the correlations (Supplementary Materials 1).

### The impact of plasma proteins on asthma

**Fig. 3:**
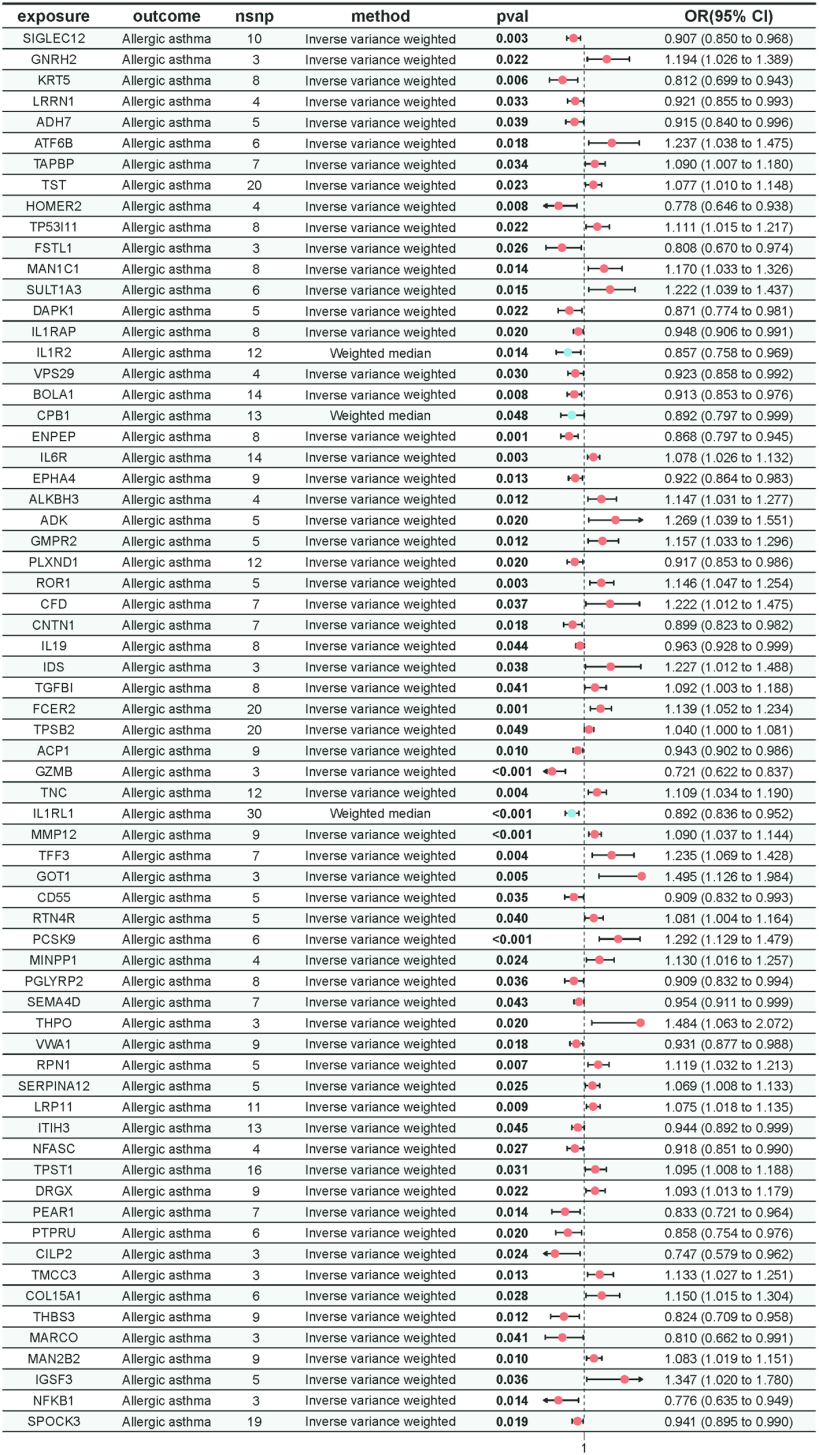
The results of Mendelian randomization for plasma proteins and allergic asthma

In our MR analysis investigating the relationship between plasma proteins and allergic asthma, we used the MR-Egger intercept test to eliminate results affected by horizontal pleiotropy. Ultimately, we identified 67 plasma proteins that exhibit a causal relationship with allergic asthma. Among these, 33 plasma proteins were found to serve as protective factors, while 34 were identified as detrimental factors for allergic asthma. The specific results can be seen in Fig. 3. Cochran’s Q statistic assessments revealed significant heterogeneity in the MR results for IL1R2, CPB1, and IL1RL1. Consequently, we utilized the weighted median method for the analysis of IL1R2, CPB1, and IL1RL1, while the IVW method was applied as the primary analytical approach for the remaining plasma proteins (Supplementary Materials 2). Scatter plots, funnel plots, and leave-one-out analyses further demonstrated the robustness of these associations (Supplementary Materials 1).

### The impact of BMI on plasma proteins

**Fig. 4:**
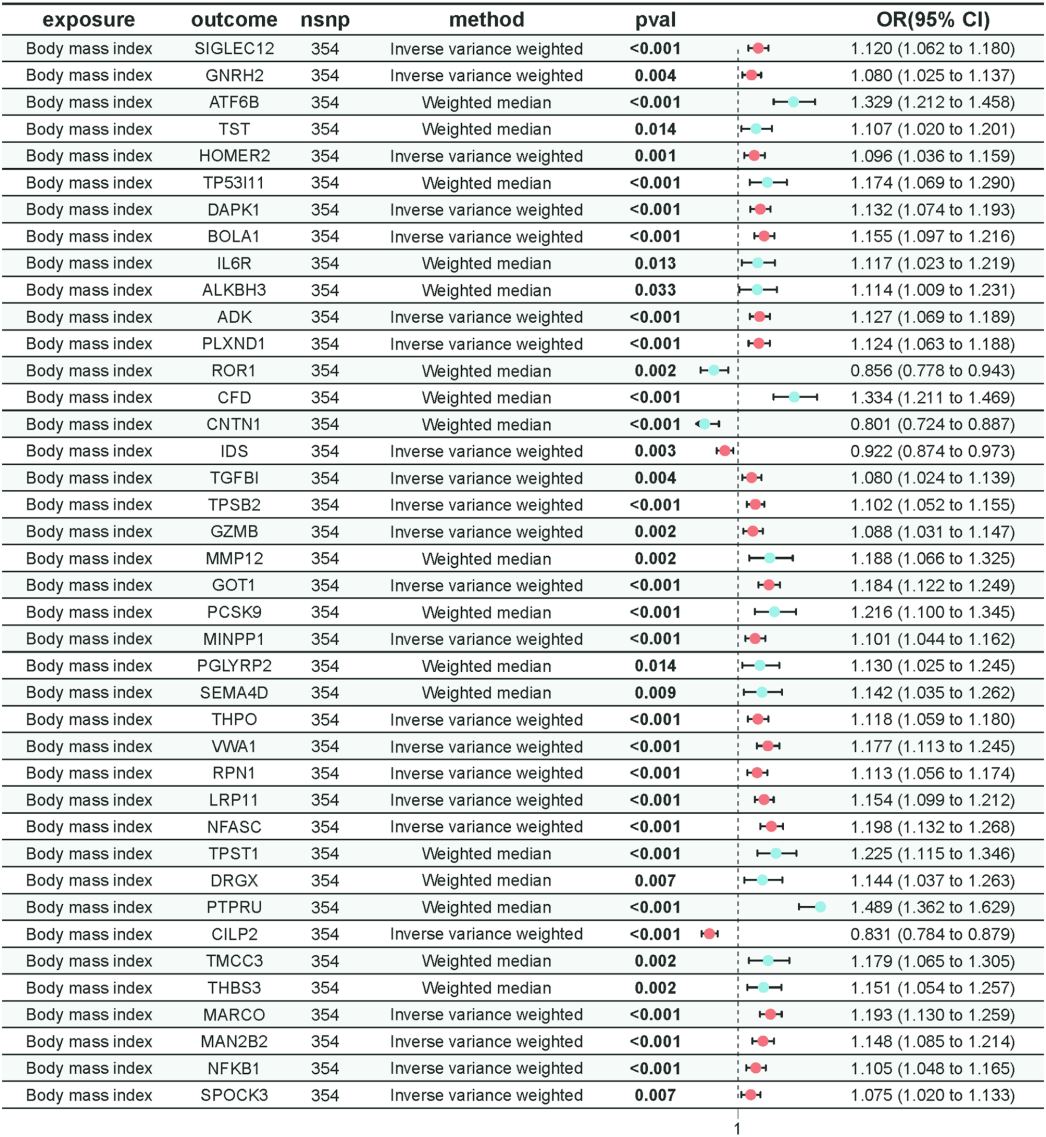
The results of Mendelian randomization for BMI and plasma proteins

In our MR analysis investigating the relationship between BMI and the selected plasma proteins, we used the MR-Egger intercept test to eliminate results affected by horizontal pleiotropy. Ultimately, we identified a total of 40 plasma proteins with potential mediating factor roles, as illustrated in the accompanying figure (Fig. 4). Cochran’s Q statistic evaluation indicated significant heterogeneity in the MR results for ATF6B, TST, TP53I11, IL6R, ALKBH3, ROR1, CFD, CNTN1, MMP12, PCSK9, PGLYRP2, SEMA4D, TPST1, DRGX, PTPRU, TMCC3, and THBS3 (Supplementary Materials 3). Consequently, we applied the weighted median method for the analysis of these specific plasma proteins, while the IVW method was utilized as the primary analytical approach for the remaining plasma proteins. Scatter plots, funnel plots, and leave-one-out analyses further supported the robustness of these associations (Supplementary Materials 3).

### Mediation results

**Fig. 5:**
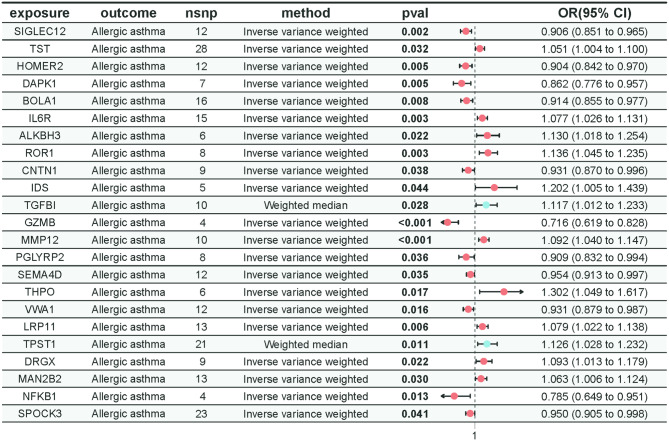
The results of Mendelian randomization for mediating plasma proteins and allergic asthma

We utilized a two-step approach for mediation MR to study the plasma proteins identified as having mediator factor potential. In this analysis, we used the MR-Egger intercept test to eliminate results affected by horizontal pleiotropy. Ultimately, we identified 23 significant mediator factors and recalculated the β2 results, as detailed in Fig. 5. In the second step of mediation MR, when recalculating the β2 results, the Cochran’s Q statistic assessment indicated significant heterogeneity in the MR results for TGFBI and TPST1 (Supplementary Material 4). Therefore, we applied the weighted median method for the analysis of these specific plasma proteins, while the IVW method was used as the primary analysis method for the remaining plasma proteins. Scatter plots, funnel plots, and leave-one-out analyses further supported the robustness of these associations (Supplementary Materials 4). By assessing the calculated β1 and β2 results, we evaluated the significance of the mediation effect (β1 × β2) and determined the proportion of the mediation effect within the total effect. Notably, the mediation effects of SIGLEC12, HOMER2, DAPK1, BOLA1, ROR1, VWA1, LRP11, TPST1, and MAN2B2 were found to be significant. Detailed results are presented in Table 2.

**Table 2:** The results of the mediating mendelian randomization analysis

Exposure	Metabolite	Outcome	Mediated_effect	Mediated_proportion	*p* value
BMI	SIGLEC12	Allergic asthma	-0.0112(-0.0198, -0.00253)	-5.2%(-9.22%, -1.18%)	0.01123966
BMI	HOMER2	Allergic asthma	-0.00927(-0.0181, -0.000466)	-4.32%(-8.43%, -0.217%)	0.039049369
BMI	DAPK1	Allergic asthma	-0.0184(-0.0353, -0.00155)	-8.58%(-16.4%, -0.721%)	0.032366324
BMI	BOLA1	Allergic asthma	-0.013(-0.0226, -0.00345)	-6.06%(-10.5%, -1.61%)	0.007652693
BMI	ROR1	Allergic asthma	-0.0198(-0.038, -0.00151)	-9.22%(-17.7%, -0.705%)	0.033811865
BMI	VWA1	Allergic asthma	-0.0116(-0.0217, -0.00158)	-5.42%(-10.1%, -0.736%)	0.023326572
BMI	LRP11	Allergic asthma	0.0109(0.00278, 0.0189)	5.06%(1.3%, 8.82%)	0.008415302
BMI	TPST1	Allergic asthma	0.024(0.0021, 0.046)	11.2%(0.979%, 21.4%)	0.031740497
BMI	MAN2B2	Allergic asthma	0.00848(1.09e-05, 0.0169)	3.95%(0.00507%, 7.9%)	0.049702042

### GO and KEGG analyses

**Fig. 6:**
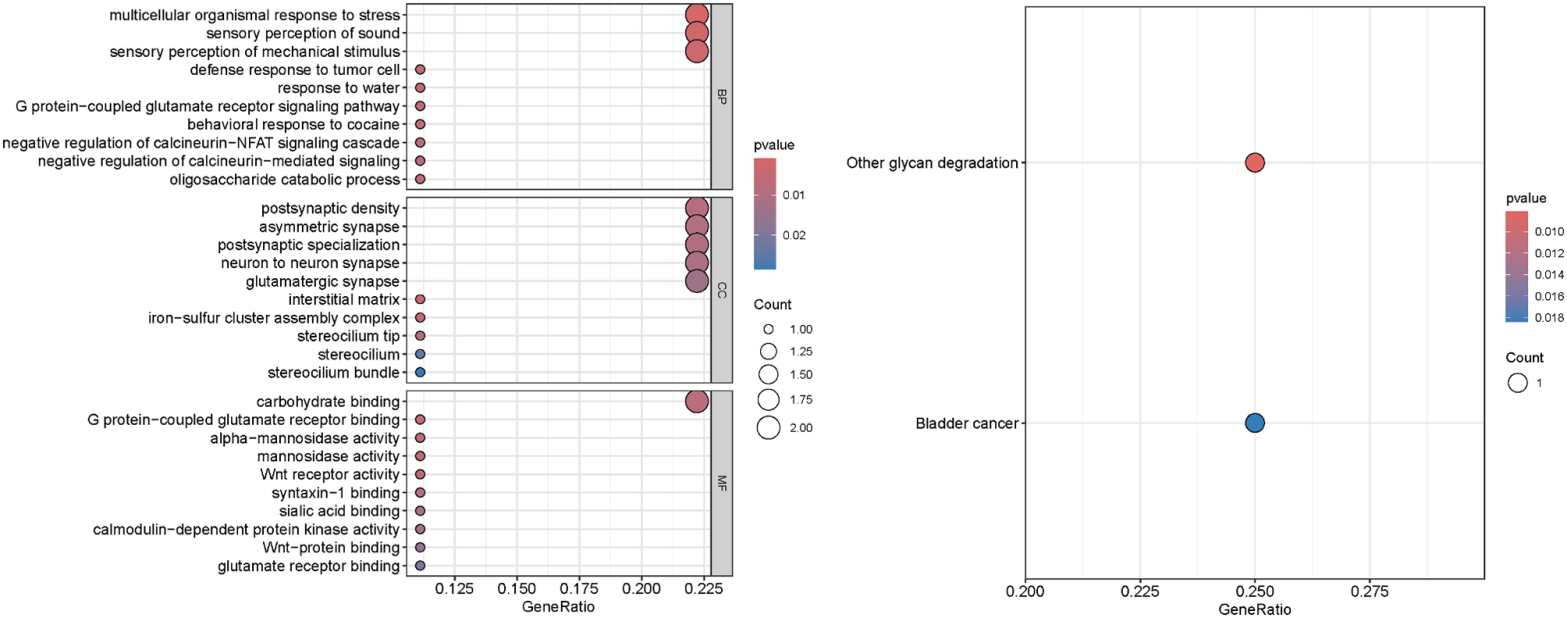
(**A**) The results of GO analyses. (**b**) The results of KEGG analyses

To further explore the functions and enriched pathways of the genes corresponding to these plasma proteins, we performed GO and KEGG analyses. The results indicated that these genes are primarily enriched in pathways related to responses to stimuli, carbohydrate synthesis and metabolism, regulation of certain protein activities, and synaptic connections (Fig. 6).

### The drug-targeted MR

**Fig. 7:**
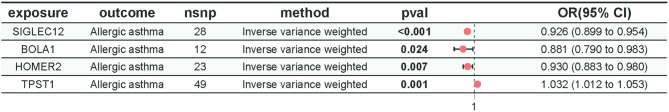
The results of drug-targeted Mendelian randomization

We conducted drug-targeted MR analysis on the selected mediator plasma proteins. The results showed the following: SIGLEC12 (OR = 0.93, 95% CI = 0.90–0.95, *p* < 0.01), BOLA1 (OR = 0.88, 95% CI = 0.79–0.98, *p* = 0.02), HOMER2 (OR = 0.93, 95% CI = 0.88–0.98, *p* < 0.01), and TPST1 (OR = 1.03, 95% CI = 1.01–1.05, *p* < 0.01) (Fig. 7). Furthermore, the Cochran’s Q statistic indicated that there is no heterogeneity in the results (Supplementary Material 5). Therefore, we used the IVW method as the primary analytical approach for this study. Additionally, the MR-Egger intercept test indicated that our MR analysis was not affected by horizontal pleiotropy (Supplementary Material 5). Scatter plots, funnel plots, and leave-one-out analysis further illustrated the robustness of the correlations (Supplementary Materials 5).

## Discussion

Obesity-related asthma is widely recognized as a neutrophilic, non-allergic type of asthma. However, the relationship between obesity-related asthma and allergic asthma has garnered limited discussion. In this study, we used BMI as a surrogate for obesity and conducted a two-step mediation MR analysis to further clarify the underlying mechanisms of obesity-related asthma. Our goal was to investigate the causal relationship between obesity-related asthma and allergic asthma while identifying plasma proteins that may serve as mediators in this context. This approach aims to provide new insights for the treatment of obesity-related asthma.

Recent studies have demonstrated that obesity is not only an independent risk factor for asthma but also affects the phenotype and clinical manifestations of the disease [16]. Asthma that is closely associated with obesity is clinically defined as obesity-related asthma. This type of asthma is characterized by more frequent attacks, more severe symptoms, and higher mortality rates compared to other forms of asthma [17]. The pathogenesis of obesity-related asthma appears to be related to the inflammatory effects of lipids and the mechanical alterations in the airways [18]. However, the specific mechanisms underlying this condition remain unclear. Current pharmacological treatments often show limited efficacy in managing obesity-related asthma [19].

Based on existing literature, asthma can be broadly categorized into allergic and non-allergic types. The former is characterized by eosinophilic airway inflammation, whereas the latter is associated with neutrophilic inflammation. Among these, a distinctive form known as obesity-related asthma has emerged. Most studies unequivocally report that airway inflammation in obesity-related asthma is predominantly neutrophilic, which suggests its classification as a non-allergic asthma [20]. However, a minority of studies have indicated that obesity-related asthma may also exhibit eosinophilic inflammation [21, 22]. This discrepancy raises an intriguing question: Should obesity-related asthma be classified as allergic or non-allergic? This question is particularly significant as it directly influences treatment strategies. Allergic asthma typically shows a favorable response to corticosteroids, resulting in considerable symptom improvement under standardized treatment regimens. In contrast, non-allergic asthma is often resistant to corticosteroids, leading to persistent symptoms that necessitate additional therapeutic approaches [23]. Our study’s findings indicate a causal relationship between obesity and allergic asthma, suggesting that, under certain conditions, obesity may lead to the development of allergic asthma in individuals. Integrating prior research, it appears that the immunological profile of obesity-related asthma may not be fixed; it has the potential to manifest as either allergic or non-allergic asthma. The variability in how obesity triggers different asthma phenotypes may be linked to disease progression or patient age. However, the precise mechanisms remain to be elucidated. This variability in immune imbalance may partly explain the difficulties in achieving effective treatment for obesity-related asthma. Given the possibility of diverse immunological profiles, a standardized pharmacological approach may be ineffective.

The results of this study indicate that plasma proteins such as TPST1, ROR1, and DAPK1 may mediate the relationship between obesity and allergic asthma. Notably, the mediation effect of TPST1 exceeds 10%, underscoring its significant clinical relevance. GO analysis suggests that these genes are primarily involved in responses to stimuli, carbohydrate synthesis and metabolism, regulation of certain protein activities, and synaptic connections. Furthermore, KEGG analysis reveals that these genes are predominantly associated with pathways related to carbohydrate metabolism and bladder cancer. It is hypothesized that some genes may influence carbohydrate synthesis and metabolism, altering energy supply mechanisms and contributing to obesity. Additionally, abnormalities in synaptic connections between neurons may impair the function of the pulmonary autonomic nervous system, thereby affecting normal lung physiology.

Tyrosyl sulfotransferase 1 (TPST1) is an enzyme responsible for the sulfation modification of substrates, specifically by transferring sulfate groups to tyrosine residues, thus facilitating the sulfation of tyrosine. This sulfation process occurs throughout the body and is closely linked to not only normal physiological functions but also various pathological conditions. Research has indicated that the sulfation of heparan sulfate and chondroitin sulfate chains can significantly impact multiple aspects of inflammation, playing a crucial role in T-cell infiltration [24, 25]. N-acetylglucosamine (GlcNAc) 6-O sulfation has been shown to protect gut microbiota and modulate immune responses [26]. Additionally, sterol sulfate has been found to alleviate insulin resistance and systemic inflammation in obese mice, suggesting that TPST1 may also influence obesity-related asthma [27]. Therefore, a deeper exploration of the specific mechanisms by which sulfation contributes to inflammation is of great significance for developing novel anti-inflammatory therapeutic strategies.

The drug-targeted MR analysis indicated that SIGLEC12, BOLA1, HOMER2, and TPST1 all have the potential to be drug target genes. However, a search of the DrugBank database revealed that there are currently no approved drugs targeting these genes. Future research is anticipated to explore this potential further.

However, this study has several limitations. First, the use of BMI to define obesity has inherent limitations, as BMI does not accurately reflect the distribution of body fat. Future research should aim to utilize more comprehensive indicators to represent different types of obese populations, enhancing the generalizability of the conclusions drawn. Second, the sample was exclusively drawn from a European population, raising concerns about the general applicability of the findings in a global context, which still needs validation. Future directions should involve collecting data from diverse geographic regions for further verification. Third, we did not employ multiple comparison controls in this study in order to explore a wider range of plasma proteins with potential as drug targets, thus providing more hypotheses and possibilities for future research. Nevertheless, the clinical relevance of the identified drug target proteins requires further experimental validation. Our next research plan includes establishing corresponding gene amplification expression and knockout models of obese asthmatic mice to test our hypotheses and extend our findings. Lastly, although Mendelian Randomization analysis has advantages in controlling for known confounding factors, there remains a risk that undetected confounding variables may influence the study outcomes.

Future research should delve deeper into the mechanisms underlying obesity-related asthma and explore the mediating factors involved in its pathogenesis. Furthermore, it is advisable to conduct multi-center, large-scale studies to validate and expand upon the findings of this study, thereby enhancing the generalizability and reliability of the results.

## Conclusion

This study suggests that obese patients defined by BMI may promote the development of allergic asthma by influencing the expression of plasma proteins such as TPST1, ROR1, and DAPK1. Furthermore, some of these plasma proteins, including TPST1, could potentially serve as therapeutic targets for treating allergic asthma in these patients. However, further research is needed to explore their therapeutic potential and the mechanisms underlying their effects.

## Electronic supplementary material

Below is the link to the electronic supplementary material.

Supplementary Material 1: Supplementary Materials 1. The MR results of BMI and allergic asthma. Supplementary Materials 2. The MR results of plasma proteins and allergic asthma. Supplementary Materials 3. The MR results of BMI and plasma proteins. Supplementary Materials 4. The MR results of mediation factor plasma proteins and allergic asthma. Supplementary Materials 5. The results of drug-targeted MR

## Data Availability

The data for allergic asthma was obtained from the FinnGen database (https://storage.googleapis.com/finngen-public-data-r11/summary_stats/finngen_R11_ALLERG_ASTHMA.gz).The BMI data was sourced from the IEU-OpenGWAS project (https://gwas.mrcieu.ac.uk/datasets/ebi-a-GCST90025994/).The plasma protein data were obtained from the aggregated dataset provided by Ferkingstad et al. in the Decode database (https://www.decode.com/summarydata/).

## References

[1] Pinedo Sierra C, Curto Sánchez E, Diaz Campos R et al. Asma Open Respiratory Archives, 2024;6(2). 10.1016/j.opresp.2024.100324 PMC11067451 38707659

[2] Porsbjerg C, Melén E, Lehtimäki L, et al. Asthma. Lancet. 2023;401(10379):858–73. 36682372 10.1016/S0140-6736(22)02125-0

[3] King-Biggs MB. Asthma. Ann Intern Med. 2019;171(7):ITC49–64. 31569251 10.7326/AITC201910010

[4] Reyes-Angel J, Kaviany P, Rastogi D, et al. Obesity-related asthma in children and adolescents. Lancet Child Adolesc Health. 2022;6(10):713–24. 35988550 10.1016/S2352-4642(22)00185-7 PMC12090466

[5] Ahmadizar F, Vijverberg SJH, Arets HGM, et al. Childhood obesity in relation to poor asthma control and exacerbation: a meta-analysis. Eur Respir J. 2016;48(4):1063–73. 27587561 10.1183/13993003.00766-2016

[6] Dixon AE, Que LG. Obesity and asthma. Semin Respir Crit Care Med. 2022;43(05):662–74. 35176784 10.1055/s-0042-1742384

[7] Nyambuya TM, Dludla PV, Mxinwa V, et al. Obesity-related asthma in children is characterized by T-helper 1 rather than T-helper 2 immune response: a meta-analysis. Annals of Allergy, Asthma & Immunology; 2020;125:425–e432424. 4. 10.1016/j.anai.2020.06.020 32561508

[8] To M, Arimoto Y, Honda N et al. Clinical characteristics and cytokine profiles of adult obese asthma with type2 inflammation. Sci Rep, 2023;13(1). 10.1038/s41598-023-41889-6 PMC10491644 37684314

[9] Mcgarry ME, Castellanos E, Thakur N, et al. Obesity and Bronchodilator Response in black and hispanic children and adolescents with asthma. Chest. 2015;147(6):1591–8. 25742612 10.1378/chest.14-2689 PMC4451713

[10] Chen LG, Tubbs JD, Liu Z, et al. Mendelian randomization: causal inference leveraging genetic data. Psychol Med. 2024;54(8):1461–74. 38639006 10.1017/S0033291724000321

[11] Barton AR, Sherman MA, Mukamel RE, et al. Whole-exome imputation within UK Biobank powers rare coding variant association and fine-mapping analyses. Nat Genet. 2021;53(8):1260–9. 34226706 10.1038/s41588-021-00892-1 PMC8349845

[12] Ferkingstad E, Sulem P, Atlason BA, et al. Large-scale integration of the plasma proteome with genetics and disease. Nat Genet. 2021;53(12):1712–21. 34857953 10.1038/s41588-021-00978-w

[13] Kurki MI, Karjalainen J, Palta P, et al. FinnGen provides genetic insights from a well-phenotyped isolated population. Nature. 2023;613(7944):508–18. 36653562 10.1038/s41586-022-05473-8 PMC9849126

[14] Zeng W, Hu M, Zhou L, et al. Exploring genetic links between blood metabolites and gout susceptibility. Clin Rheumatol. 2024;43(12):3901–12. 39467906 10.1007/s10067-024-07215-9

[15] Zeng W, Wu Y, Liang X et al. Causal associations between human gut microbiota and osteomyelitis: a mendelian randomization study. Front Cell Infect Microbiol, 2024;14. 10.3389/fcimb.2024.1338989 PMC11035795 38655282

[16] Miethe S, Karsonova A, Karaulov A, et al. Obesity and asthma. J Allergy Clin Immunol. 2020;146(4):685–93. 33032723 10.1016/j.jaci.2020.08.011

[17] Mohan A, Grace J, Wang BR, et al. The effects of obesity in asthma. Curr Allergy Asthma Rep. 2019;19(10):49. 31506820 10.1007/s11882-019-0877-z

[18] Sharma V, Cowan DC, Obesity. Inflammation, and severe asthma: an update. Curr Allergy Asthma Rep. 2021;21(12):46. 34921631 10.1007/s11882-021-01024-9 PMC8684548

[19] Forno E, Lescher R, Strunk R, et al. Decreased response to inhaled steroids in overweight and obese asthmatic children. J Allergy Clin Immunol. 2011;127(3):741–9. 21377042 10.1016/j.jaci.2010.12.010 PMC3056233

[20] Tooba R, Wu TD. Obesity and asthma: a focused review. Respir Med. 2022;204:107012. 36279813 10.1016/j.rmed.2022.107012 PMC9671155

[21] Desai D, Newby C, Symon FA, et al. Elevated Sputum Interleukin-5 and Submucosal Eosinophilia in obese individuals with severe asthma. Am J Respir Crit Care Med. 2013;188(6):657–63. 23590263 10.1164/rccm.201208-1470OC PMC3826183

[22] Grasemann H, Holguin F. Oxidative stress and obesity-related asthma. Paediatr Respir Rev. 2021;37:18–21. 32660723 10.1016/j.prrv.2020.05.004

[23] Li Y, Yang T, Jiang B. Neutrophil and neutrophil extracellular trap involvement in neutrophilic asthma: a review. Medicine, 2024, 103(34). 10.1097/MD.0000000000039342 PMC11346896 39183388

[24] Gopal S. Syndecans in inflammation at a glance. Front Immunol, 2020;11. 10.3389/fimmu.2020.00227 PMC7040480 32133006

[25] Hirani P, Mcdermott J, Rajeeve V, et al. Versican Associates with Tumor Immune phenotype and limits T-cell trafficking via chondroitin sulfate. Cancer Res Commun. 2024;4(4):970–85. 38517140 10.1158/2767-9764.CRC-23-0548 PMC10989462

[26] Abo H, Muraki A, Harusato A et al. N-acetylglucosamine-6-O sulfation on intestinal mucins prevents obesity and intestinal inflammation by regulating gut microbiota. JCI Insight, 2023;8(16). 10.1172/jci.insight.165944 PMC10543739 37463055

[27] Zhang H-J, Chen C, Ding L et al. Sea cucumbers-derived sterol sulfate alleviates insulin resistance and inflammation in high-fat-high-fructose diet-induced obese mice. Pharmacol Res, 2020;160. 10.1016/j.phrs.2020.105191 32911073

