# Supplementary material for: Mediating Mendelian randomization in the proteome identified potential drug targets for obesity-related allergic asthma
Source: Hereditas. 2025 Feb 1;162:12. doi: 10.1186/s41065-025-00376-w (PMC11786417; doi:10.1186/s41065-025-00376-w)
Supplement: Supplementary file 1 — Supplementary Material 1: Supplementary Materials 1. The MR results of BMI and allergic asthma. Supplementary Materials 2. The MR results of plasma proteins and allergic asthma. Supplementary Materials 3. The MR results of BMI and plasma proteins. Supplementary Materials 4. The MR results of mediation factor plasma proteins and allergic asthma. Supplementary Materials 5. The results of drug-targeted MR [file 41065_2025_376_MOESM1_ESM.zip › Supplementary Materials/S2 The MR results of plasma proteins and allergic asthma/S2 plasma proteins-Allergic asthma.scatter_plot.pdf]

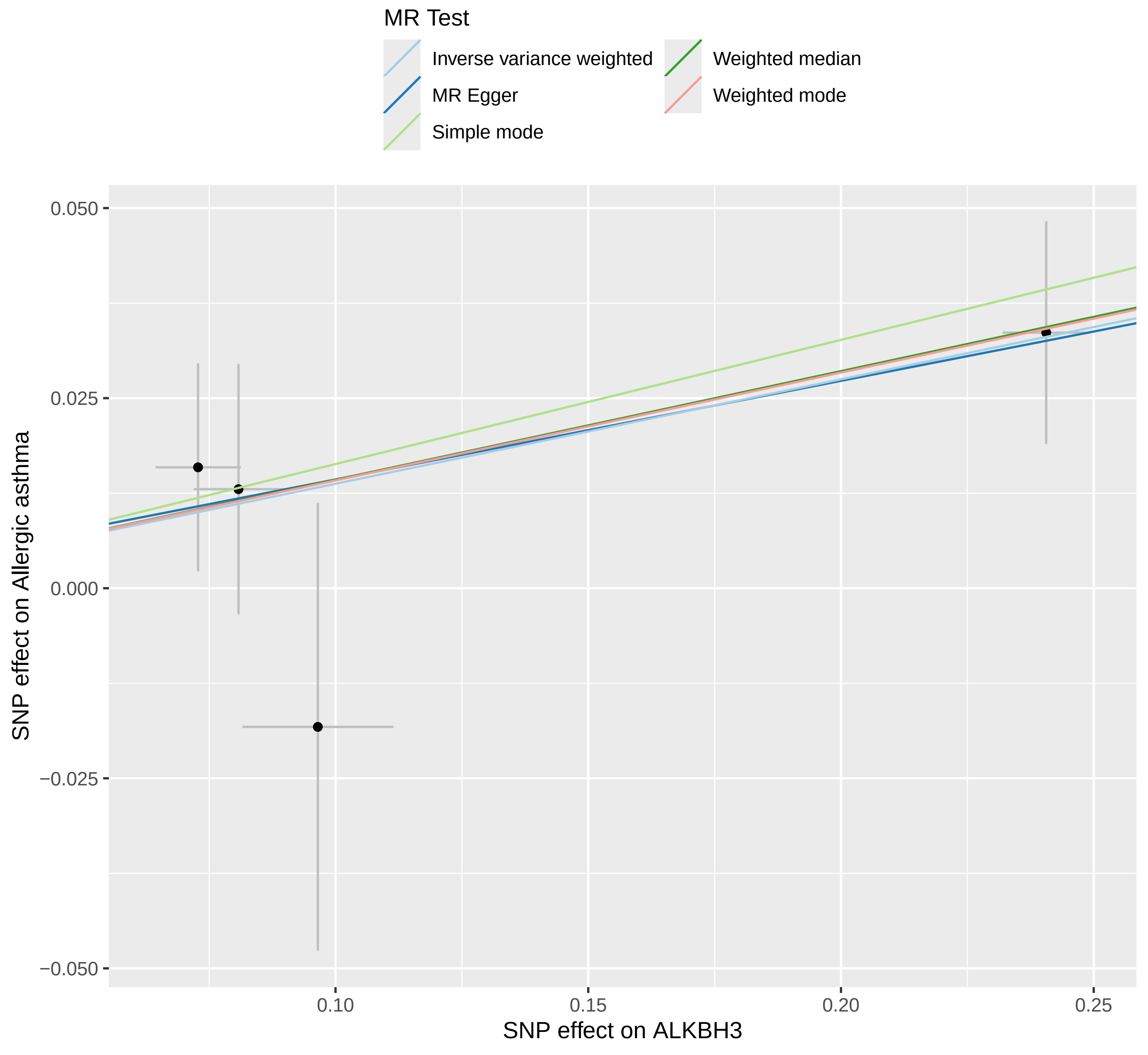

MR Test
Inverse variance weighted
MR Egger
Simple mode
Weighted median
Weighted mode
0.050
0.025
0.000
−0.025
−0.050
SNP effect on Allergic asthma
0.10
0.15
0.20
0.25
SNP effect on ALKBH3

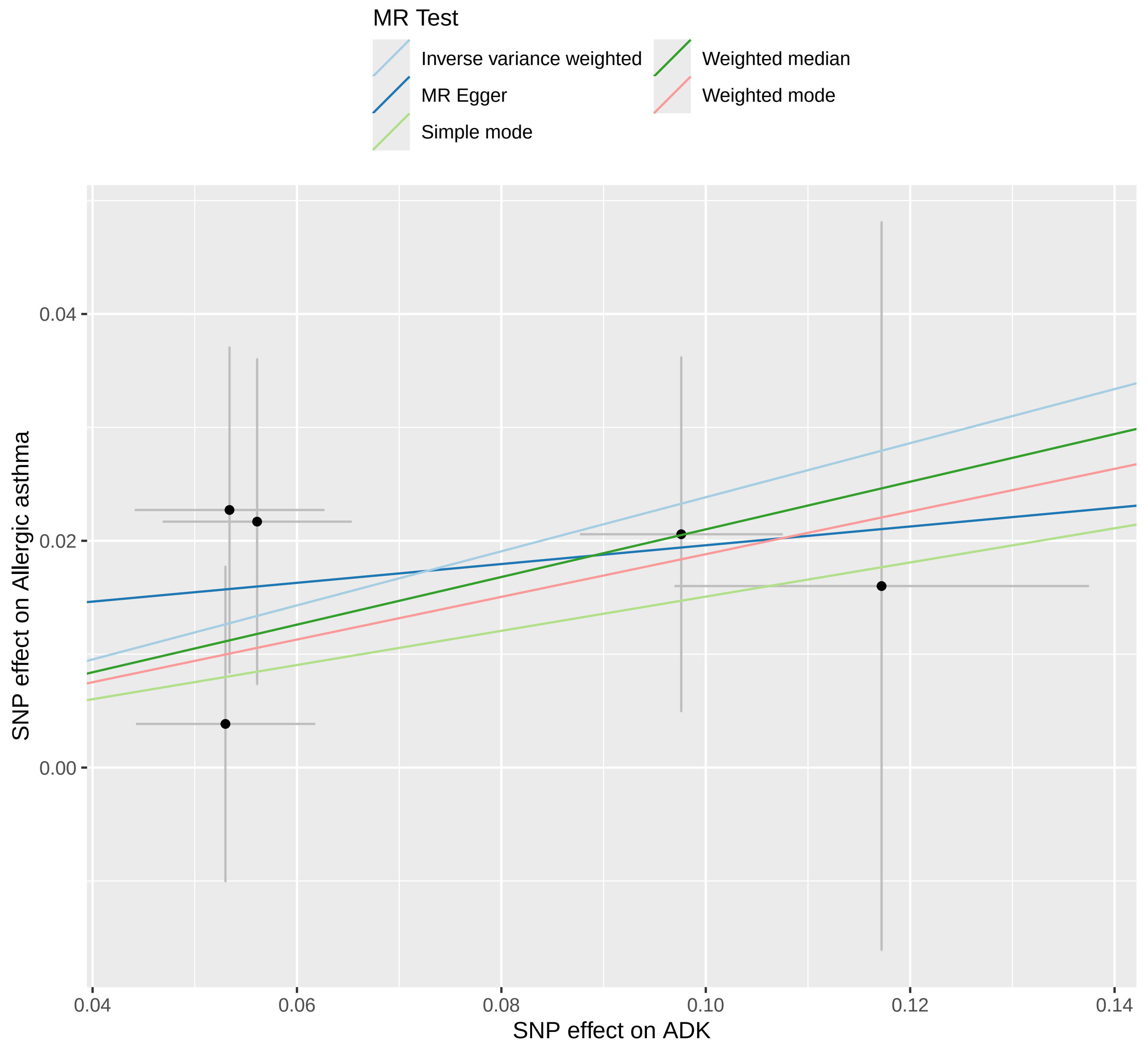
MR Test
Inverse variance weighted
MR Egger
Simple mode
Weighted median
Weighted mode
0.04
0.02
0.00
SNP effect on Allergic asthma
0.04
0.06
0.08
0.10
0.12
0.14
SNP effect on ADK

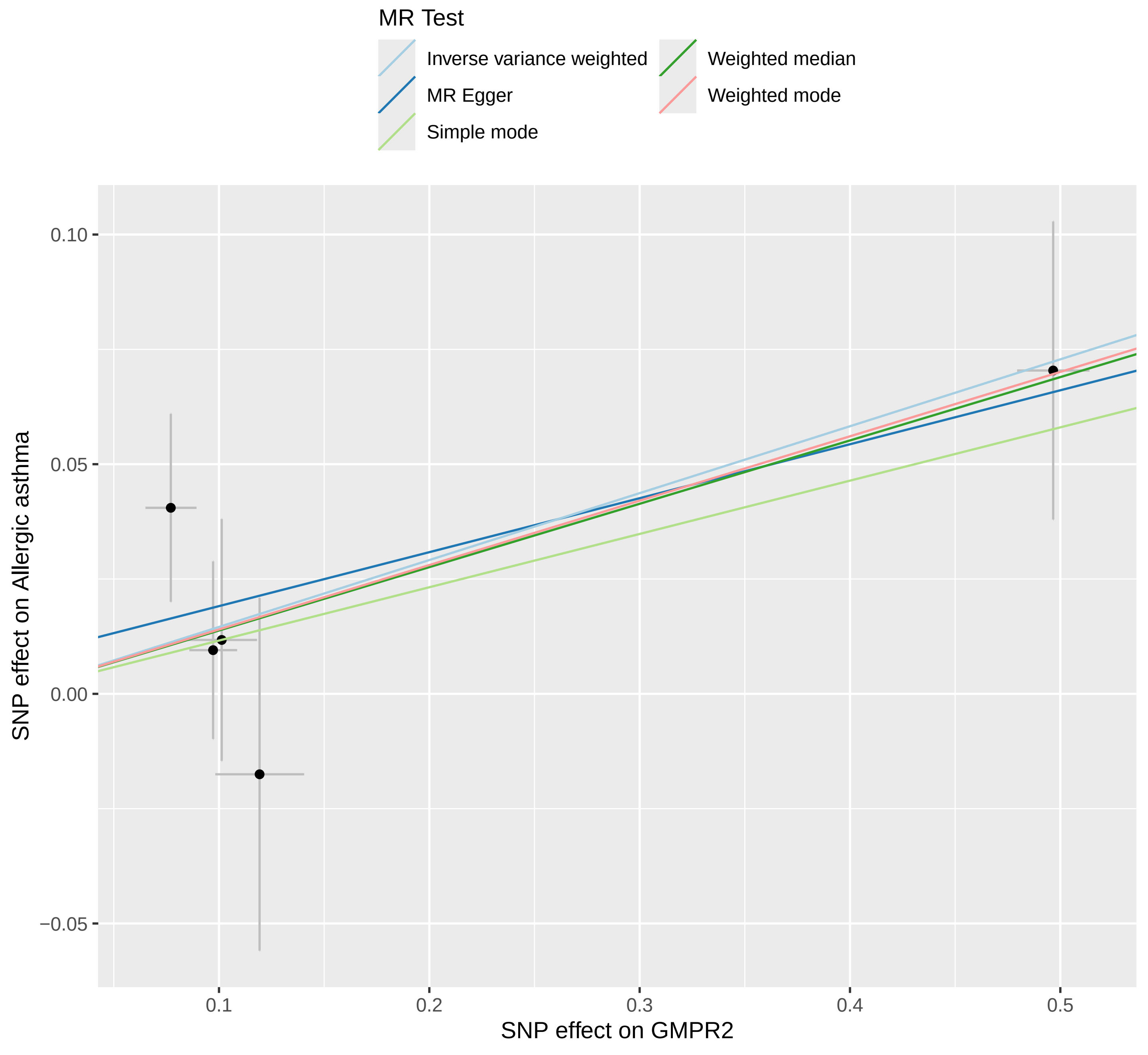
MR Test
Inverse variance weighted
MR Egger
Simple mode
Weighted median
Weighted mode
0.10
0.05
0.00
−0.05
0.1
0.2
0.3
0.4
0.5
SNP effect on Allergic asthma
SNP effect on GMPR2

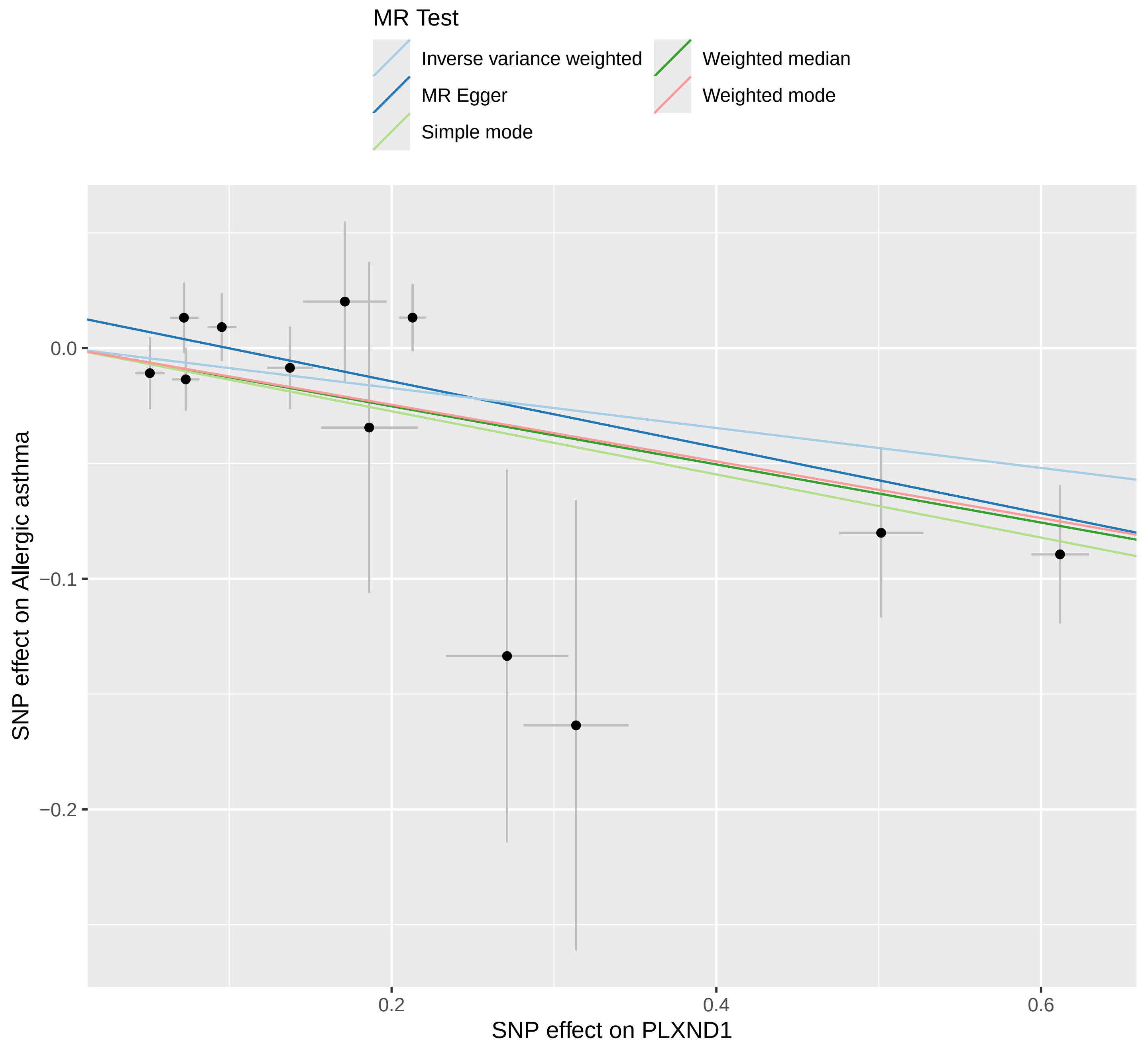
MR Test
Inverse variance weighted
MR Egger
Simple mode
Weighted median
Weighted mode
0.0
−0.1
−0.2
0.2
0.4
0.6
SNP effect on Allergic asthma
SNP effect on PLXND1

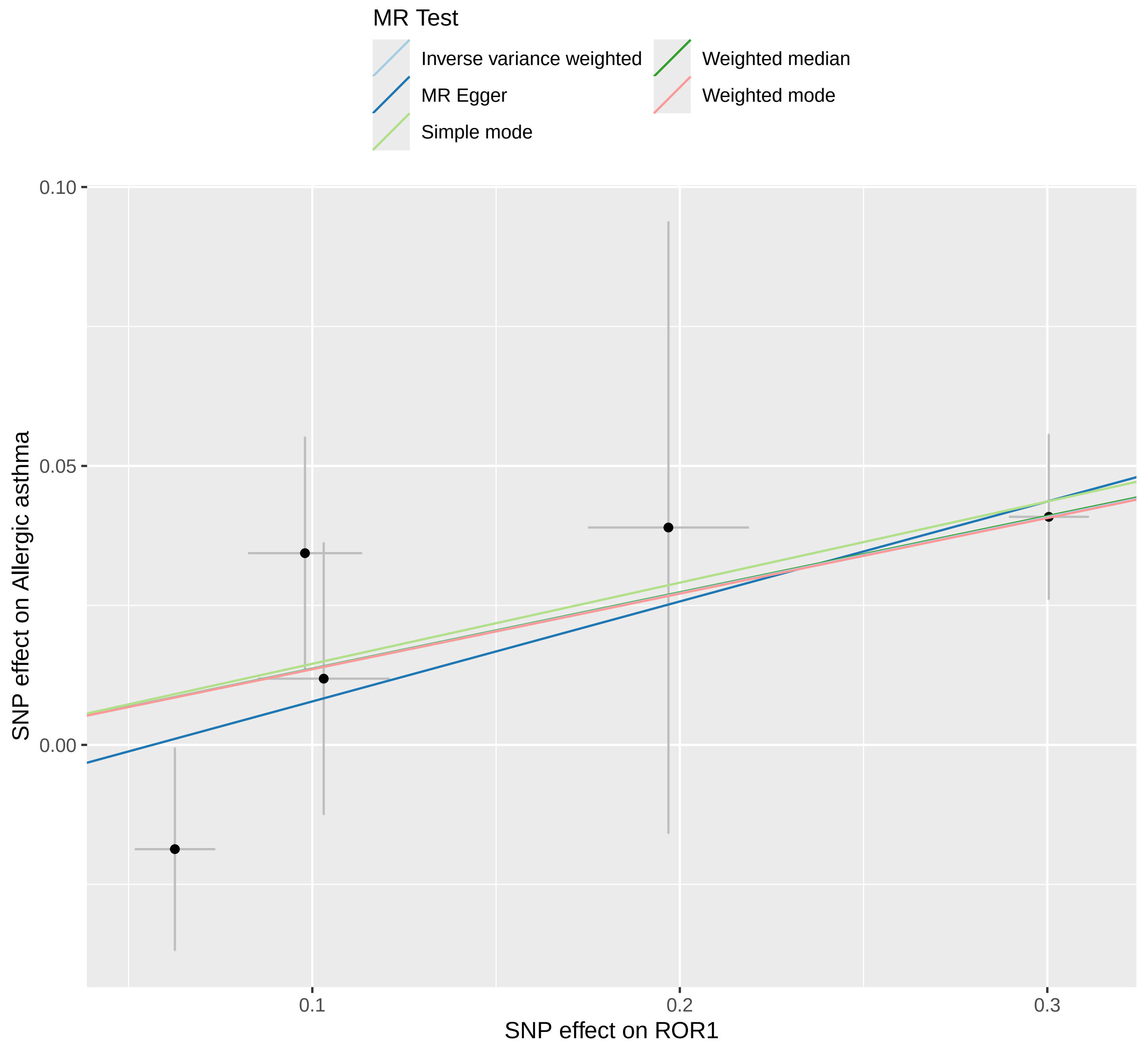
MR Test
Inverse variance weighted
MR Egger
Simple mode
Weighted median
Weighted mode
0.10
0.05
0.00
0.1
0.2
0.3
SNP effect on Allergic asthma
SNP effect on ROR1

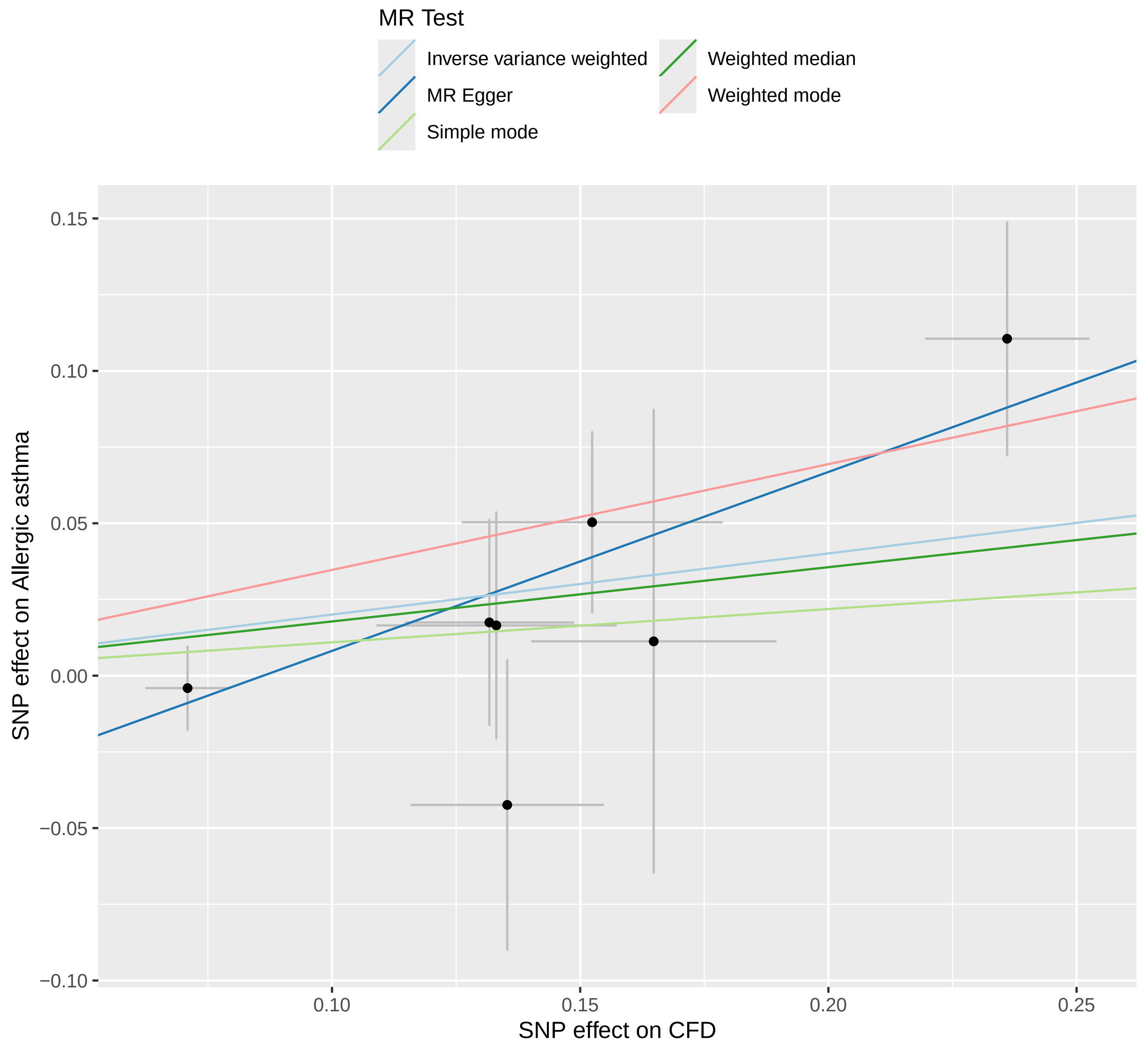

MR Test
Inverse variance weighted
MR Egger
Simple mode
Weighted median
Weighted mode
0.15
0.10
0.05
0.00
−0.05
−0.10
SNP effect on Allergic asthma
0.10
0.15
0.20
0.25
SNP effect on CFD

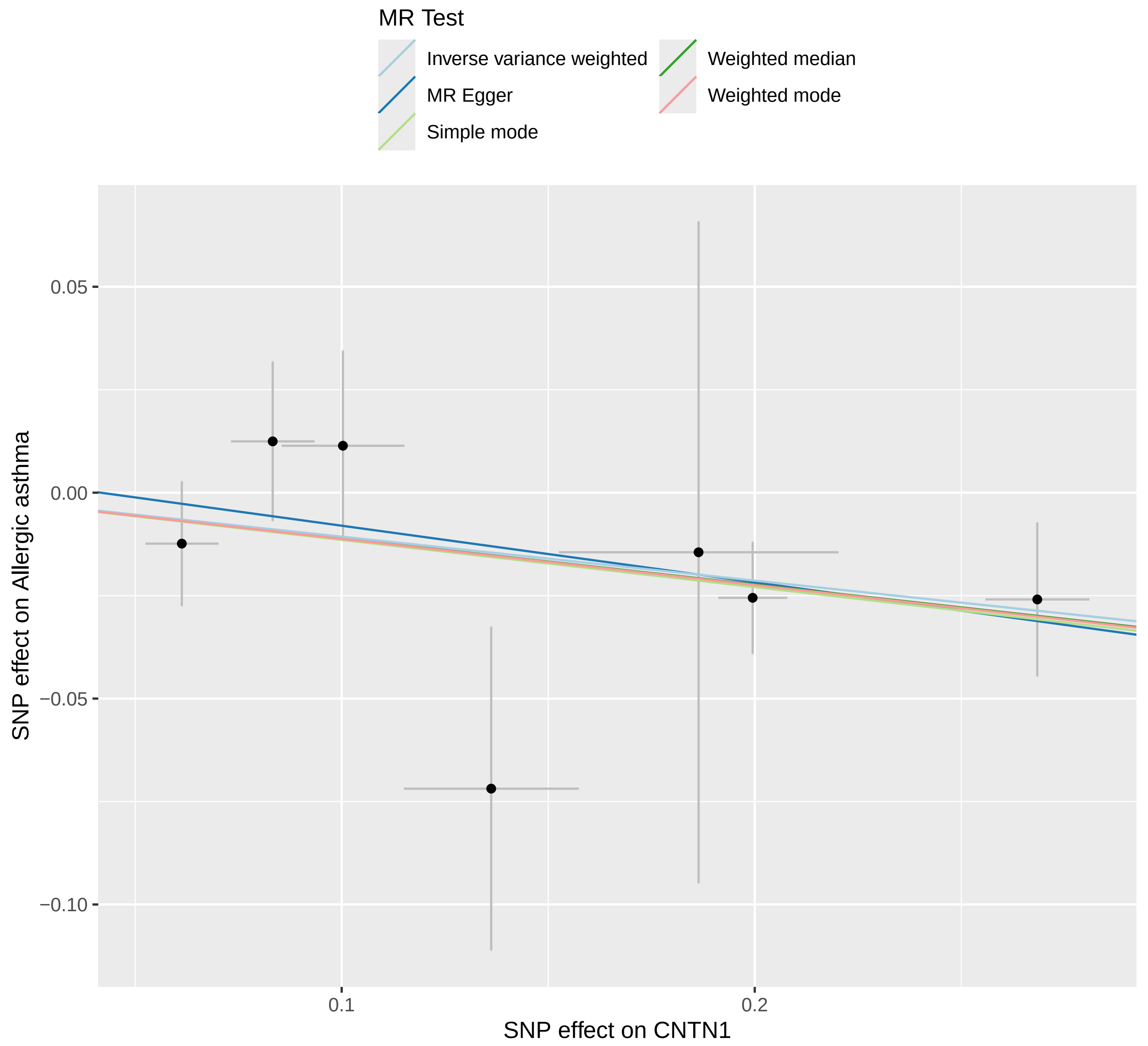
MR Test
Inverse variance weighted
MR Egger
Simple mode
Weighted median
Weighted mode
0.05
0.00
−0.05
−0.10
SNP effect on Allergic asthma
0.1
0.2
SNP effect on CNTN1

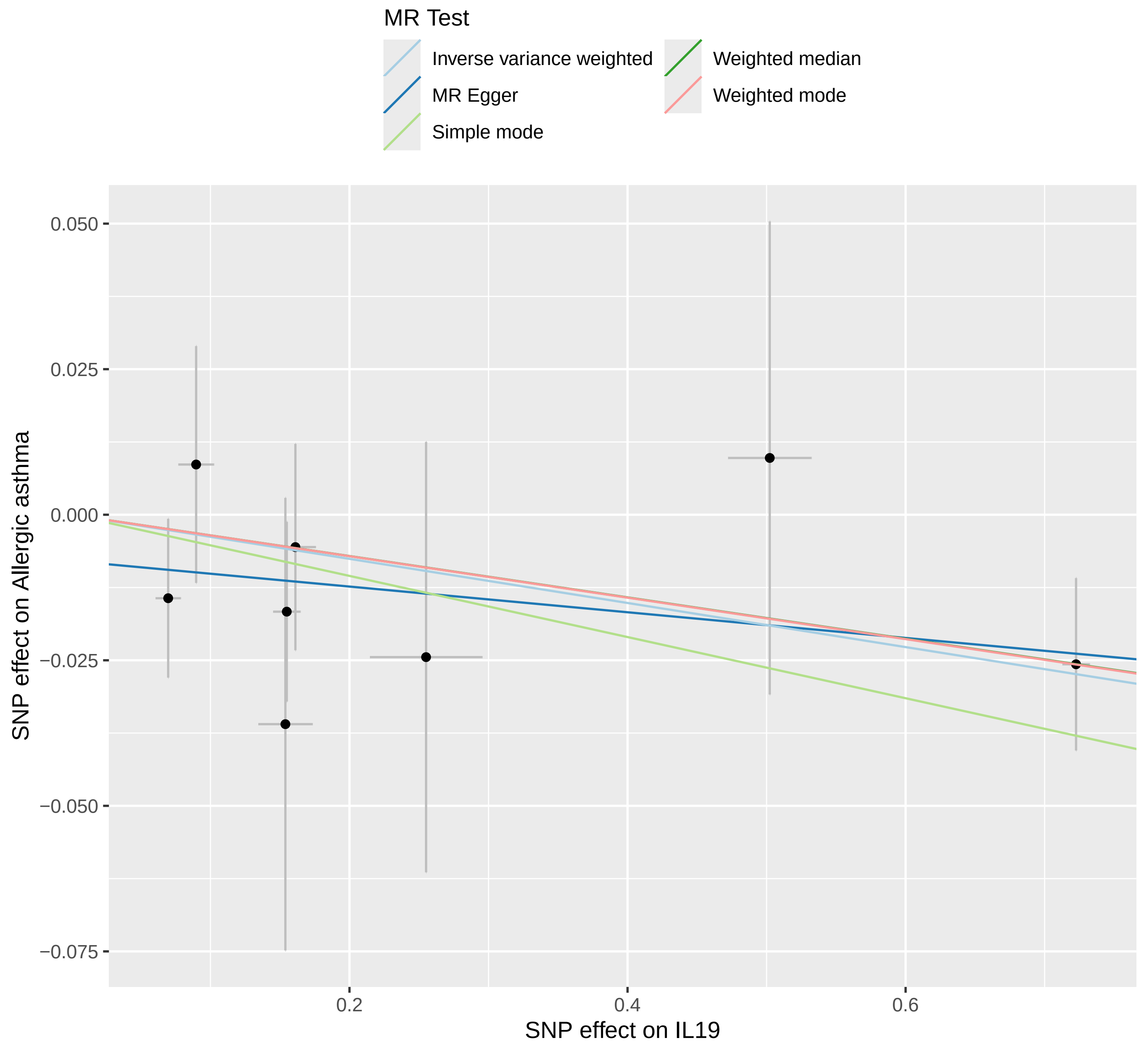
MR Test
Inverse variance weighted
MR Egger
Simple mode
Weighted median
Weighted mode
0.050
0.025
0.000
−0.025
−0.050
−0.075
0.2
0.4
0.6
SNP effect on IL19
SNP effect on Allergic asthma

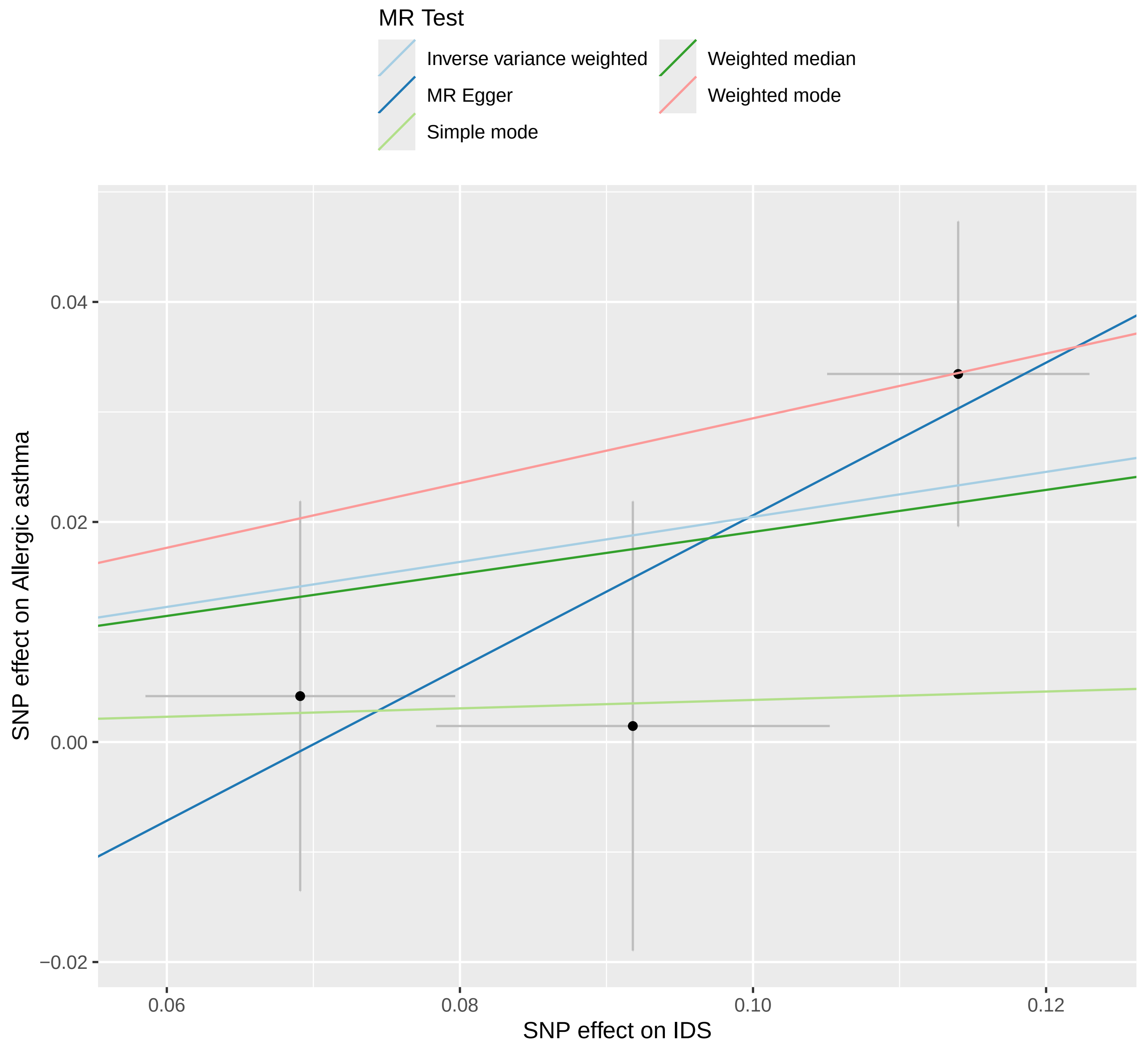
MR Test
Inverse variance weighted
MR Egger
Simple mode
Weighted median
Weighted mode
0.04
0.02
0.00
−0.02
SNP effect on Allergic asthma
0.06
0.08
0.10
0.12
SNP effect on IDS

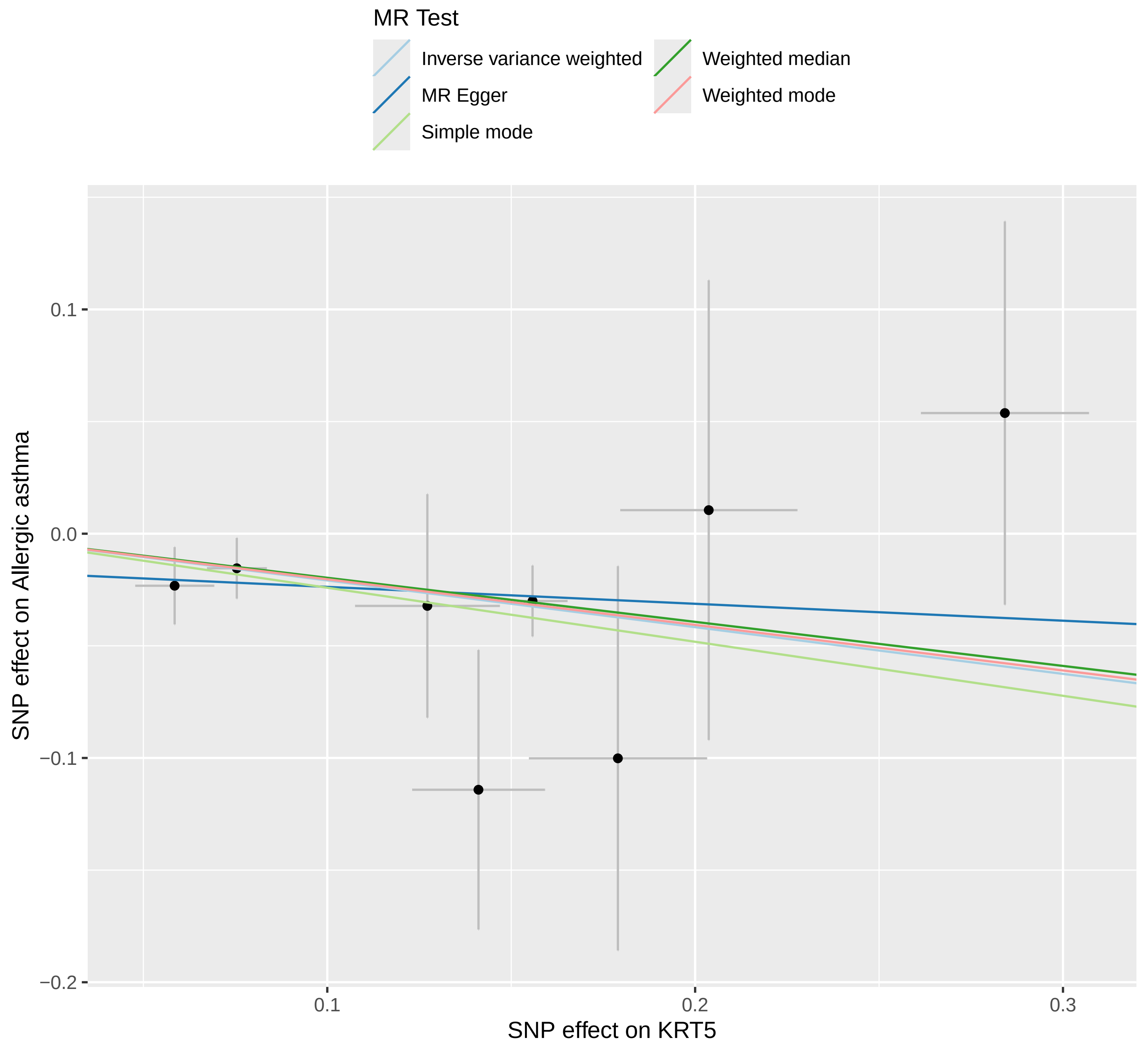
MR Test
Inverse variance weighted
MR Egger
Simple mode
Weighted median
Weighted mode
SNP effect on Allergic asthma
0.1
0.0
−0.1
−0.2
0.1
0.2
0.3
SNP effect on KRT5

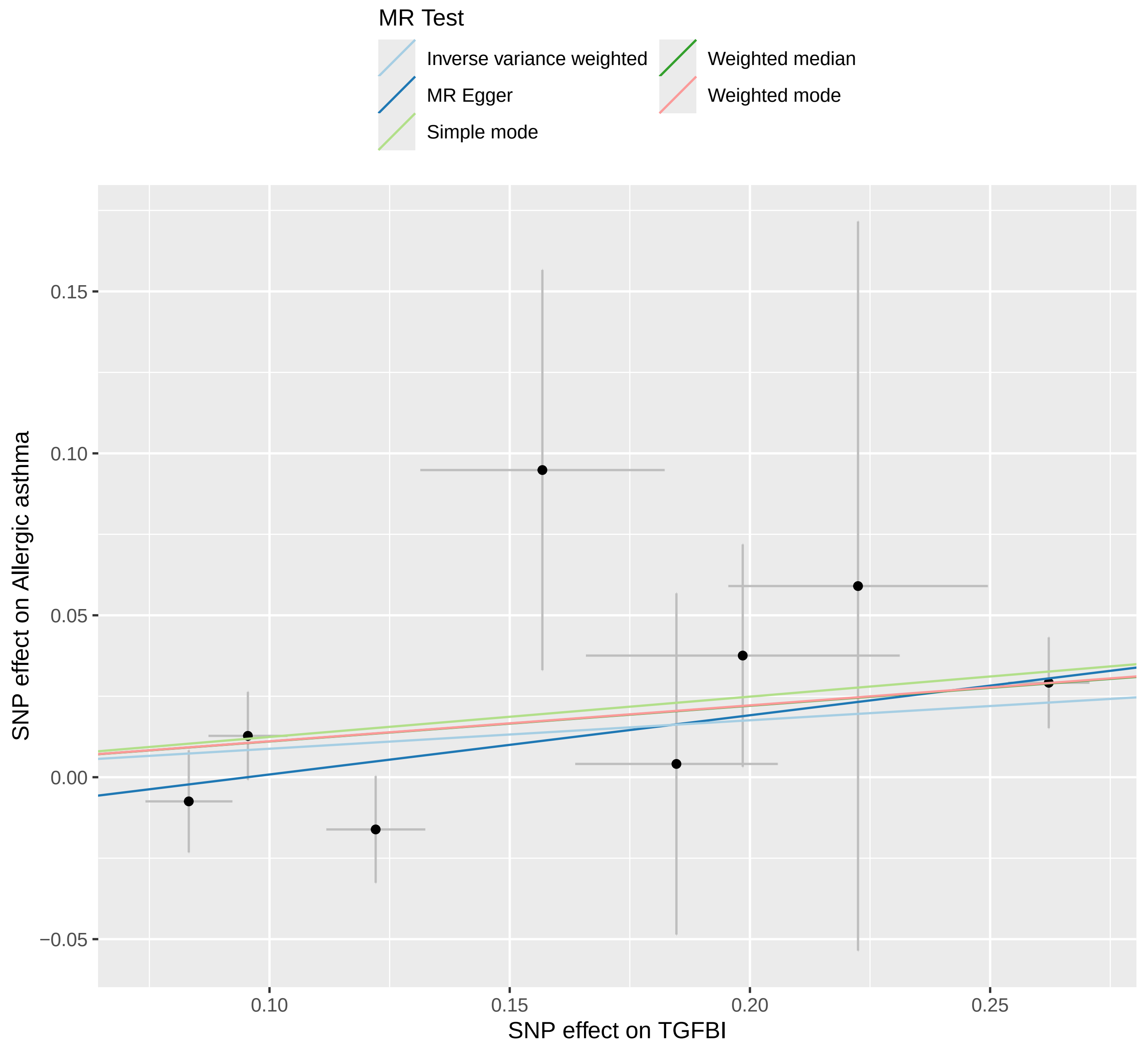
MR Test
Inverse variance weighted
MR Egger
Simple mode
Weighted median
Weighted mode
0.15
0.10
0.05
0.00
−0.05
SNP effect on Allergic asthma
0.10
0.15
0.20
0.25
SNP effect on TGFBI

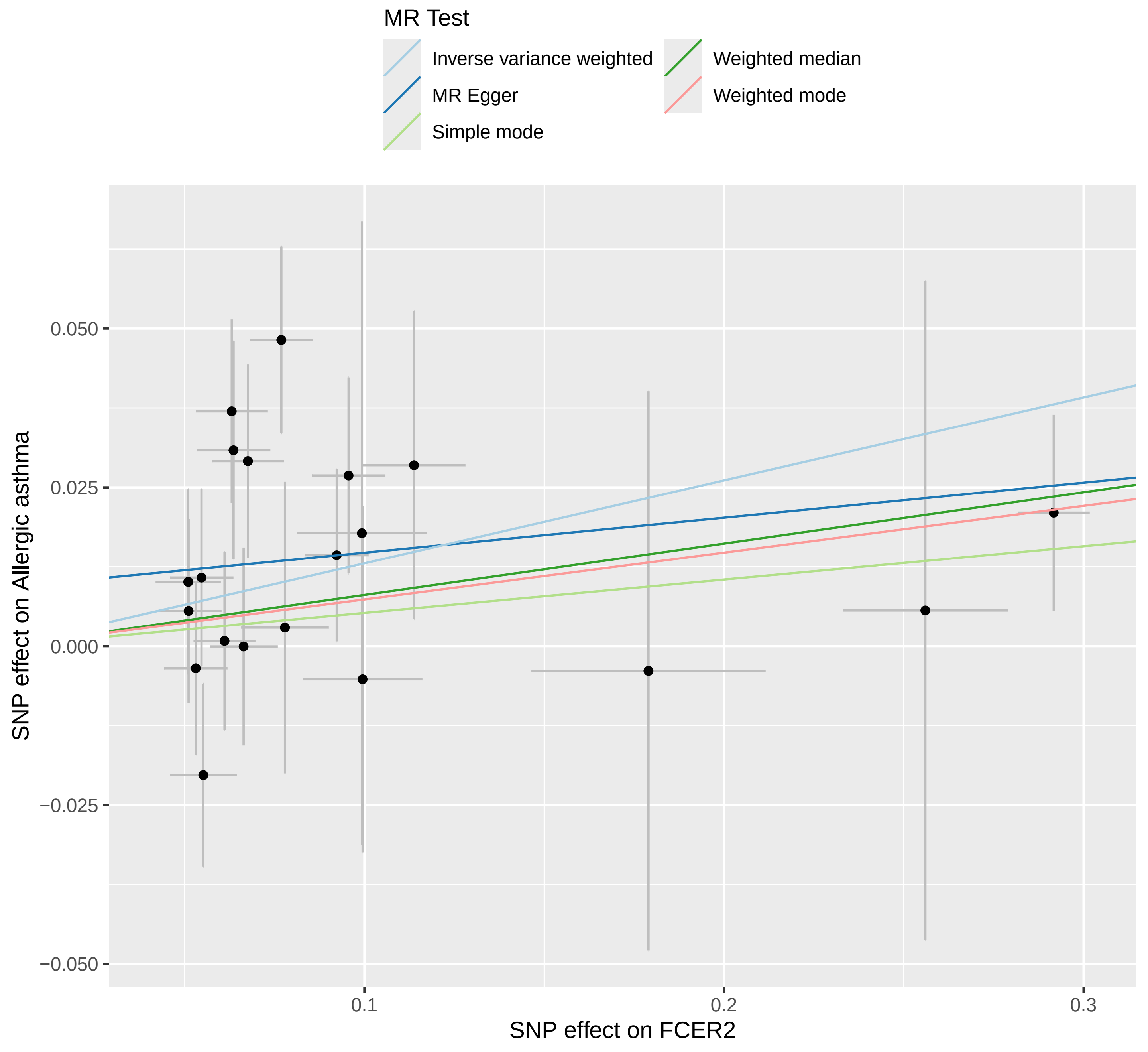
MR Test
Inverse variance weighted
MR Egger
Simple mode
Weighted median
Weighted mode
0.050
0.025
0.000
−0.025
−0.050
SNP effect on Allergic asthma
0.1
0.2
0.3
SNP effect on FCER2

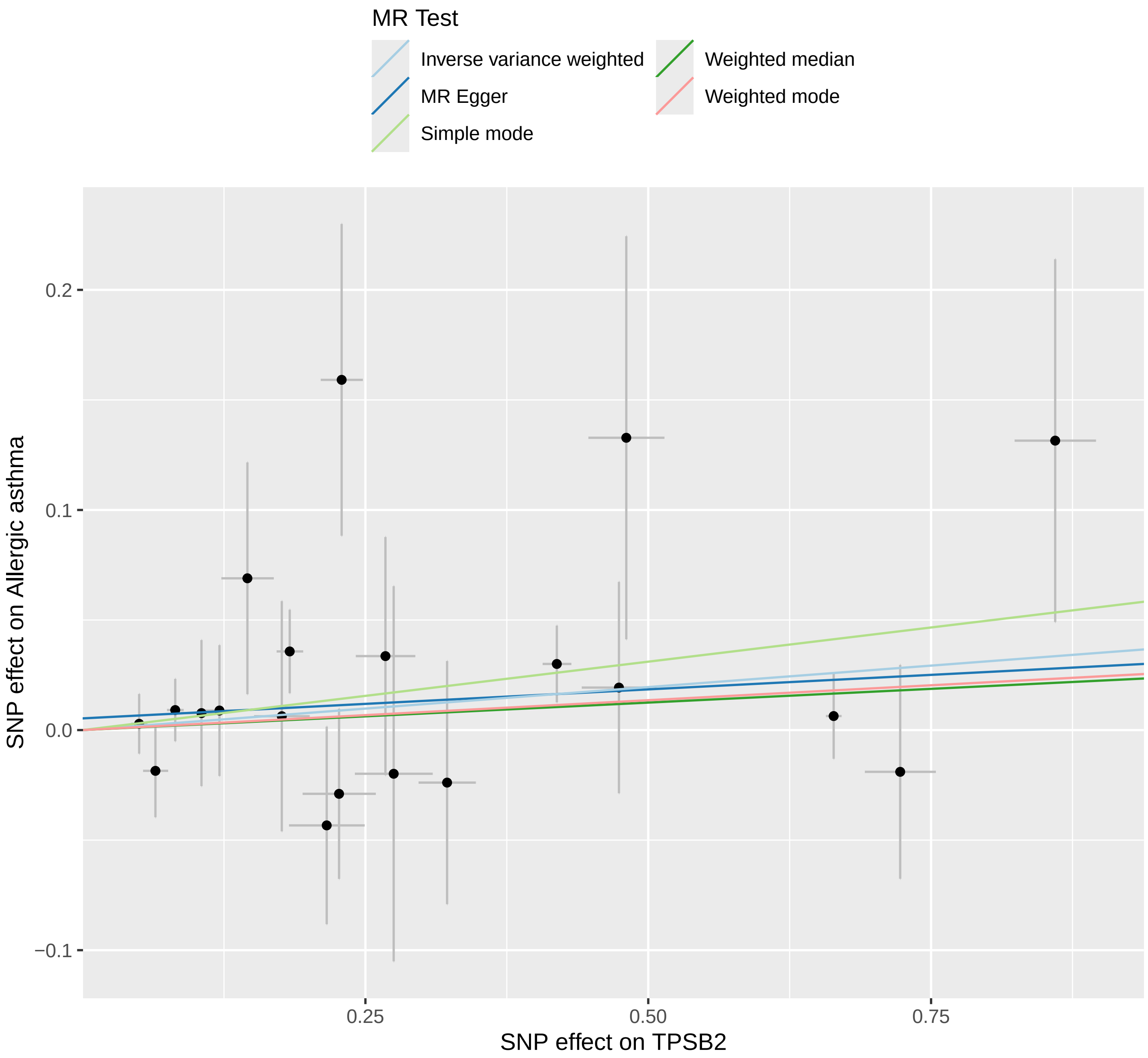

MR Test
Inverse variance weighted
MR Egger
Simple mode
Weighted median
Weighted mode
0.2
0.1
0.0
−0.1
SNP effect on Allergic asthma
0.25
0.50
0.75
SNP effect on TPSB2

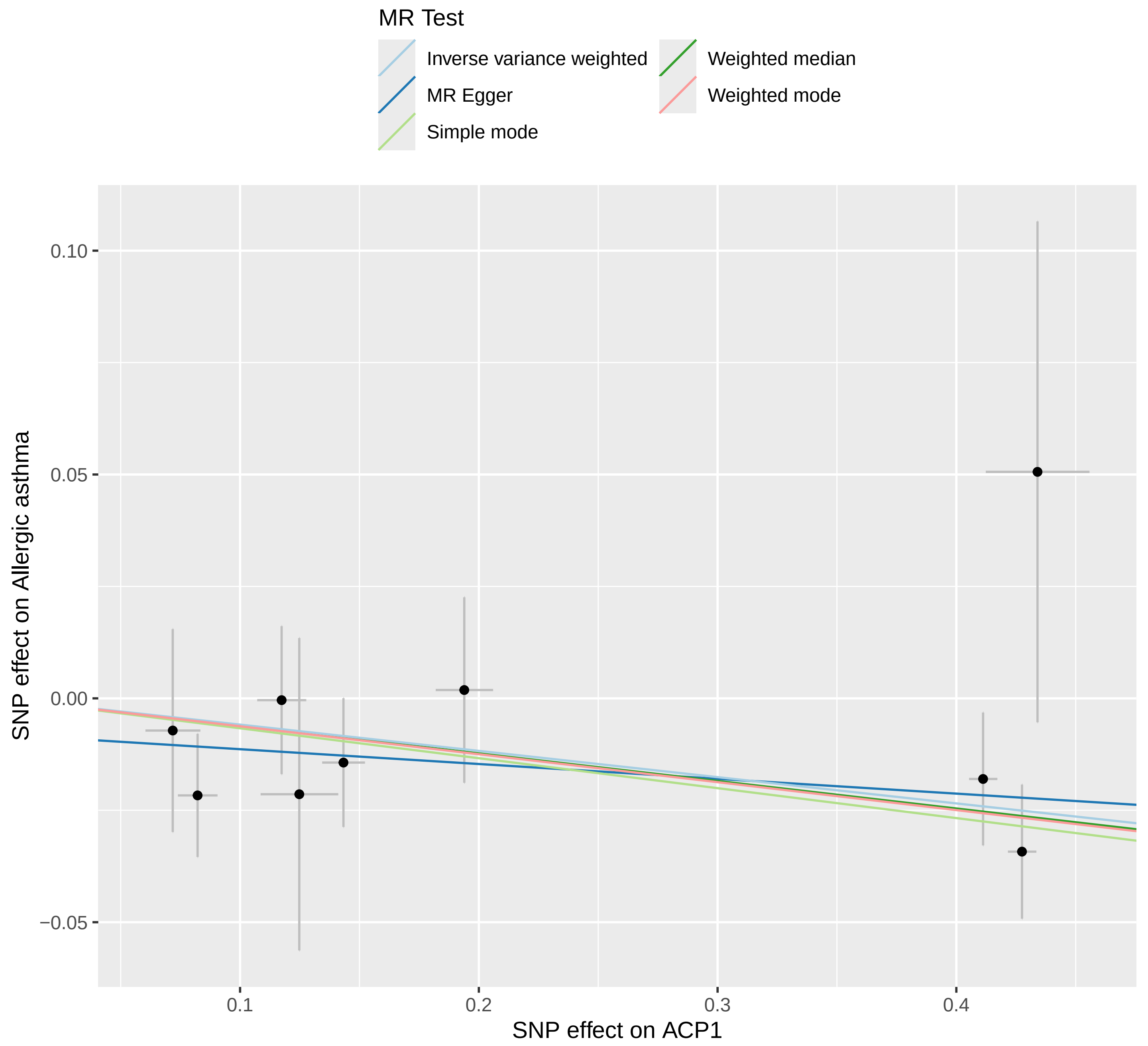
MR Test
Inverse variance weighted
MR Egger
Simple mode
Weighted median
Weighted mode
SNP effect on Allergic asthma
0.10
0.05
0.00
−0.05
0.1
0.2
0.3
0.4
SNP effect on ACP1

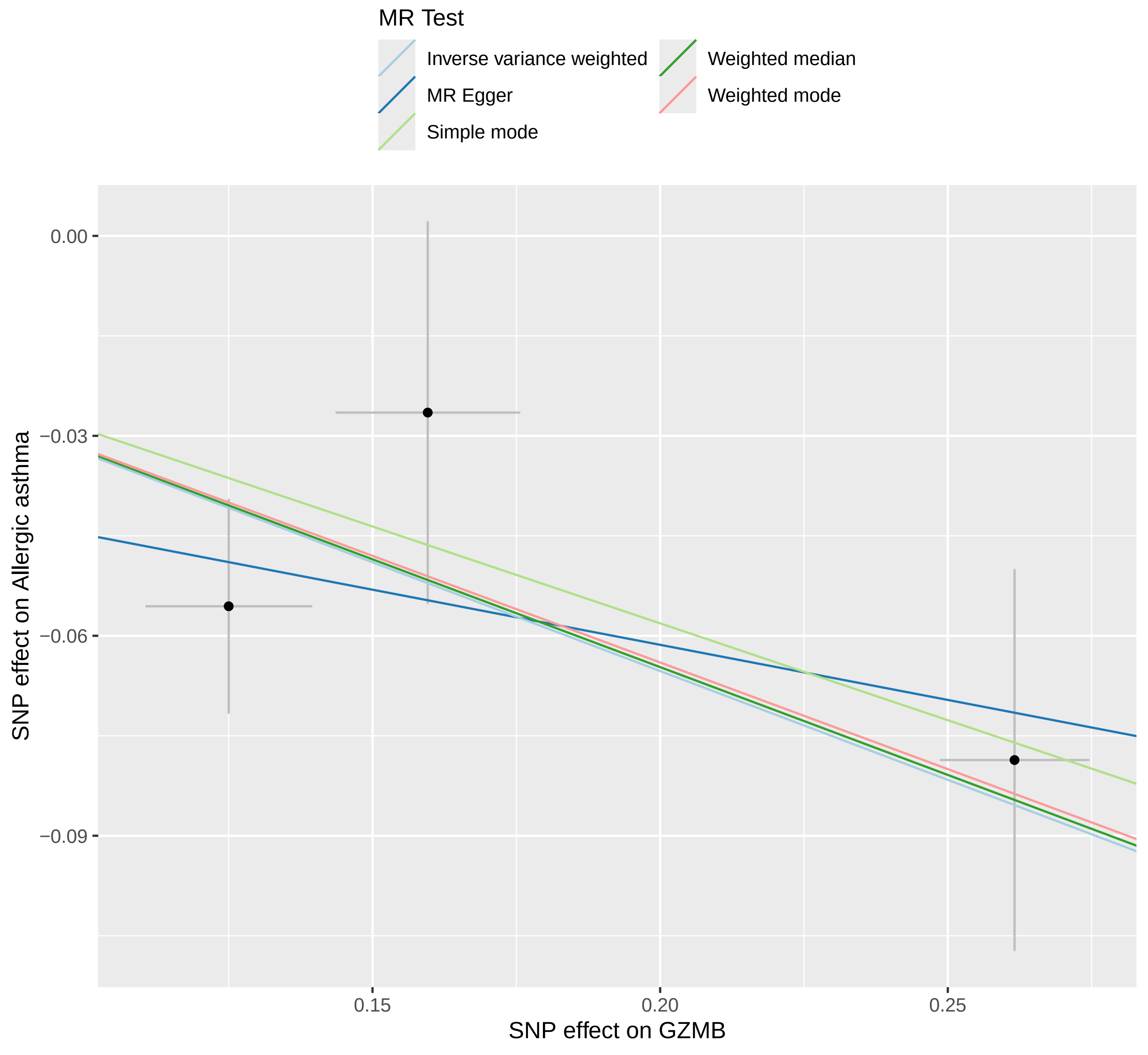

MR Test
Inverse variance weighted
MR Egger
Simple mode
Weighted median
Weighted mode
0.00
−0.03
−0.06
−0.09
SNP effect on Allergic asthma
0.15
0.20
0.25
SNP effect on GZMB

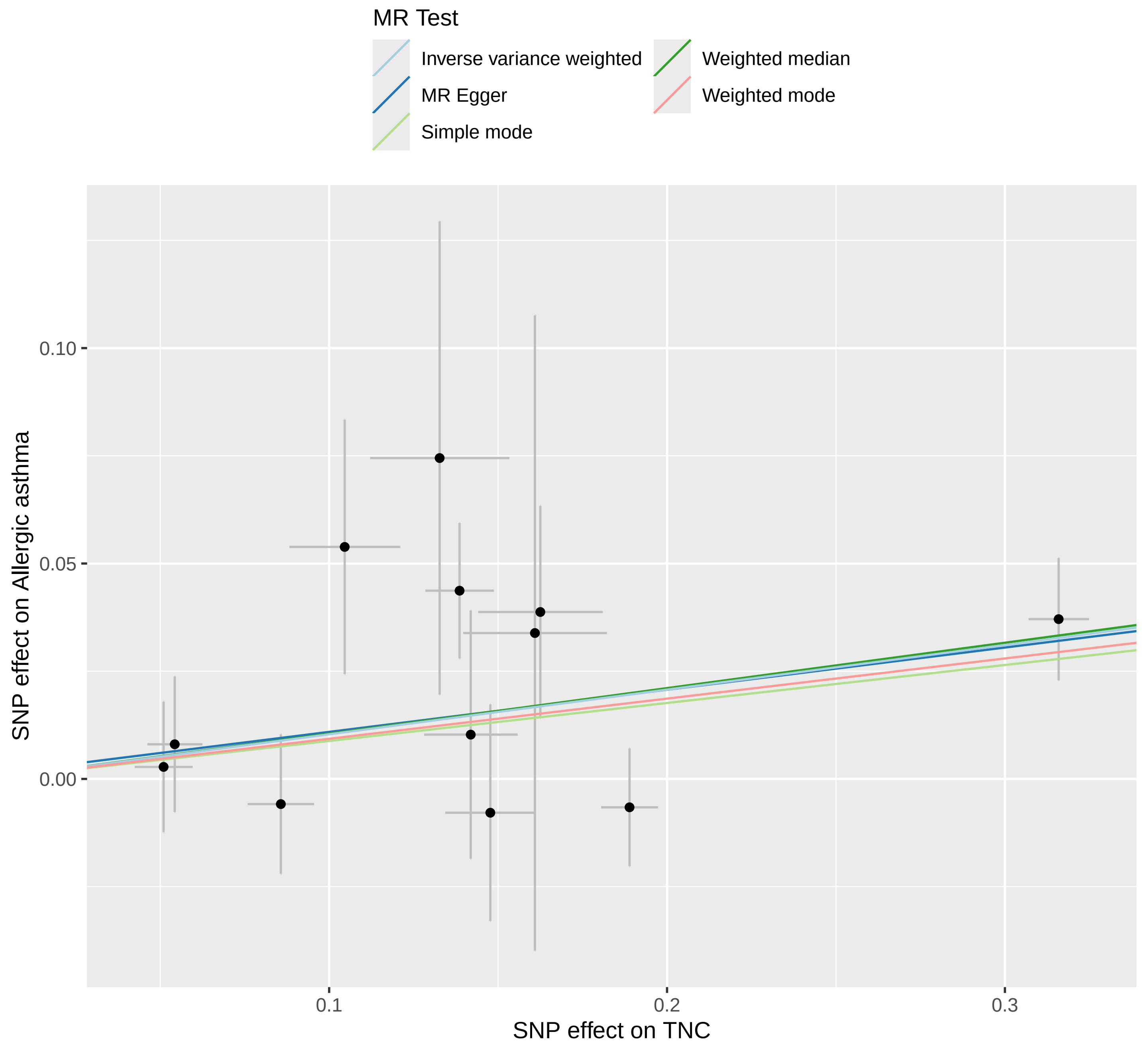
MR Test
Inverse variance weighted
MR Egger
Simple mode
Weighted median
Weighted mode
0.10
0.05
0.00
SNP effect on Allergic asthma
0.1
0.2
0.3
SNP effect on TNC

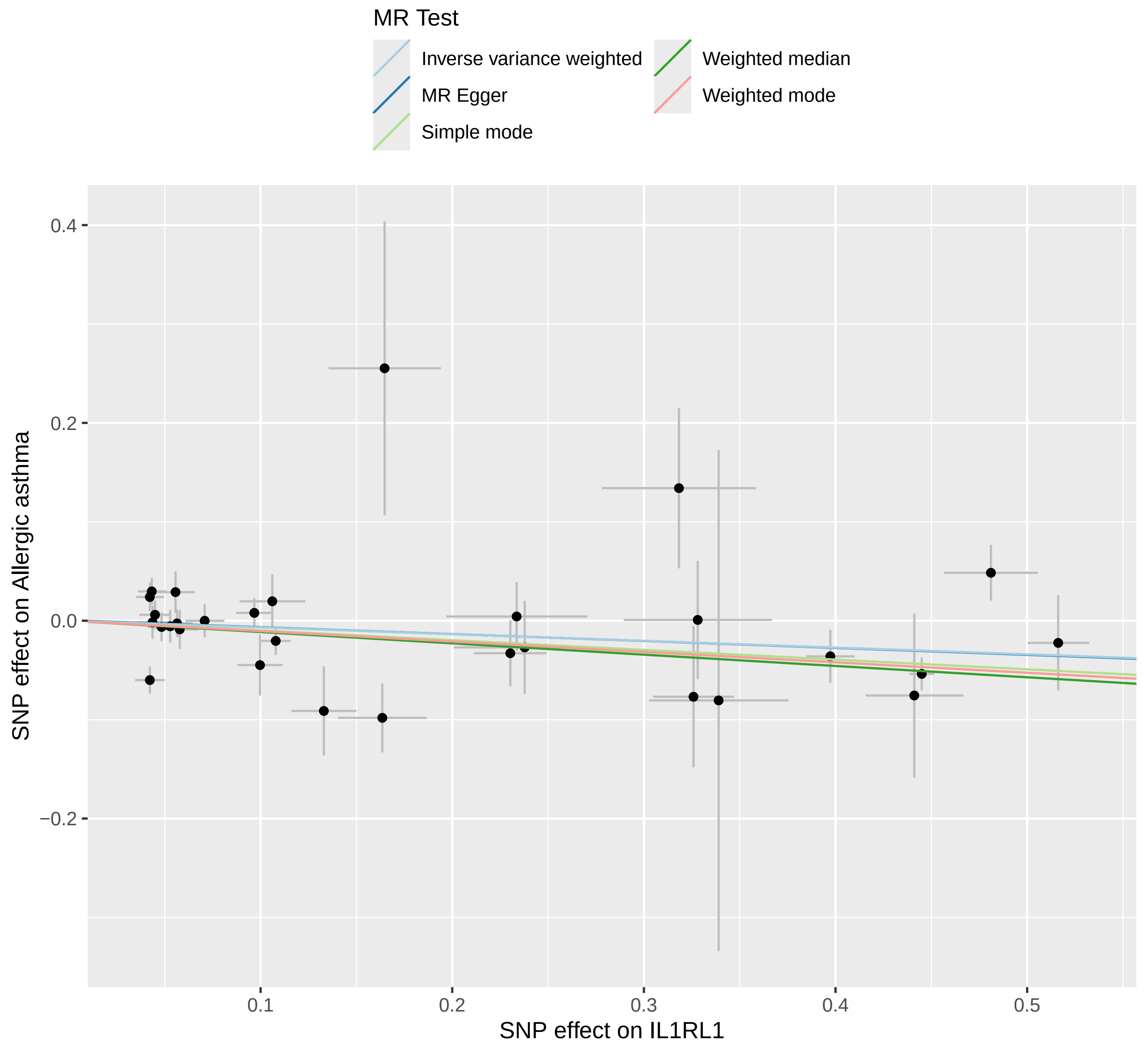
MR Test
Inverse variance weighted
MR Egger
Simple mode
Weighted median
Weighted mode
SNP effect on Allergic asthma
0.4
0.2
0.0
−0.2
0.1
0.2
0.3
0.4
0.5
SNP effect on IL1RL1

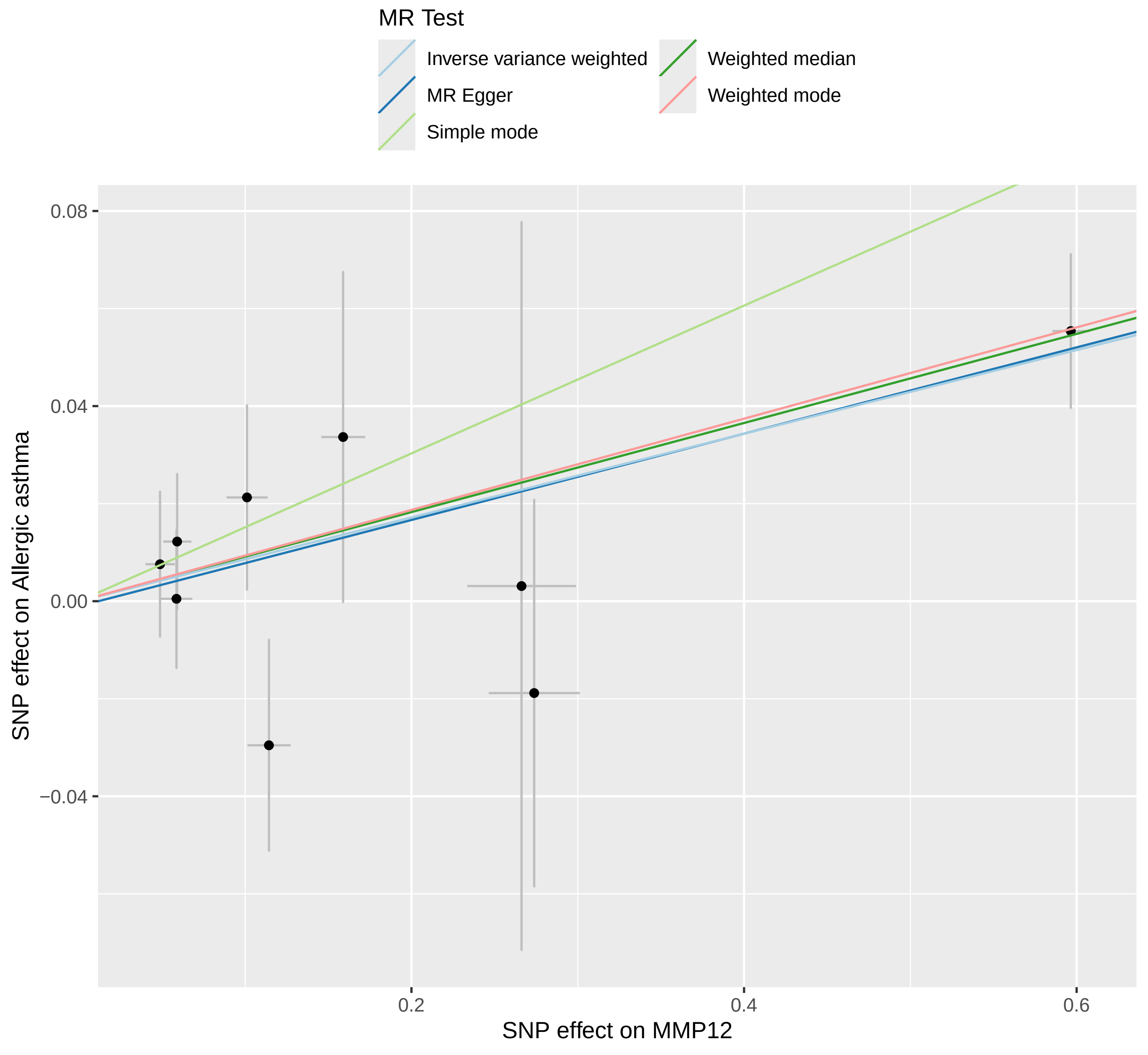

MR Test
Inverse variance weighted
MR Egger
Simple mode
Weighted median
Weighted mode
0.08
0.04
0.00
−0.04
0.2
0.4
0.6
SNP effect on MMP12
SNP effect on Allergic asthma

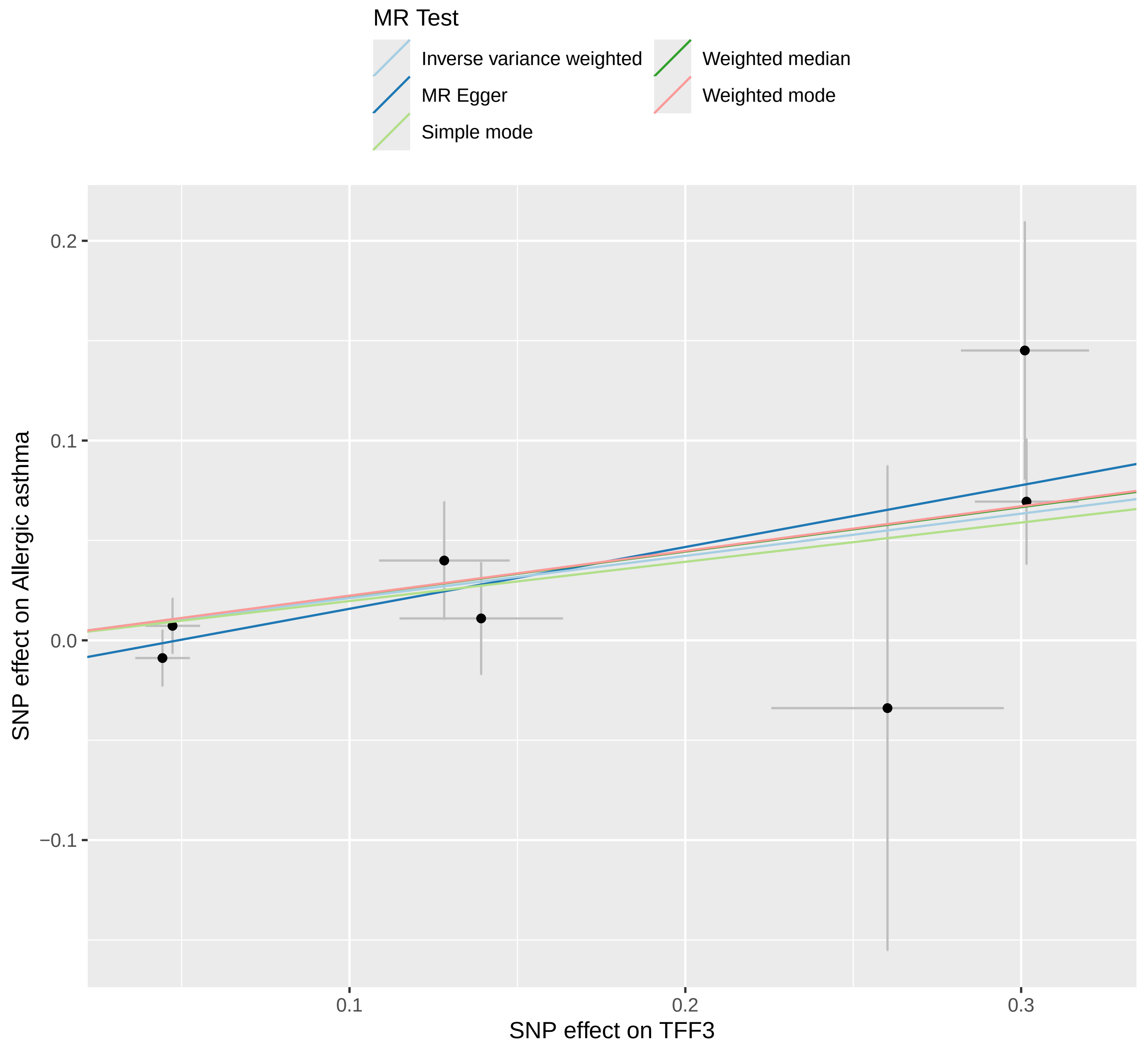
MR Test
Inverse variance weighted
MR Egger
Simple mode
Weighted median
Weighted mode
0.2
0.1
0.0
−0.1
0.1
0.2
0.3
SNP effect on Allergic asthma
SNP effect on TFF3

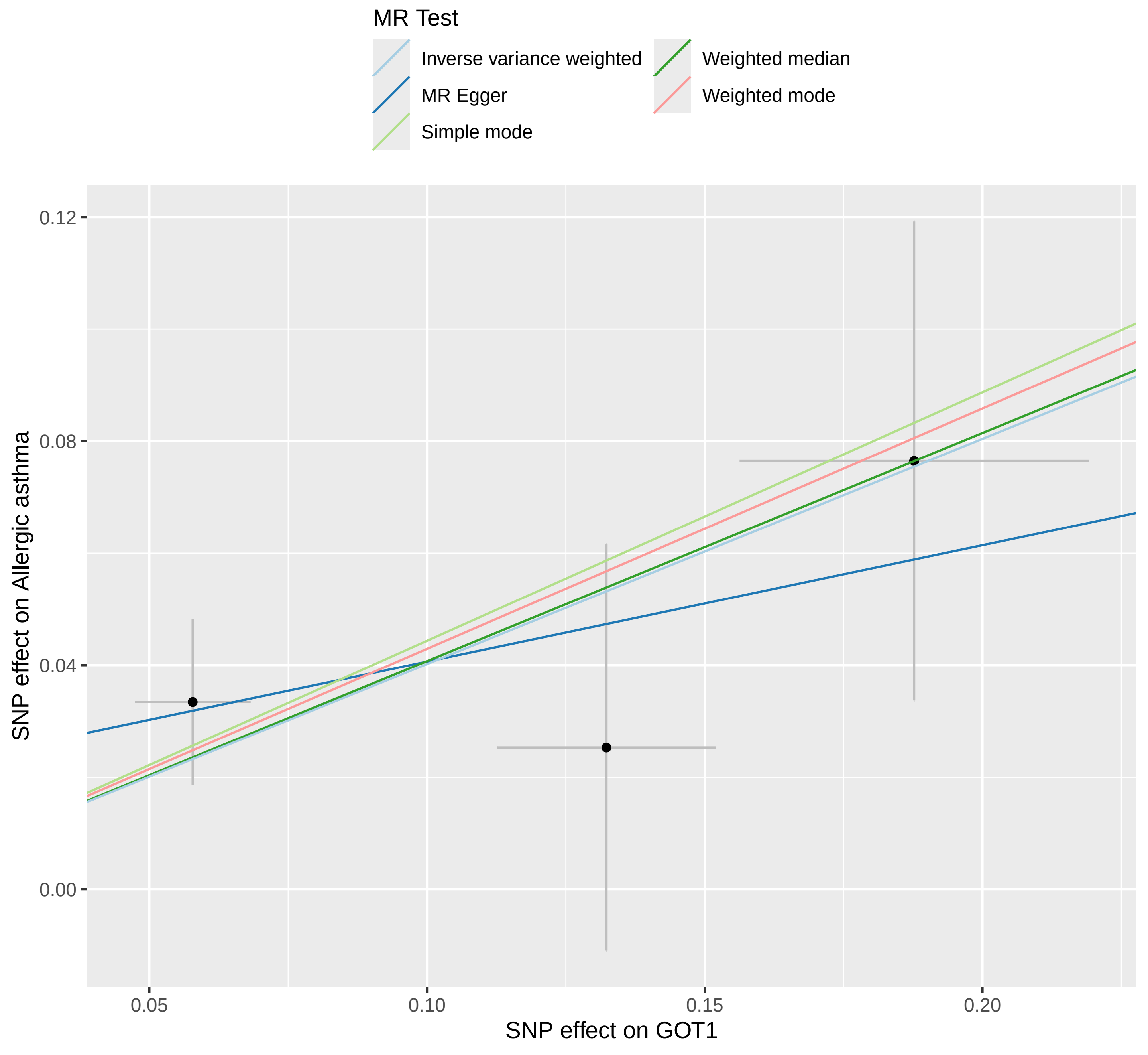

MR Test
Inverse variance weighted
MR Egger
Simple mode
Weighted median
Weighted mode
0.12
0.08
0.04
0.00
SNP effect on Allergic asthma
0.05
0.10
0.15
0.20
SNP effect on GOT1

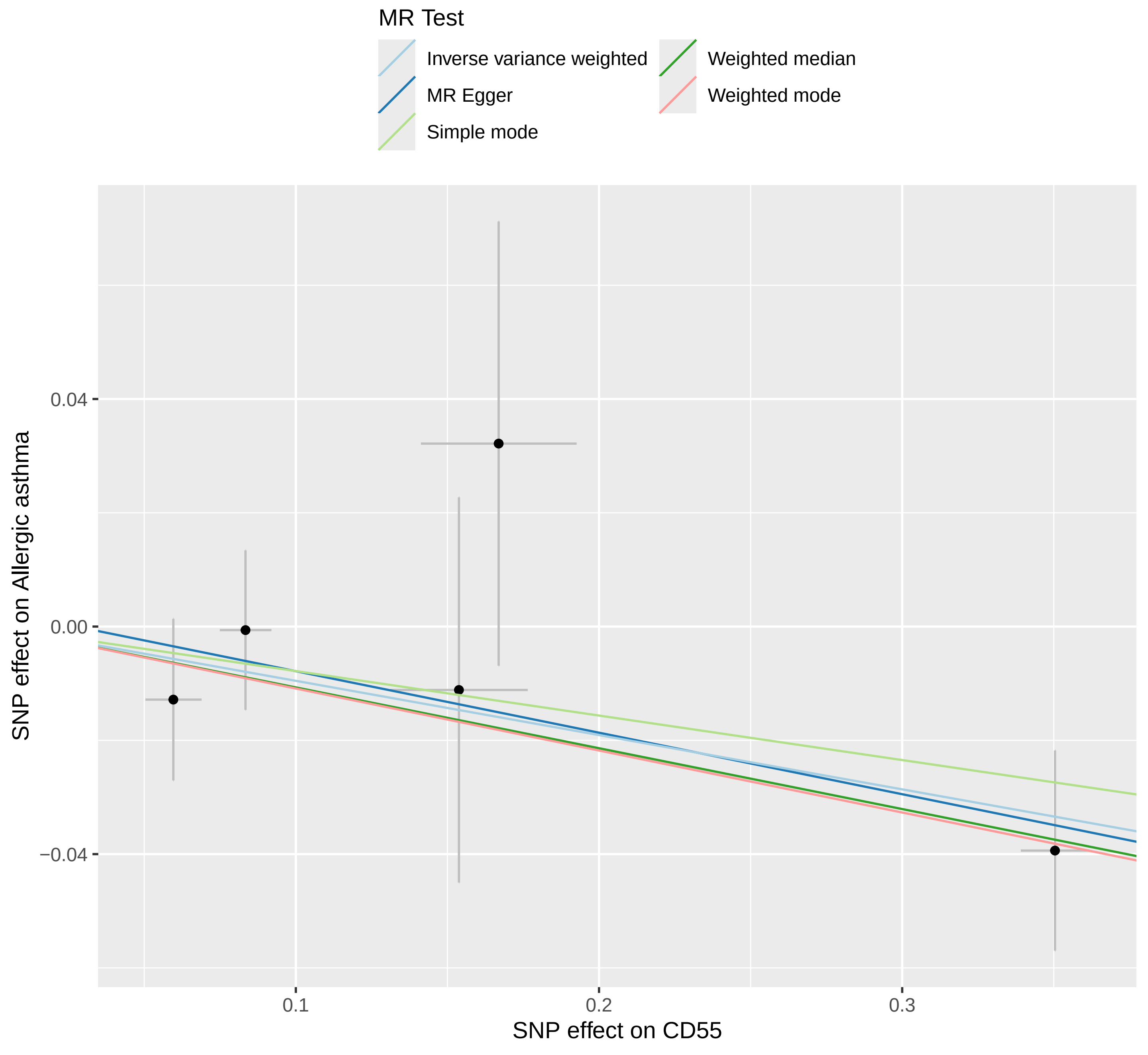

MR Test
Inverse variance weighted
MR Egger
Simple mode
Weighted median
Weighted mode
0.04
0.00
−0.04
SNP effect on Allergic asthma
0.1
0.2
0.3
SNP effect on CD55

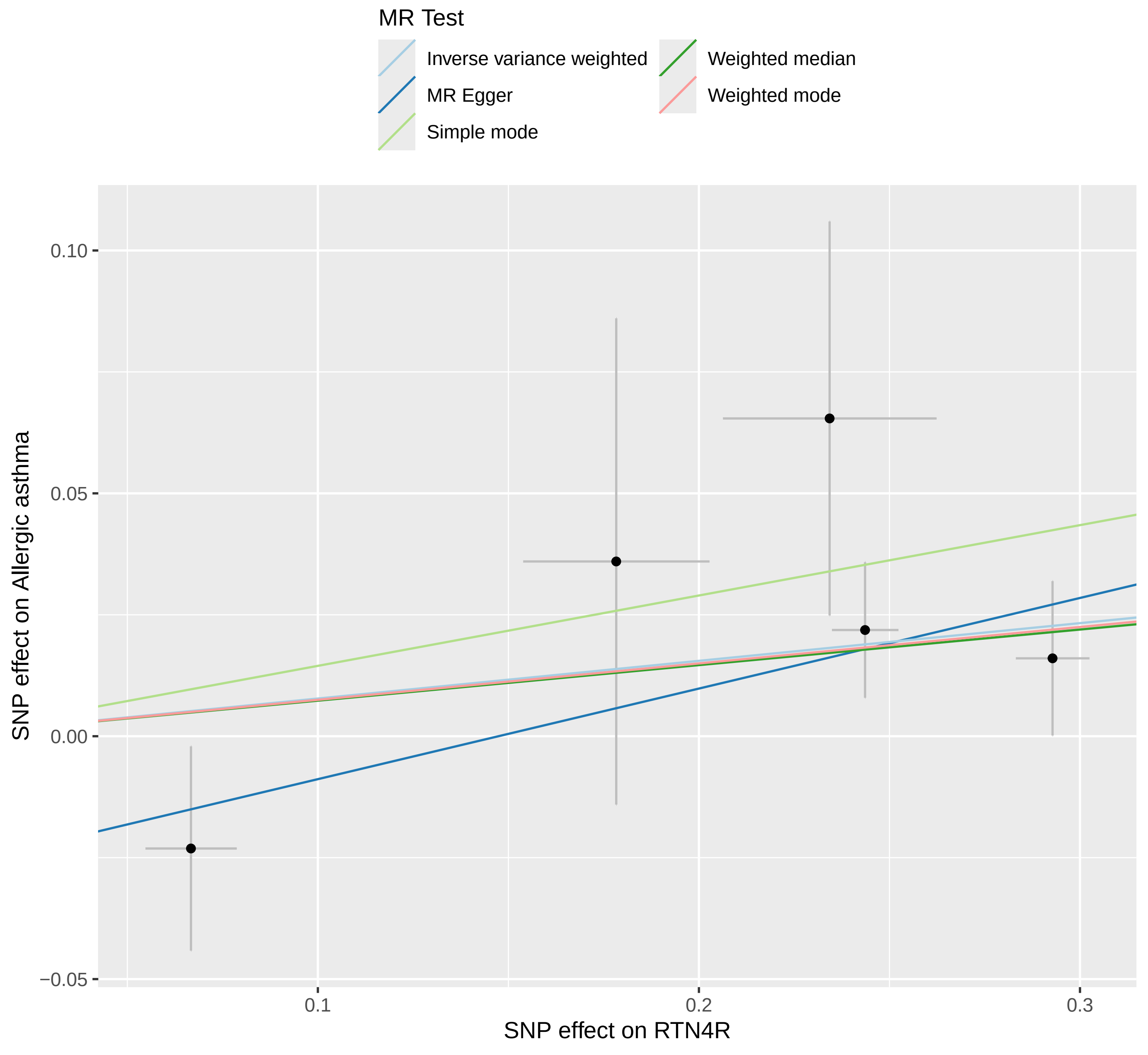
MR Test
Inverse variance weighted
MR Egger
Simple mode
Weighted median
Weighted mode
0.10
0.05
0.00
−0.05
0.1
0.2
0.3
SNP effect on Allergic asthma
SNP effect on RTN4R

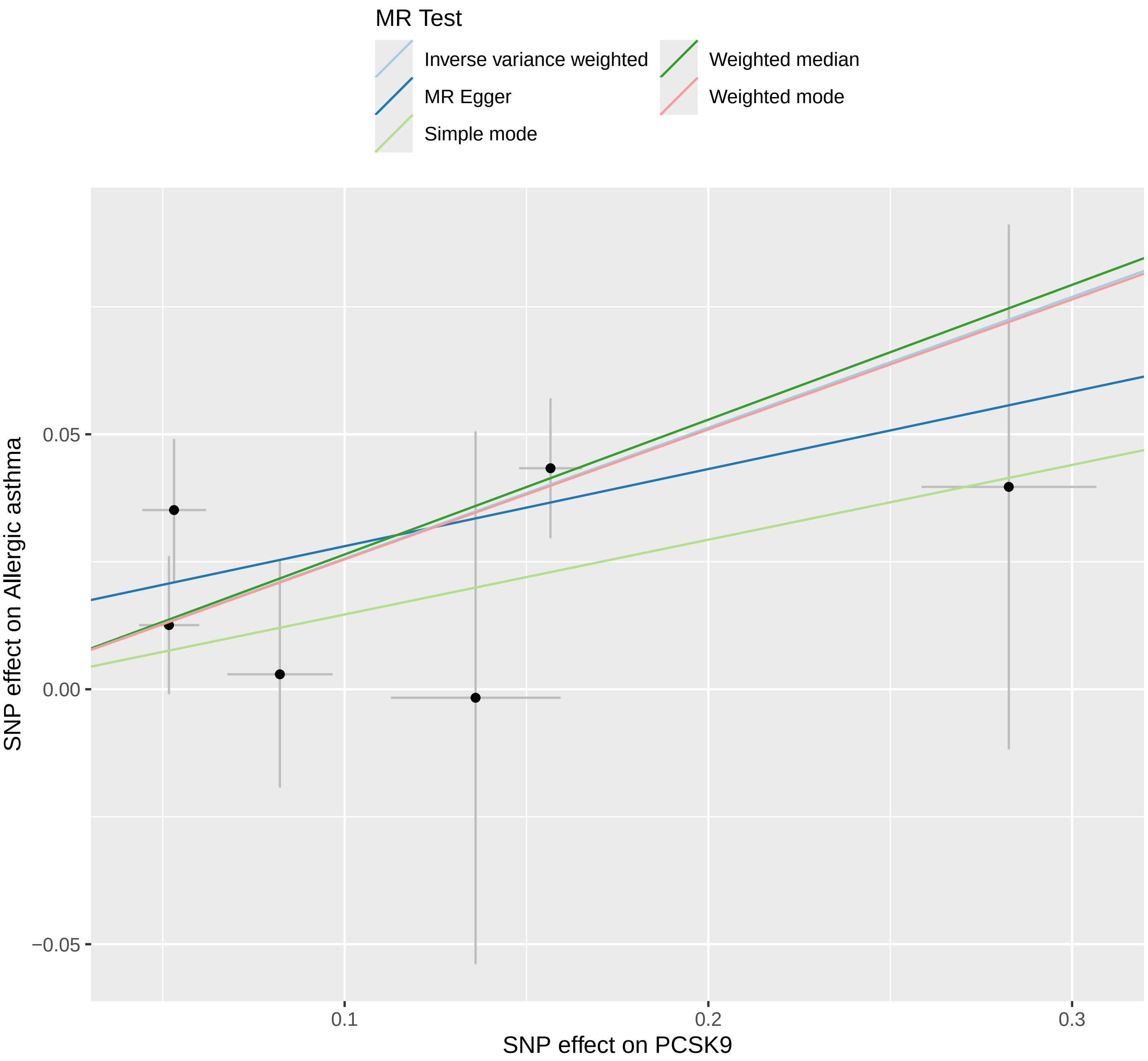

MR Test
Inverse variance weighted
MR Egger
Simple mode
Weighted median
Weighted mode
0.05
0.00
−0.05
0.1
0.2
0.3
SNP effect on Allergic asthma
SNP effect on PCSK9

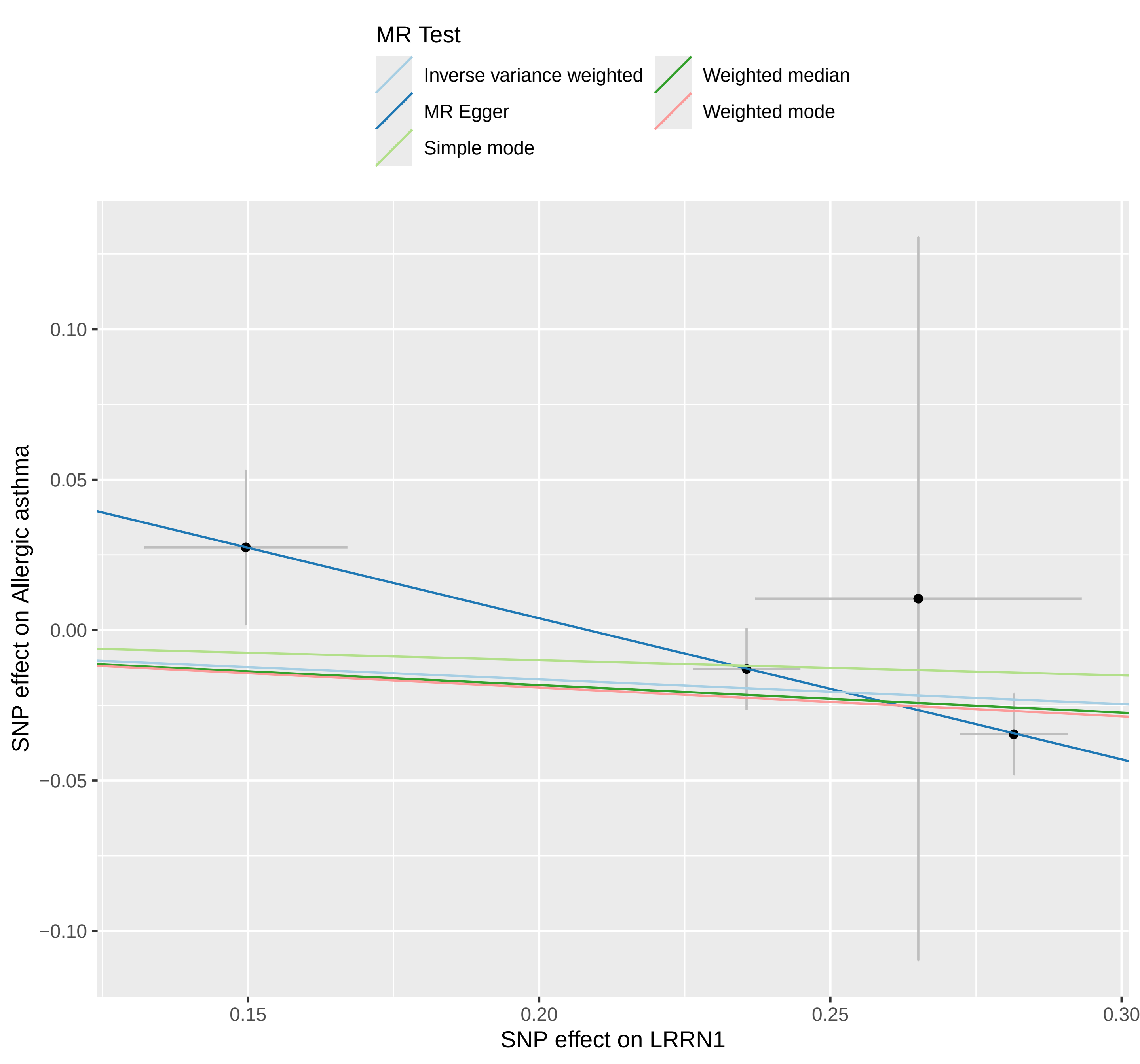
MR Test
Inverse variance weighted
MR Egger
Simple mode
Weighted median
Weighted mode
SNP effect on Allergic asthma
0.10
0.05
0.00
−0.05
−0.10
0.15
0.20
0.25
0.30
SNP effect on LRRN1

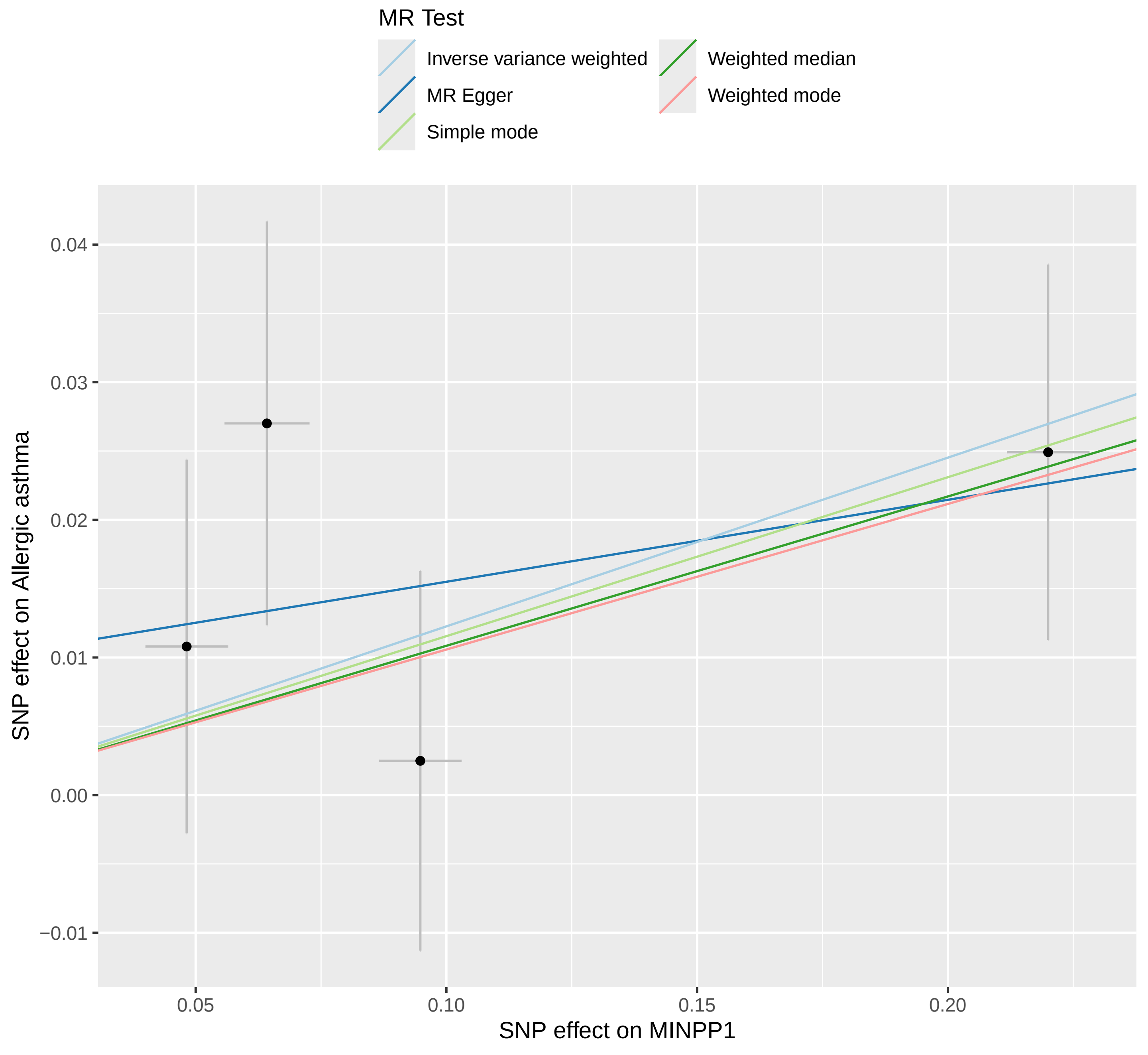

MR Test
Inverse variance weighted
MR Egger
Simple mode
Weighted median
Weighted mode
0.04
0.03
0.02
0.01
0.00
−0.01
0.05
0.10
0.15
0.20
SNP effect on Allergic asthma
SNP effect on MINPP1

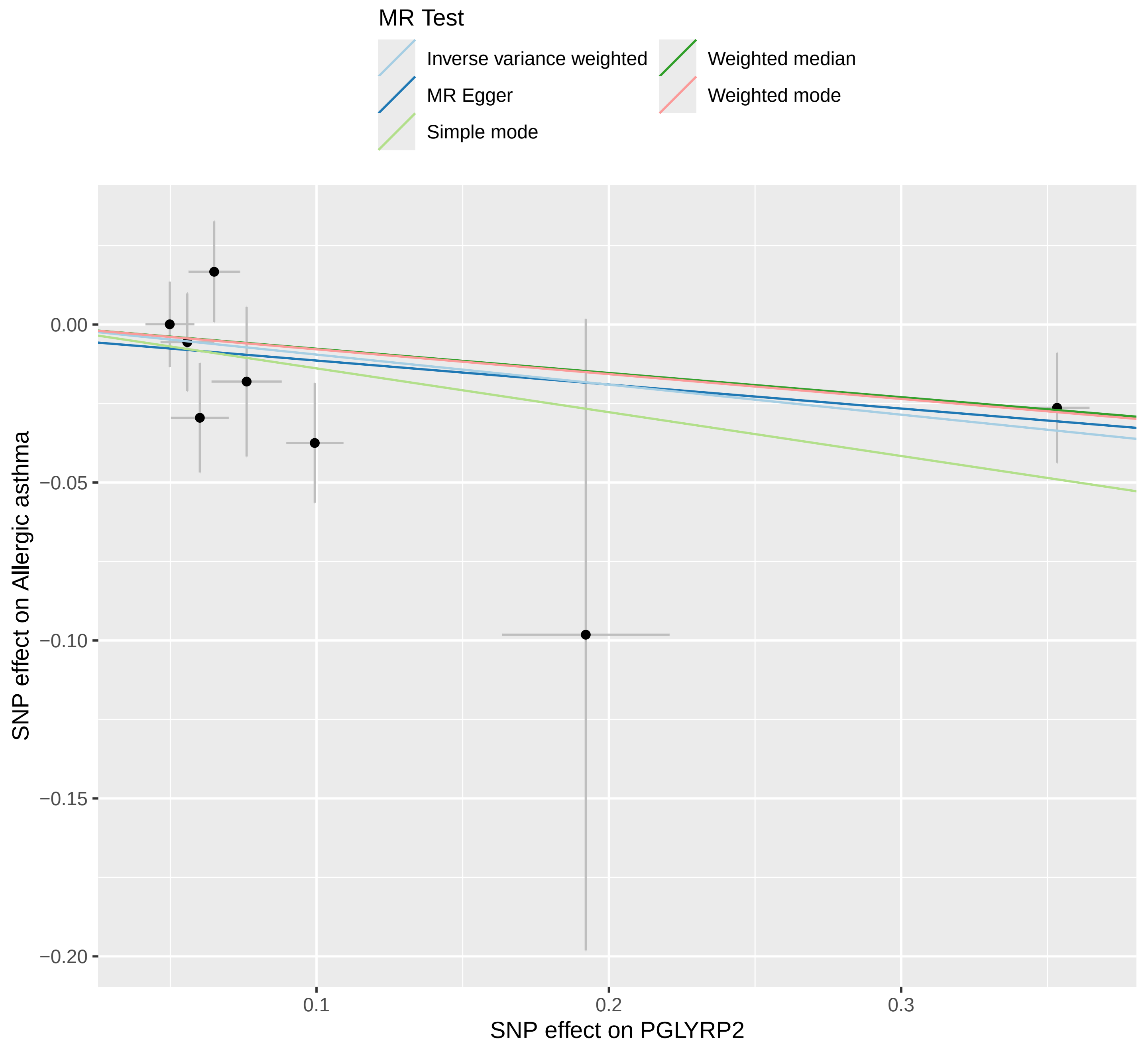
MR Test
Inverse variance weighted
MR Egger
Simple mode
Weighted median
Weighted mode
0.00
−0.05
−0.10
−0.15
−0.20
SNP effect on Allergic asthma
0.1
0.2
0.3
SNP effect on PGLYRP2

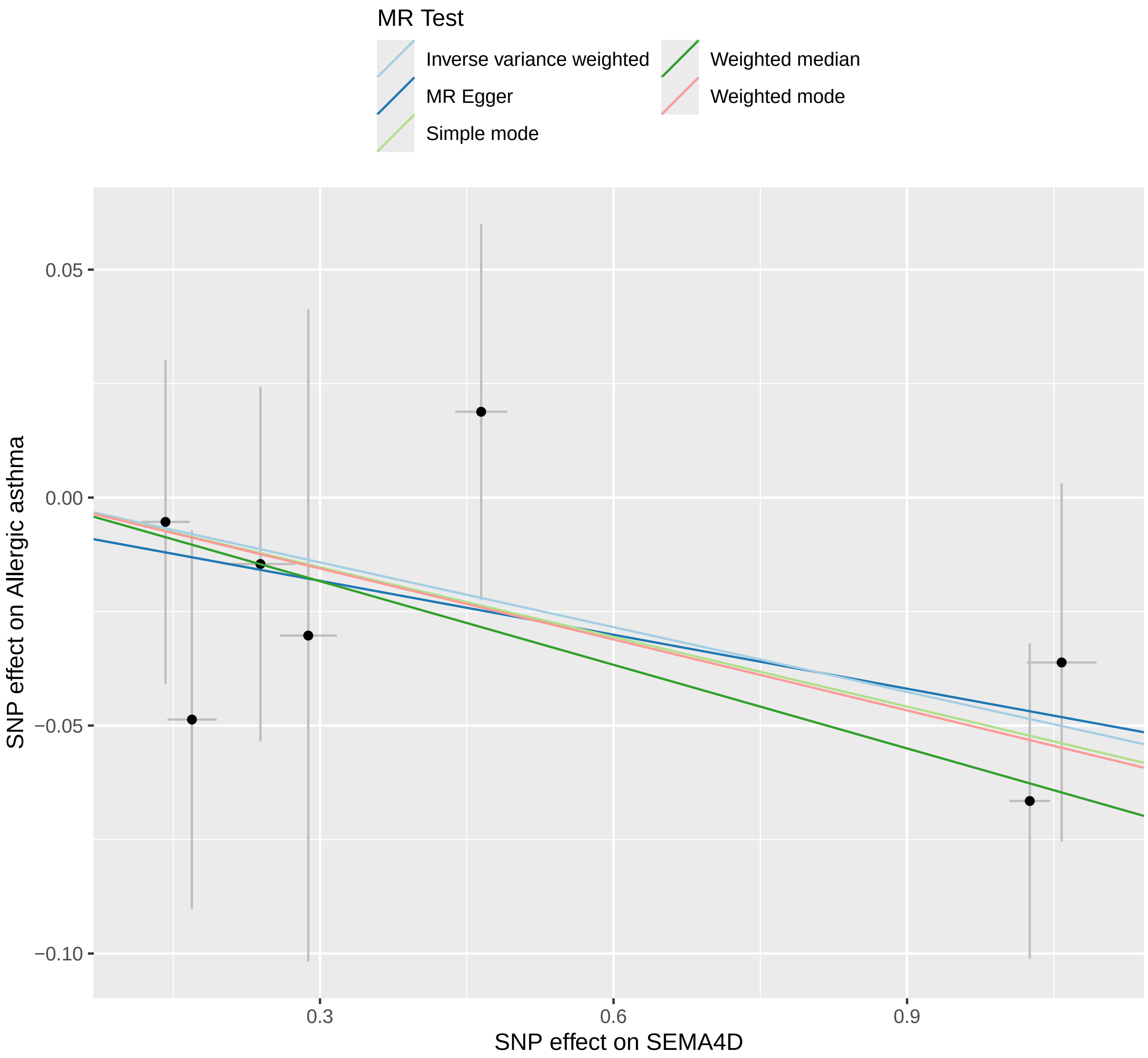
MR Test
Inverse variance weighted
MR Egger
Simple mode
Weighted median
Weighted mode
0.05
0.00
−0.05
−0.10
SNP effect on Allergic asthma
0.3
0.6
0.9
SNP effect on SEMA4D

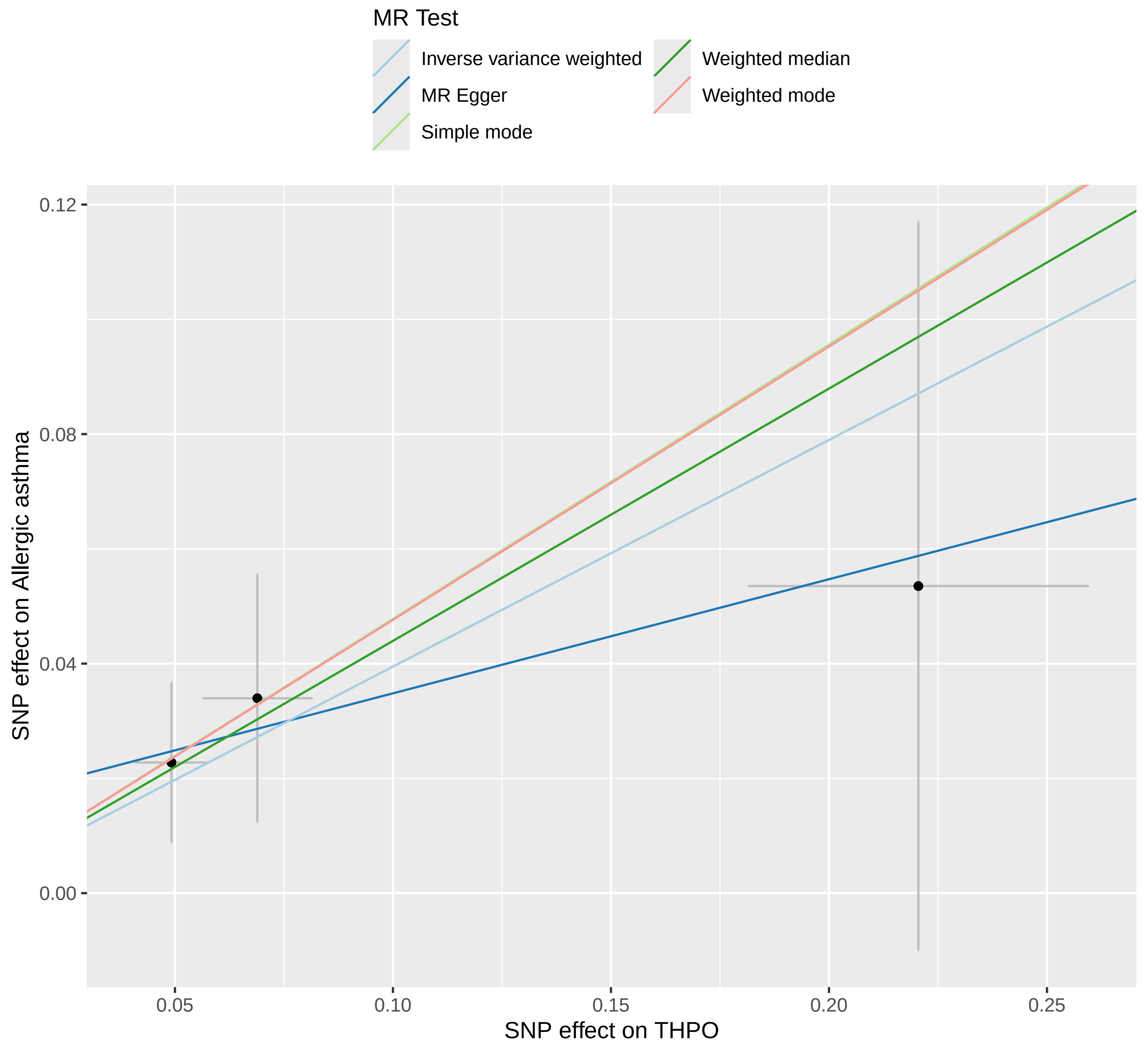
MR Test
Inverse variance weighted
MR Egger
Simple mode
Weighted median
Weighted mode
0.12
0.08
0.04
0.00
SNP effect on Allergic asthma
0.05
0.10
0.15
0.20
0.25
SNP effect on THPO

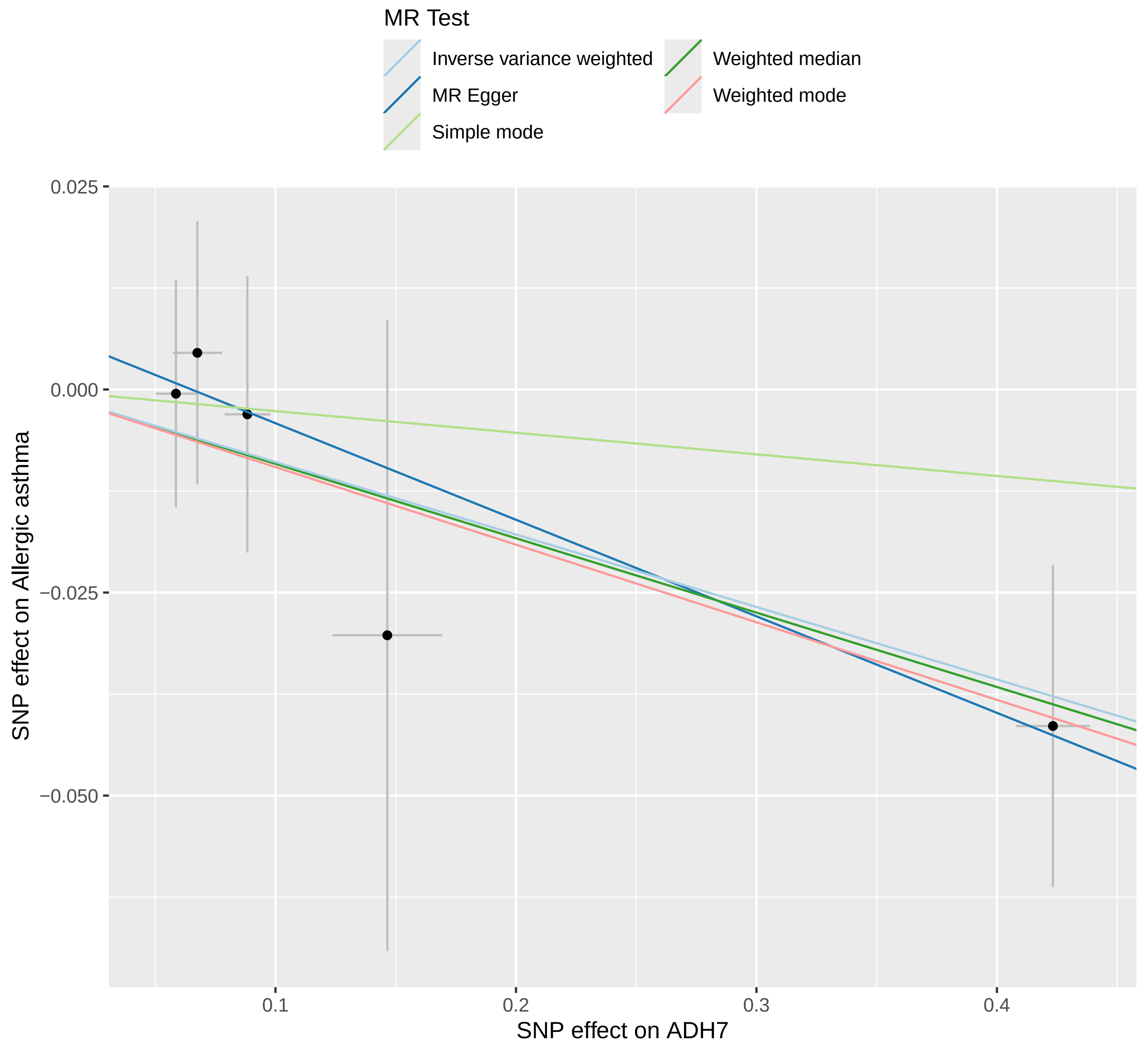
MR Test
Inverse variance weighted
MR Egger
Simple mode
Weighted median
Weighted mode
0.025
0.000
−0.025
−0.050
0.1
0.2
0.3
0.4
SNP effect on Allergic asthma
SNP effect on ADH7

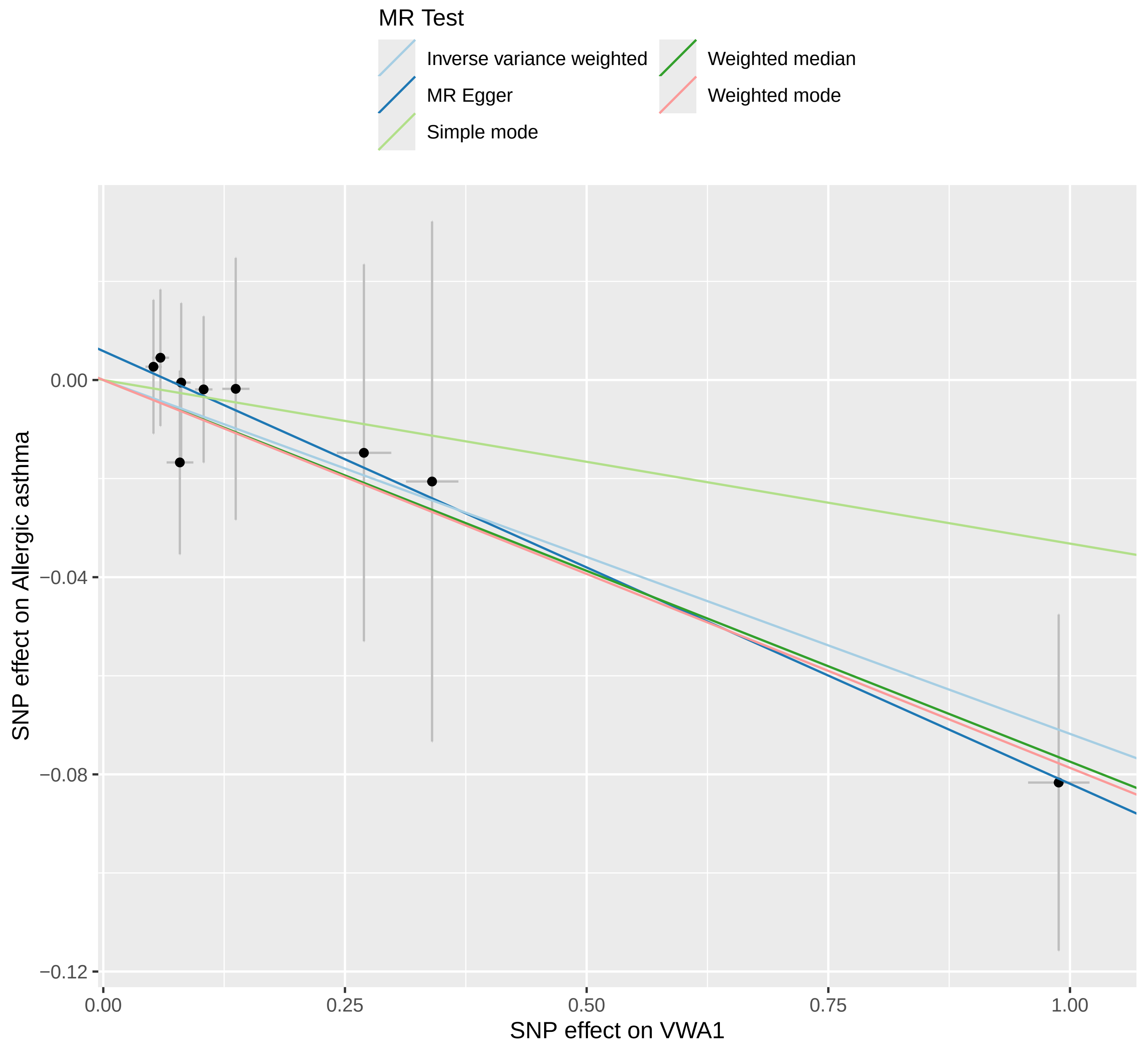

MR Test
Inverse variance weighted
MR Egger
Simple mode
Weighted median
Weighted mode
0.00
−0.04
−0.08
−0.12
0.00
0.25
0.50
0.75
1.00
SNP effect on Allergic asthma
SNP effect on VWA1

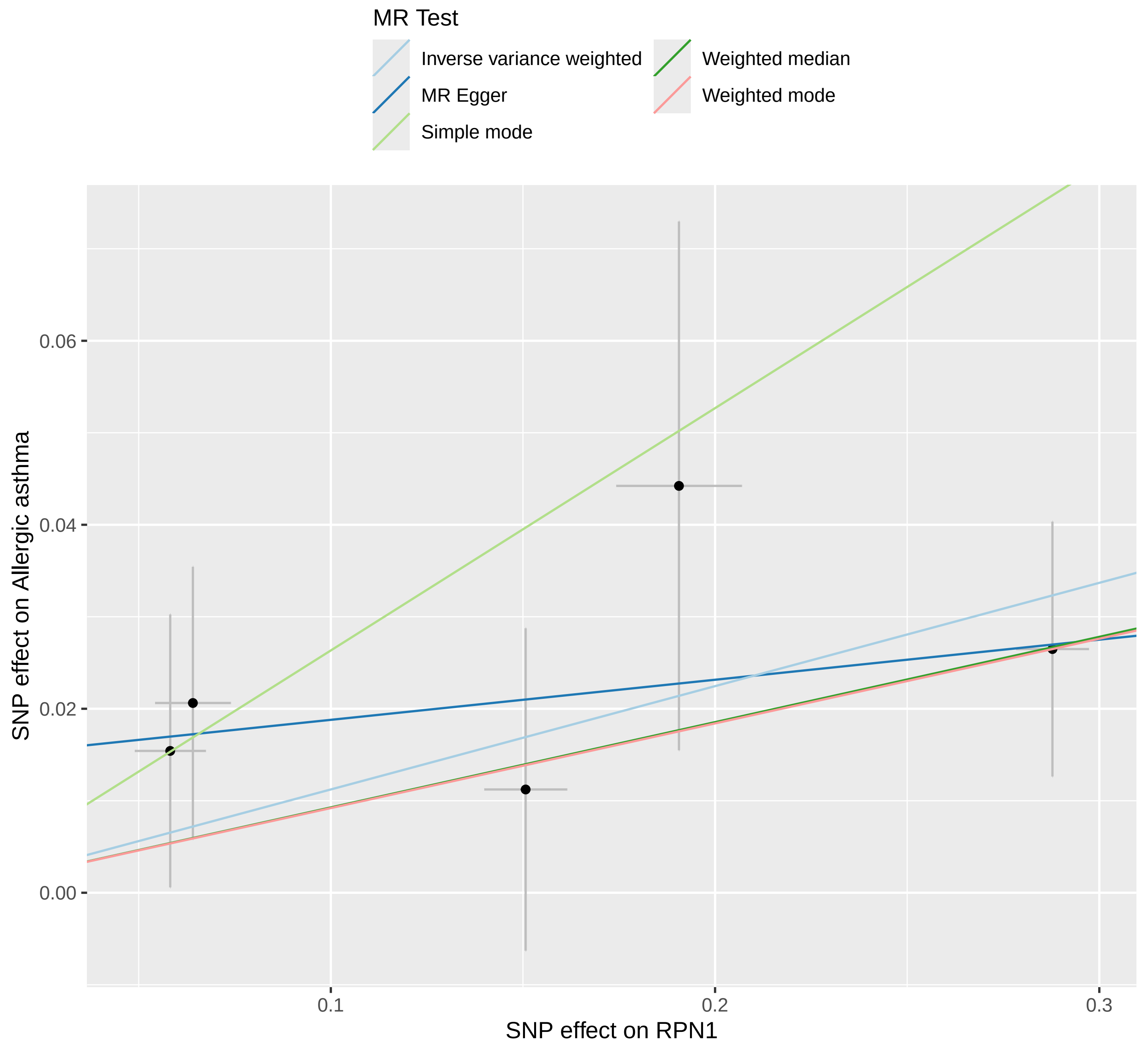
MR Test
Inverse variance weighted
MR Egger
Simple mode
Weighted median
Weighted mode
0.06
0.04
0.02
0.00
SNP effect on Allergic asthma
0.1
0.2
0.3
SNP effect on RPN1

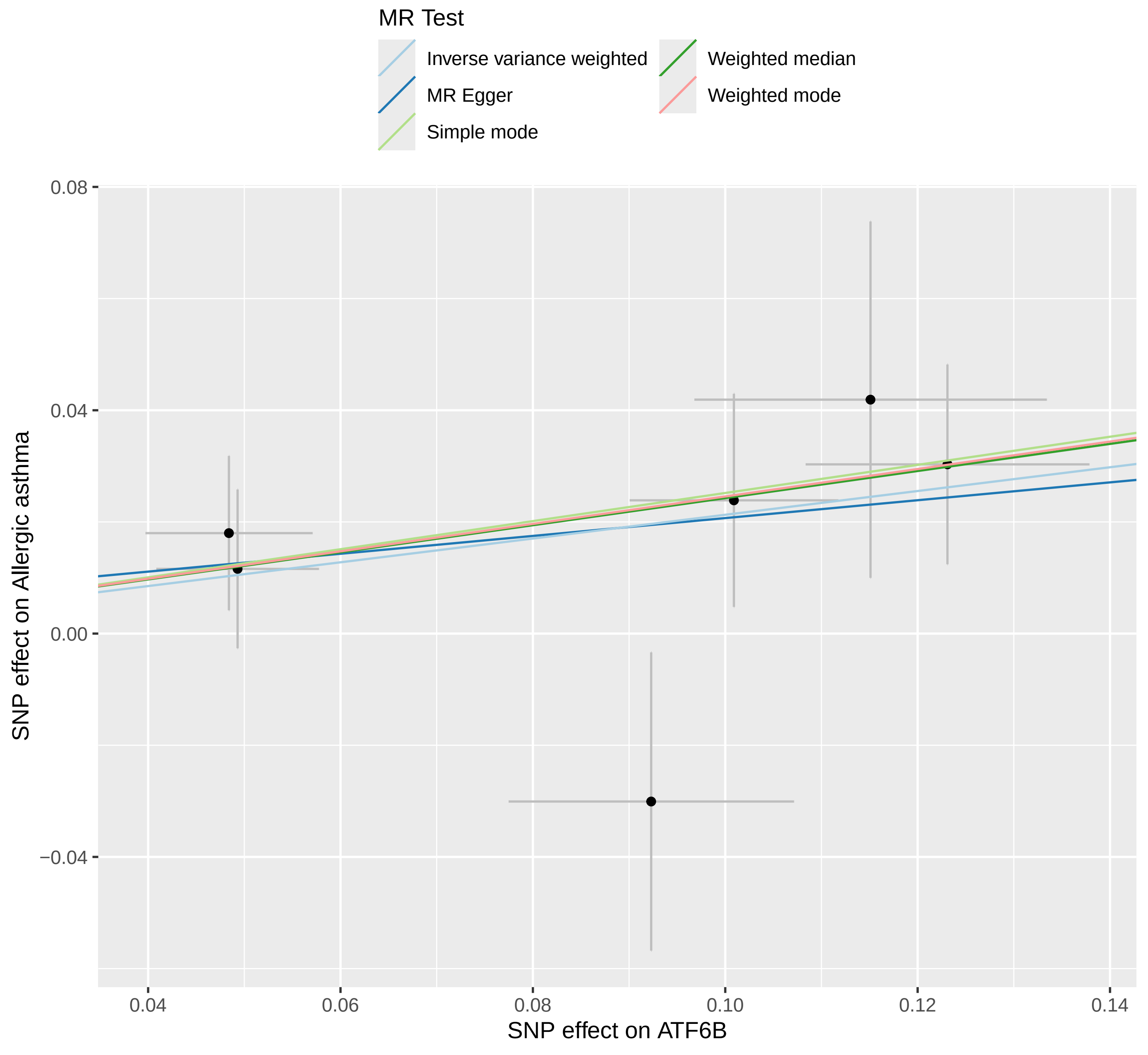
MR Test
Inverse variance weighted
MR Egger
Simple mode
Weighted median
Weighted mode
0.08
0.04
0.00
−0.04
SNP effect on Allergic asthma
0.04
0.06
0.08
0.10
0.12
0.14
SNP effect on ATF6B

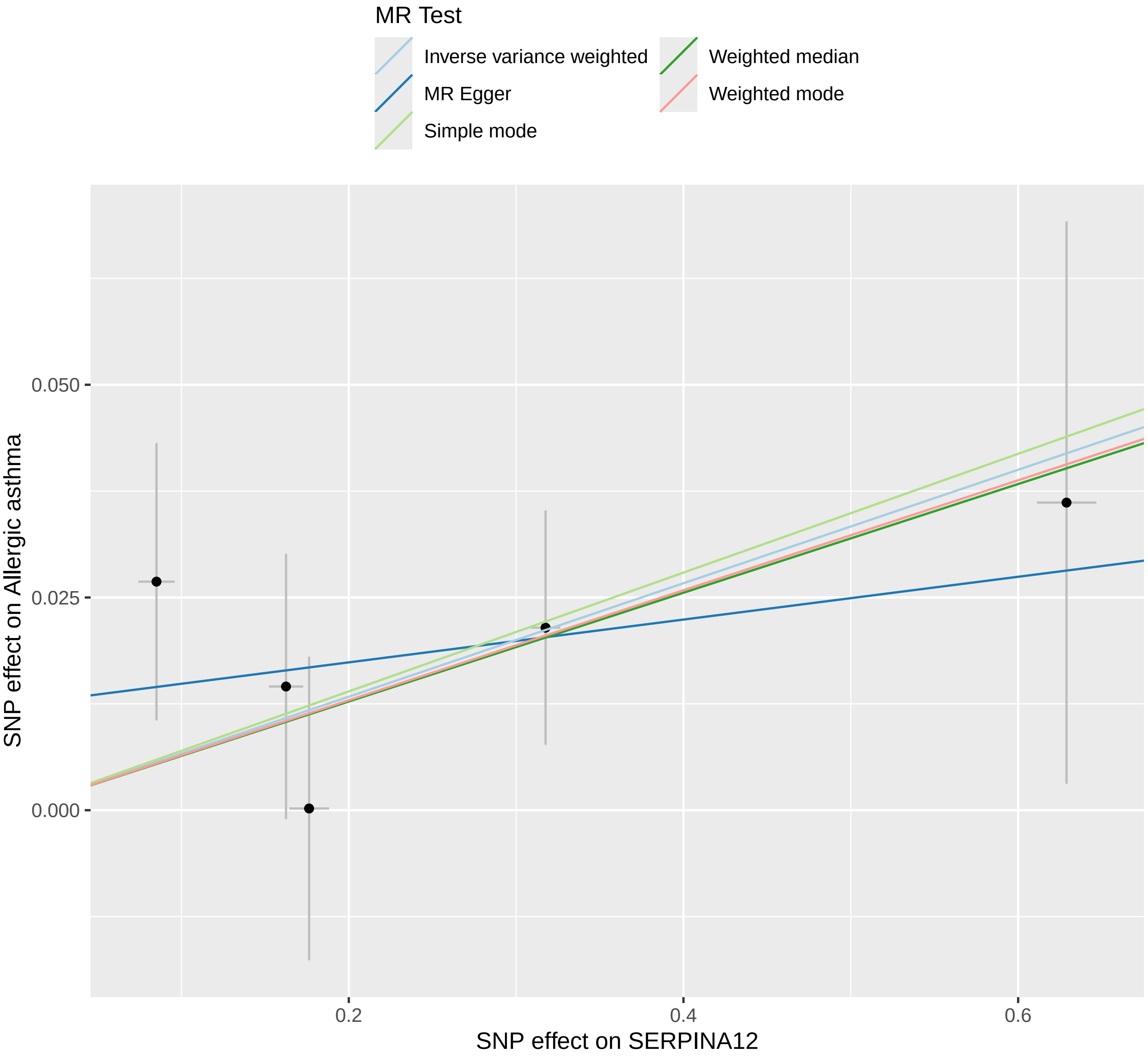
MR Test
Inverse variance weighted
MR Egger
Simple mode
Weighted median
Weighted mode
0.050
0.025
0.000
0.2
0.4
0.6
SNP effect on SERPINA12
SNP effect on Allergic asthma

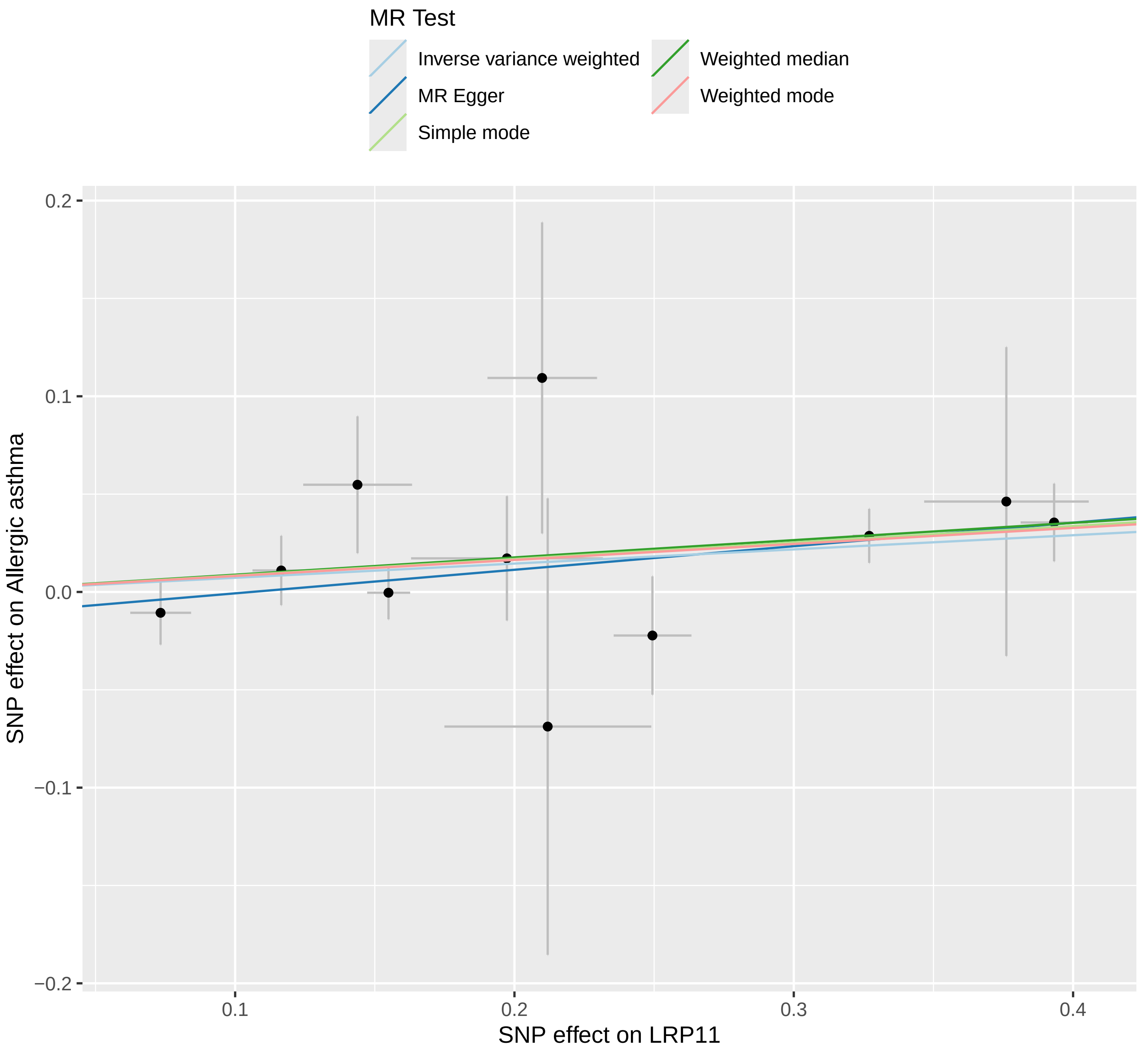
MR Test
Inverse variance weighted
MR Egger
Simple mode
Weighted median
Weighted mode
SNP effect on Allergic asthma
SNP effect on LRP11
0.2
0.1
0.0
−0.1
−0.2
0.1
0.2
0.3
0.4

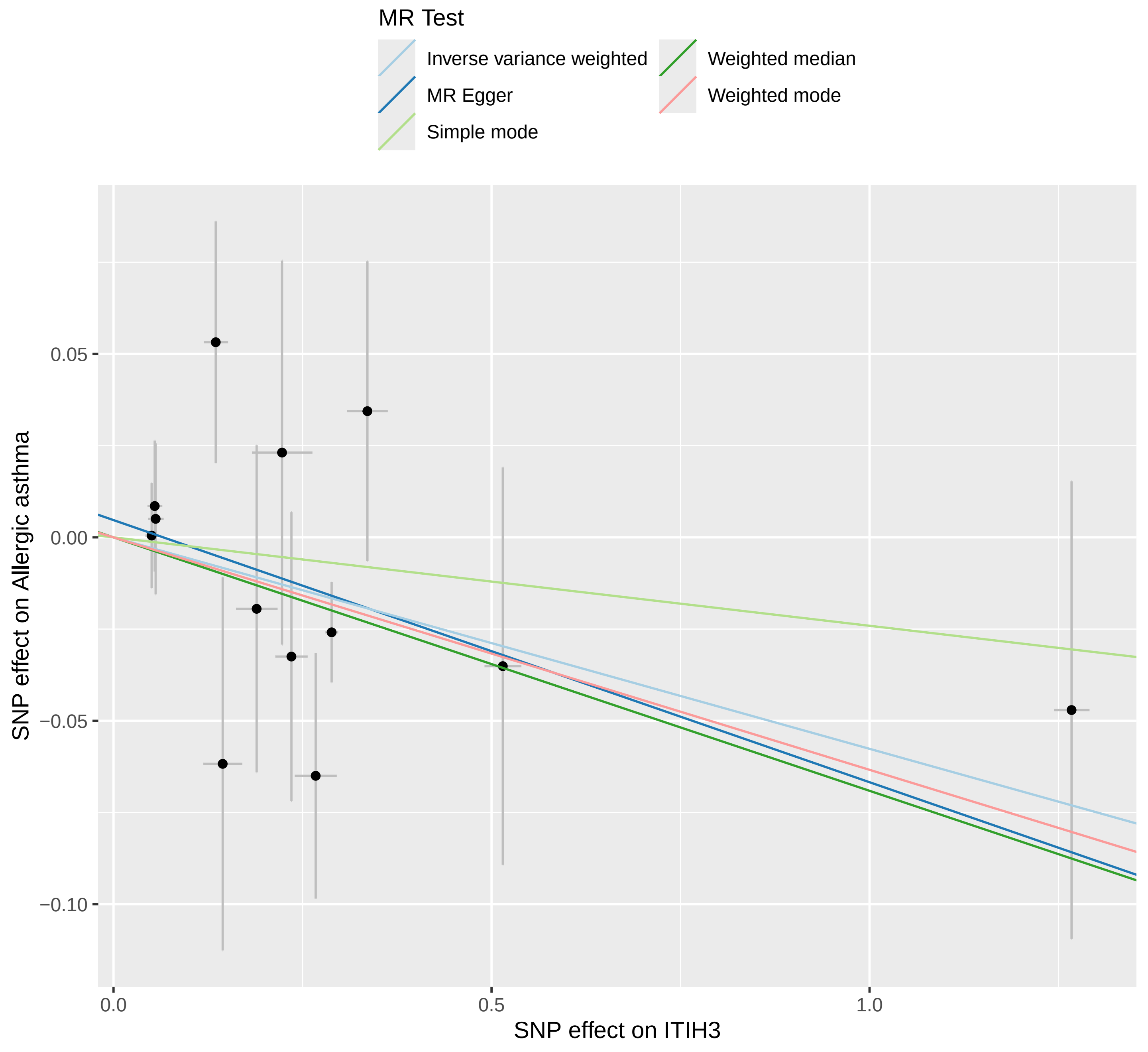
MR Test
Inverse variance weighted
MR Egger
Simple mode
Weighted median
Weighted mode
SNP effect on Allergic asthma
0.05
0.00
−0.05
−0.10
0.0
0.5
1.0
SNP effect on ITIH3

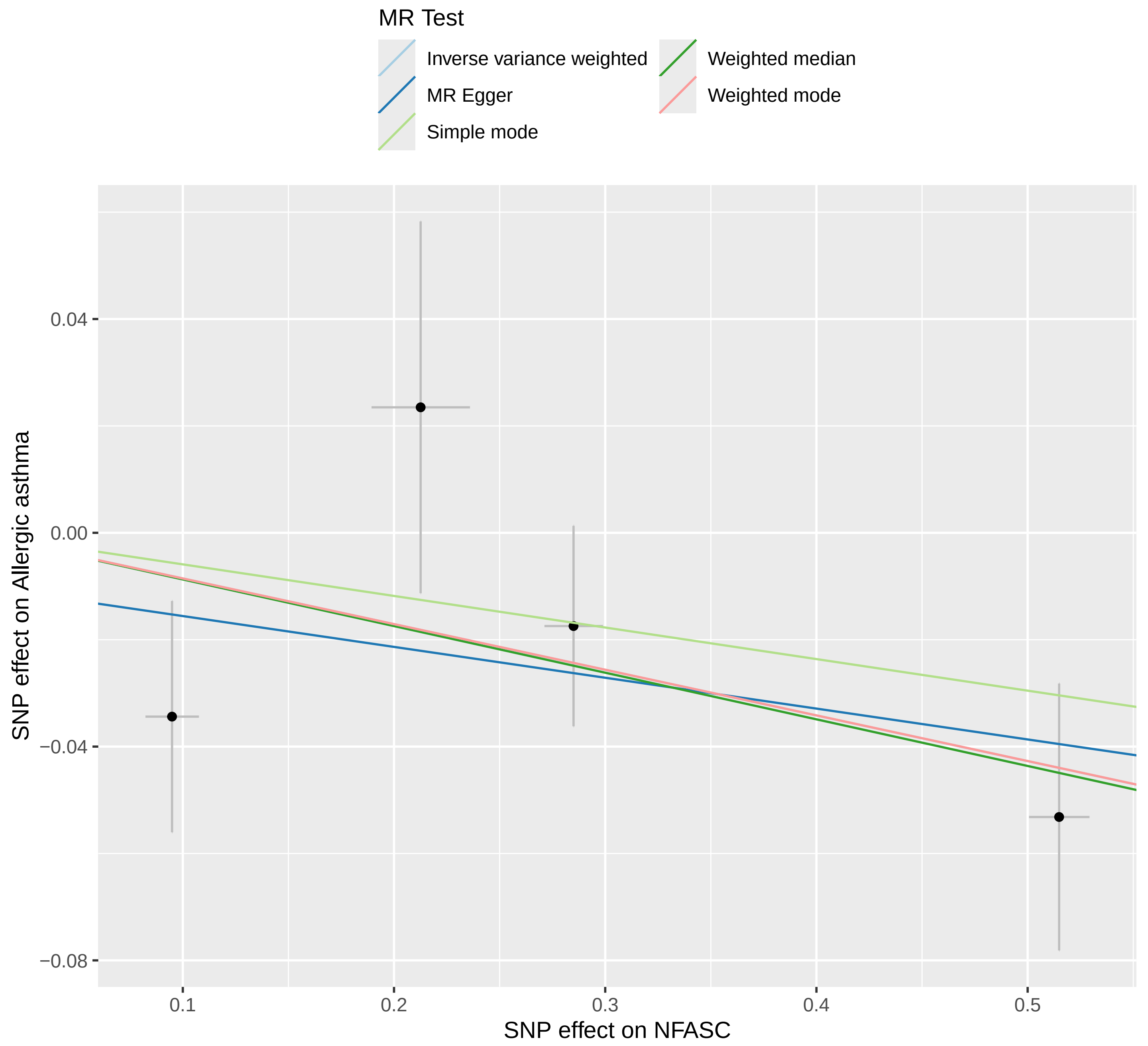
MR Test
Inverse variance weighted
MR Egger
Simple mode
Weighted median
Weighted mode
0.04
0.00
−0.04
−0.08
SNP effect on Allergic asthma
0.1
0.2
0.3
0.4
0.5
SNP effect on NFASC

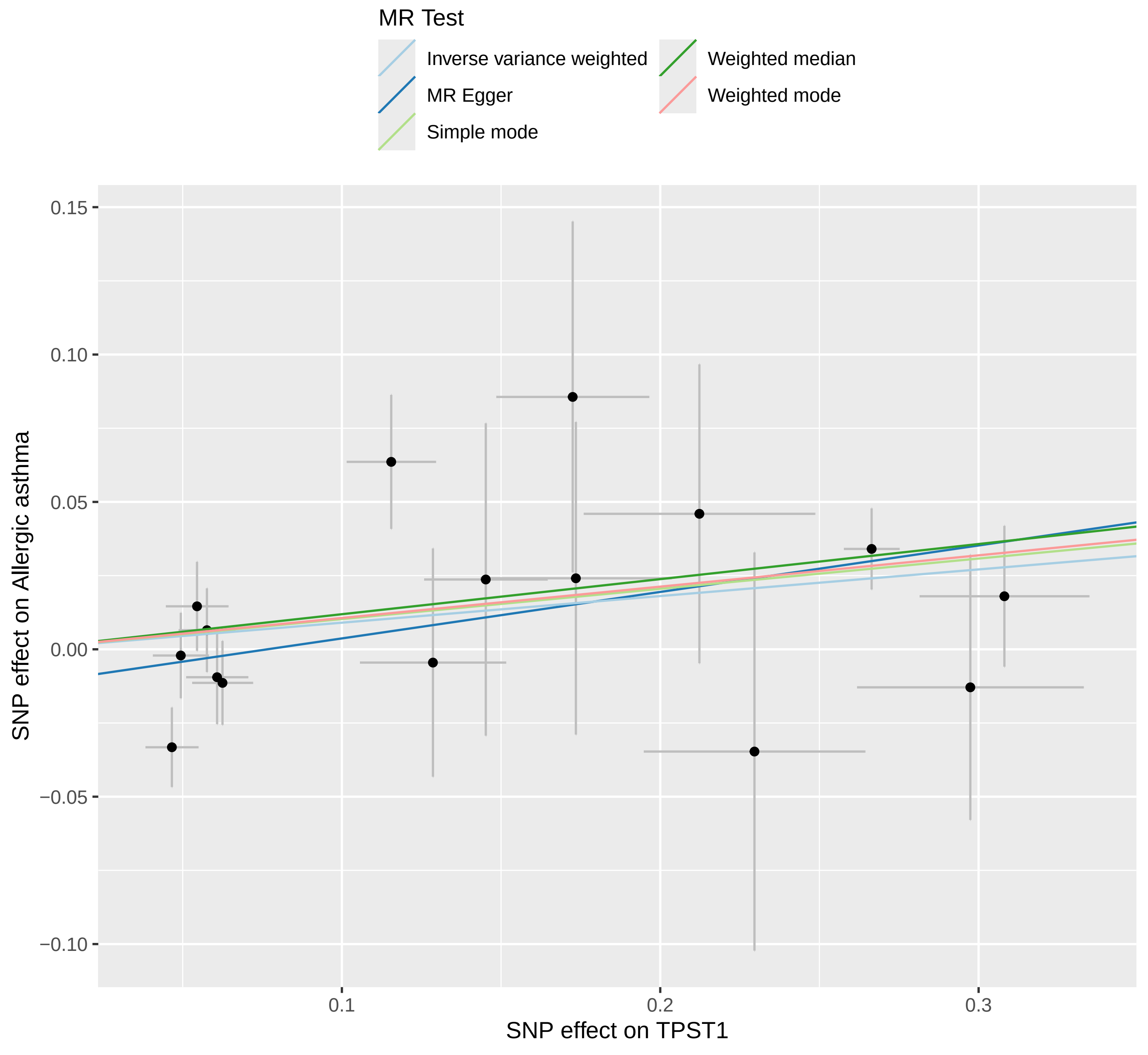
MR Test
Inverse variance weighted
MR Egger
Simple mode
Weighted median
Weighted mode
0.15
0.10
0.05
0.00
−0.05
−0.10
SNP effect on Allergic asthma
0.1
0.2
0.3
SNP effect on TPST1

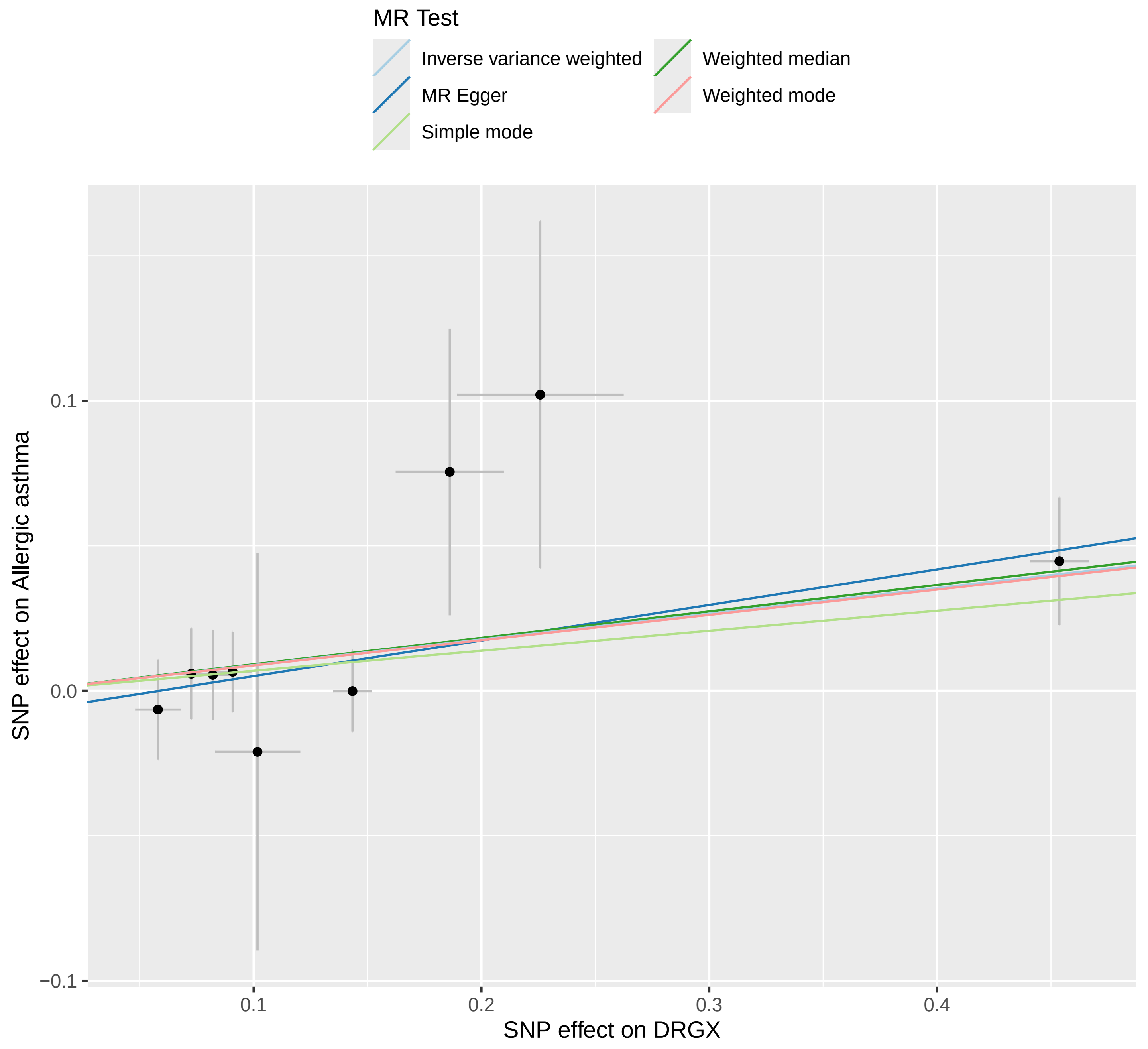

MR Test
Inverse variance weighted
MR Egger
Simple mode
Weighted median
Weighted mode
SNP effect on Allergic asthma
0.1
0.0
−0.1
0.1
0.2
0.3
0.4
SNP effect on DRGX

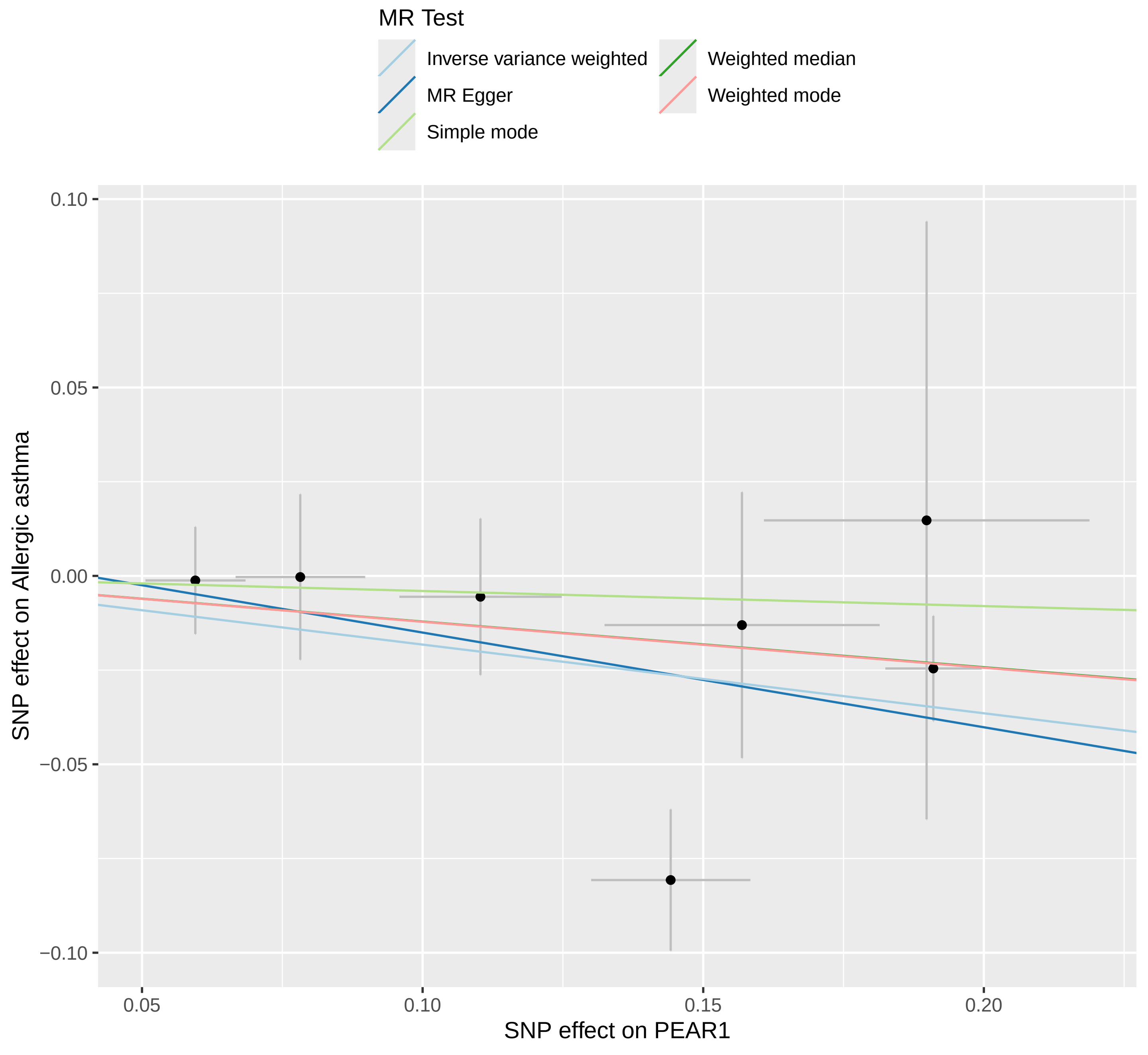
MR Test
Inverse variance weighted
MR Egger
Simple mode
Weighted median
Weighted mode
SNP effect on Allergic asthma
0.10
0.05
0.00
−0.05
−0.10
0.05
0.10
0.15
0.20
SNP effect on PEAR1

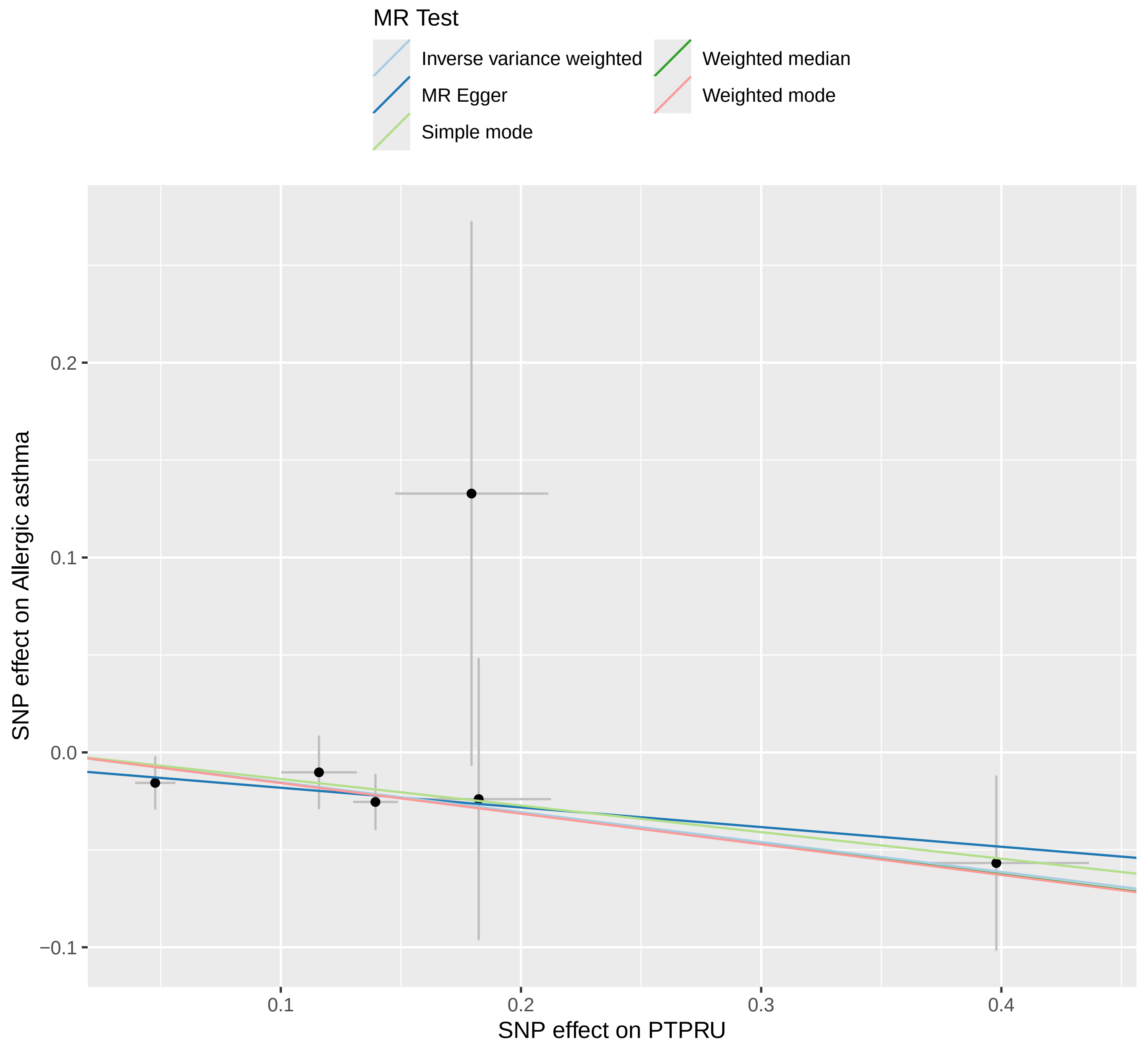
MR Test
Inverse variance weighted
MR Egger
Simple mode
Weighted median
Weighted mode
0.2
0.1
0.0
−0.1
0.1
0.2
0.3
0.4
SNP effect on Allergic asthma
SNP effect on PTPRU

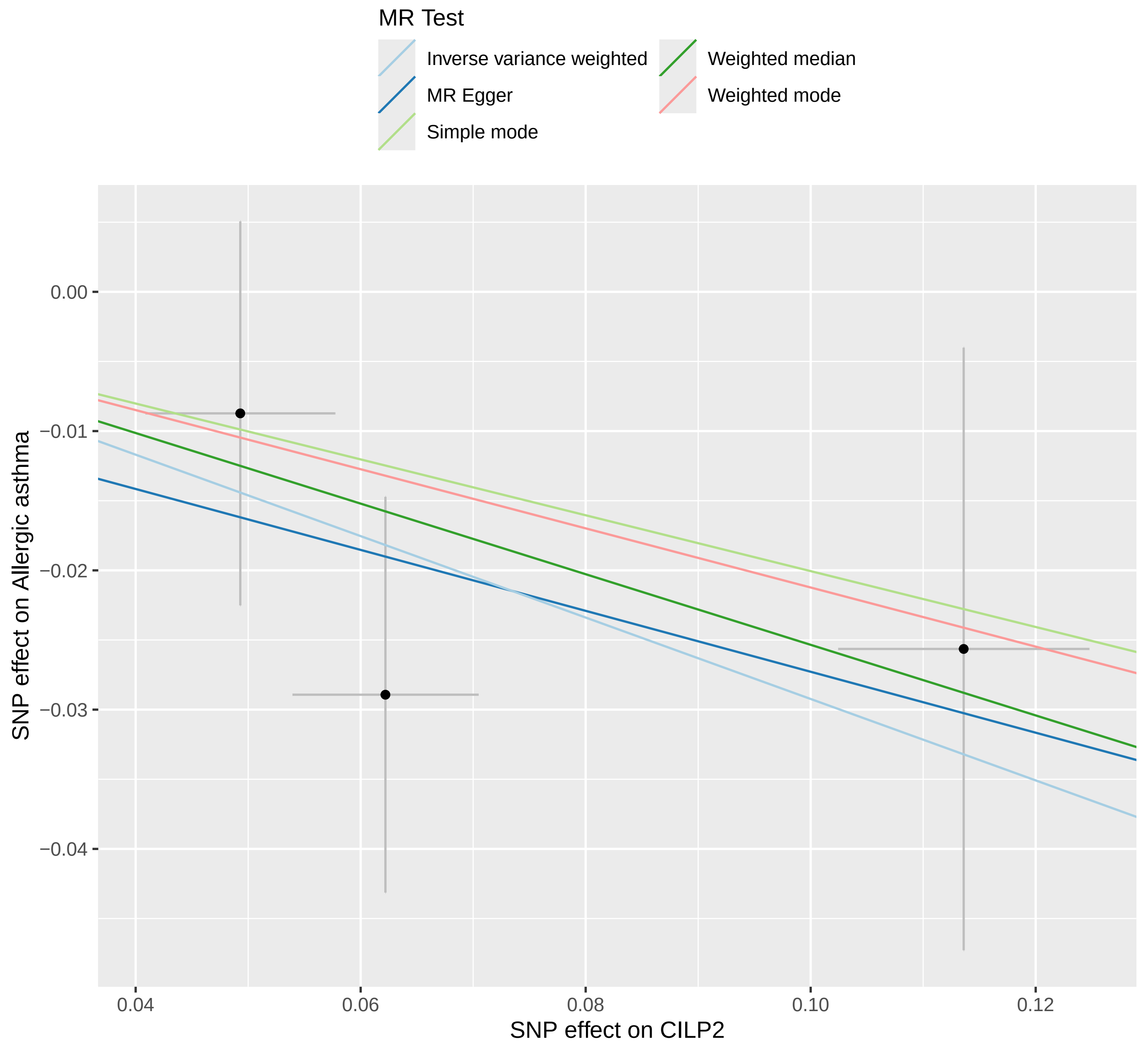

MR Test
Inverse variance weighted
MR Egger
Simple mode
Weighted median
Weighted mode
0.00
−0.01
−0.02
−0.03
−0.04
SNP effect on Allergic asthma
0.04
0.06
0.08
0.10
0.12
SNP effect on CILP2

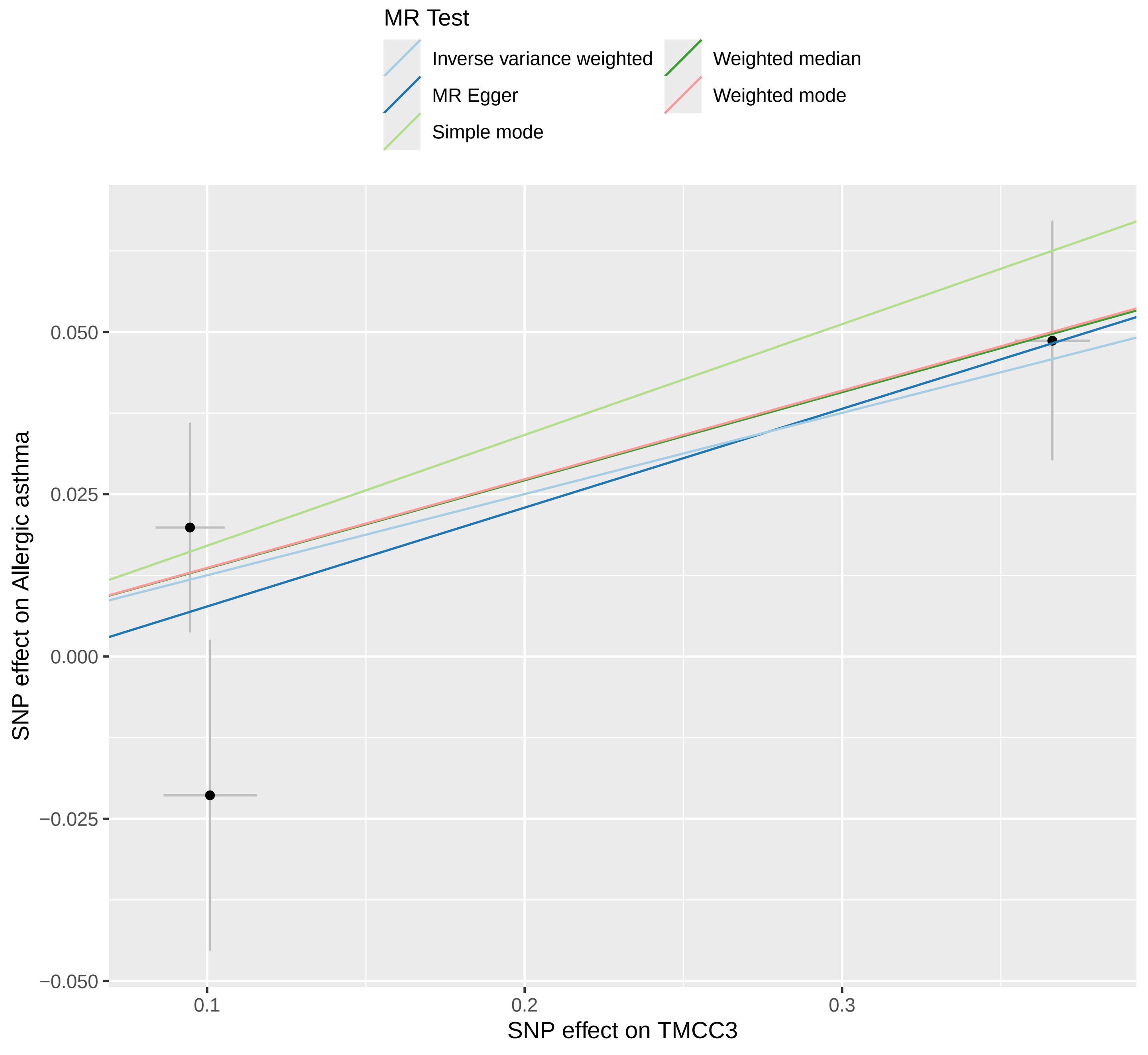

MR Test
Inverse variance weighted
MR Egger
Simple mode
Weighted median
Weighted mode
SNP effect on Allergic asthma
0.050
0.025
0.000
−0.025
−0.050
0.1
0.2
0.3
SNP effect on TMCC3

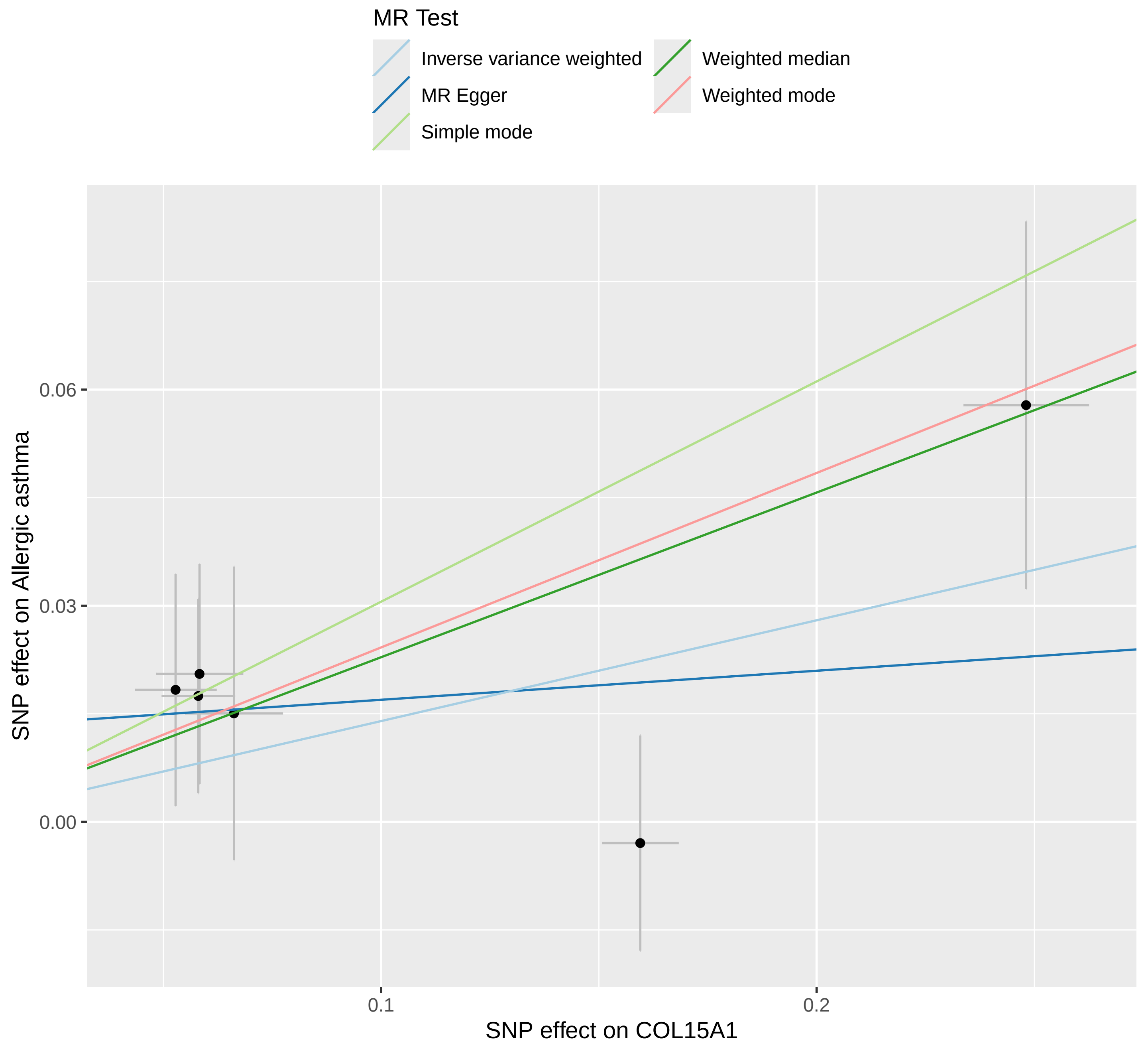
MR Test
Inverse variance weighted
MR Egger
Simple mode
Weighted median
Weighted mode
0.06
0.03
0.00
SNP effect on Allergic asthma
0.1
0.2
SNP effect on COL15A1

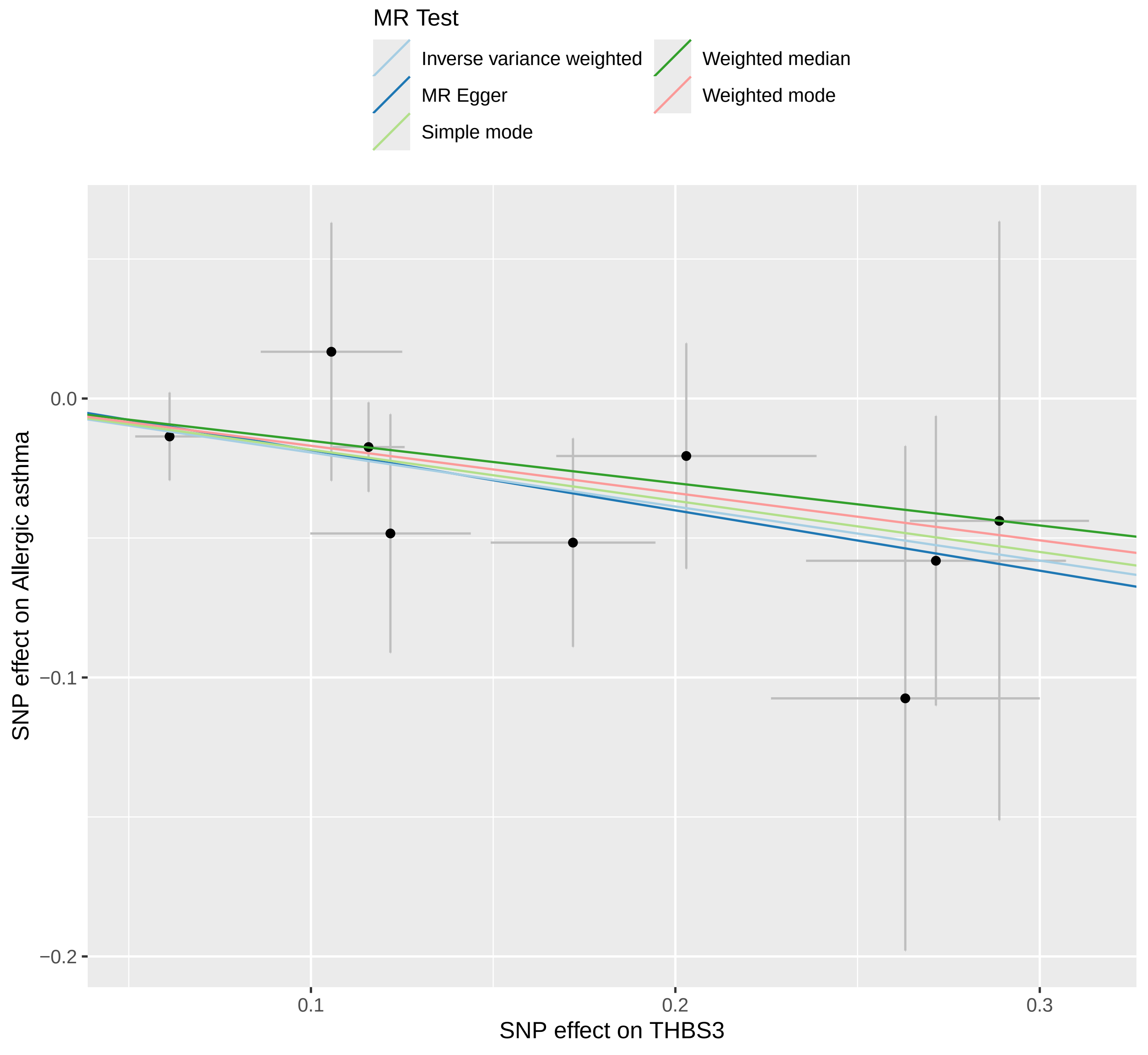
MR Test
Inverse variance weighted
MR Egger
Simple mode
Weighted median
Weighted mode
0.0
−0.1
−0.2
0.1
0.2
0.3
SNP effect on Allergic asthma
SNP effect on THBS3

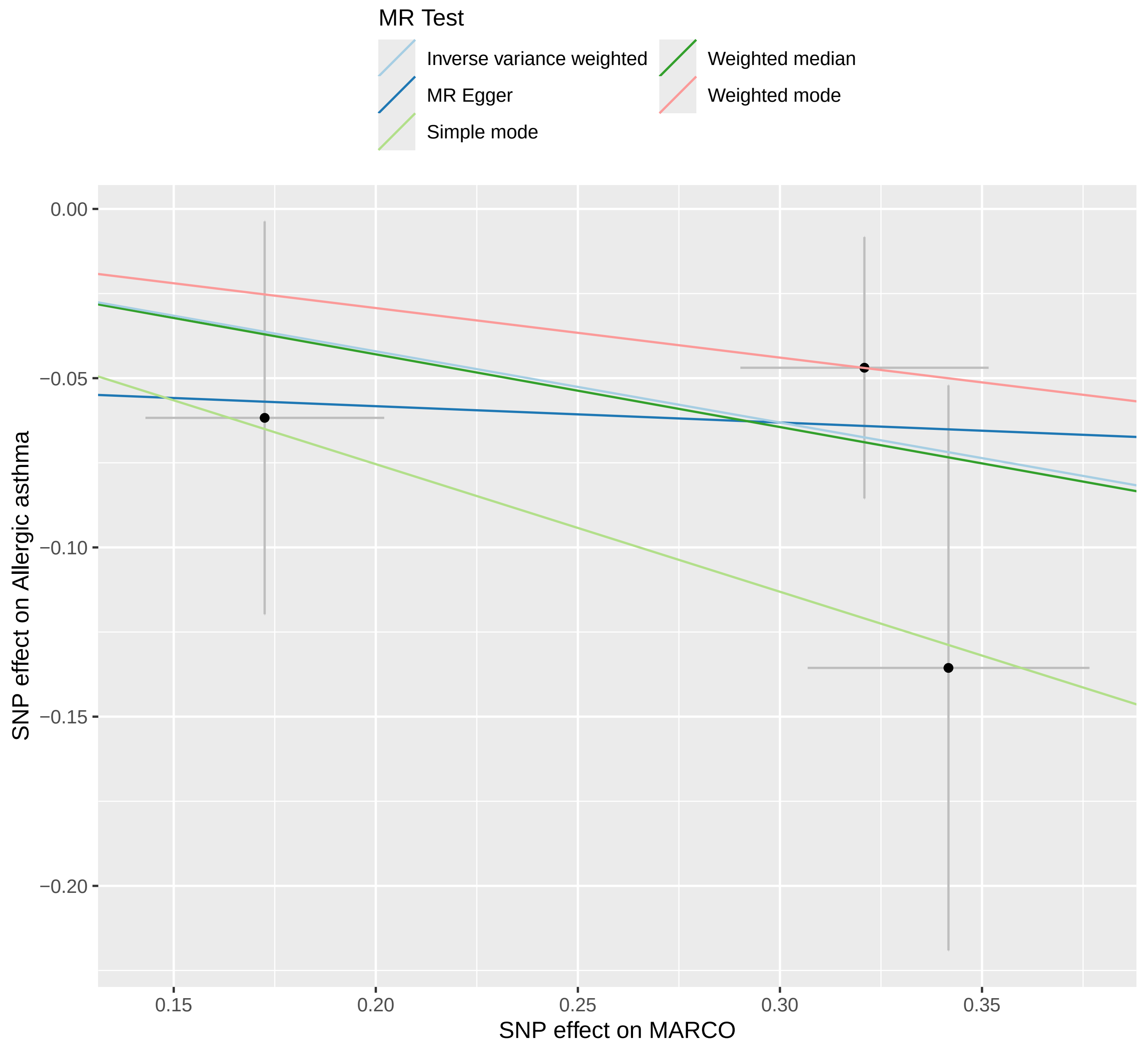
MR Test
Inverse variance weighted
MR Egger
Simple mode
Weighted median
Weighted mode
0.00
−0.05
−0.10
−0.15
−0.20
SNP effect on Allergic asthma
0.15
0.20
0.25
0.30
0.35
SNP effect on MARCO

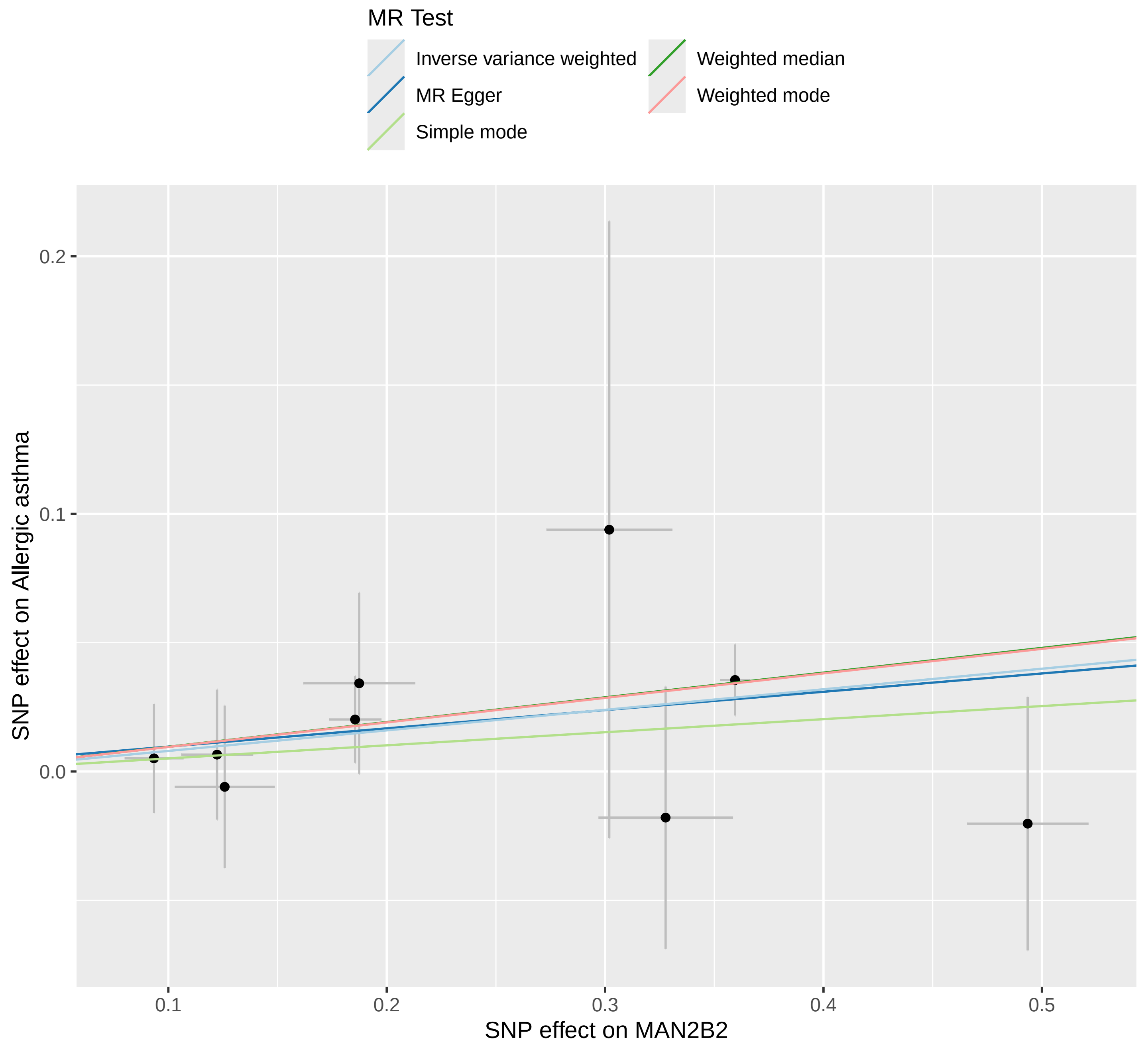
MR Test
Inverse variance weighted
MR Egger
Simple mode
Weighted median
Weighted mode
0.2
0.1
0.0
0.1
0.2
0.3
0.4
0.5
SNP effect on Allergic asthma
SNP effect on MAN2B2

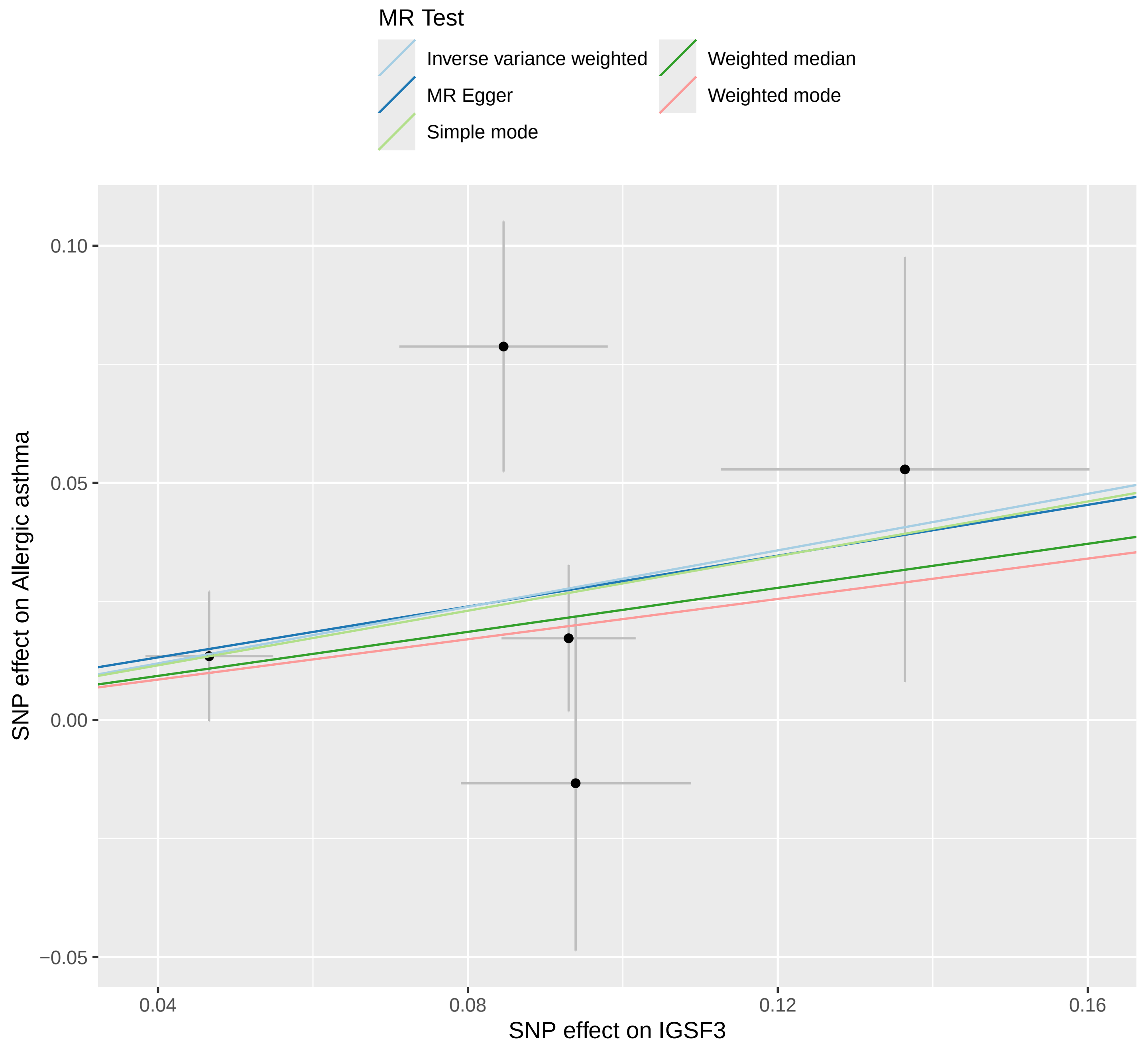

MR Test
Inverse variance weighted
MR Egger
Simple mode
Weighted median
Weighted mode
0.10
0.05
0.00
−0.05
0.04
0.08
0.12
0.16
SNP effect on Allergic asthma
SNP effect on IGSF3

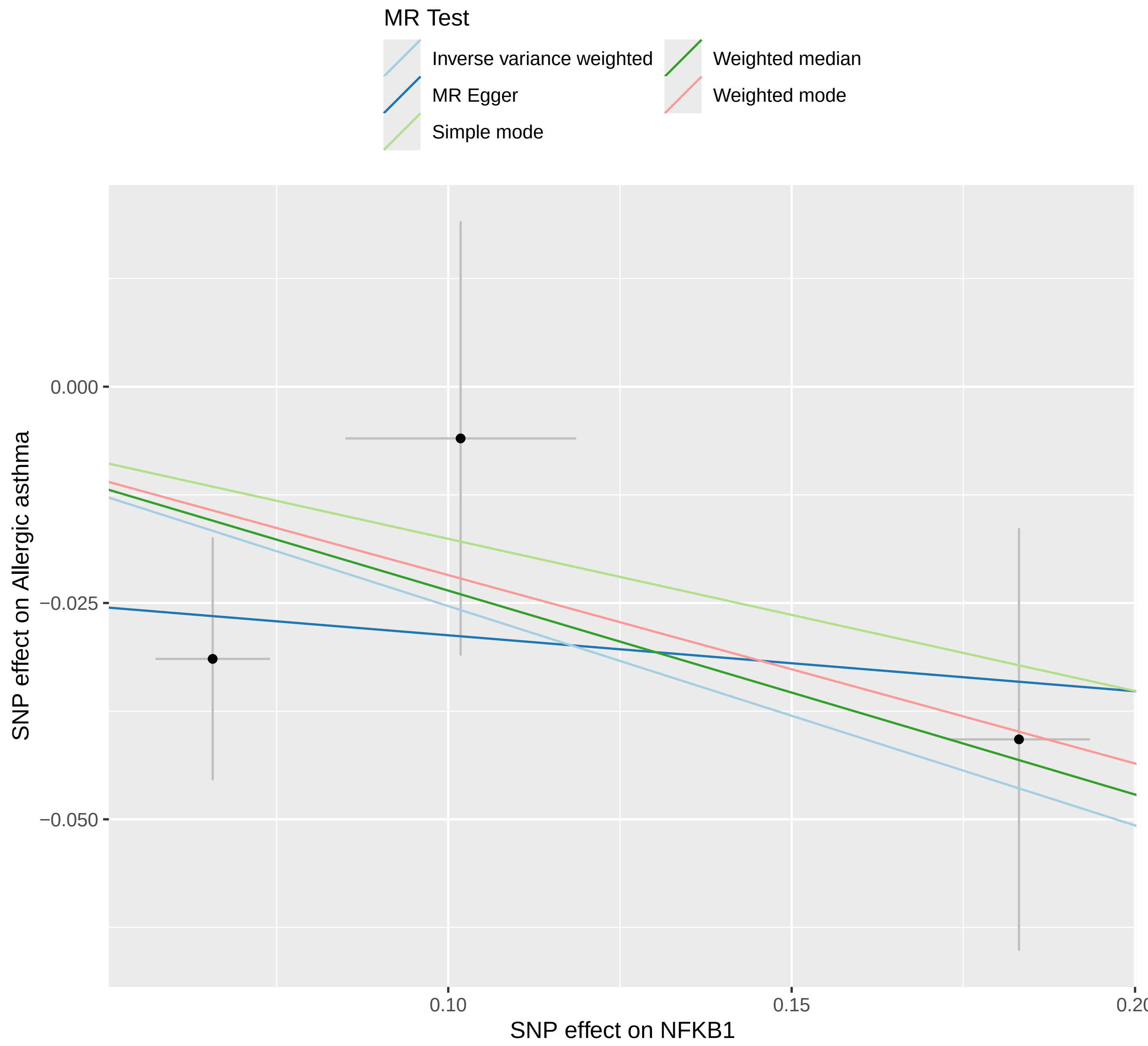

MR Test
Inverse variance weighted
MR Egger
Simple mode
Weighted median
Weighted mode
0.000
−0.025
−0.050
SNP effect on Allergic asthma
0.10
0.15
0.20
SNP effect on NFKB1

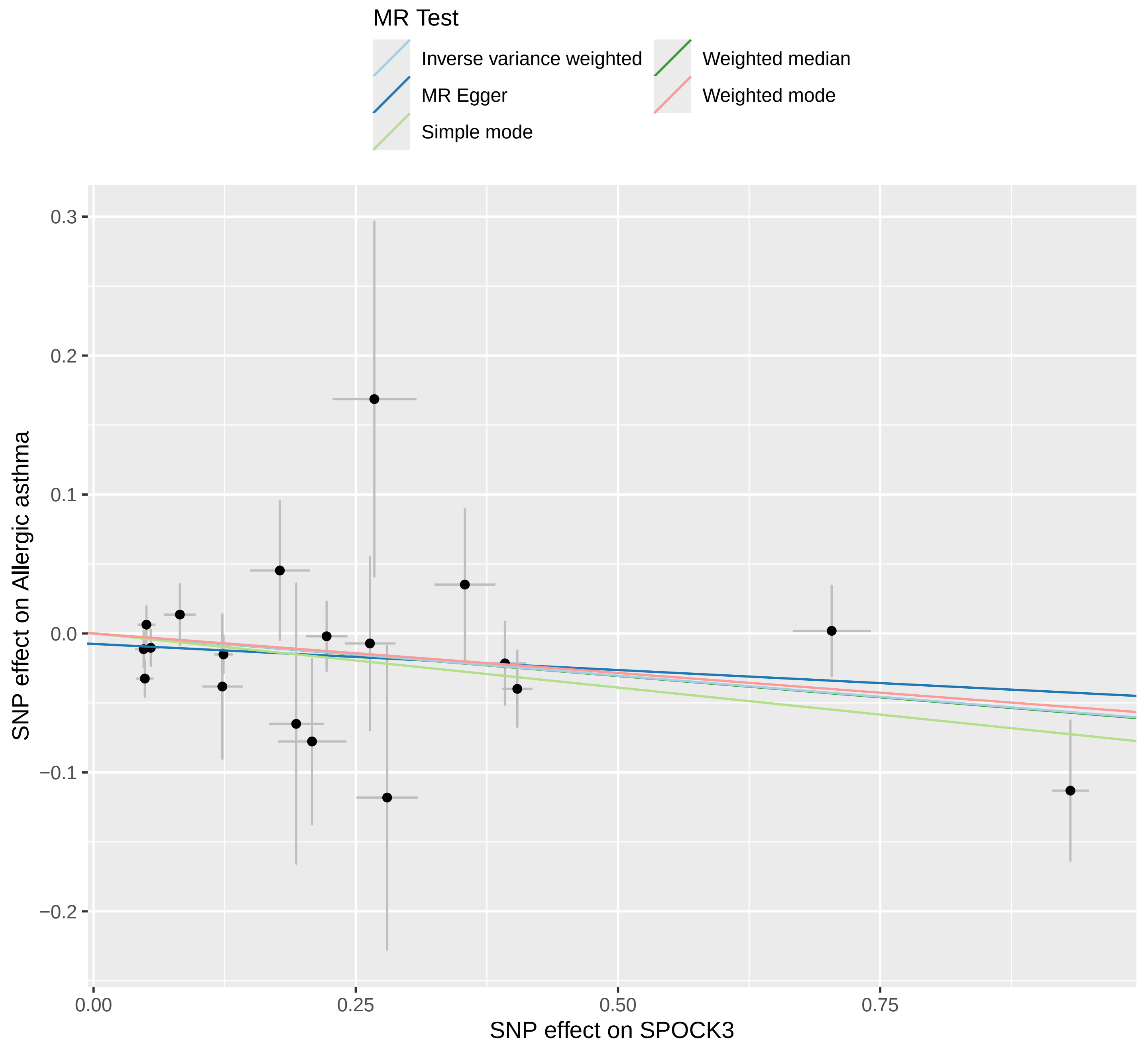
MR Test
Inverse variance weighted
MR Egger
Simple mode
Weighted median
Weighted mode
0.3
0.2
0.1
0.0
−0.1
−0.2
SNP effect on Allergic asthma
0.00
0.25
0.50
0.75
SNP effect on SPOCK3

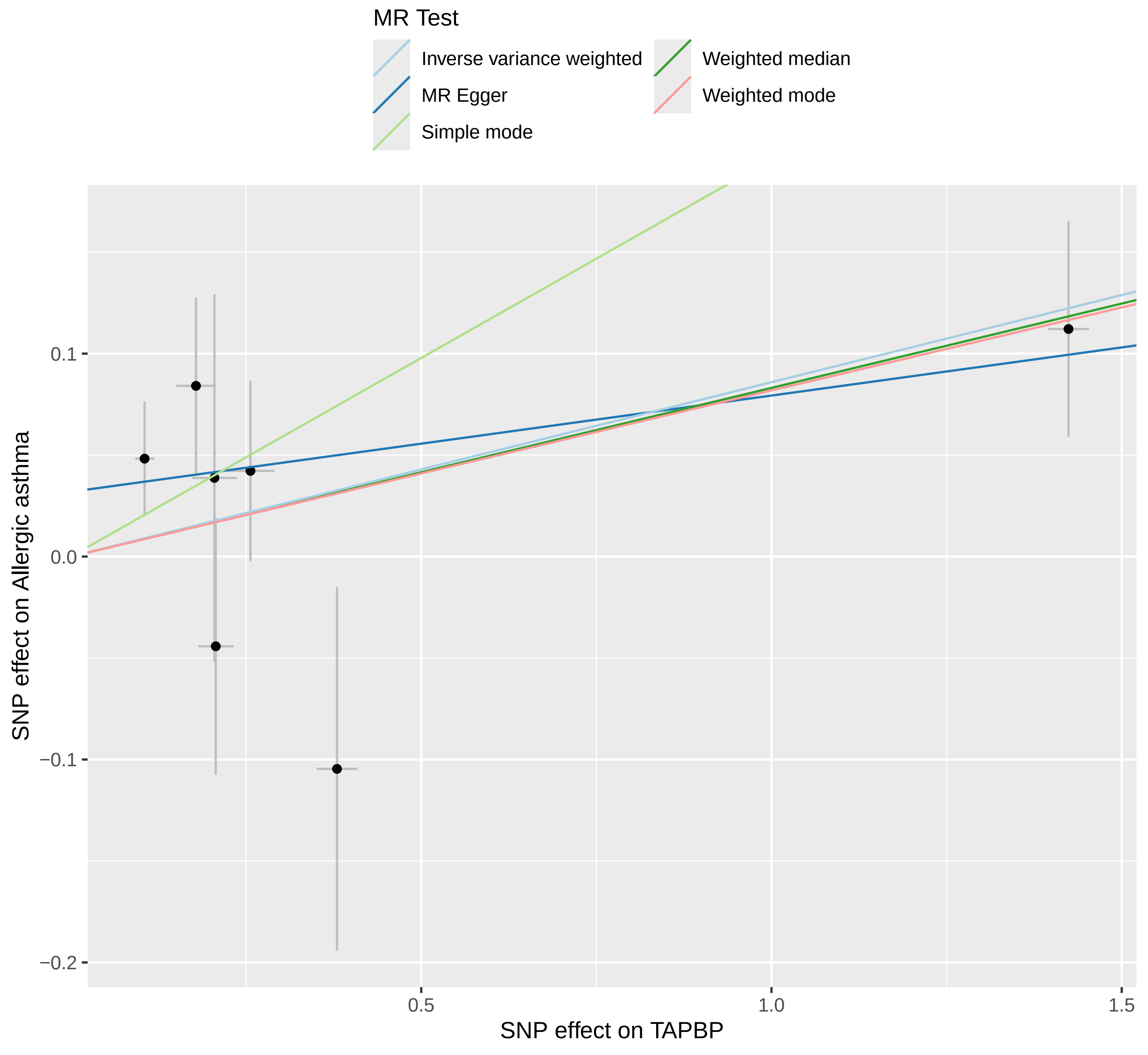
MR Test
Inverse variance weighted
MR Egger
Simple mode
Weighted median
Weighted mode
0.1
0.0
−0.1
−0.2
0.5
1.0
1.5
SNP effect on Allergic asthma
SNP effect on TAPBP

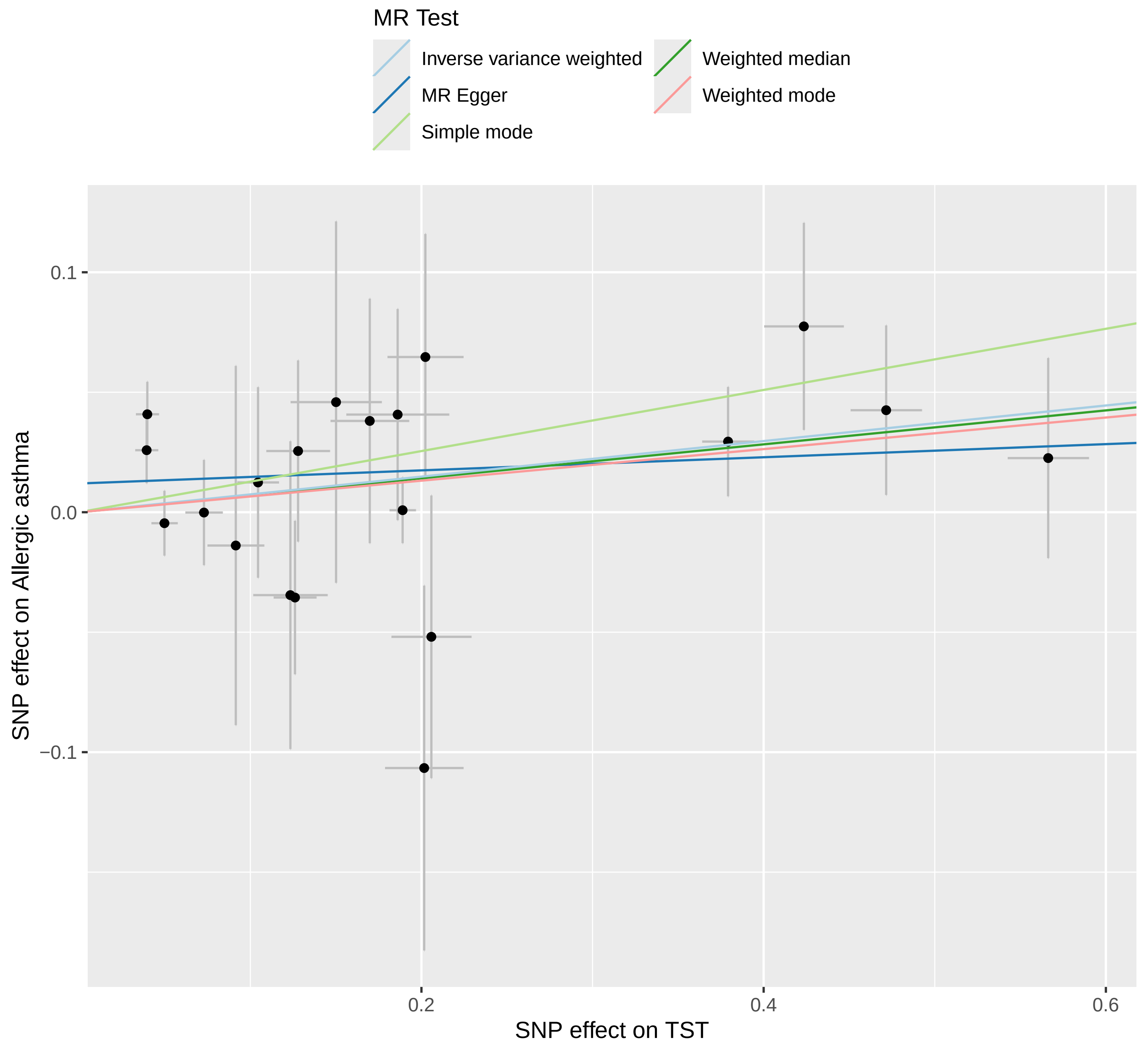
MR Test
Inverse variance weighted
MR Egger
Simple mode
Weighted median
Weighted mode
0.1
0.0
−0.1
0.2
0.4
0.6
SNP effect on Allergic asthma
SNP effect on TST

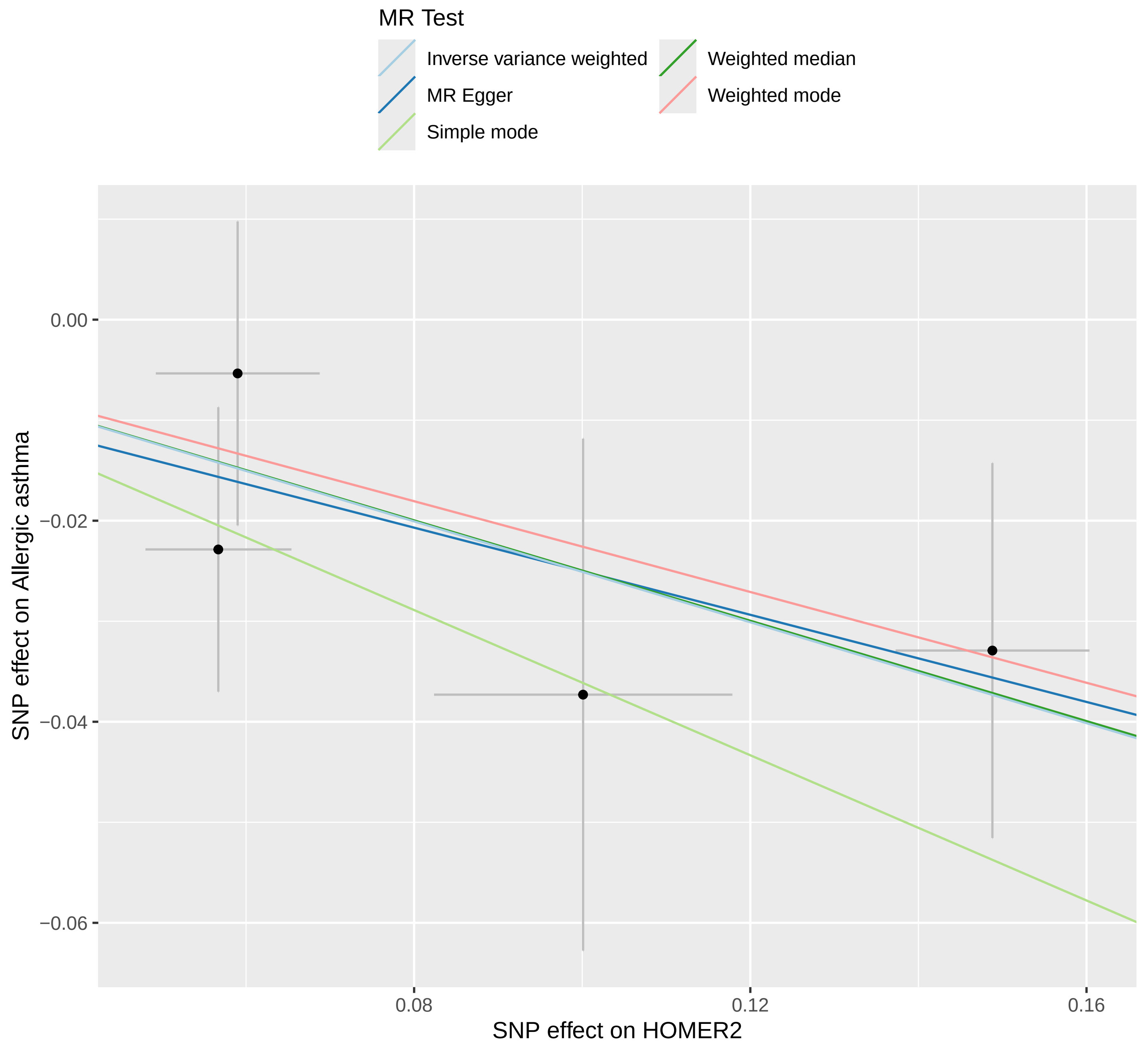

MR Test
Inverse variance weighted
MR Egger
Simple mode
Weighted median
Weighted mode
0.00
−0.02
−0.04
−0.06
0.08
0.12
0.16
SNP effect on Allergic asthma
SNP effect on HOMER2

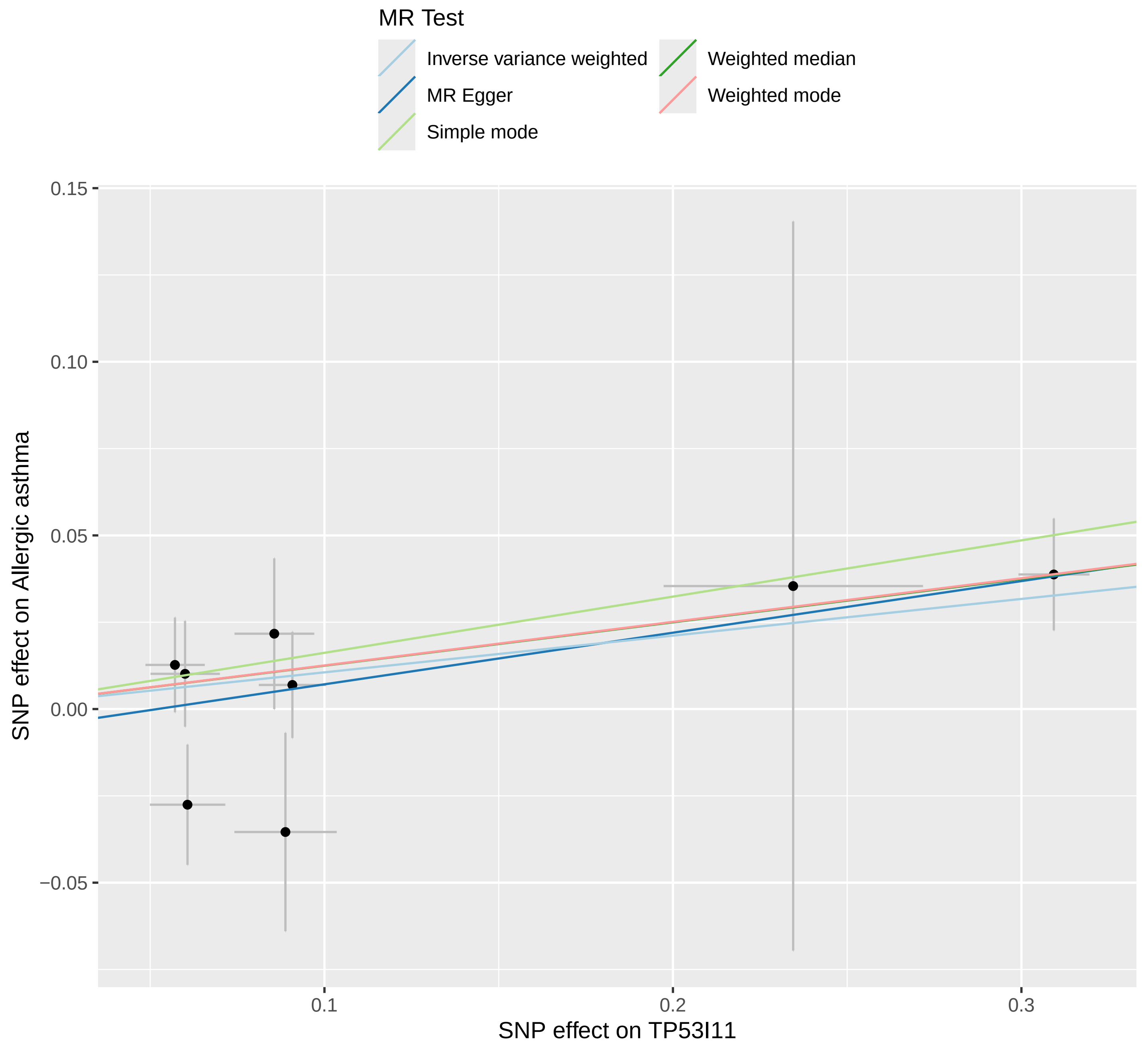
MR Test
Inverse variance weighted
MR Egger
Simple mode
Weighted median
Weighted mode
0.15
0.10
0.05
0.00
−0.05
0.1
0.2
0.3
SNP effect on Allergic asthma
SNP effect on TP53I11

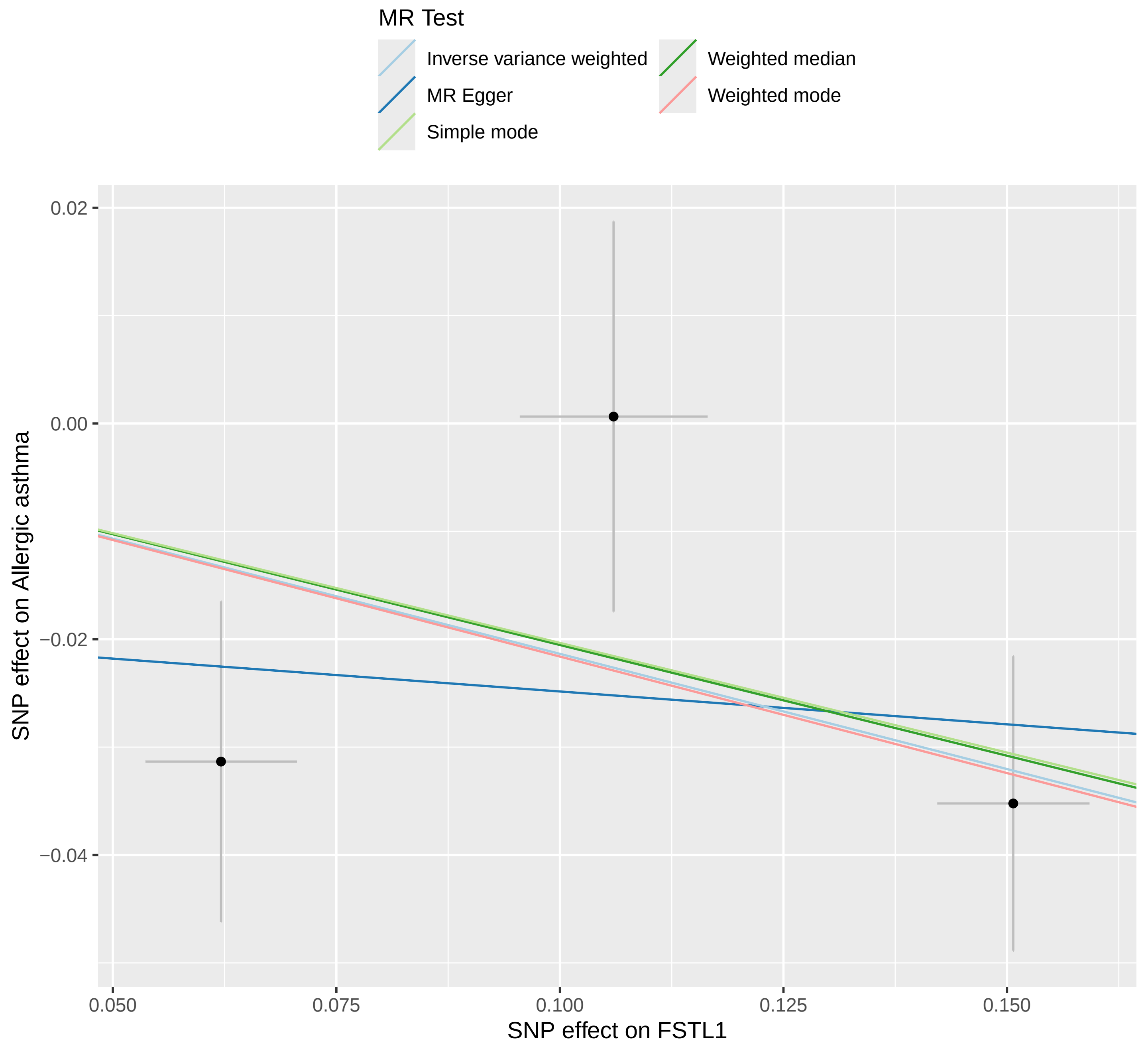
MR Test
Inverse variance weighted
MR Egger
Simple mode
Weighted median
Weighted mode
0.02
0.00
−0.02
−0.04
0.050
0.075
0.100
0.125
0.150
SNP effect on FSTL1
SNP effect on Allergic asthma

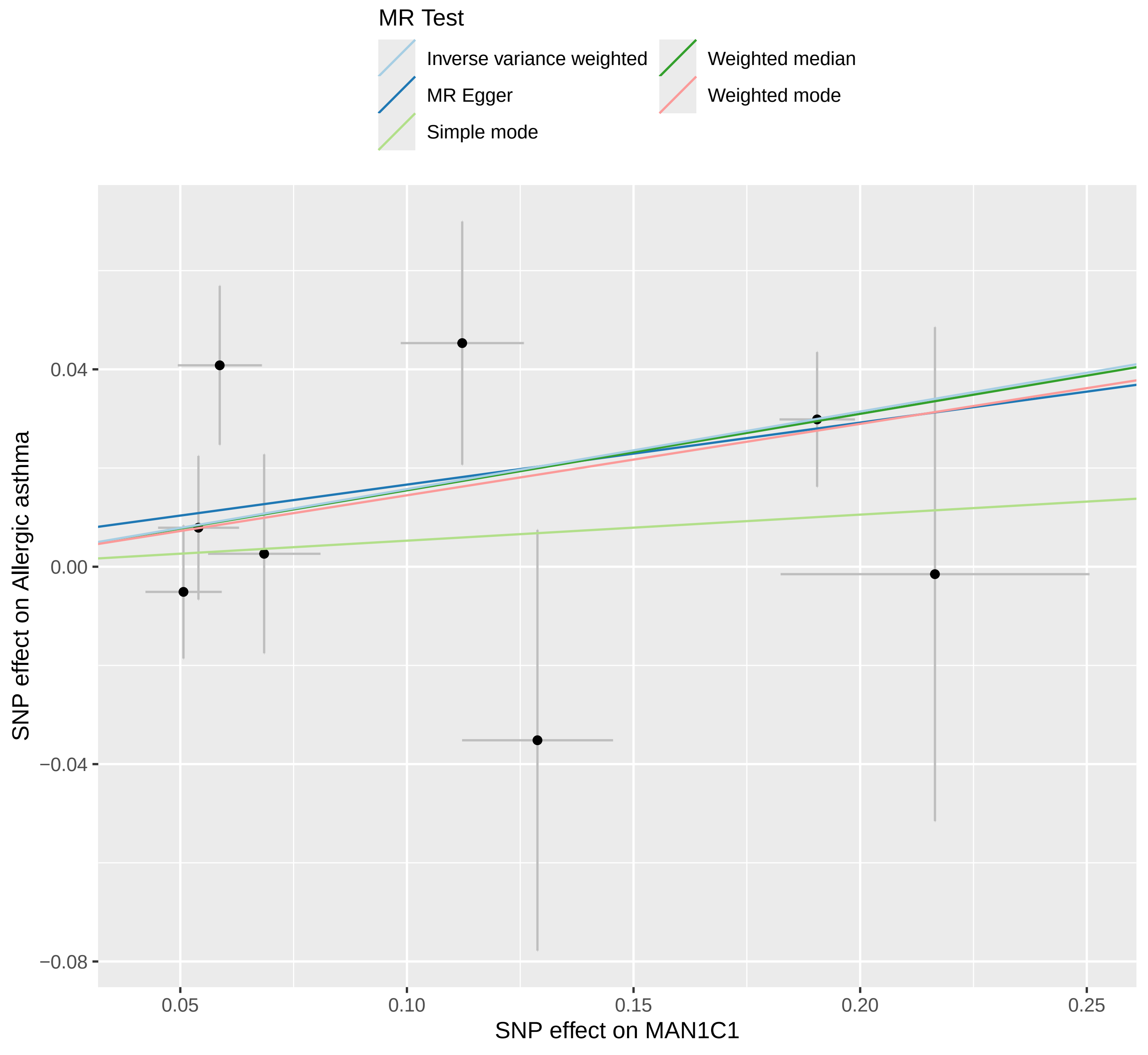

MR Test
Inverse variance weighted
MR Egger
Simple mode
Weighted median
Weighted mode
0.04
0.00
−0.04
−0.08
0.05
0.10
0.15
0.20
0.25
SNP effect on MAN1C1
SNP effect on Allergic asthma

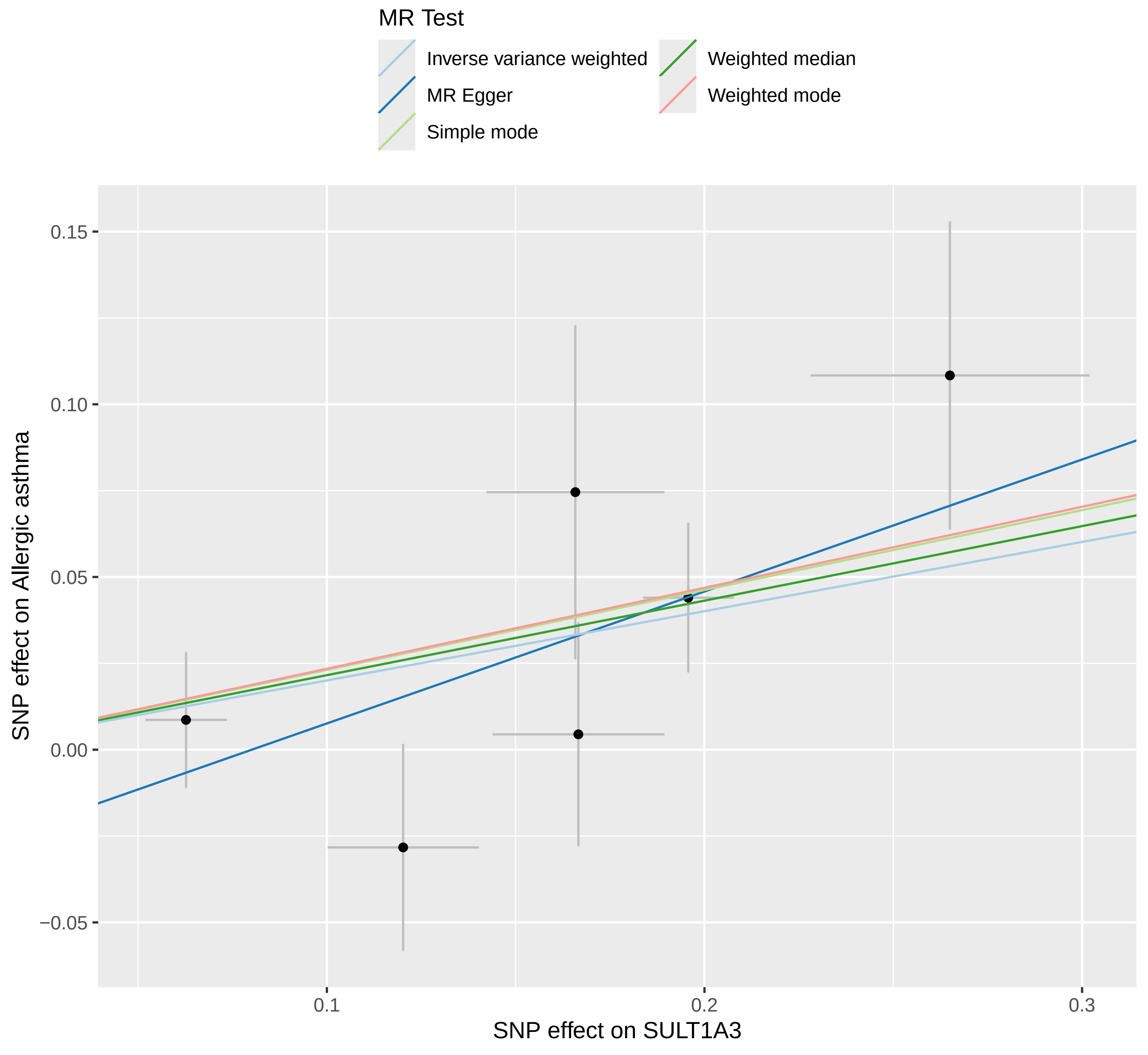
MR Test
Inverse variance weighted
MR Egger
Simple mode
Weighted median
Weighted mode
0.15
0.10
0.05
0.00
−0.05
SNP effect on Allergic asthma
0.1
0.2
0.3
SNP effect on SULT1A3

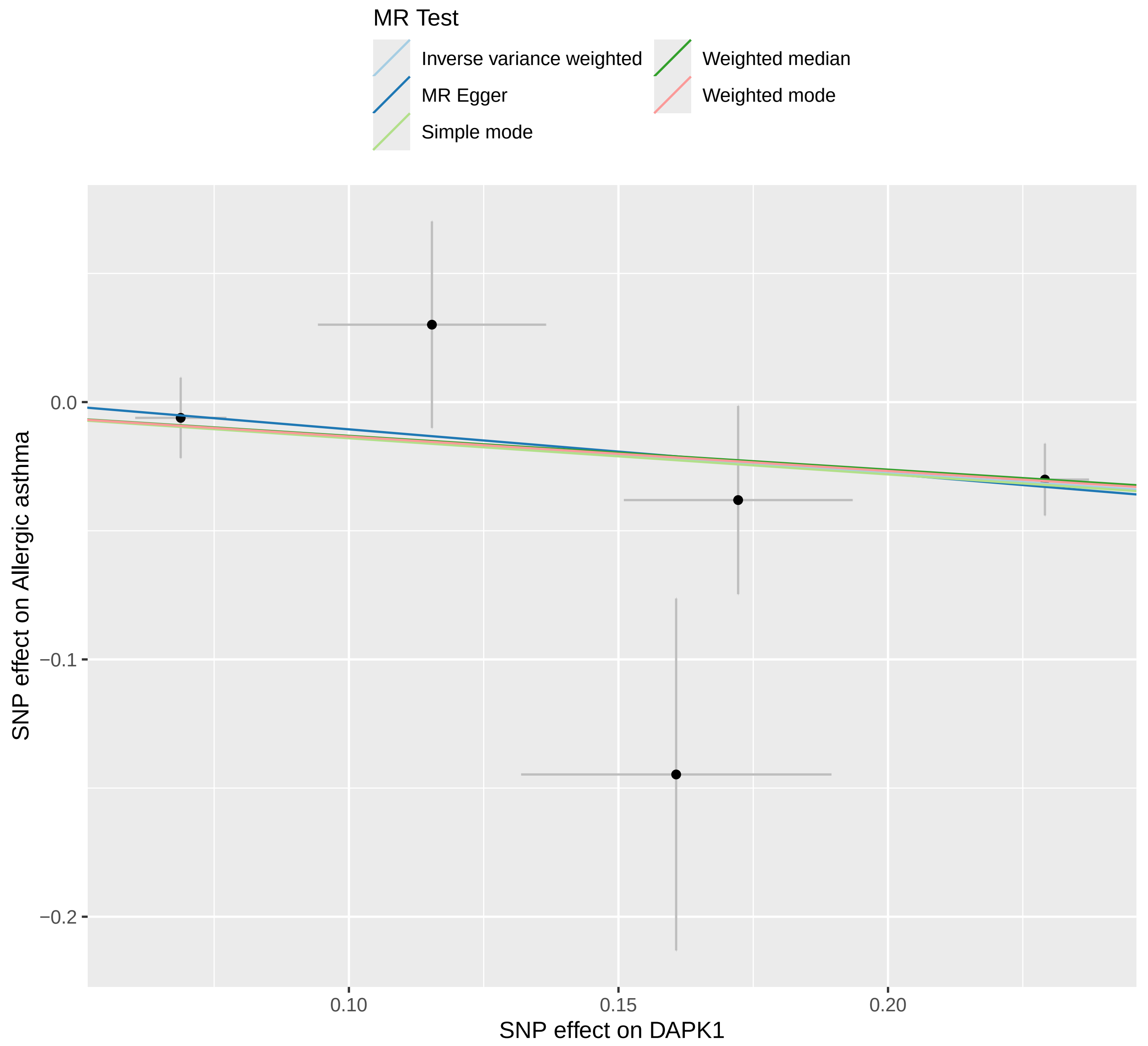
MR Test
Inverse variance weighted
MR Egger
Simple mode
Weighted median
Weighted mode
0.0
−0.1
−0.2
SNP effect on Allergic asthma
0.10
0.15
0.20
SNP effect on DAPK1

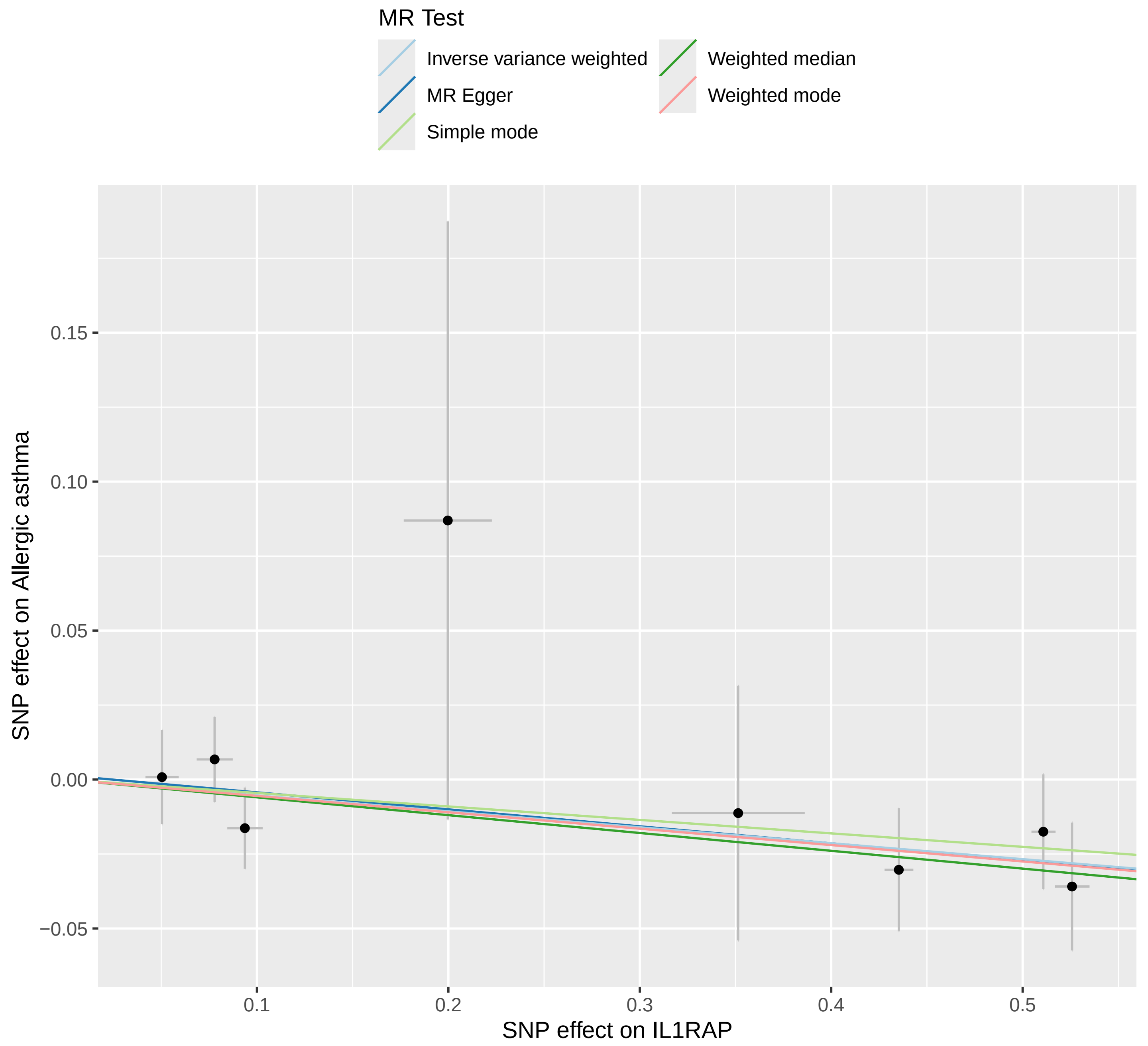

MR Test
Inverse variance weighted
MR Egger
Simple mode
Weighted median
Weighted mode
0.15
0.10
0.05
0.00
−0.05
0.1
0.2
0.3
0.4
0.5
SNP effect on IL1RAP
SNP effect on Allergic asthma

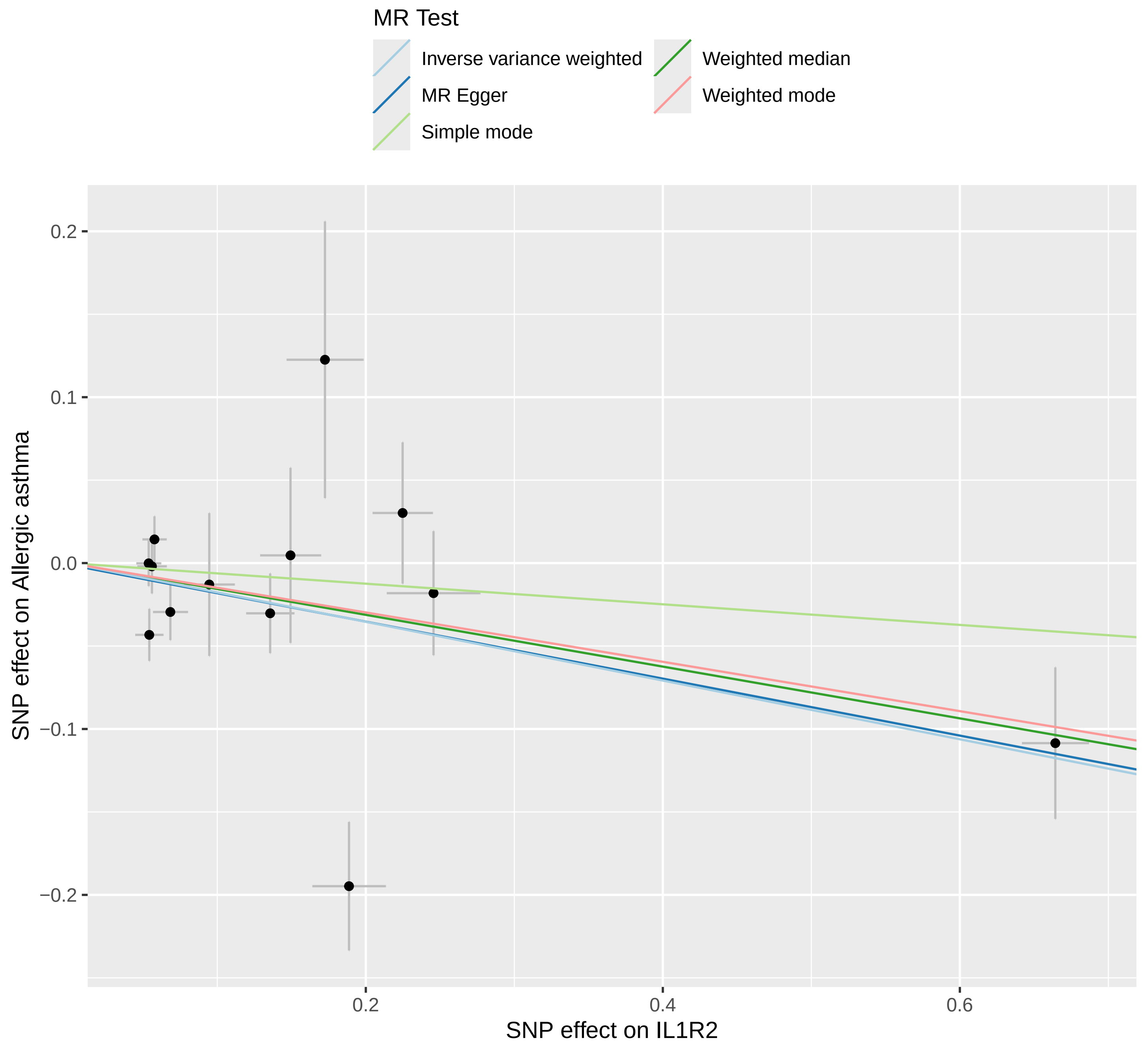
MR Test
Inverse variance weighted
MR Egger
Simple mode
Weighted median
Weighted mode
0.2
0.1
0.0
−0.1
−0.2
0.2
0.4
0.6
SNP effect on Allergic asthma
SNP effect on IL1R2

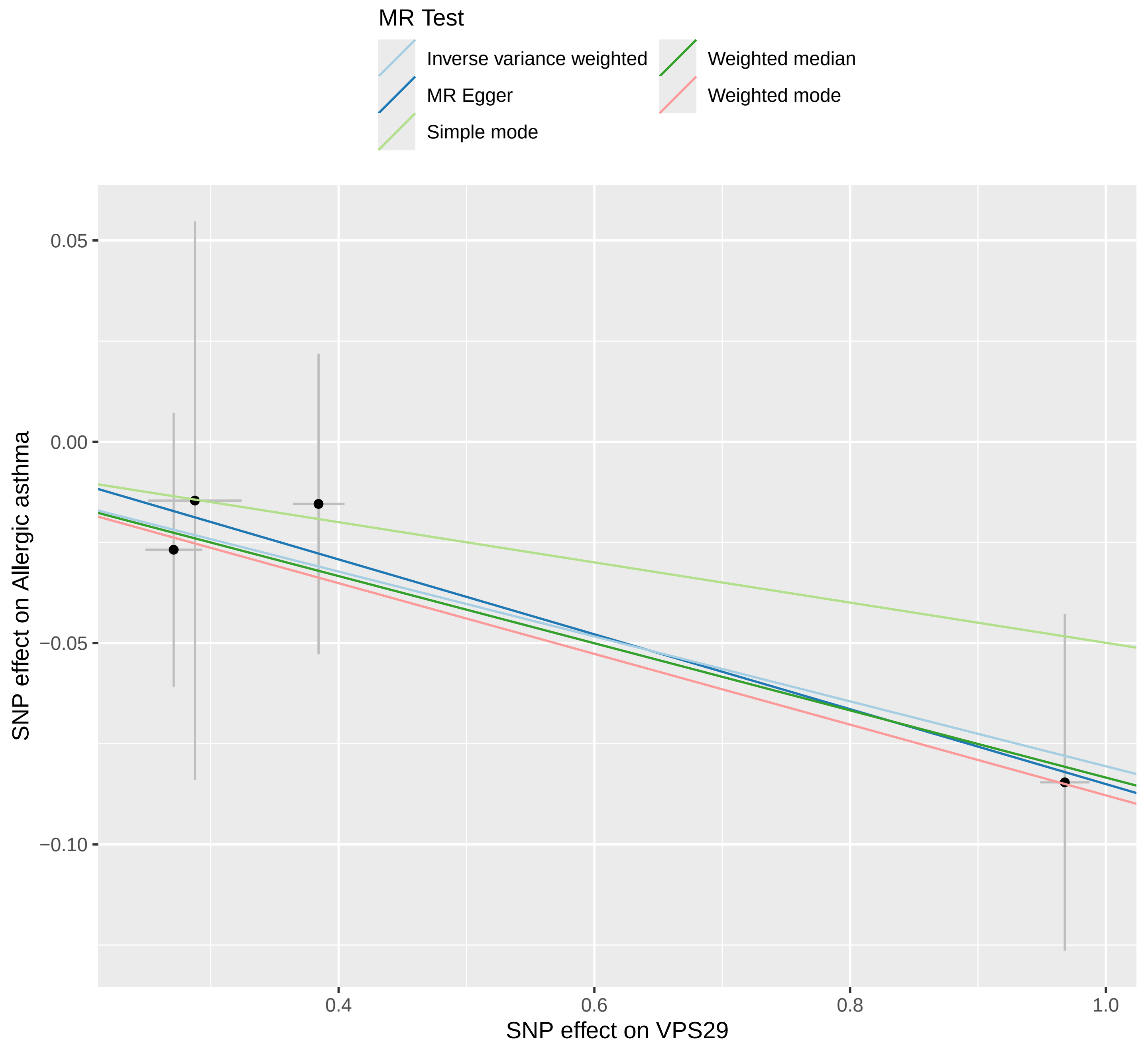

MR Test
Inverse variance weighted
MR Egger
Simple mode
Weighted median
Weighted mode
0.05
0.00
−0.05
−0.10
SNP effect on Allergic asthma
0.4
0.6
0.8
1.0
SNP effect on VPS29

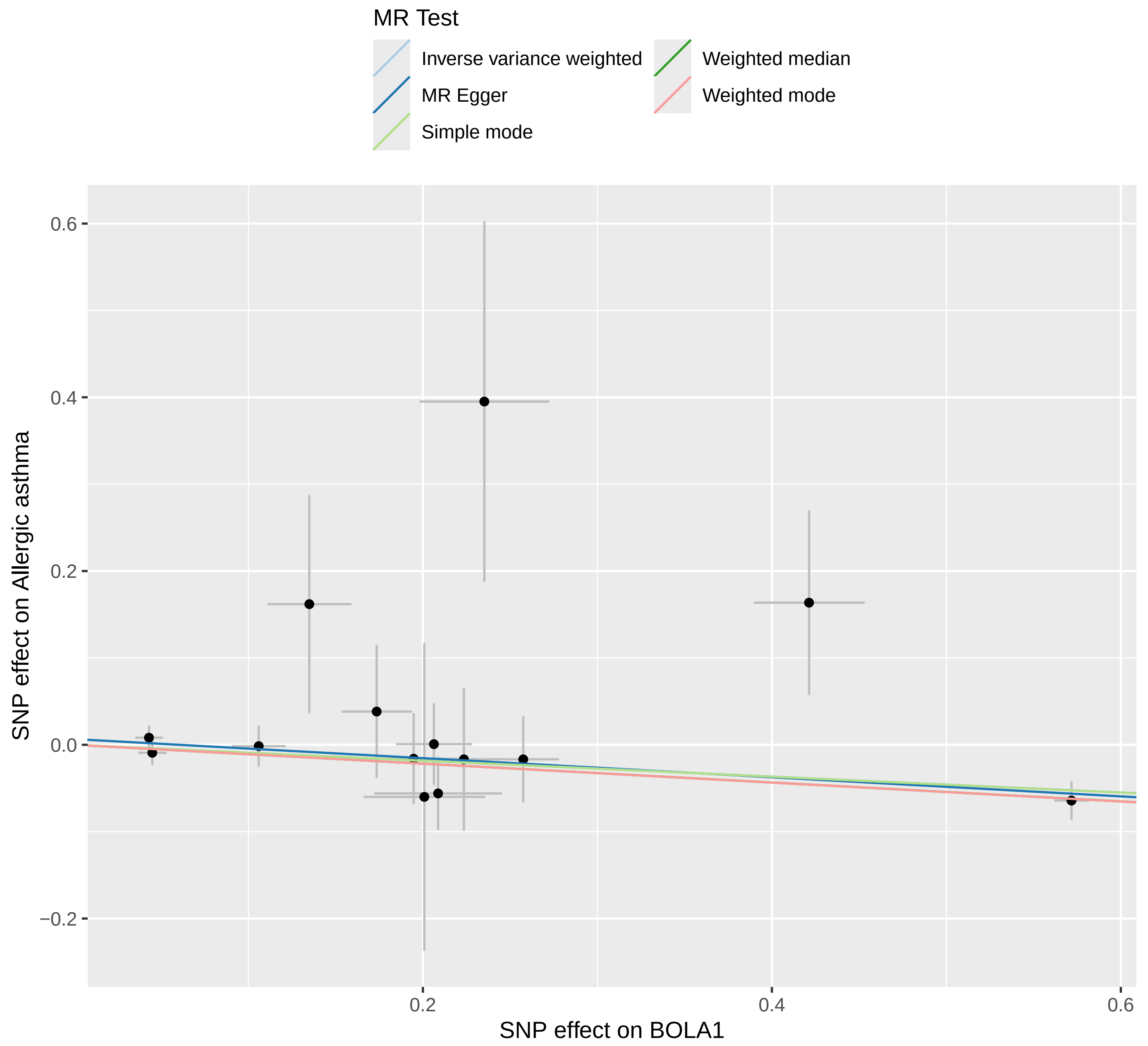

MR Test
Inverse variance weighted
MR Egger
Simple mode
Weighted median
Weighted mode
0.6
0.4
0.2
0.0
−0.2
SNP effect on Allergic asthma
0.2
0.4
0.6
SNP effect on BOLA1

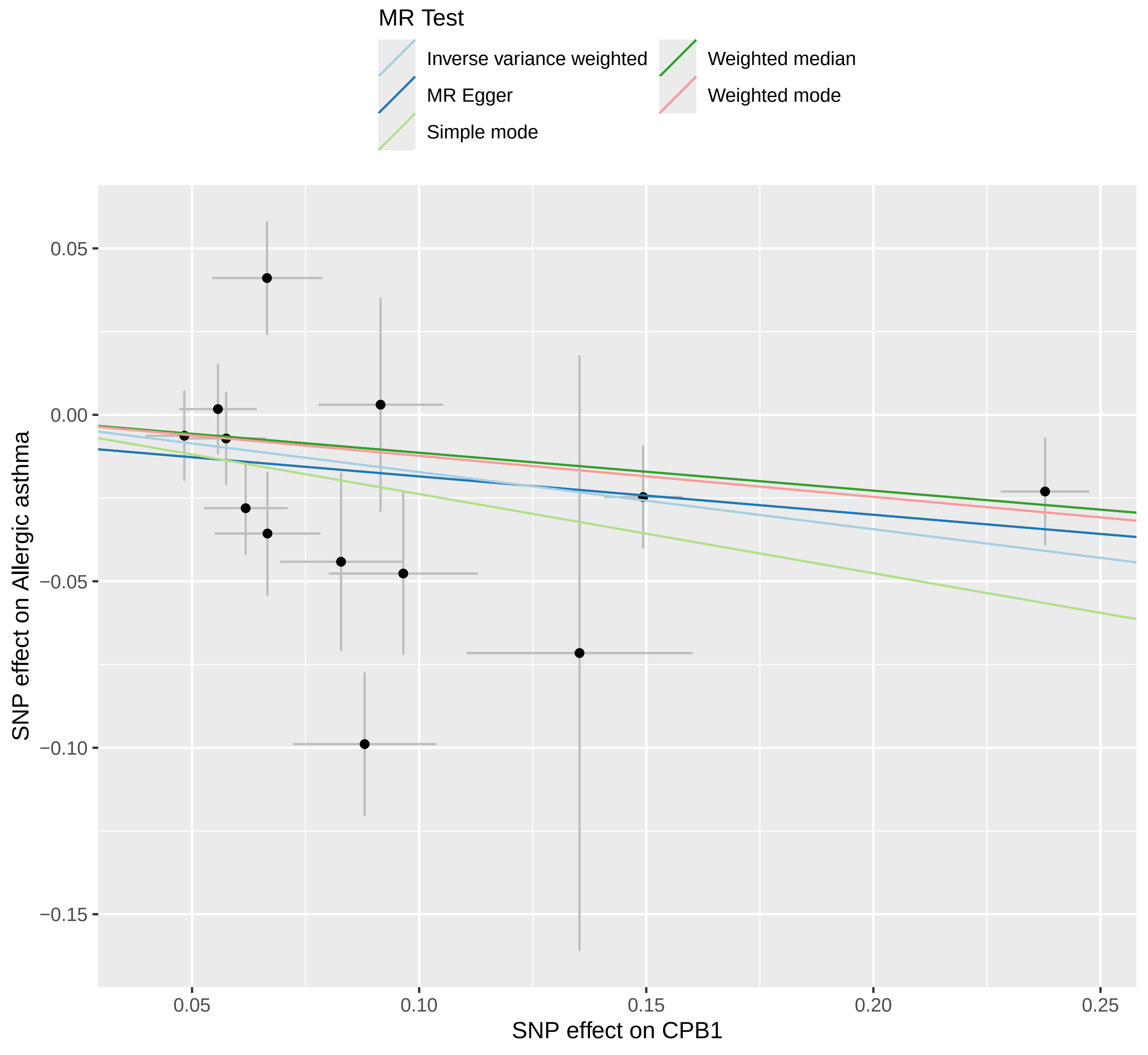

MR Test
Inverse variance weighted
MR Egger
Simple mode
Weighted median
Weighted mode
0.05
0.00
−0.05
−0.10
−0.15
SNP effect on Allergic asthma
0.05
0.10
0.15
0.20
0.25
SNP effect on CPB1

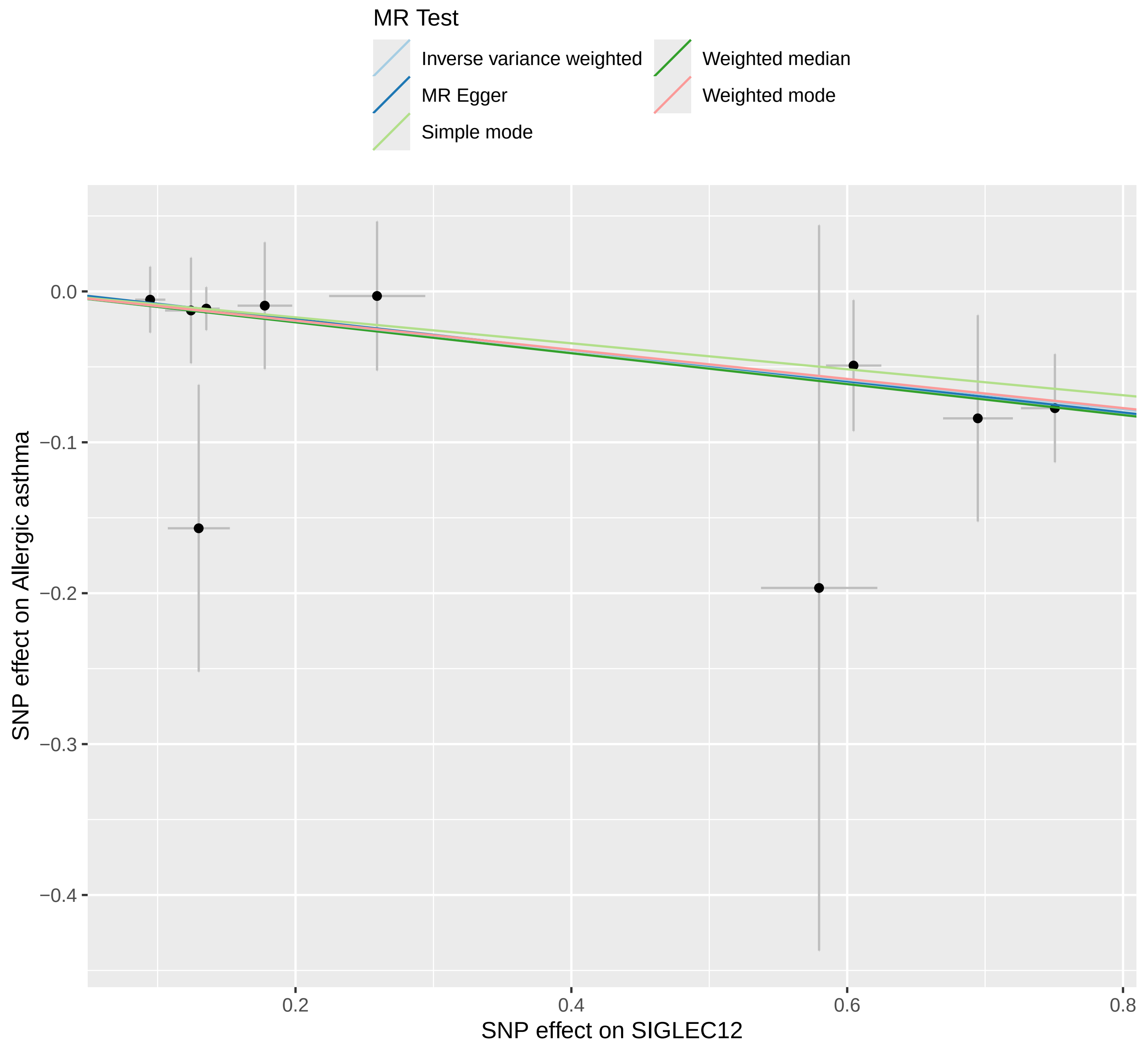
MR Test
Inverse variance weighted
MR Egger
Simple mode
Weighted median
Weighted mode
0.0
−0.1
−0.2
−0.3
−0.4
0.2
0.4
0.6
0.8
SNP effect on Allergic asthma
SNP effect on SIGLEC12

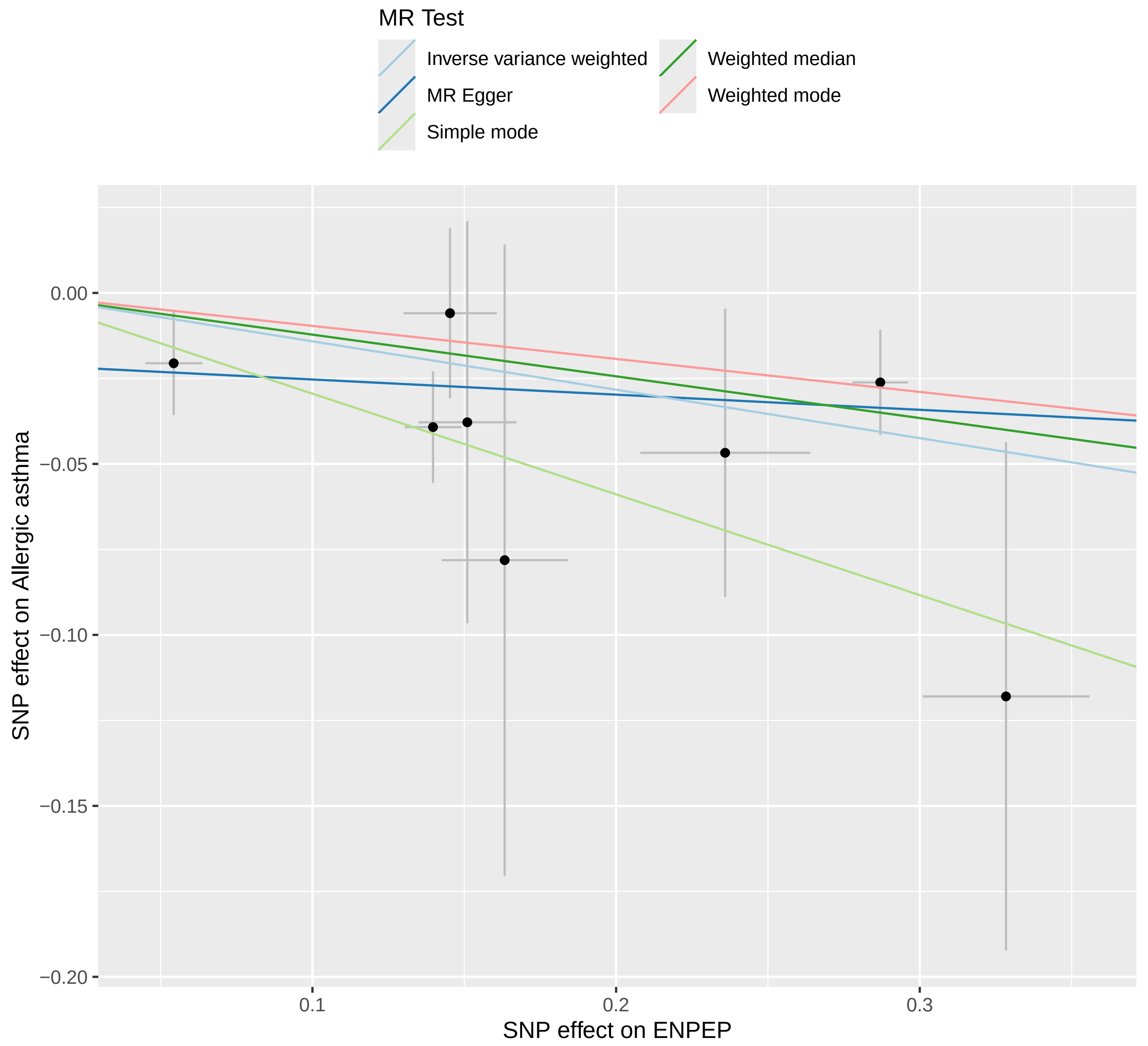
MR Test
Inverse variance weighted
MR Egger
Simple mode
Weighted median
Weighted mode
0.00
−0.05
−0.10
−0.15
−0.20
0.1
0.2
0.3
SNP effect on Allergic asthma
SNP effect on ENPEP

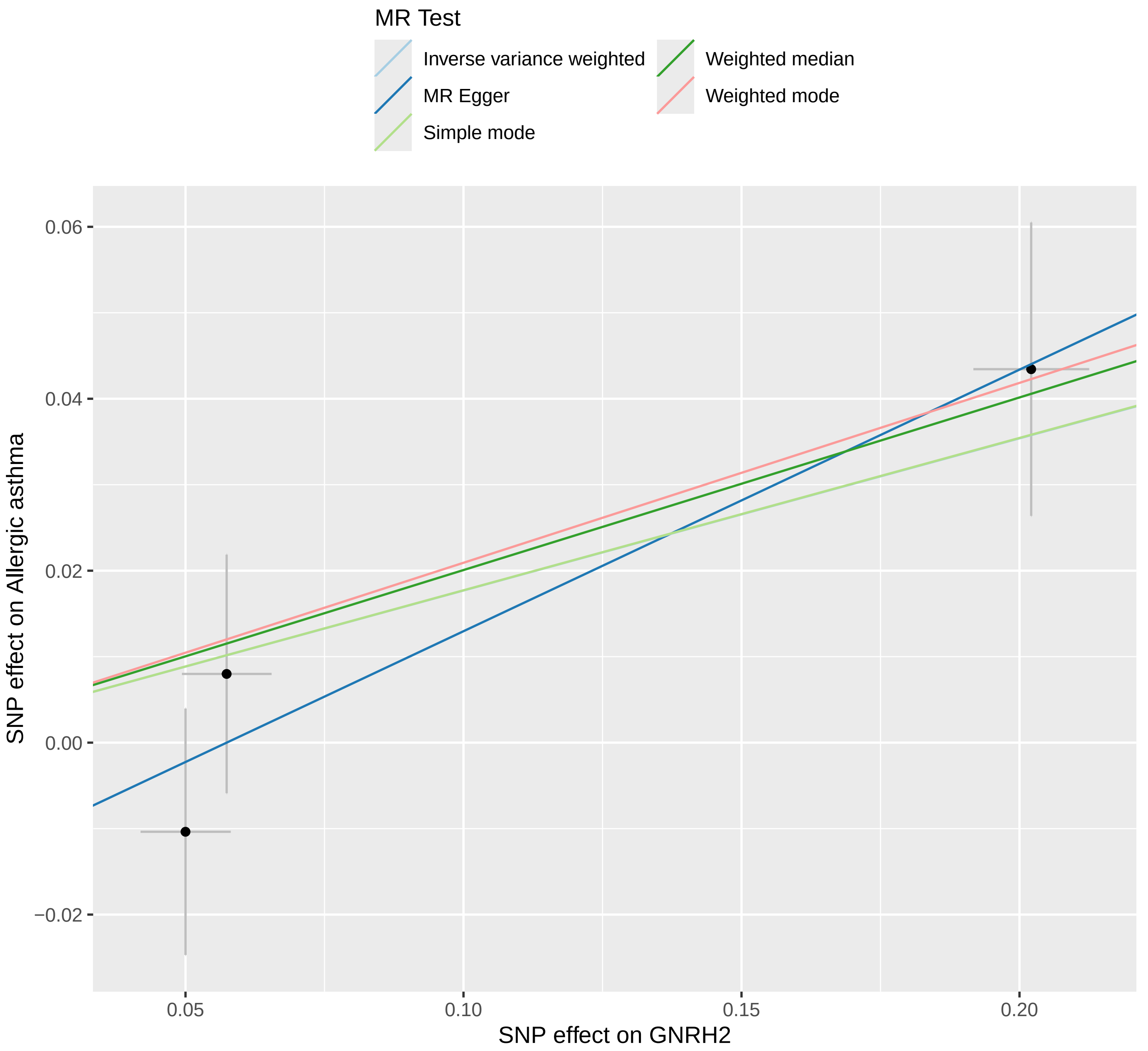

MR Test
Inverse variance weighted
MR Egger
Simple mode
Weighted median
Weighted mode
0.06
0.04
0.02
0.00
−0.02
SNP effect on Allergic asthma
0.05
0.10
0.15
0.20
SNP effect on GNRH2

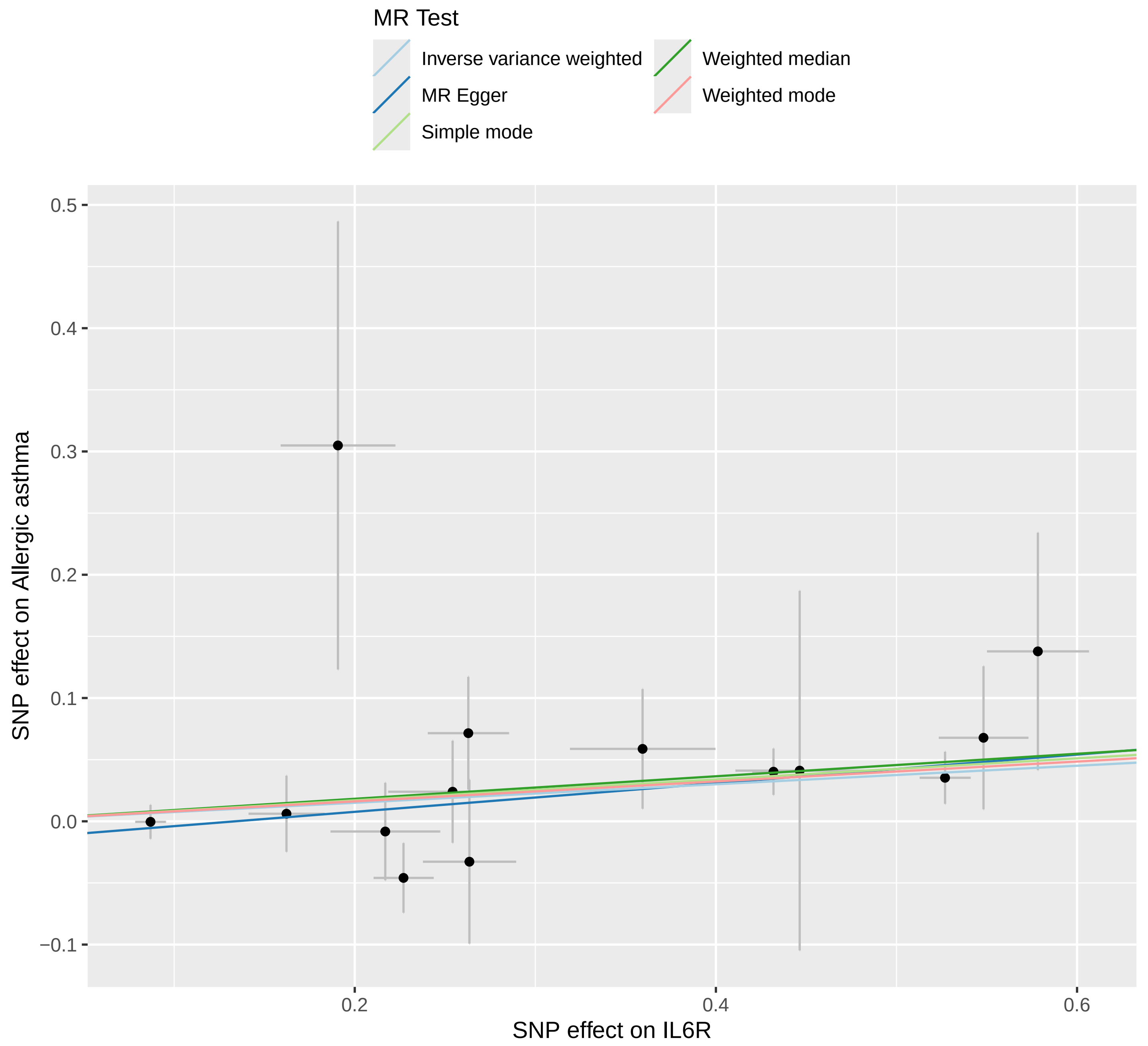
MR Test
Inverse variance weighted
MR Egger
Simple mode
Weighted median
Weighted mode
0.5
0.4
0.3
0.2
0.1
0.0
−0.1
SNP effect on Allergic asthma
0.2
0.4
0.6
SNP effect on IL6R

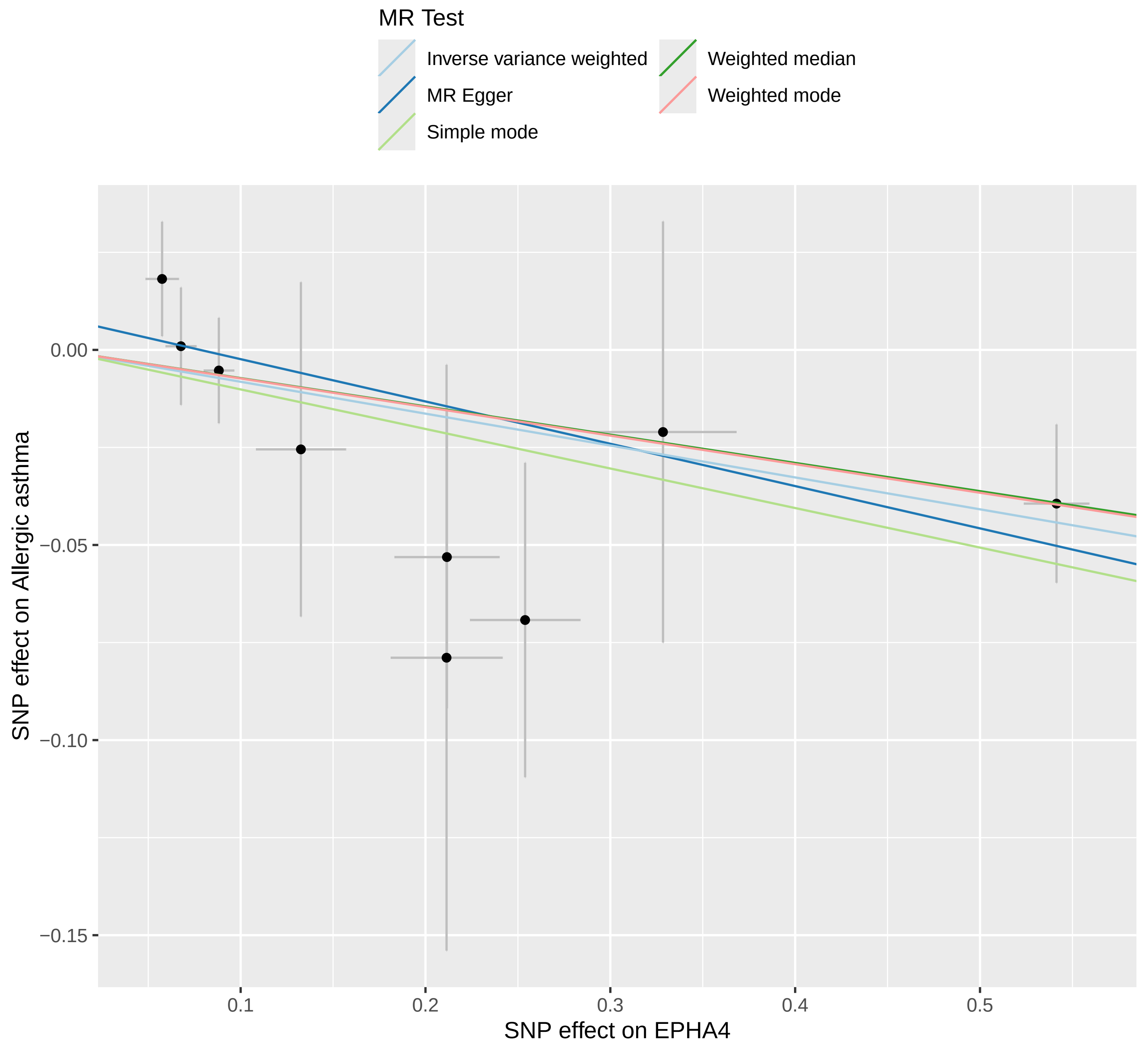
MR Test
Inverse variance weighted
MR Egger
Simple mode
Weighted median
Weighted mode
0.00
−0.05
−0.10
−0.15
0.1
0.2
0.3
0.4
0.5
SNP effect on Allergic asthma
SNP effect on EPHA4
