# Supplementary material for: Mediating Mendelian randomization in the proteome identified potential drug targets for obesity-related allergic asthma
Source: Hereditas. 2025 Feb 1;162:12. doi: 10.1186/s41065-025-00376-w (PMC11786417; doi:10.1186/s41065-025-00376-w)
Supplement: Supplementary file 1 — Supplementary Material 1: Supplementary Materials 1. The MR results of BMI and allergic asthma. Supplementary Materials 2. The MR results of plasma proteins and allergic asthma. Supplementary Materials 3. The MR results of BMI and plasma proteins. Supplementary Materials 4. The MR results of mediation factor plasma proteins and allergic asthma. Supplementary Materials 5. The results of drug-targeted MR [file 41065_2025_376_MOESM1_ESM.zip › Supplementary Materials/S3 The MR results of BMI and plasma proteins/S3 BMI-plasma proteins.forest.pdf]

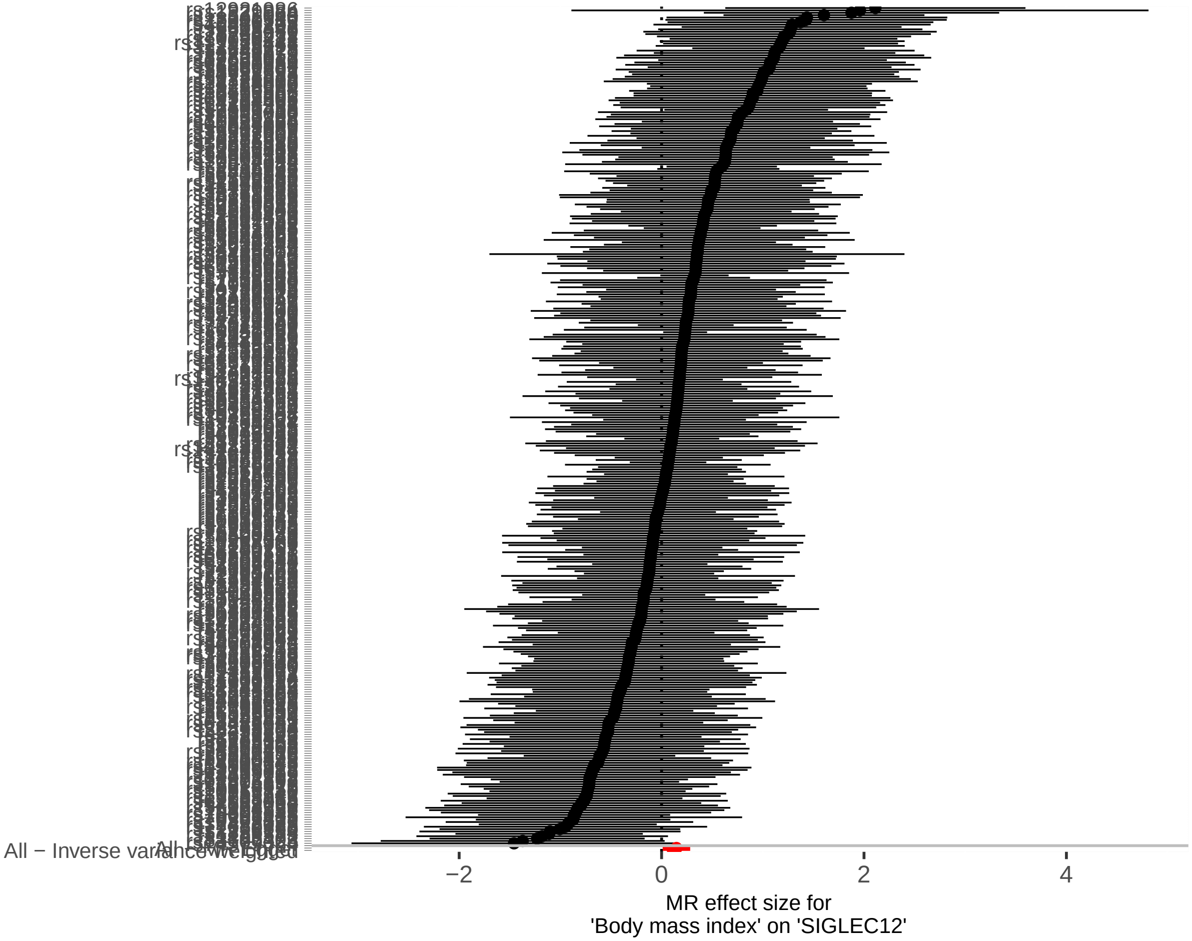

All − Inverse variance weighted
−2
0
2
4
MR effect size for
'Body mass index' on 'SIGLEC12'

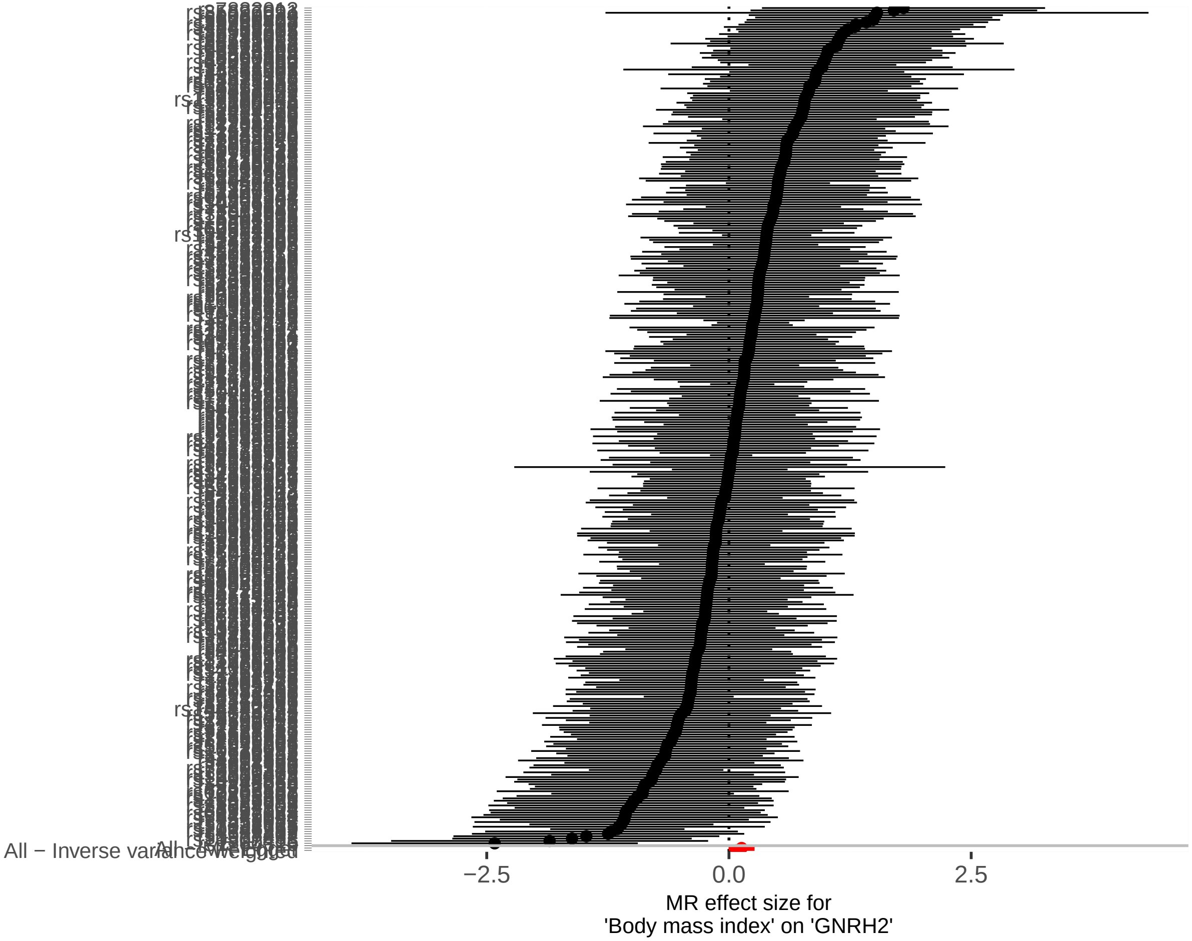

All − Inverse variance weighted
−2.5
0.0
2.5
MR effect size for
'Body mass index' on 'GNRH2'

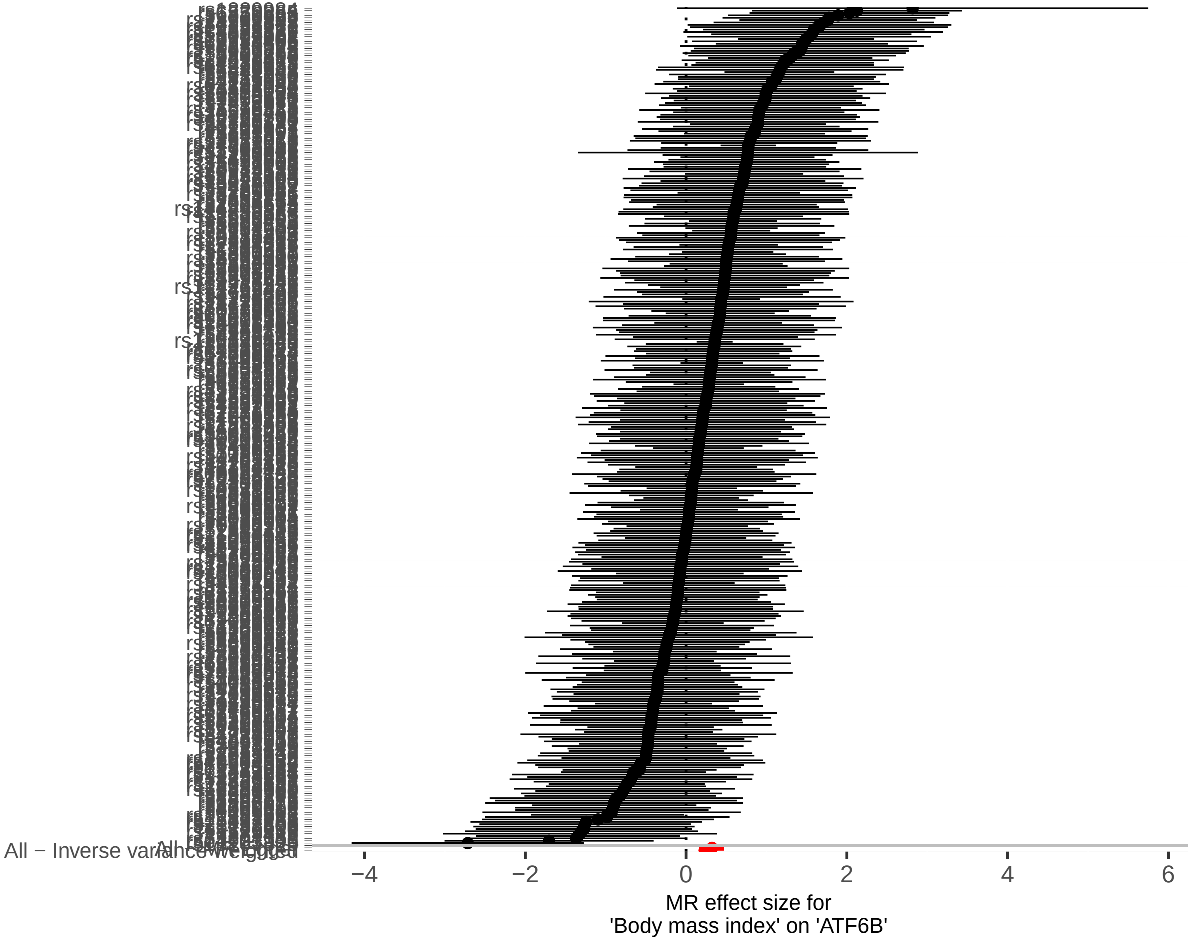
All − Inverse variance weighted
All − MR Egger
−4
−2
0
2
4
6
MR effect size for
'Body mass index' on 'ATF6B'

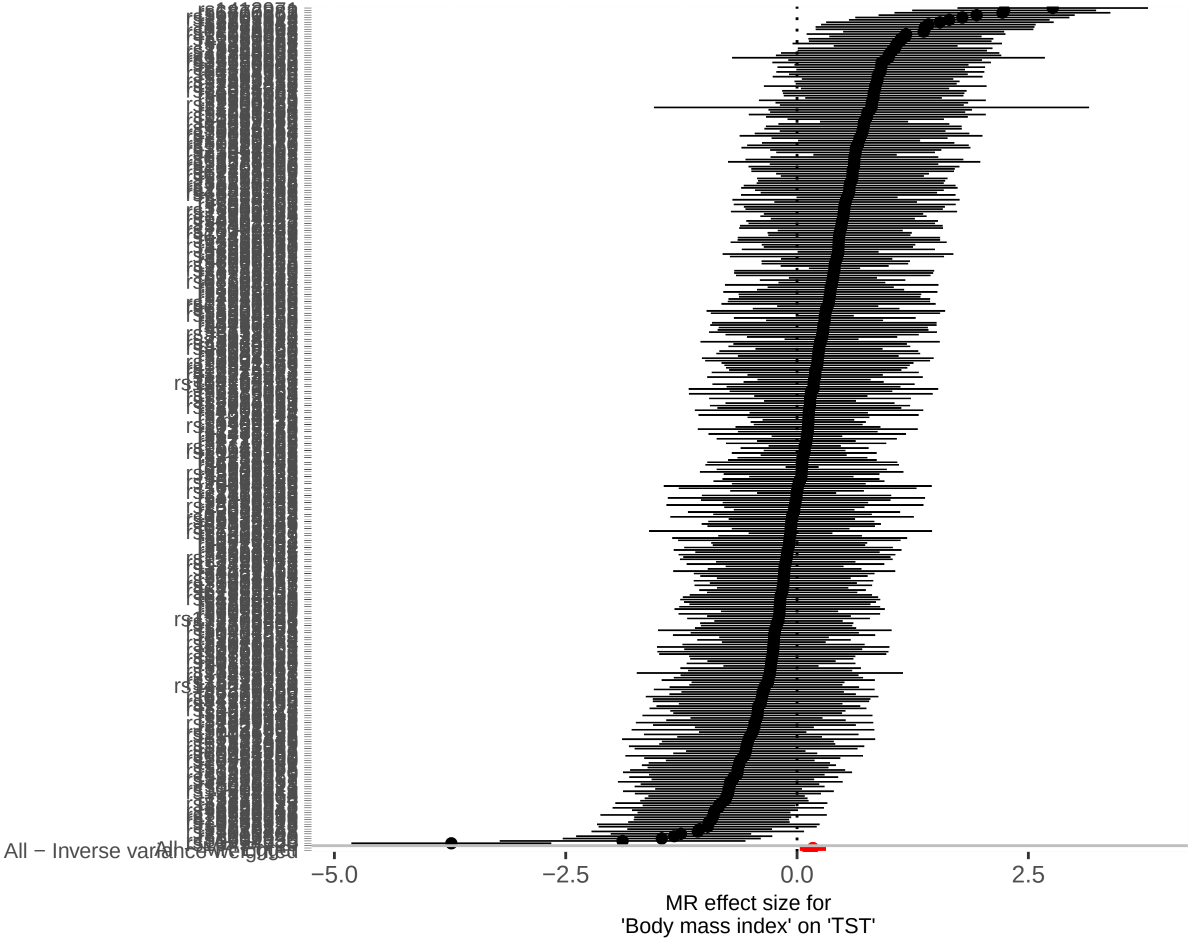
All − Inverse variance weighted
−5.0
−2.5
0.0
2.5
MR effect size for
'Body mass index' on 'TST'

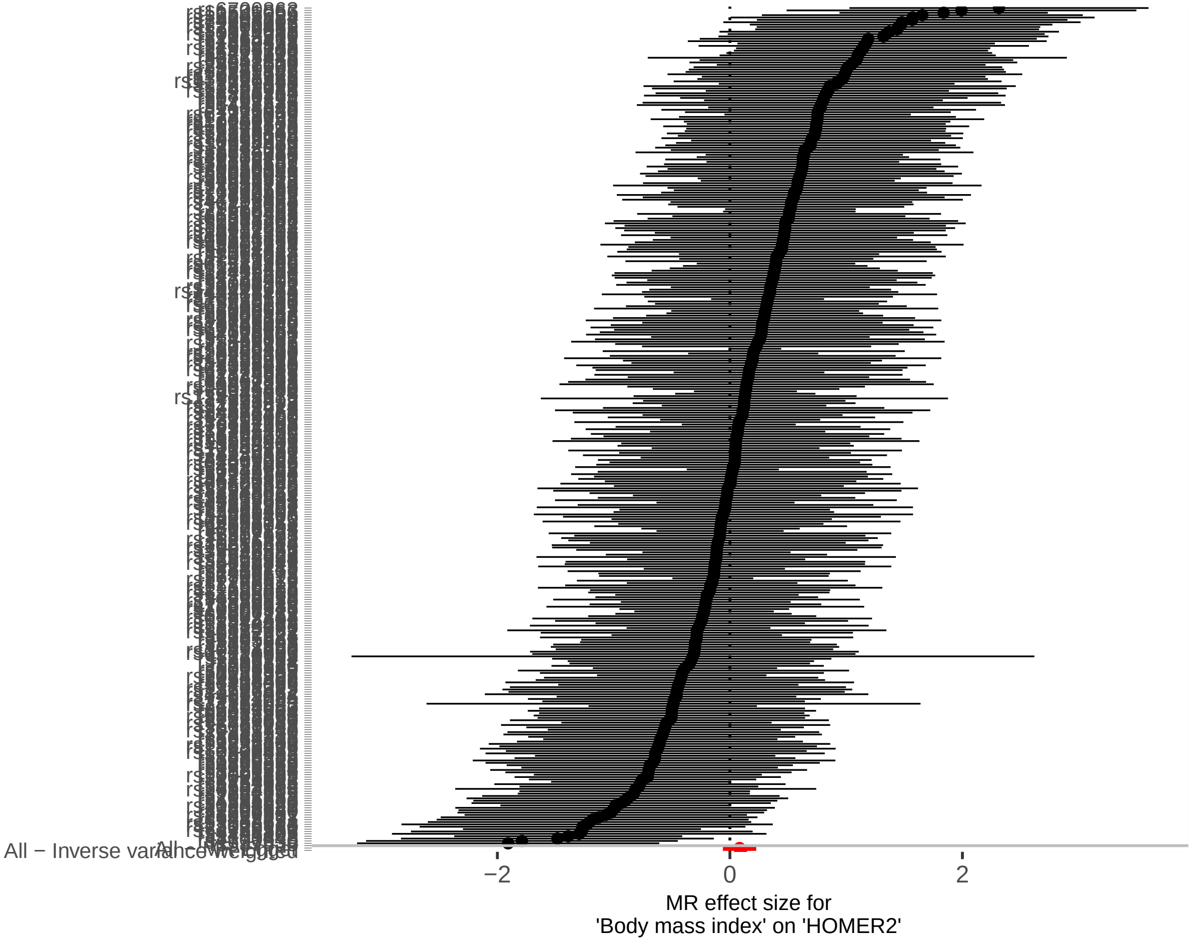
All − Inverse variance weighted
−2
0
2
MR effect size for
'Body mass index' on 'HOMER2'

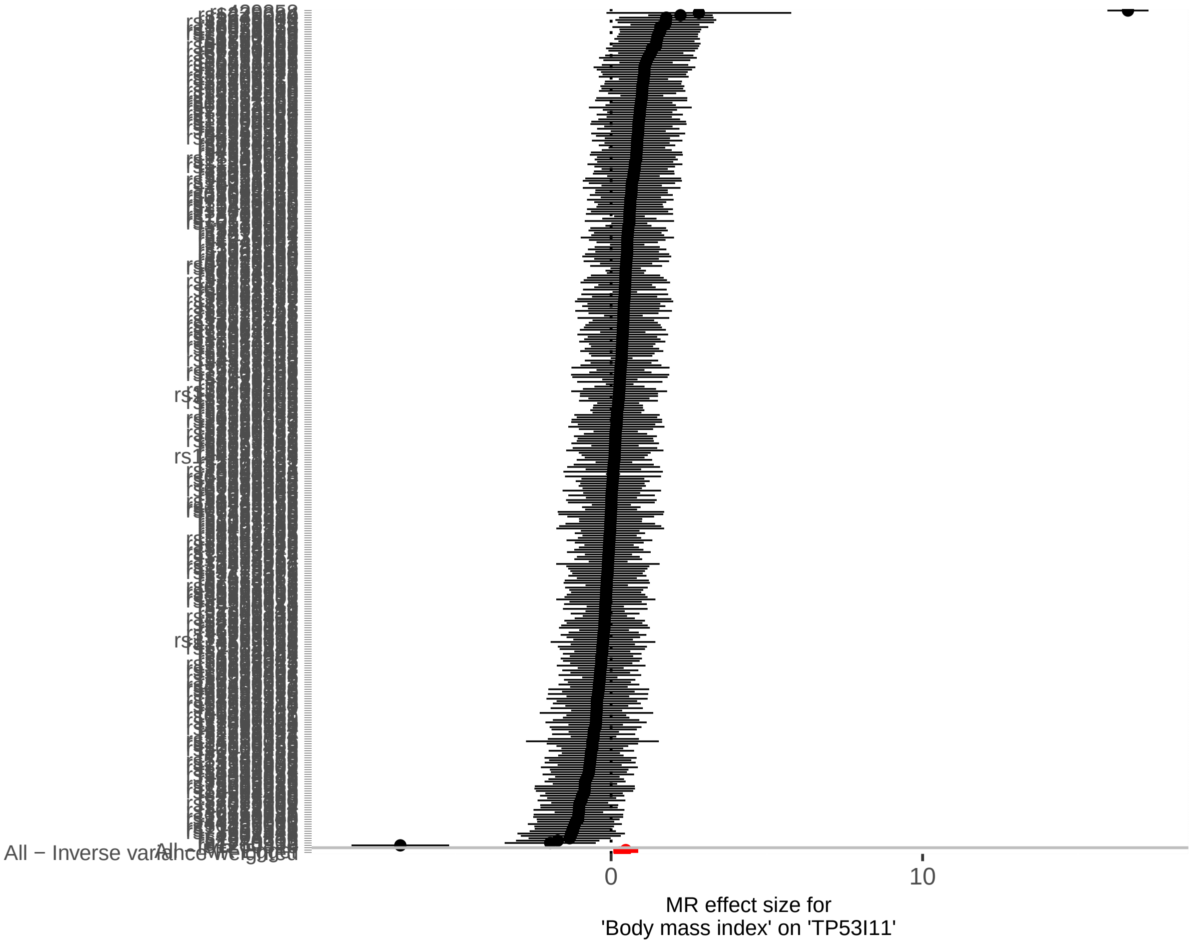
0
10
MR effect size for
'Body mass index' on 'TP53I11'

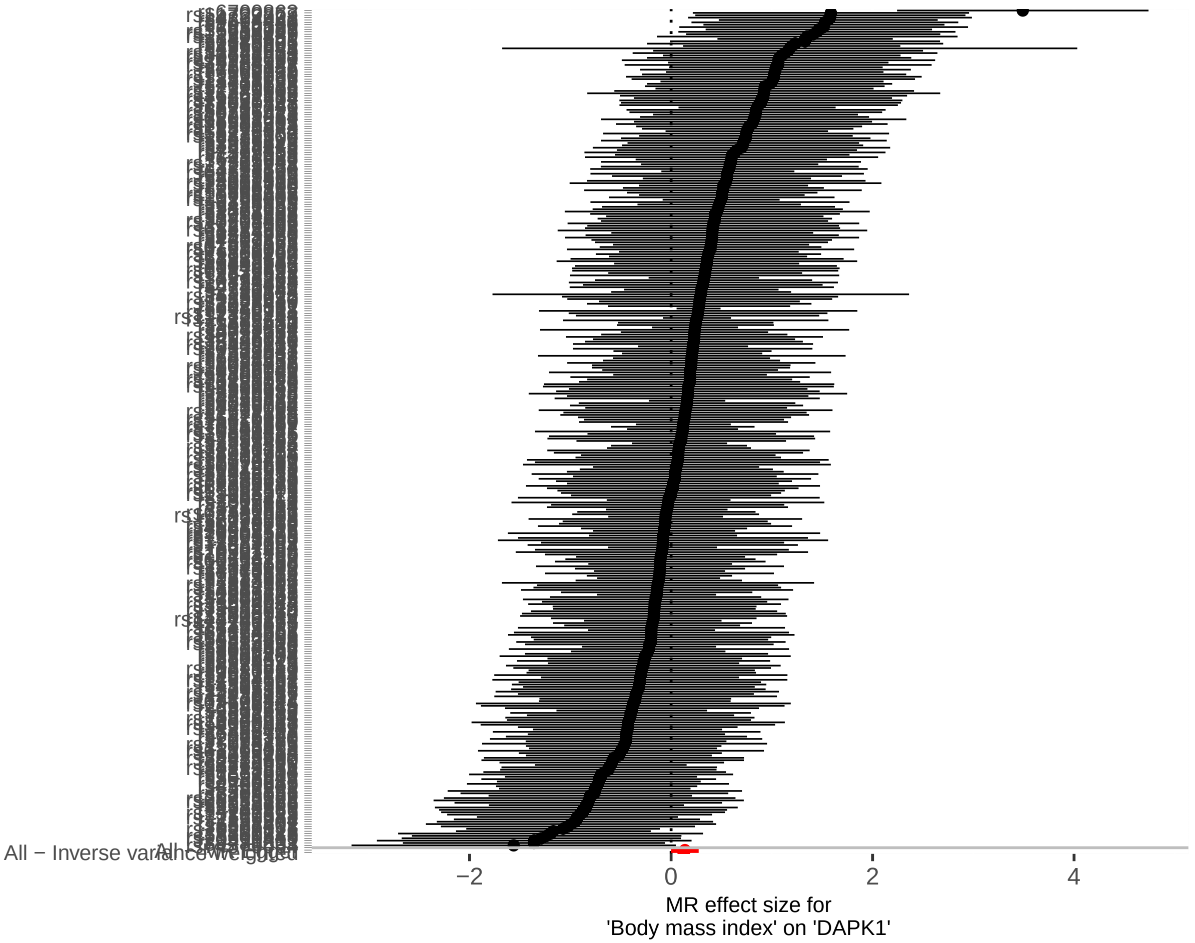

−2
0
2
4
MR effect size for
'Body mass index' on 'DAPK1'

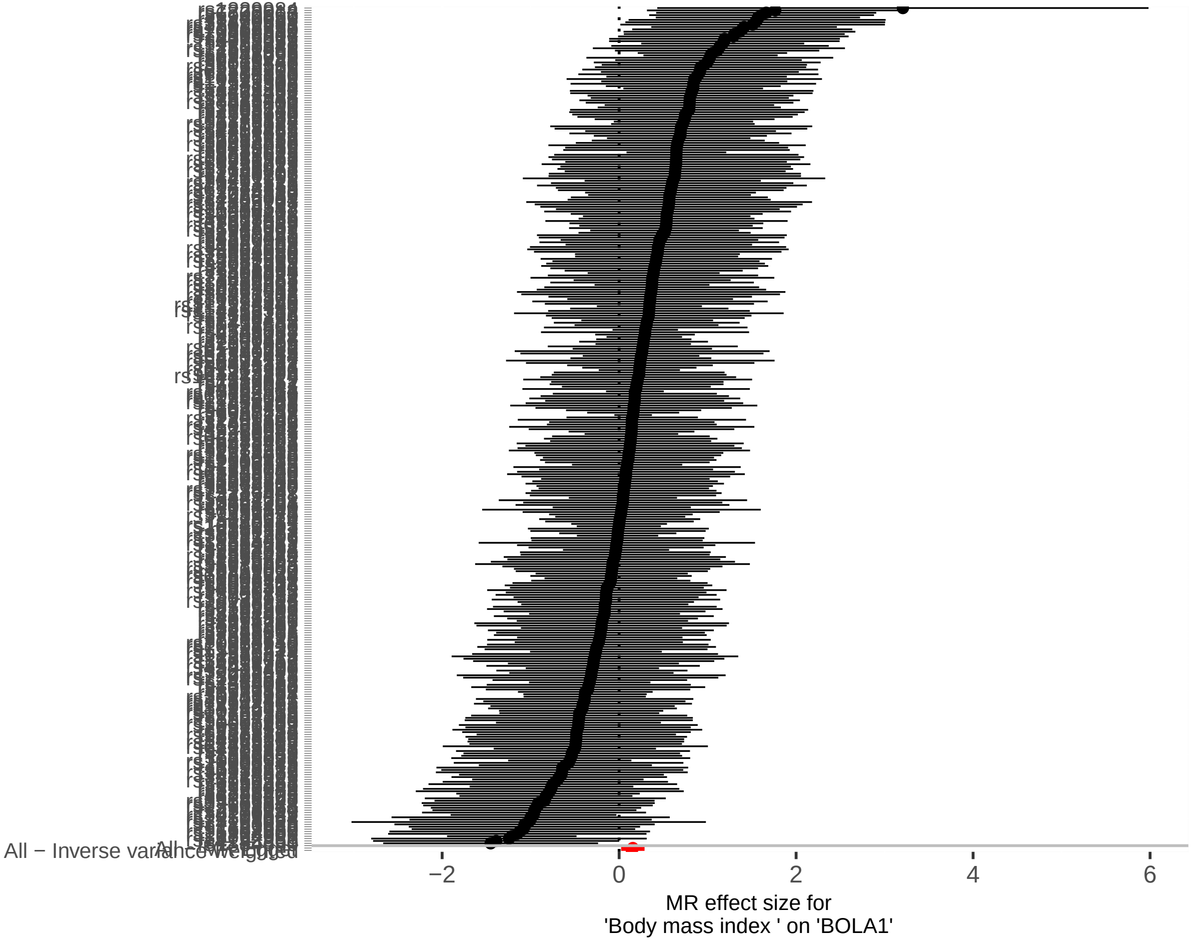
All − Inverse variance weighted
−2
0
2
4
6
MR effect size for
'Body mass index ' on 'BOLA1'

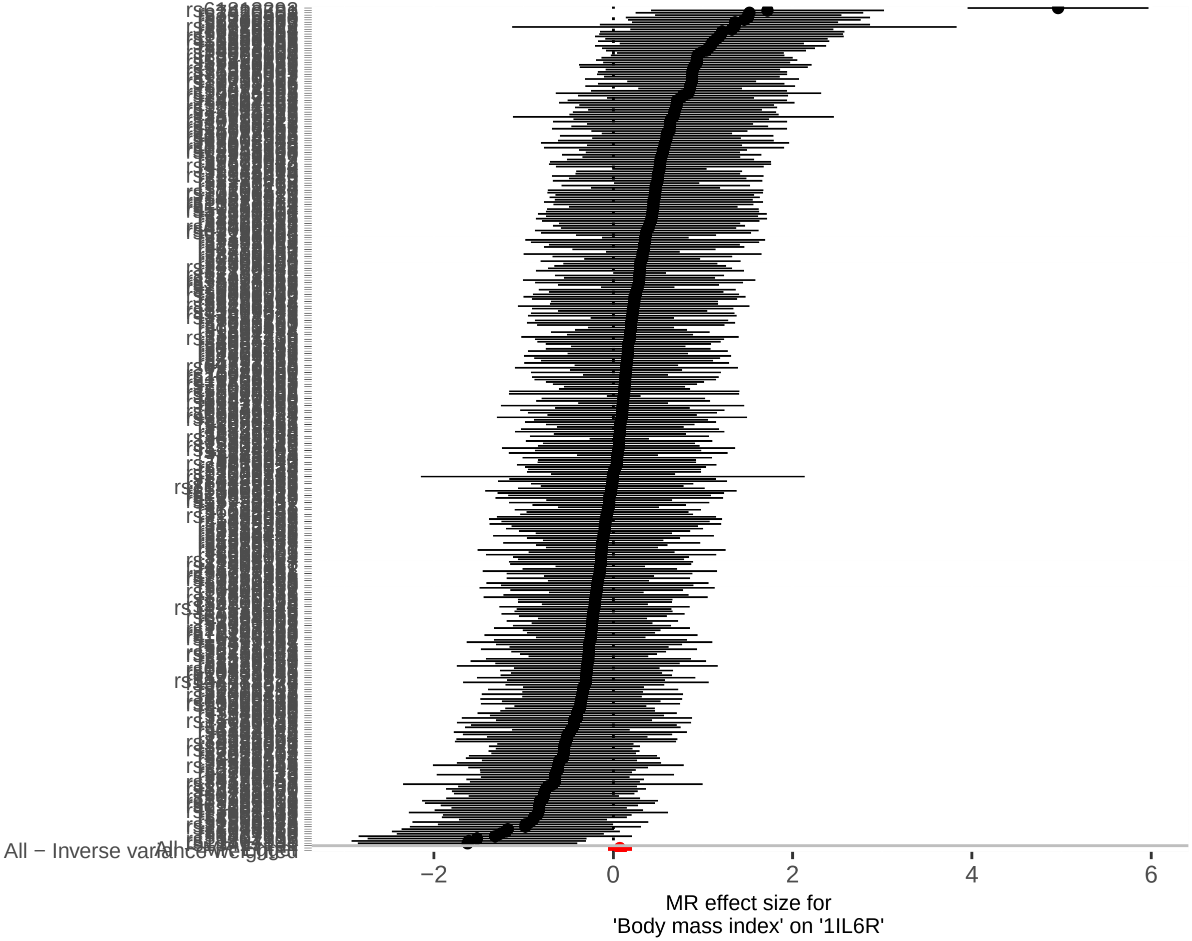
All − Inverse variance weighted
−2
0
2
4
6
MR effect size for
'Body mass index' on '1IL6R'

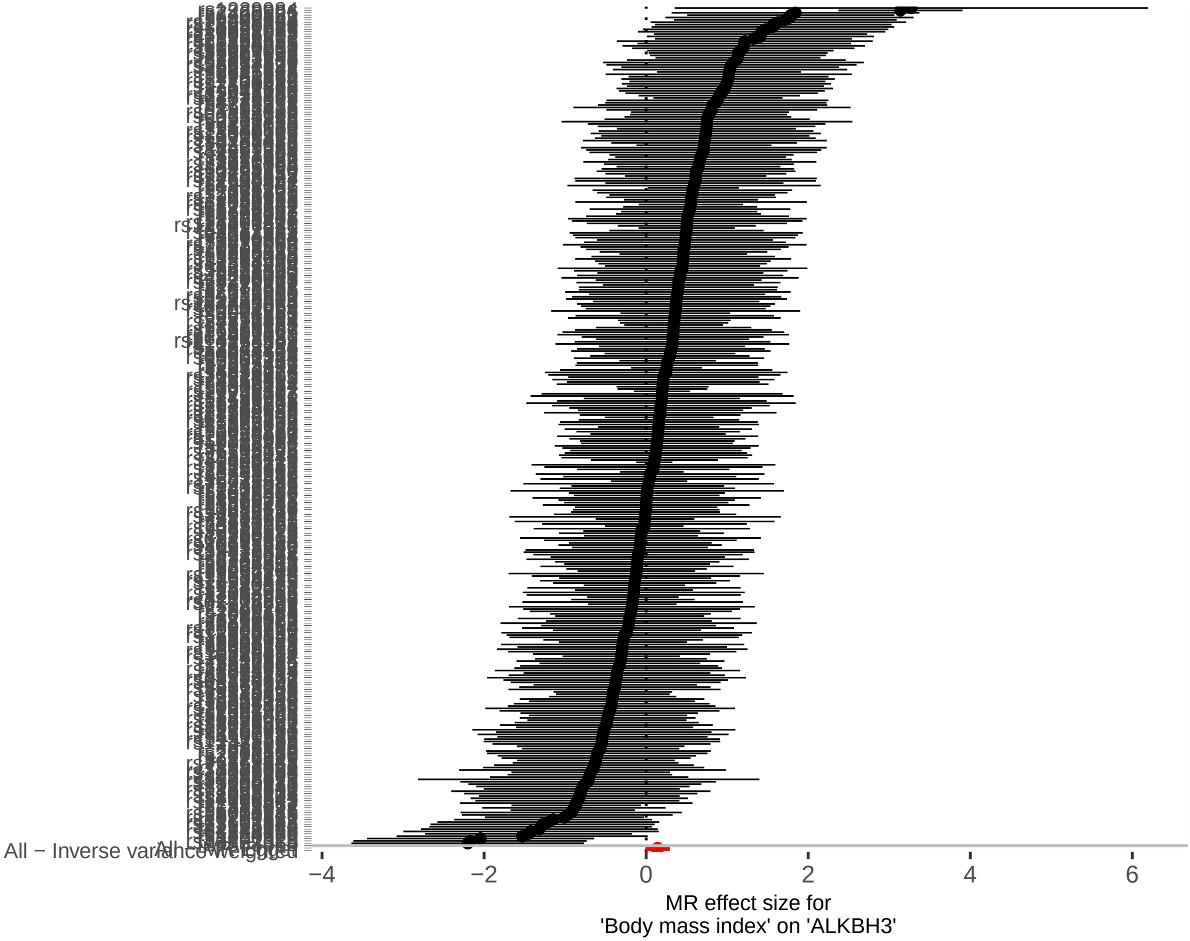
All − Inverse variance weighted
−4
−2
0
2
4
6
MR effect size for
'Body mass index' on 'ALKBH3'

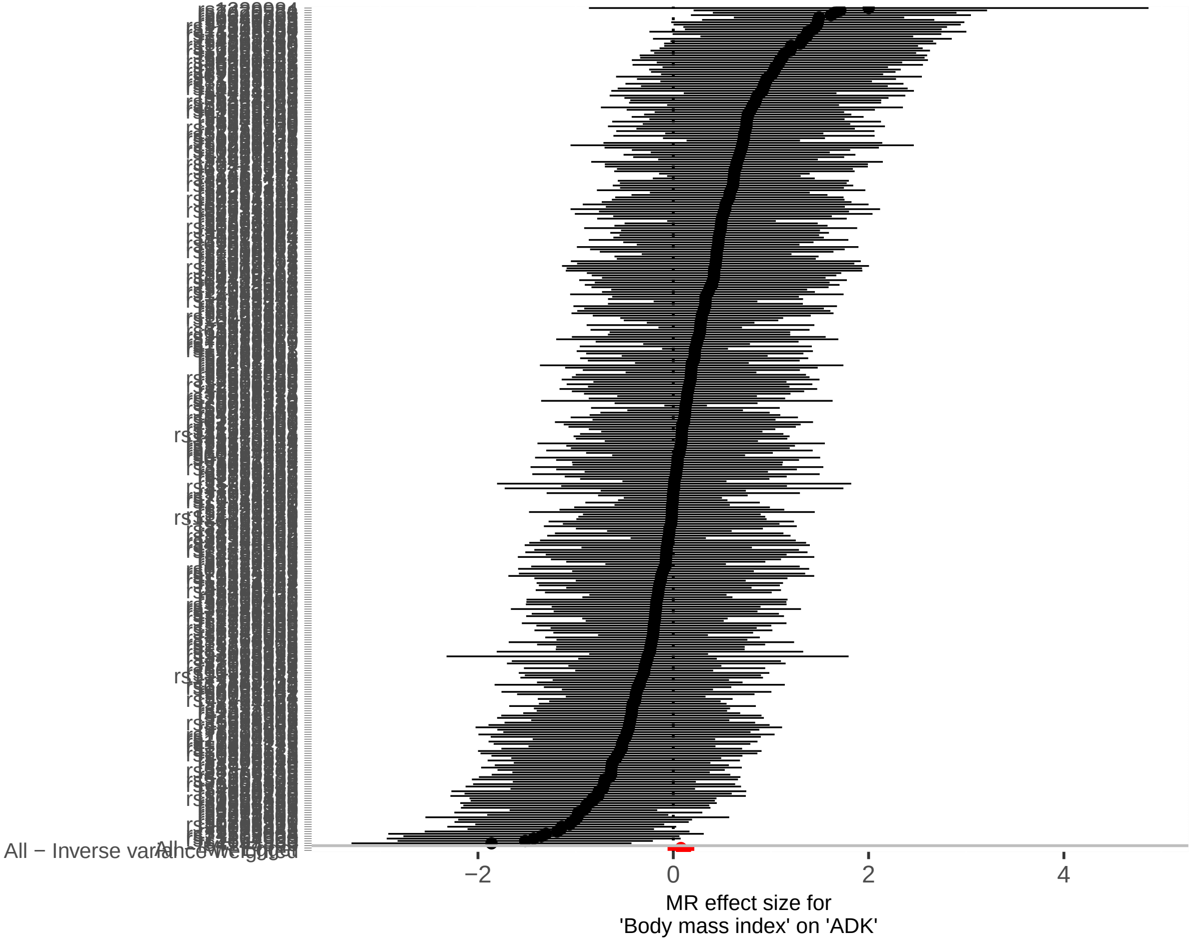
All − Inverse variance weighted
−2
0
2
4
MR effect size for
'Body mass index' on 'ADK'

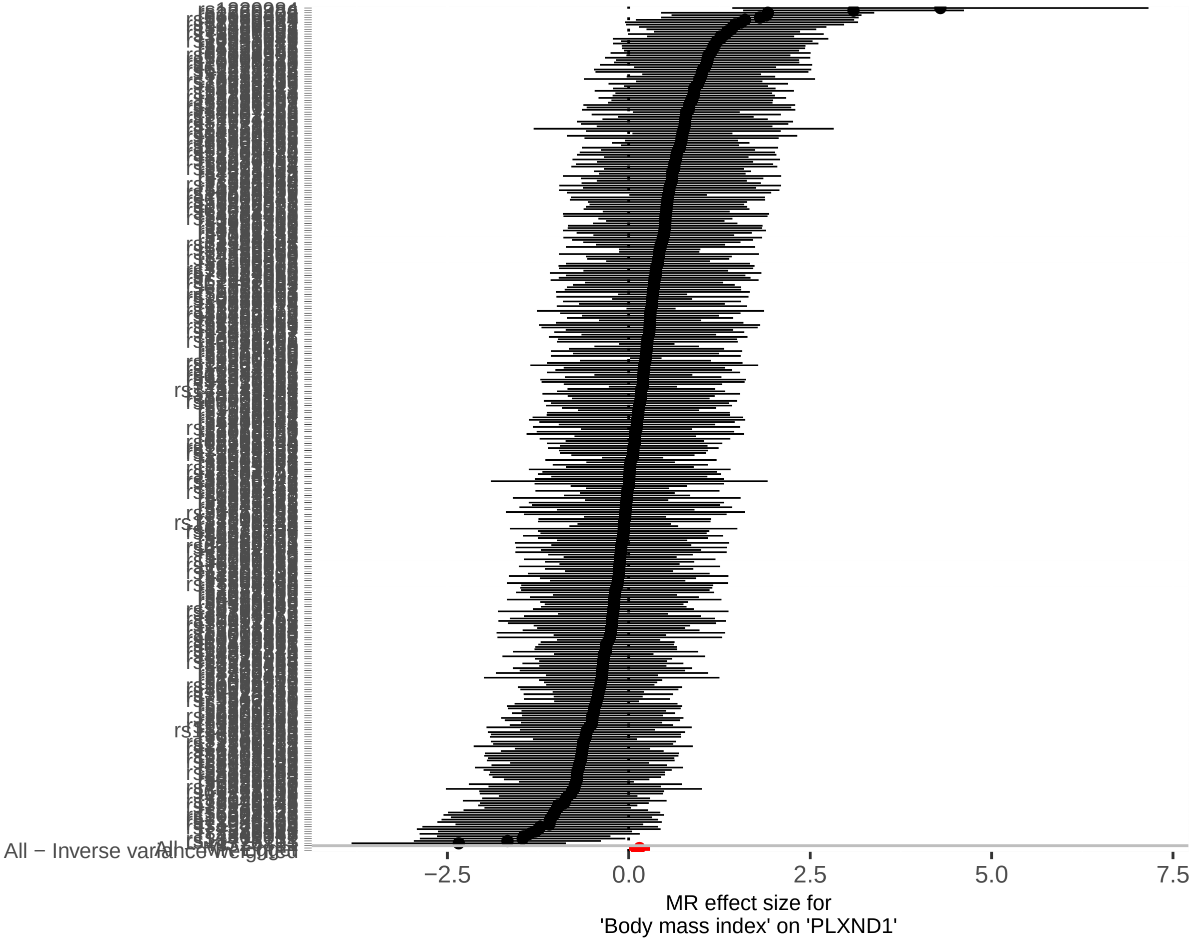

All − Inverse variance weighted
−2.5
0.0
2.5
5.0
7.5
MR effect size for
'Body mass index' on 'PLXND1'

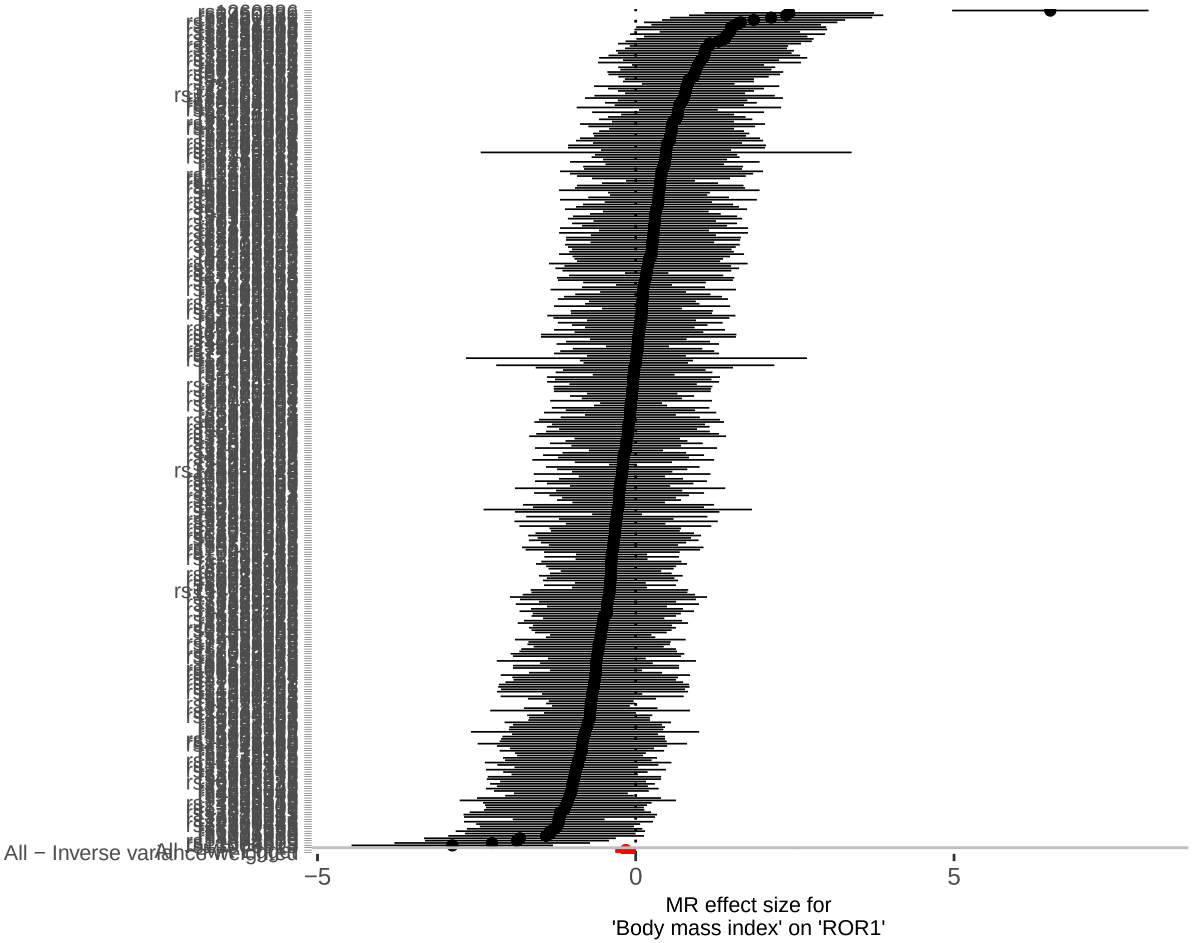
All − Inverse variance weighted
−5
0
5
MR effect size for
'Body mass index' on 'ROR1'

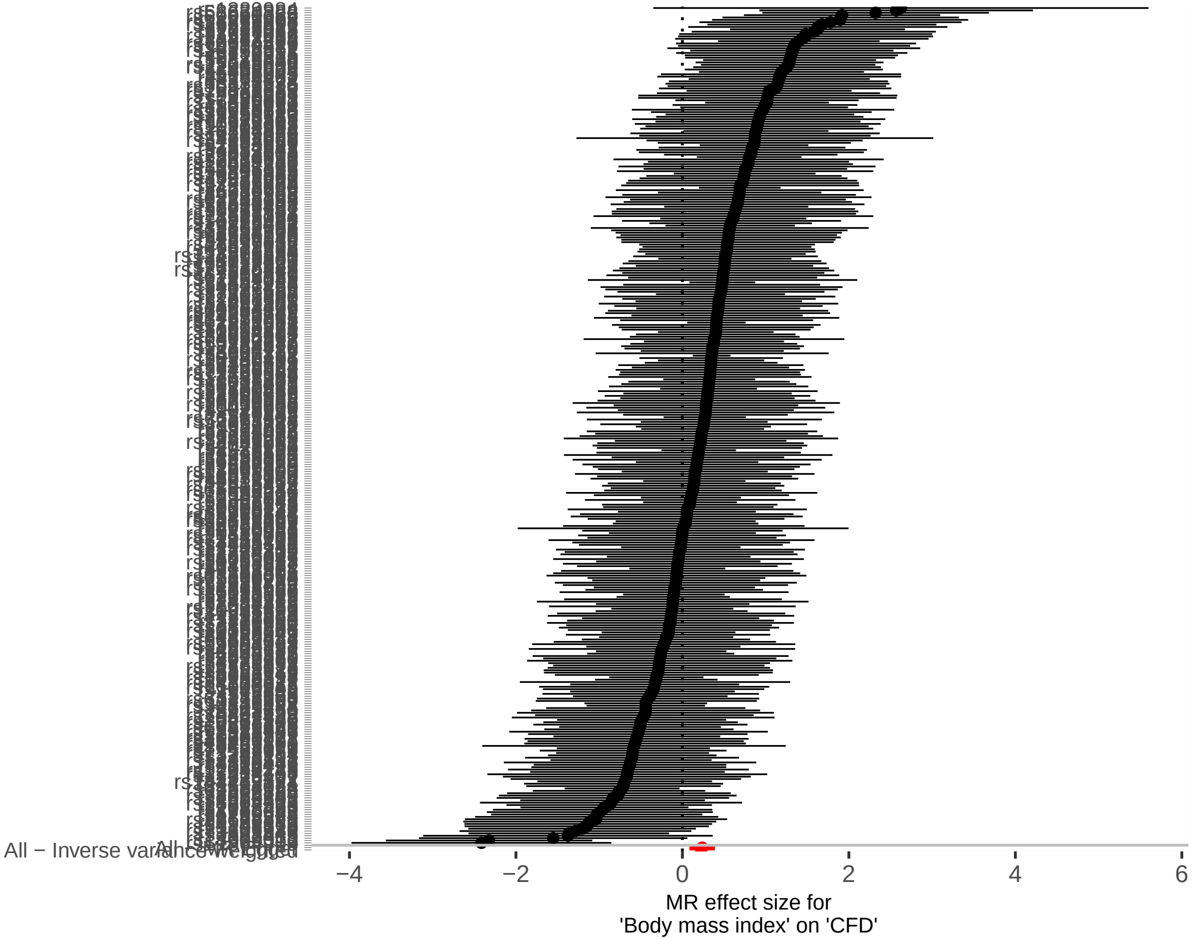
All − Inverse variance weighted
−4
−2
0
2
4
6
MR effect size for
'Body mass index' on 'CFD'

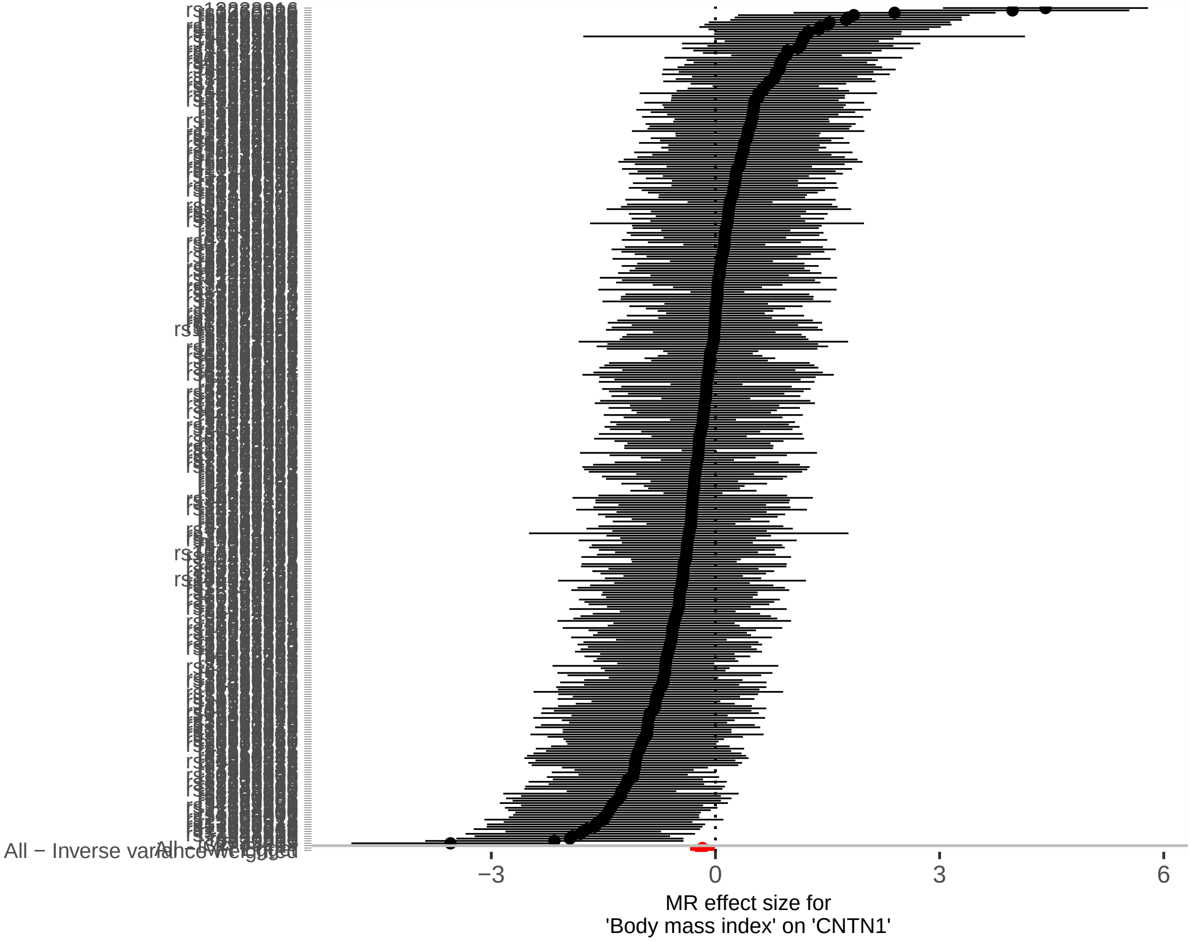

−3
0
3
6
MR effect size for
'Body mass index' on 'CNTN1'

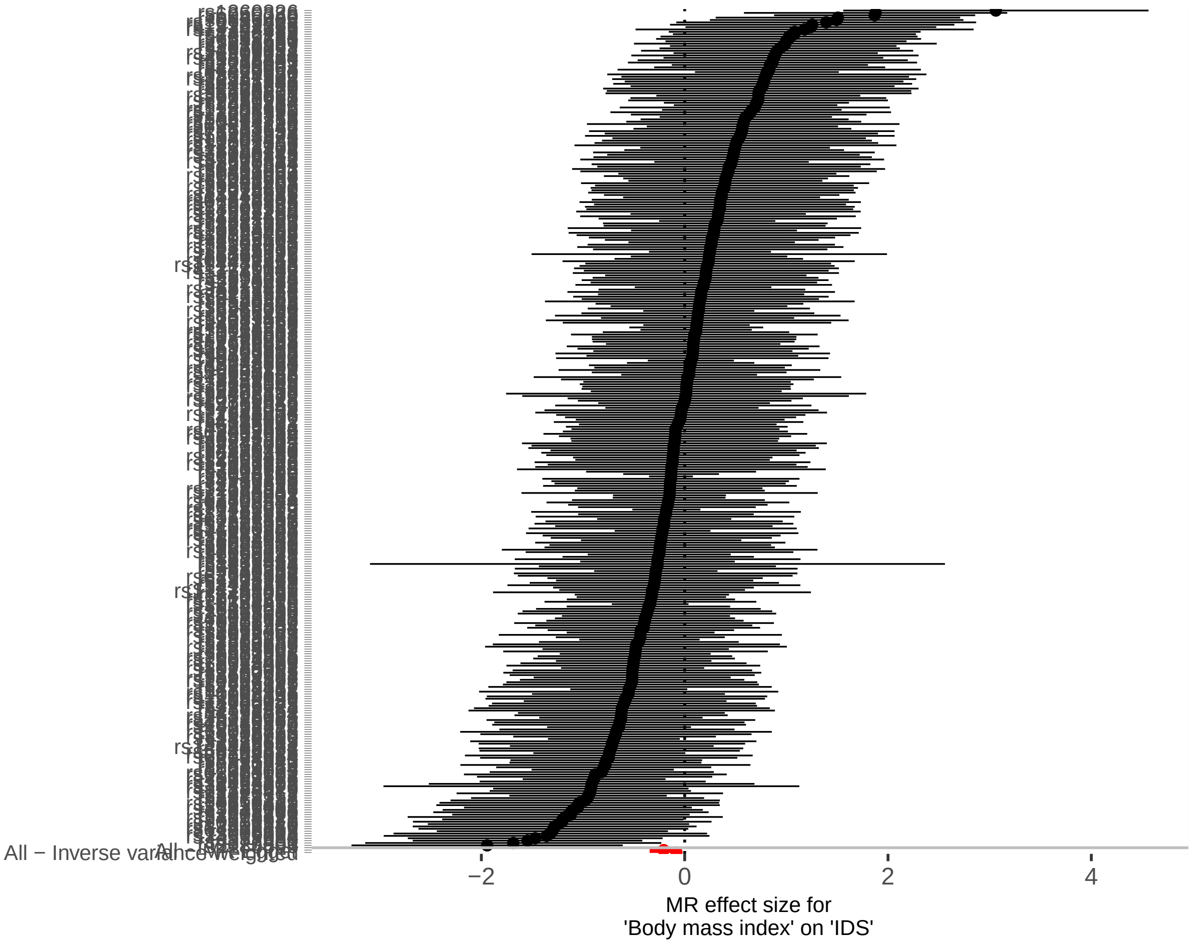

All − Inverse variance weighted
All − MR Egger
−2
0
2
4
MR effect size for
'Body mass index' on 'IDS'

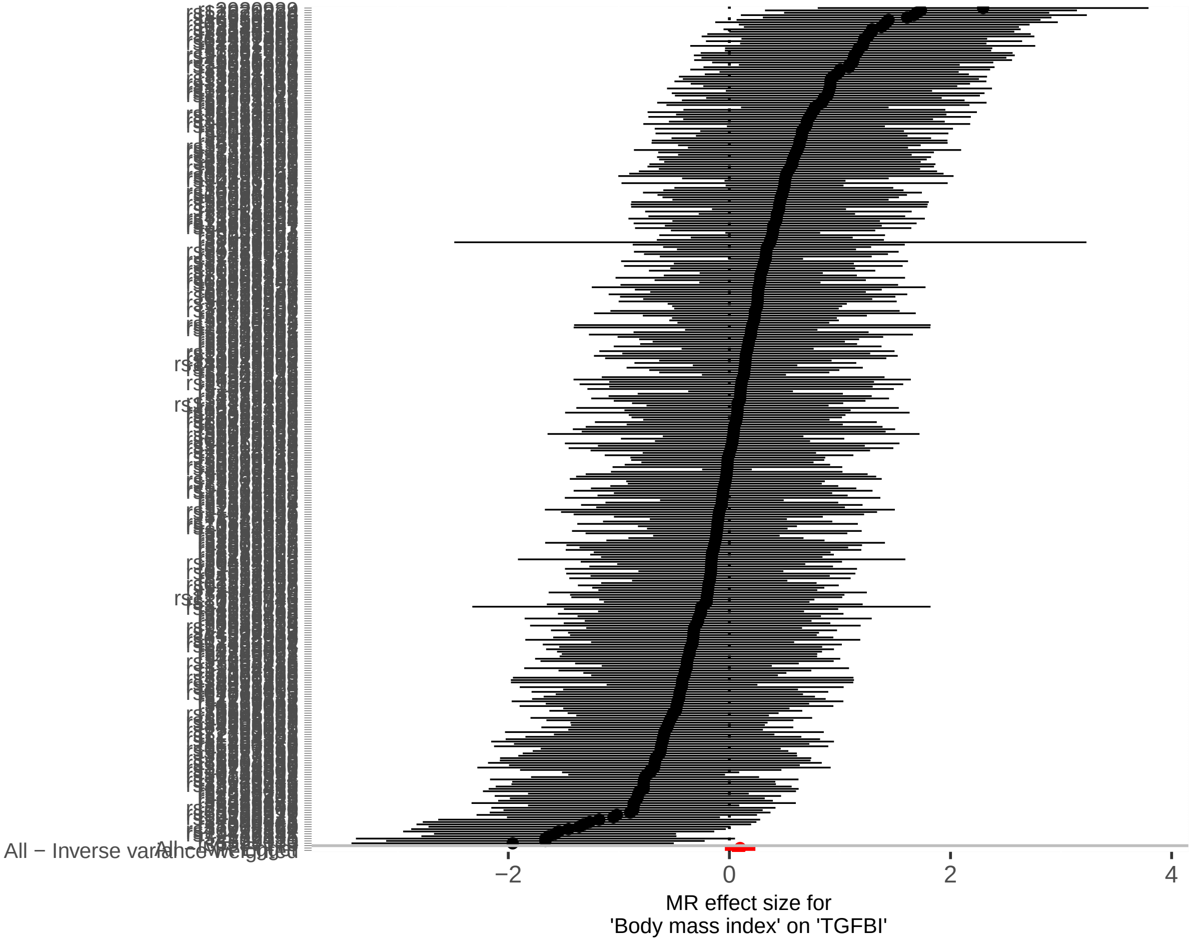
All − Inverse variance weighted
−2
0
2
4
MR effect size for
'Body mass index' on 'TGFBI'

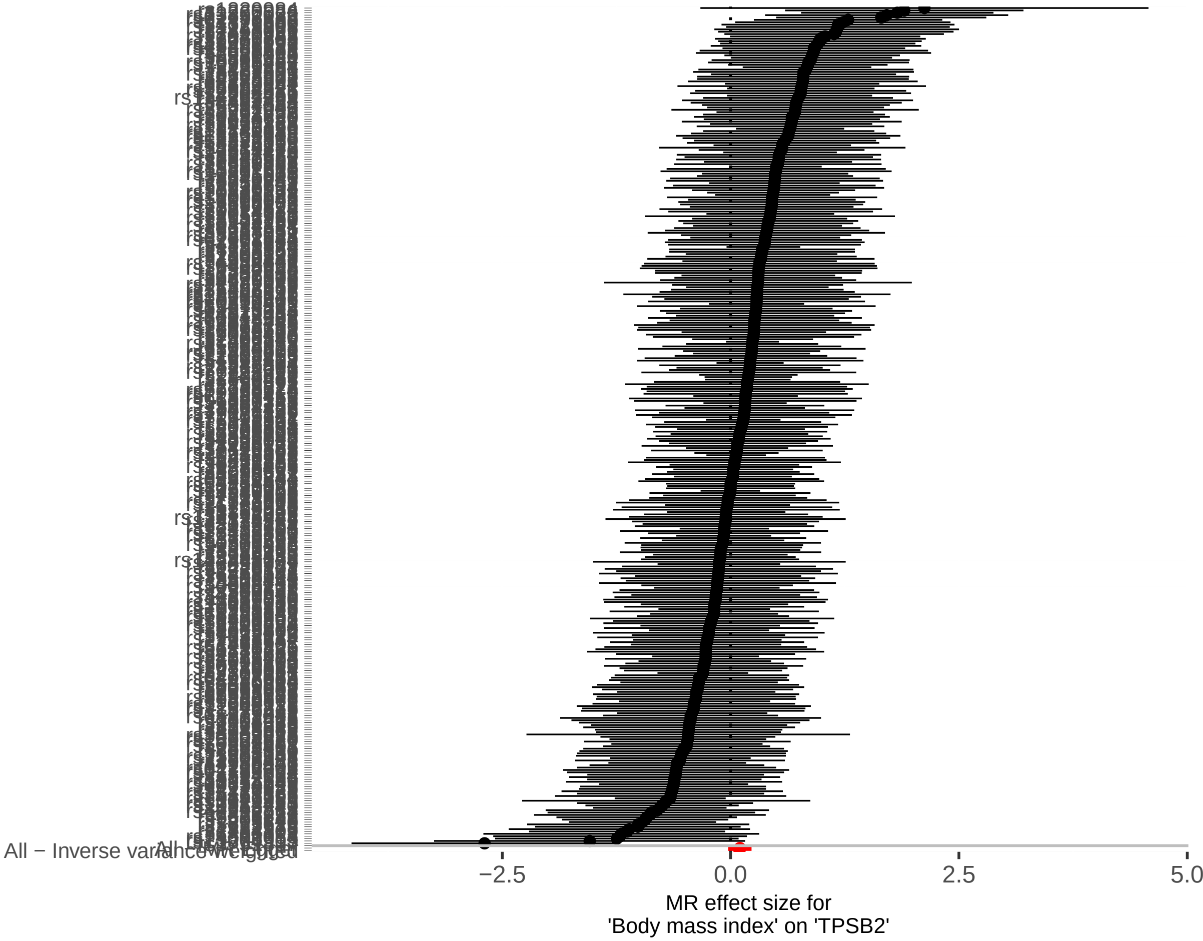
−2.5
0.0
2.5
5.0
MR effect size for
'Body mass index' on 'TPSB2'

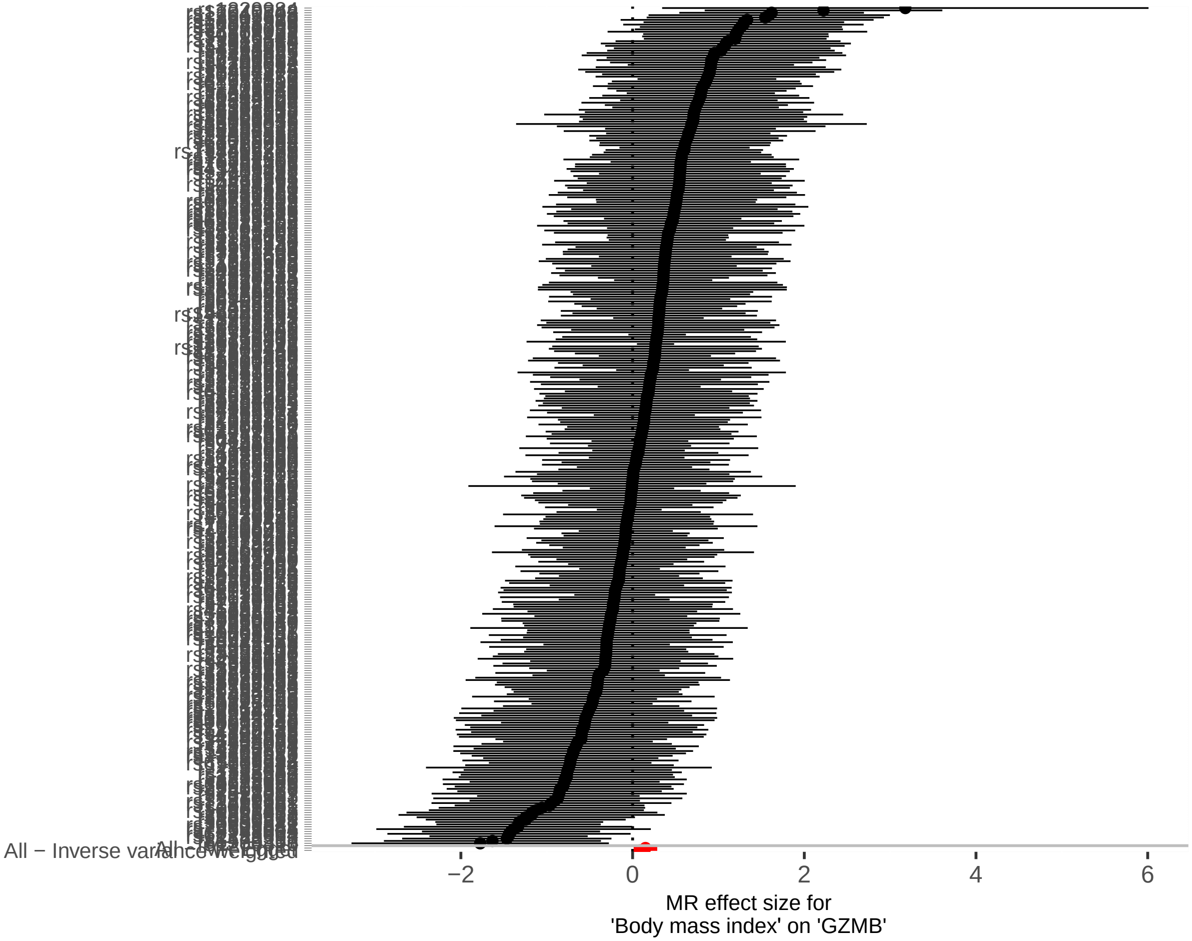
All − Inverse variance weighted
−2
0
2
4
6
MR effect size for
'Body mass index' on 'GZMB'

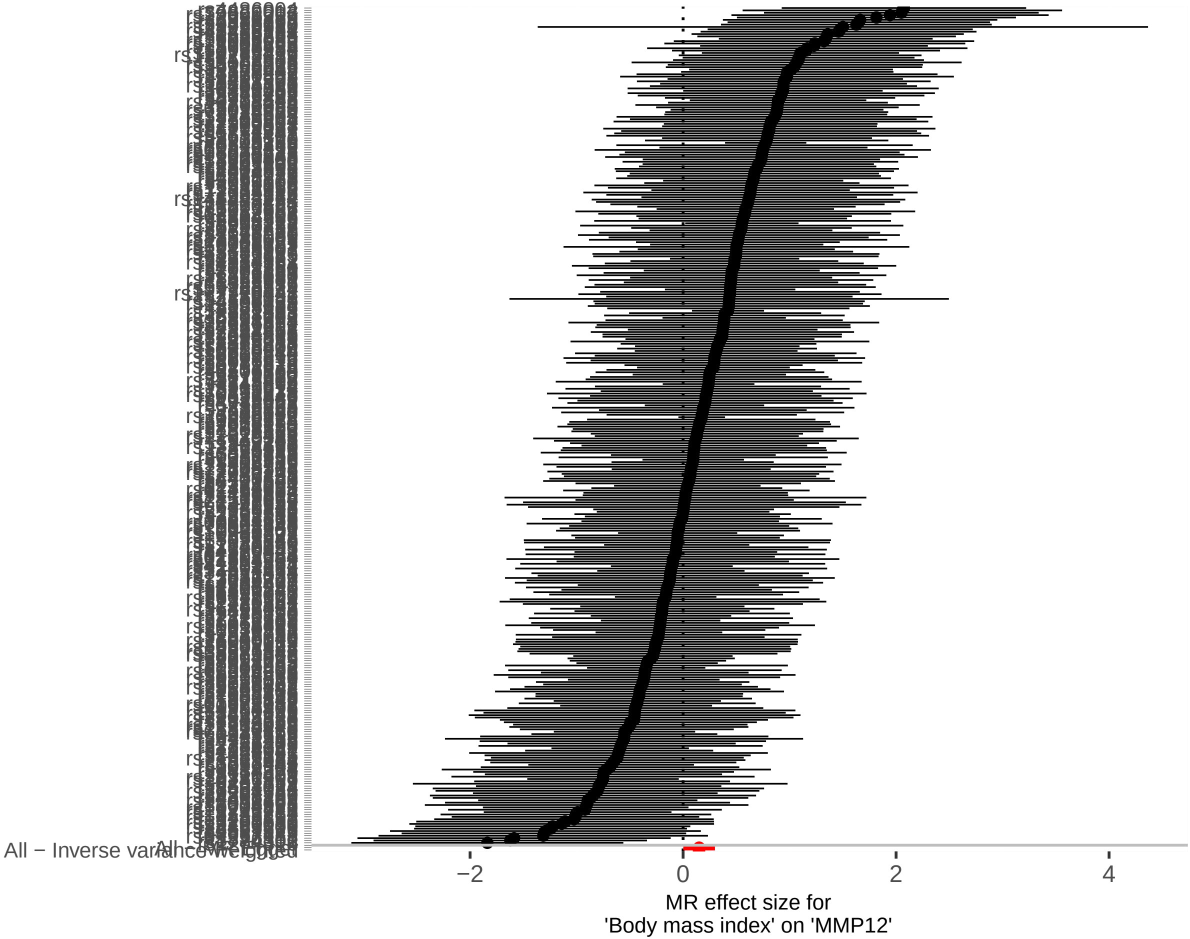
All − Inverse variance weighted
−2
0
2
4
MR effect size for
'Body mass index' on 'MMP12'

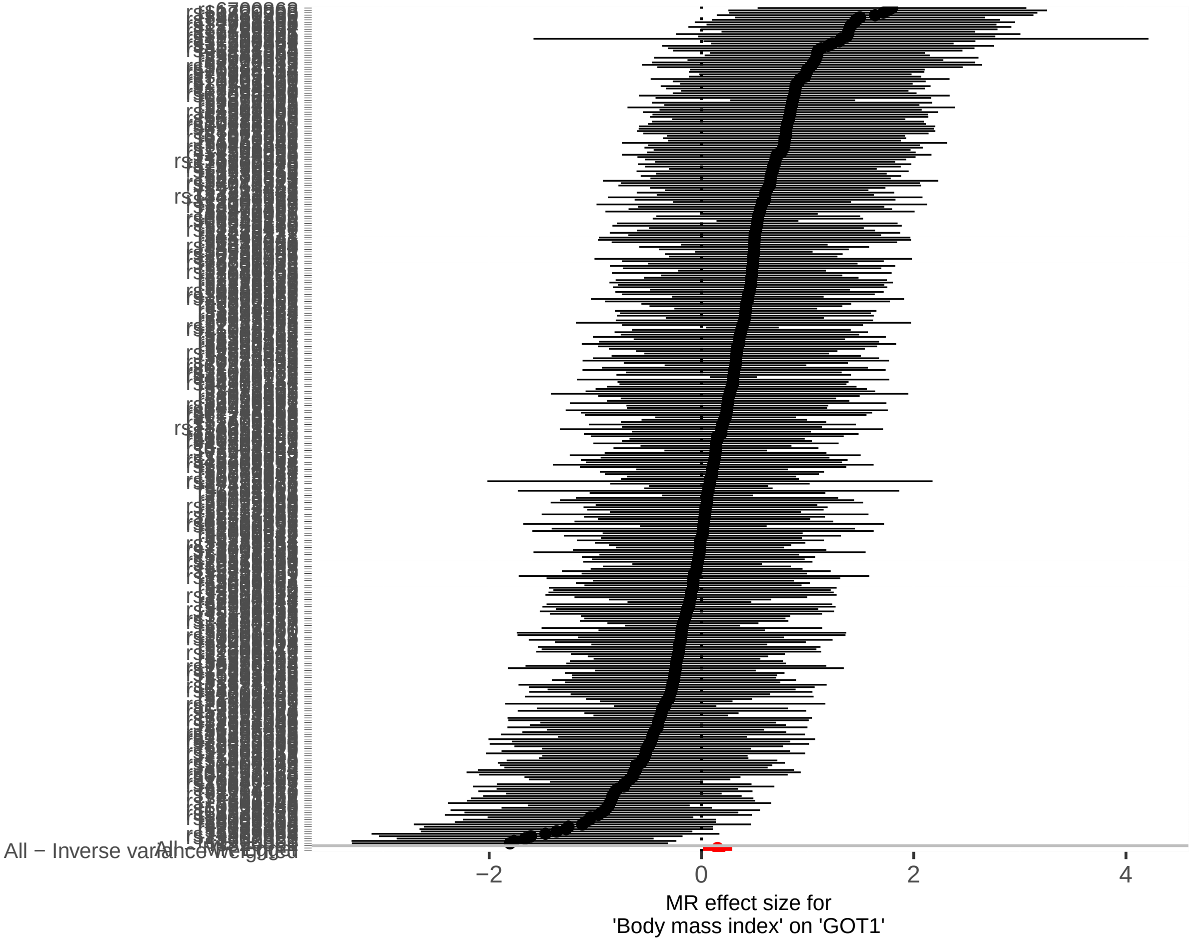

All − Inverse variance weighted
−2
0
2
4
MR effect size for
'Body mass index' on 'GOT1'

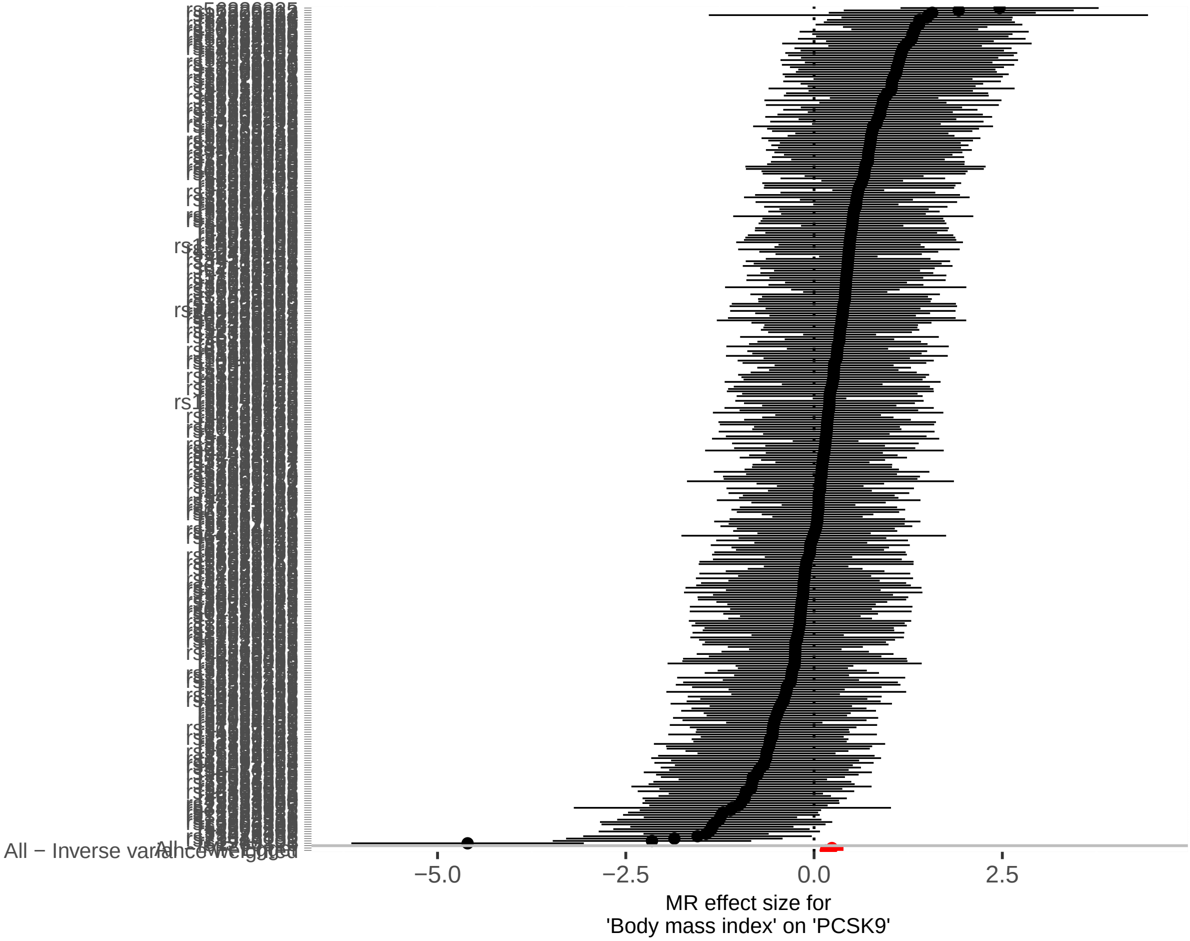

All − Inverse variance weighted
−5.0
−2.5
0.0
2.5
MR effect size for
'Body mass index' on 'PCSK9'

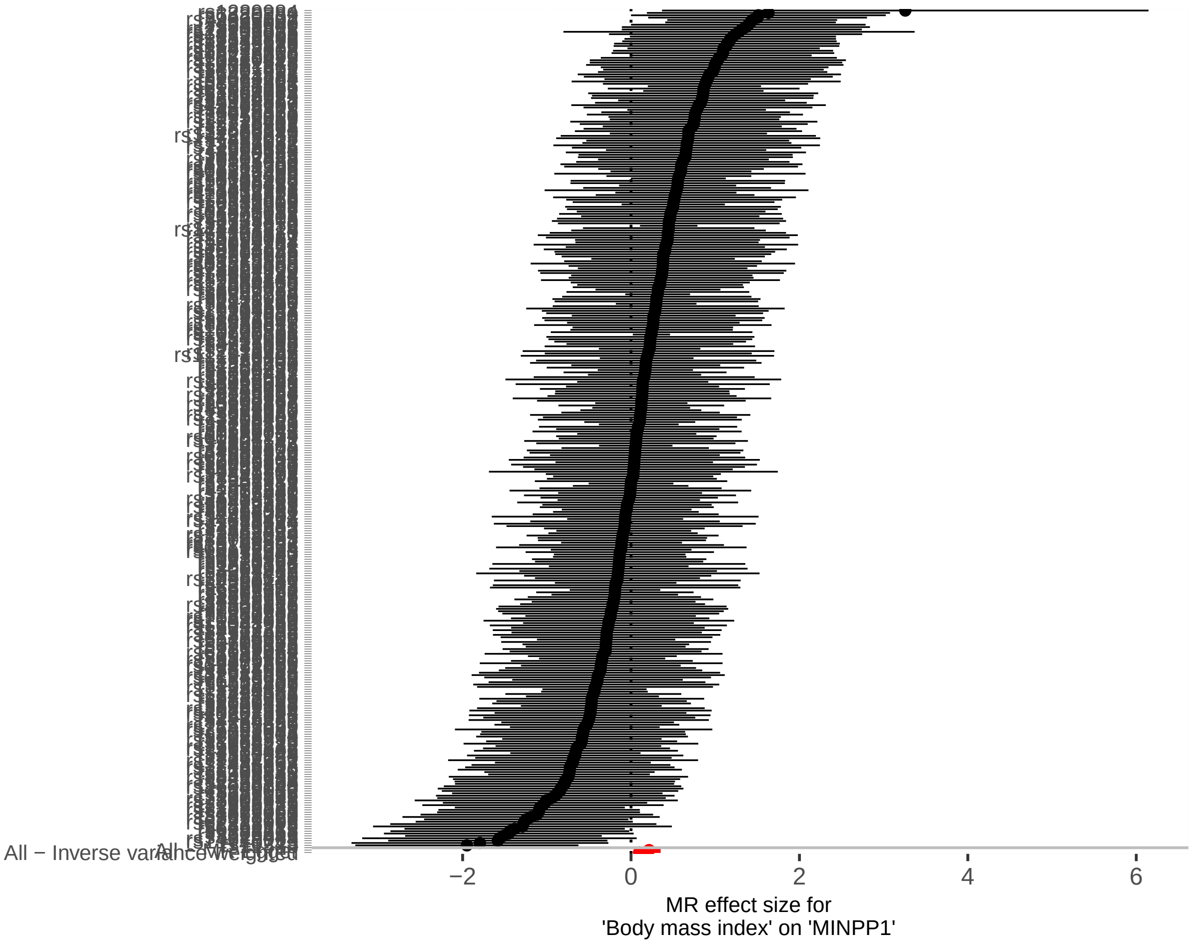

All − Inverse variance weighted
All − MR Egger
−2
0
2
4
6
MR effect size for
'Body mass index' on 'MINPP1'

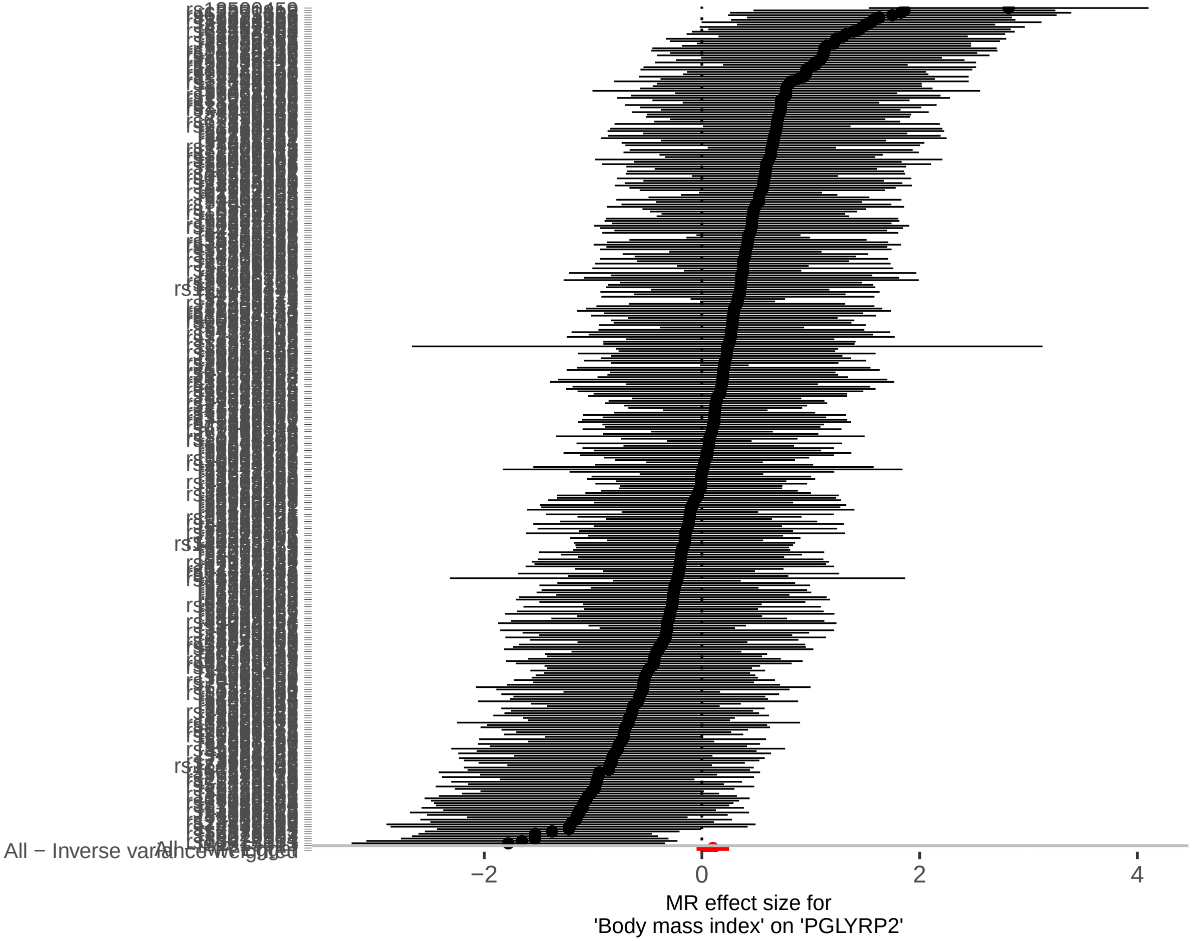

All − Inverse variance weighted
−2
0
2
4
MR effect size for
'Body mass index' on 'PGLYRP2'

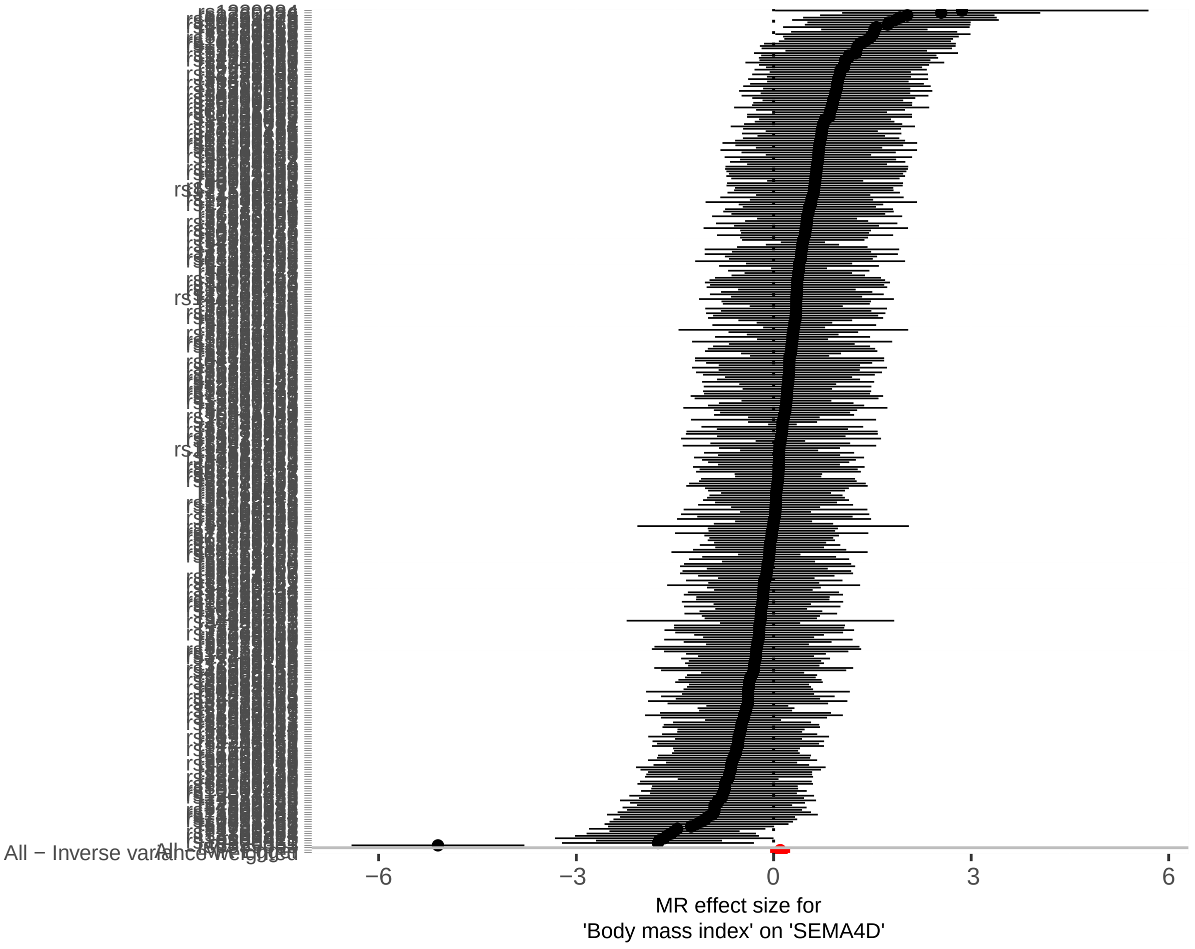
All − Inverse variance weighted
−6
−3
0
3
6
MR effect size for
'Body mass index' on 'SEMA4D'

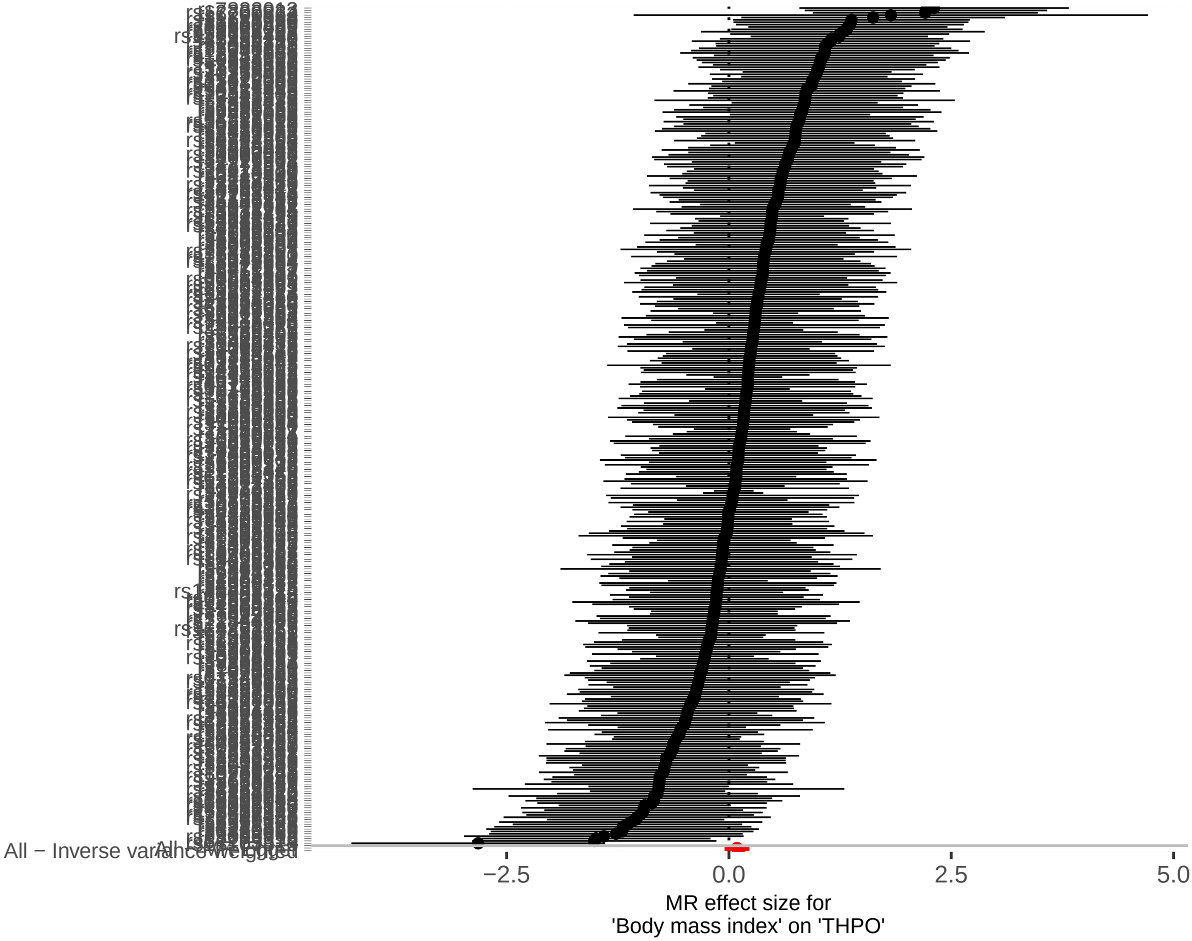
All − Inverse variance weighted
−2.5
0.0
2.5
5.0
MR effect size for
'Body mass index' on 'THPO'

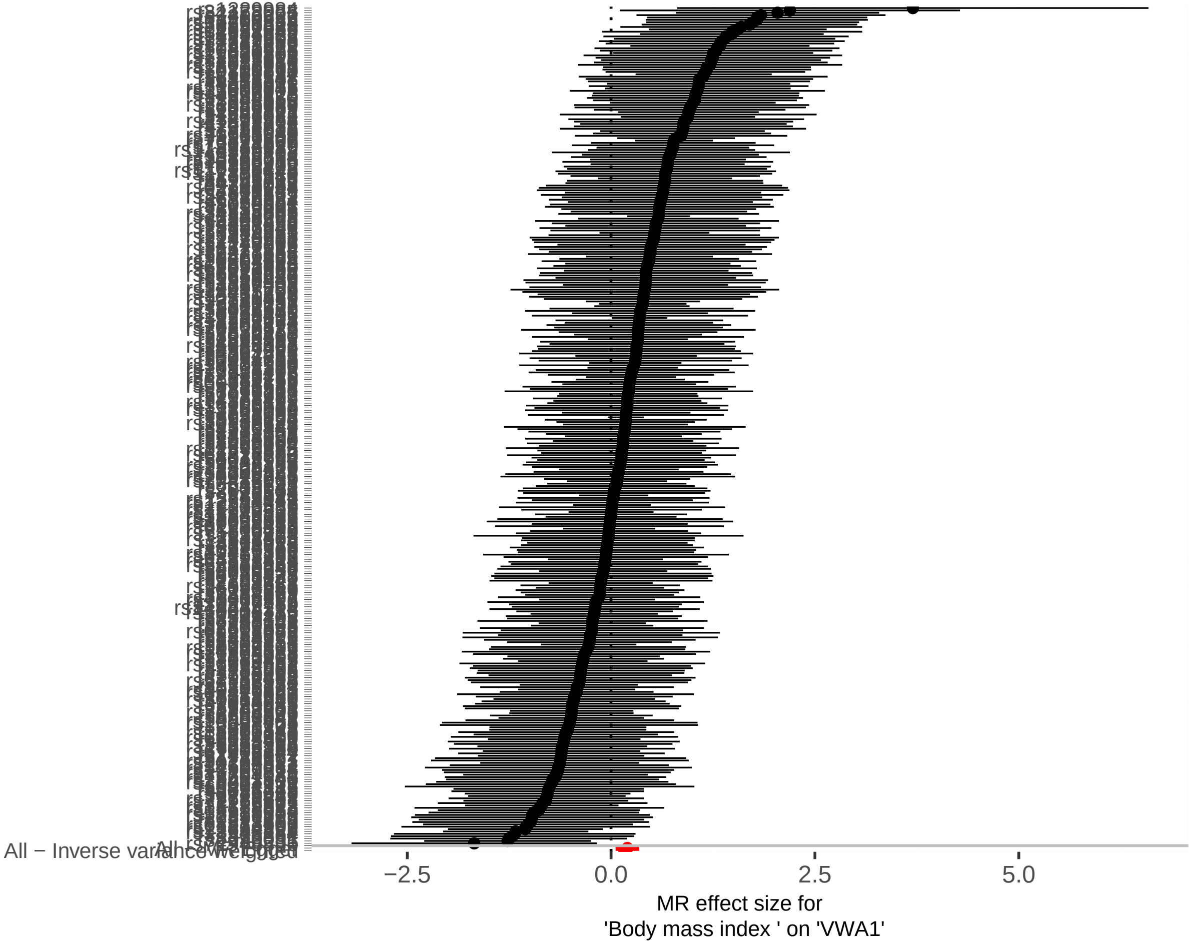

All − Inverse variance weighted
−2.5
0.0
2.5
5.0
MR effect size for
'Body mass index ' on 'VWA1'

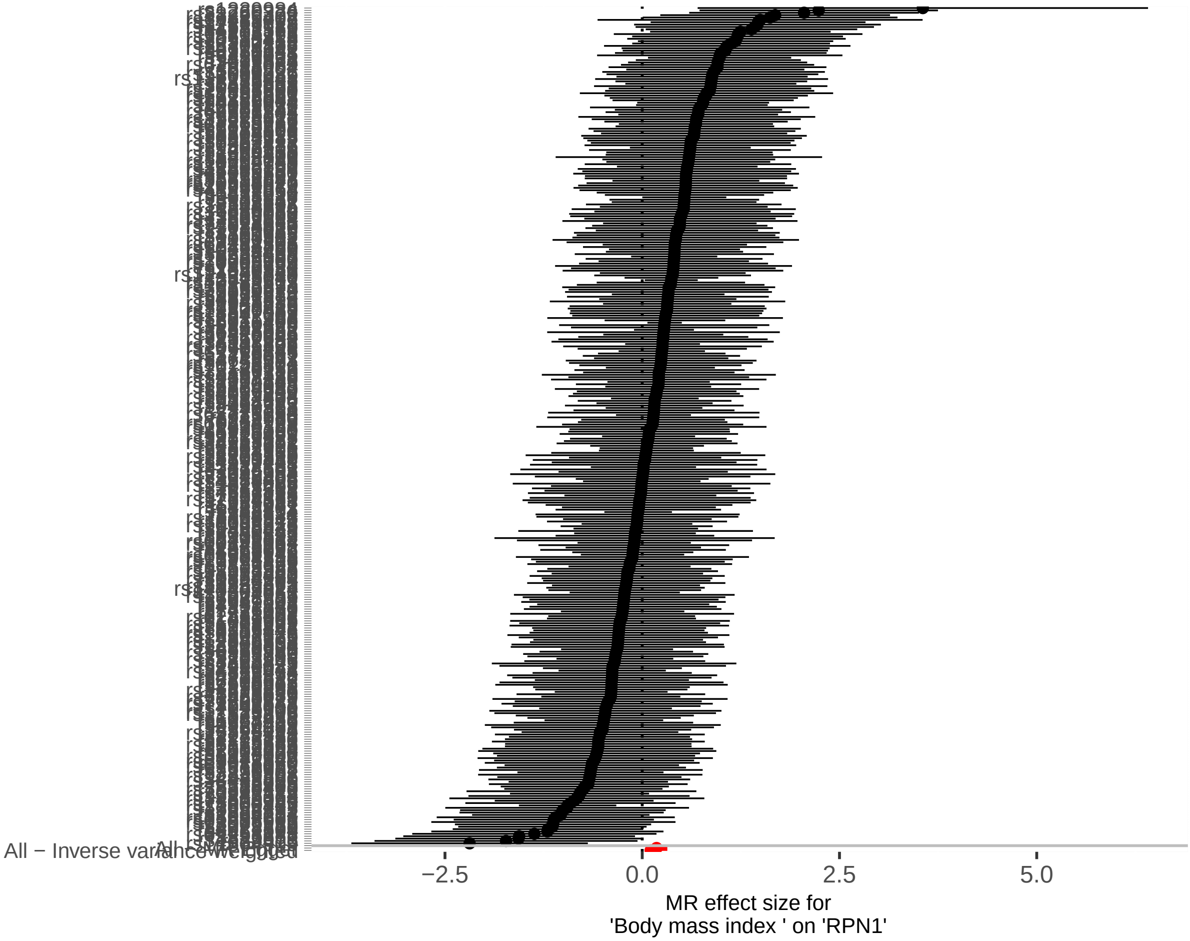

All − Inverse variance weighted
−2.5
0.0
2.5
5.0
MR effect size for
'Body mass index ' on 'RPN1'

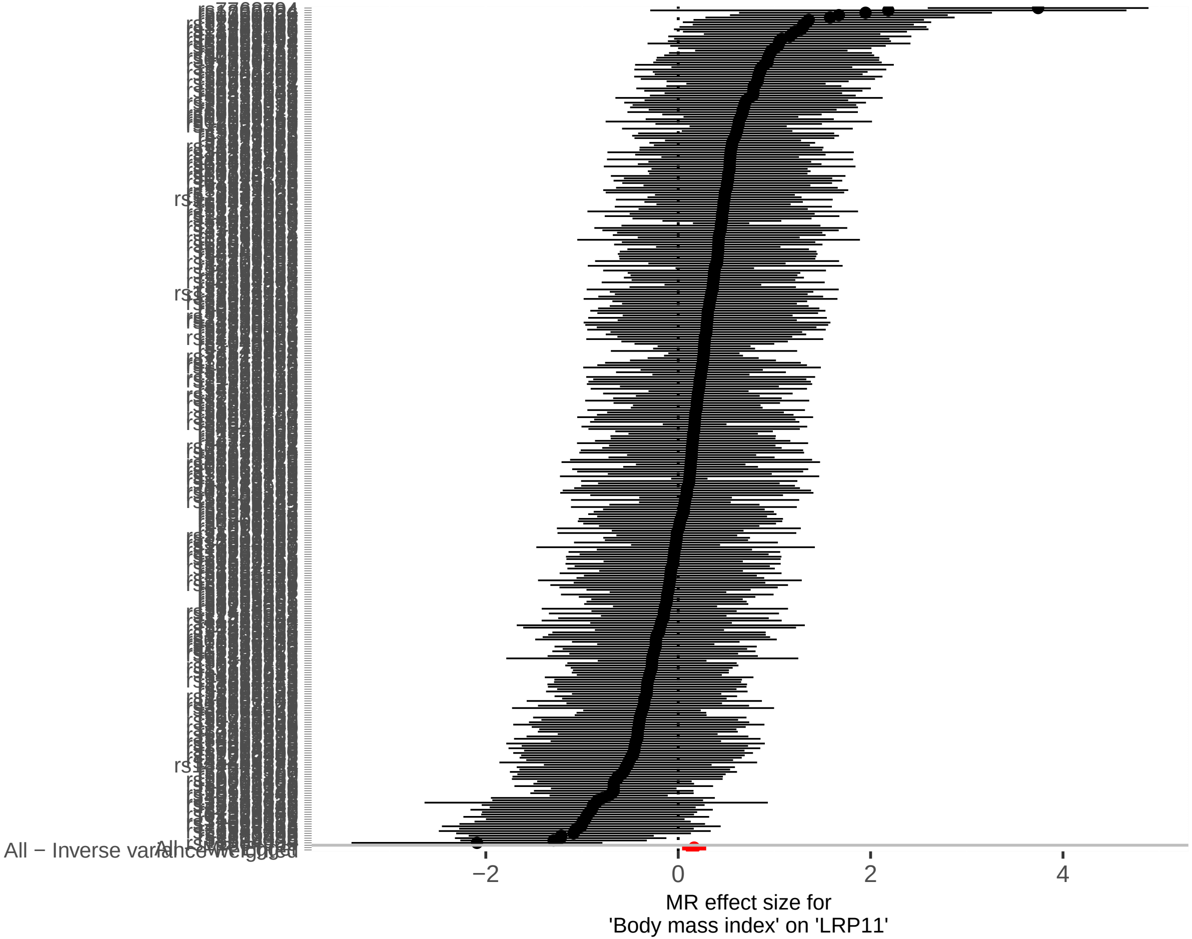
All − Inverse variance weighted
−2
0
2
4
MR effect size for
'Body mass index' on 'LRP11'

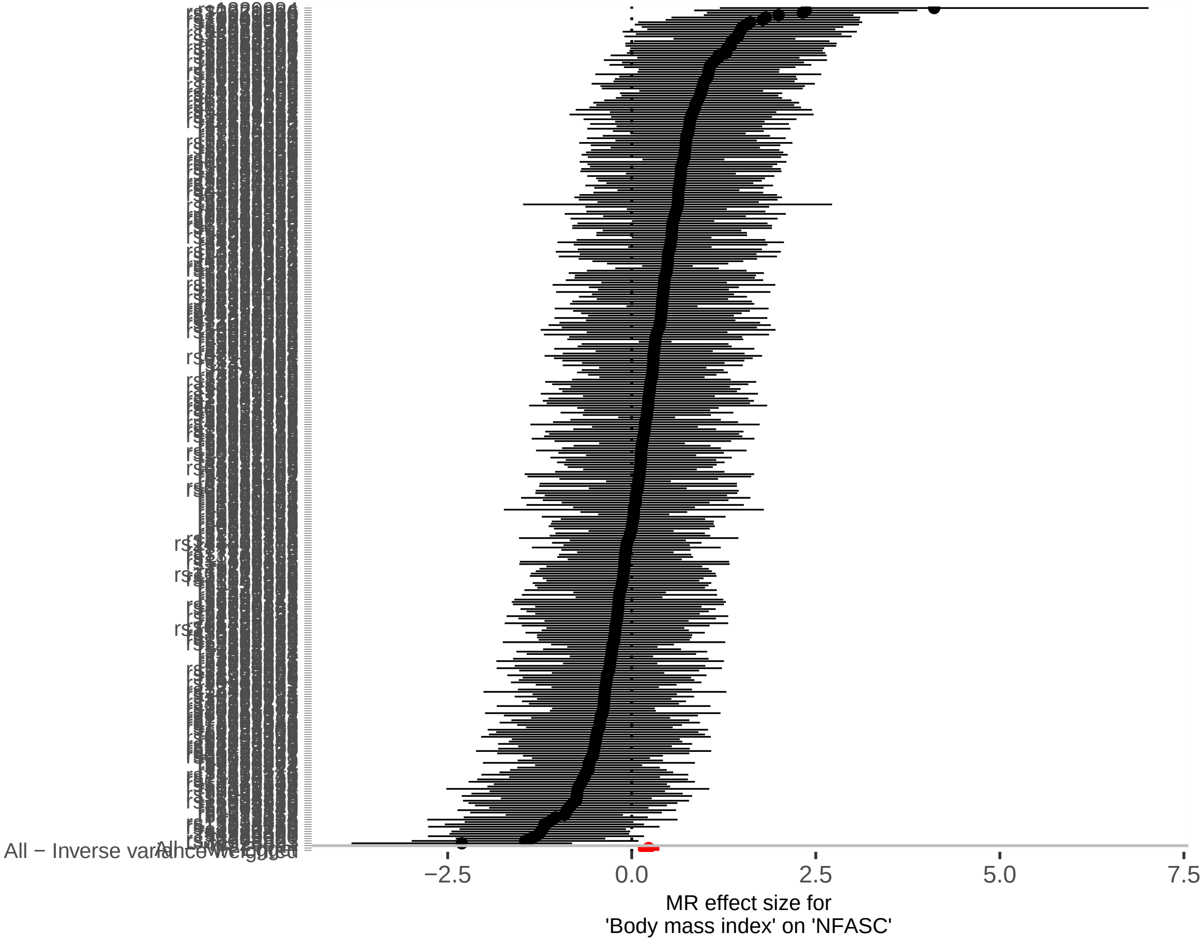

All − Inverse variance weighted
−2.5
0.0
2.5
5.0
7.5
MR effect size for
'Body mass index' on 'NFASC'

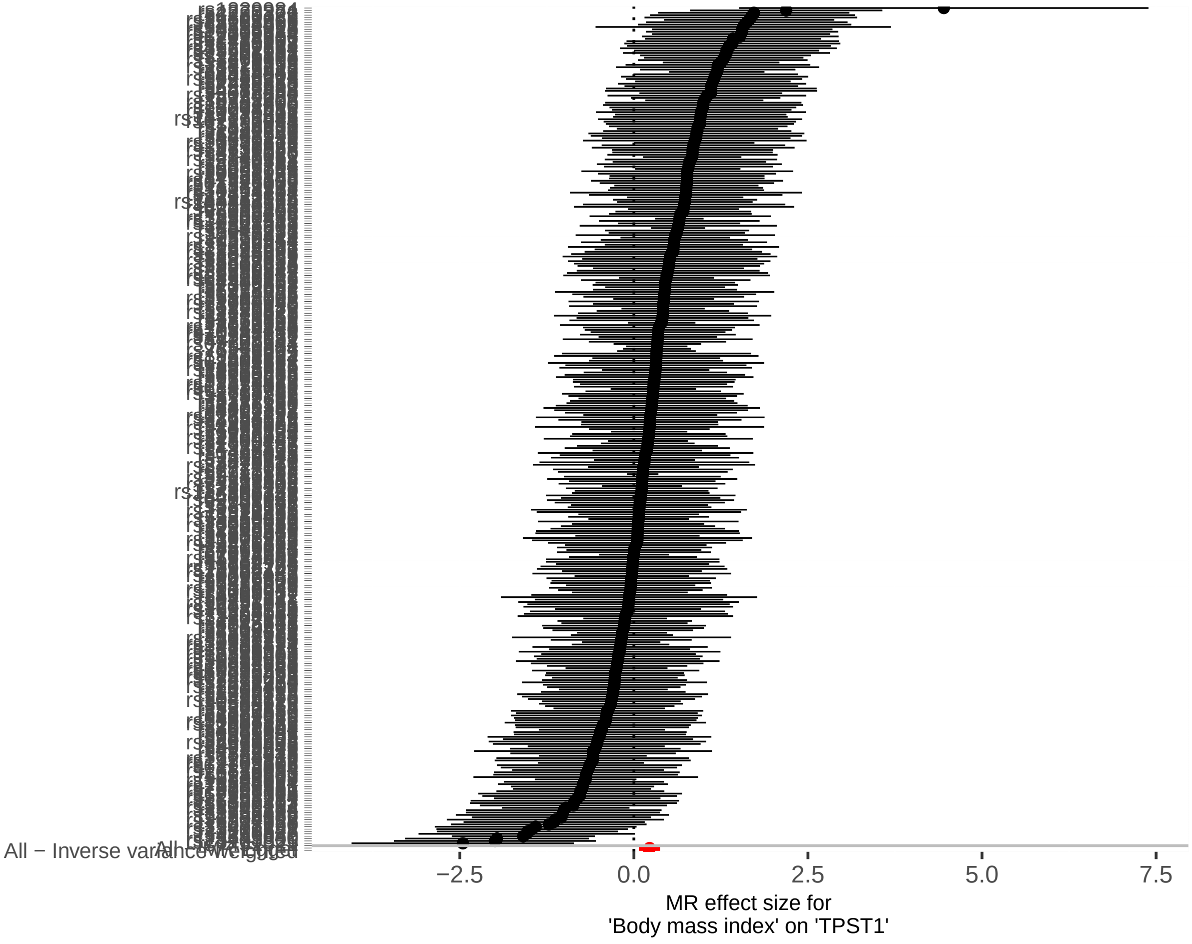

−2.5
0.0
2.5
5.0
7.5
MR effect size for
'Body mass index' on 'TPST1'

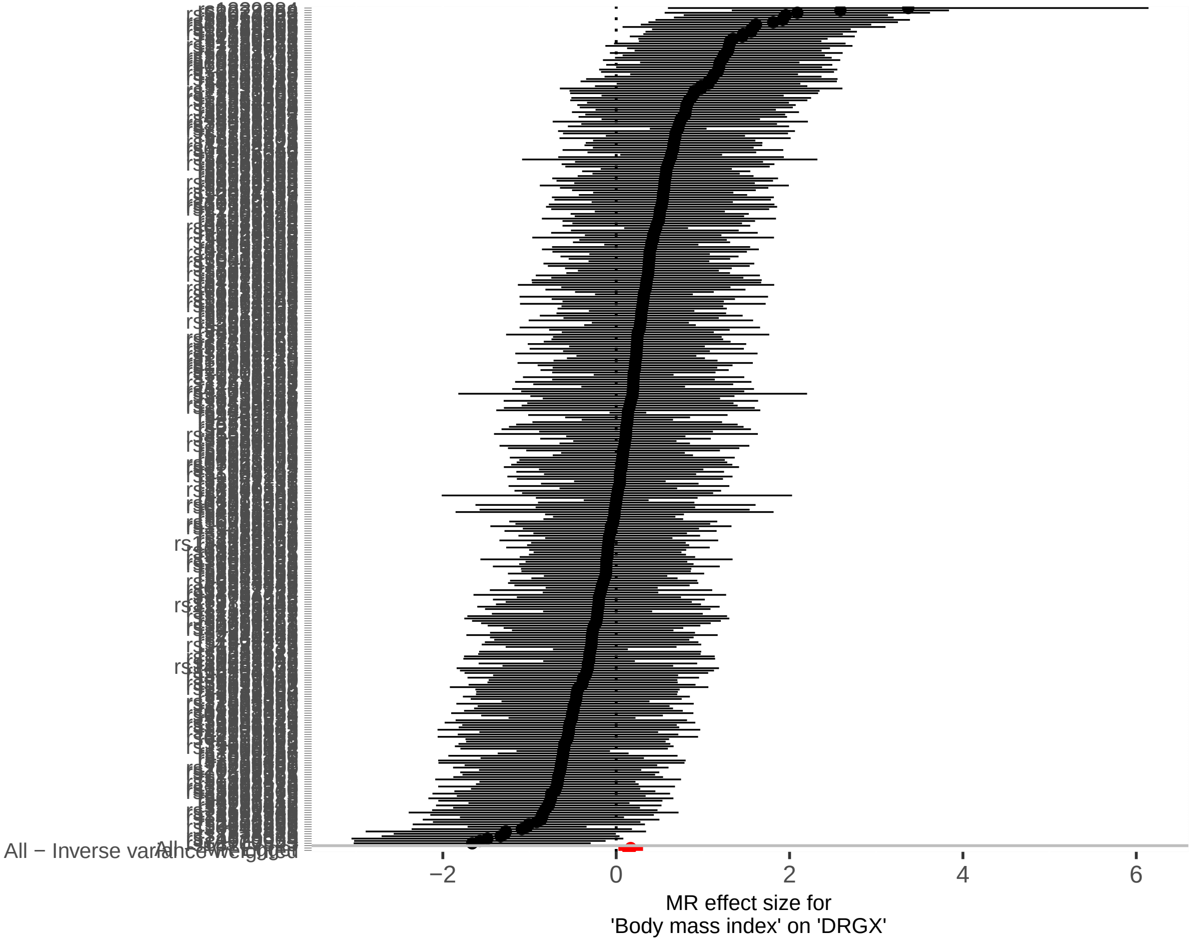

All − Inverse variance weighted
−2
0
2
4
6
MR effect size for
'Body mass index' on 'DRGX'

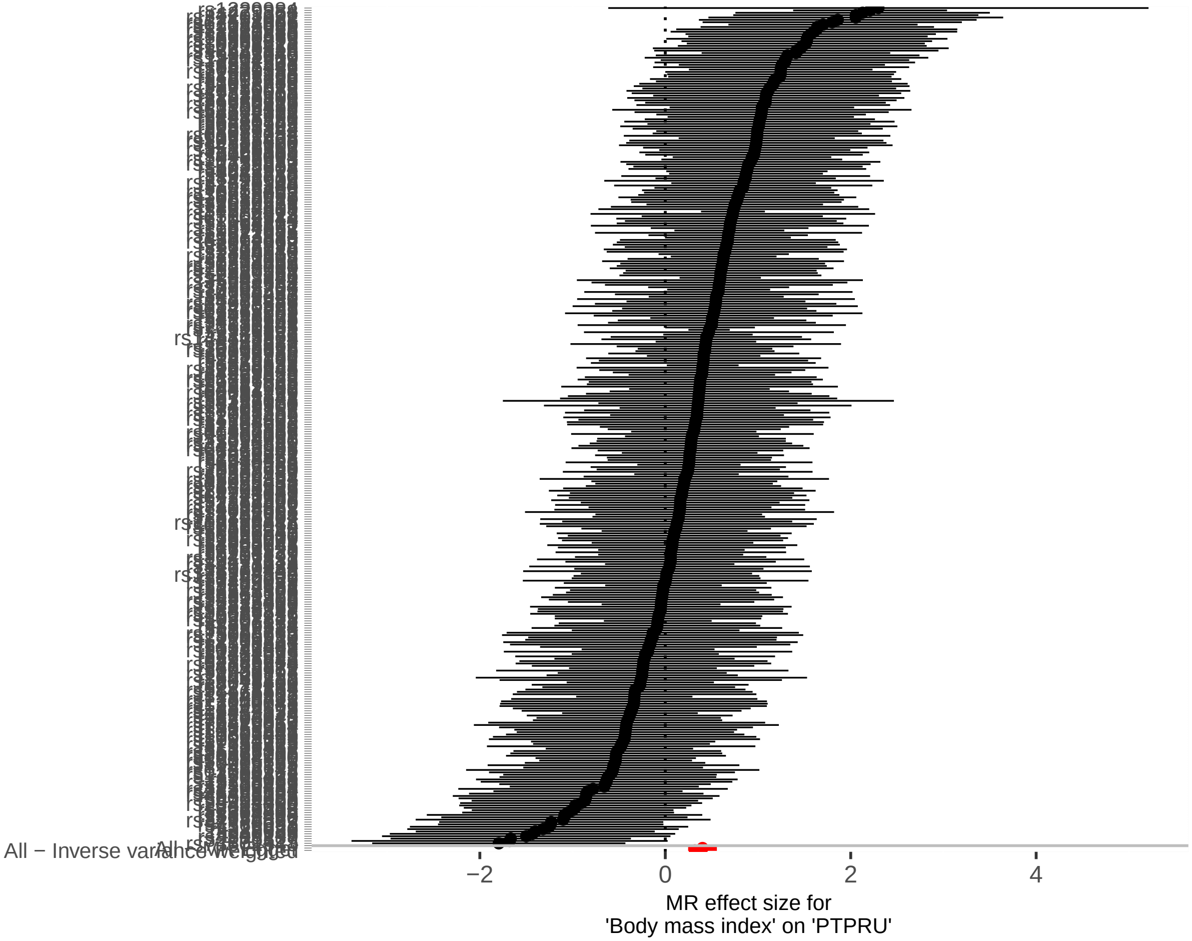
All − Inverse variance weighted
−2
0
2
4
MR effect size for
'Body mass index' on 'PTPRU'

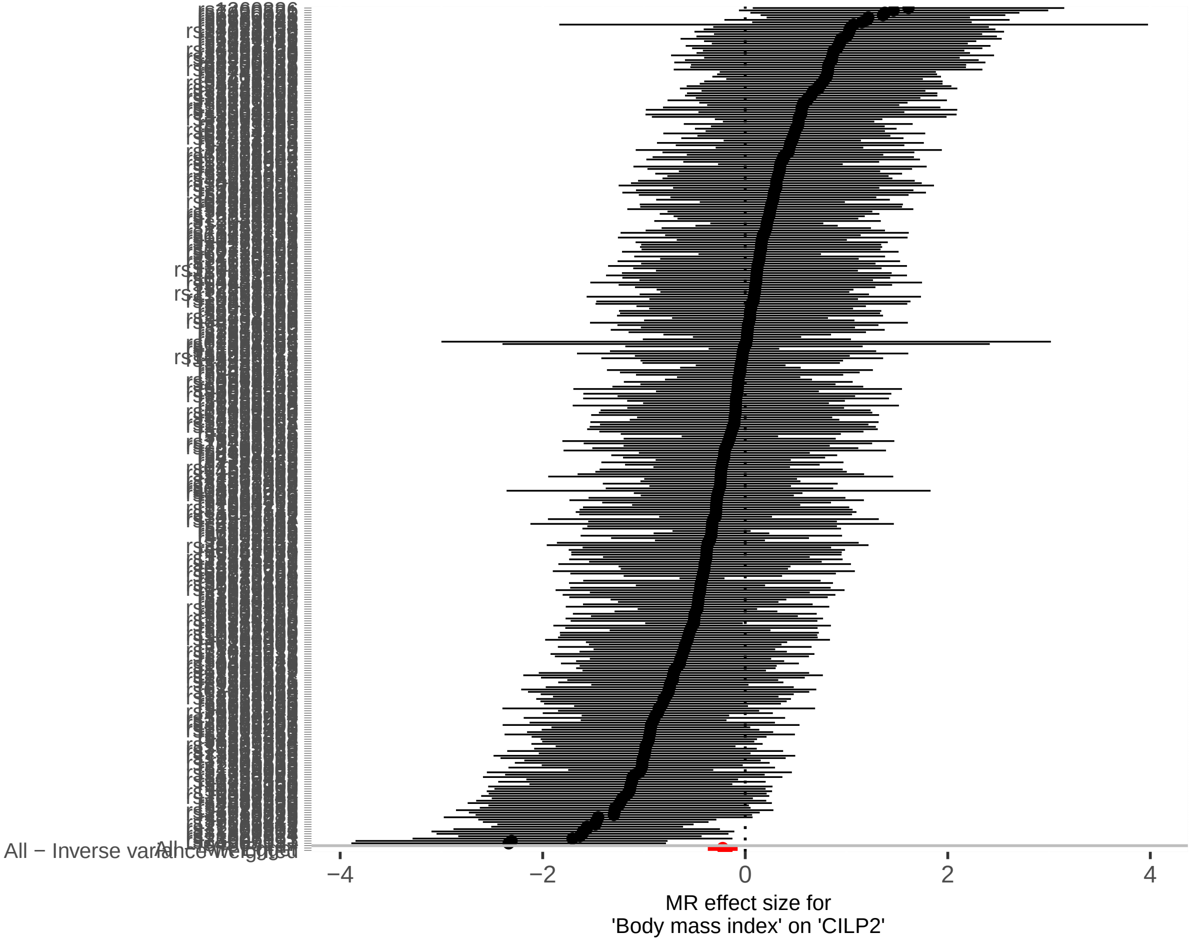
All − Inverse variance weighted
−4
−2
0
2
4
MR effect size for
'Body mass index' on 'CILP2'

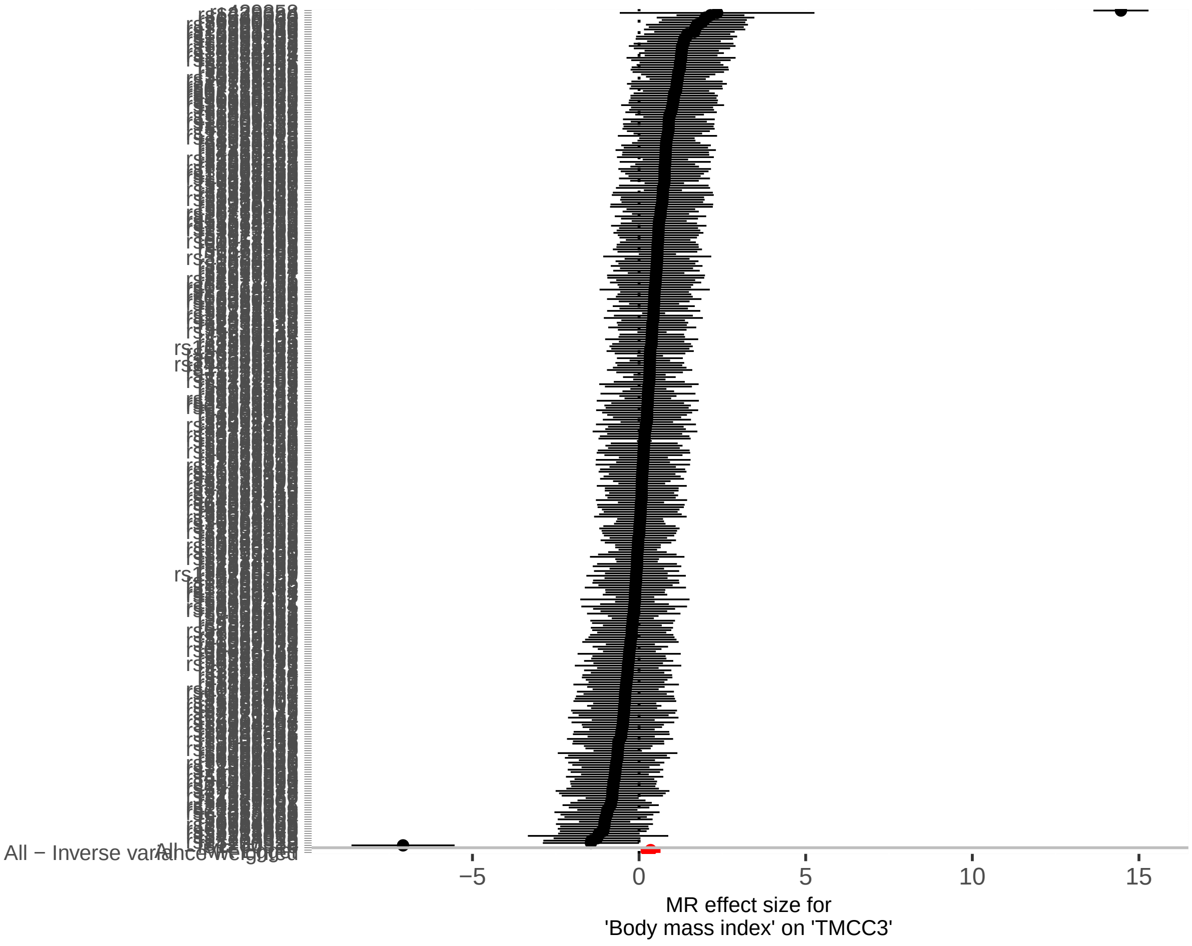

All − Inverse variance weighted
−5
0
5
10
15
MR effect size for
'Body mass index' on 'TMCC3'

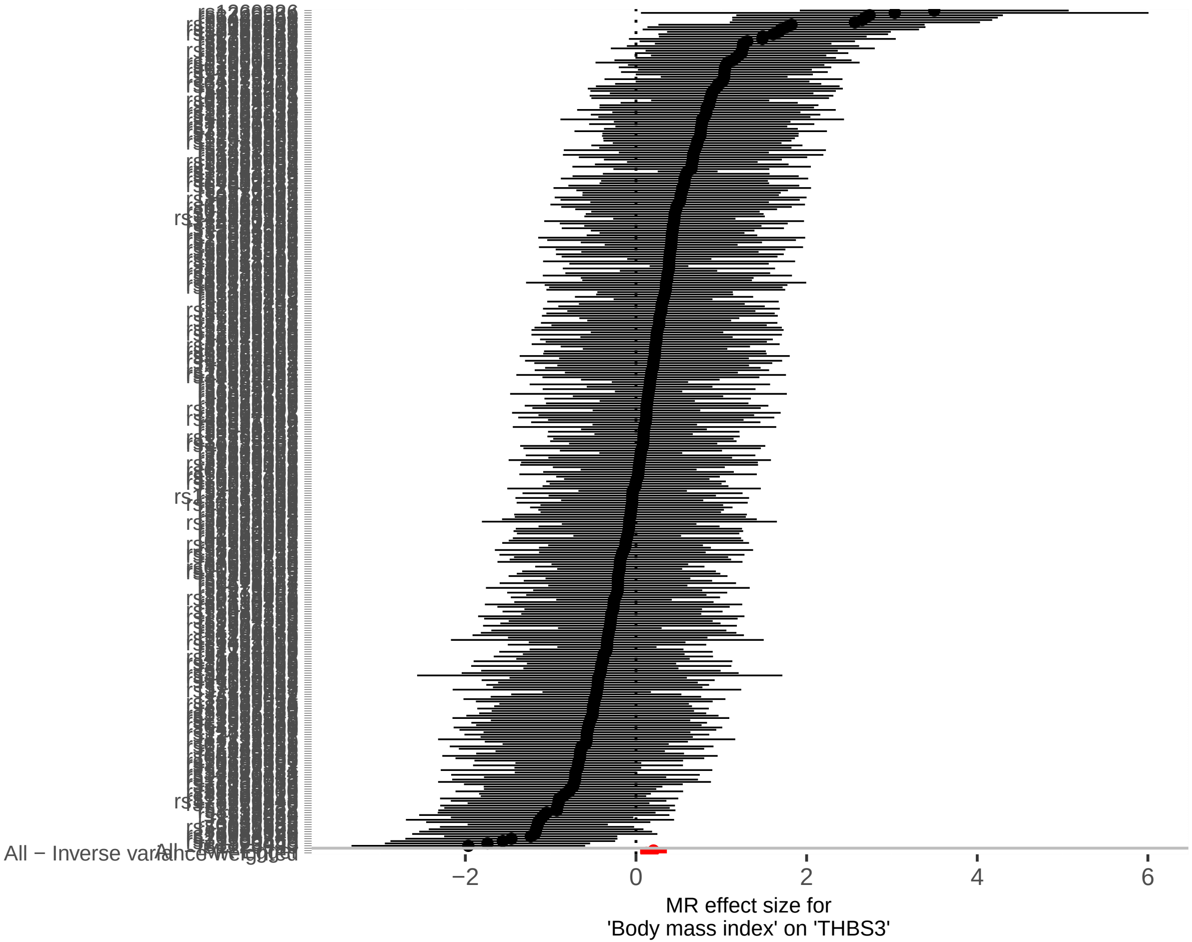

All − Inverse variance weighted
−2
0
2
4
6
MR effect size for
'Body mass index' on 'THBS3'

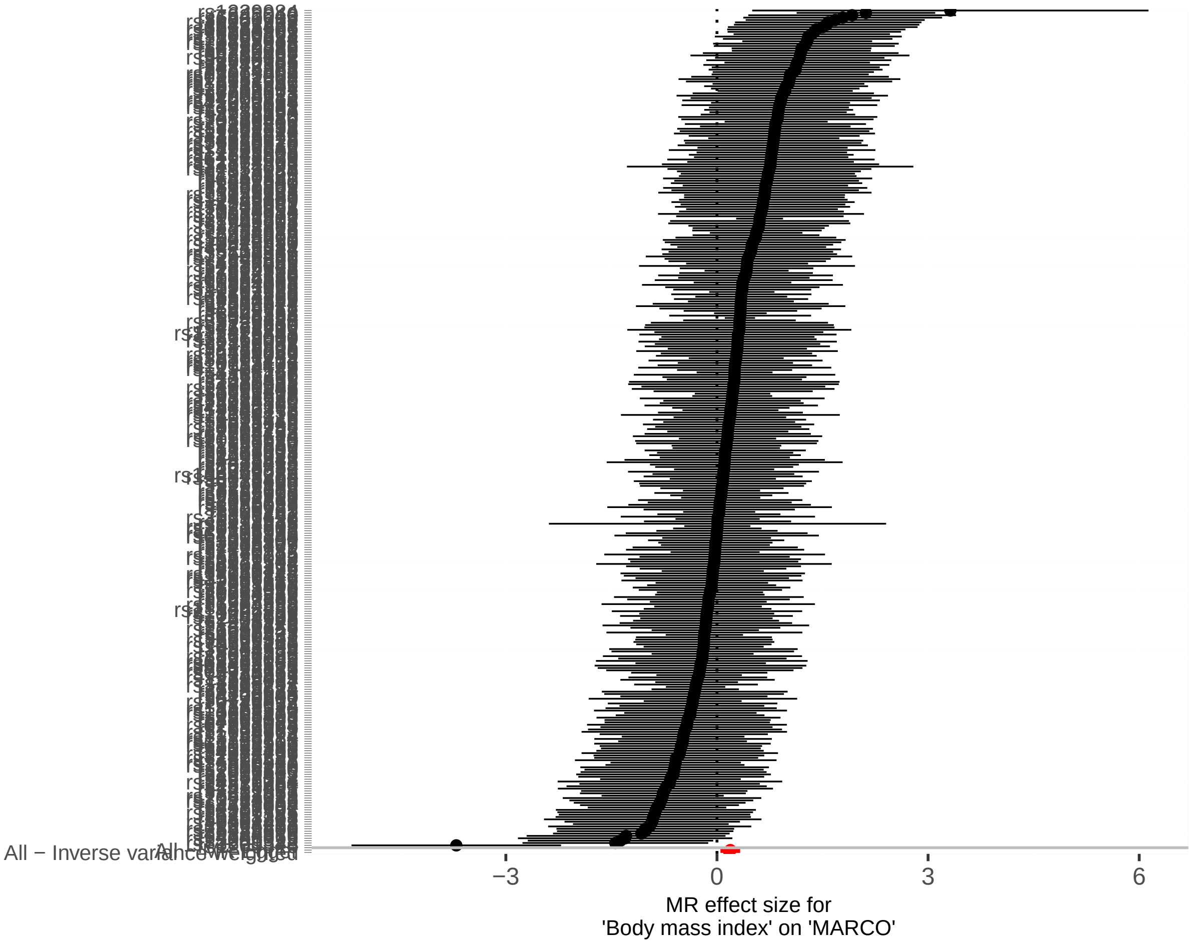
All − Inverse variance weighted
−3
0
3
6
MR effect size for
'Body mass index' on 'MARCO'

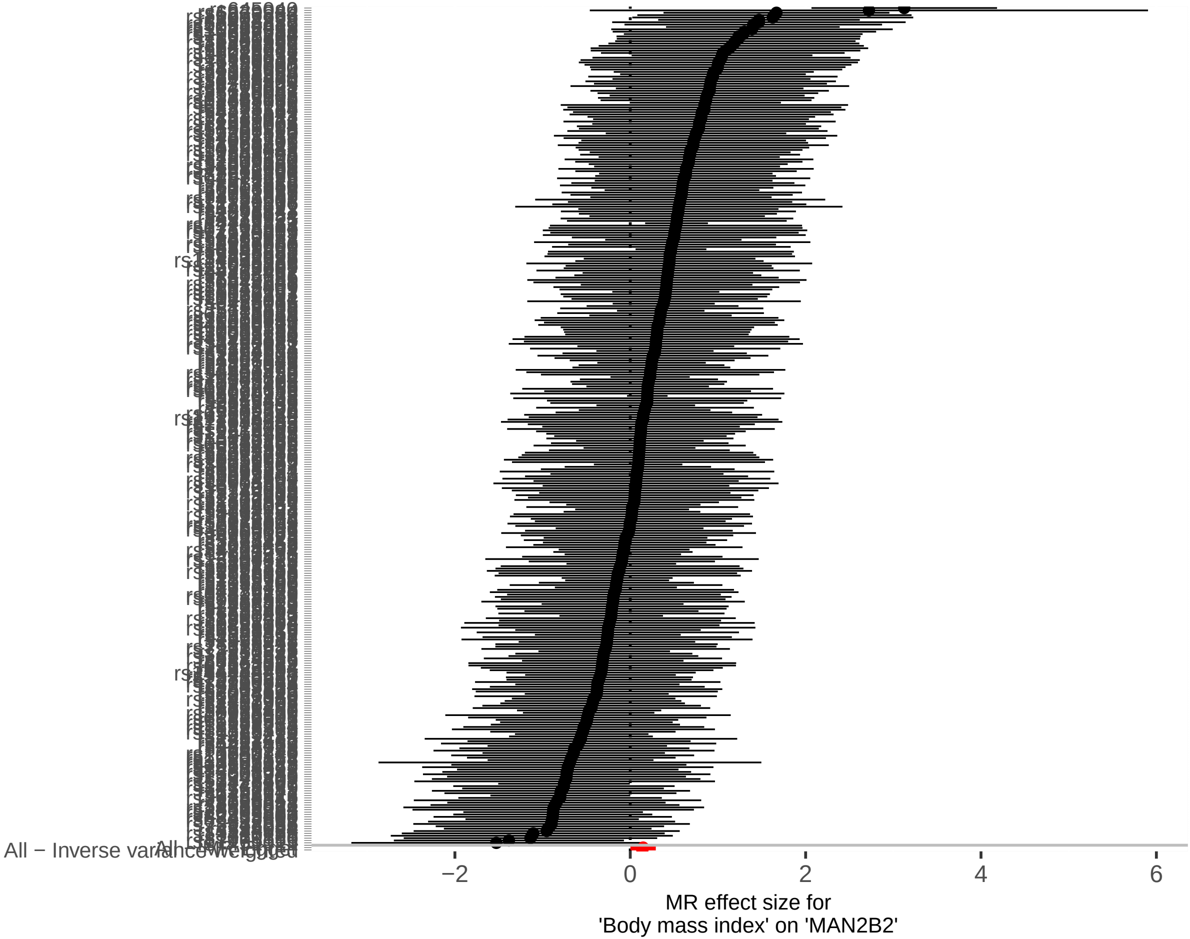

All − Inverse variance weighted
−2
0
2
4
6
MR effect size for
'Body mass index' on 'MAN2B2'

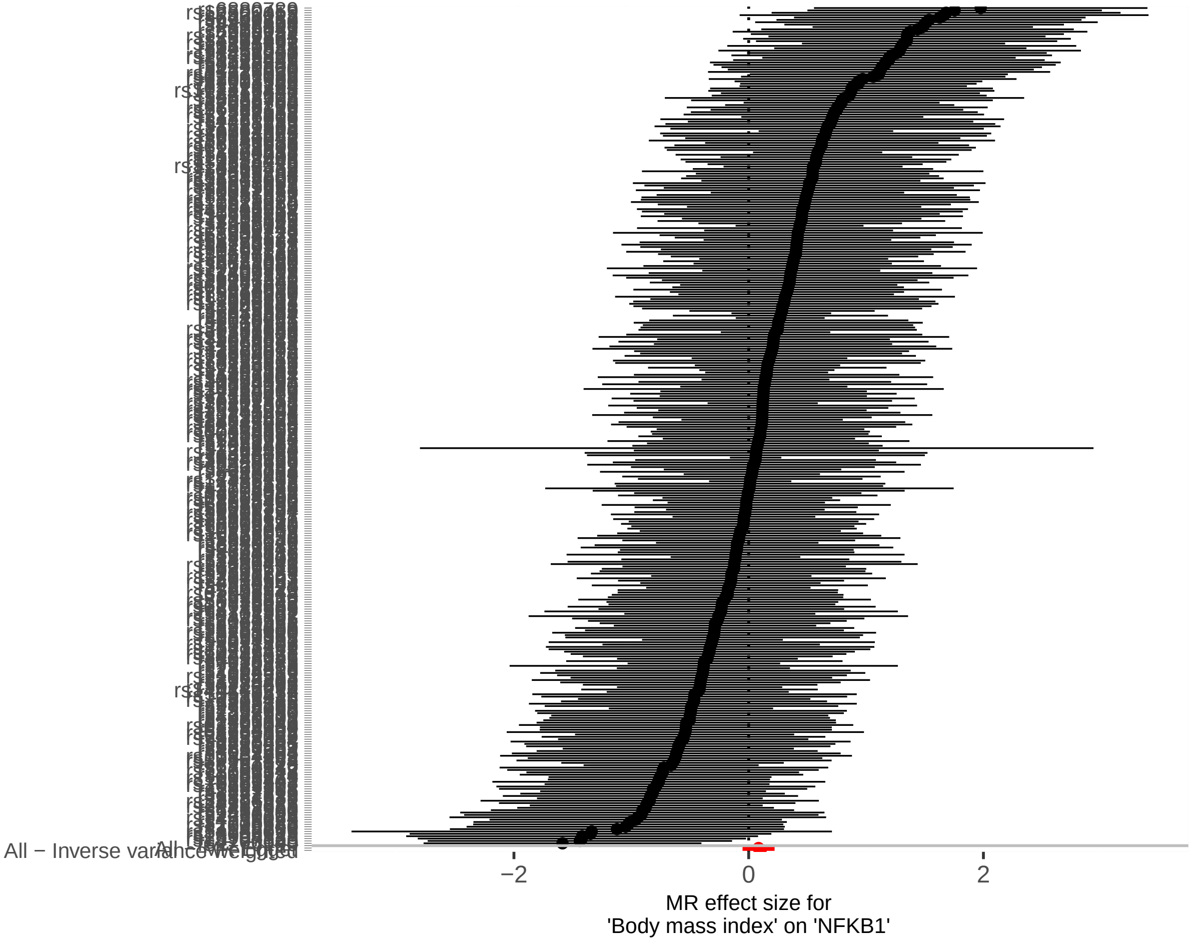

All − Inverse variance weighted
−2
0
2
MR effect size for
'Body mass index' on 'NFKB1'

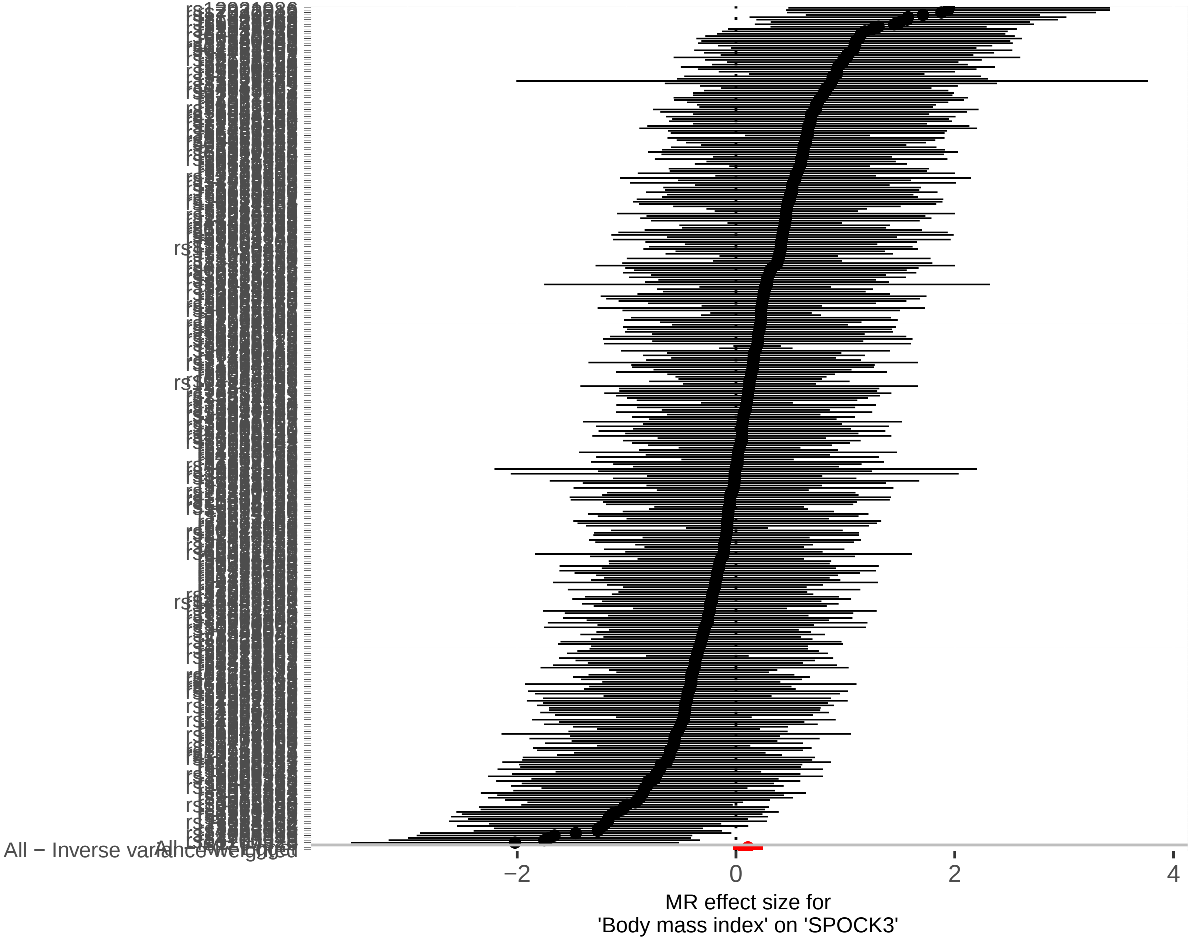
All − Inverse variance weighted
−2
0
2
4
MR effect size for
'Body mass index' on 'SPOCK3'
