# Supplementary material for: Mediating Mendelian randomization in the proteome identified potential drug targets for obesity-related allergic asthma
Source: Hereditas. 2025 Feb 1;162:12. doi: 10.1186/s41065-025-00376-w (PMC11786417; doi:10.1186/s41065-025-00376-w)
Supplement: Supplementary file 1 — Supplementary Material 1: Supplementary Materials 1. The MR results of BMI and allergic asthma. Supplementary Materials 2. The MR results of plasma proteins and allergic asthma. Supplementary Materials 3. The MR results of BMI and plasma proteins. Supplementary Materials 4. The MR results of mediation factor plasma proteins and allergic asthma. Supplementary Materials 5. The results of drug-targeted MR [file 41065_2025_376_MOESM1_ESM.zip › Supplementary Materials/S3 The MR results of BMI and plasma proteins/S3 BMI-plasma proteins.scatter_plot.pdf]

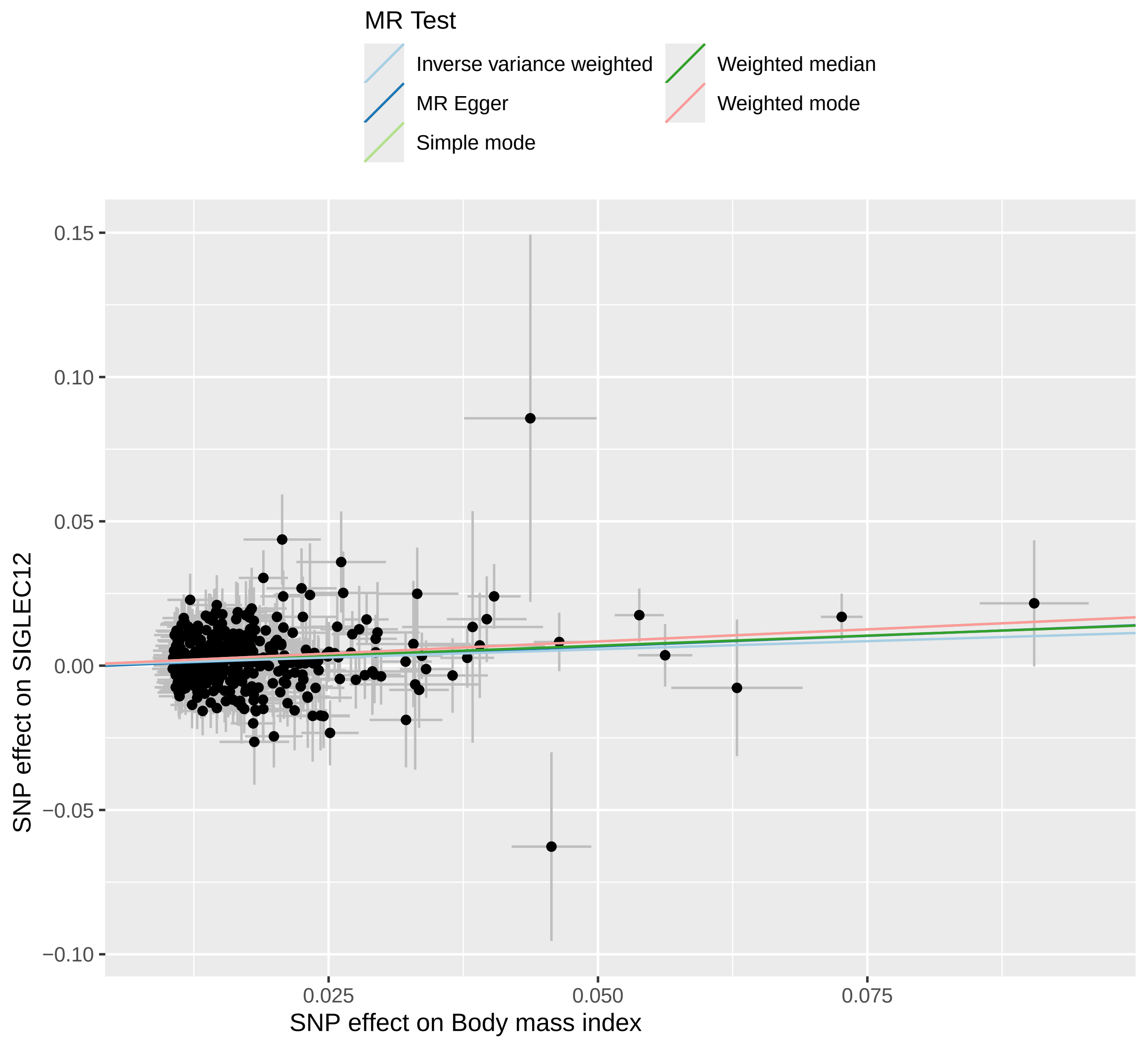
MR Test
Inverse variance weighted
MR Egger
Simple mode
Weighted median
Weighted mode
0.15
0.10
0.05
0.00
−0.05
−0.10
SNP effect on SIGLEC12
0.025
0.050
0.075
SNP effect on Body mass index

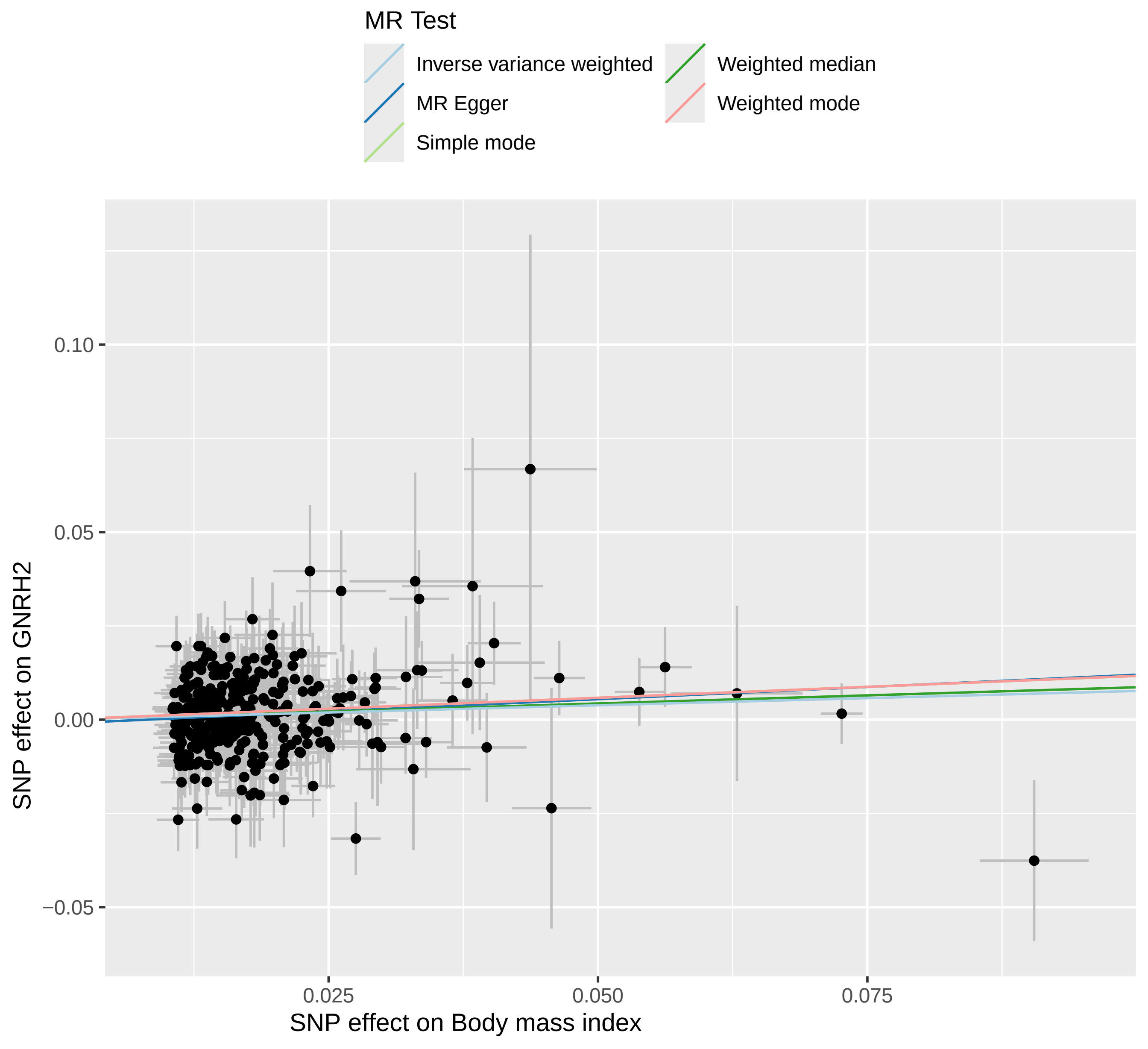
MR Test
Inverse variance weighted
MR Egger
Simple mode
Weighted median
Weighted mode
0.10
0.05
0.00
−0.05
0.025
0.050
0.075
SNP effect on GNRH2
SNP effect on Body mass index

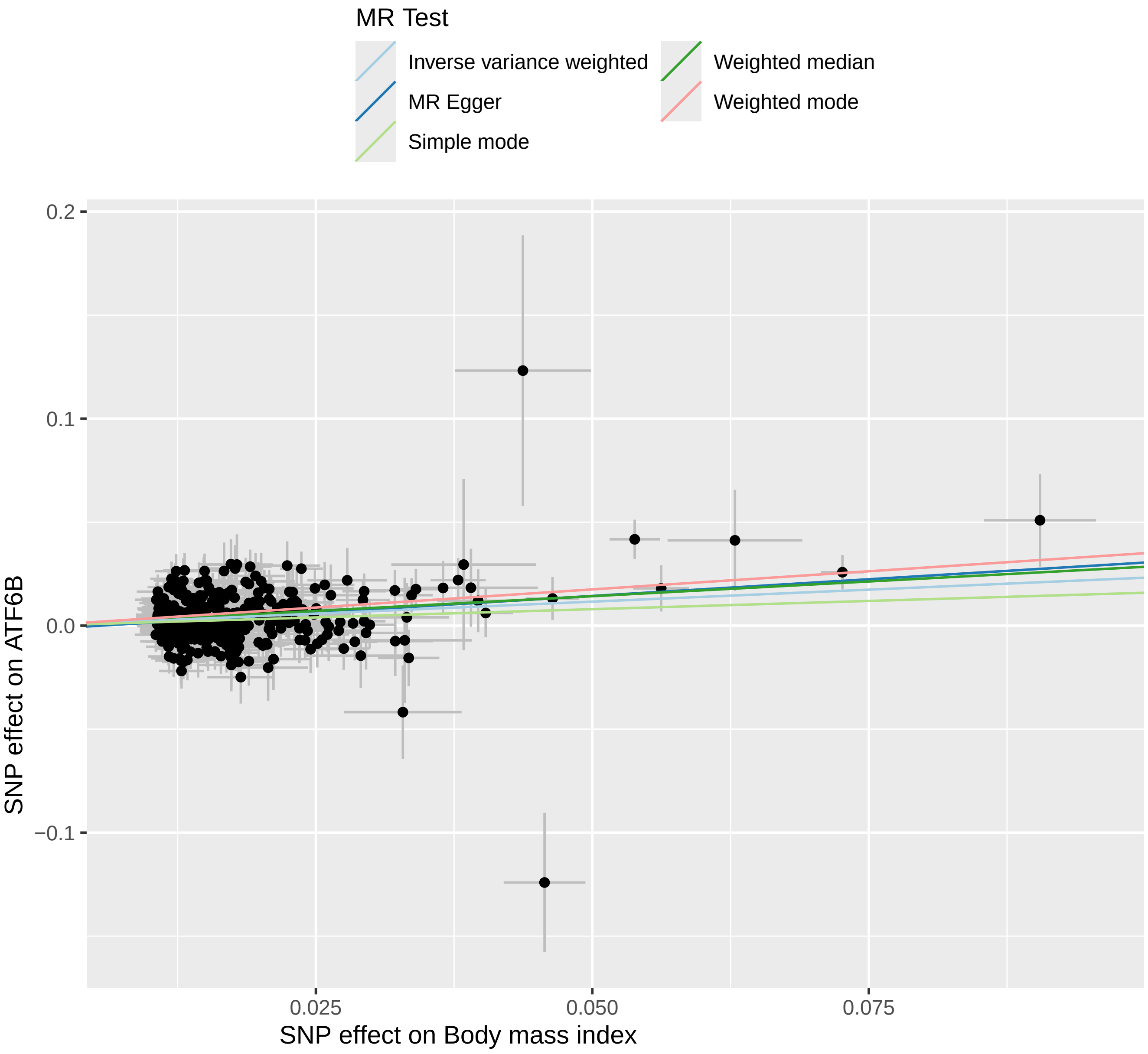
MR Test
Inverse variance weighted
MR Egger
Simple mode
Weighted median
Weighted mode
0.2
0.1
0.0
−0.1
0.025
0.050
0.075
SNP effect on ATF6B
SNP effect on Body mass index

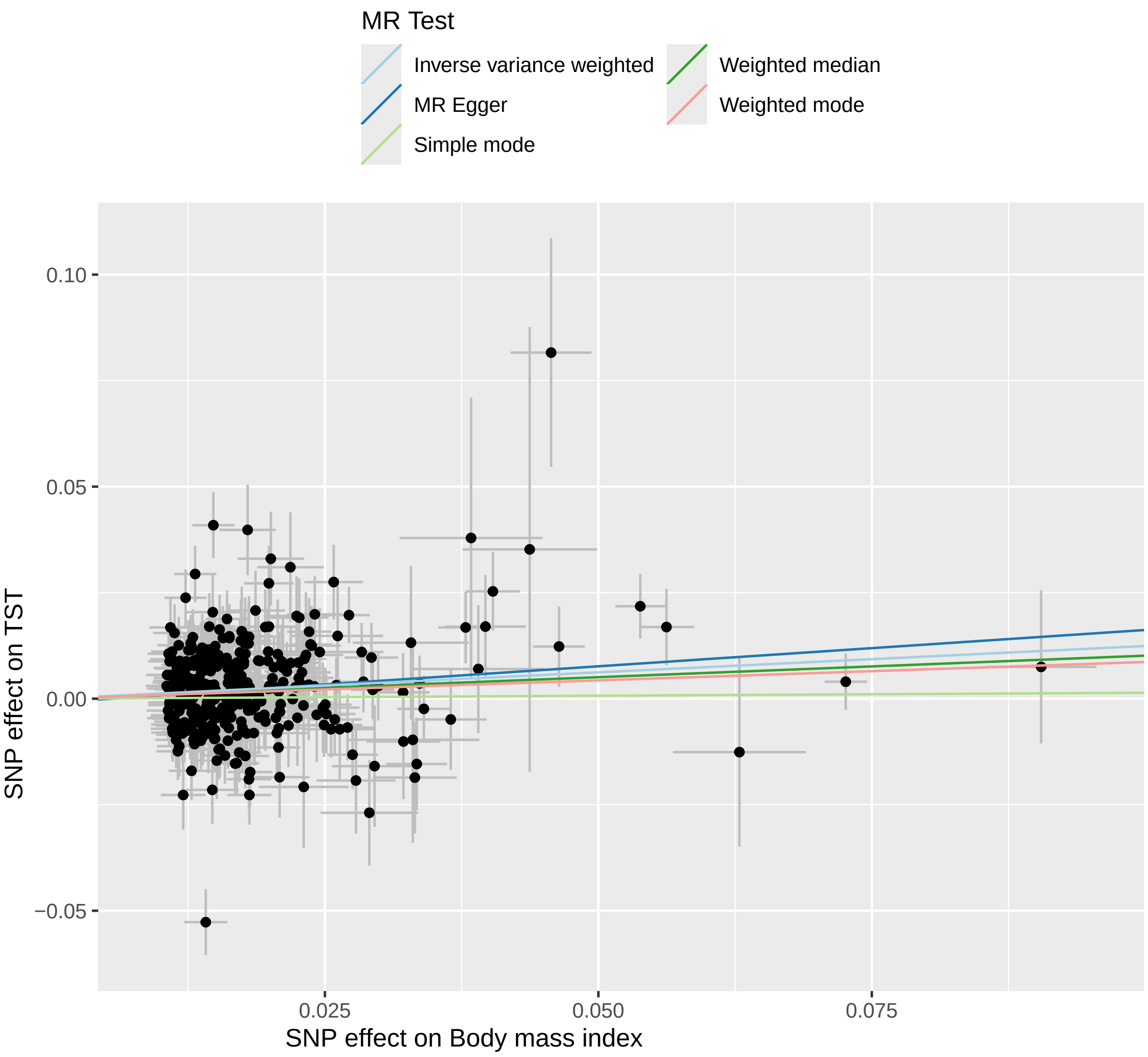
MR Test
Inverse variance weighted
MR Egger
Simple mode
Weighted median
Weighted mode
0.10
0.05
0.00
−0.05
SNP effect on TST
0.025
0.050
0.075
SNP effect on Body mass index

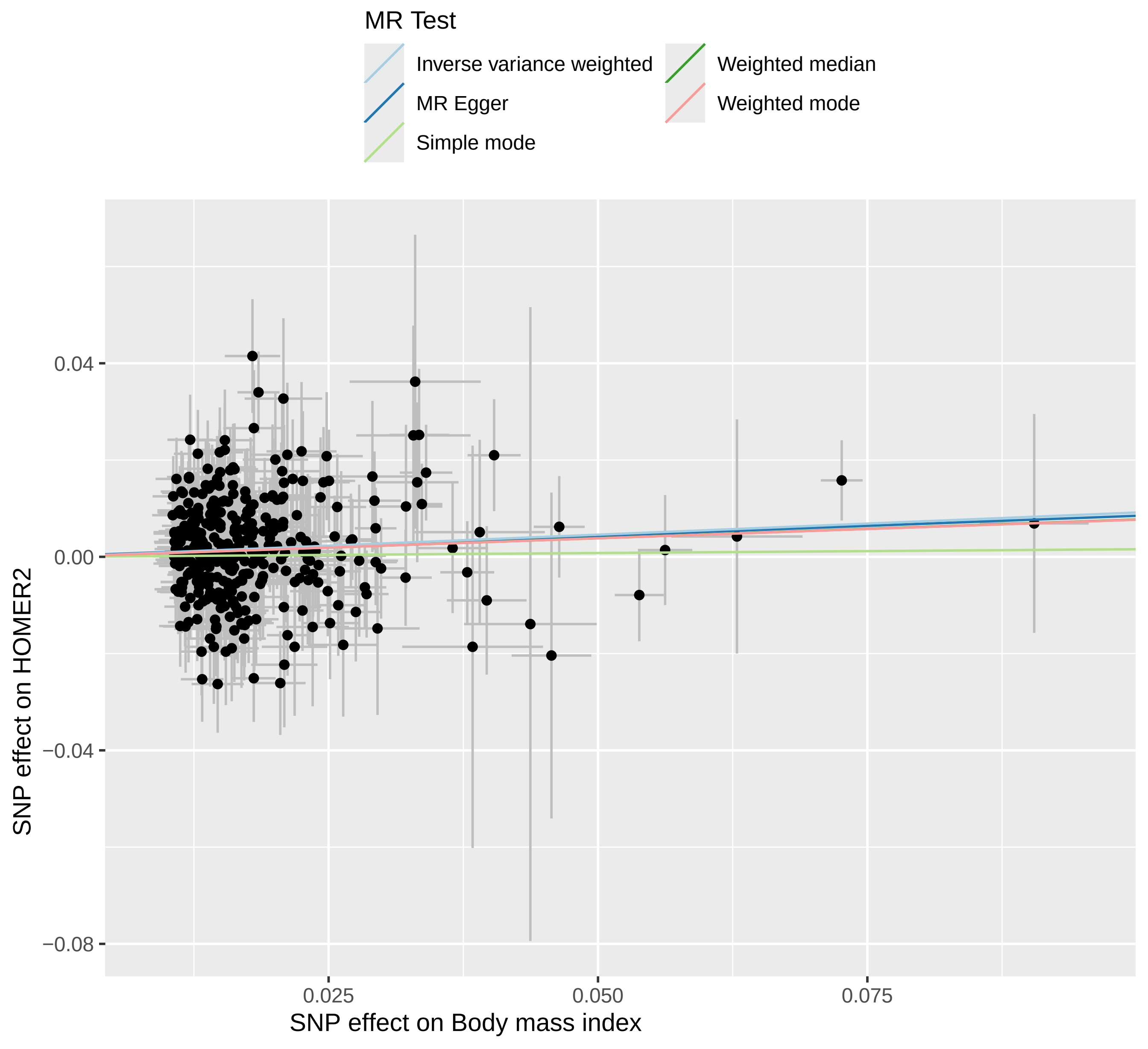
MR Test
Inverse variance weighted
MR Egger
Simple mode
Weighted median
Weighted mode
0.04
0.00
−0.04
−0.08
0.025
0.050
0.075
SNP effect on HOMER2
SNP effect on Body mass index

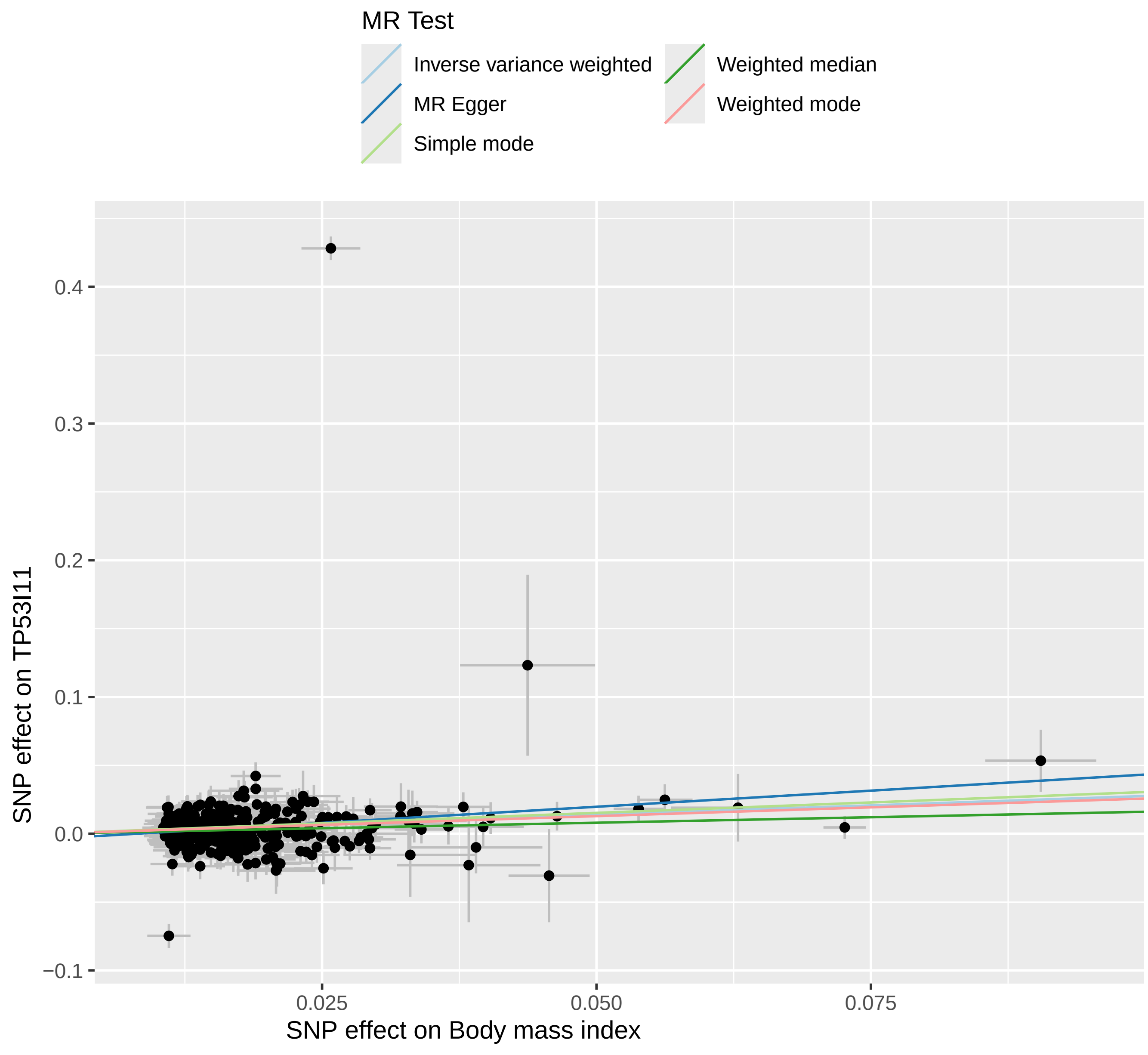
MR Test
Inverse variance weighted
MR Egger
Simple mode
Weighted median
Weighted mode
SNP effect on TP53I11
0.4
0.3
0.2
0.1
0.0
−0.1
0.025
0.050
0.075
SNP effect on Body mass index

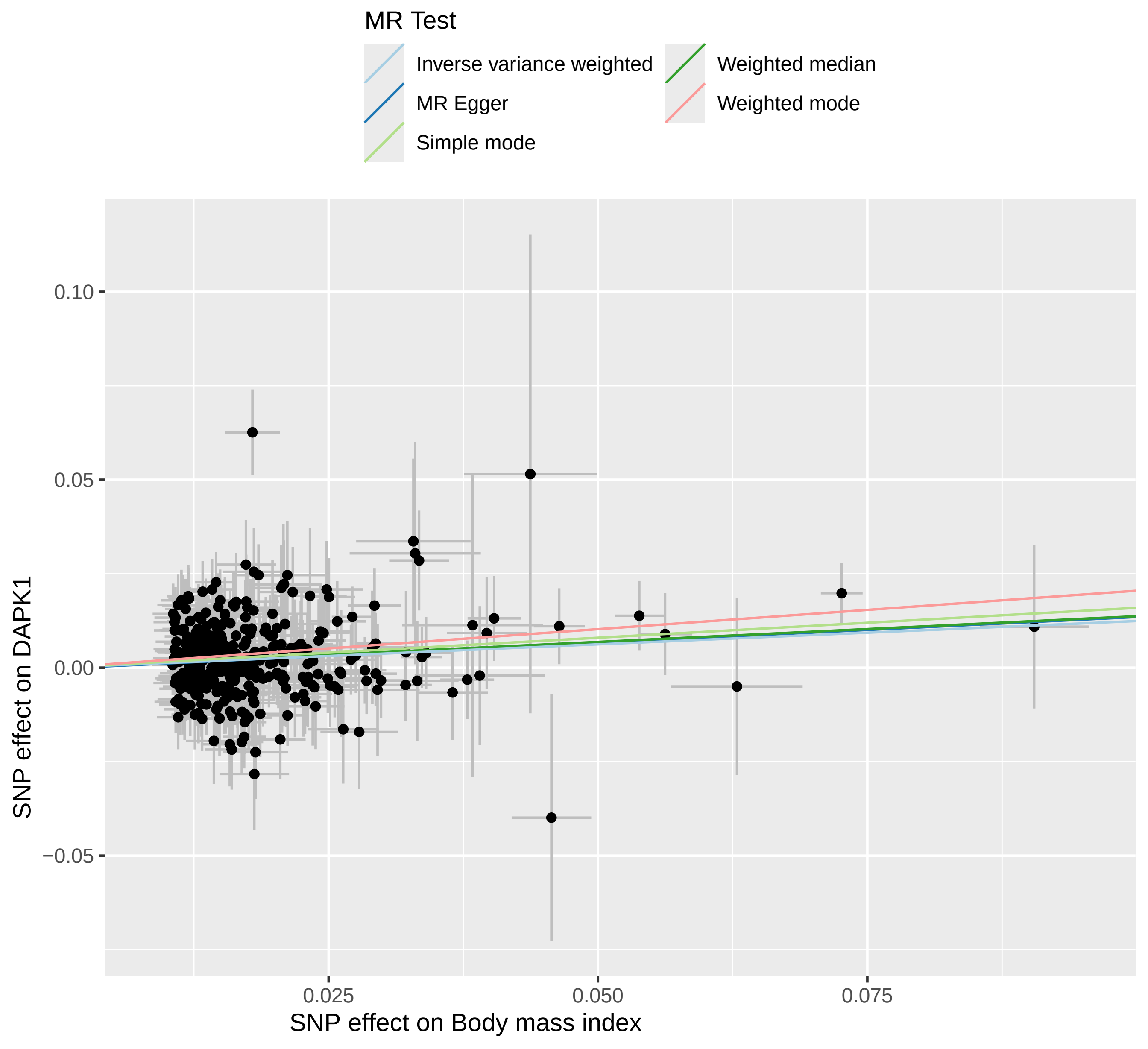
MR Test
Inverse variance weighted
MR Egger
Simple mode
Weighted median
Weighted mode
0.10
0.05
0.00
−0.05
0.025
0.050
0.075
SNP effect on DAPK1
SNP effect on Body mass index

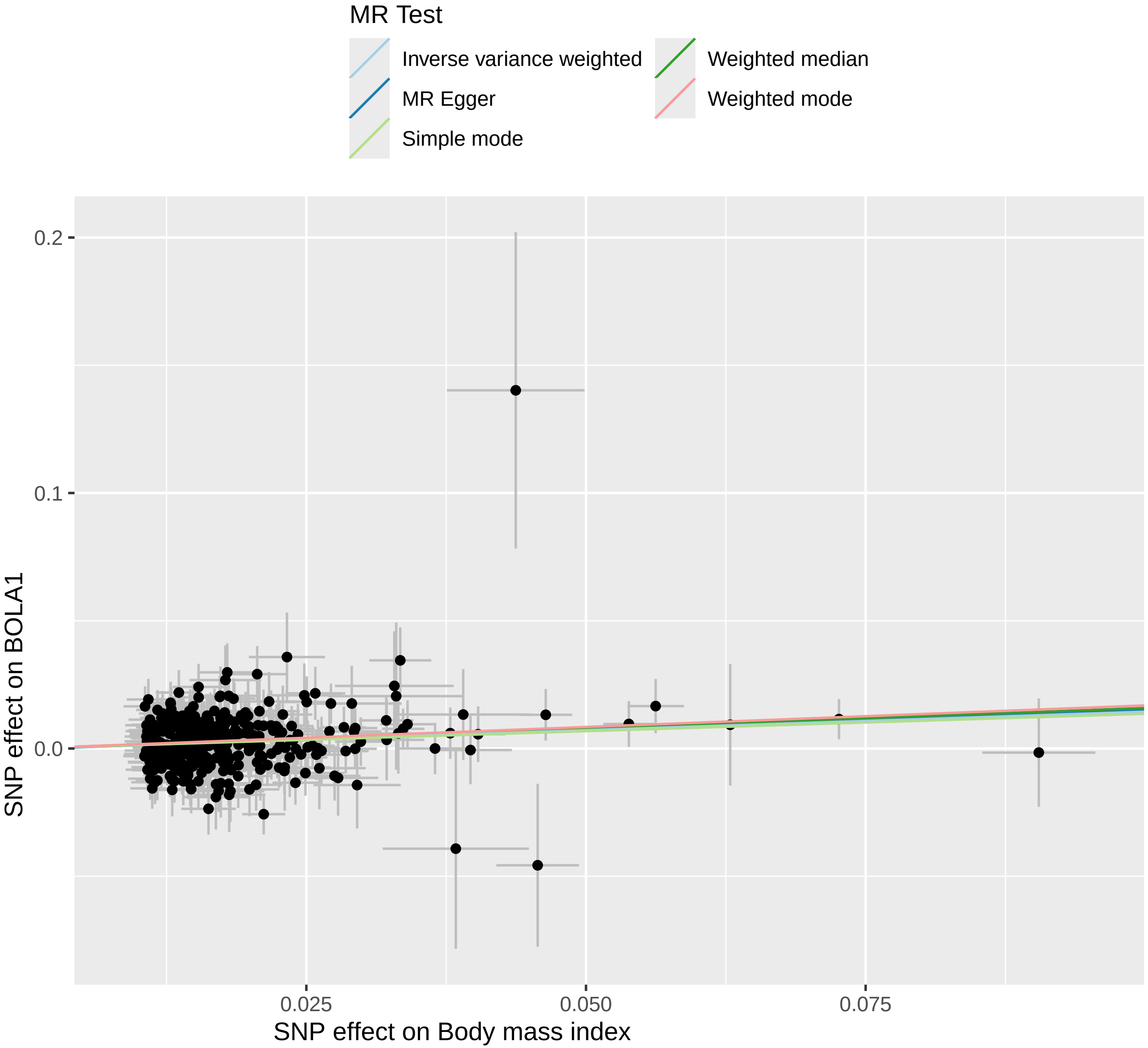

MR Test
Inverse variance weighted
MR Egger
Simple mode
Weighted median
Weighted mode
0.2
0.1
0.0
0.025
0.050
0.075
SNP effect on BOLA1
SNP effect on Body mass index

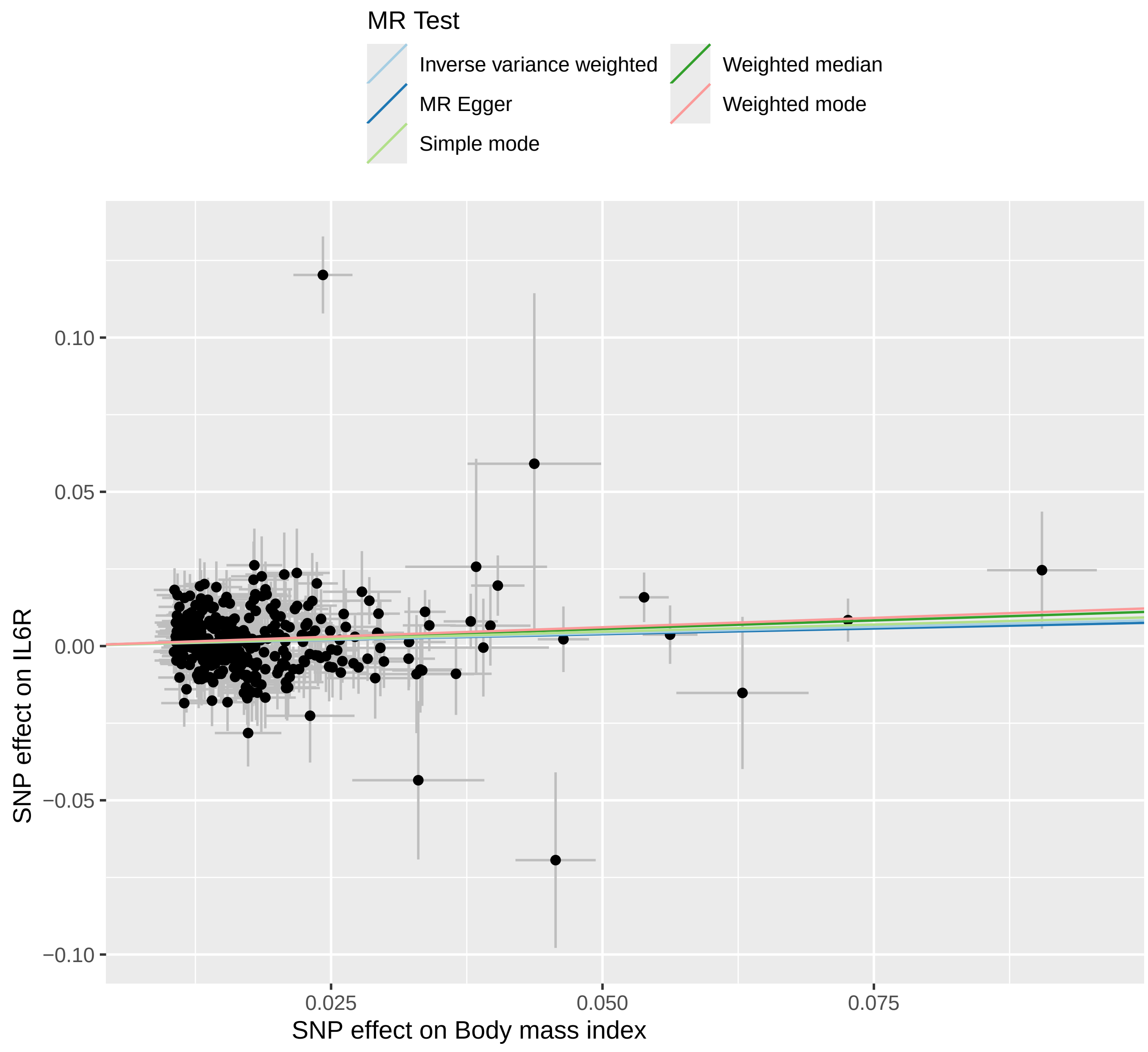
MR Test
Inverse variance weighted
MR Egger
Simple mode
Weighted median
Weighted mode
0.10
0.05
0.00
−0.05
−0.10
0.025
0.050
0.075
SNP effect on IL6R
SNP effect on Body mass index

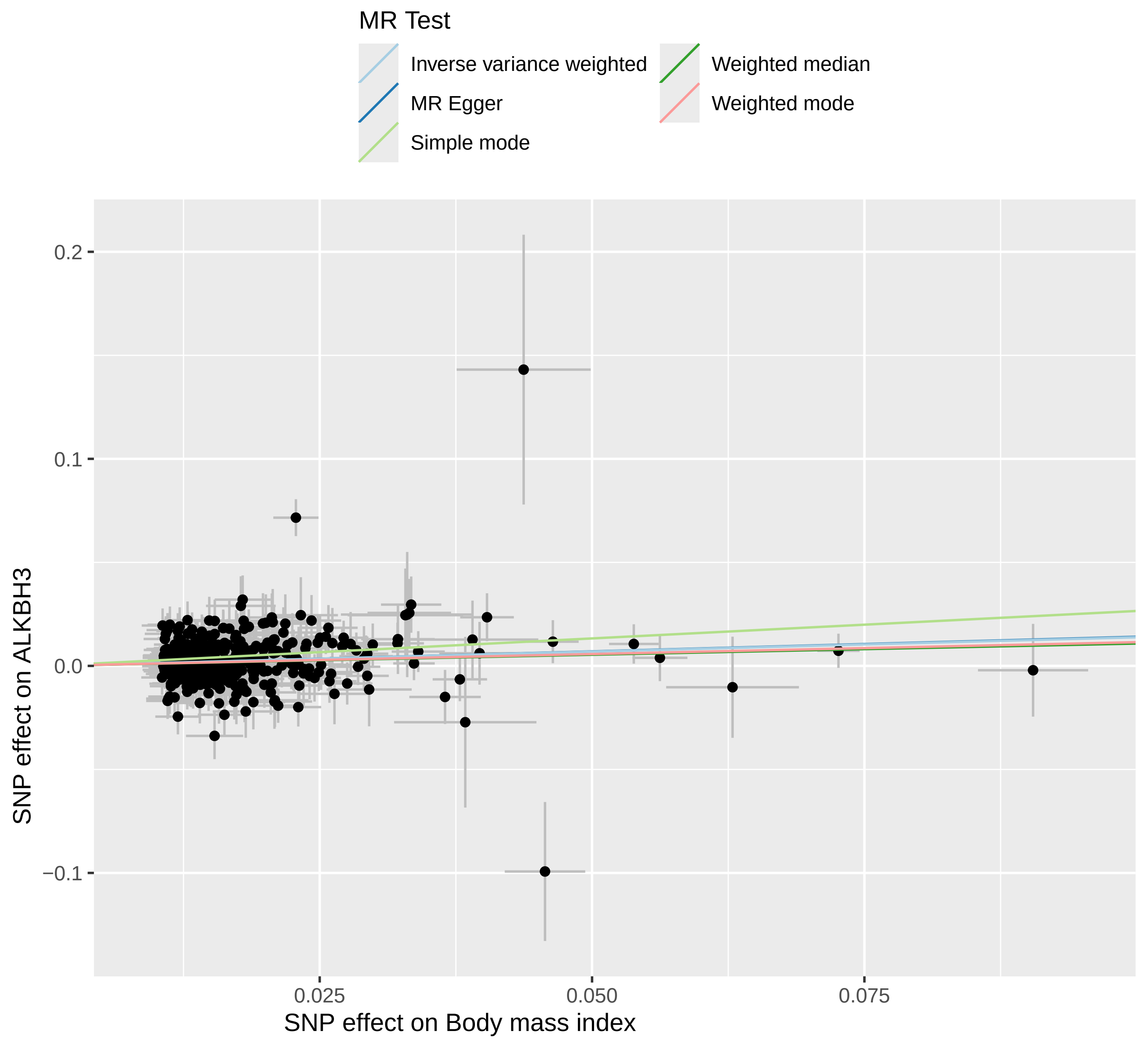
MR Test
Inverse variance weighted
MR Egger
Simple mode
Weighted median
Weighted mode
0.2
0.1
0.0
−0.1
0.025
0.050
0.075
SNP effect on ALKBH3
SNP effect on Body mass index

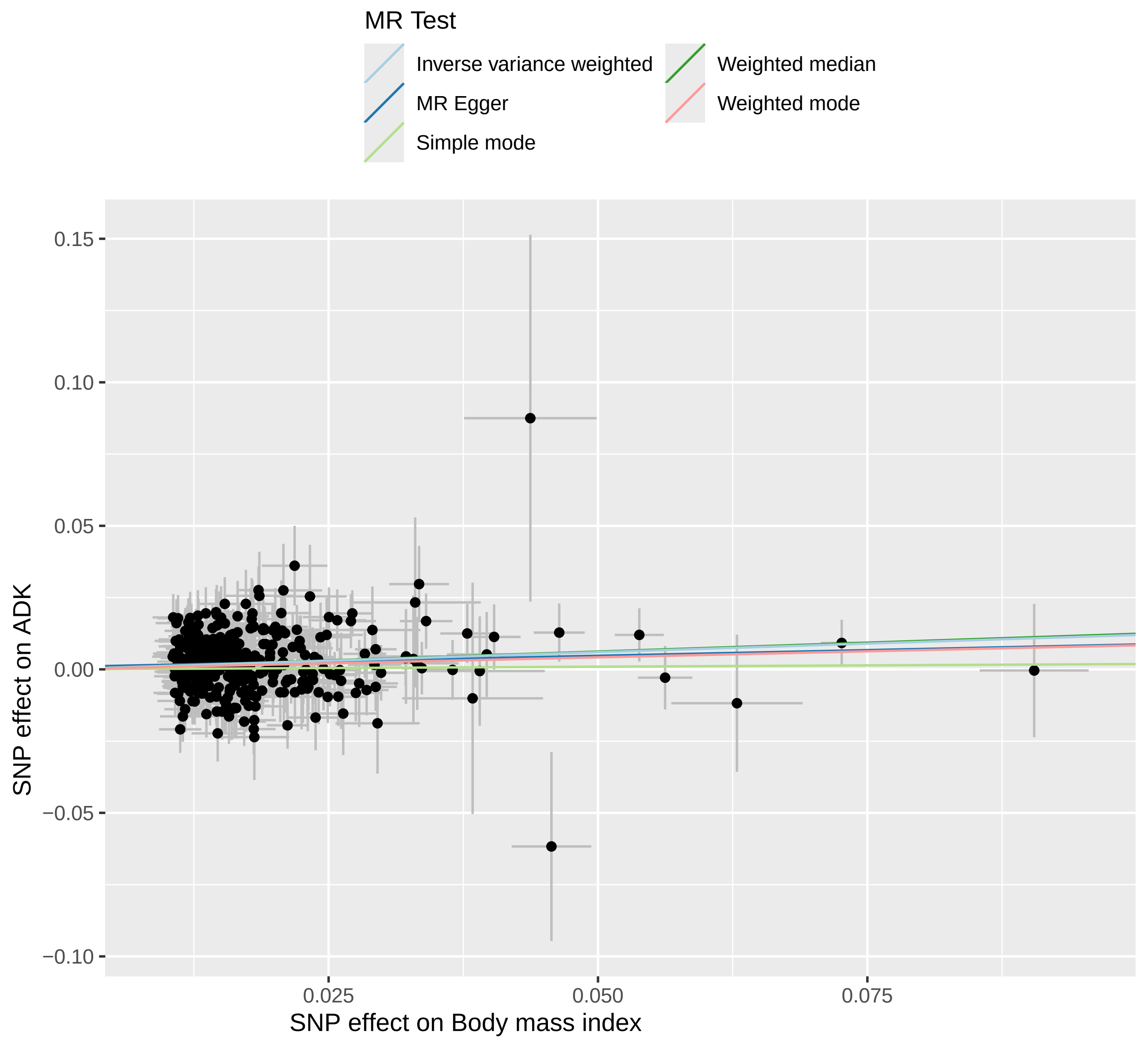
MR Test
Inverse variance weighted
MR Egger
Simple mode
Weighted median
Weighted mode
0.15
0.10
0.05
0.00
−0.05
−0.10
SNP effect on ADK
0.025
0.050
0.075
SNP effect on Body mass index

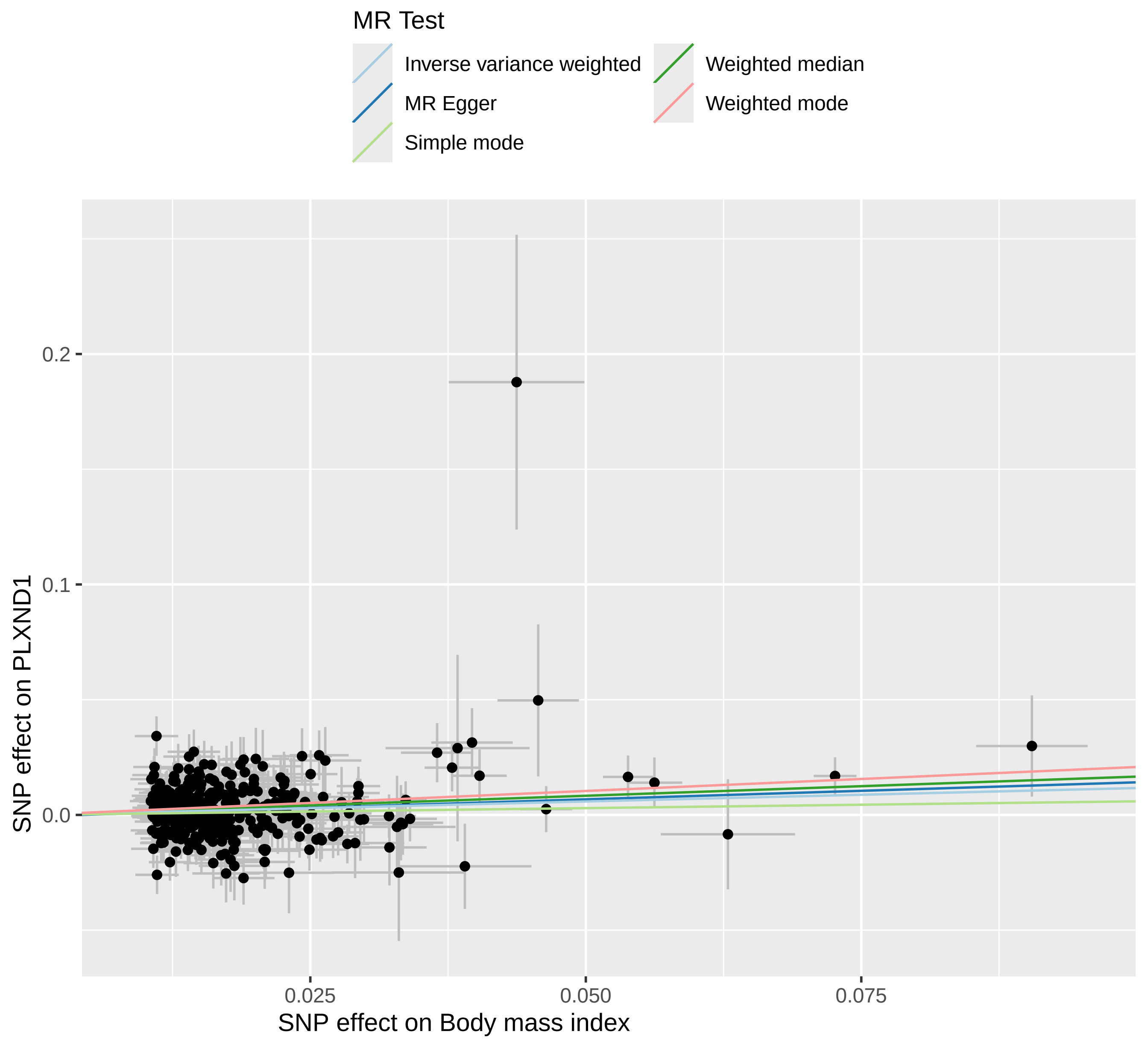
MR Test
Inverse variance weighted
MR Egger
Simple mode
Weighted median
Weighted mode
SNP effect on PLXND1
0.2
0.1
0.0
0.025
0.050
0.075
SNP effect on Body mass index

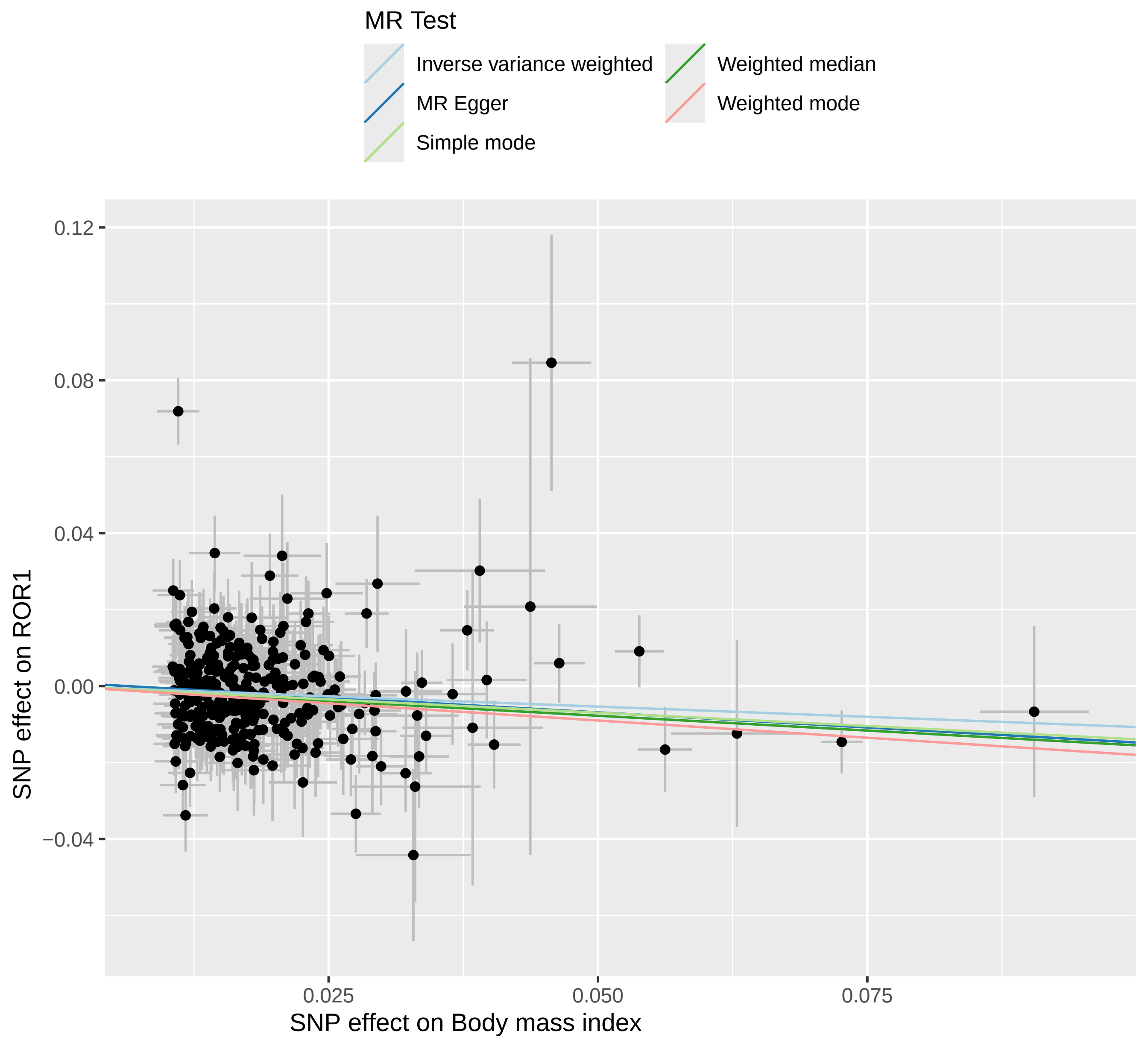

MR Test
Inverse variance weighted
MR Egger
Simple mode
Weighted median
Weighted mode
0.12
0.08
0.04
0.00
−0.04
0.025
0.050
0.075
SNP effect on ROR1
SNP effect on Body mass index

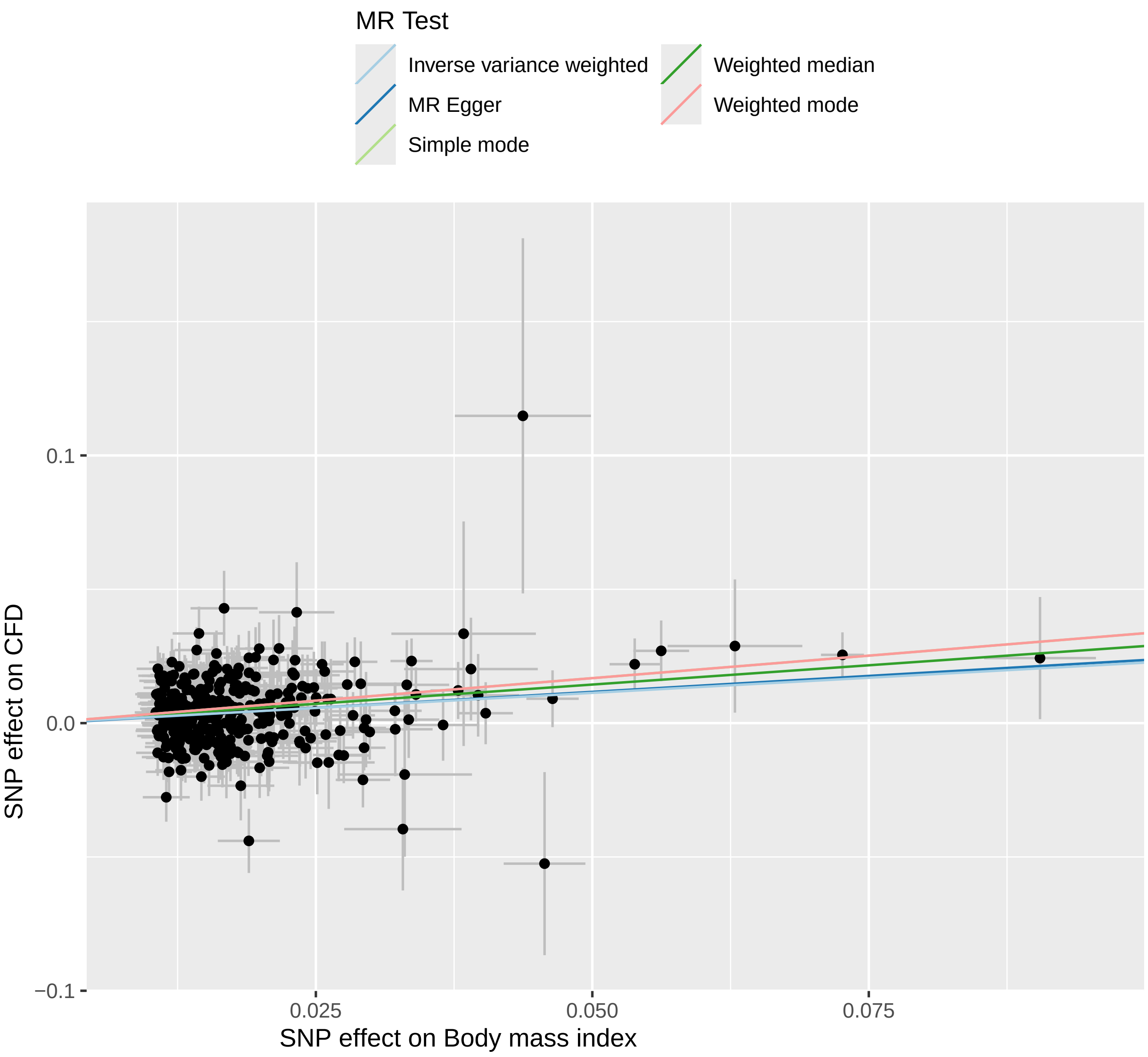

MR Test
Inverse variance weighted
MR Egger
Simple mode
Weighted median
Weighted mode
0.1
0.0
−0.1
0.025
0.050
0.075
SNP effect on CFD
SNP effect on Body mass index

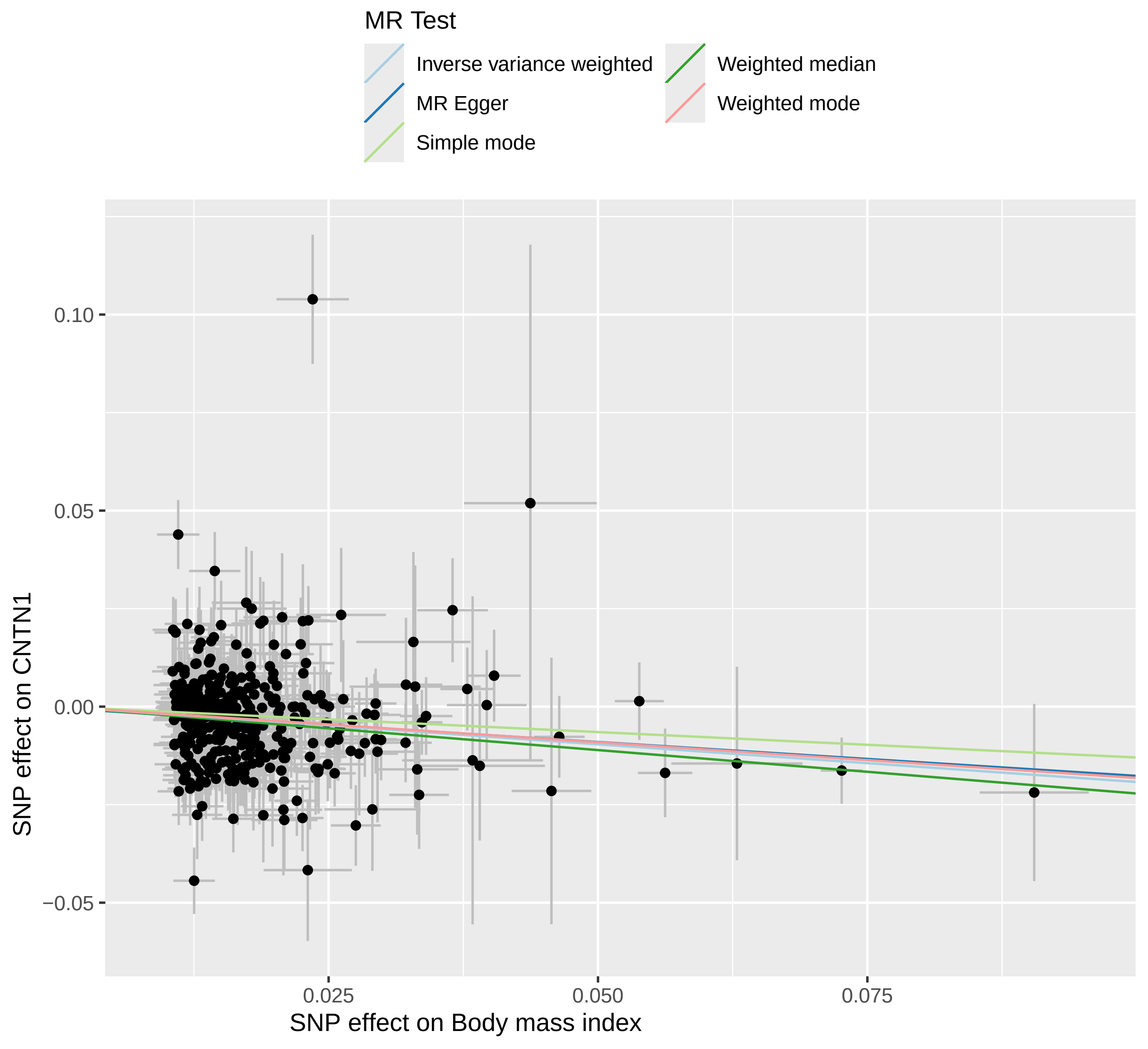

MR Test
Inverse variance weighted
MR Egger
Simple mode
Weighted median
Weighted mode
0.10
0.05
0.00
−0.05
0.025
0.050
0.075
SNP effect on CNTN1
SNP effect on Body mass index

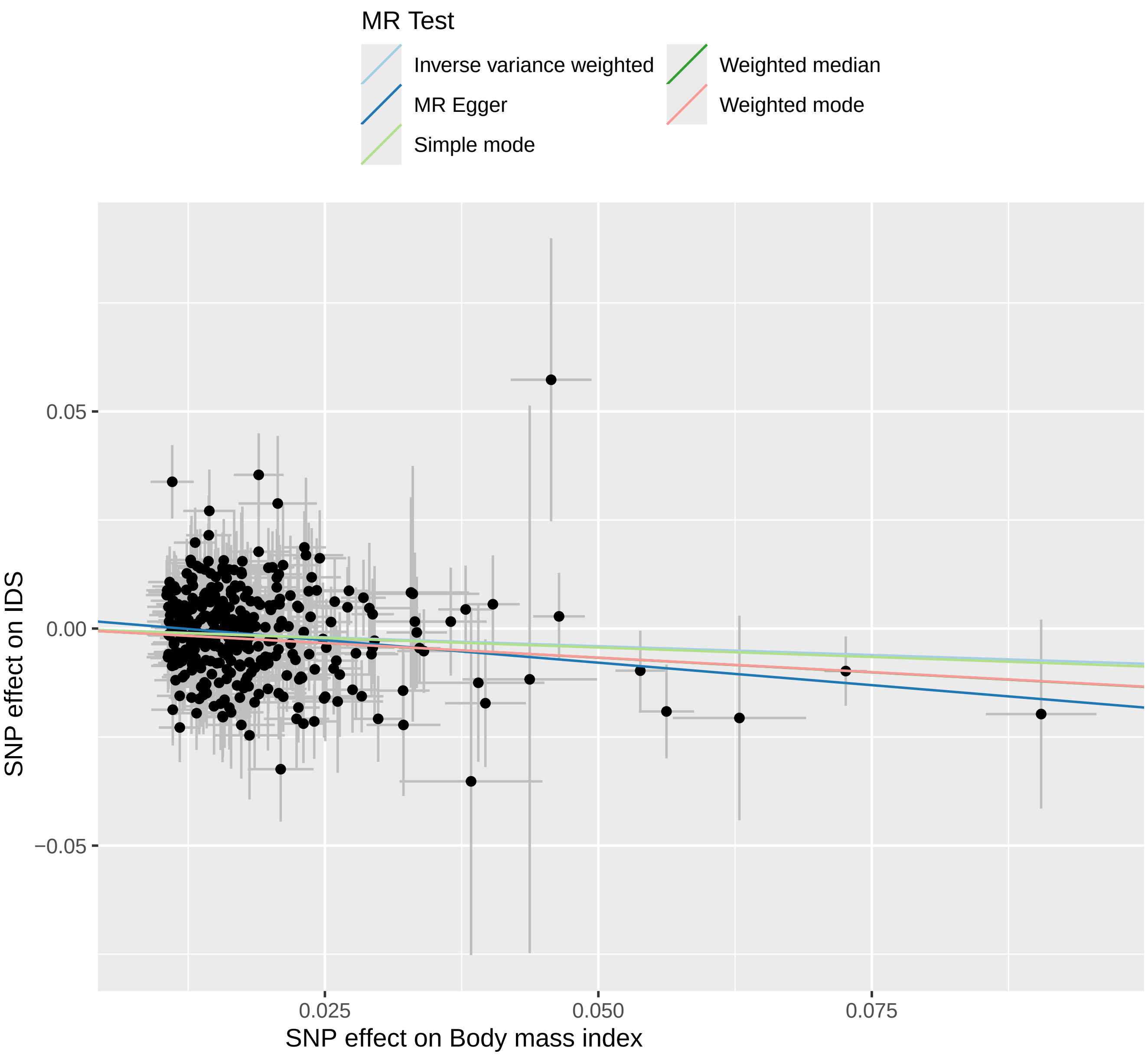

MR Test
Inverse variance weighted
MR Egger
Simple mode
Weighted median
Weighted mode
0.05
0.00
−0.05
SNP effect on IDS
0.025
0.050
0.075
SNP effect on Body mass index

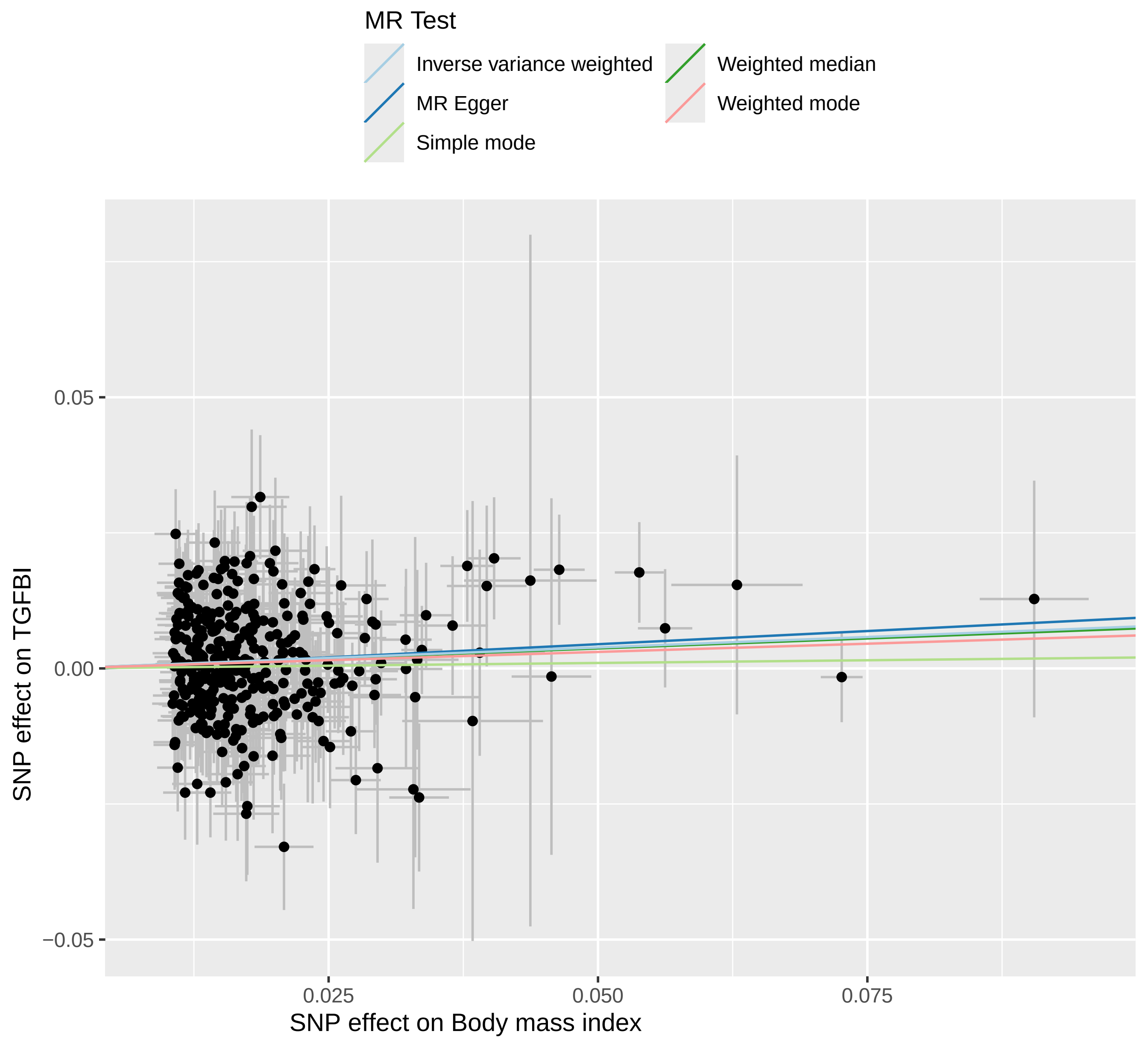
MR Test
Inverse variance weighted
MR Egger
Simple mode
Weighted median
Weighted mode
0.05
0.00
−0.05
0.025
0.050
0.075
SNP effect on Body mass index
SNP effect on TGFBI

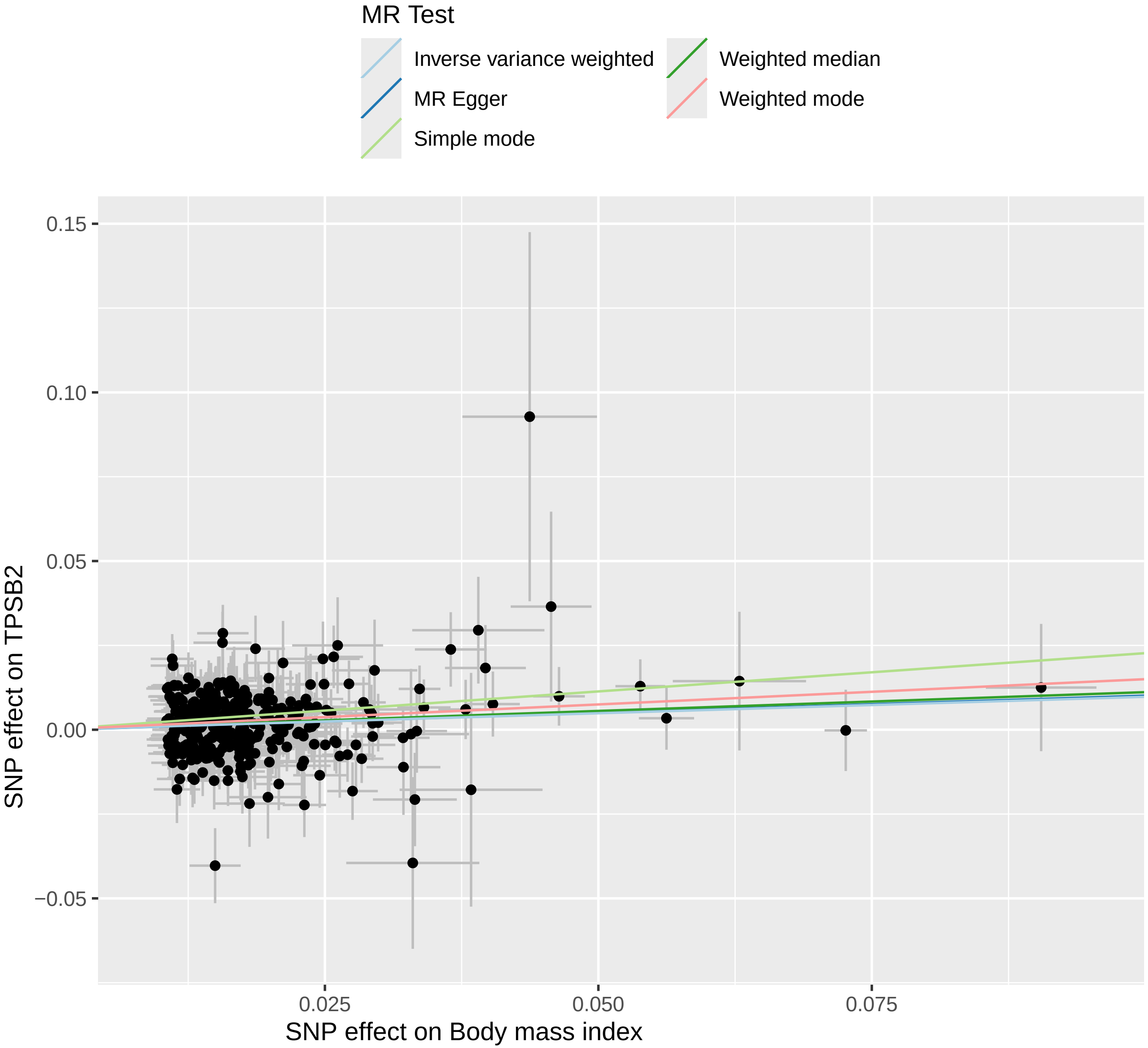
MR Test
Inverse variance weighted
MR Egger
Simple mode
Weighted median
Weighted mode
0.15
0.10
0.05
0.00
−0.05
SNP effect on TPSB2
0.025
0.050
0.075
SNP effect on Body mass index

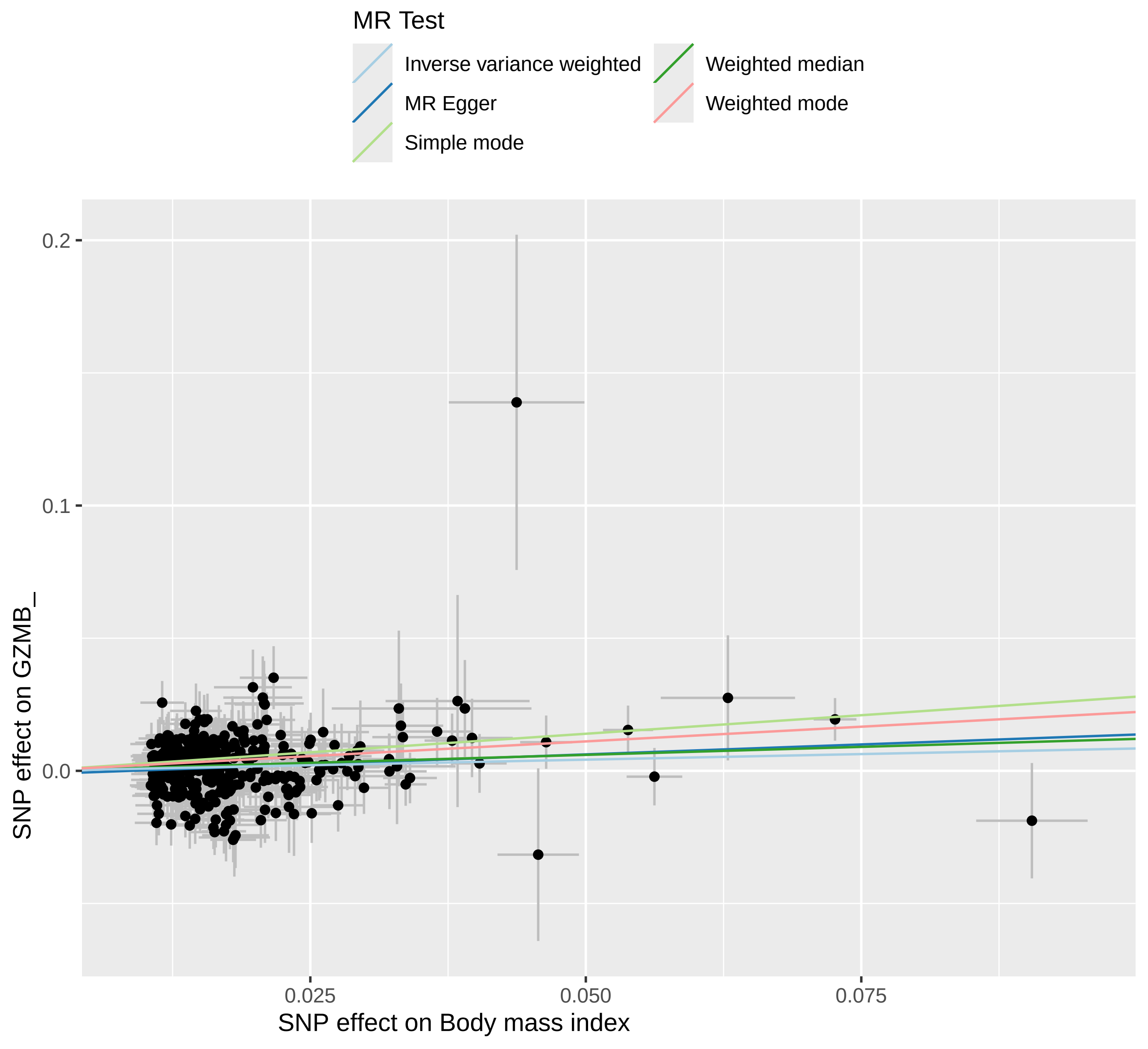

MR Test
Inverse variance weighted
MR Egger
Simple mode
Weighted median
Weighted mode
0.2
0.1
0.0
SNP effect on GZMB_
0.025
0.050
0.075
SNP effect on Body mass index

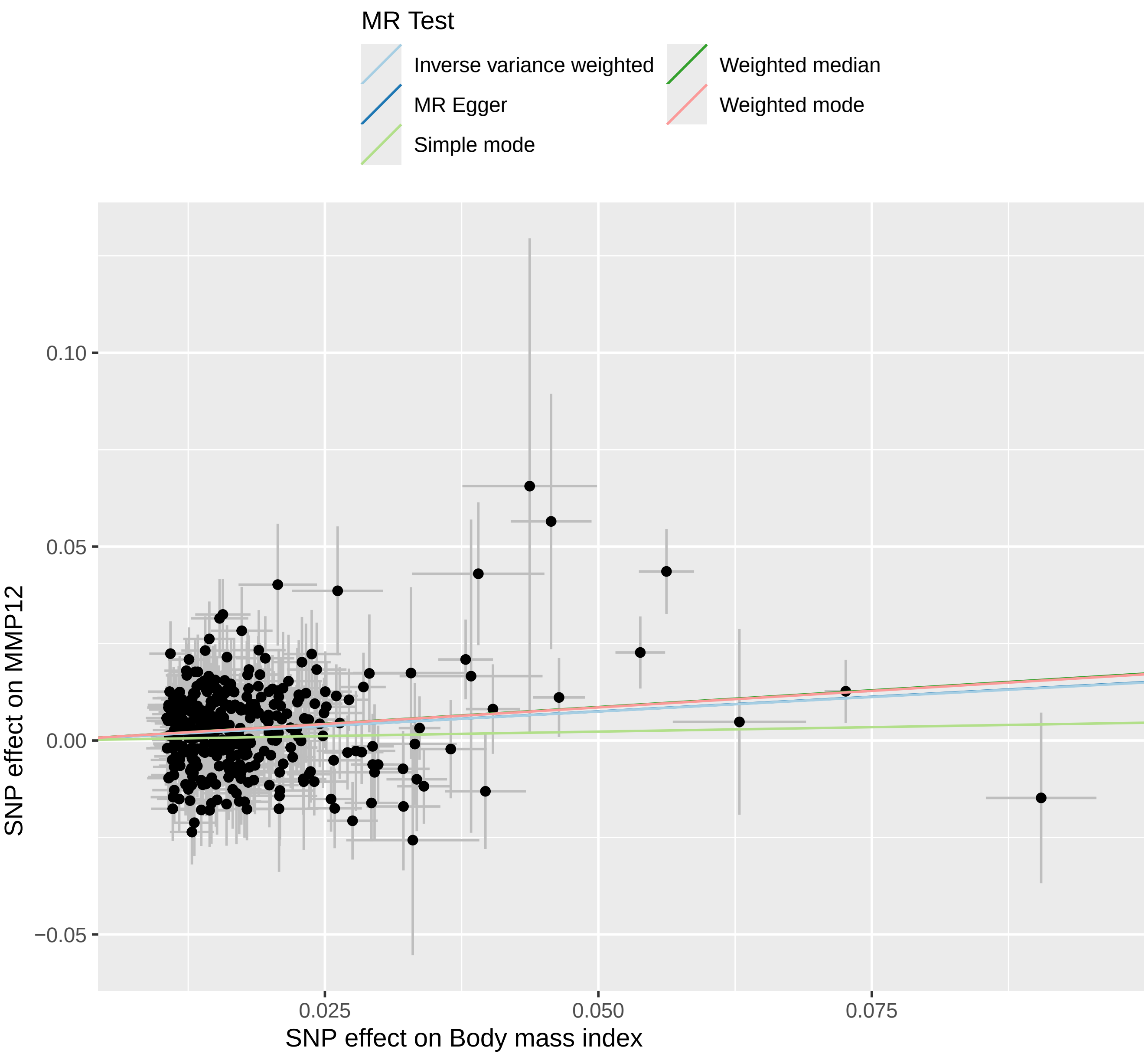

MR Test
Inverse variance weighted
MR Egger
Simple mode
Weighted median
Weighted mode
0.10
0.05
0.00
−0.05
0.025
0.050
0.075
SNP effect on MMP12
SNP effect on Body mass index

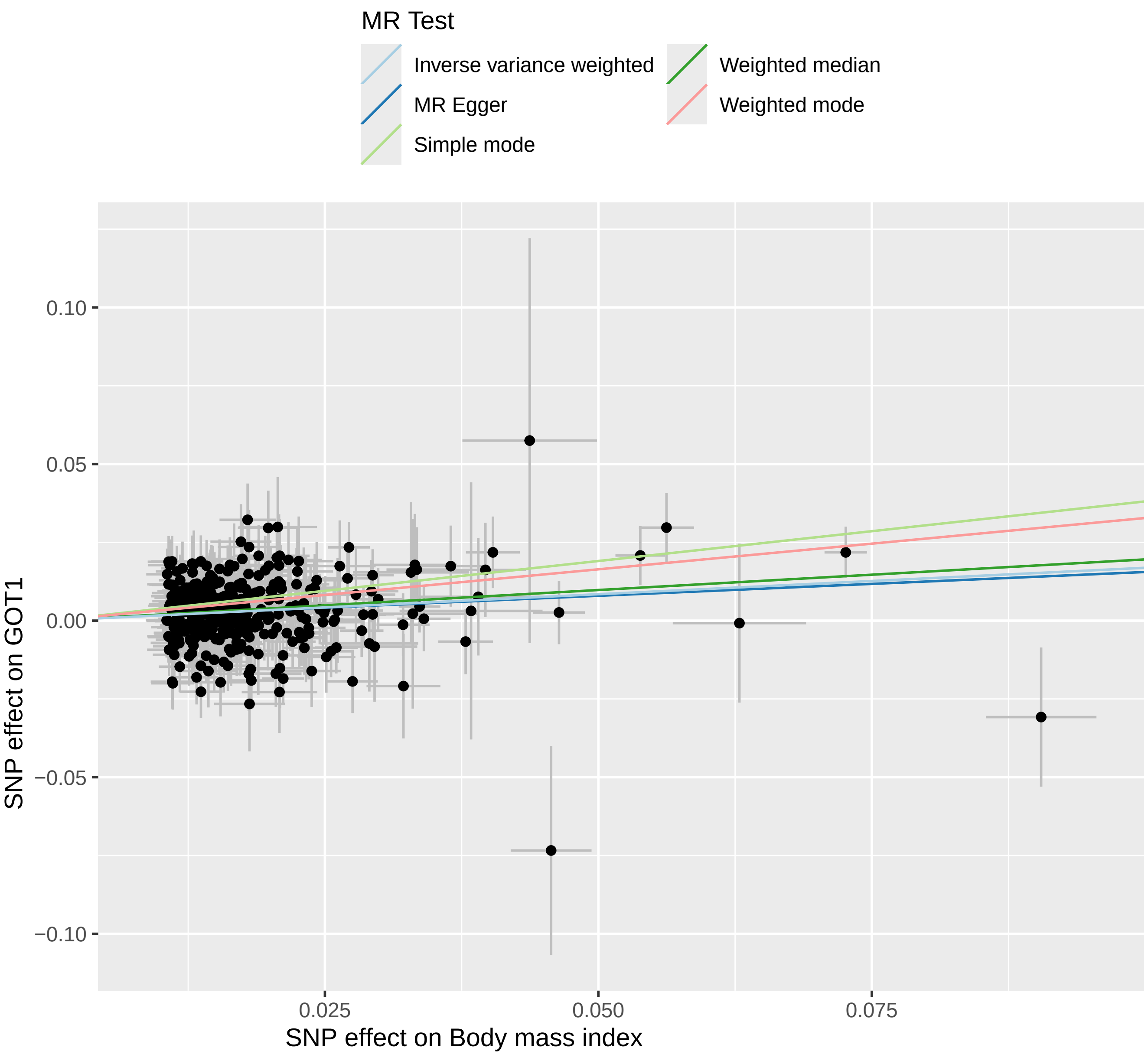
MR Test
Inverse variance weighted
MR Egger
Simple mode
Weighted median
Weighted mode
0.10
0.05
0.00
−0.05
−0.10
SNP effect on GOT1
0.025
0.050
0.075
SNP effect on Body mass index

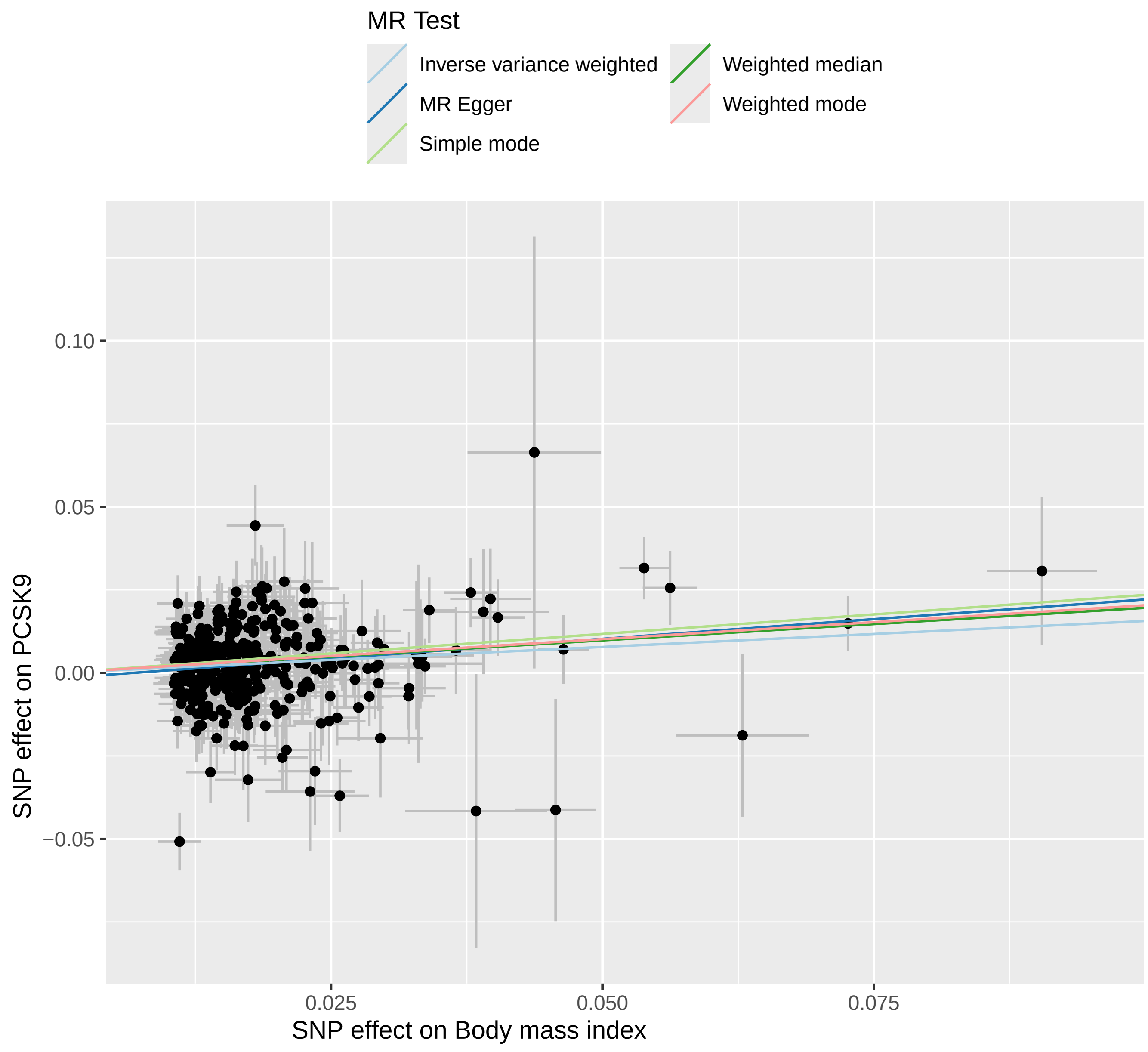

MR Test
Inverse variance weighted
MR Egger
Simple mode
Weighted median
Weighted mode
0.10
0.05
0.00
−0.05
0.025
0.050
0.075
SNP effect on PCSK9
SNP effect on Body mass index

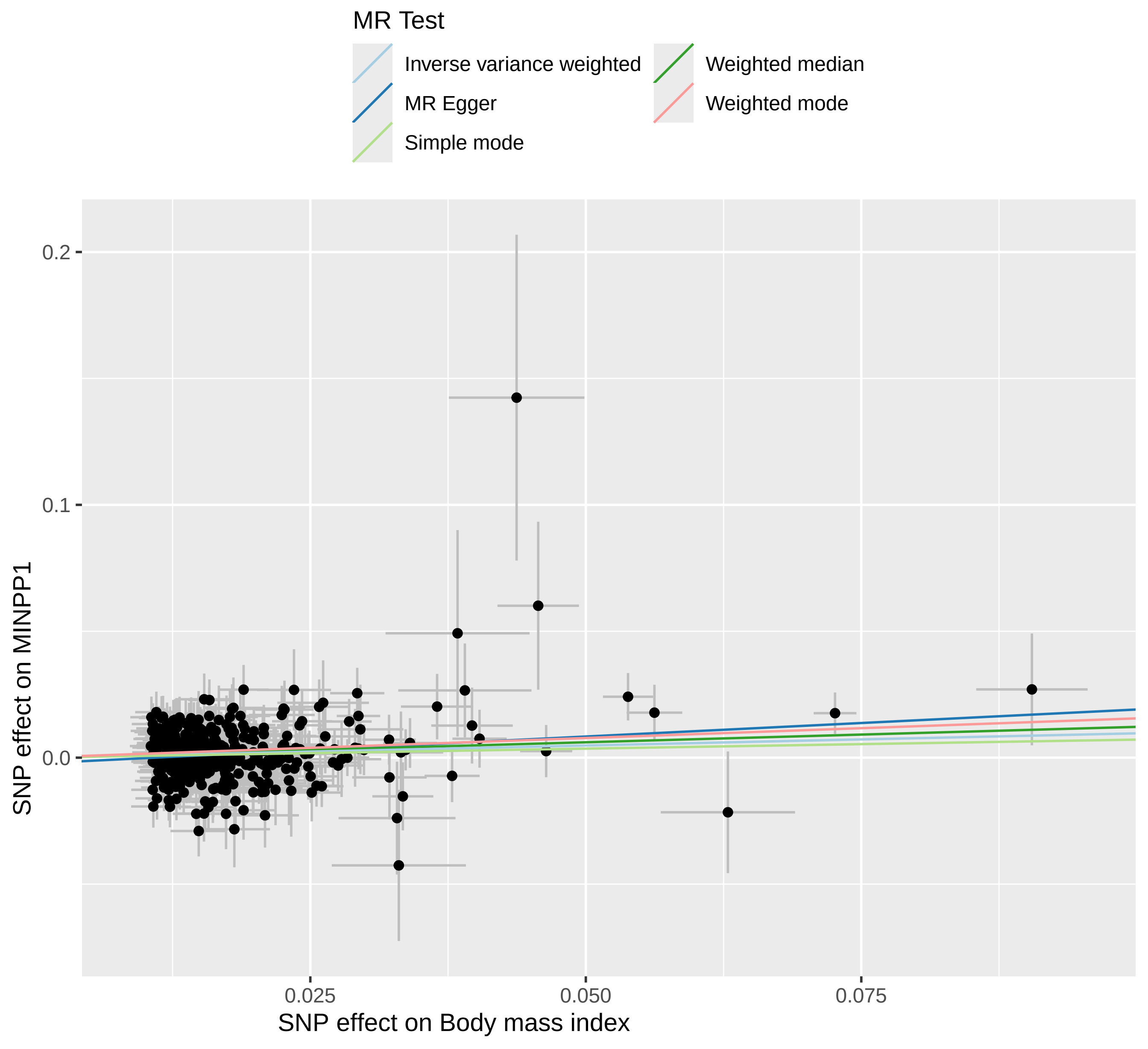
MR Test
Inverse variance weighted
MR Egger
Simple mode
Weighted median
Weighted mode
0.2
0.1
0.0
0.025
0.050
0.075
SNP effect on Body mass index
SNP effect on MINPP1

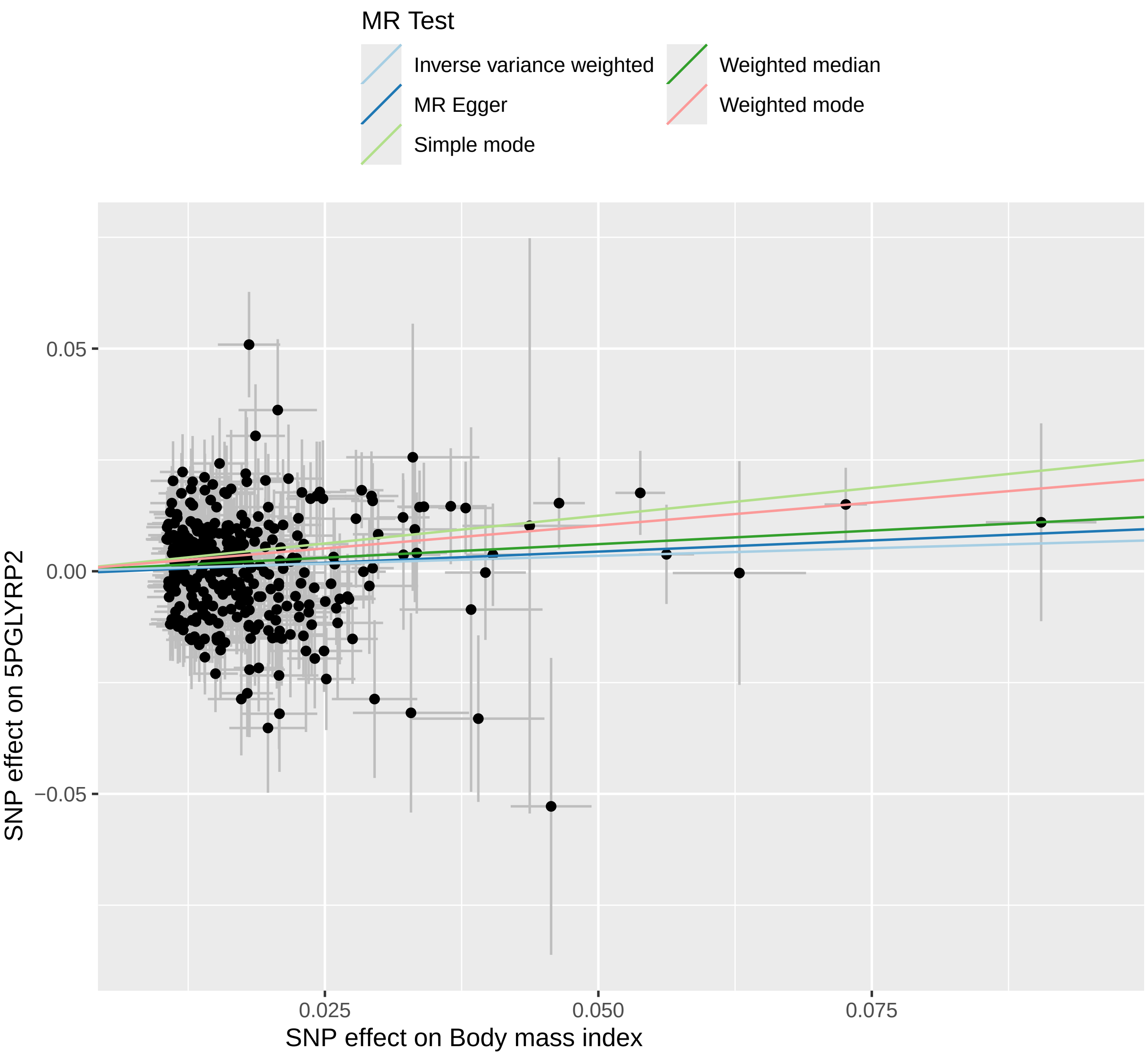

MR Test
Inverse variance weighted
MR Egger
Simple mode
Weighted median
Weighted mode
0.05
0.00
−0.05
0.025
0.050
0.075
SNP effect on 5PGLYRP2
SNP effect on Body mass index

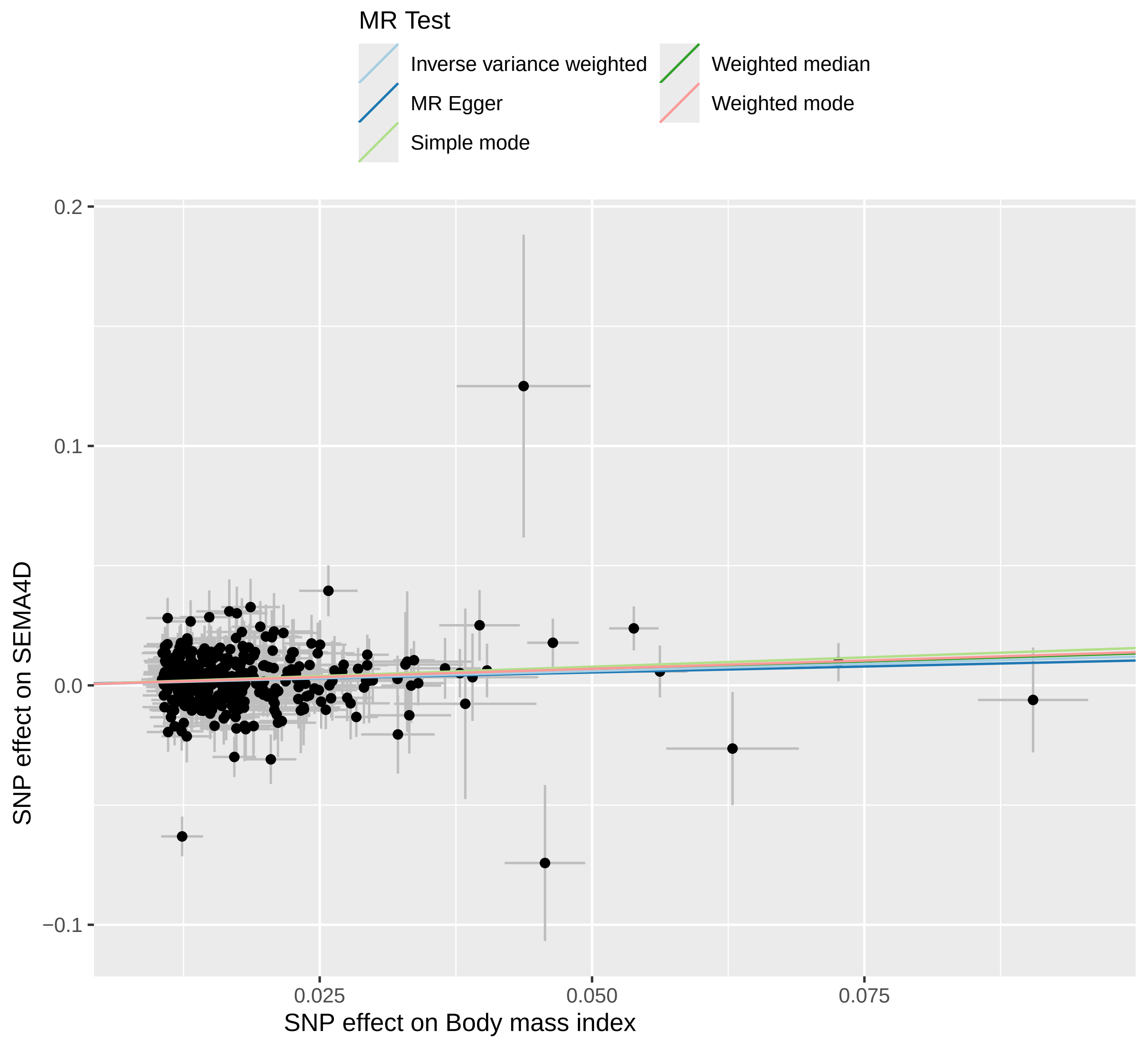
MR Test
Inverse variance weighted
MR Egger
Simple mode
Weighted median
Weighted mode
0.2
0.1
0.0
−0.1
0.025
0.050
0.075
SNP effect on SEMA4D
SNP effect on Body mass index

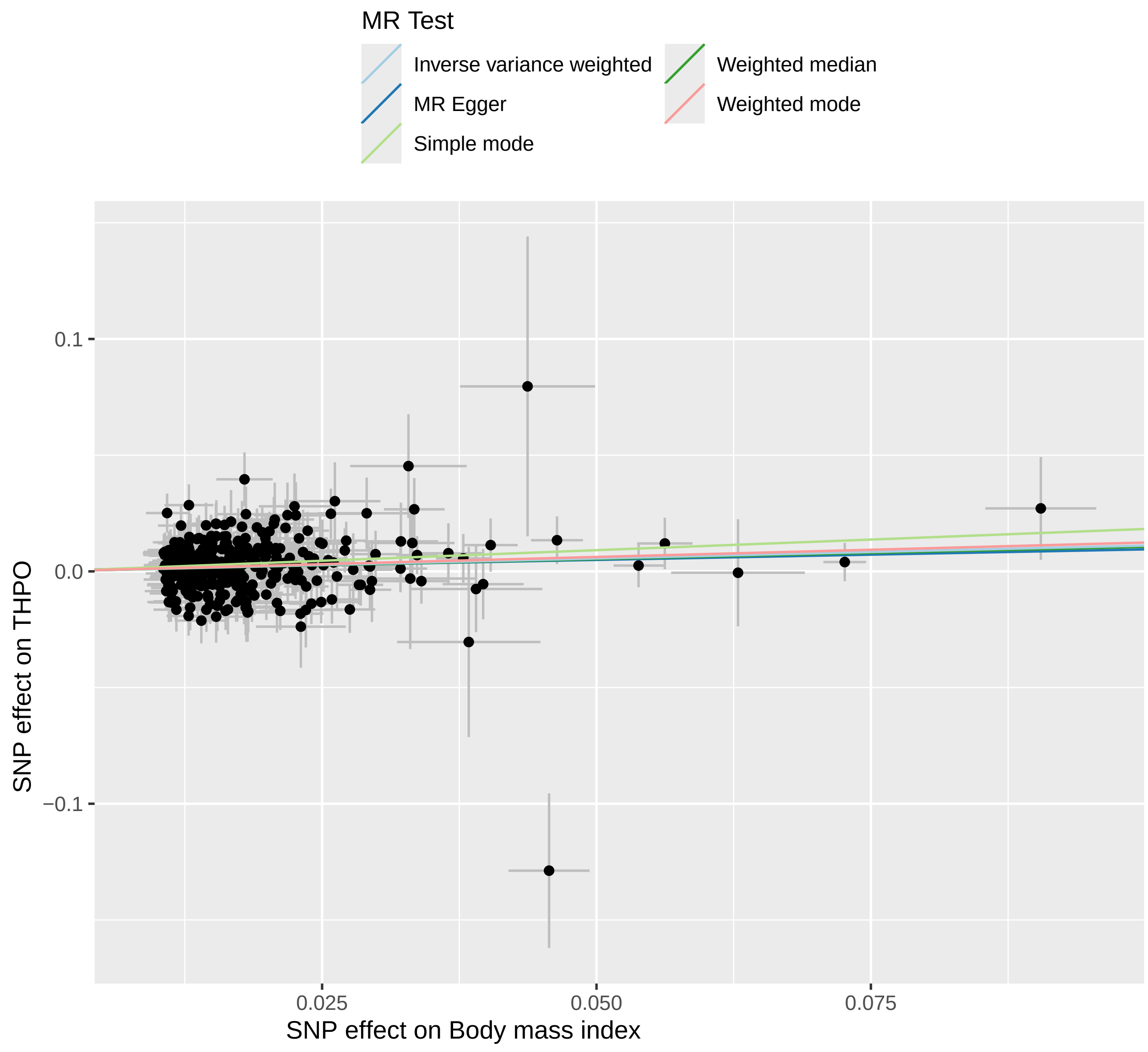
MR Test
Inverse variance weighted
MR Egger
Simple mode
Weighted median
Weighted mode
SNP effect on THPO
0.1
0.0
−0.1
0.025
0.050
0.075
SNP effect on Body mass index

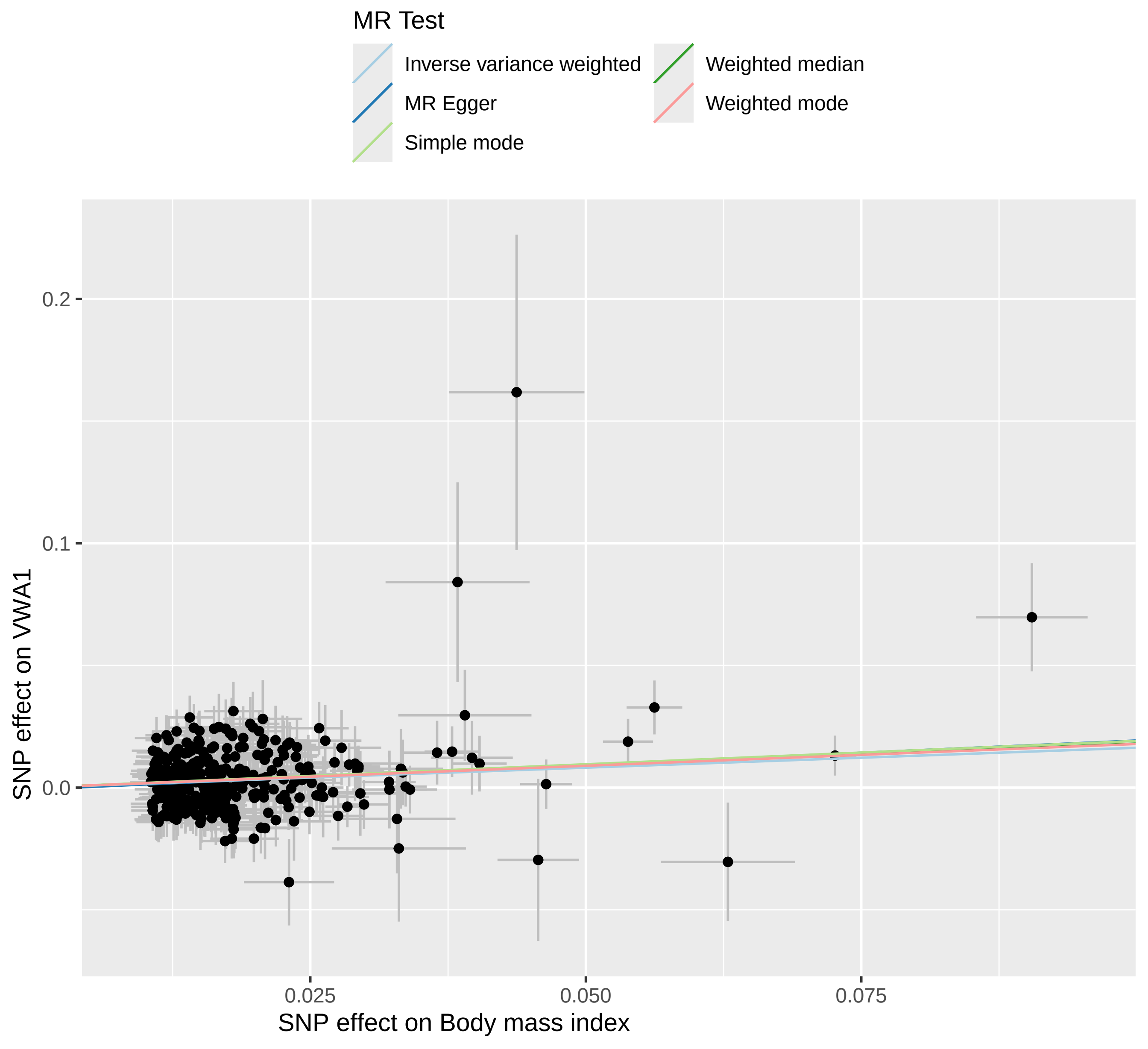
MR Test
Inverse variance weighted
MR Egger
Simple mode
Weighted median
Weighted mode
0.2
0.1
0.0
0.025
0.050
0.075
SNP effect on VWA1
SNP effect on Body mass index

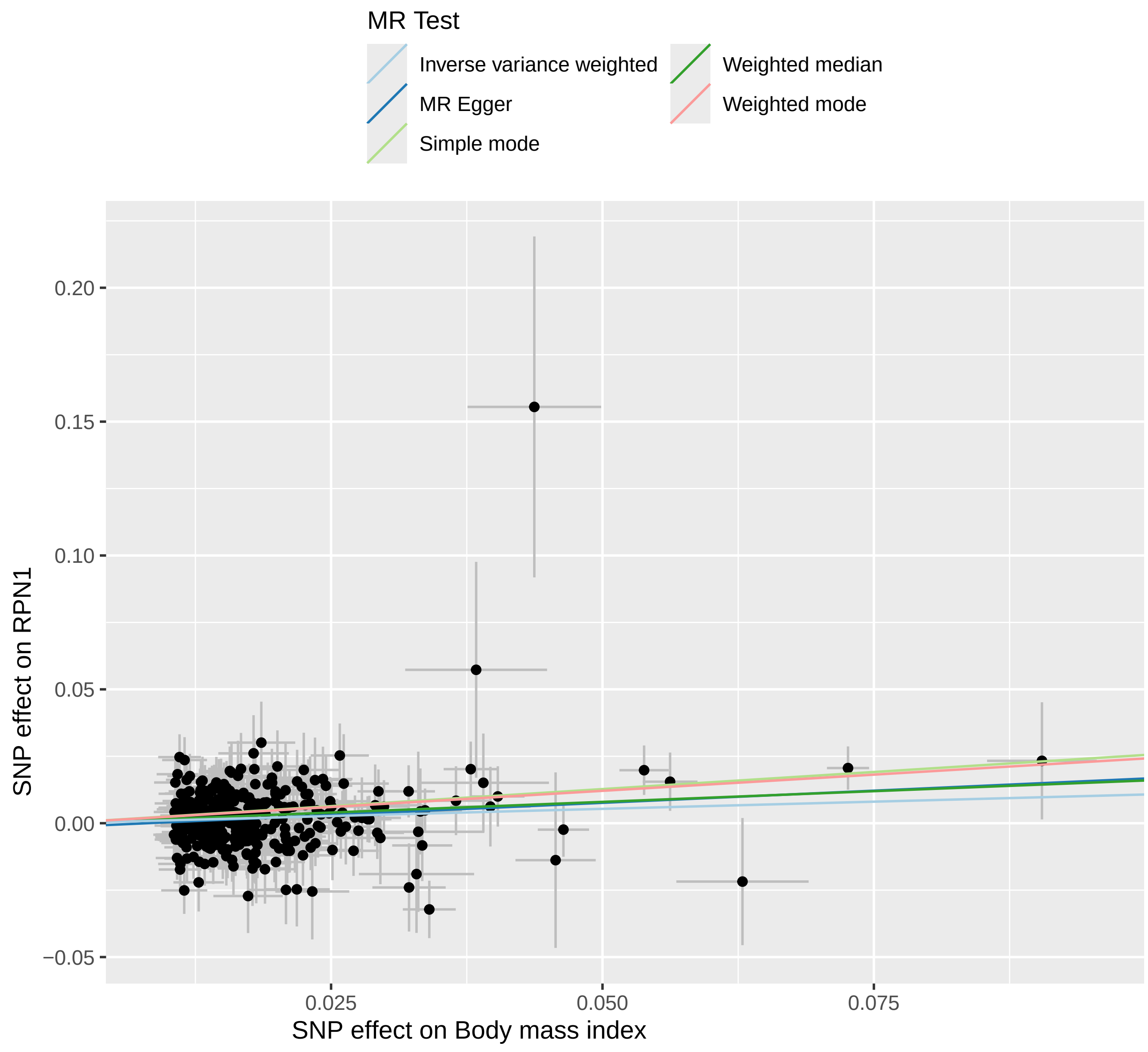
MR Test
Inverse variance weighted
MR Egger
Simple mode
Weighted median
Weighted mode
0.20
0.15
0.10
0.05
0.00
−0.05
0.025
0.050
0.075
SNP effect on RPN1
SNP effect on Body mass index

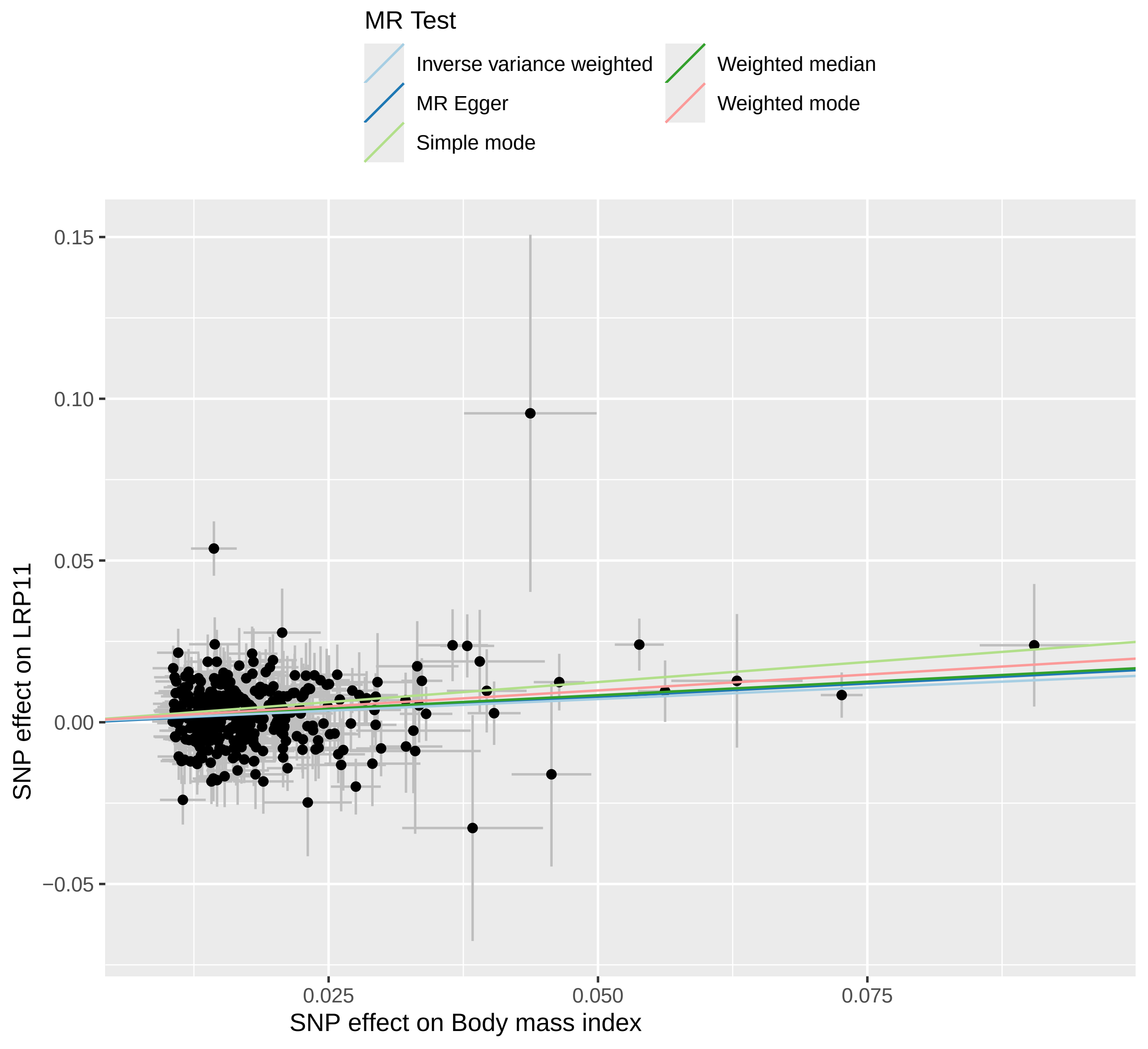
MR Test
Inverse variance weighted
MR Egger
Simple mode
Weighted median
Weighted mode
0.15
0.10
0.05
0.00
−0.05
0.025
0.050
0.075
SNP effect on LRP11
SNP effect on Body mass index

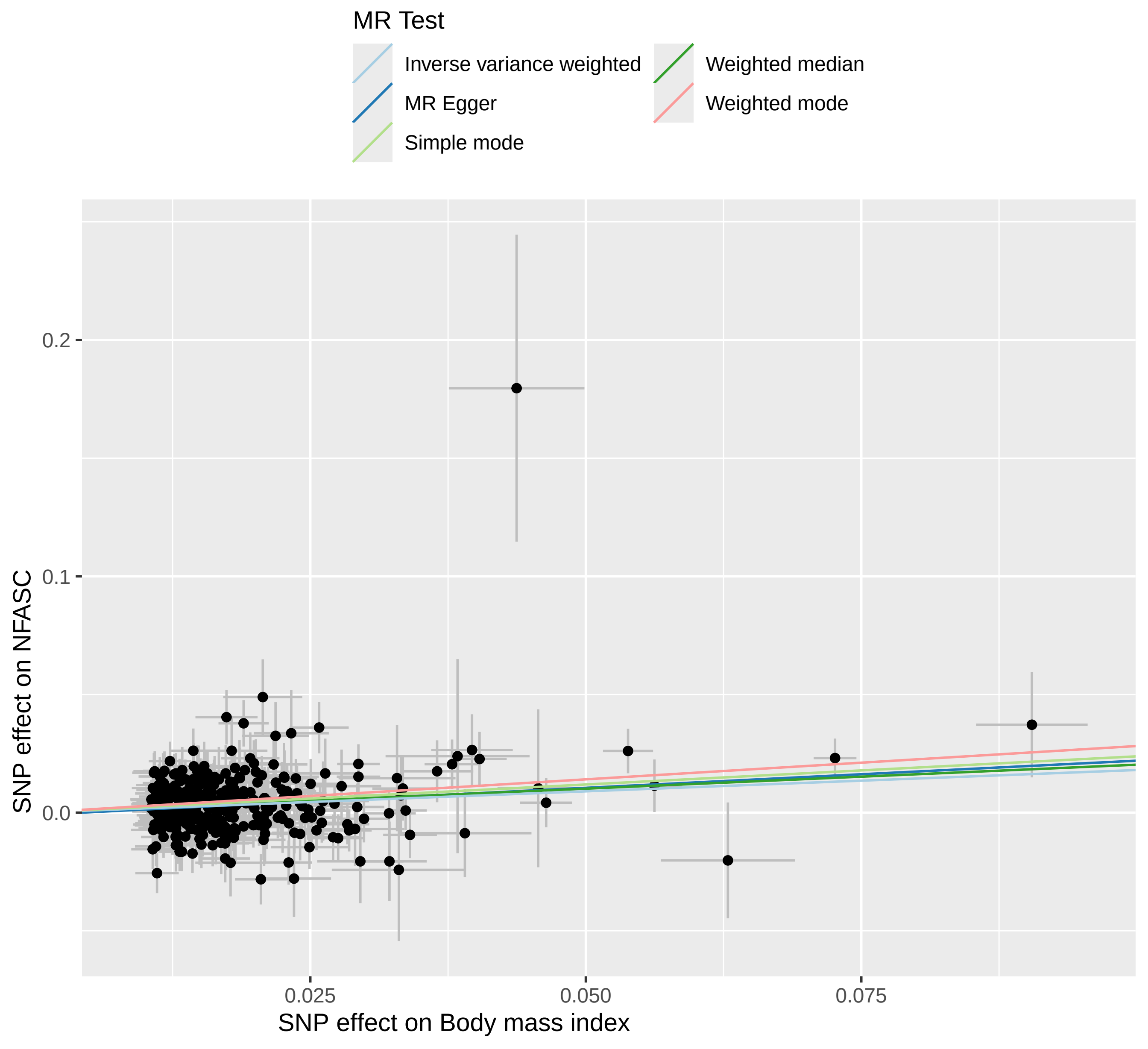
MR Test
Inverse variance weighted
MR Egger
Simple mode
Weighted median
Weighted mode
SNP effect on NFASC
0.2
0.1
0.0
0.025
0.050
0.075
SNP effect on Body mass index

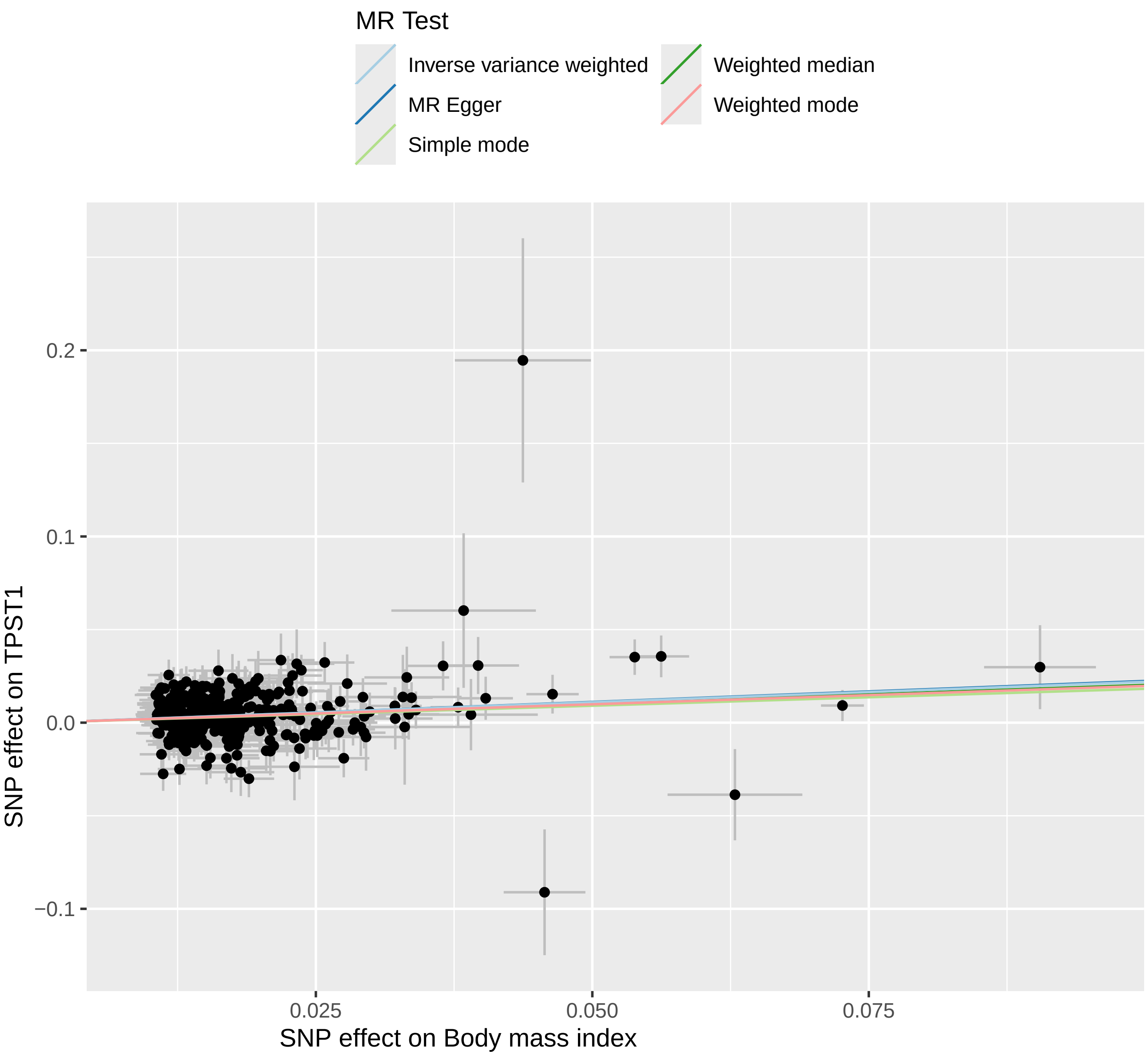
MR Test
Inverse variance weighted
MR Egger
Simple mode
Weighted median
Weighted mode
0.2
0.1
0.0
−0.1
0.025
0.050
0.075
SNP effect on Body mass index
SNP effect on TPST1

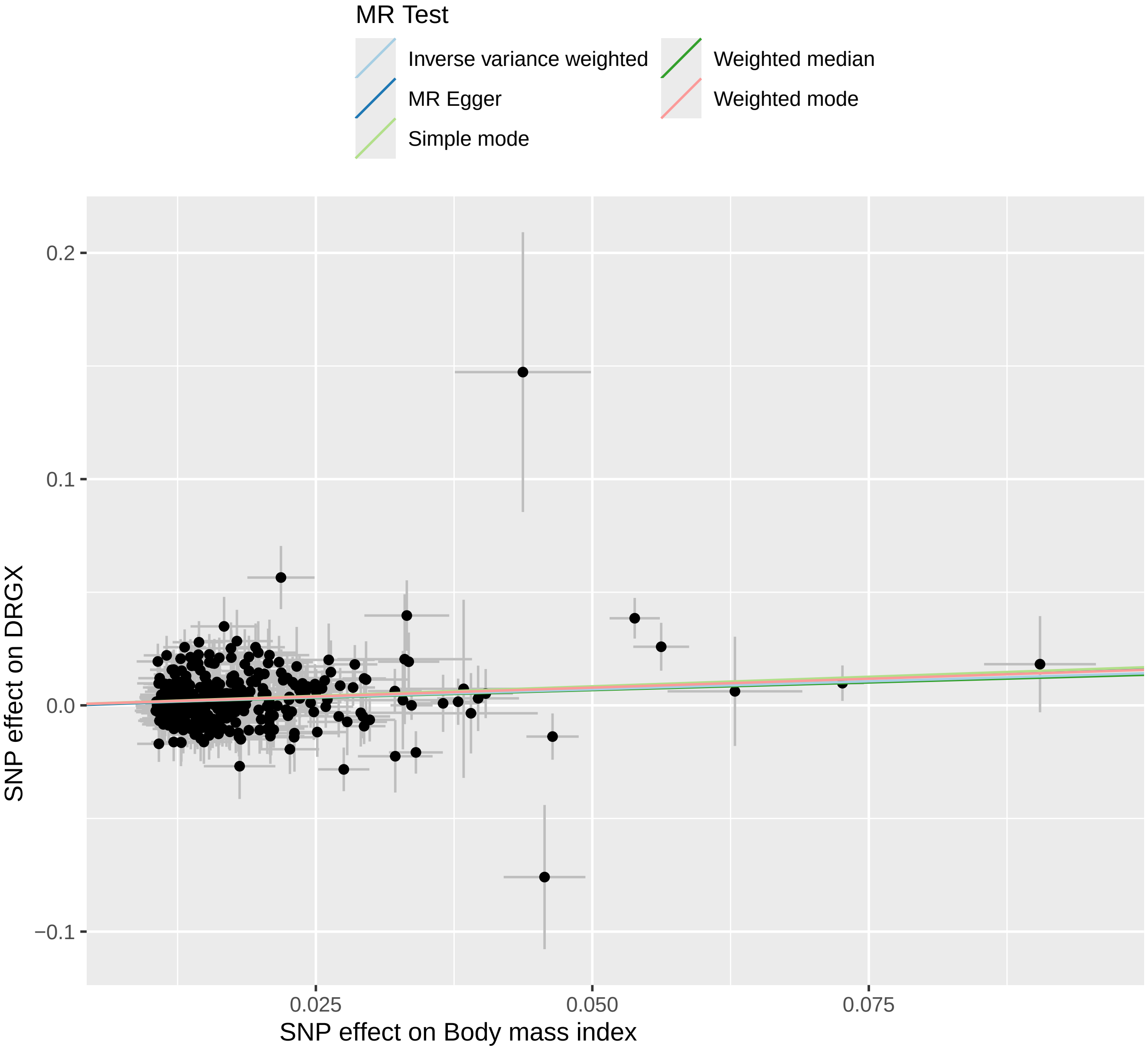
MR Test
Inverse variance weighted
MR Egger
Simple mode
Weighted median
Weighted mode
0.2
0.1
0.0
−0.1
0.025
0.050
0.075
SNP effect on DRGX
SNP effect on Body mass index

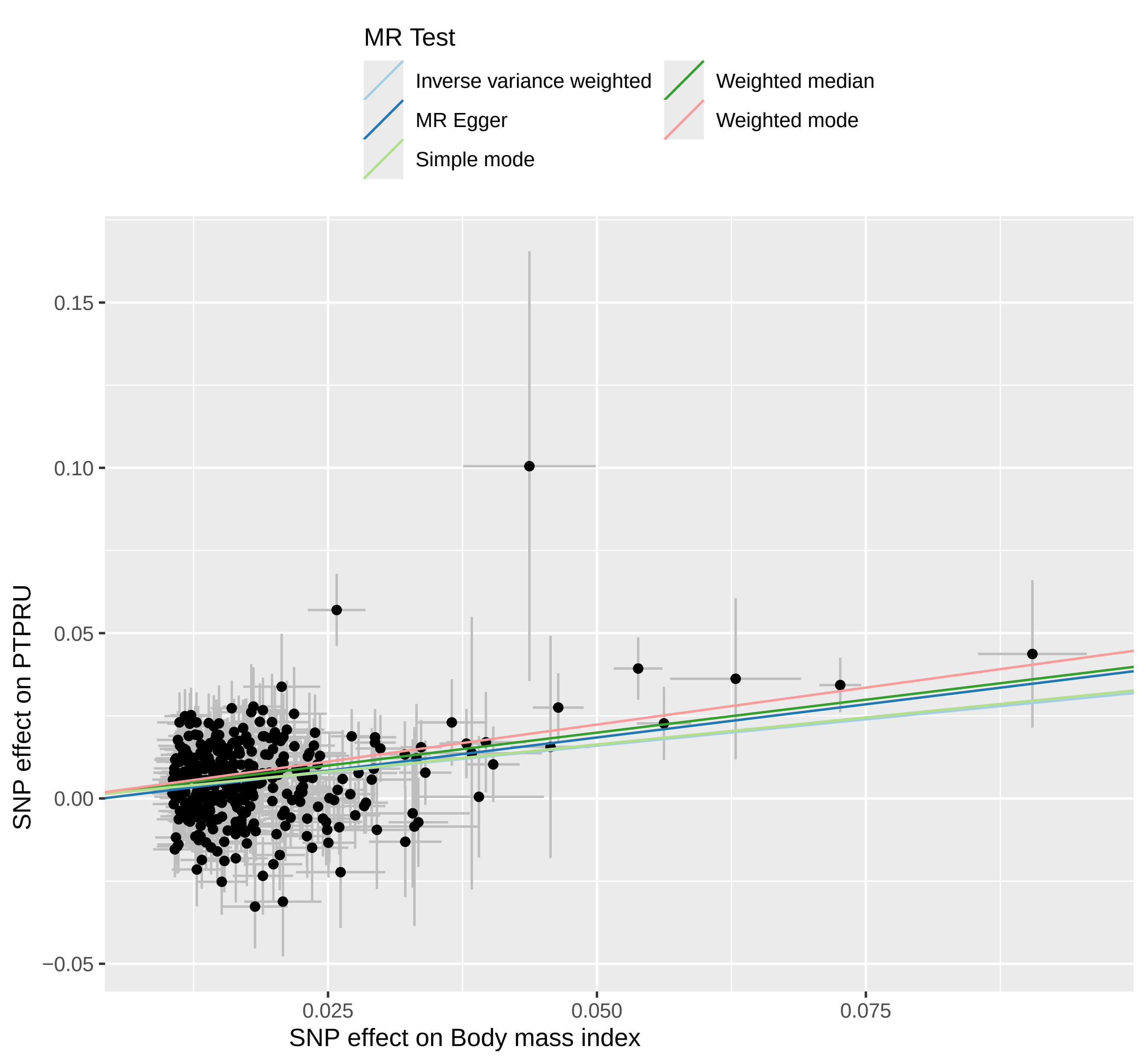
MR Test
Inverse variance weighted
MR Egger
Simple mode
Weighted median
Weighted mode
0.15
0.10
0.05
0.00
−0.05
0.025
0.050
0.075
SNP effect on Body mass index
SNP effect on PTPRU

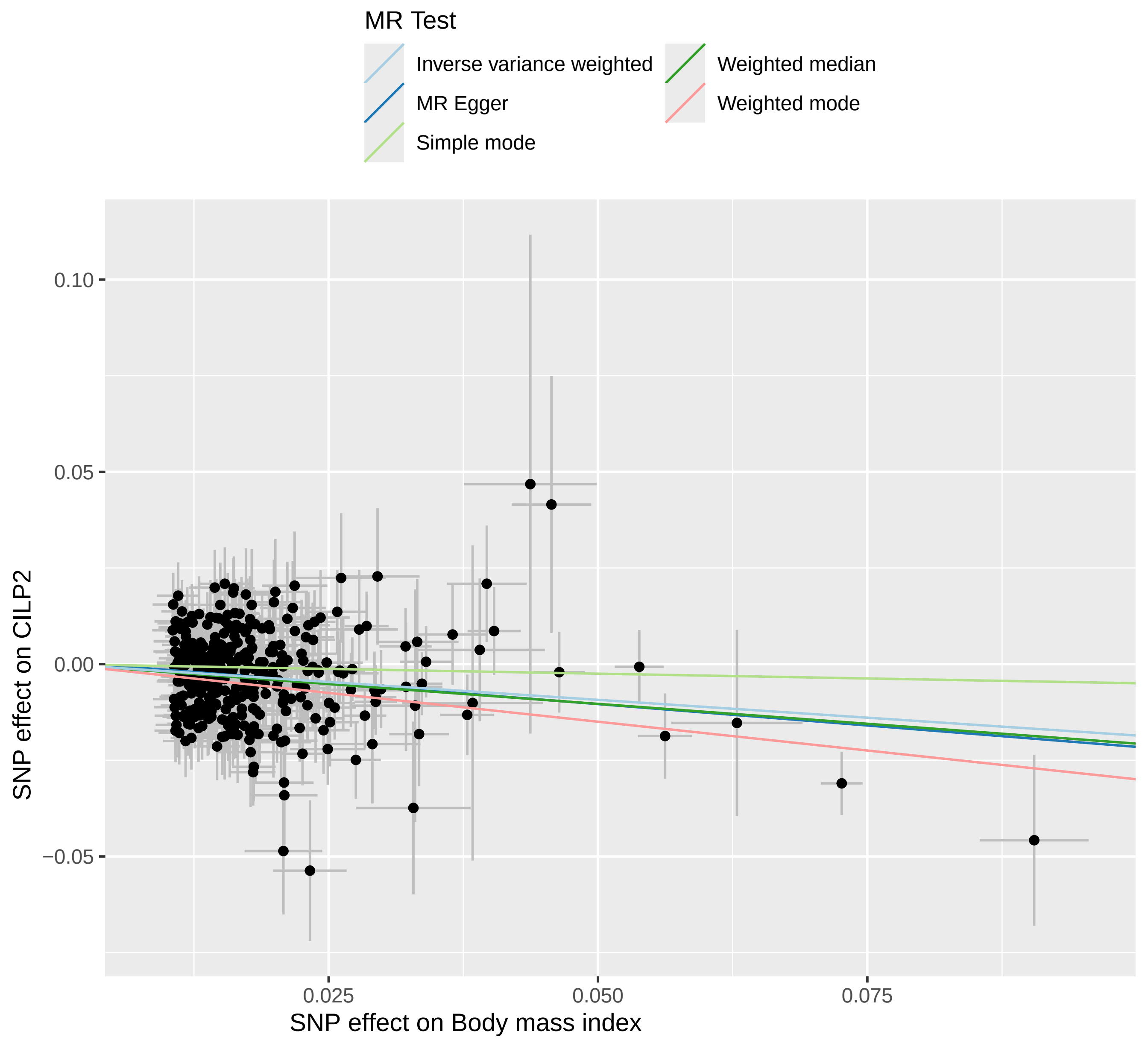
MR Test
Inverse variance weighted
MR Egger
Simple mode
Weighted median
Weighted mode
0.10
0.05
0.00
−0.05
0.025
0.050
0.075
SNP effect on CILP2
SNP effect on Body mass index

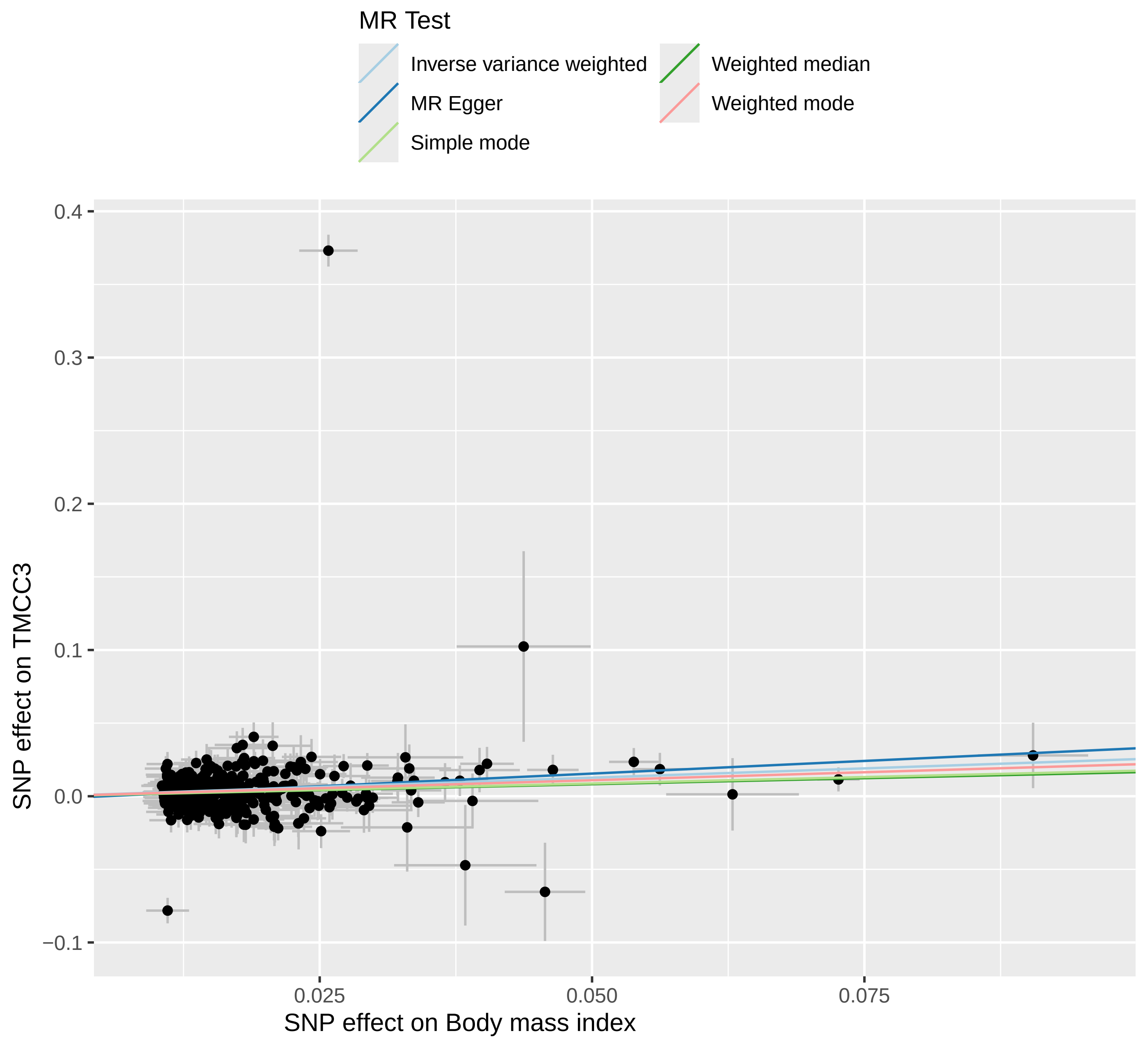
MR Test
Inverse variance weighted
MR Egger
Simple mode
Weighted median
Weighted mode
SNP effect on TMCC3
0.4
0.3
0.2
0.1
0.0
−0.1
0.025
0.050
0.075
SNP effect on Body mass index

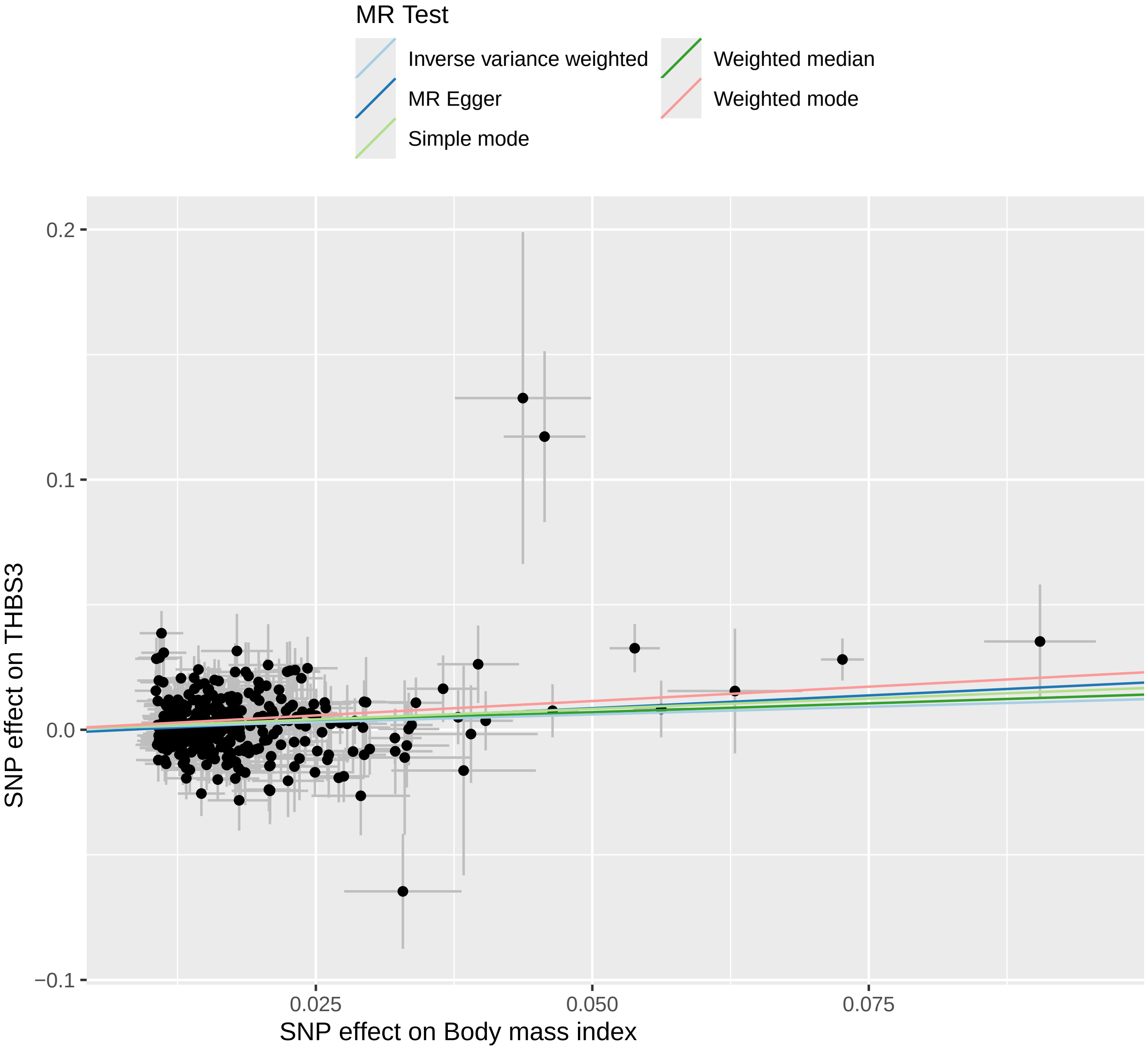

MR Test
Inverse variance weighted
MR Egger
Simple mode
Weighted median
Weighted mode
0.2
0.1
0.0
−0.1
0.025
0.050
0.075
SNP effect on THBS3
SNP effect on Body mass index

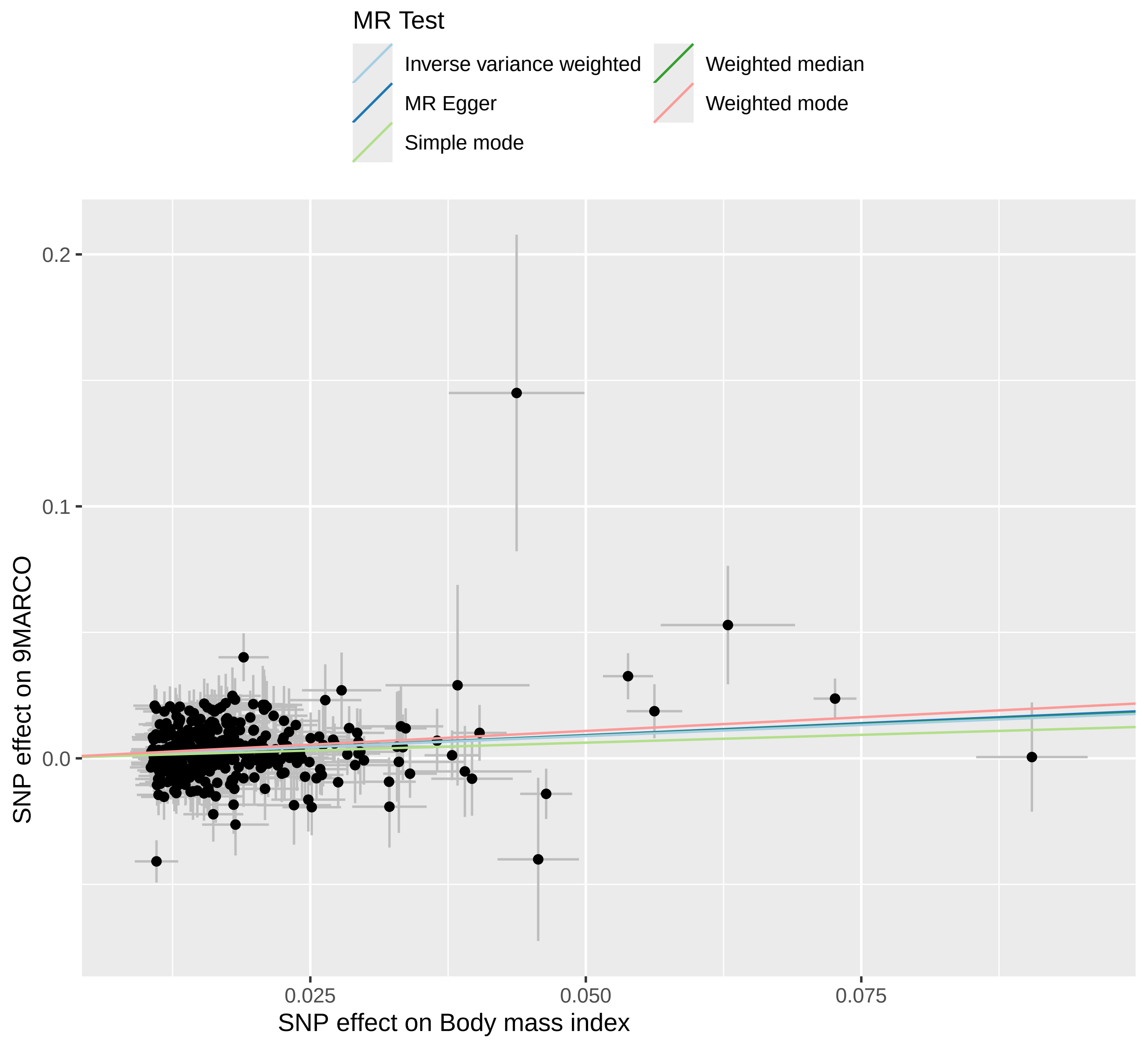
MR Test
Inverse variance weighted
MR Egger
Simple mode
Weighted median
Weighted mode
0.2
0.1
0.0
0.025
0.050
0.075
SNP effect on 9MARCO
SNP effect on Body mass index

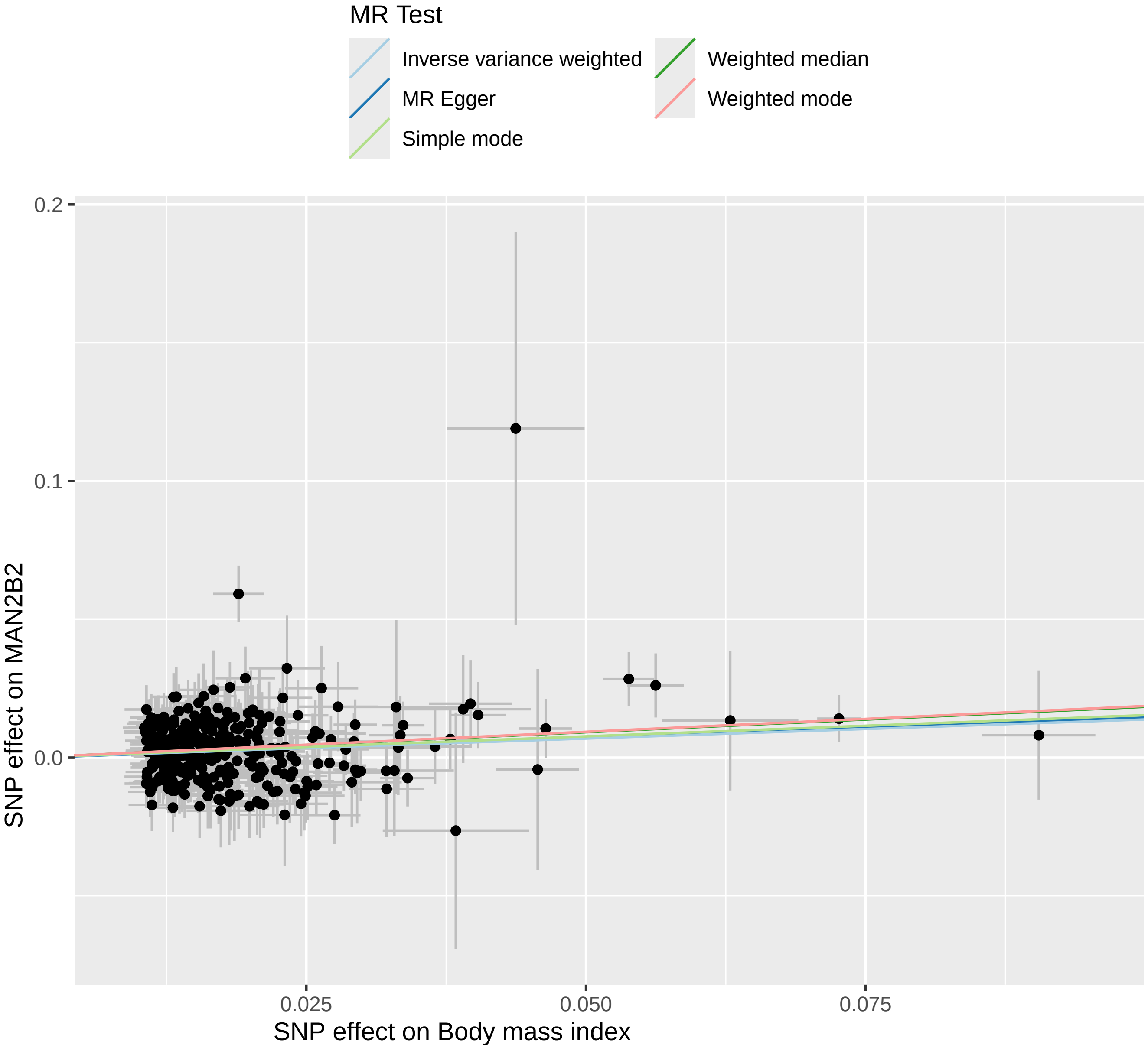
MR Test
Inverse variance weighted
MR Egger
Simple mode
Weighted median
Weighted mode
0.2
0.1
0.0
0.025
0.050
0.075
SNP effect on MAN2B2
SNP effect on Body mass index

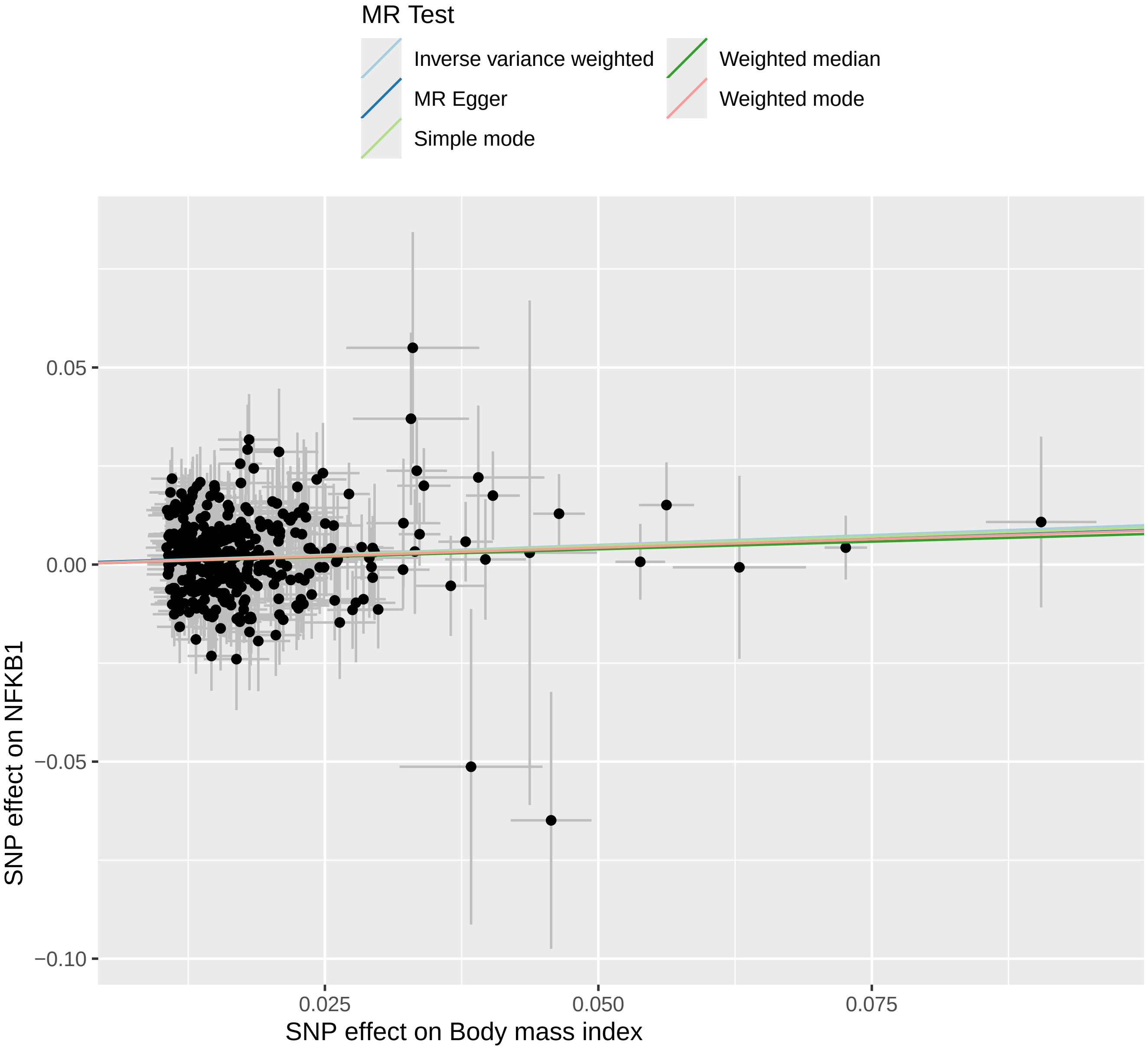
MR Test
Inverse variance weighted
MR Egger
Simple mode
Weighted median
Weighted mode
0.05
0.00
−0.05
−0.10
0.025
0.050
0.075
SNP effect on NFKB1
SNP effect on Body mass index

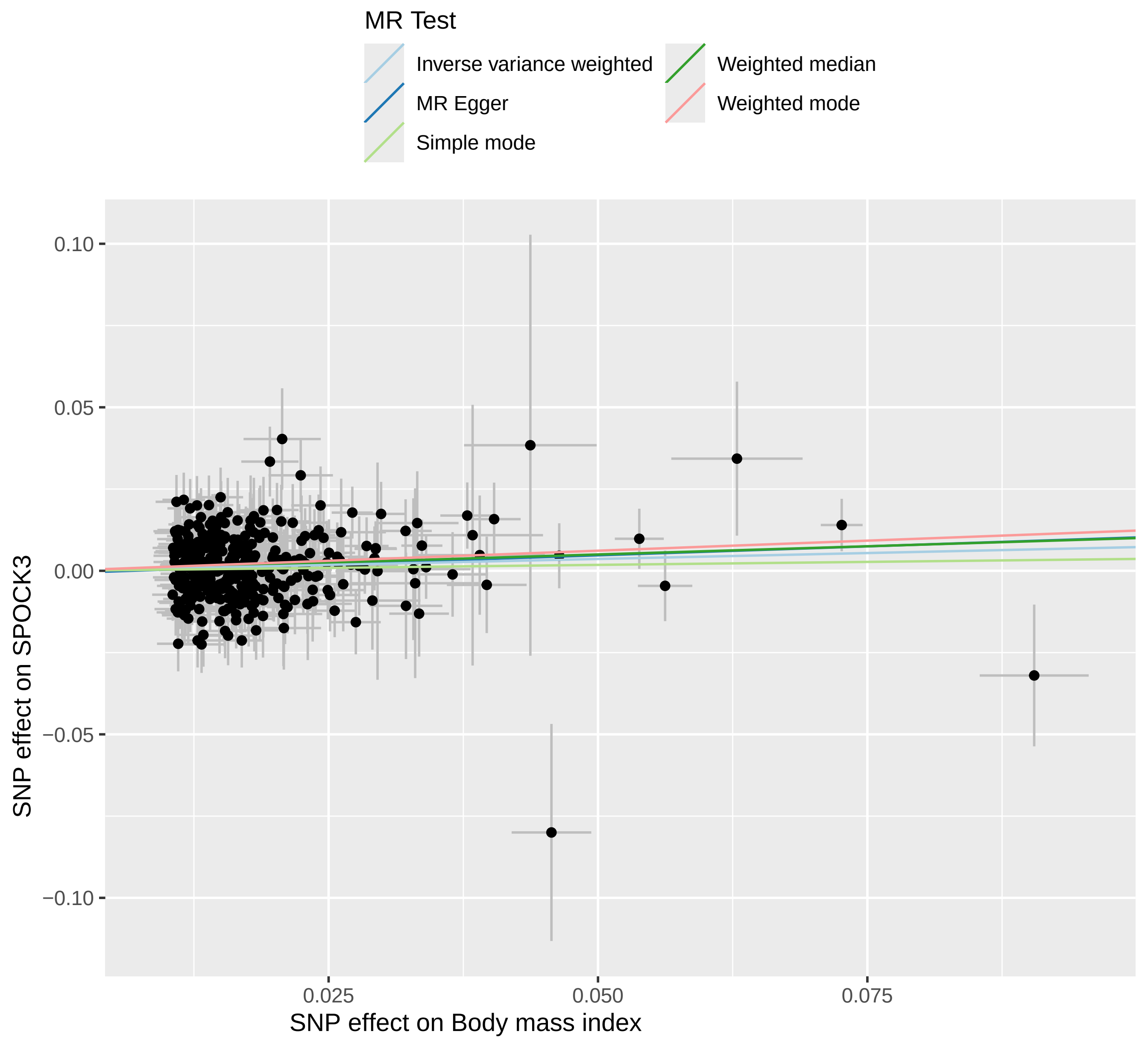
MR Test
Inverse variance weighted
MR Egger
Simple mode
Weighted median
Weighted mode
0.10
0.05
0.00
−0.05
−0.10
0.025
0.050
0.075
SNP effect on SPOCK3
SNP effect on Body mass index
