# Supplementary material for: Mediating Mendelian randomization in the proteome identified potential drug targets for obesity-related allergic asthma
Source: Hereditas. 2025 Feb 1;162:12. doi: 10.1186/s41065-025-00376-w (PMC11786417; doi:10.1186/s41065-025-00376-w)
Supplement: Supplementary file 1 — Supplementary Material 1: Supplementary Materials 1. The MR results of BMI and allergic asthma. Supplementary Materials 2. The MR results of plasma proteins and allergic asthma. Supplementary Materials 3. The MR results of BMI and plasma proteins. Supplementary Materials 4. The MR results of mediation factor plasma proteins and allergic asthma. Supplementary Materials 5. The results of drug-targeted MR [file 41065_2025_376_MOESM1_ESM.zip › Supplementary Materials/S4 The MR results of mediation factor plasma proteins and allergic asthma/S4 plasma proteins-Allergic asthma.funnel_plot.pdf]

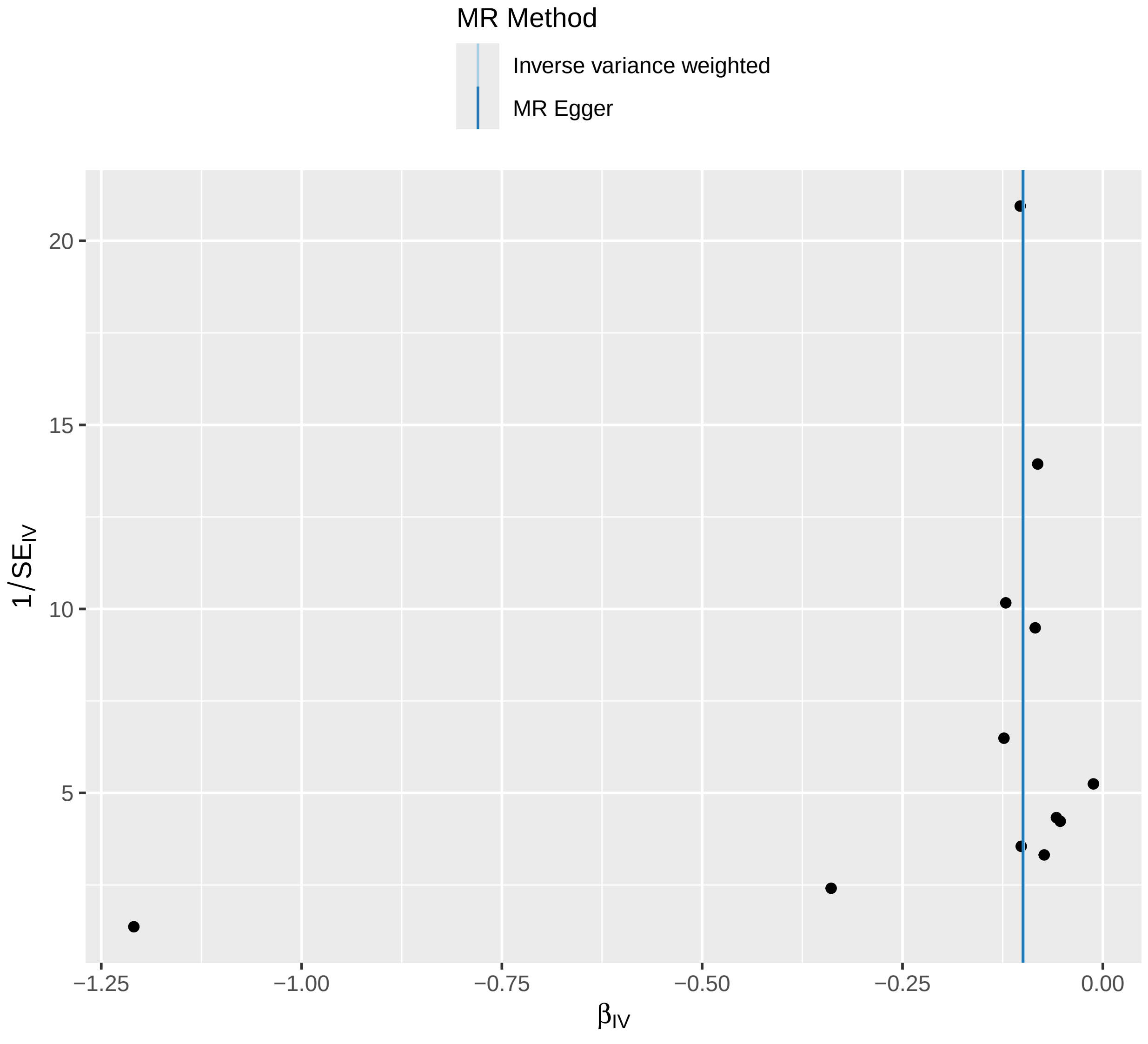
MR Method
Inverse variance weighted
MR Egger
20
15
10
5
1/SE_IV
−1.25
−1.00
−0.75
−0.50
−0.25
0.00
β_IV

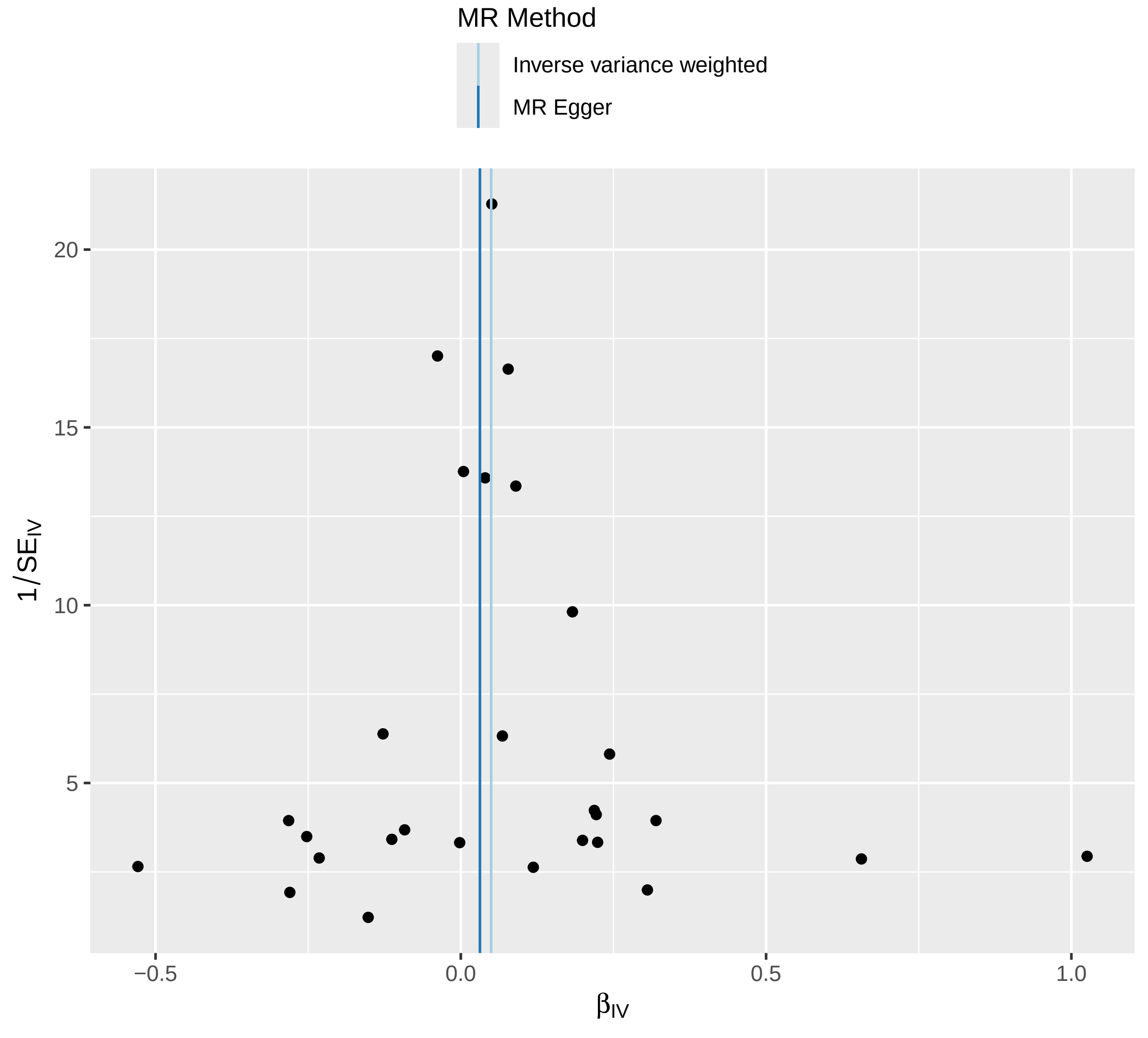
MR Method
Inverse variance weighted
MR Egger
1/SE_IV
β_IV
20
15
10
5
−0.5
0.0
0.5
1.0

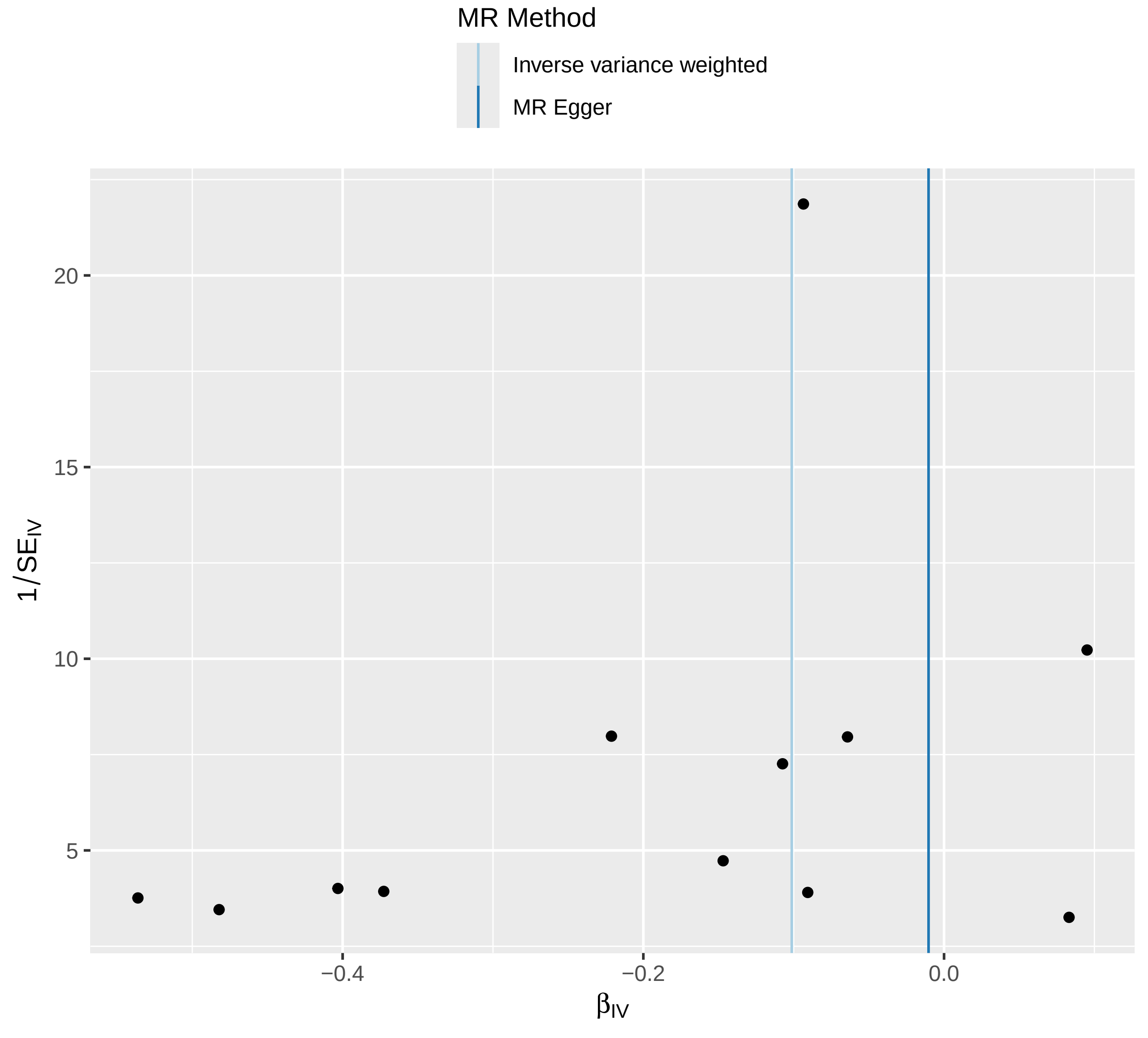

MR Method
Inverse variance weighted
MR Egger
20
15
10
5
−0.4
−0.2
0.0
$1/SE_{IV}$
β_{IV}

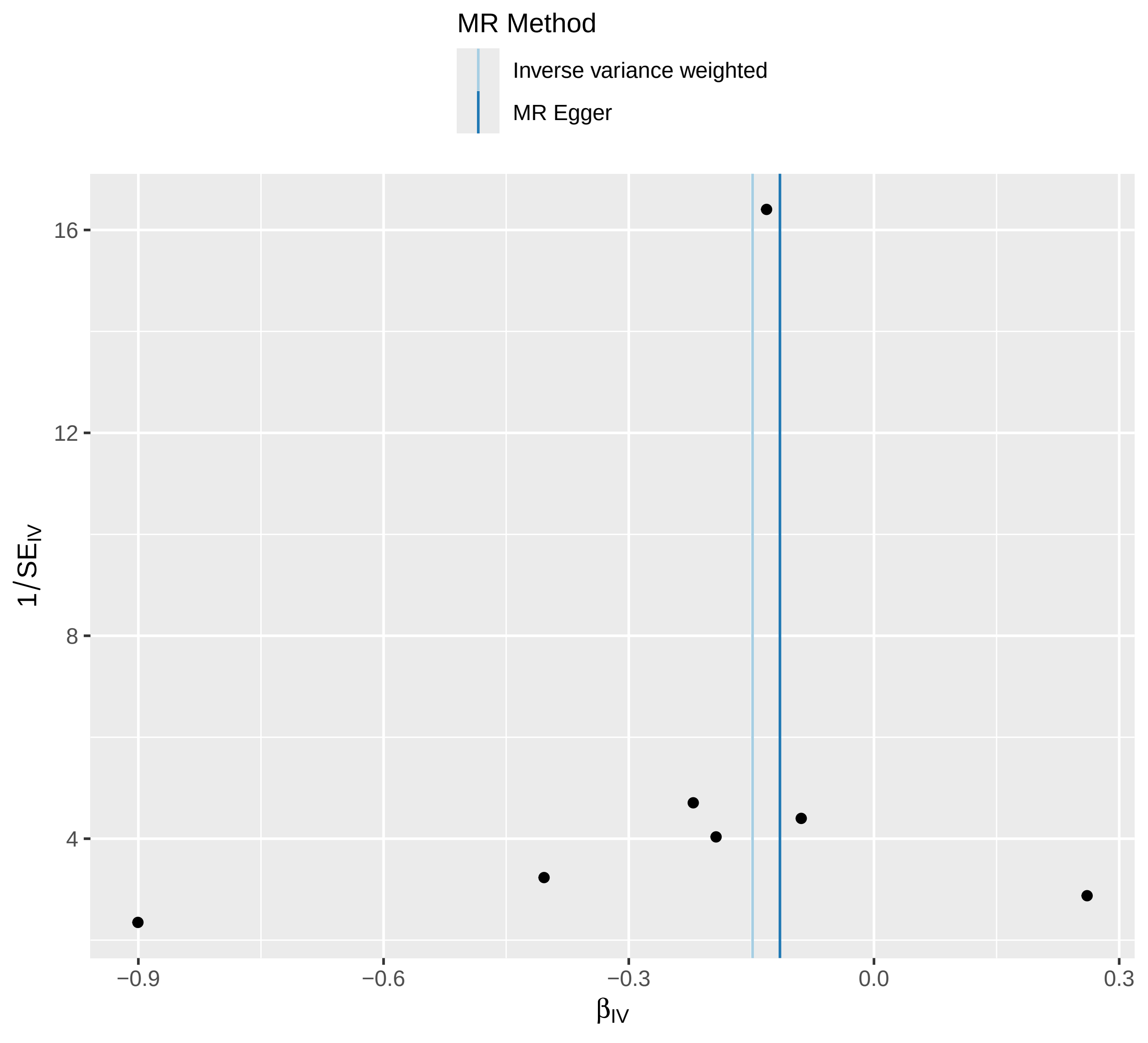

MR Method
Inverse variance weighted
MR Egger
16
12
8
4
1/SE_IV
−0.9
−0.6
−0.3
0.0
0.3
β_IV

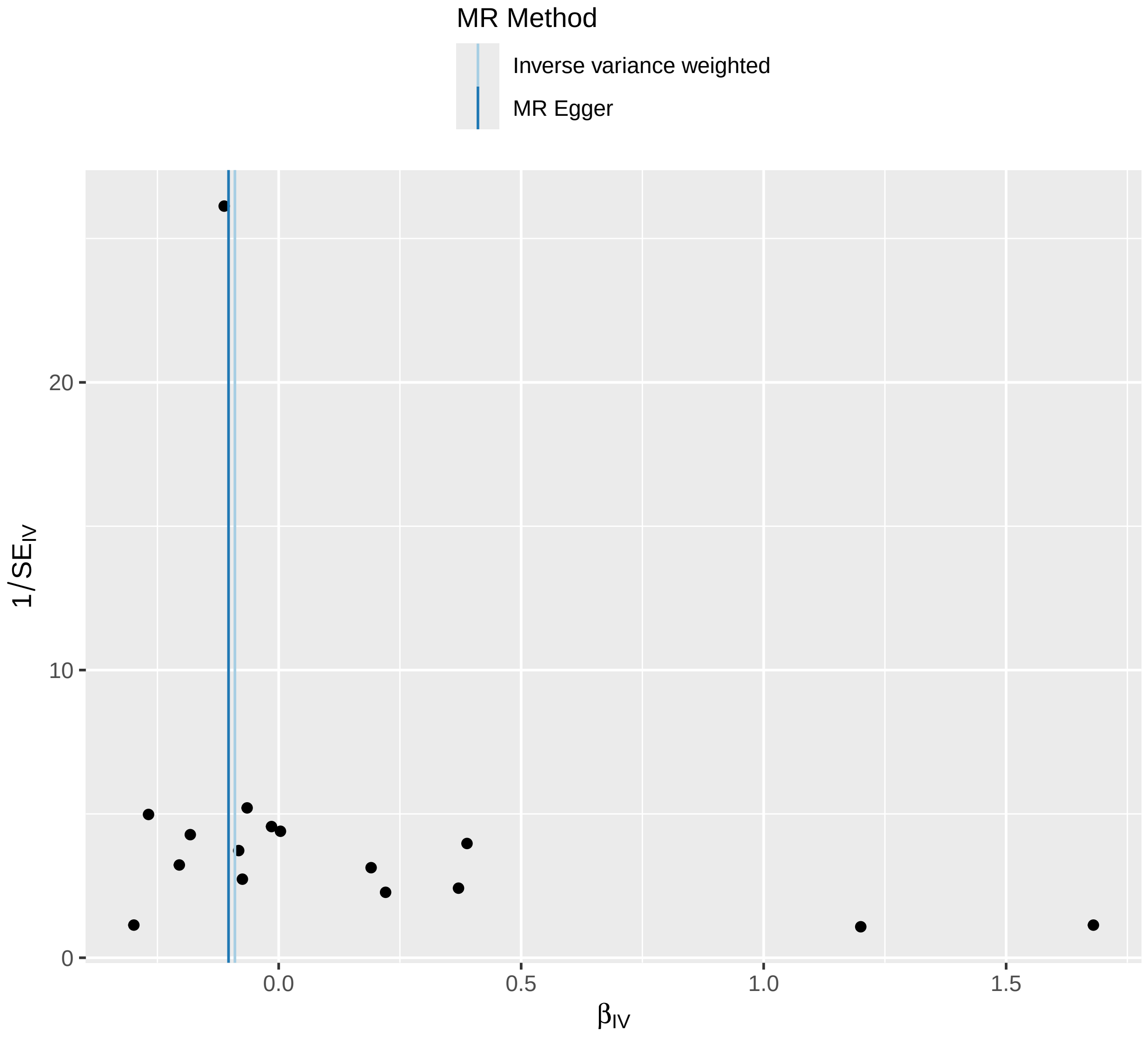
MR Method
Inverse variance weighted
MR Egger
20
10
0
$1/SE_{IV}$
0.0
0.5
1.0
1.5
β_{IV}

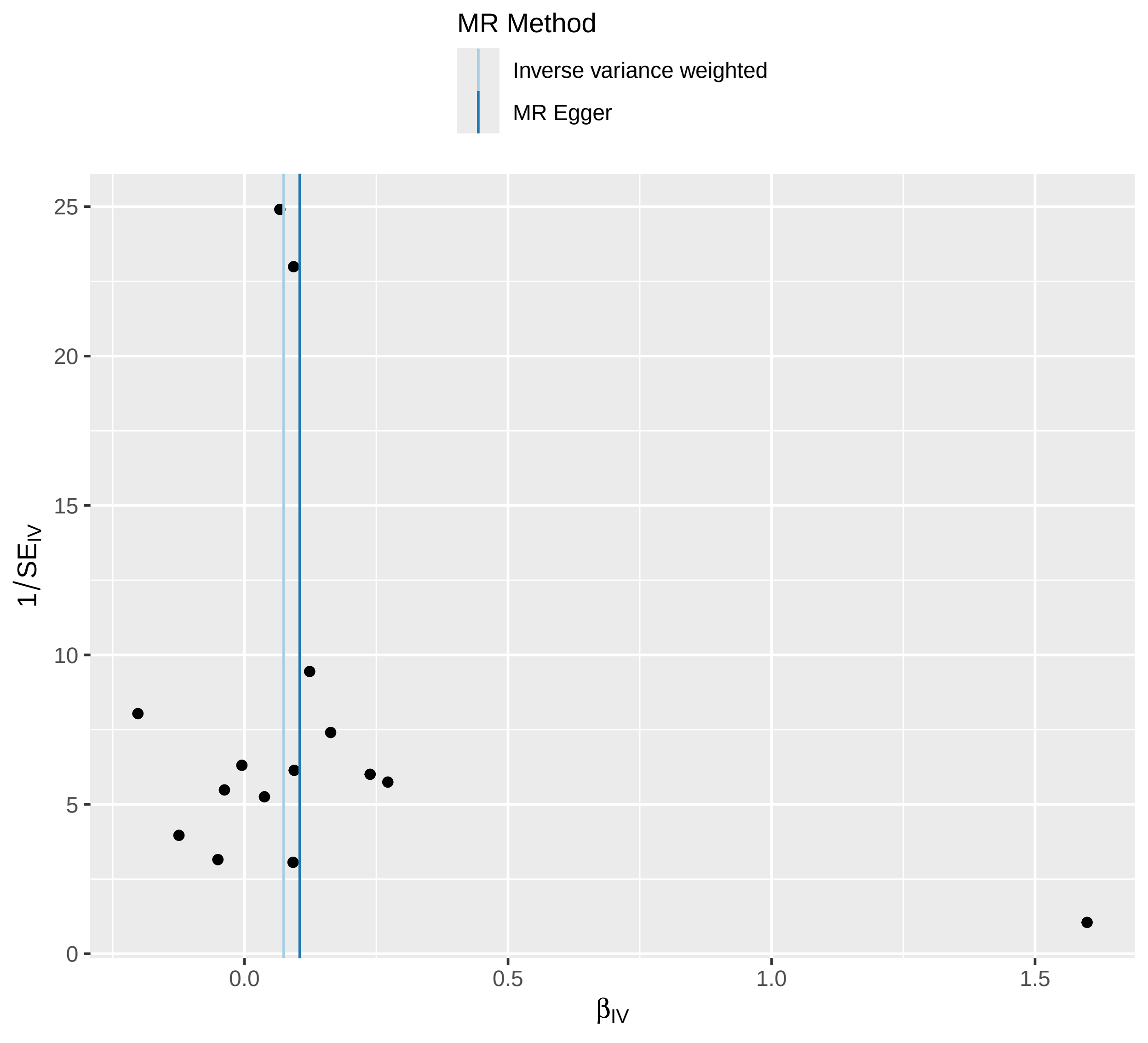
MR Method
Inverse variance weighted
MR Egger
25
20
15
10
5
0
1/SE_IV
0.0
0.5
1.0
1.5
β_IV

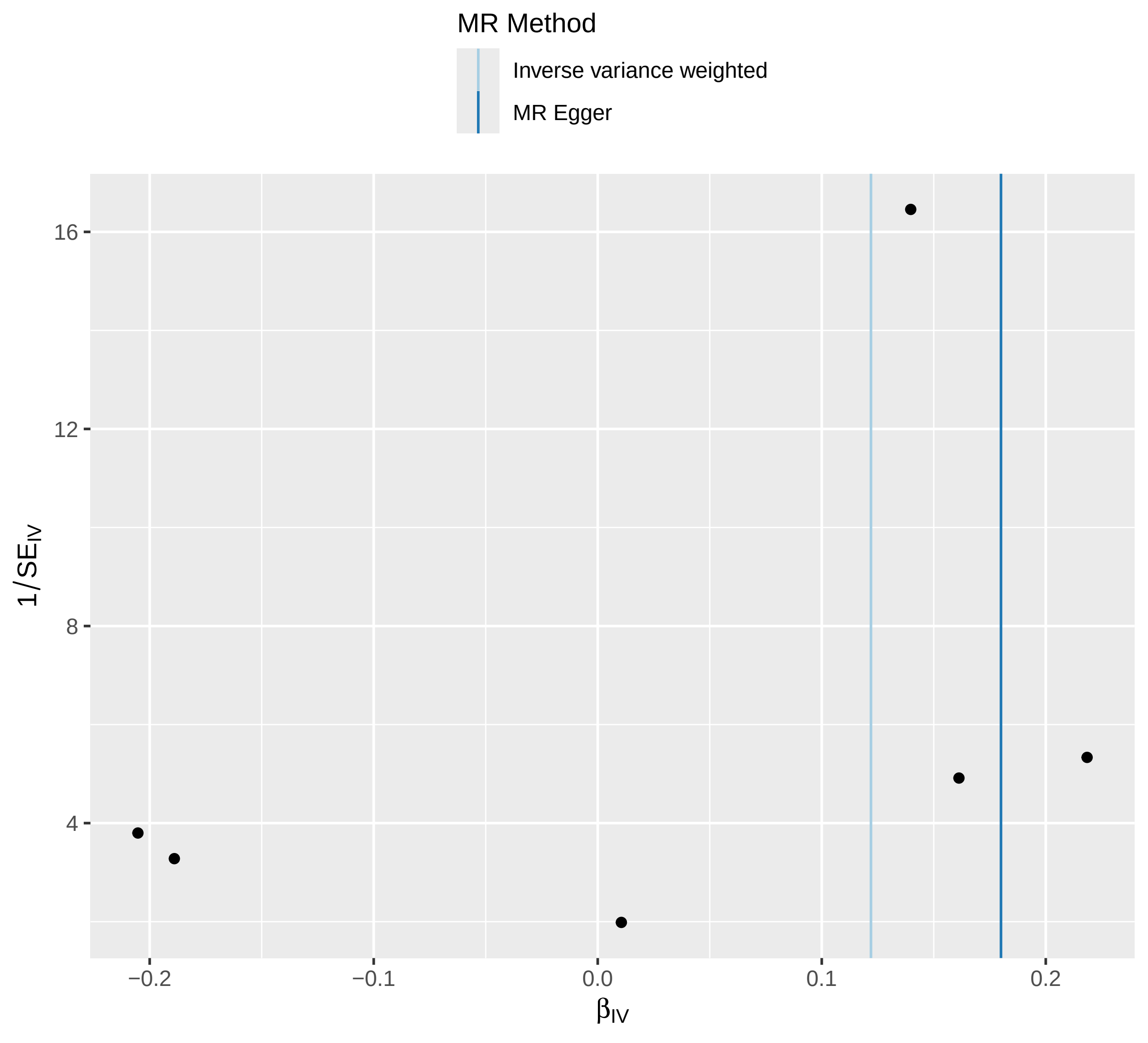
MR Method
Inverse variance weighted
MR Egger
16
12
8
4
1/SE_IV
−0.2
−0.1
0.0
0.1
0.2
β_IV

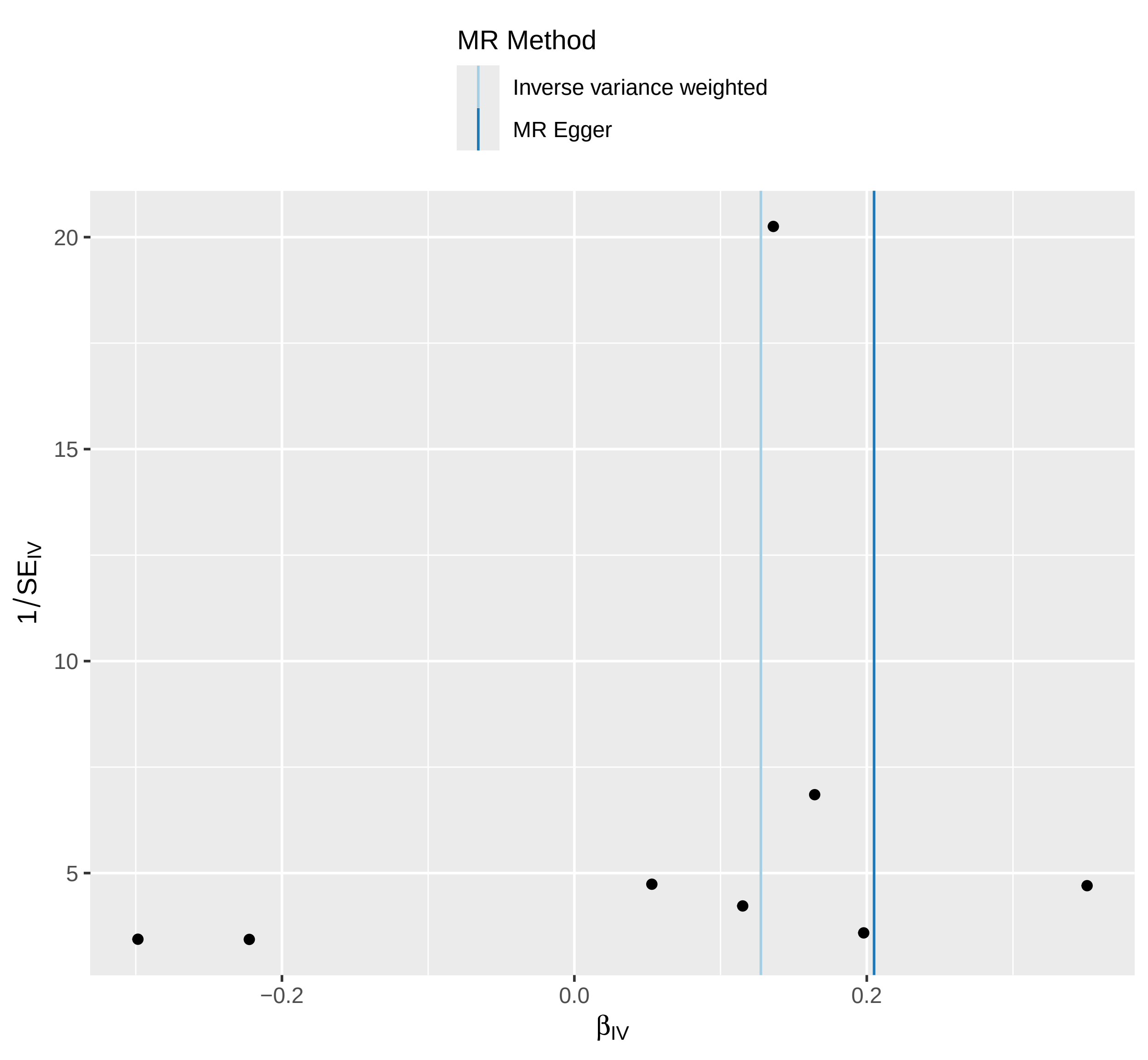
MR Method
Inverse variance weighted
MR Egger
20
15
10
5
1/SE_IV
−0.2
0.0
0.2
β_IV

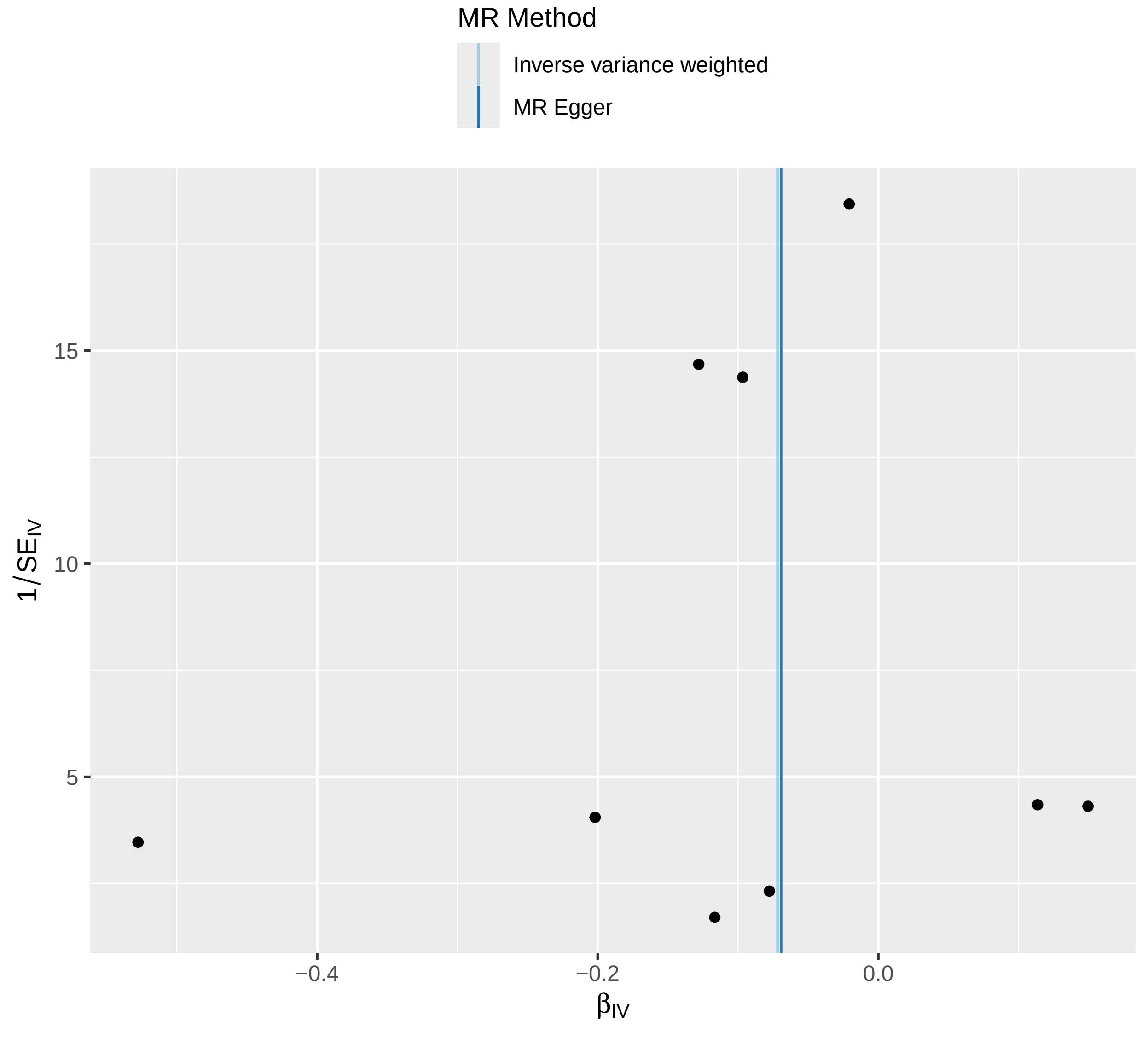
MR Method
Inverse variance weighted
MR Egger
15
10
5
1/SE$_{IV}$
−0.4
−0.2
0.0
β$_{IV}$

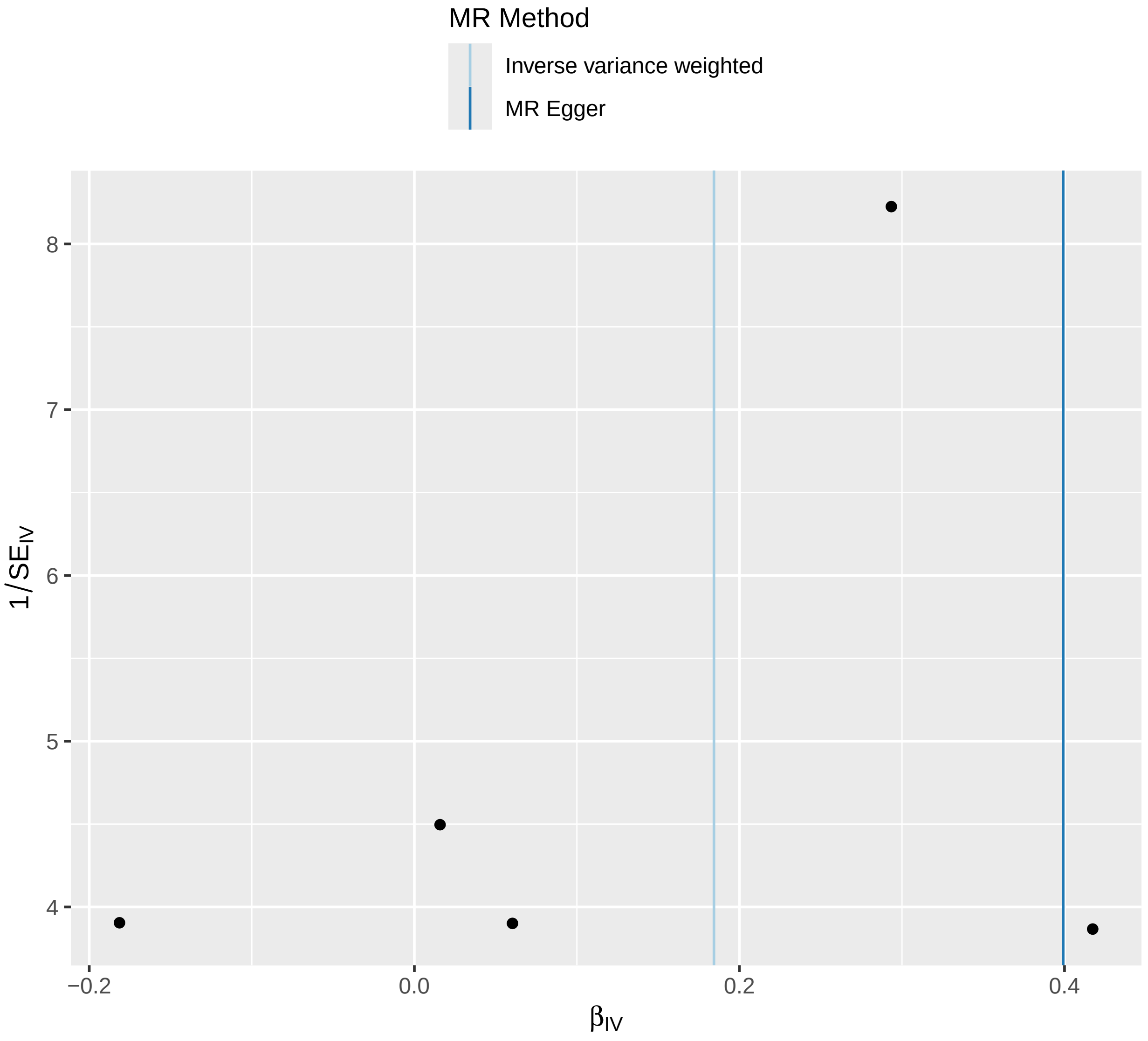

MR Method
Inverse variance weighted
MR Egger
8
7
6
5
4
1/SE_IV
−0.2
0.0
0.2
0.4
β_IV

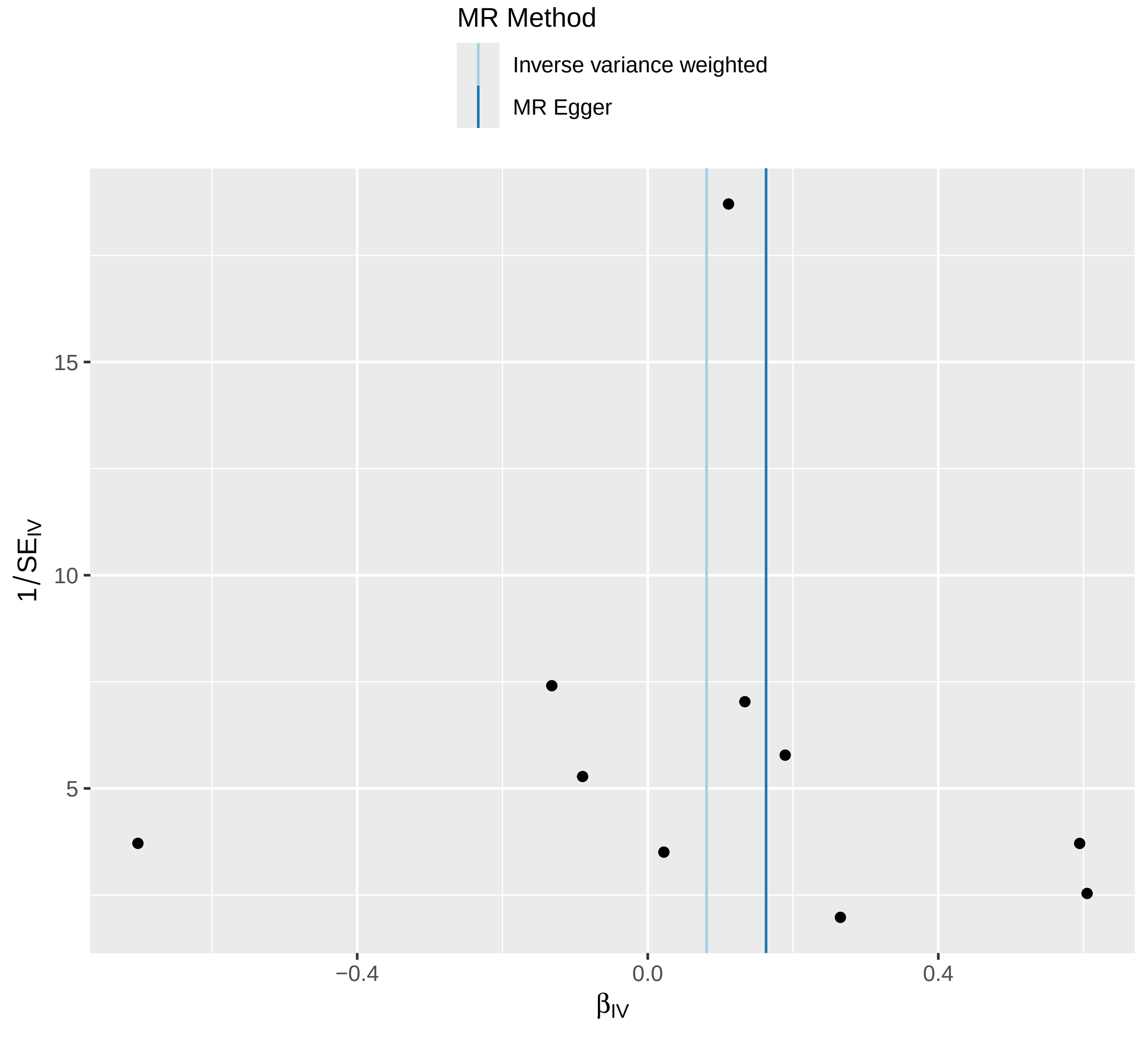

MR Method
Inverse variance weighted
MR Egger
15
10
5
−0.4
0.0
0.4
$1/SE_{IV}$
β_{IV}

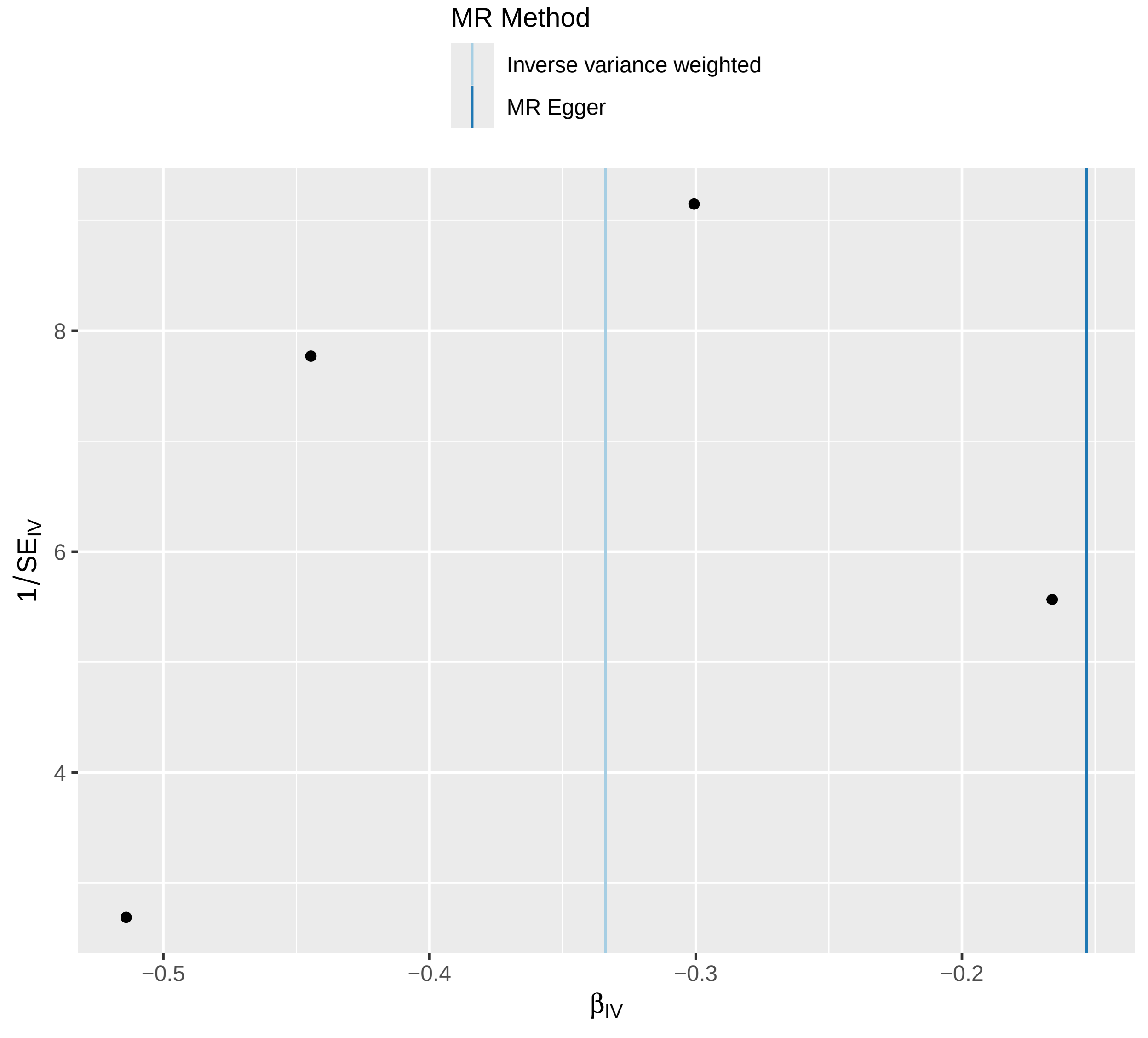

MR Method
Inverse variance weighted
MR Egger
8
6
4
1/SE$_{IV}$
−0.5
−0.4
−0.3
−0.2
β_{IV}

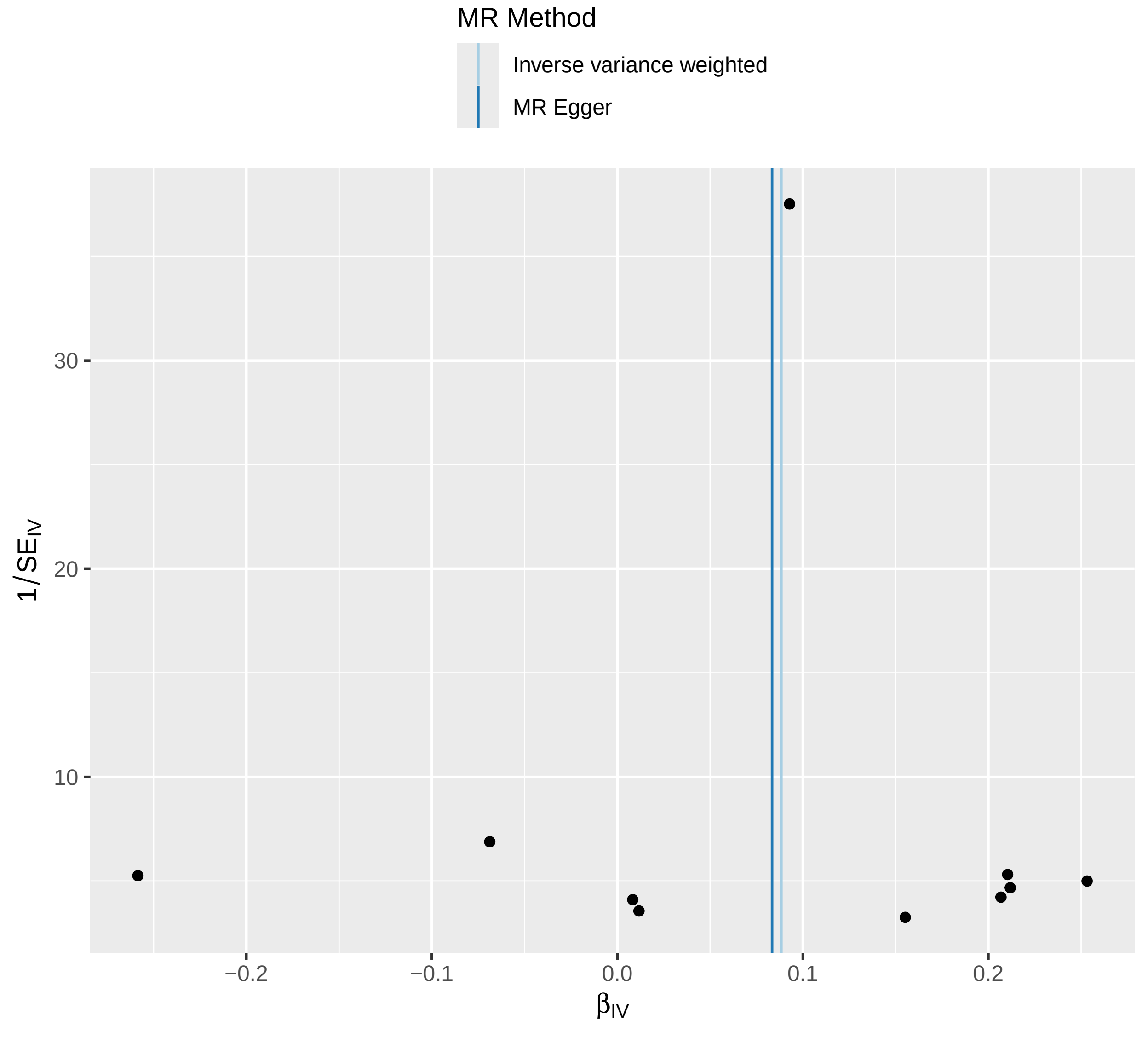
MR Method
Inverse variance weighted
MR Egger
30
20
10
1/SE$_{IV}$
−0.2
−0.1
0.0
0.1
0.2
β$_{IV}$

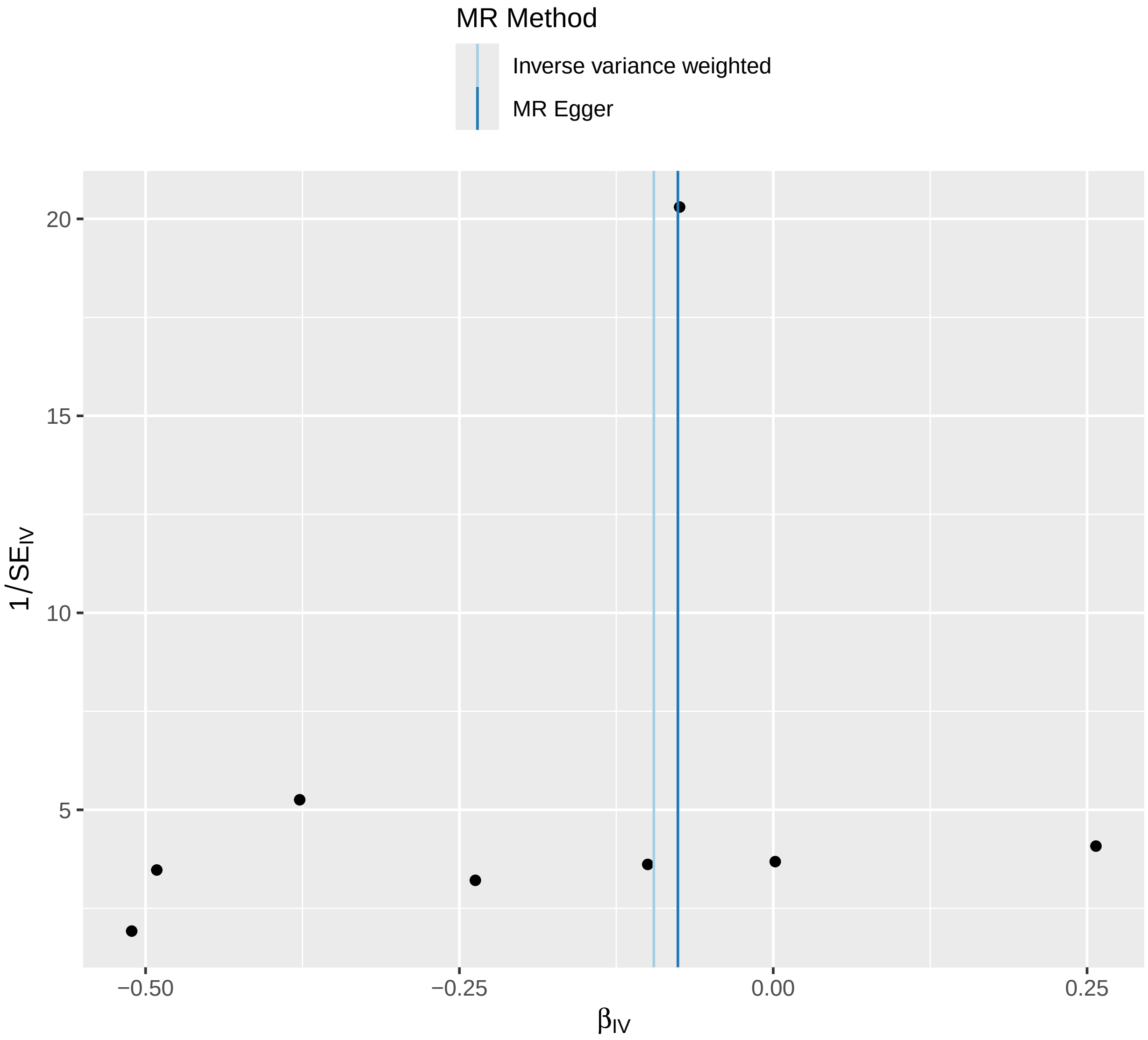
MR Method
Inverse variance weighted
MR Egger
20
15
10
5
1/SE_IV
−0.50
−0.25
0.00
0.25
β_IV

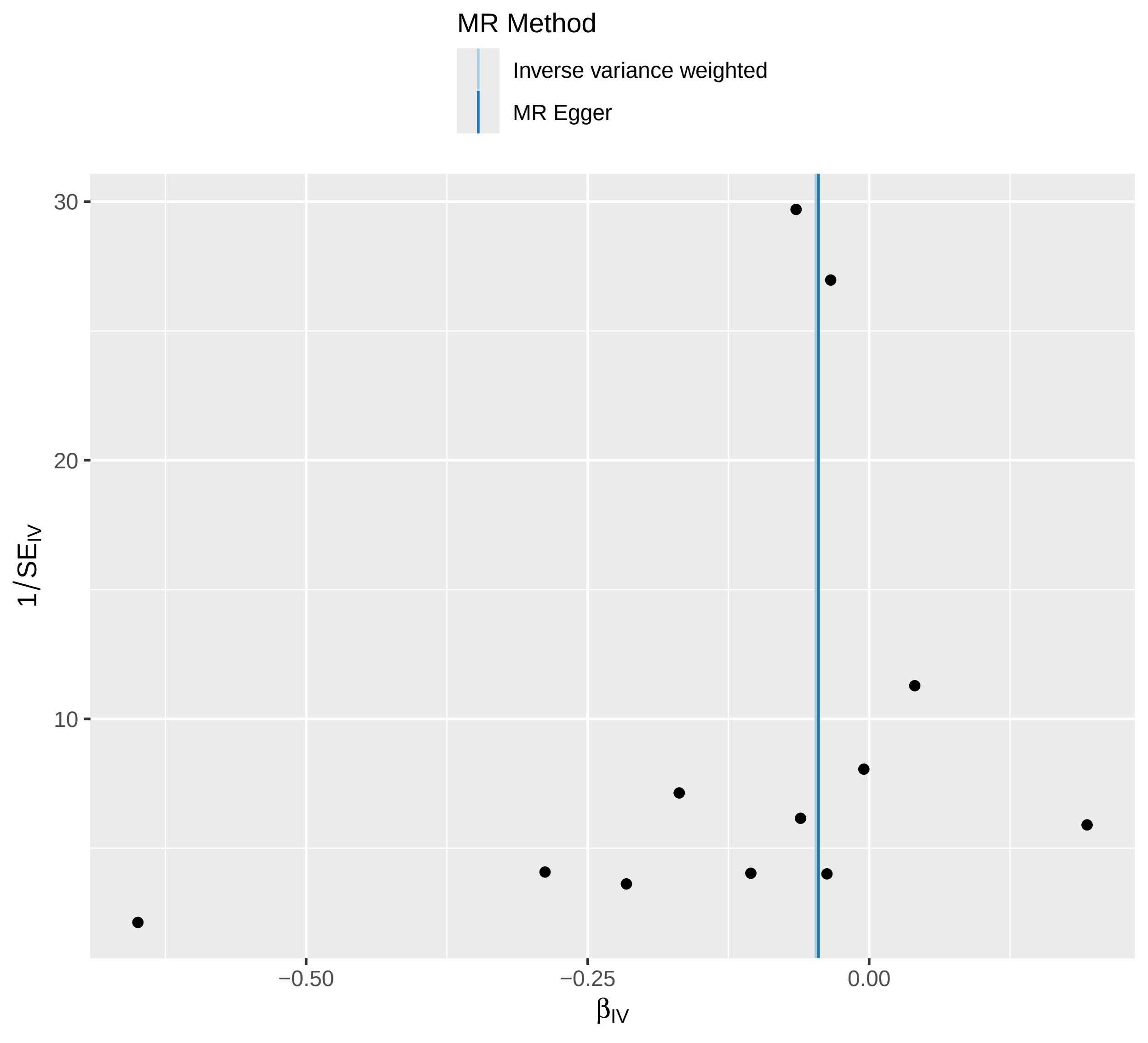
MR Method
Inverse variance weighted
MR Egger
30
20
10
1/SE$_{IV}$
−0.50
−0.25
0.00
β_{IV}

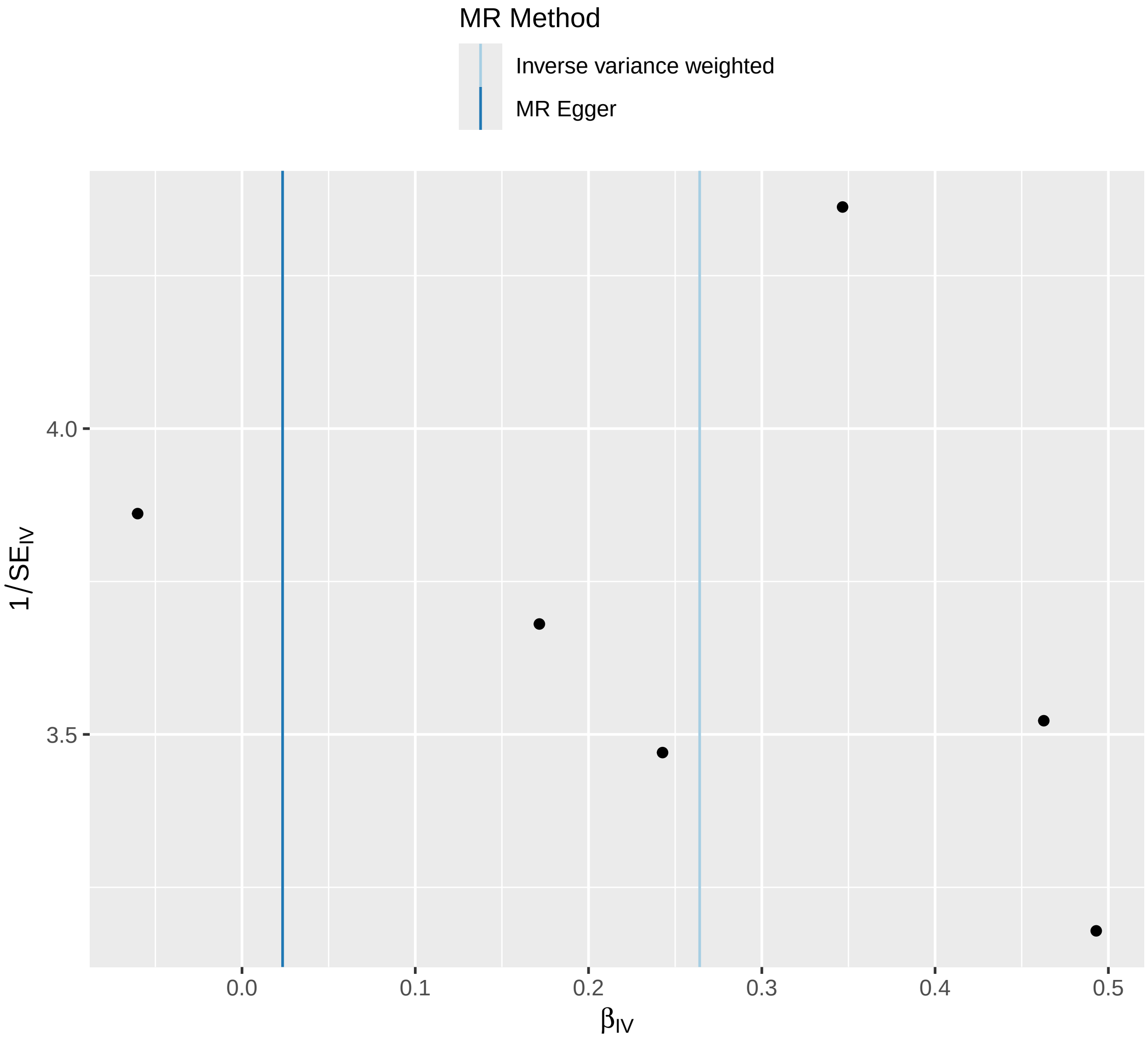
MR Method
Inverse variance weighted
MR Egger
4.0
3.5
$1/SE_{IV}$
0.0
0.1
0.2
0.3
0.4
0.5
β_{IV}

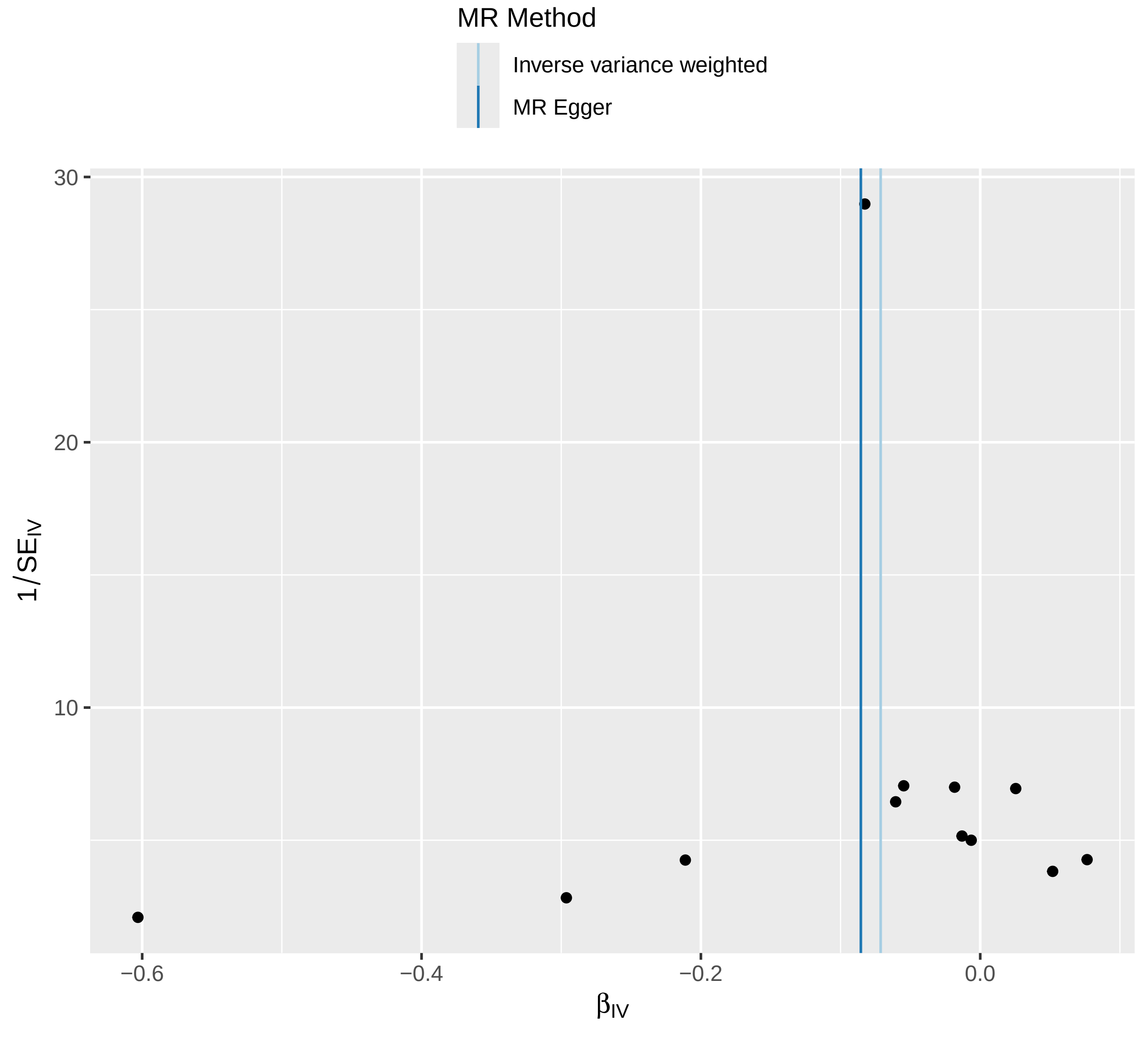
MR Method
Inverse variance weighted
MR Egger
30
20
10
1/SE_IV
−0.6
−0.4
−0.2
0.0
β_IV

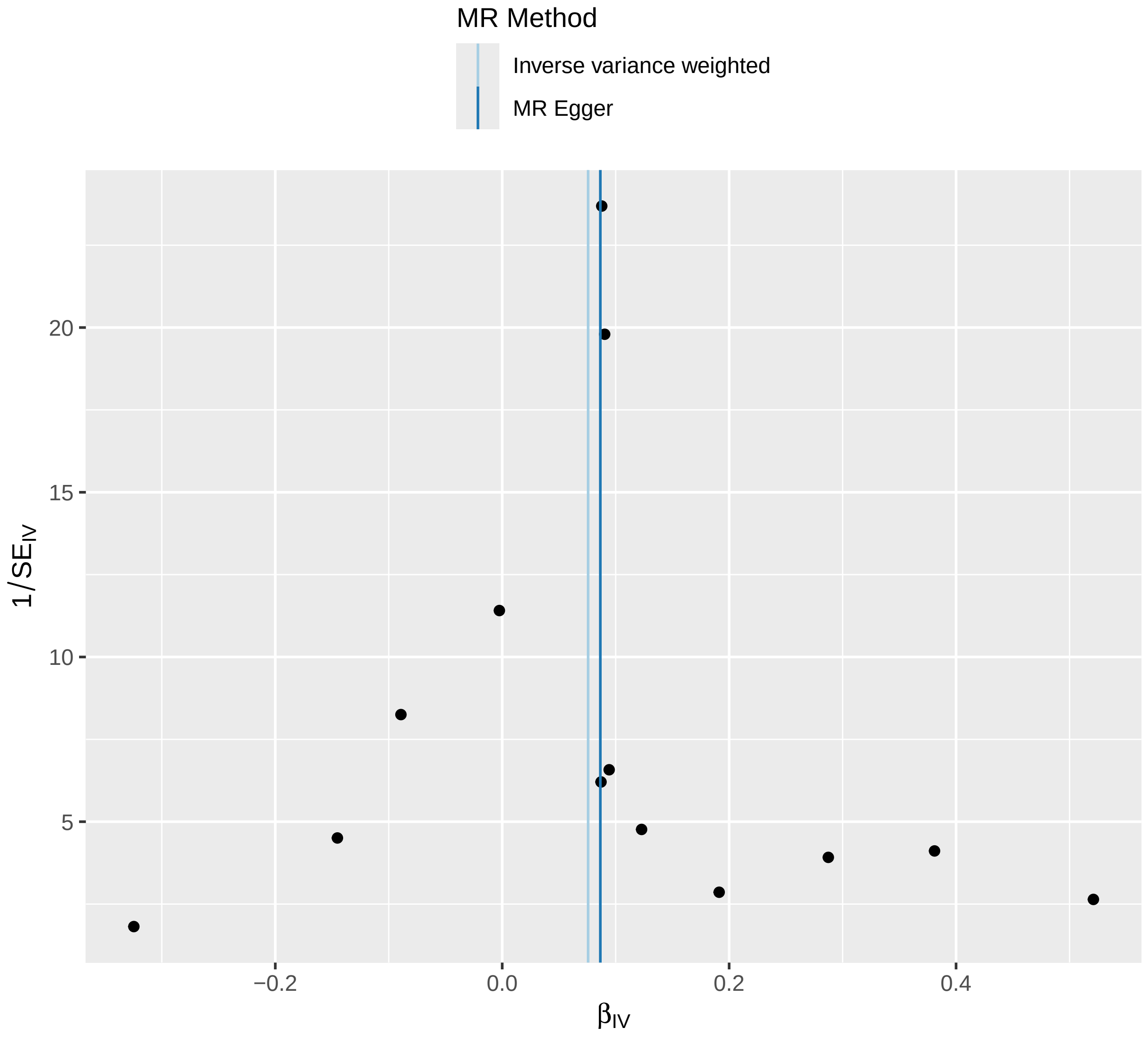
MR Method
Inverse variance weighted
MR Egger
20
15
10
5
$1/SE_{IV}$
−0.2
0.0
0.2
0.4
β_{IV}

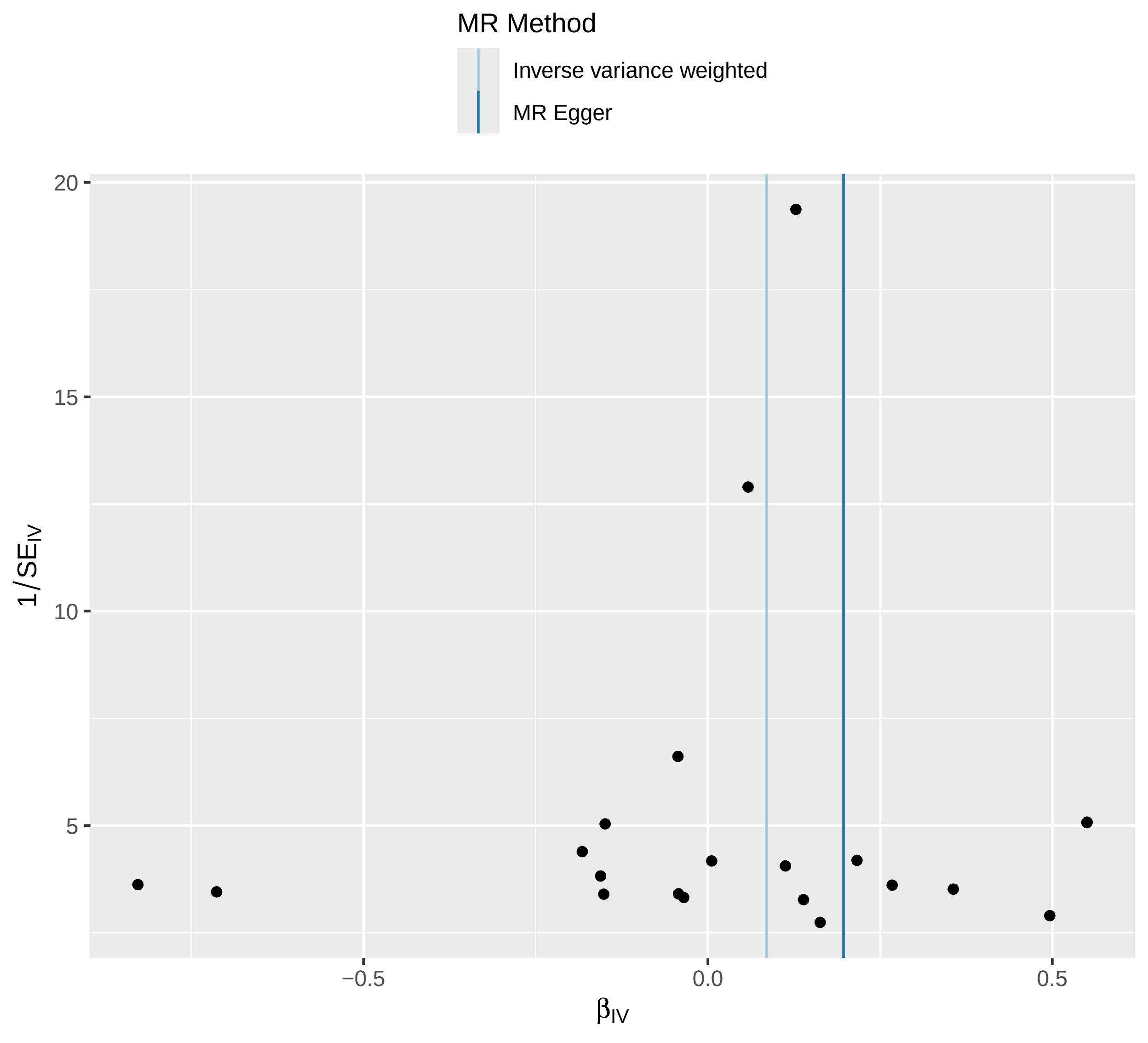

MR Method
Inverse variance weighted
MR Egger
20
15
10
5
1/SE_IV
−0.5
0.0
0.5
β_IV

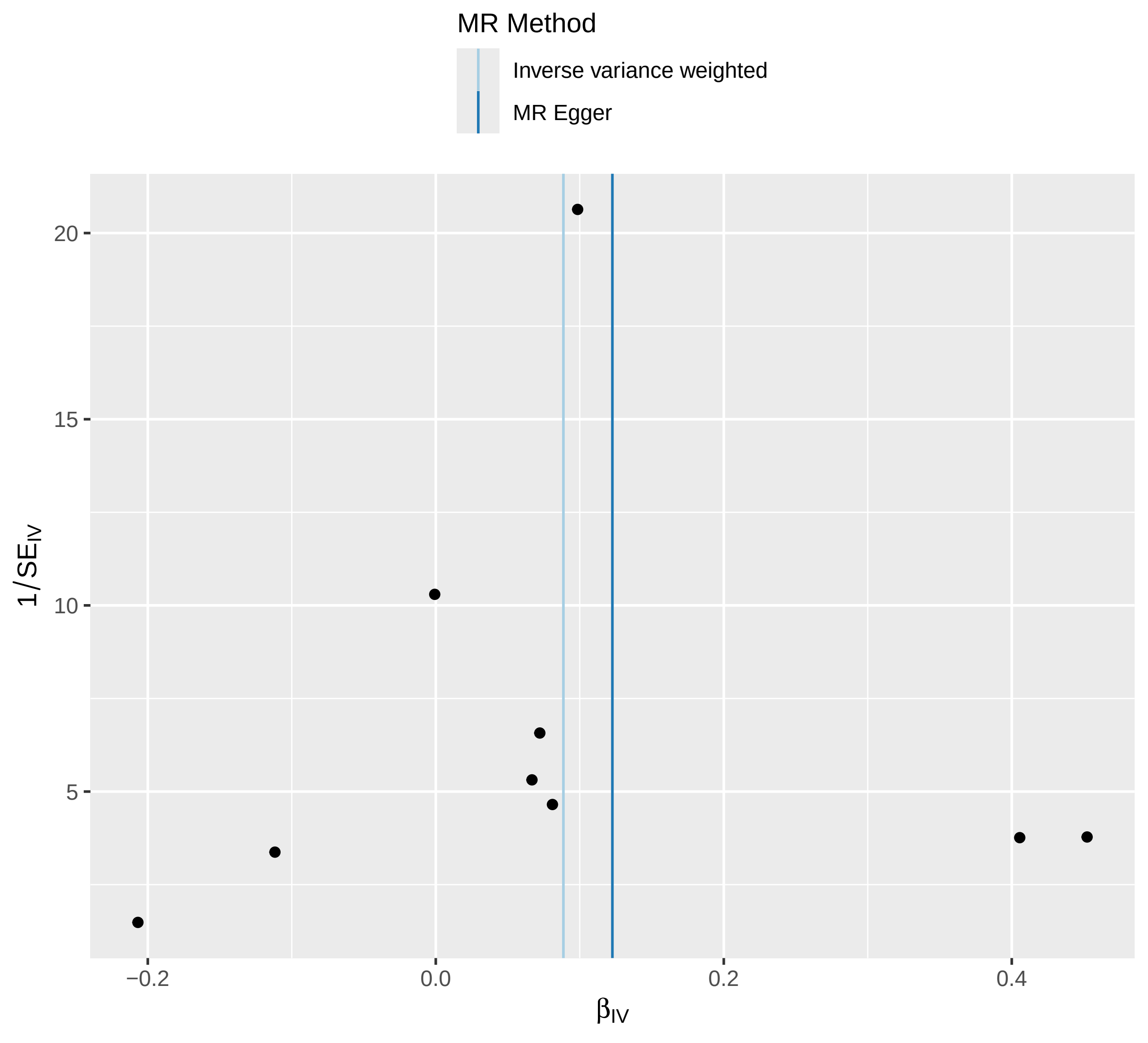
MR Method
Inverse variance weighted
MR Egger
20
15
10
5
1/SE_IV
−0.2
0.0
0.2
0.4
β_IV

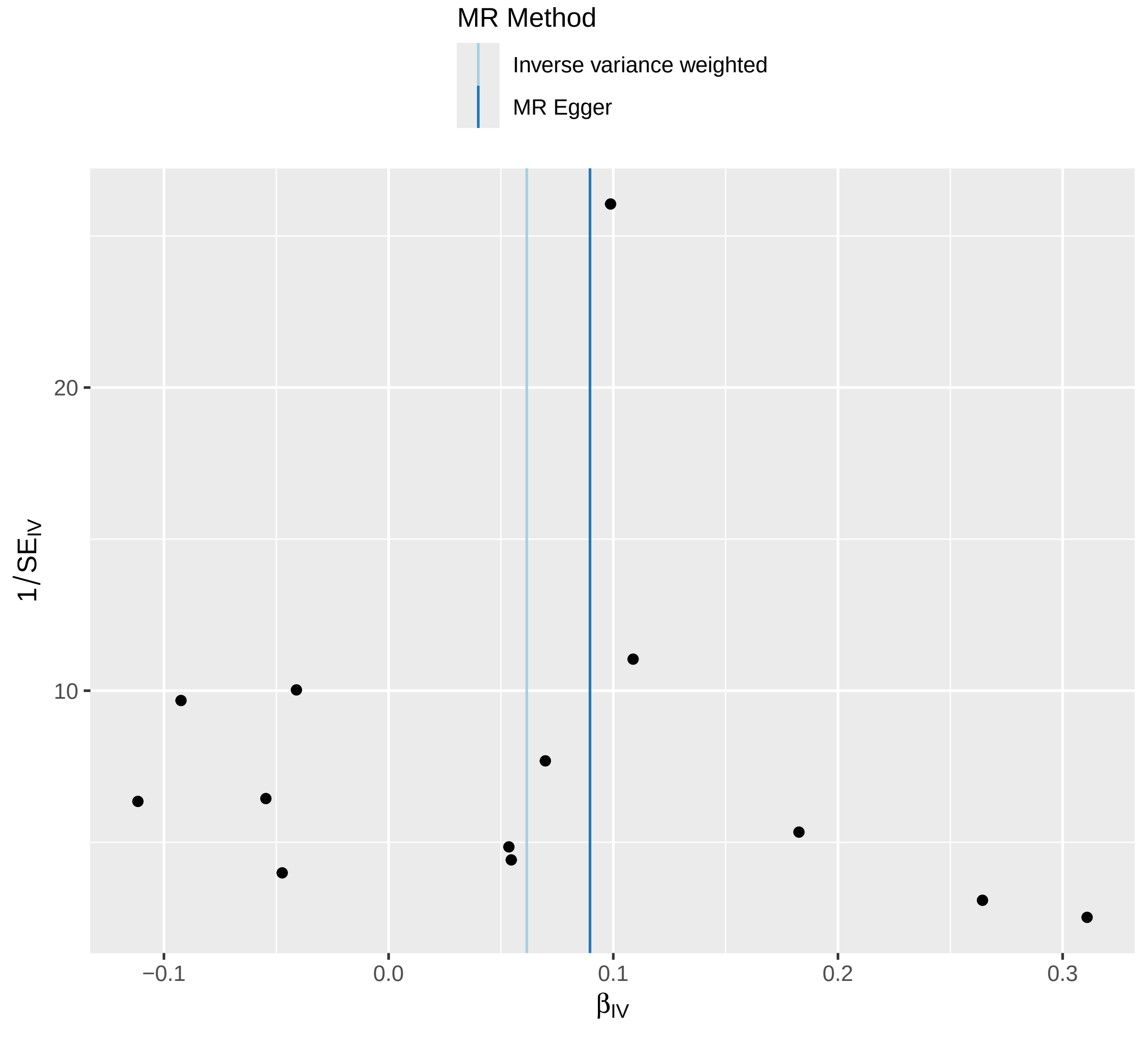
MR Method
Inverse variance weighted
MR Egger
20
10
1/SE_IV
−0.1
0.0
0.1
0.2
0.3
β_IV

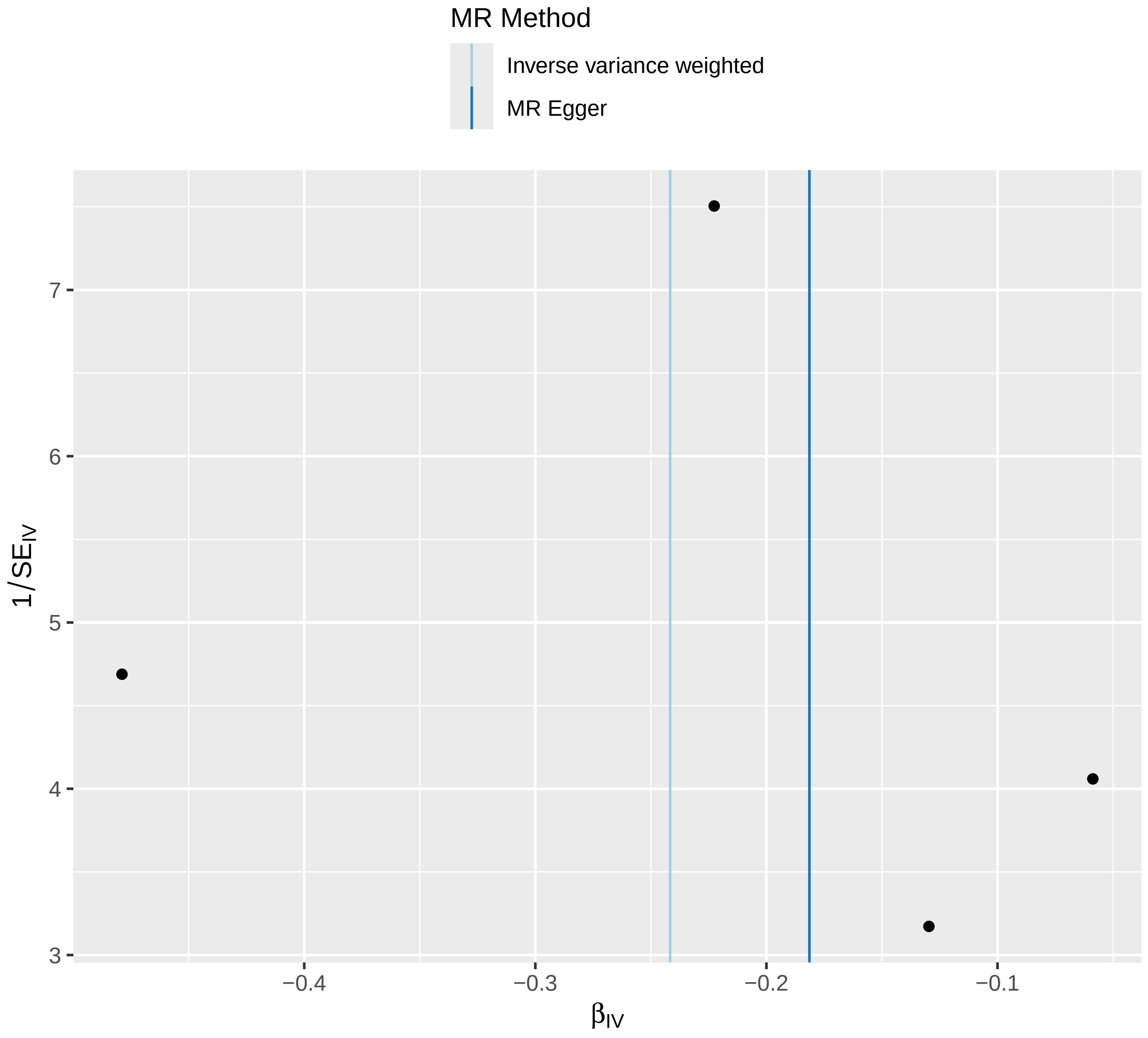
MR Method
Inverse variance weighted
MR Egger
7
6
5
4
3
1/SE_IV
−0.4
−0.3
−0.2
−0.1
β_IV

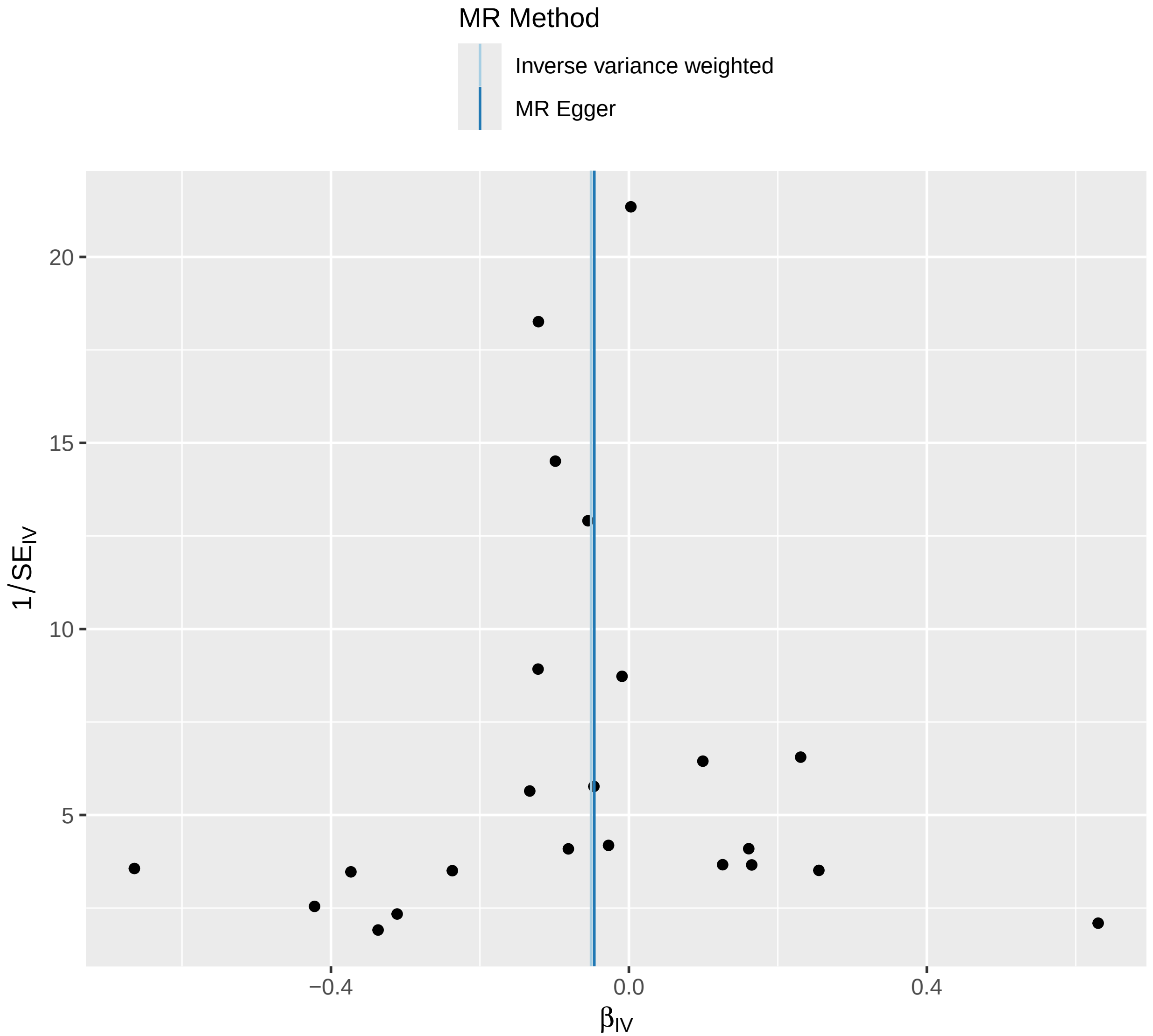

MR Method
Inverse variance weighted
MR Egger
20
15
10
5
1/SE_IV
−0.4
0.0
0.4
β_IV
