# Supplementary material for: Mediating Mendelian randomization in the proteome identified potential drug targets for obesity-related allergic asthma
Source: Hereditas. 2025 Feb 1;162:12. doi: 10.1186/s41065-025-00376-w (PMC11786417; doi:10.1186/s41065-025-00376-w)
Supplement: Supplementary file 1 — Supplementary Material 1: Supplementary Materials 1. The MR results of BMI and allergic asthma. Supplementary Materials 2. The MR results of plasma proteins and allergic asthma. Supplementary Materials 3. The MR results of BMI and plasma proteins. Supplementary Materials 4. The MR results of mediation factor plasma proteins and allergic asthma. Supplementary Materials 5. The results of drug-targeted MR [file 41065_2025_376_MOESM1_ESM.zip › Supplementary Materials/S4 The MR results of mediation factor plasma proteins and allergic asthma/S4 plasma proteins-Allergic asthma.scatter_plot.pdf]

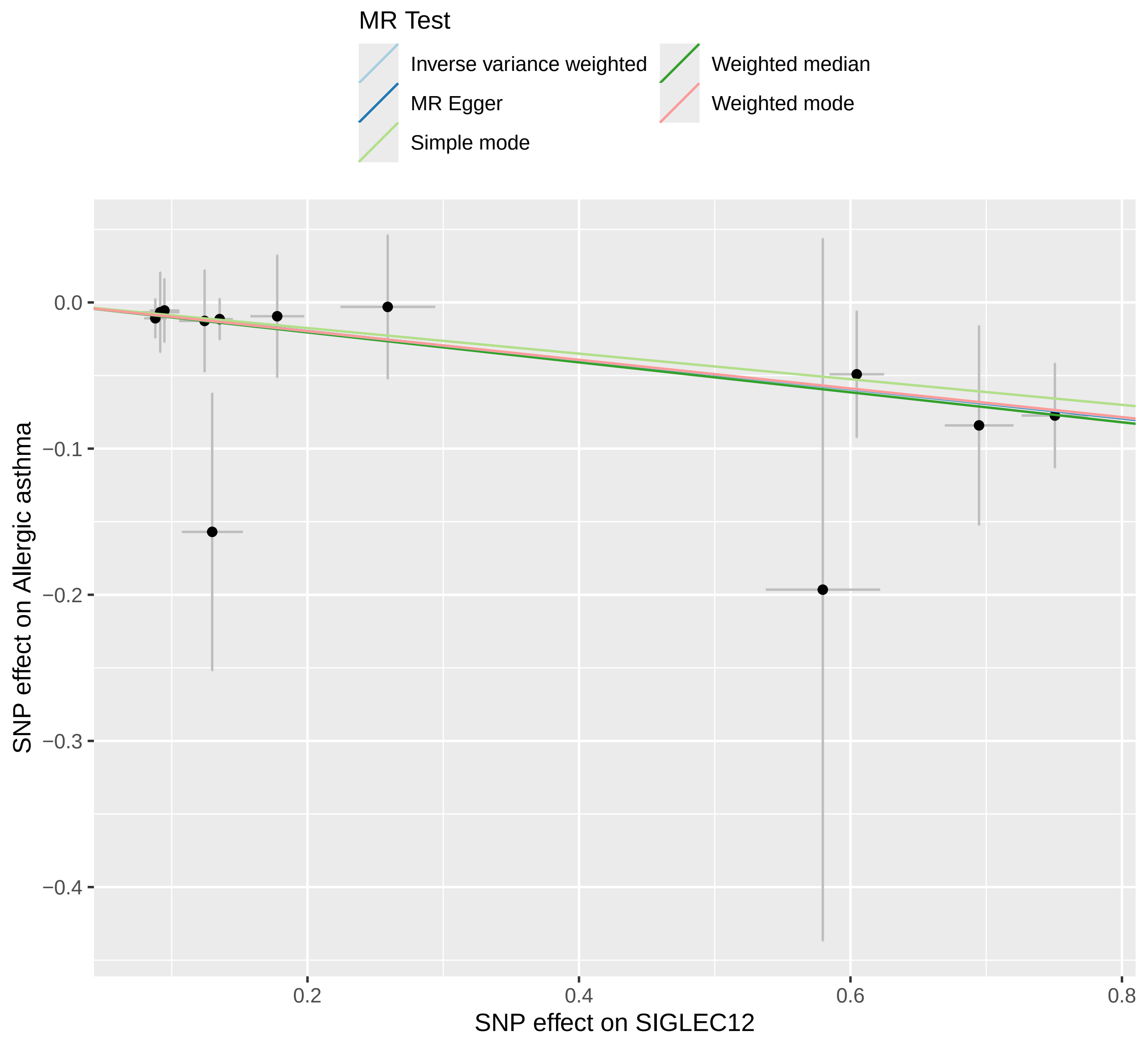
MR Test
Inverse variance weighted
MR Egger
Simple mode
Weighted median
Weighted mode
SNP effect on Allergic asthma
0.0
−0.1
−0.2
−0.3
−0.4
0.2
0.4
0.6
0.8
SNP effect on SIGLEC12

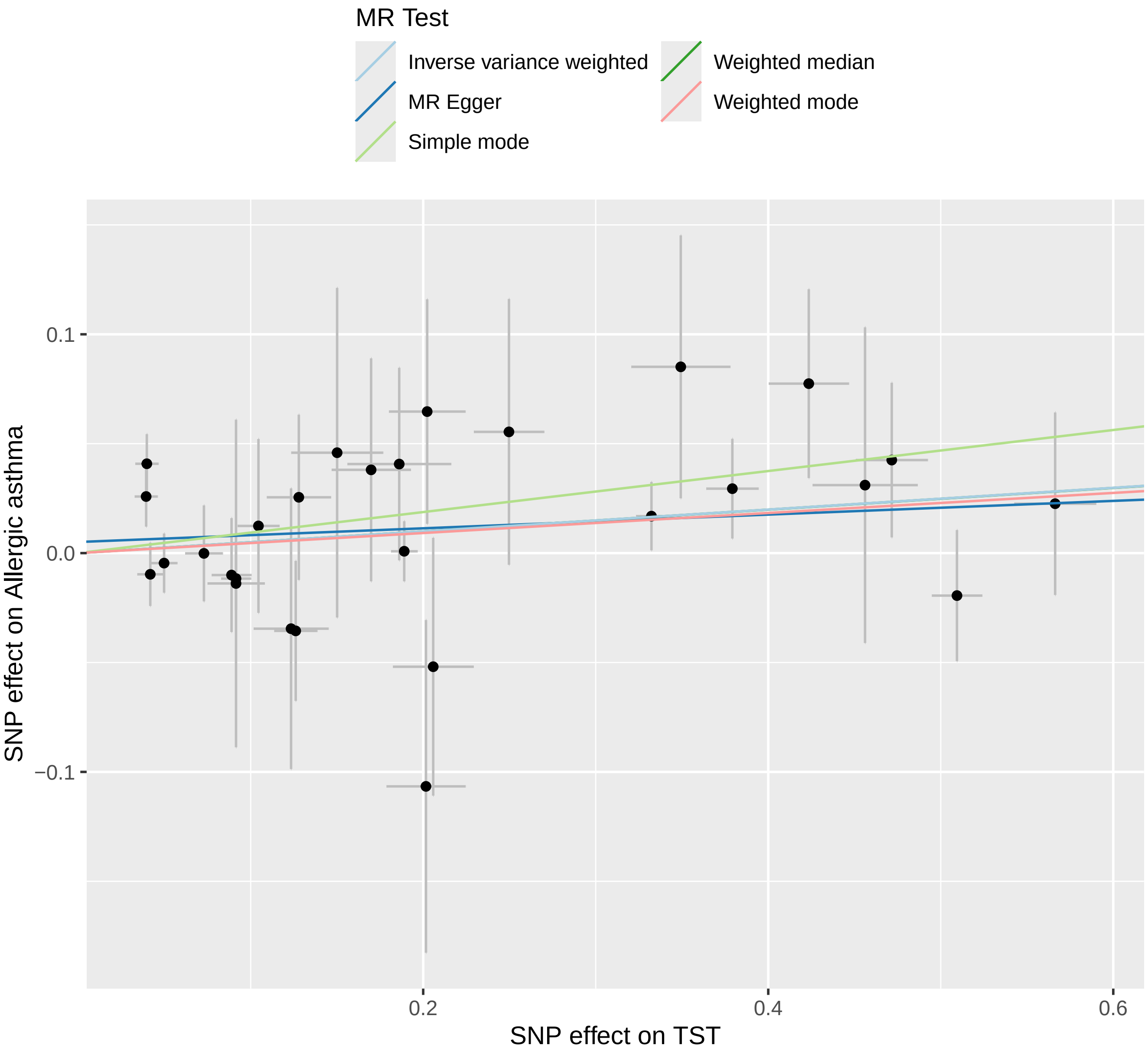

MR Test
Inverse variance weighted
MR Egger
Simple mode
Weighted median
Weighted mode
0.1
0.0
−0.1
0.2
0.4
0.6
SNP effect on Allergic asthma
SNP effect on TST

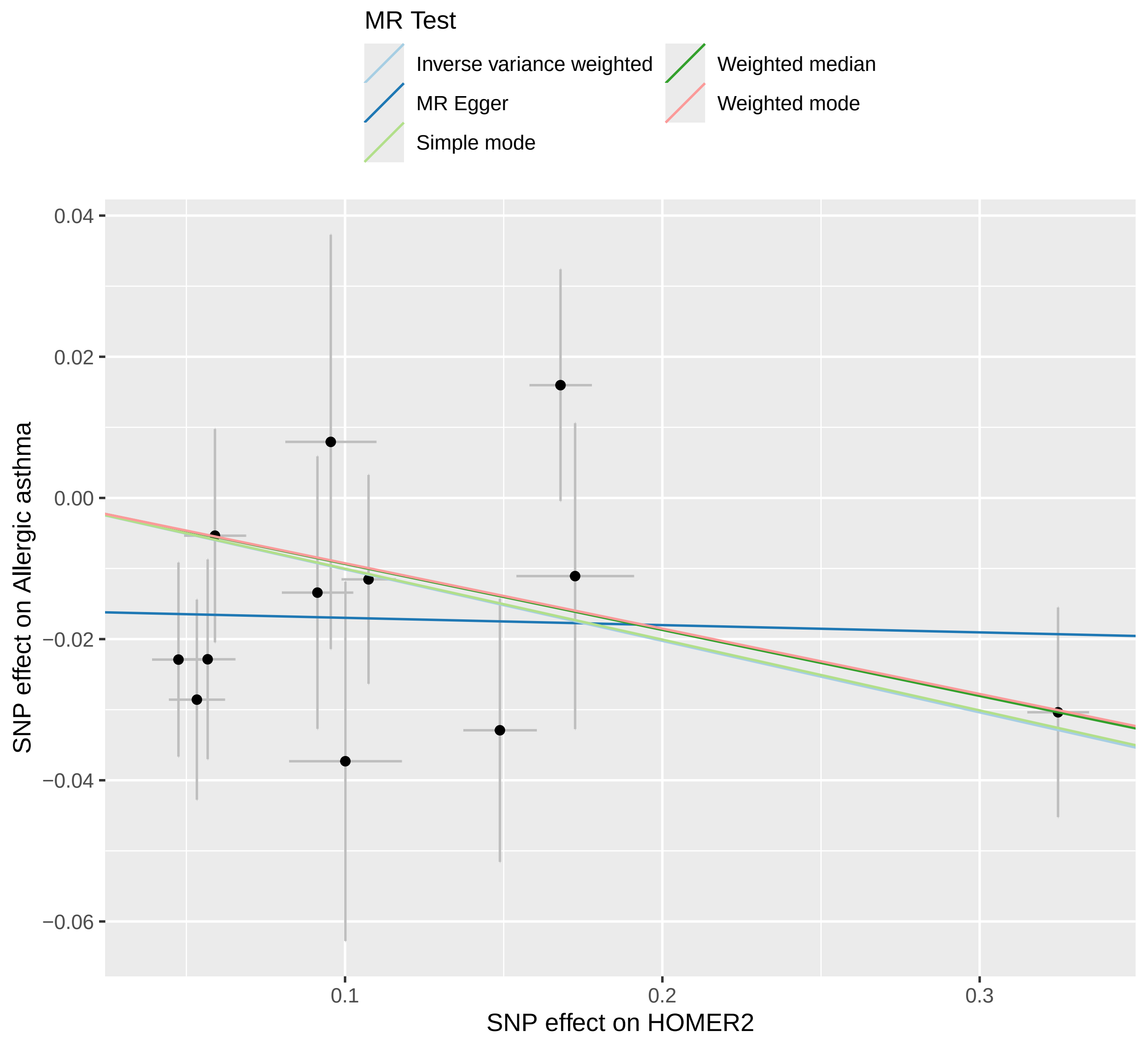
MR Test
Inverse variance weighted
MR Egger
Simple mode
Weighted median
Weighted mode
0.04
0.02
0.00
−0.02
−0.04
−0.06
SNP effect on Allergic asthma
0.1
0.2
0.3
SNP effect on HOMER2

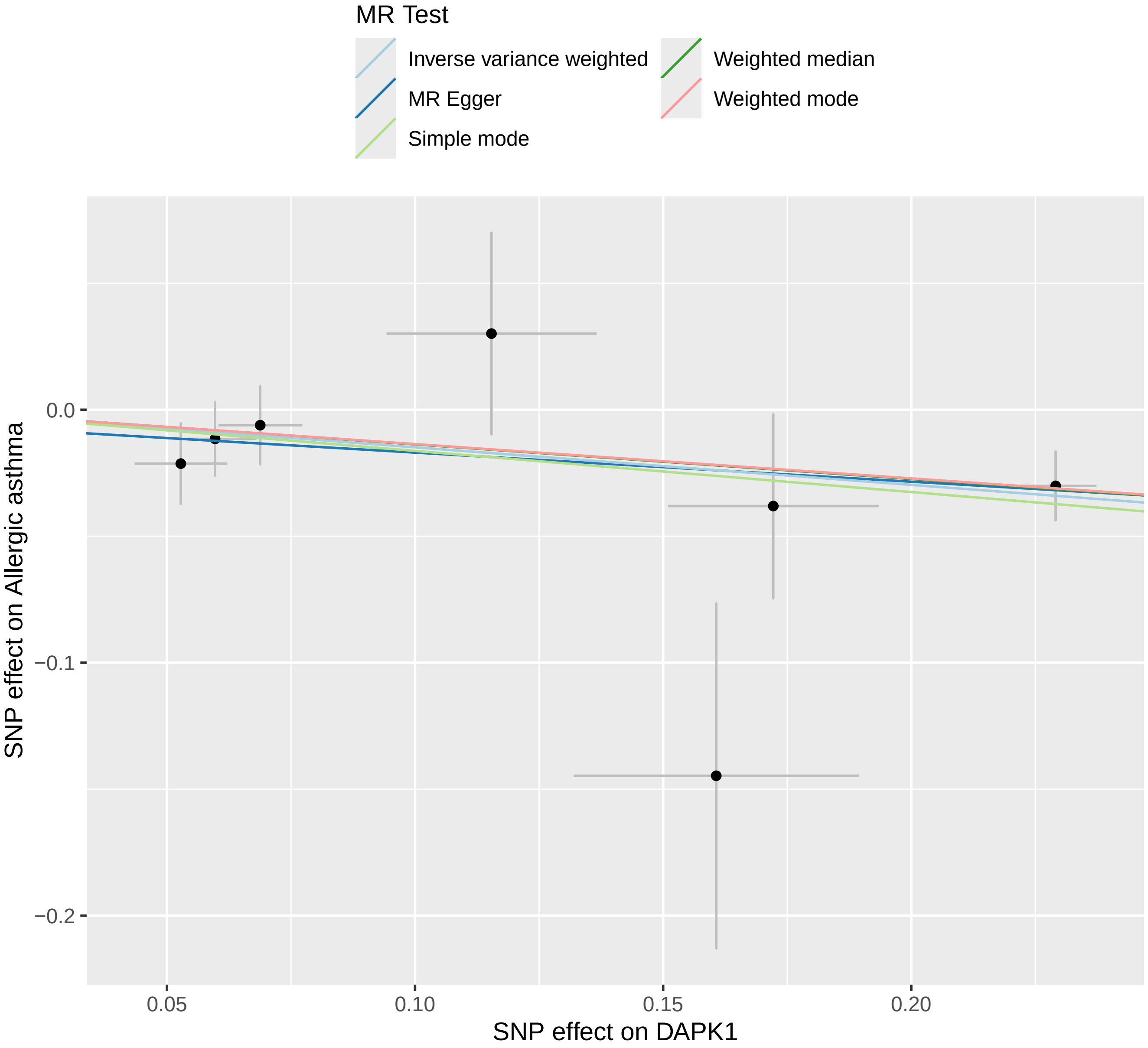
MR Test
Inverse variance weighted
MR Egger
Simple mode
Weighted median
Weighted mode
0.0
−0.1
−0.2
0.05
0.10
0.15
0.20
SNP effect on Allergic asthma
SNP effect on DAPK1

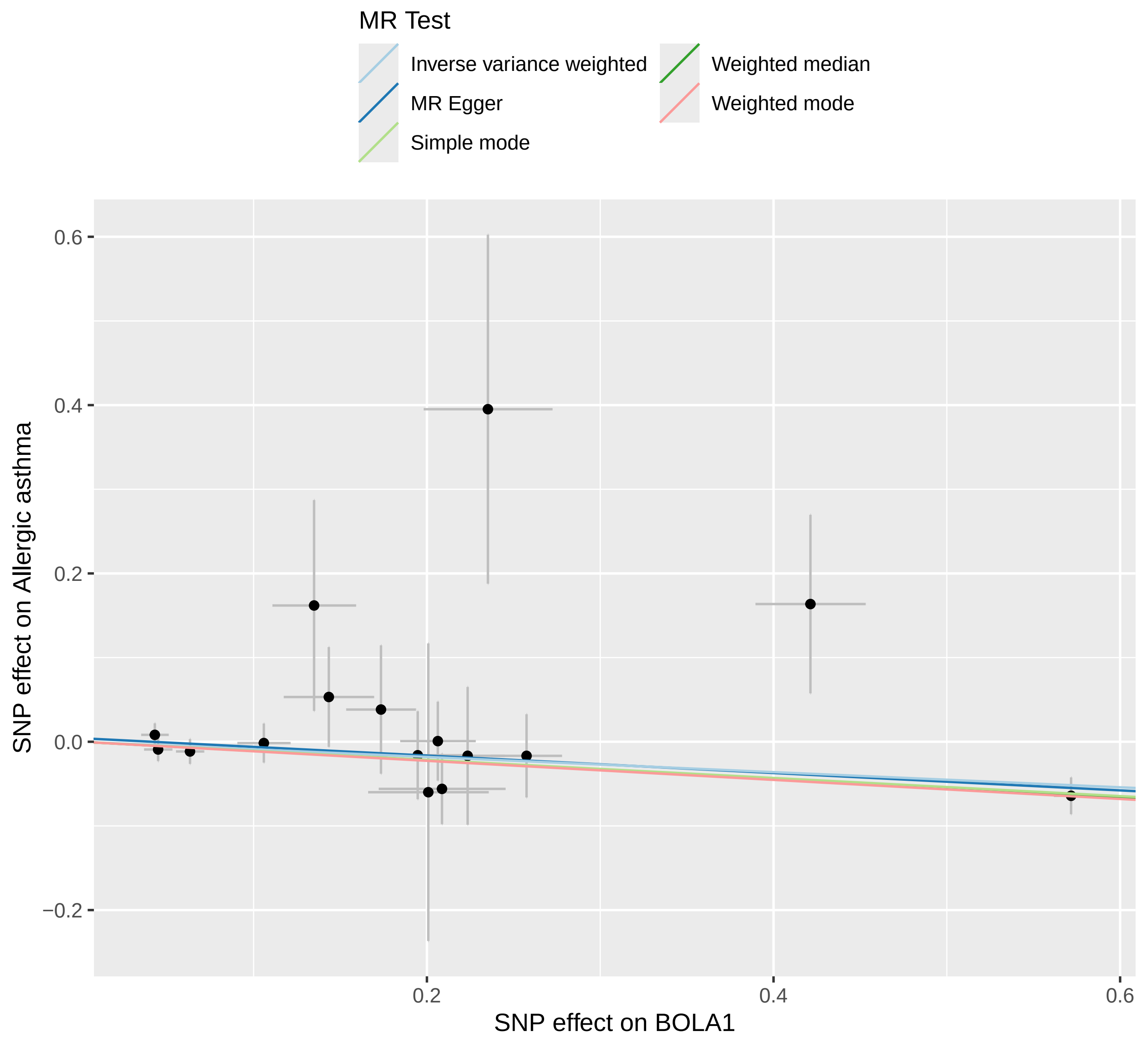
MR Test
Inverse variance weighted
MR Egger
Simple mode
Weighted median
Weighted mode
0.6
0.4
0.2
0.0
−0.2
SNP effect on Allergic asthma
0.2
0.4
0.6
SNP effect on BOLA1

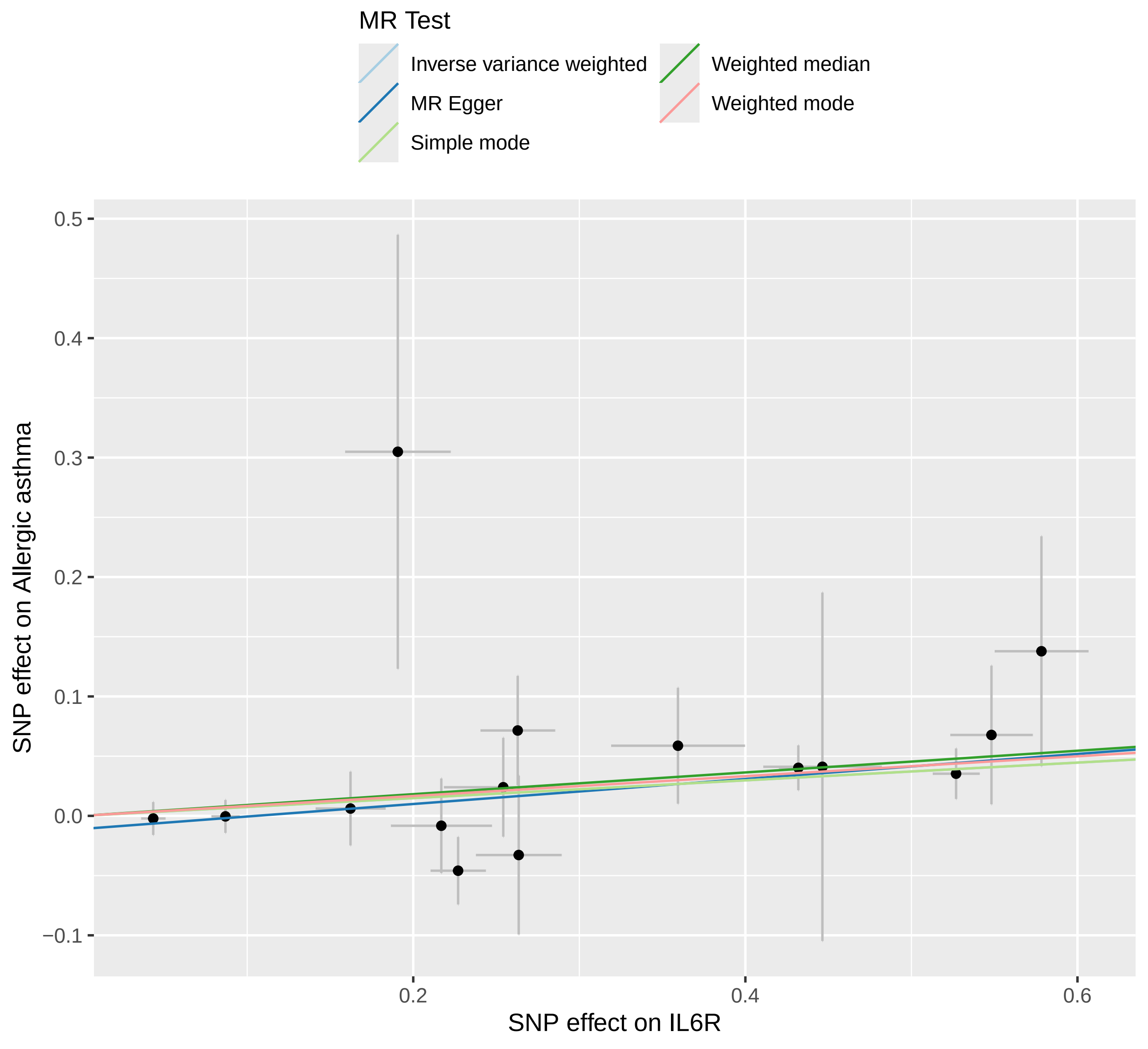

MR Test
Inverse variance weighted
MR Egger
Simple mode
Weighted median
Weighted mode
SNP effect on Allergic asthma
0.5
0.4
0.3
0.2
0.1
0.0
−0.1
0.2
0.4
0.6
SNP effect on IL6R

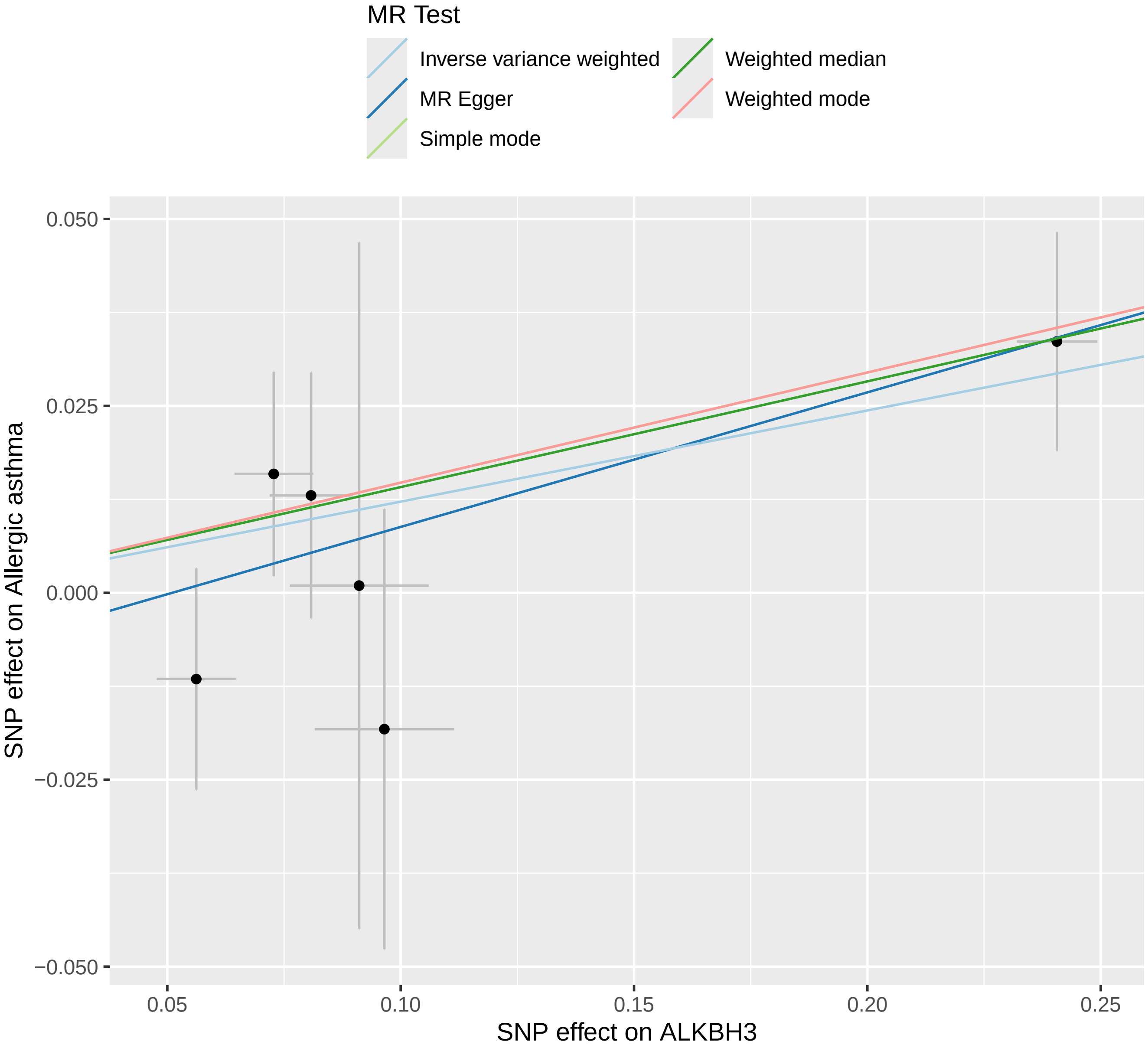

MR Test
Inverse variance weighted
MR Egger
Simple mode
Weighted median
Weighted mode
0.050
0.025
0.000
−0.025
−0.050
0.05
0.10
0.15
0.20
0.25
SNP effect on ALKBH3
SNP effect on Allergic asthma

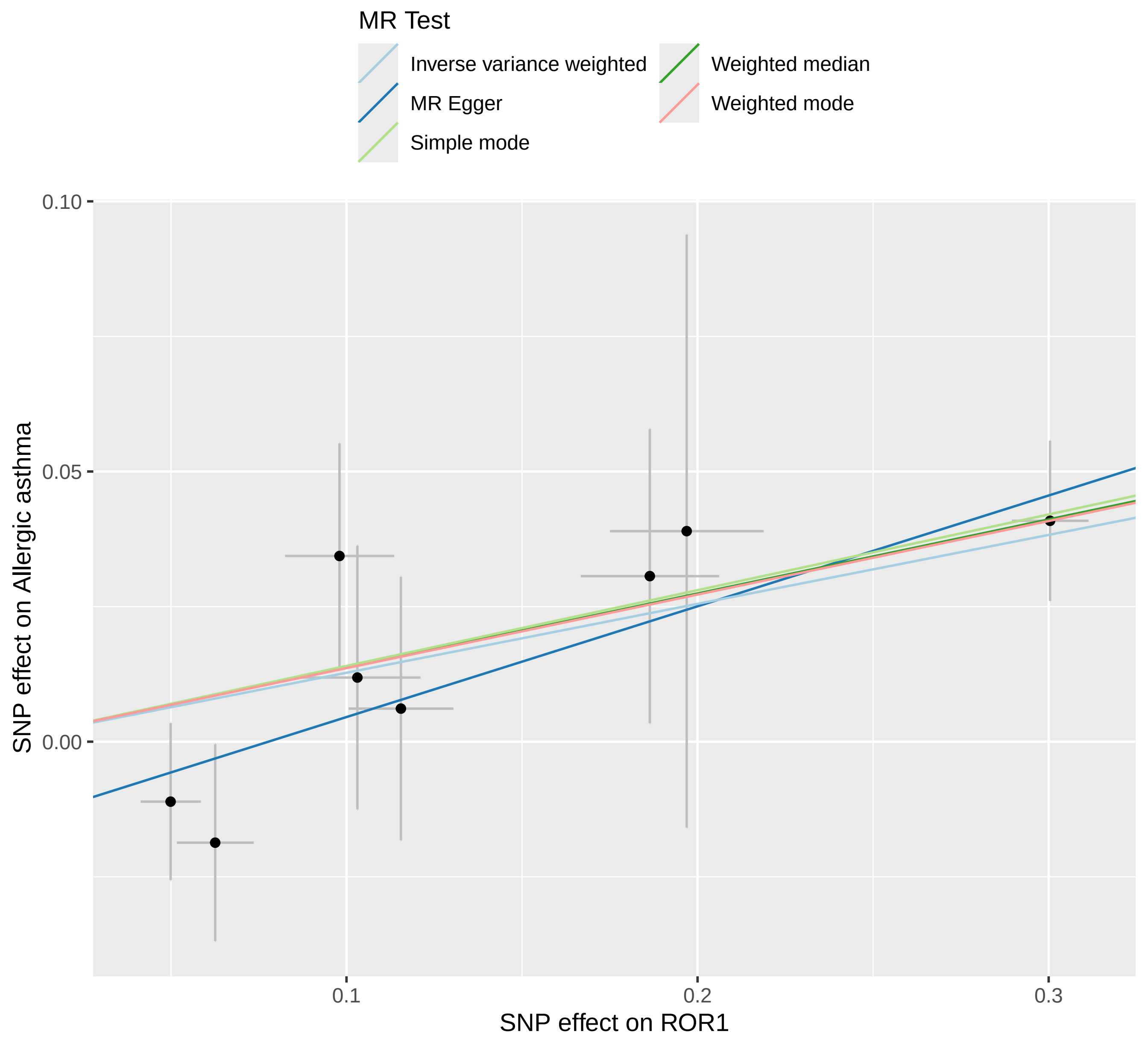
MR Test
Inverse variance weighted
MR Egger
Simple mode
Weighted median
Weighted mode
0.10
0.05
0.00
SNP effect on Allergic asthma
0.1
0.2
0.3
SNP effect on ROR1

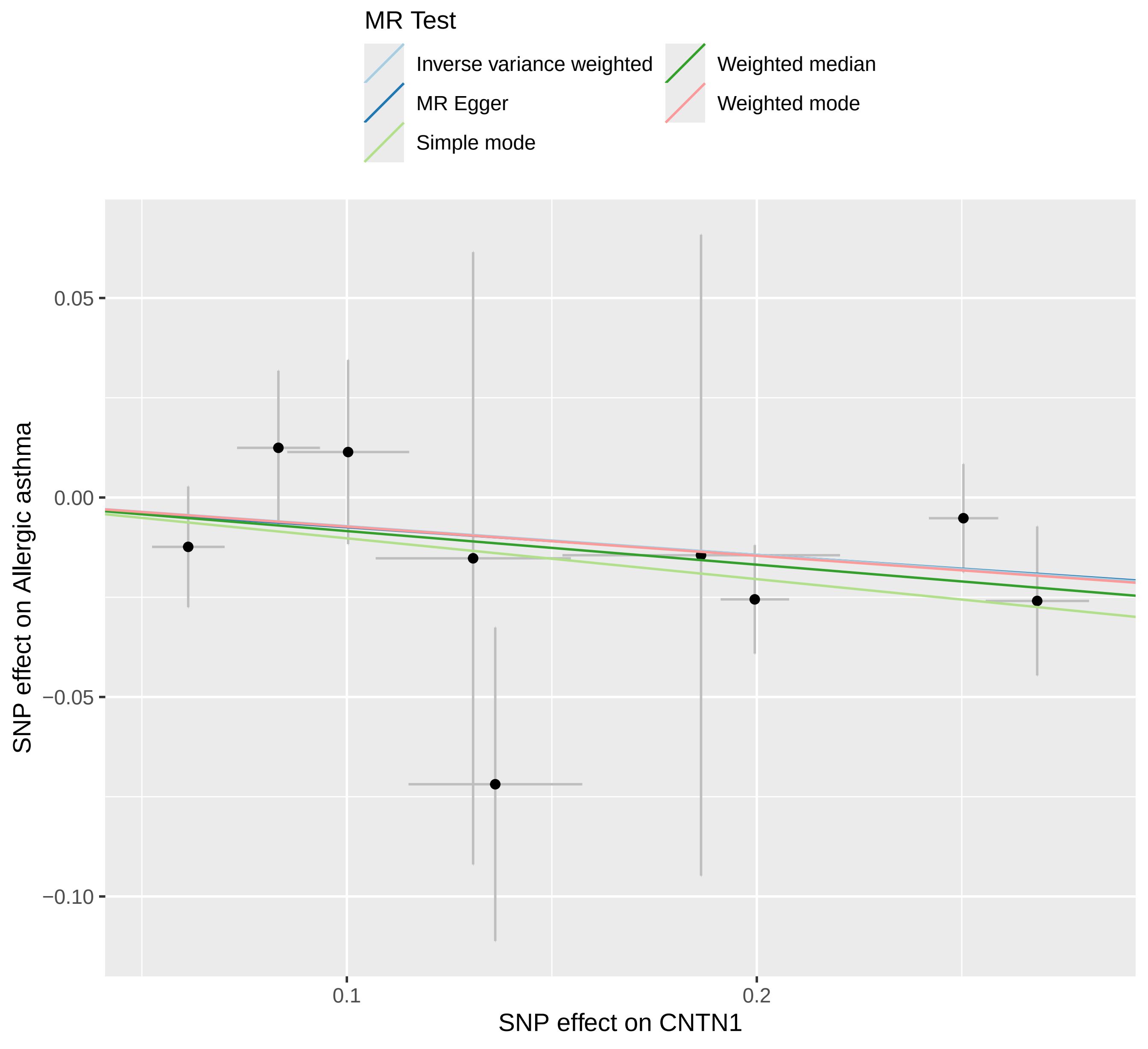
MR Test
Inverse variance weighted
MR Egger
Simple mode
Weighted median
Weighted mode
0.05
0.00
−0.05
−0.10
SNP effect on Allergic asthma
0.1
0.2
SNP effect on CNTN1

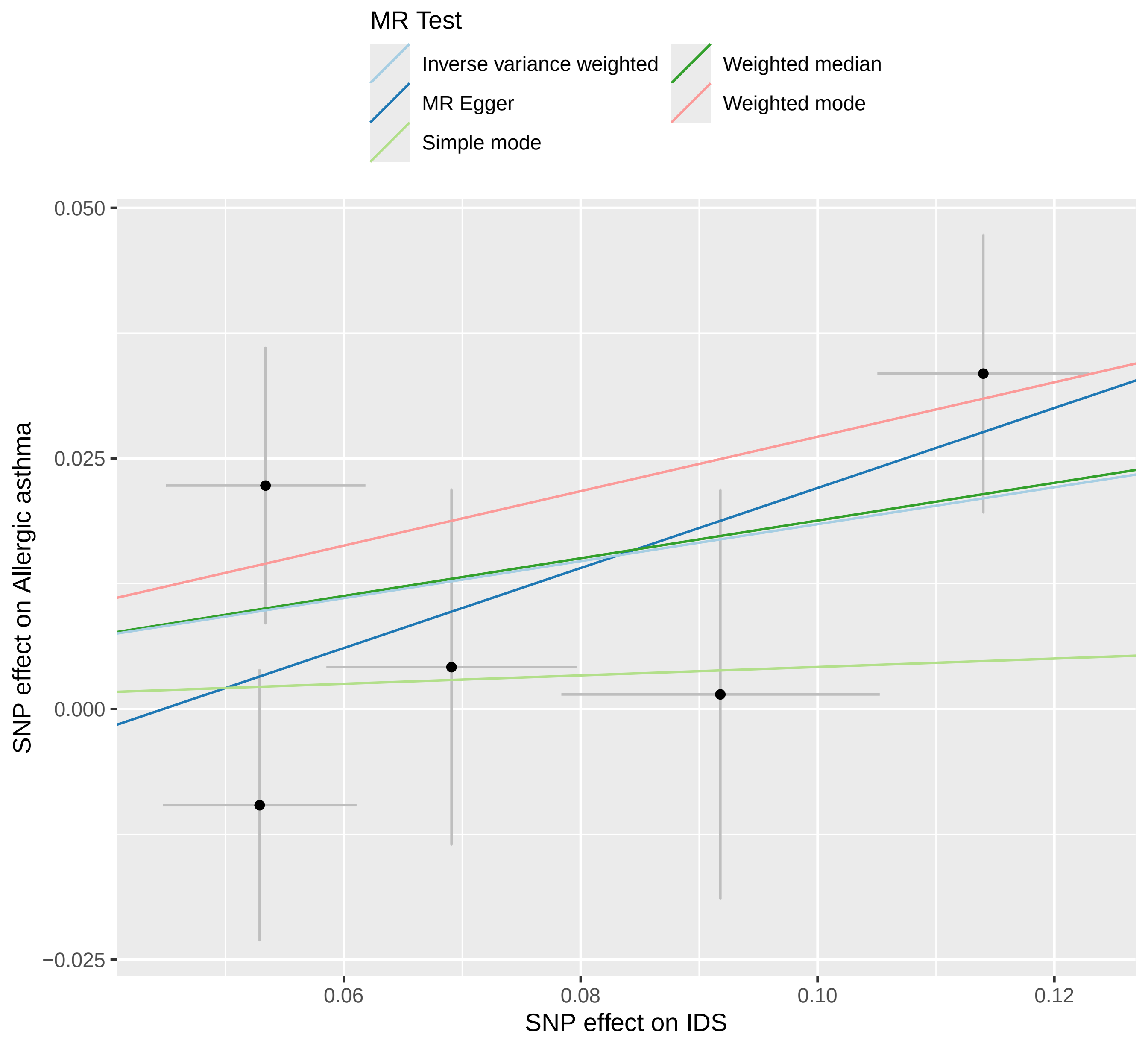
MR Test
Inverse variance weighted
MR Egger
Simple mode
Weighted median
Weighted mode
0.050
0.025
0.000
−0.025
0.06
0.08
0.10
0.12
SNP effect on IDS
SNP effect on Allergic asthma

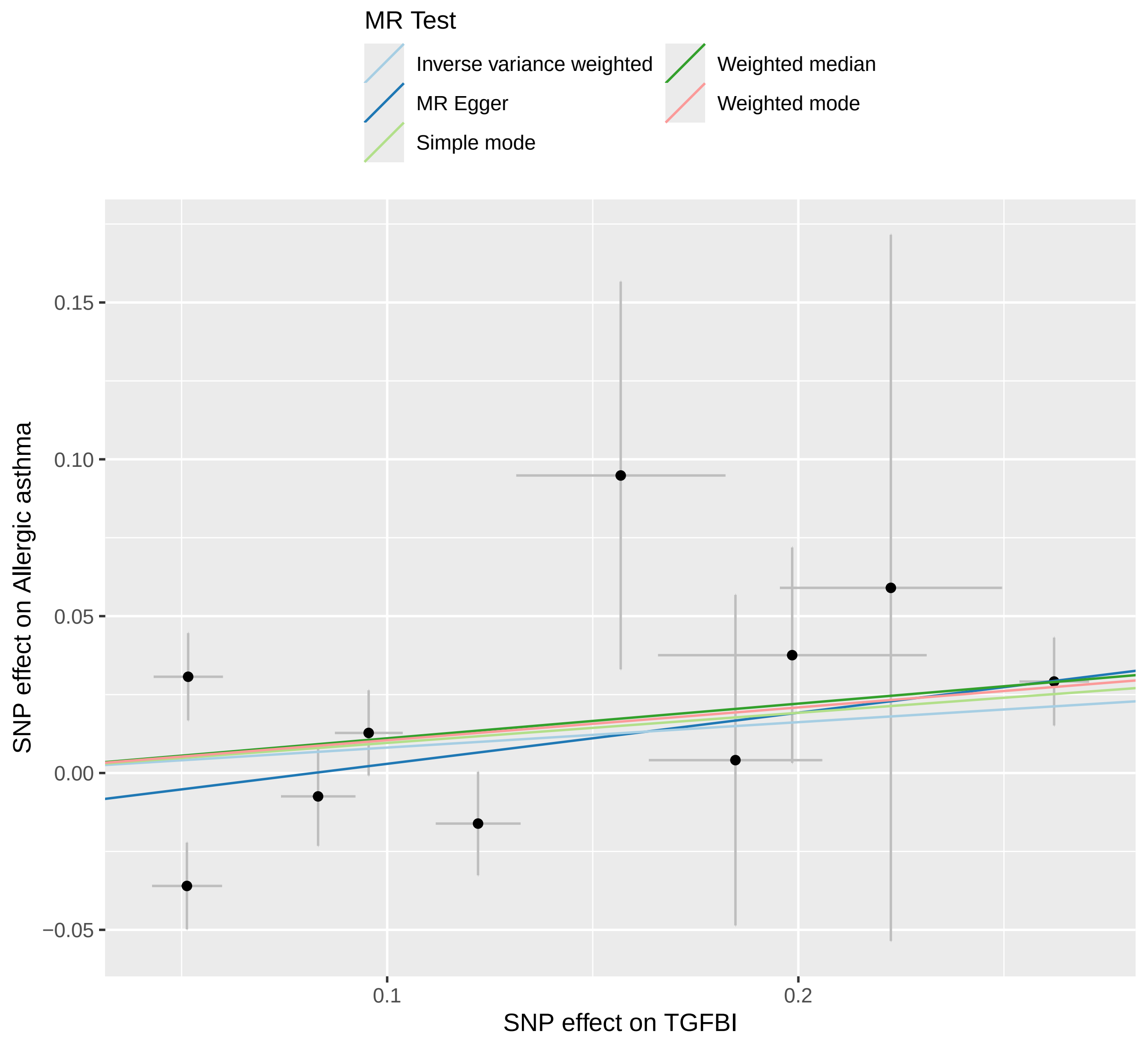

MR Test
Inverse variance weighted
MR Egger
Simple mode
Weighted median
Weighted mode
0.15
0.10
0.05
0.00
−0.05
0.1
0.2
SNP effect on Allergic asthma
SNP effect on TGFBI

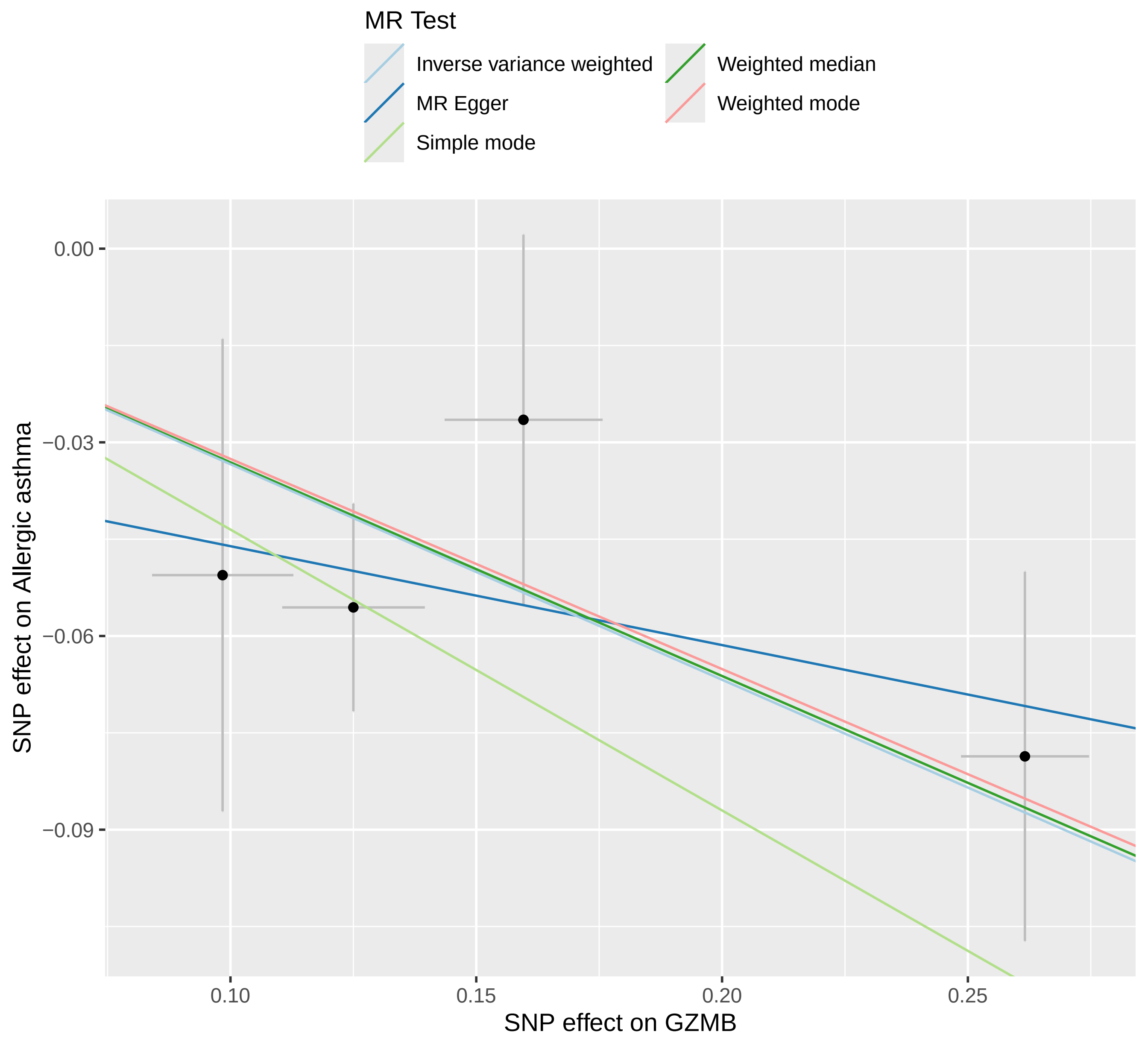
MR Test
Inverse variance weighted
MR Egger
Simple mode
Weighted median
Weighted mode
0.00
−0.03
−0.06
−0.09
SNP effect on Allergic asthma
0.10
0.15
0.20
0.25
SNP effect on GZMB

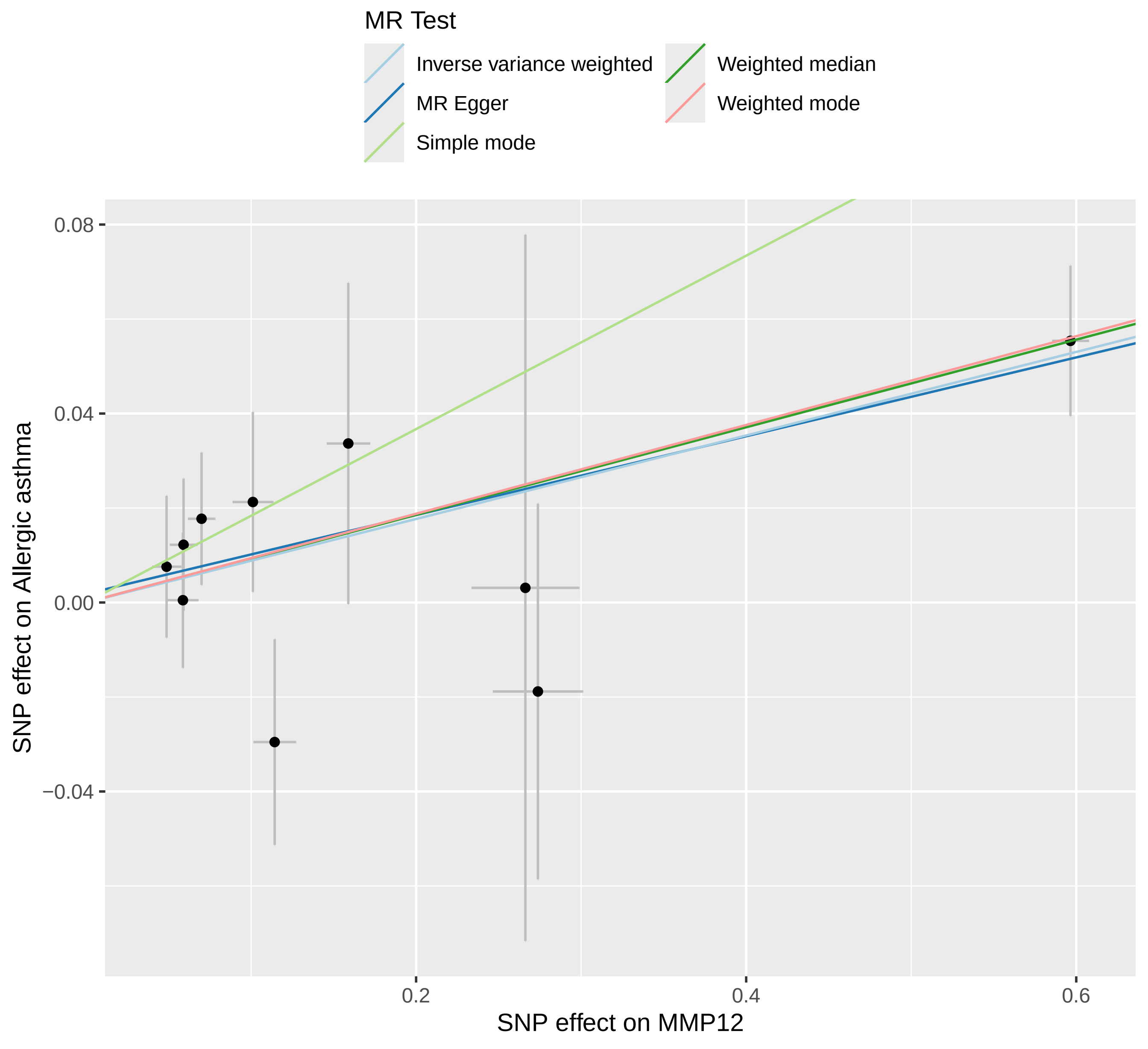

MR Test
Inverse variance weighted
MR Egger
Simple mode
Weighted median
Weighted mode
0.08
0.04
0.00
−0.04
SNP effect on Allergic asthma
0.2
0.4
0.6
SNP effect on MMP12

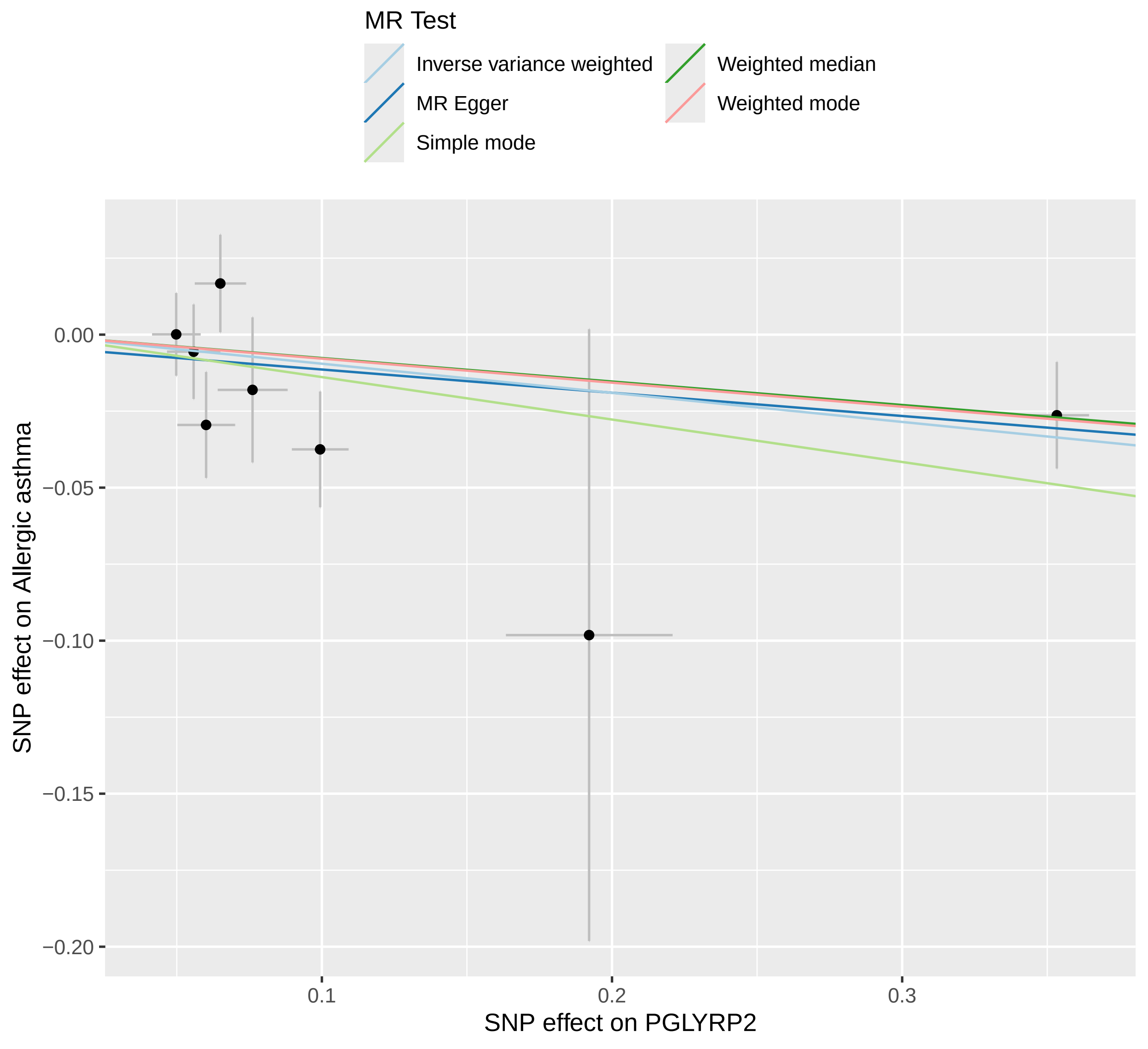
MR Test
Inverse variance weighted
MR Egger
Simple mode
Weighted median
Weighted mode
SNP effect on Allergic asthma
0.00
−0.05
−0.10
−0.15
−0.20
0.1
0.2
0.3
SNP effect on PGLYRP2

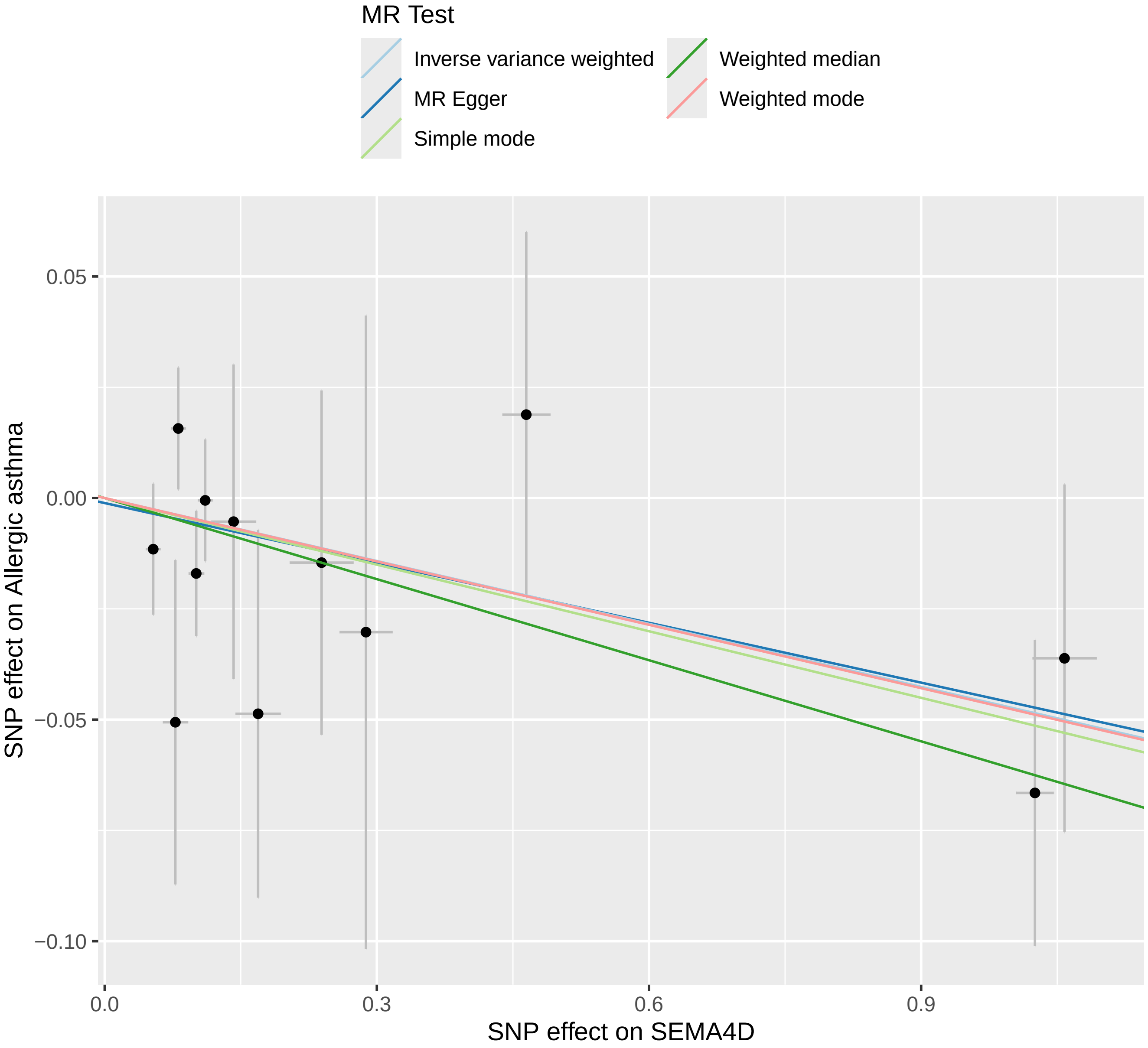

MR Test
Inverse variance weighted
MR Egger
Simple mode
Weighted median
Weighted mode
0.05
0.00
−0.05
−0.10
SNP effect on Allergic asthma
0.0
0.3
0.6
0.9
SNP effect on SEMA4D

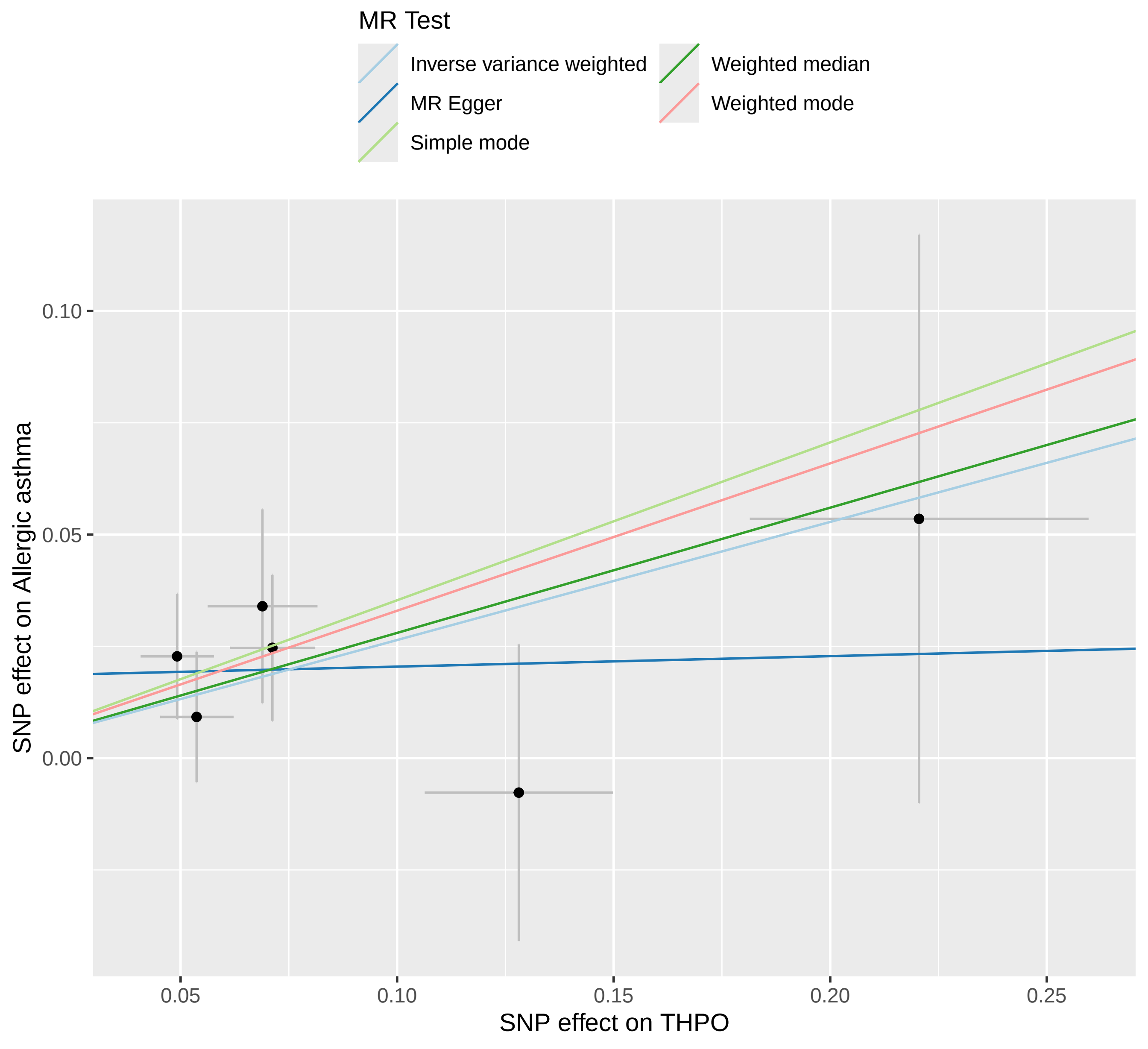
MR Test
Inverse variance weighted
MR Egger
Simple mode
Weighted median
Weighted mode
SNP effect on Allergic asthma
0.10
0.05
0.00
0.05
0.10
0.15
0.20
0.25
SNP effect on THPO

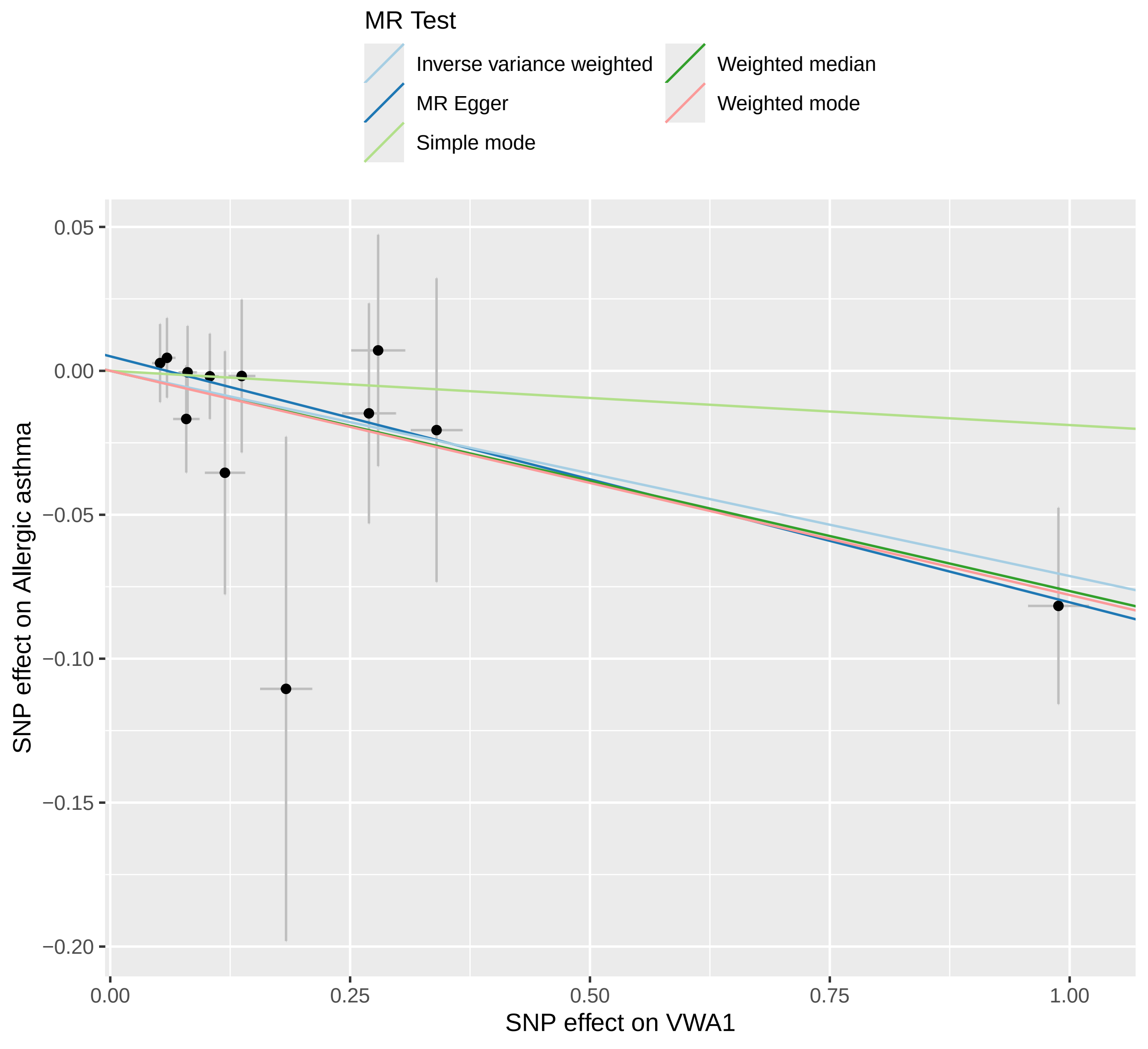

MR Test
Inverse variance weighted
MR Egger
Simple mode
Weighted median
Weighted mode
0.05
0.00
−0.05
−0.10
−0.15
−0.20
0.00
0.25
0.50
0.75
1.00
SNP effect on Allergic asthma
SNP effect on VWA1

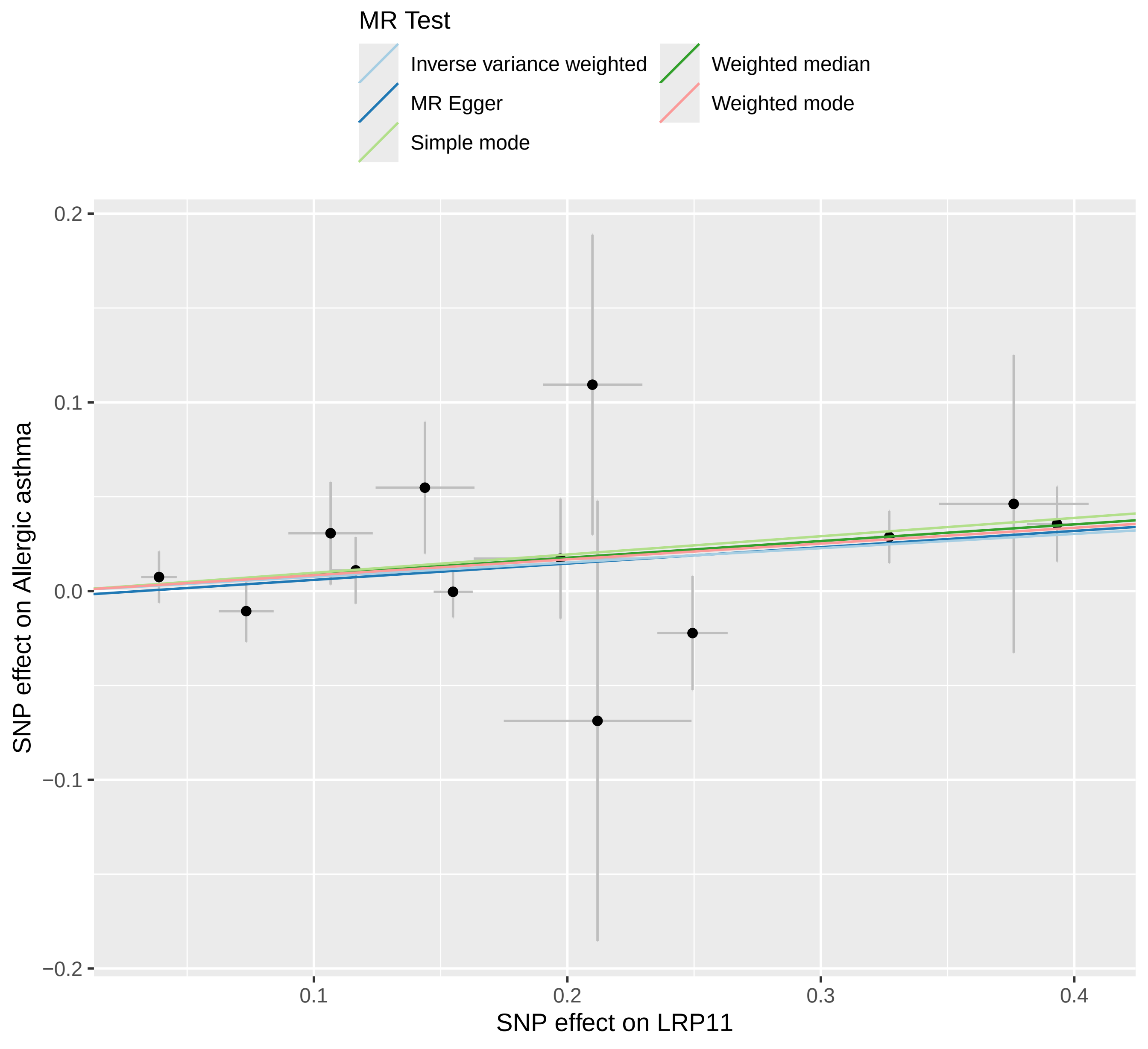

MR Test
Inverse variance weighted
MR Egger
Simple mode
Weighted median
Weighted mode
0.2
0.1
0.0
−0.1
−0.2
SNP effect on Allergic asthma
0.1
0.2
0.3
0.4
SNP effect on LRP11

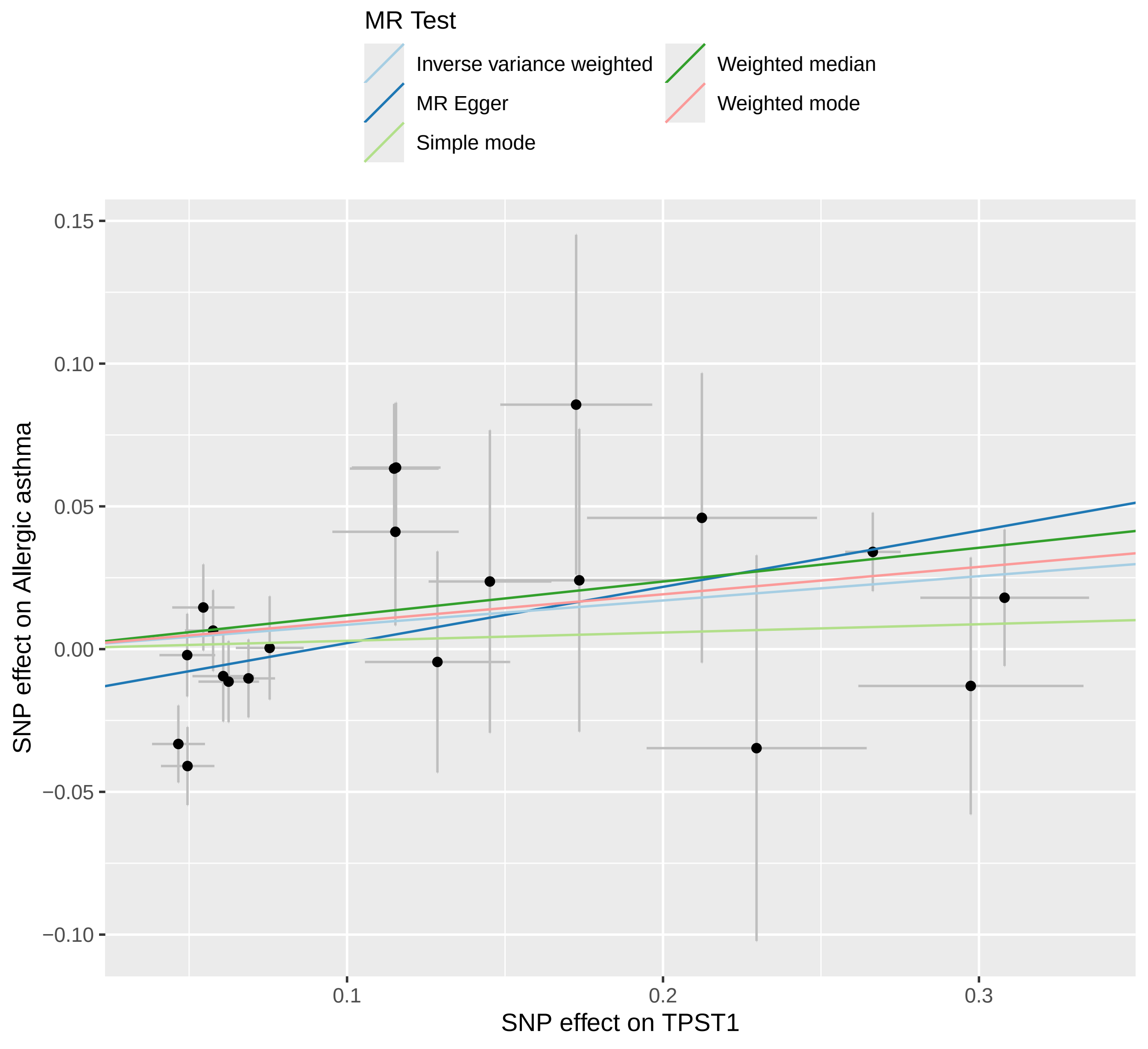
MR Test
Inverse variance weighted
MR Egger
Simple mode
Weighted median
Weighted mode
0.15
0.10
0.05
0.00
−0.05
−0.10
SNP effect on Allergic asthma
0.1
0.2
0.3
SNP effect on TPST1

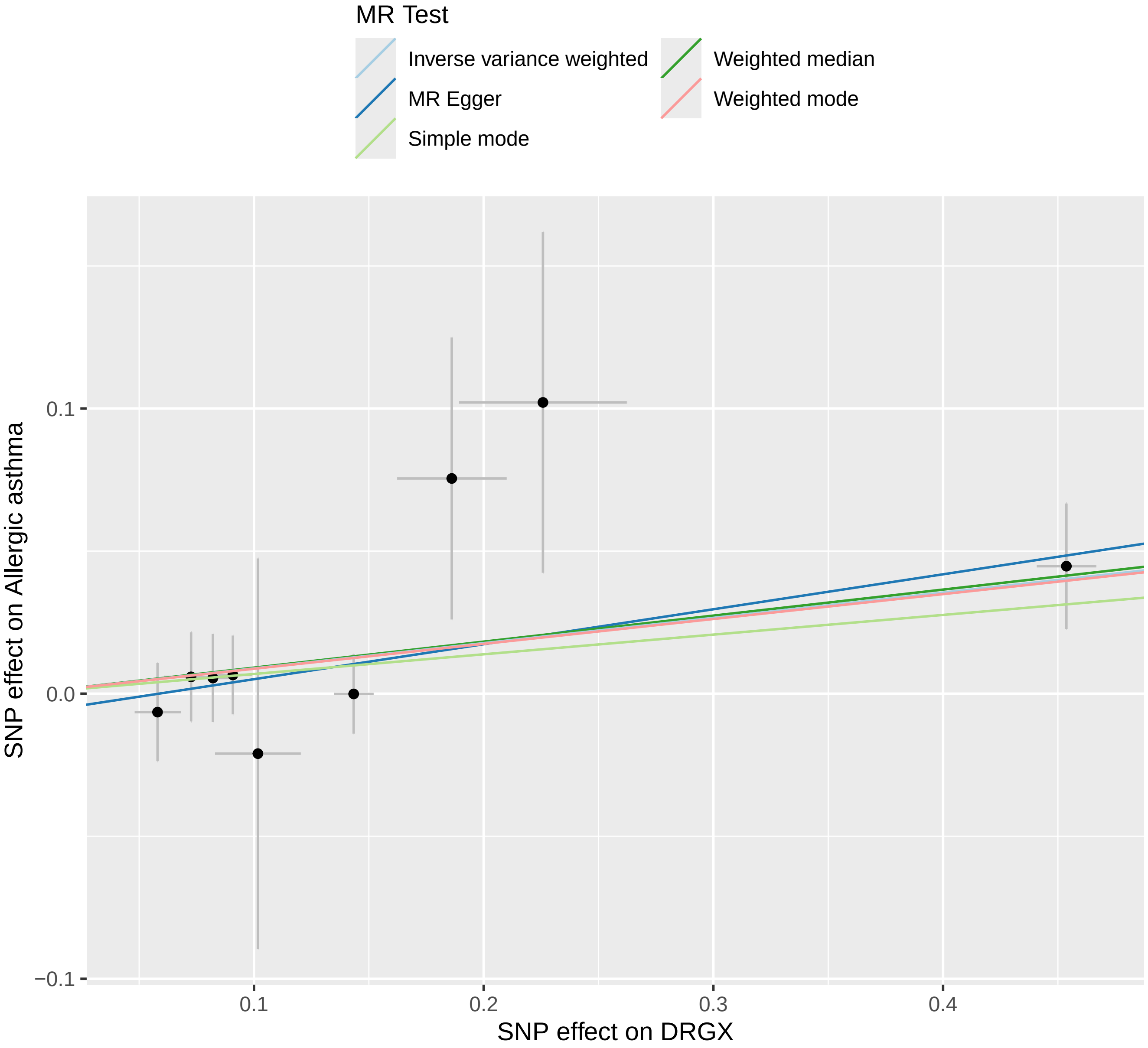

MR Test
Inverse variance weighted
MR Egger
Simple mode
Weighted median
Weighted mode
SNP effect on Allergic asthma
0.1
0.0
−0.1
0.1
0.2
0.3
0.4
SNP effect on DRGX

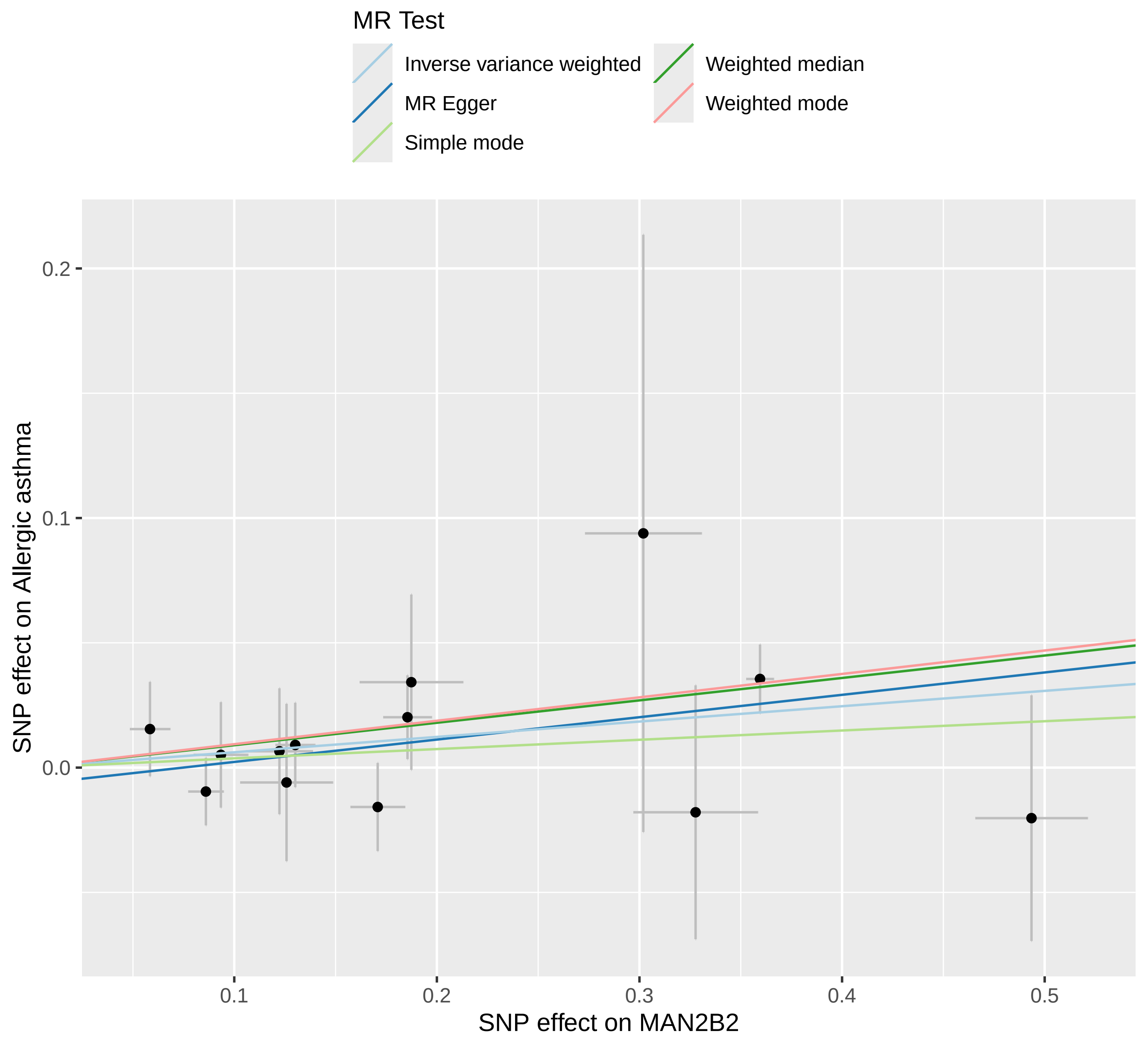
MR Test
Inverse variance weighted
MR Egger
Simple mode
Weighted median
Weighted mode
SNP effect on Allergic asthma
0.2
0.1
0.0
0.1
0.2
0.3
0.4
0.5
SNP effect on MAN2B2

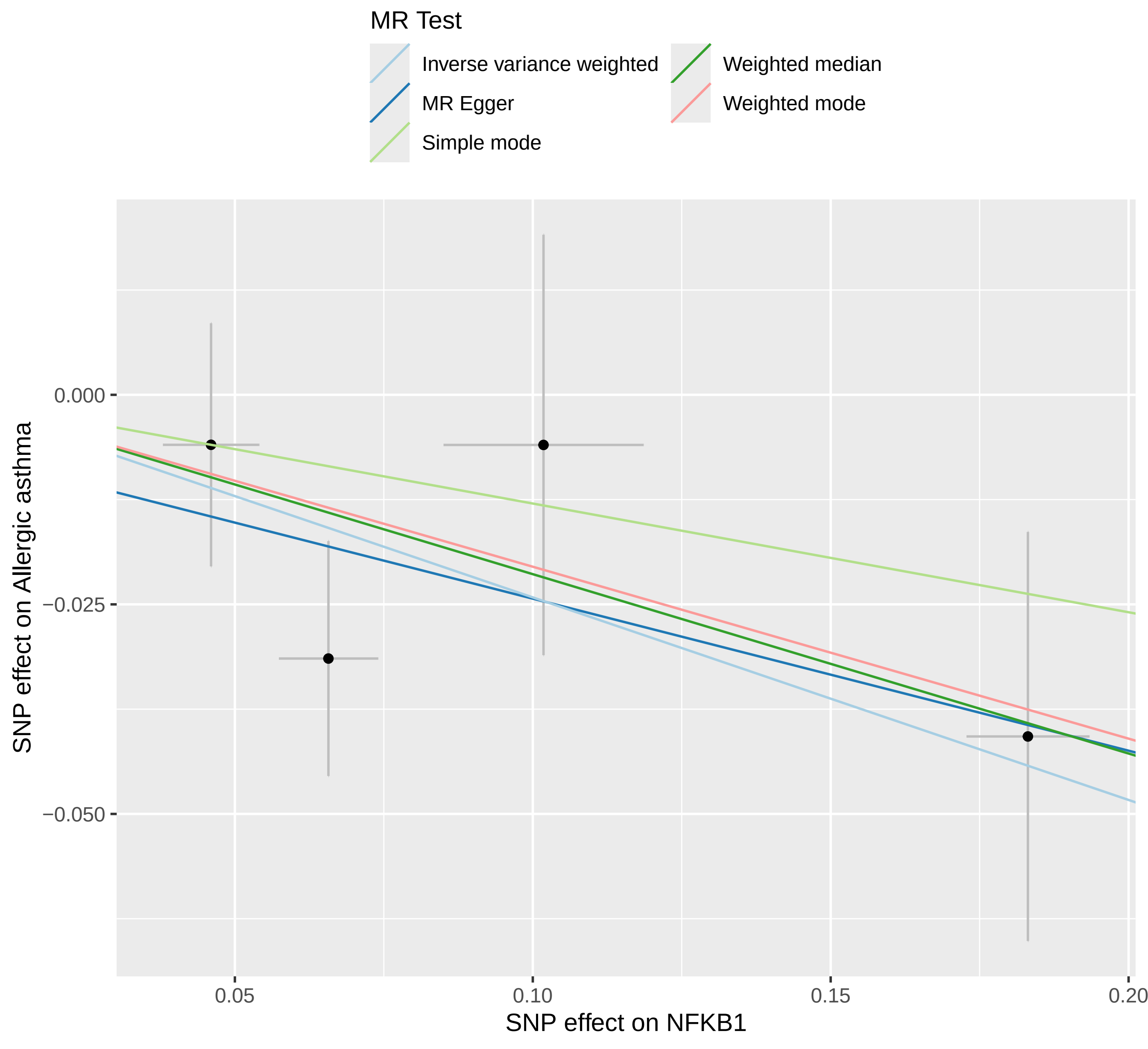

MR Test
Inverse variance weighted
MR Egger
Simple mode
Weighted median
Weighted mode
0.000
−0.025
−0.050
SNP effect on Allergic asthma
0.05
0.10
0.15
0.20
SNP effect on NFKB1

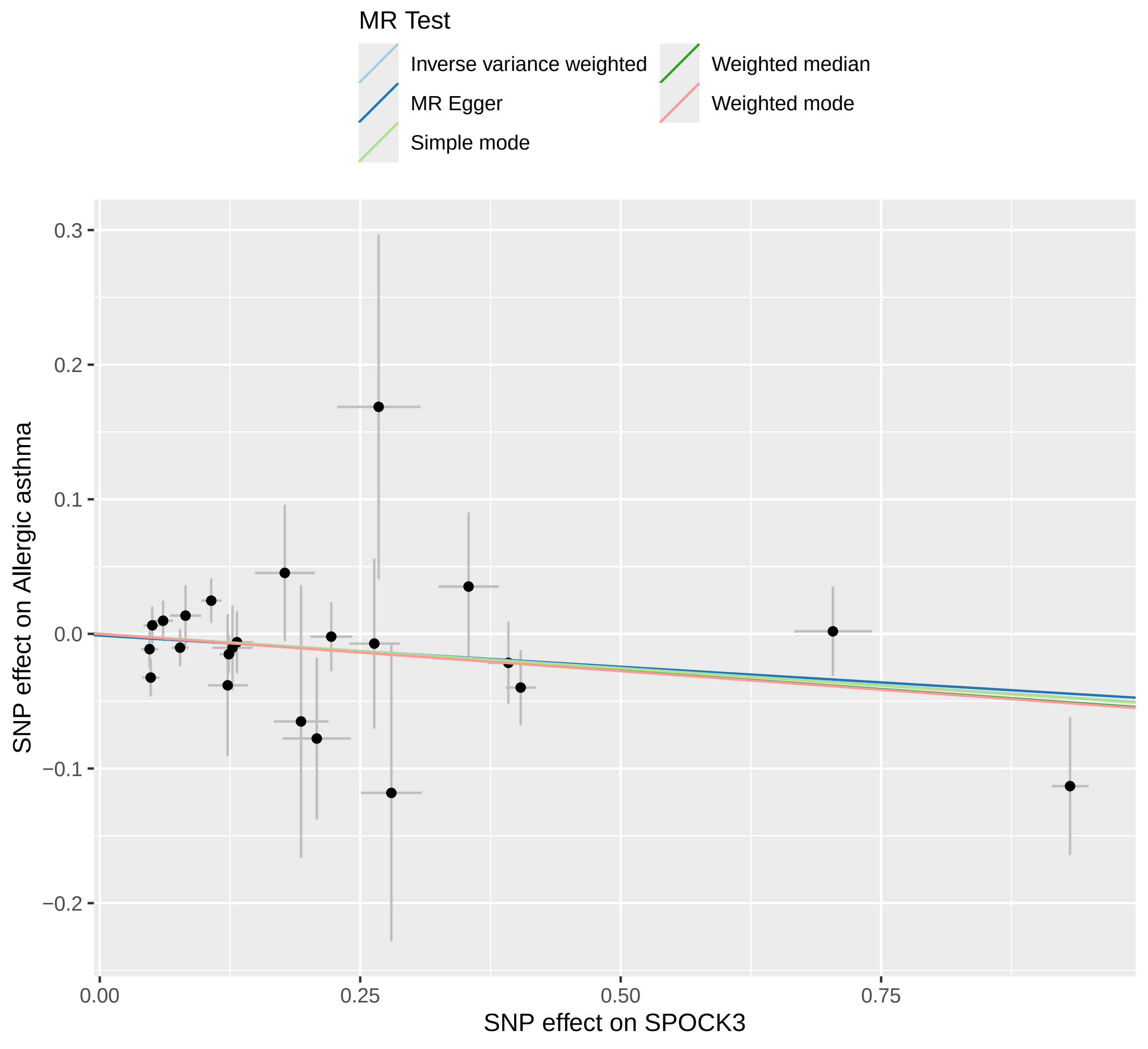
MR Test
Inverse variance weighted
MR Egger
Simple mode
Weighted median
Weighted mode
0.3
0.2
0.1
0.0
−0.1
−0.2
SNP effect on Allergic asthma
0.00
0.25
0.50
0.75
SNP effect on SPOCK3
