# Supplementary material for: Mediating Mendelian randomization in the proteome identified potential drug targets for obesity-related allergic asthma
Source: Hereditas. 2025 Feb 1;162:12. doi: 10.1186/s41065-025-00376-w (PMC11786417; doi:10.1186/s41065-025-00376-w)
Supplement: Supplementary file 1 — Supplementary Material 1: Supplementary Materials 1. The MR results of BMI and allergic asthma. Supplementary Materials 2. The MR results of plasma proteins and allergic asthma. Supplementary Materials 3. The MR results of BMI and plasma proteins. Supplementary Materials 4. The MR results of mediation factor plasma proteins and allergic asthma. Supplementary Materials 5. The results of drug-targeted MR [file 41065_2025_376_MOESM1_ESM.zip › Supplementary Materials/S5 The results of drug-targeted MR/S5 The results of drug-targeted MR.scatter_plot.pdf]

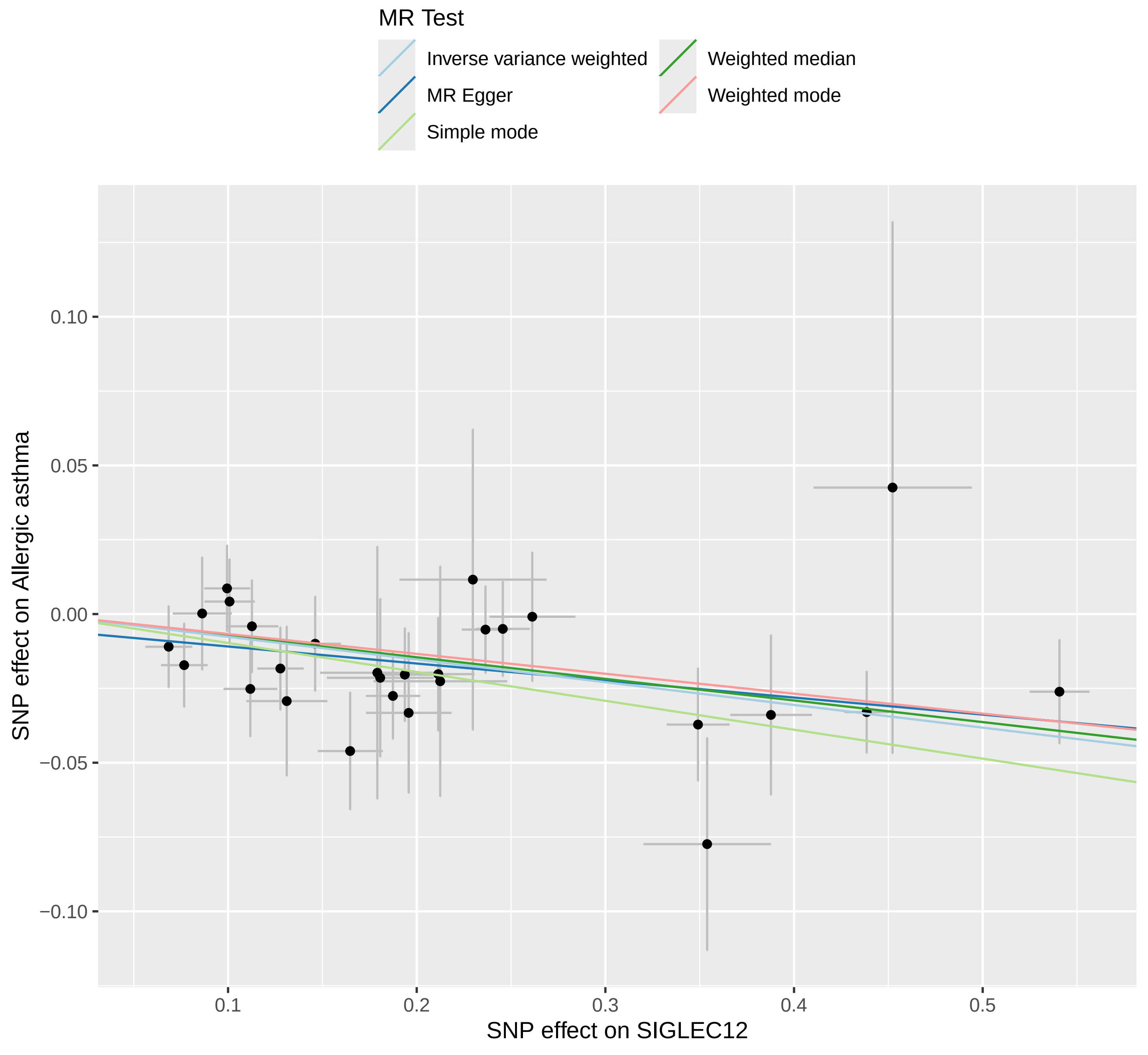
MR Test
Inverse variance weighted
MR Egger
Simple mode
Weighted median
Weighted mode
SNP effect on Allergic asthma
0.10
0.05
0.00
−0.05
−0.10
0.1
0.2
0.3
0.4
0.5
SNP effect on SIGLEC12

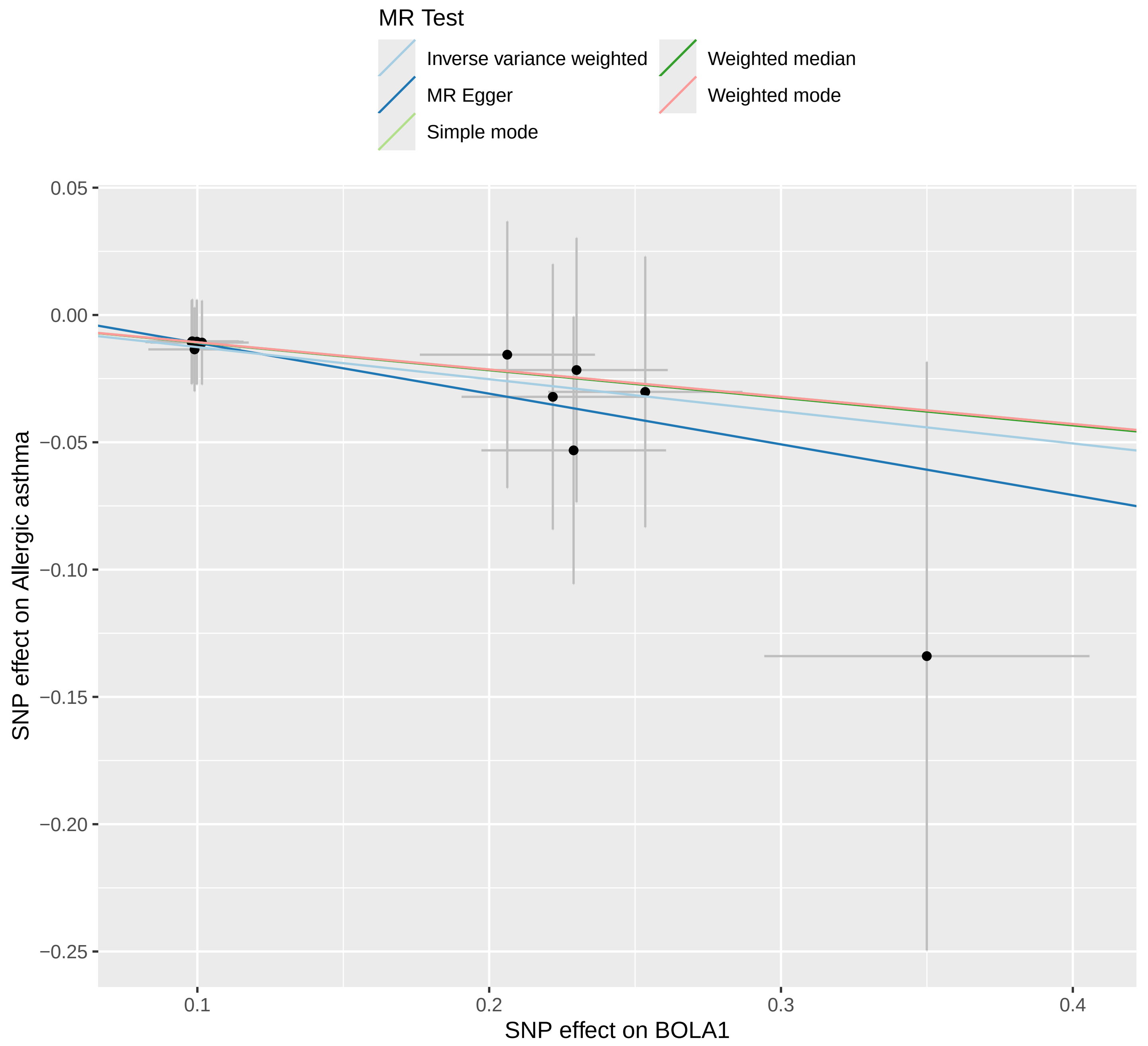
MR Test
Inverse variance weighted
MR Egger
Simple mode
Weighted median
Weighted mode
0.05
0.00
−0.05
−0.10
−0.15
−0.20
−0.25
SNP effect on Allergic asthma
0.1
0.2
0.3
0.4
SNP effect on BOLA1

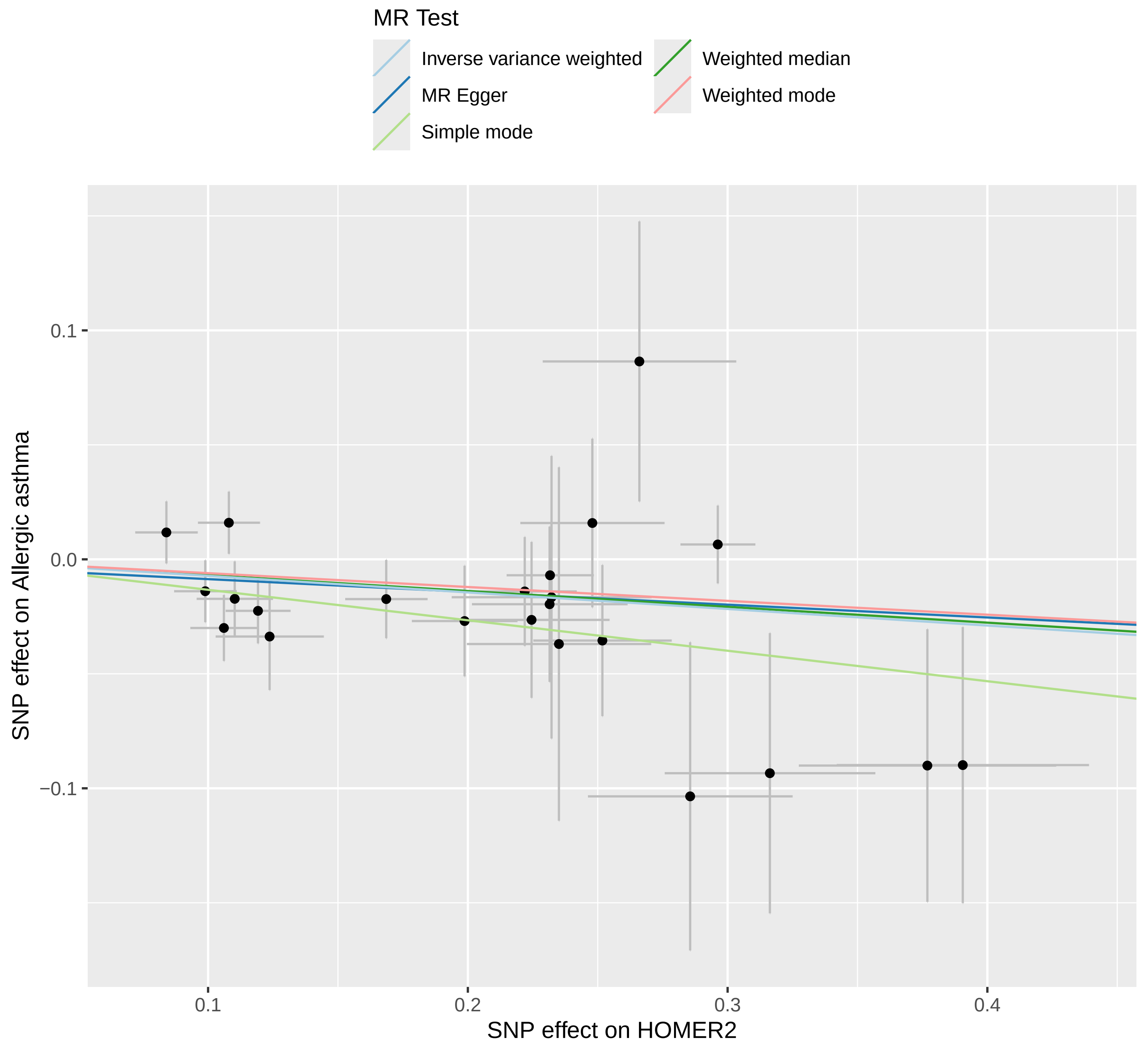
MR Test
Inverse variance weighted
MR Egger
Simple mode
Weighted median
Weighted mode
0.1
0.0
−0.1
0.1
0.2
0.3
0.4
SNP effect on HOMER2
SNP effect on Allergic asthma

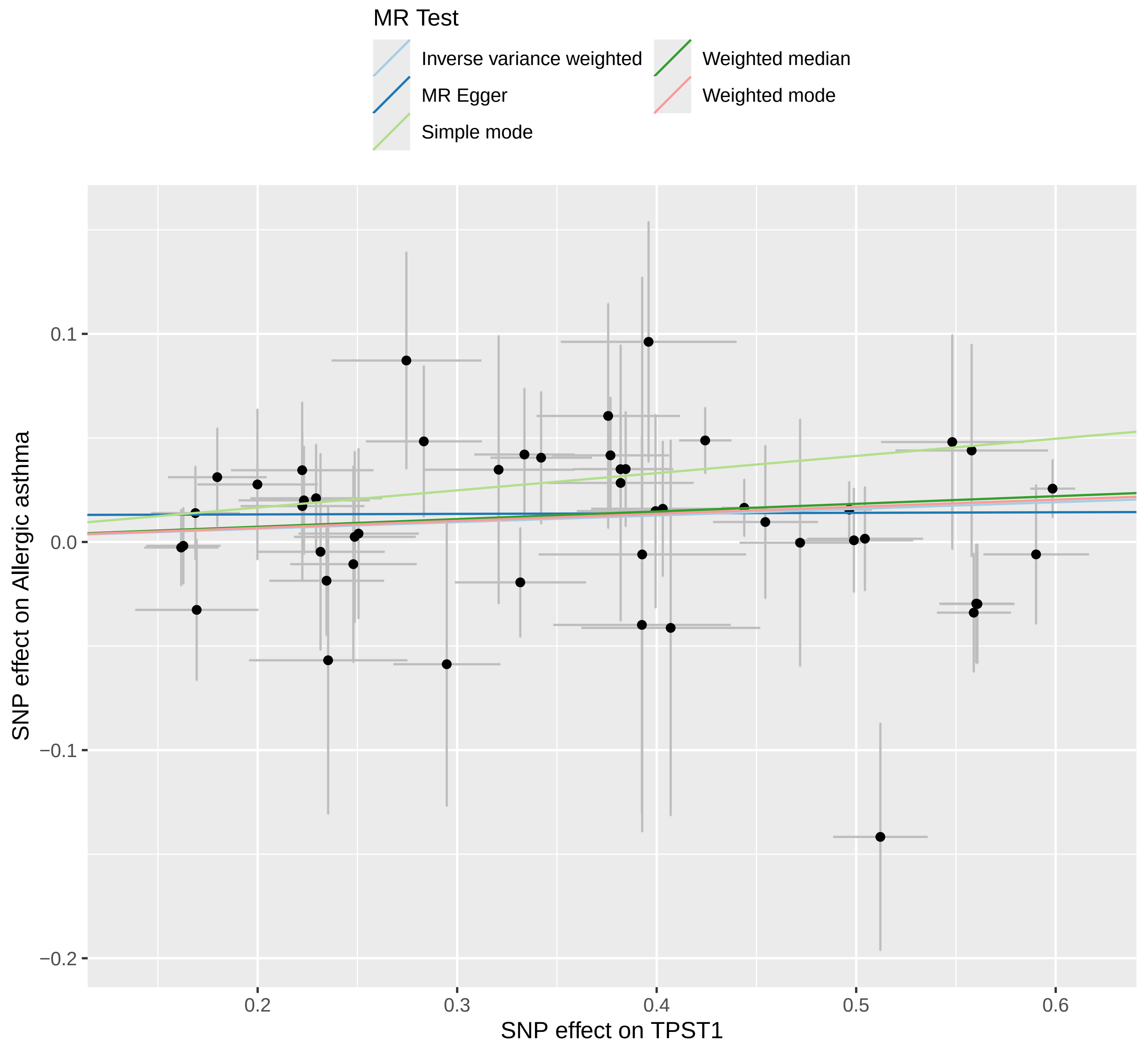
MR Test
Inverse variance weighted
MR Egger
Simple mode
Weighted median
Weighted mode
SNP effect on Allergic asthma
0.1
0.0
−0.1
−0.2
0.2
0.3
0.4
0.5
0.6
SNP effect on TPST1
