# Supplementary figures and images for: Mediating Mendelian randomization in the proteome identified potential drug targets for obesity-related allergic asthma
Source: Hereditas. 2025 Feb 1;162:12. doi: 10.1186/s41065-025-00376-w (PMC11786417; doi:10.1186/s41065-025-00376-w)

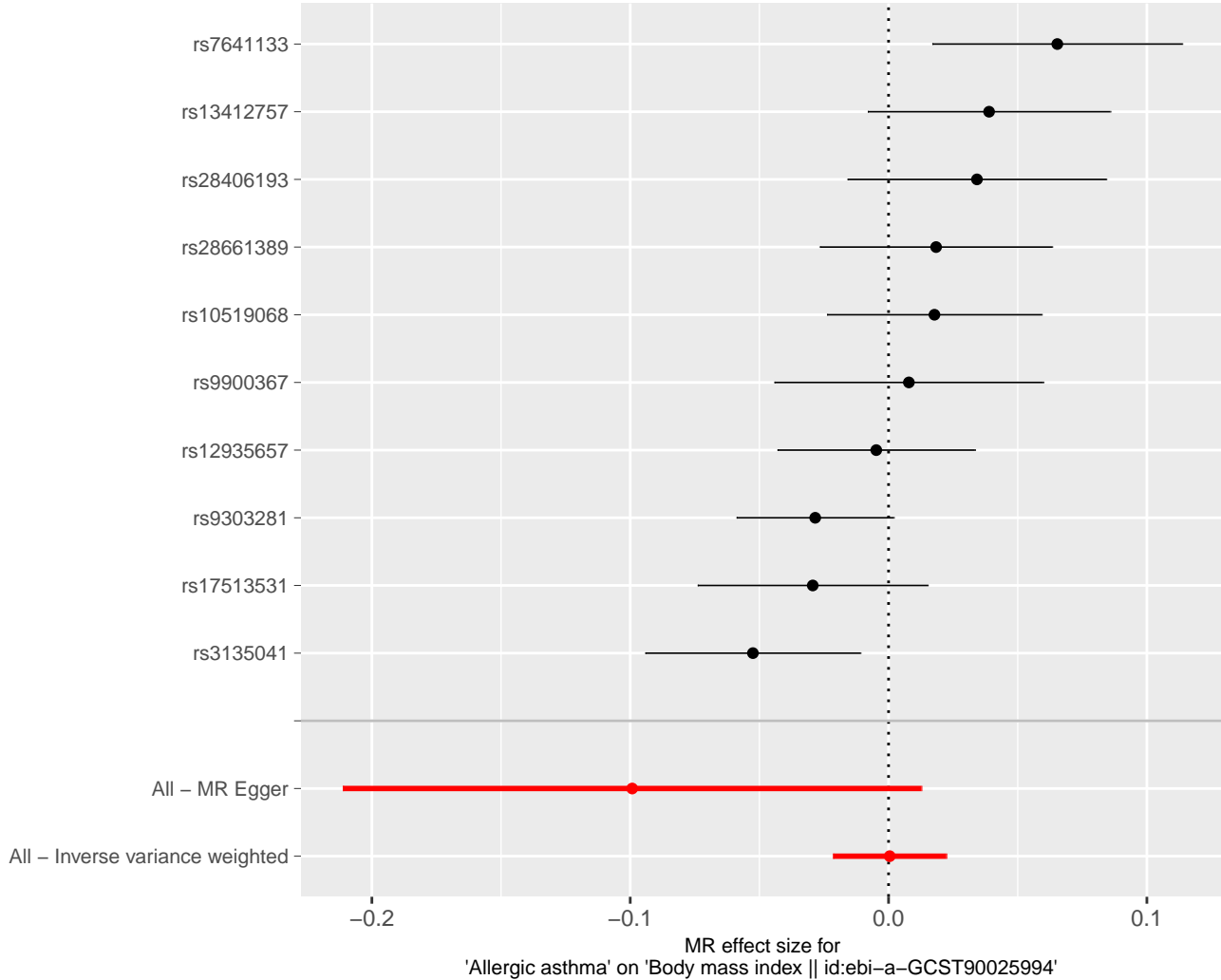

Supplement: Supplementary file 1 — Supplementary Material 1: Supplementary Materials 1. The MR results of BMI and allergic asthma. Supplementary Materials 2. The MR results of plasma proteins and allergic asthma. Supplementary Materials 3. The MR results of BMI and plasma proteins. Supplementary Materials 4. The MR results of mediation factor plasma proteins and allergic asthma. Supplementary Materials 5. The results of drug-targeted MR [file 41065_2025_376_MOESM1_ESM.zip › Supplementary Materials/S1 The MR results of BMI and allergic asthma/S1 Allergic asthma-BMI.forest.pdf]

# MR Method

- Inverse variance weighted
- MR Egger

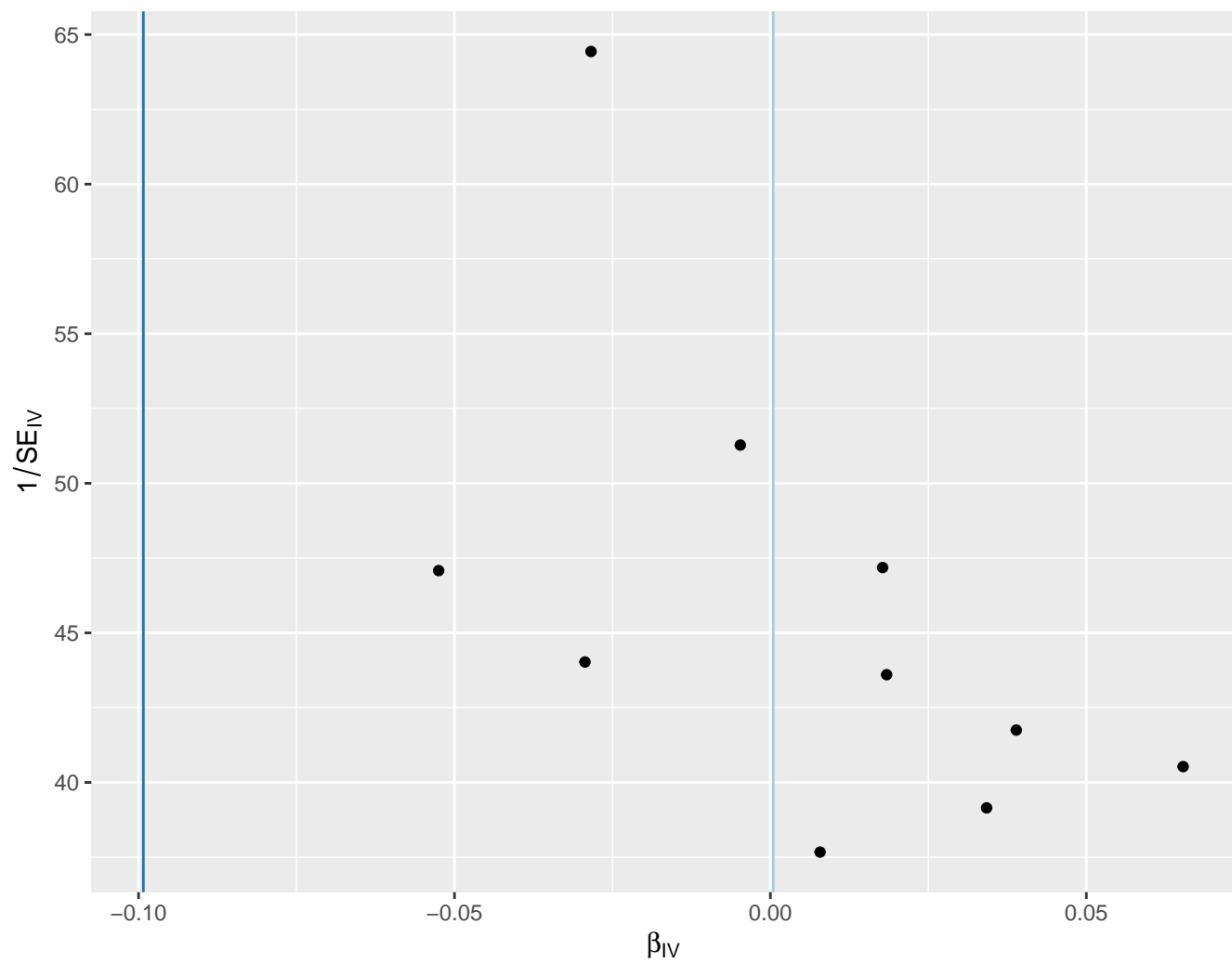

Supplement: Supplementary file 1 — Supplementary Material 1: Supplementary Materials 1. The MR results of BMI and allergic asthma. Supplementary Materials 2. The MR results of plasma proteins and allergic asthma. Supplementary Materials 3. The MR results of BMI and plasma proteins. Supplementary Materials 4. The MR results of mediation factor plasma proteins and allergic asthma. Supplementary Materials 5. The results of drug-targeted MR [file 41065_2025_376_MOESM1_ESM.zip › Supplementary Materials/S1 The MR results of BMI and allergic asthma/S1 Allergic asthma-BMI.funnel_plot.pdf]

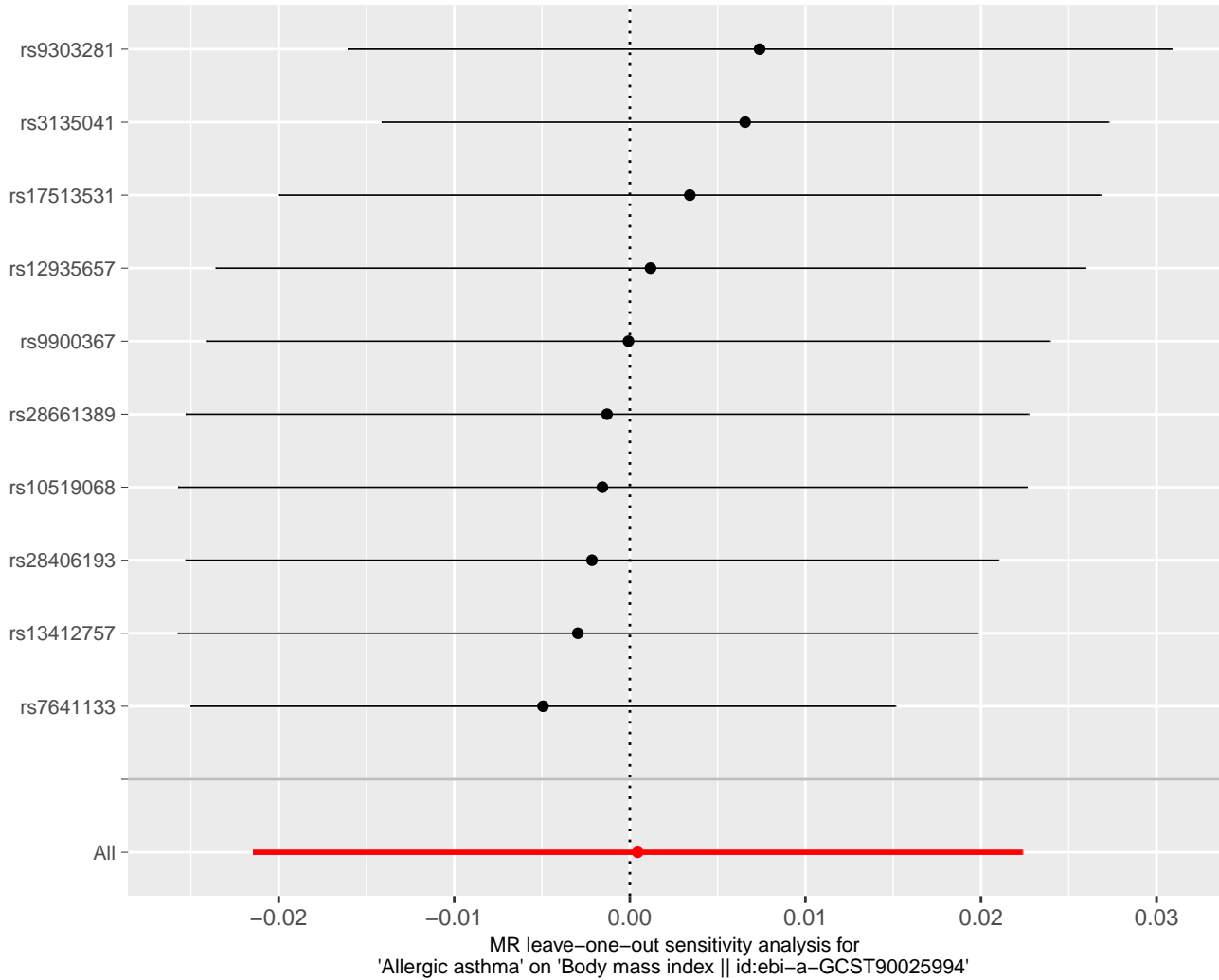

Supplement: Supplementary file 1 — Supplementary Material 1: Supplementary Materials 1. The MR results of BMI and allergic asthma. Supplementary Materials 2. The MR results of plasma proteins and allergic asthma. Supplementary Materials 3. The MR results of BMI and plasma proteins. Supplementary Materials 4. The MR results of mediation factor plasma proteins and allergic asthma. Supplementary Materials 5. The results of drug-targeted MR [file 41065_2025_376_MOESM1_ESM.zip › Supplementary Materials/S1 The MR results of BMI and allergic asthma/S1 Allergic asthma-BMI.leaveoneout.pdf]

All – Inverse variance weights

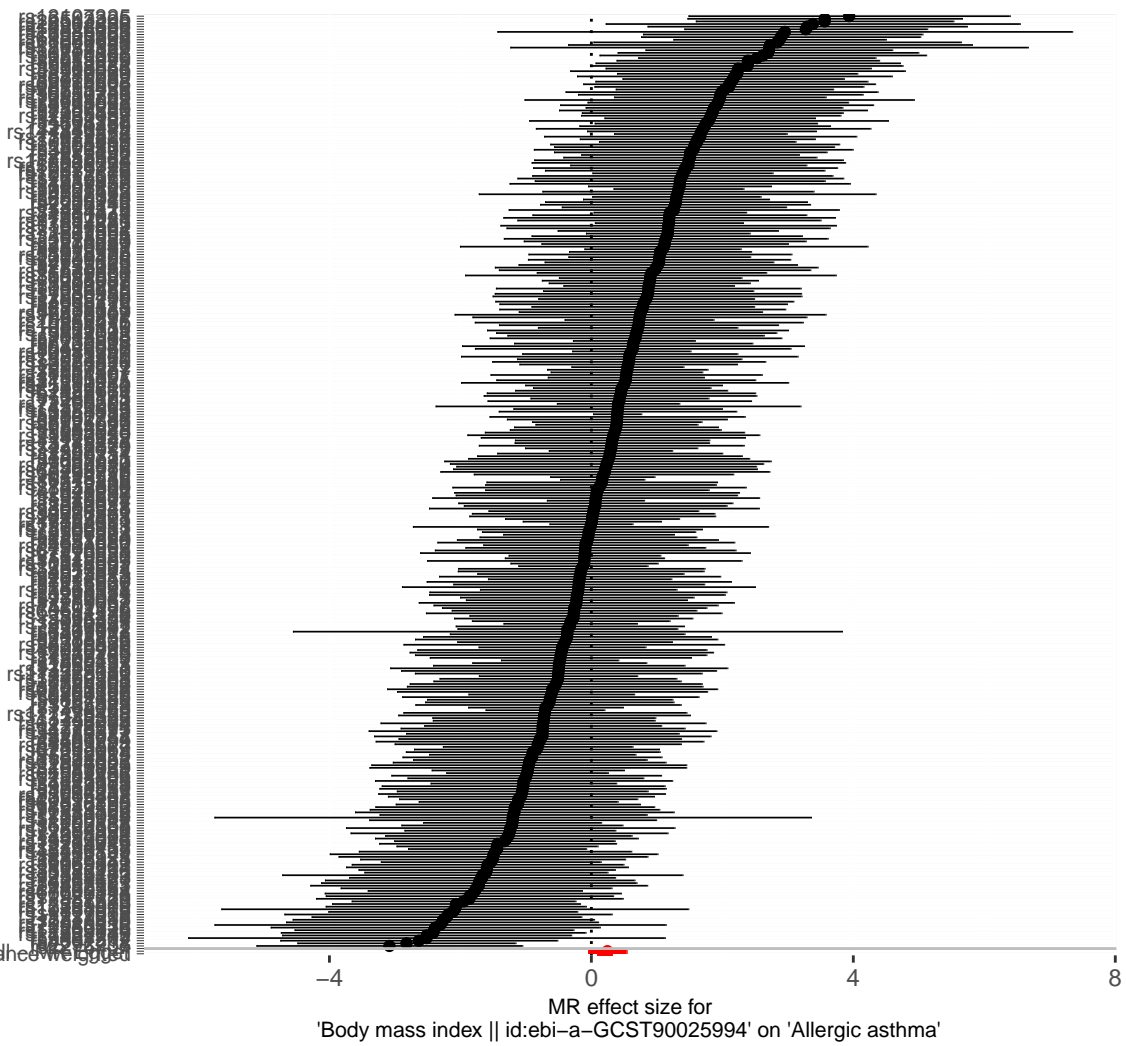

Supplement: Supplementary file 1 — Supplementary Material 1: Supplementary Materials 1. The MR results of BMI and allergic asthma. Supplementary Materials 2. The MR results of plasma proteins and allergic asthma. Supplementary Materials 3. The MR results of BMI and plasma proteins. Supplementary Materials 4. The MR results of mediation factor plasma proteins and allergic asthma. Supplementary Materials 5. The results of drug-targeted MR [file 41065_2025_376_MOESM1_ESM.zip › Supplementary Materials/S1 The MR results of BMI and allergic asthma/S1 BMI-Allergic asthma.forest.pdf]

# MR Method

- Inverse variance weighted
- MR Egger

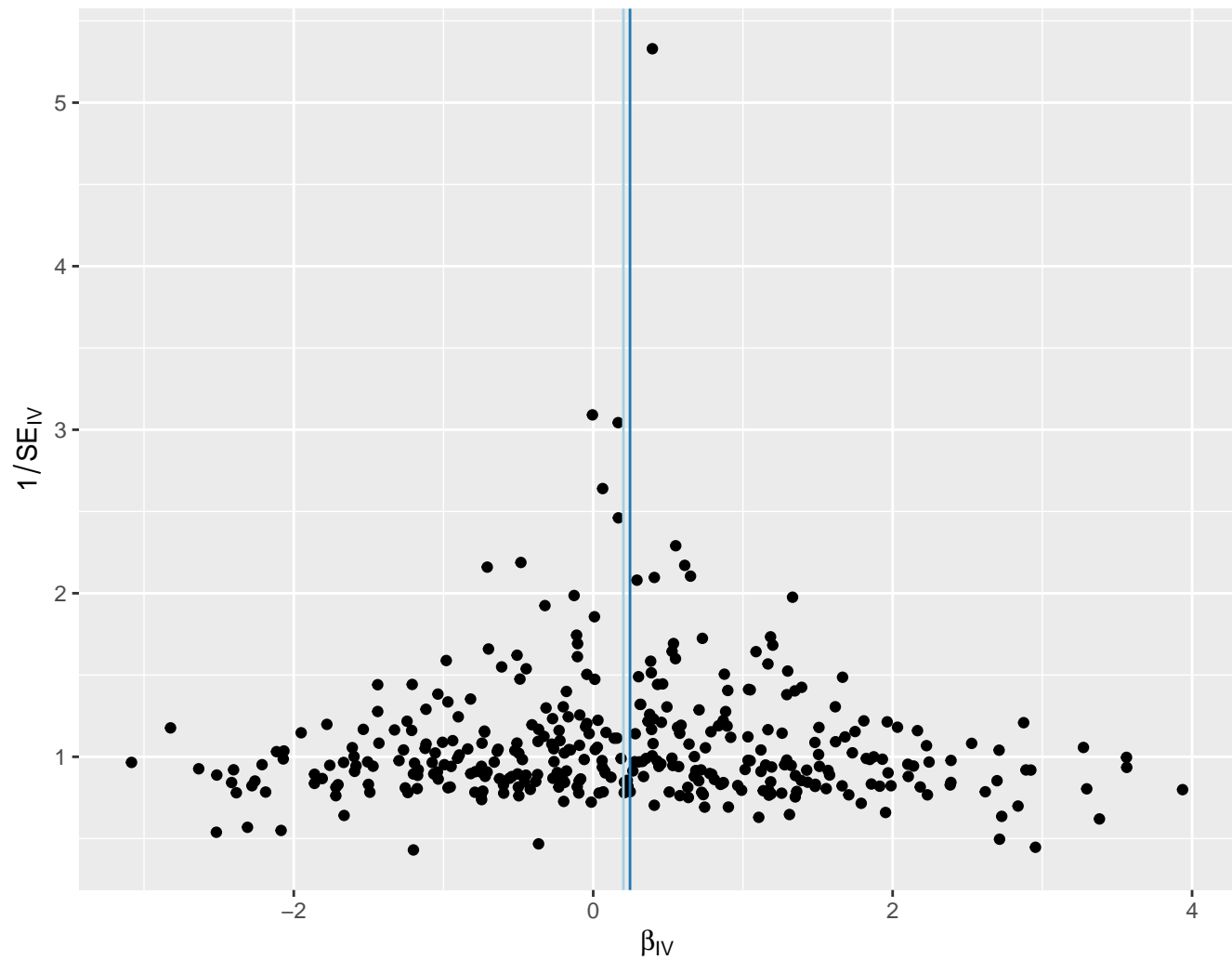

Supplement: Supplementary file 1 — Supplementary Material 1: Supplementary Materials 1. The MR results of BMI and allergic asthma. Supplementary Materials 2. The MR results of plasma proteins and allergic asthma. Supplementary Materials 3. The MR results of BMI and plasma proteins. Supplementary Materials 4. The MR results of mediation factor plasma proteins and allergic asthma. Supplementary Materials 5. The results of drug-targeted MR [file 41065_2025_376_MOESM1_ESM.zip › Supplementary Materials/S1 The MR results of BMI and allergic asthma/S1 BMI-Allergic asthma.funnel_plot.pdf]

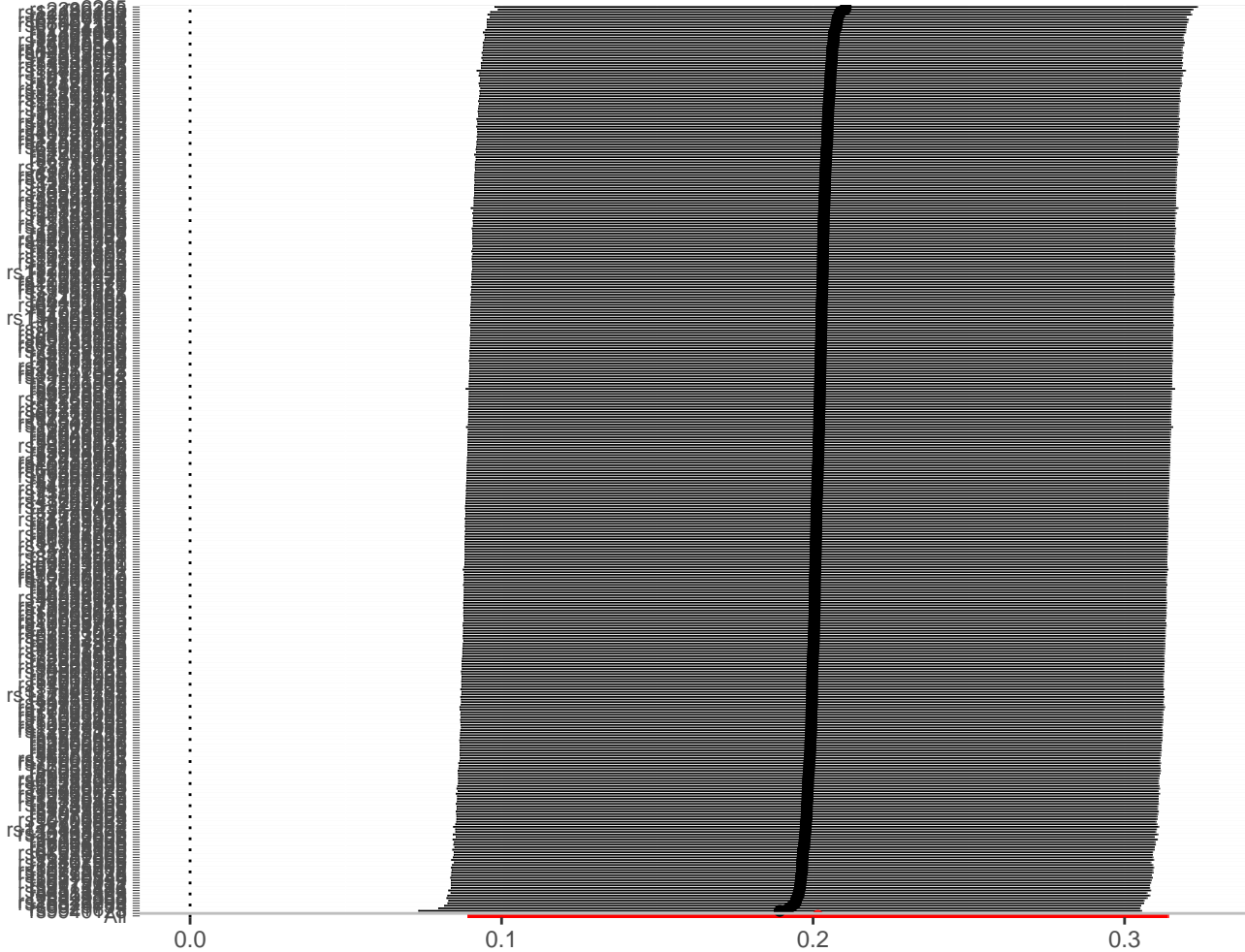

MR leave-one-out sensitivity analysis for  
'Body mass index || id:ebi-a-GCST90025994' on 'Allergic asthma'

Supplement: Supplementary file 1 — Supplementary Material 1: Supplementary Materials 1. The MR results of BMI and allergic asthma. Supplementary Materials 2. The MR results of plasma proteins and allergic asthma. Supplementary Materials 3. The MR results of BMI and plasma proteins. Supplementary Materials 4. The MR results of mediation factor plasma proteins and allergic asthma. Supplementary Materials 5. The results of drug-targeted MR [file 41065_2025_376_MOESM1_ESM.zip › Supplementary Materials/S1 The MR results of BMI and allergic asthma/S1 BMI-Allergic asthma.leaveoneout.pdf]

# MR Test

- Inverse variance weighted
- MR Egger
- Simple mode
- Weighted median
- Weighted mode

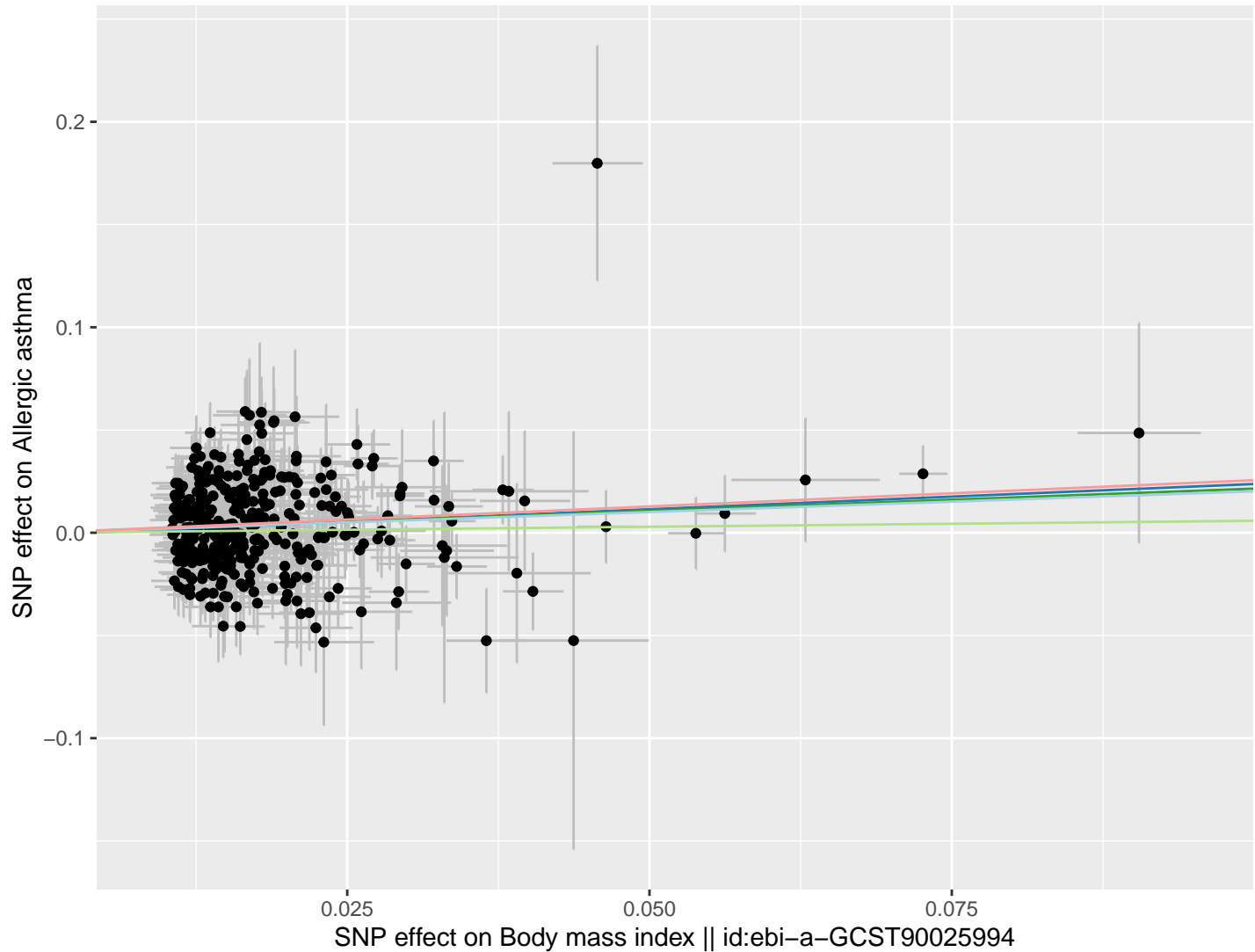

Supplement: Supplementary file 1 — Supplementary Material 1: Supplementary Materials 1. The MR results of BMI and allergic asthma. Supplementary Materials 2. The MR results of plasma proteins and allergic asthma. Supplementary Materials 3. The MR results of BMI and plasma proteins. Supplementary Materials 4. The MR results of mediation factor plasma proteins and allergic asthma. Supplementary Materials 5. The results of drug-targeted MR [file 41065_2025_376_MOESM1_ESM.zip › Supplementary Materials/S1 The MR results of BMI and allergic asthma/S1 BMI-Allergic asthma.scatter_plot.pdf]

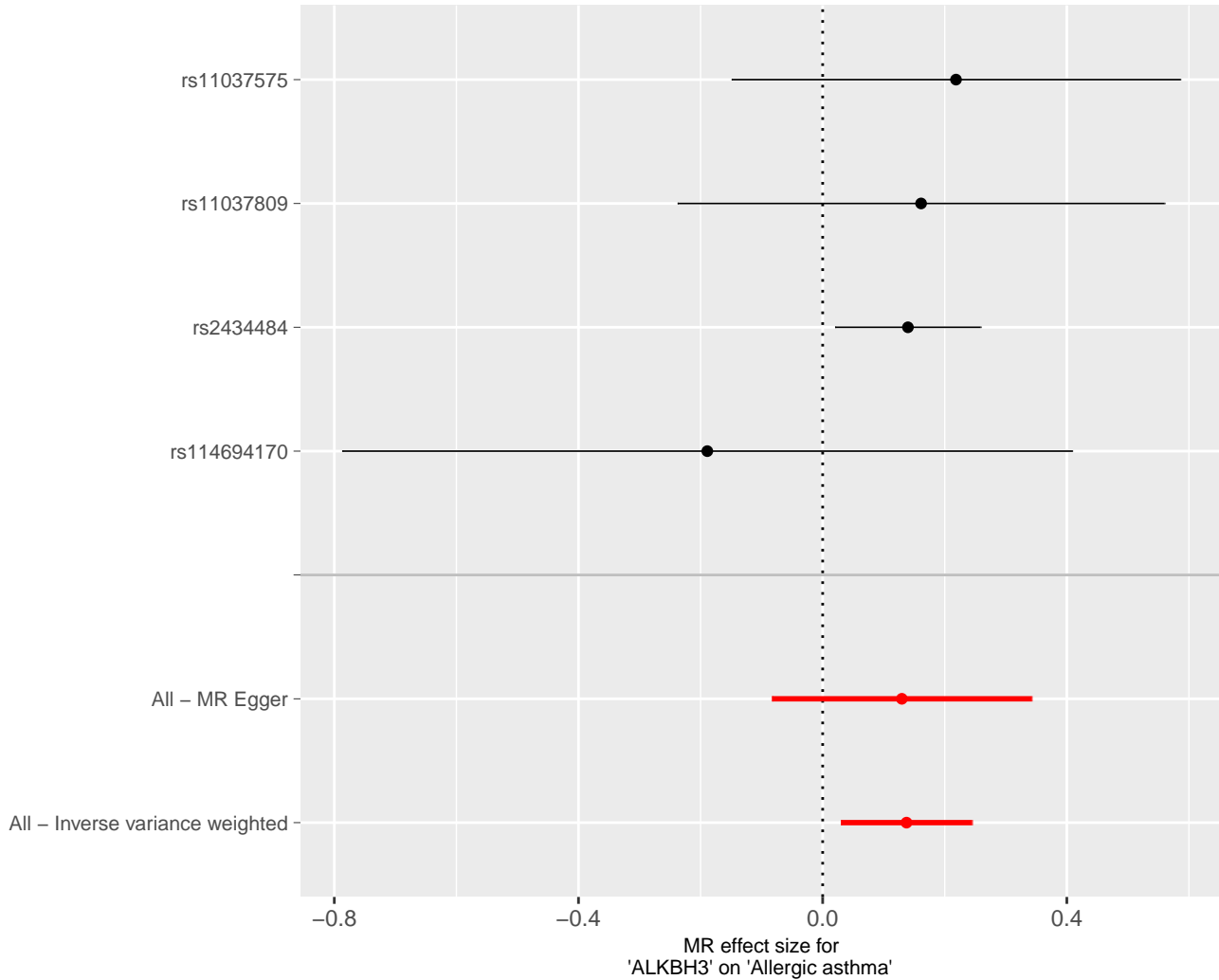

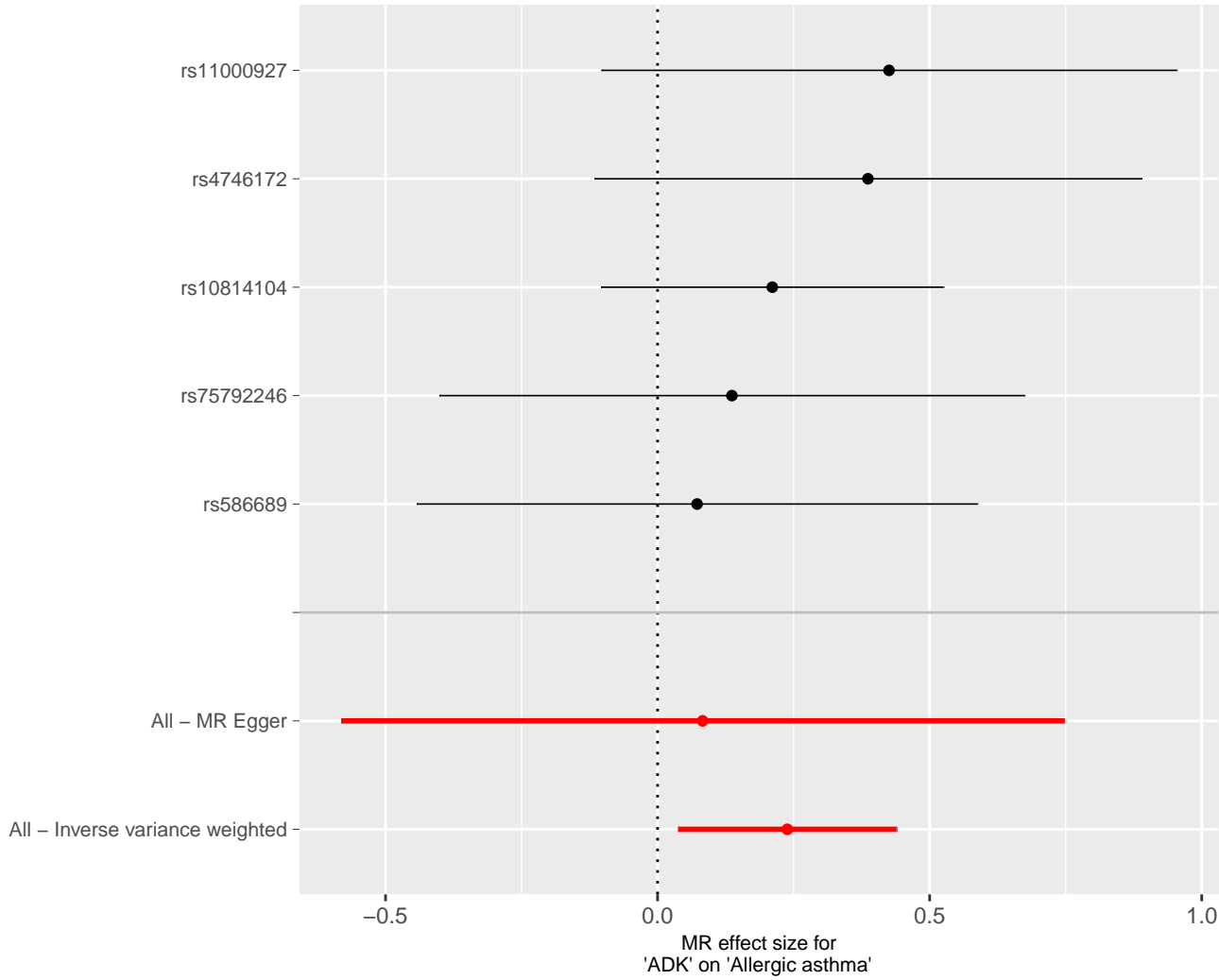

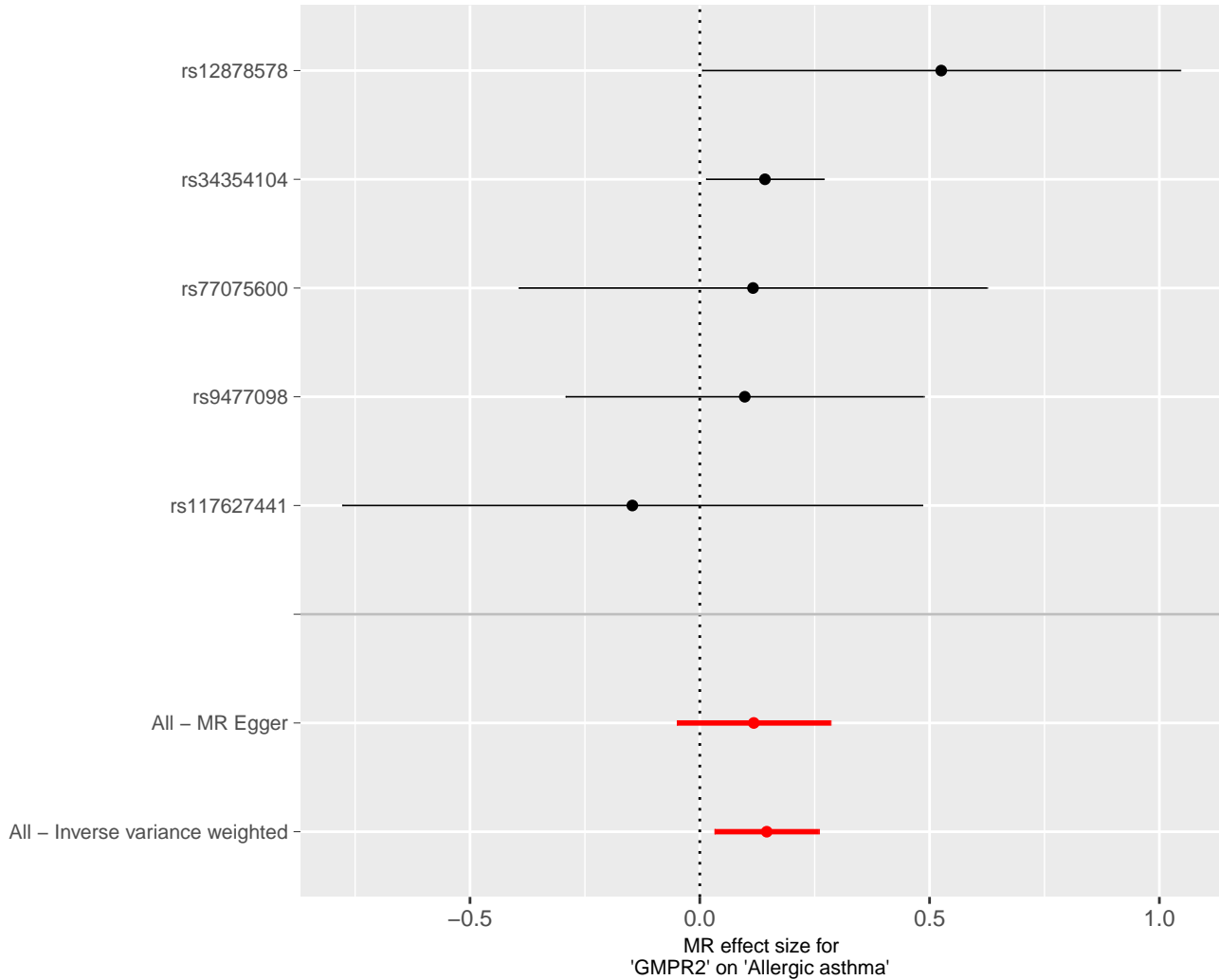

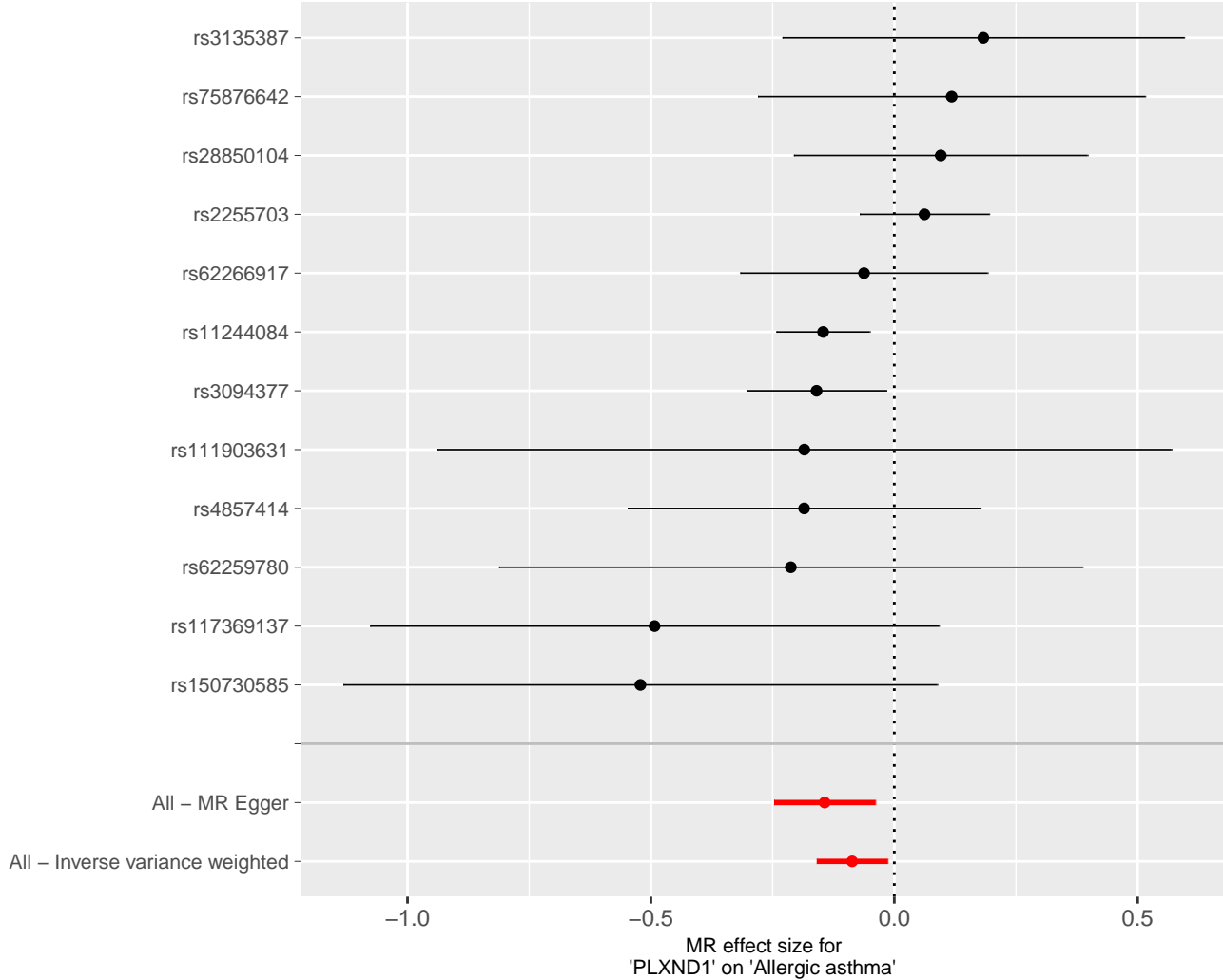

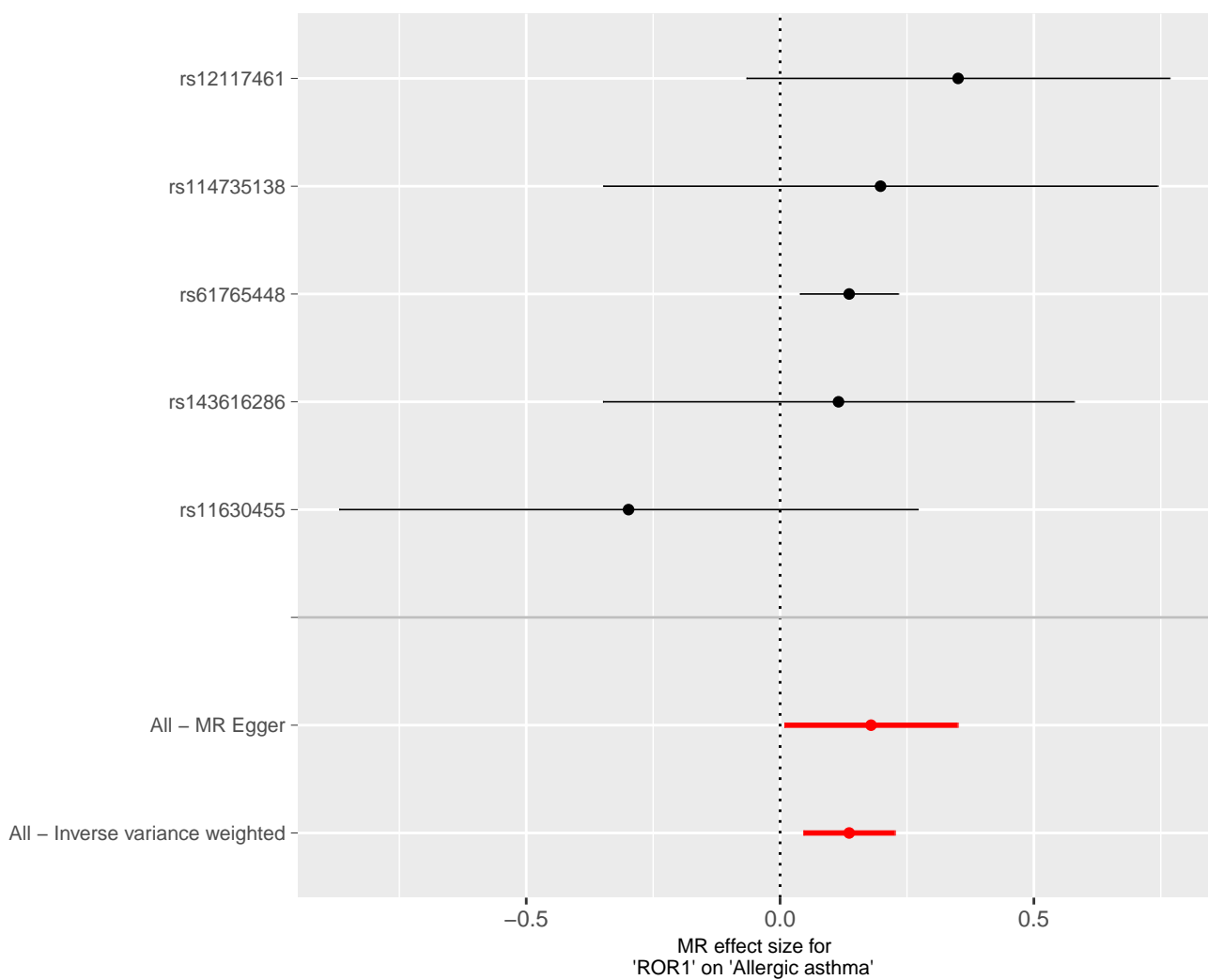

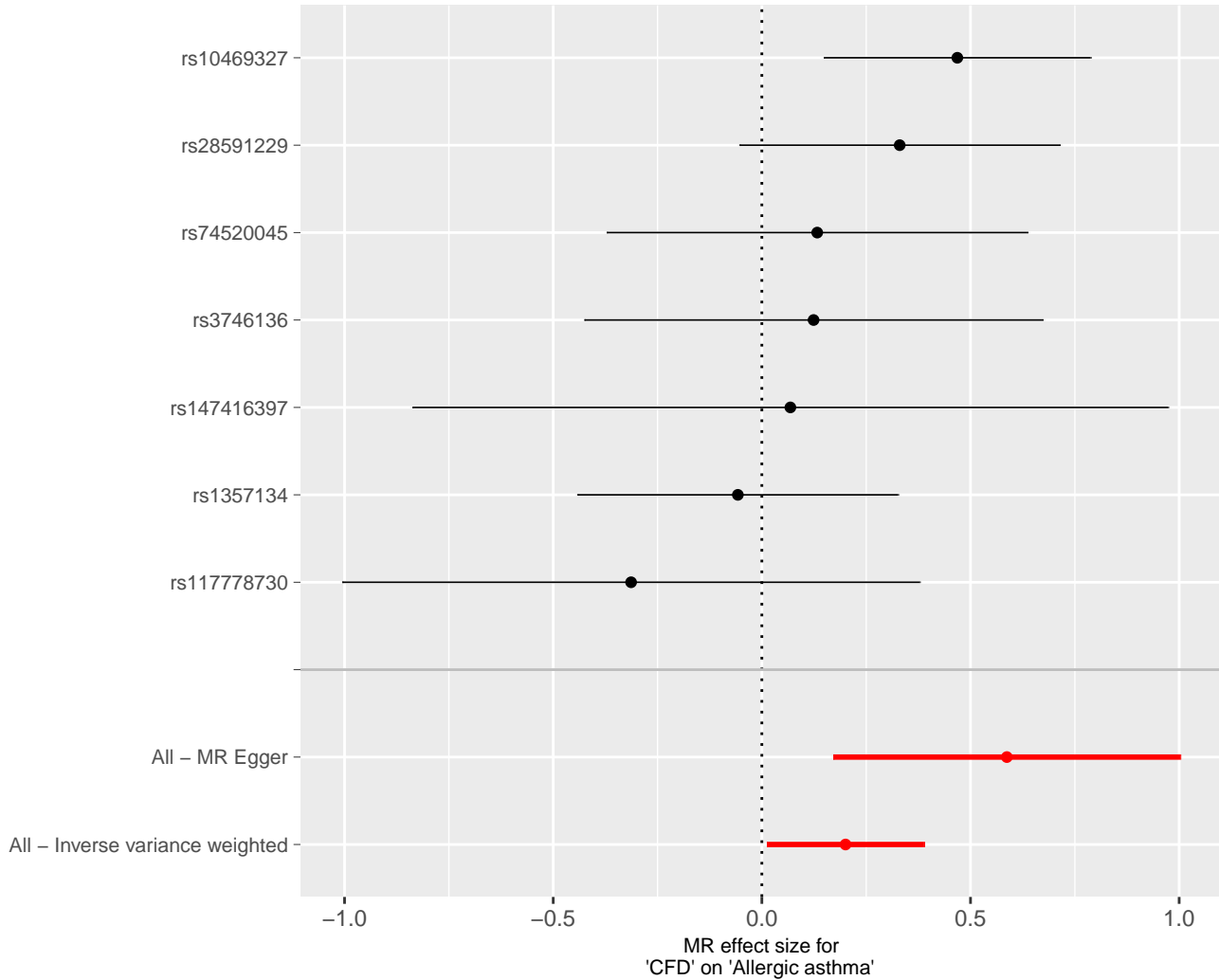

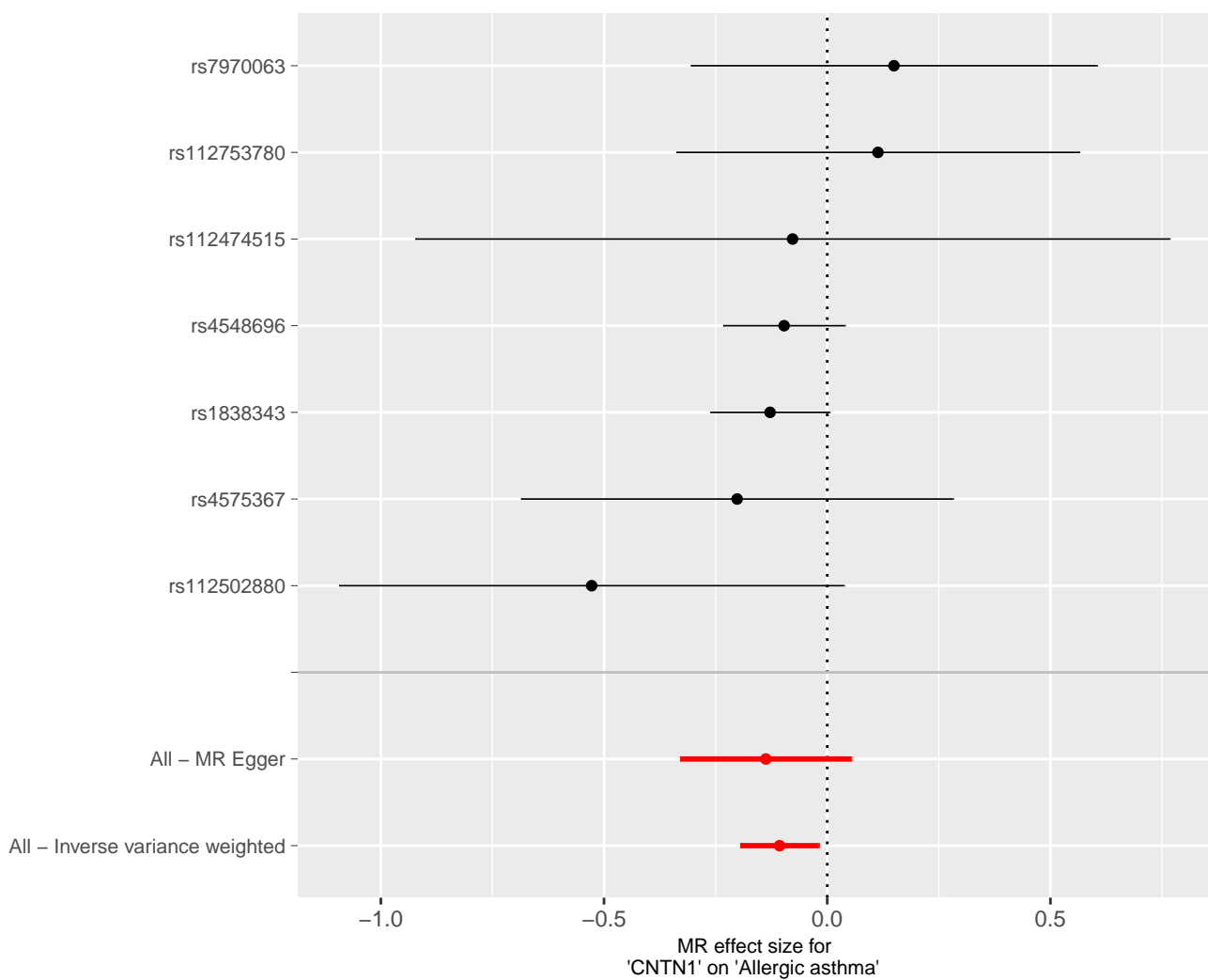

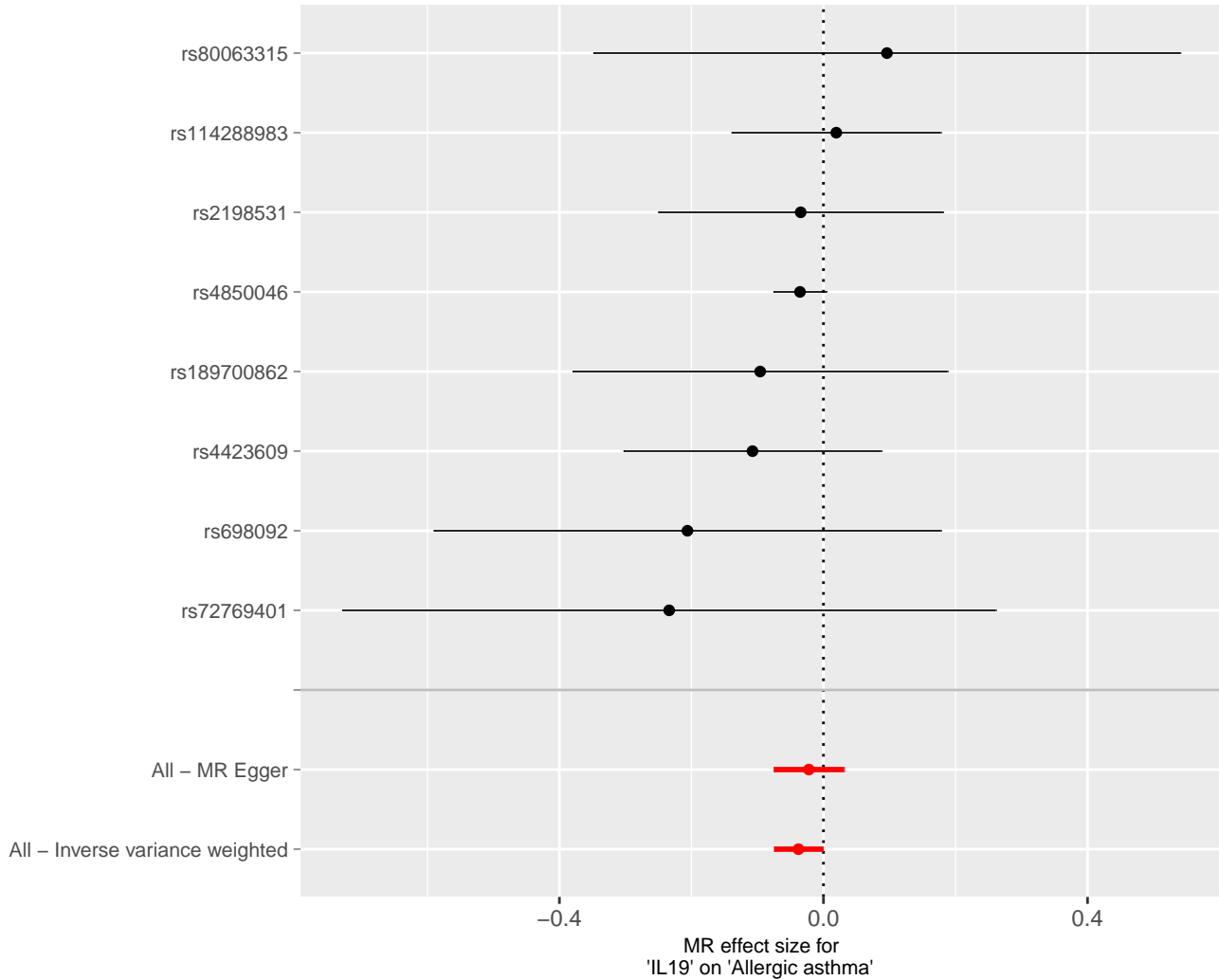

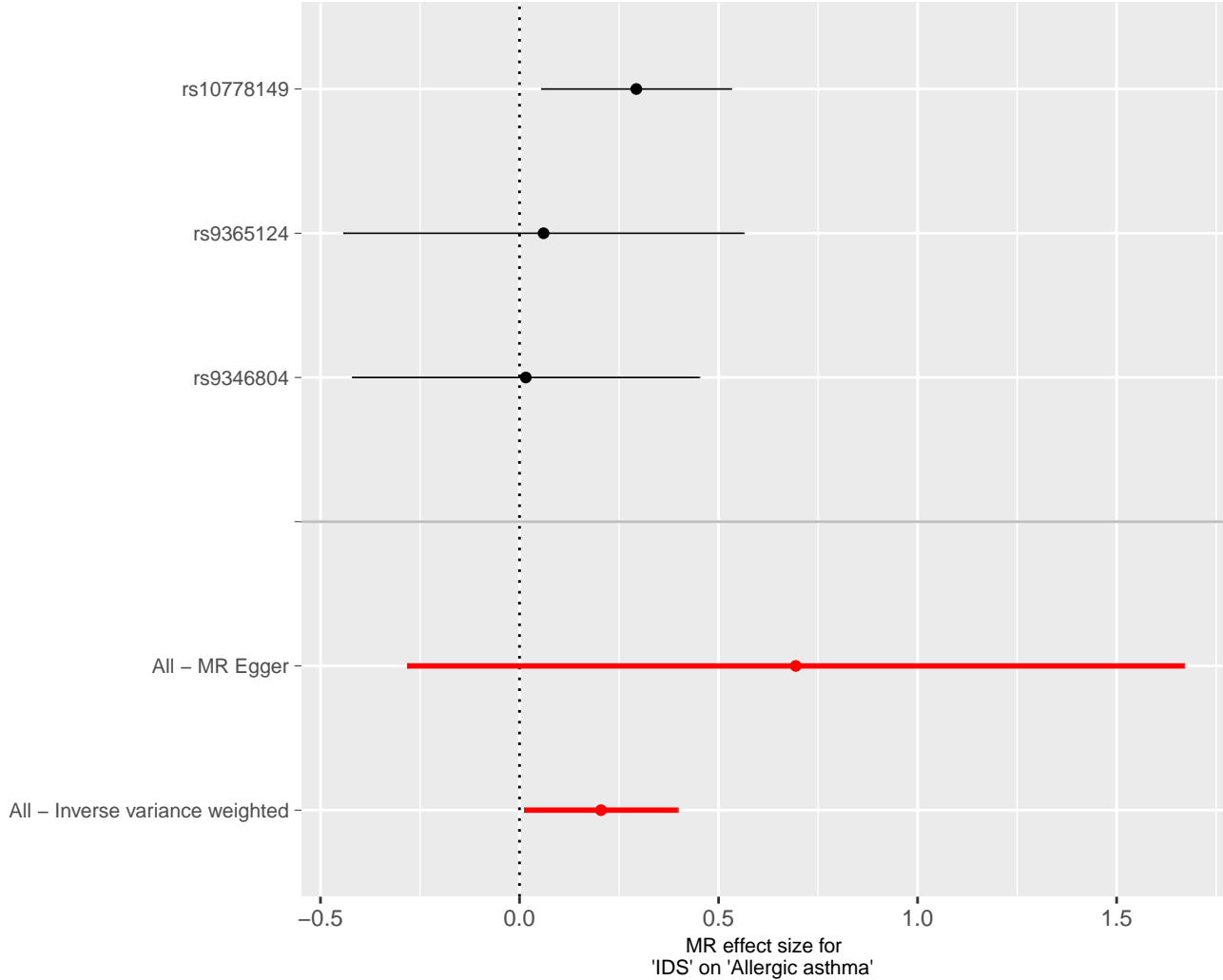

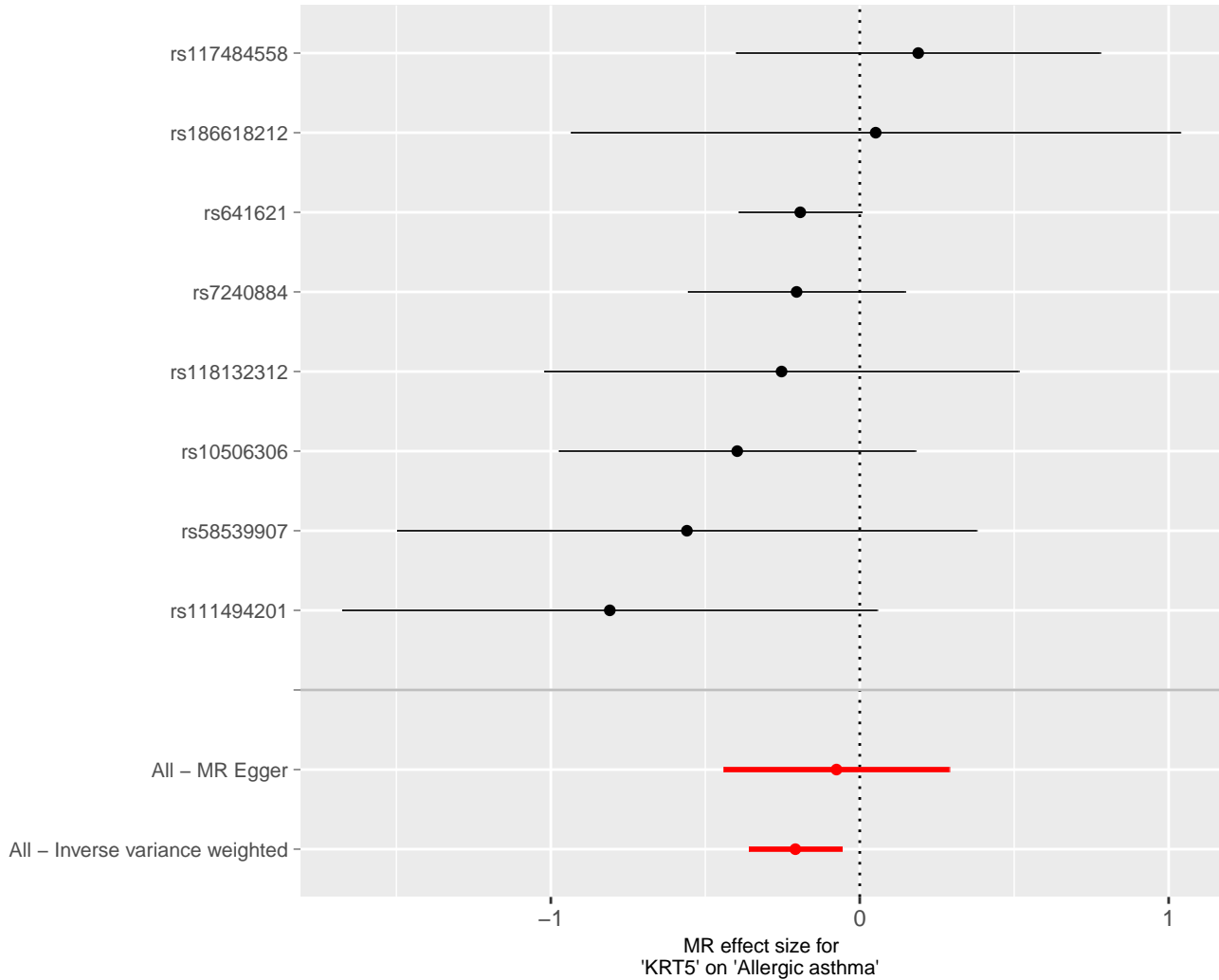

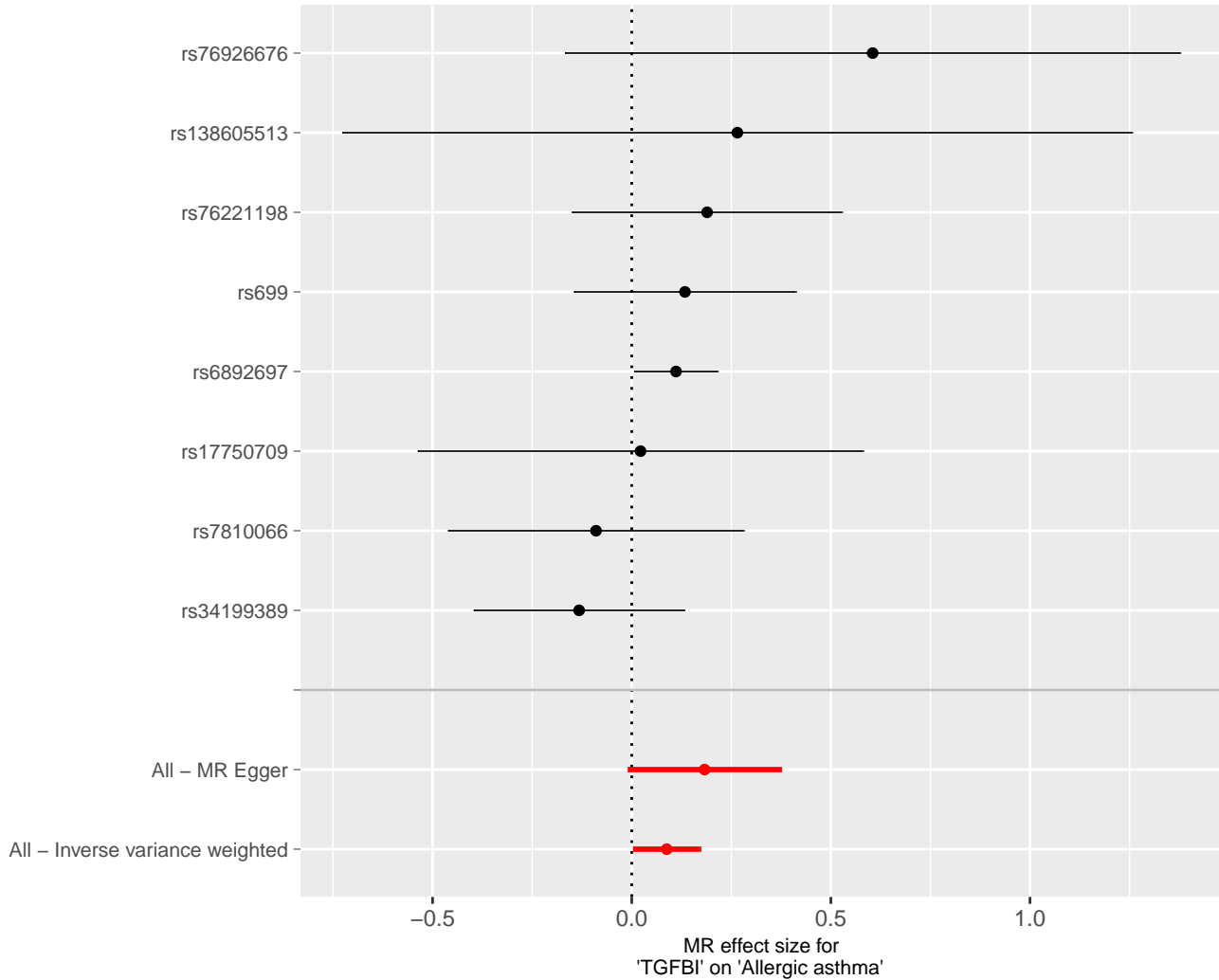

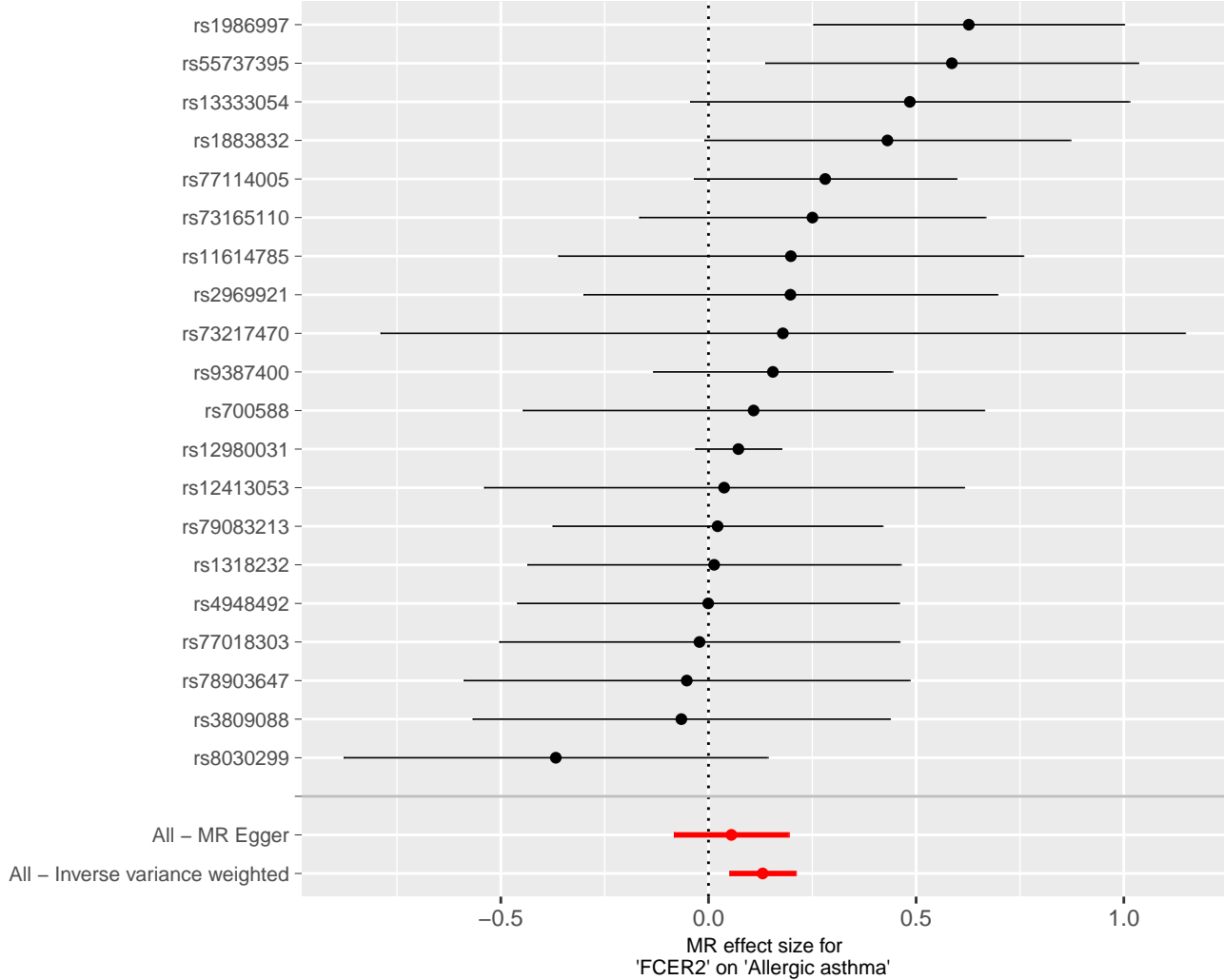

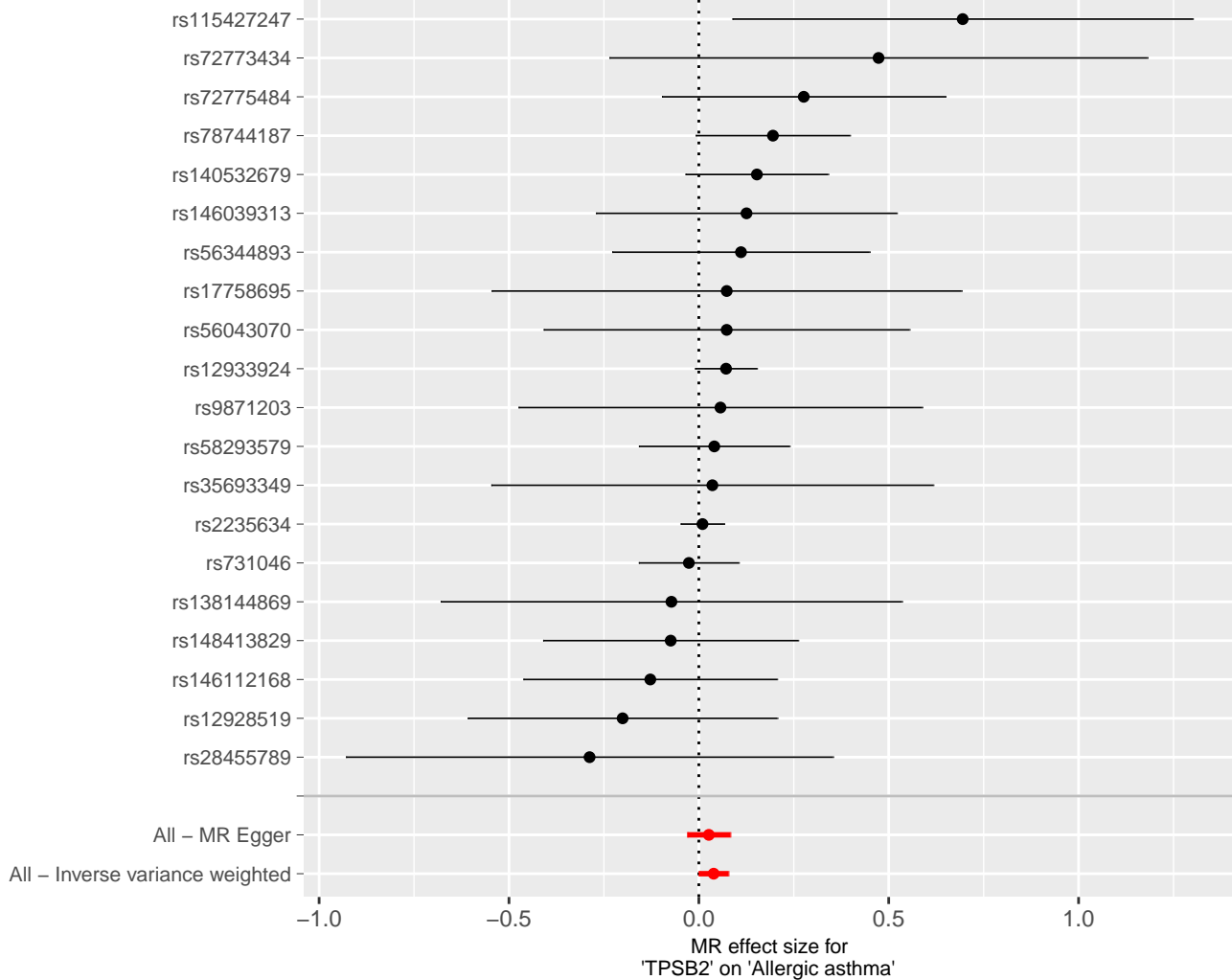

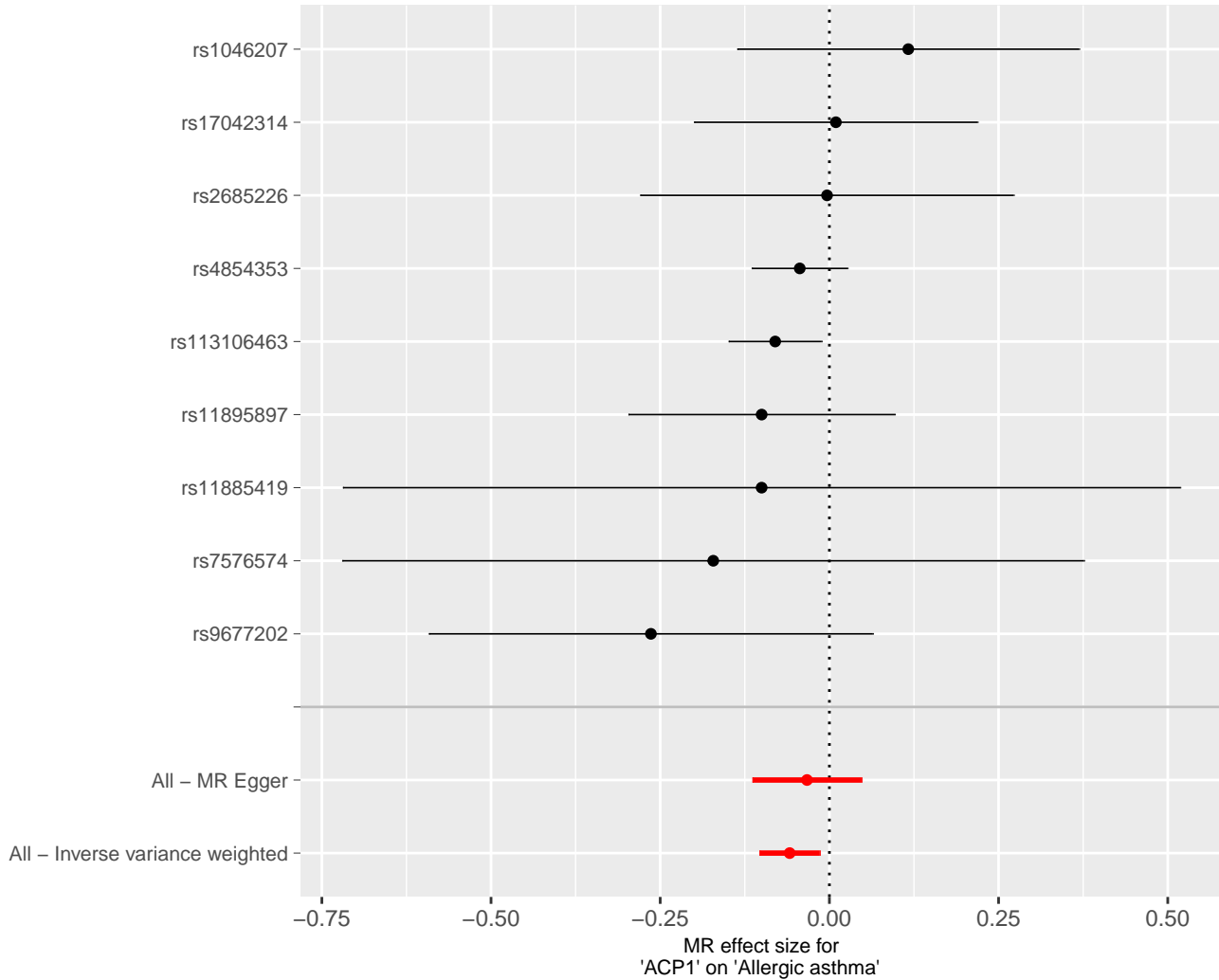

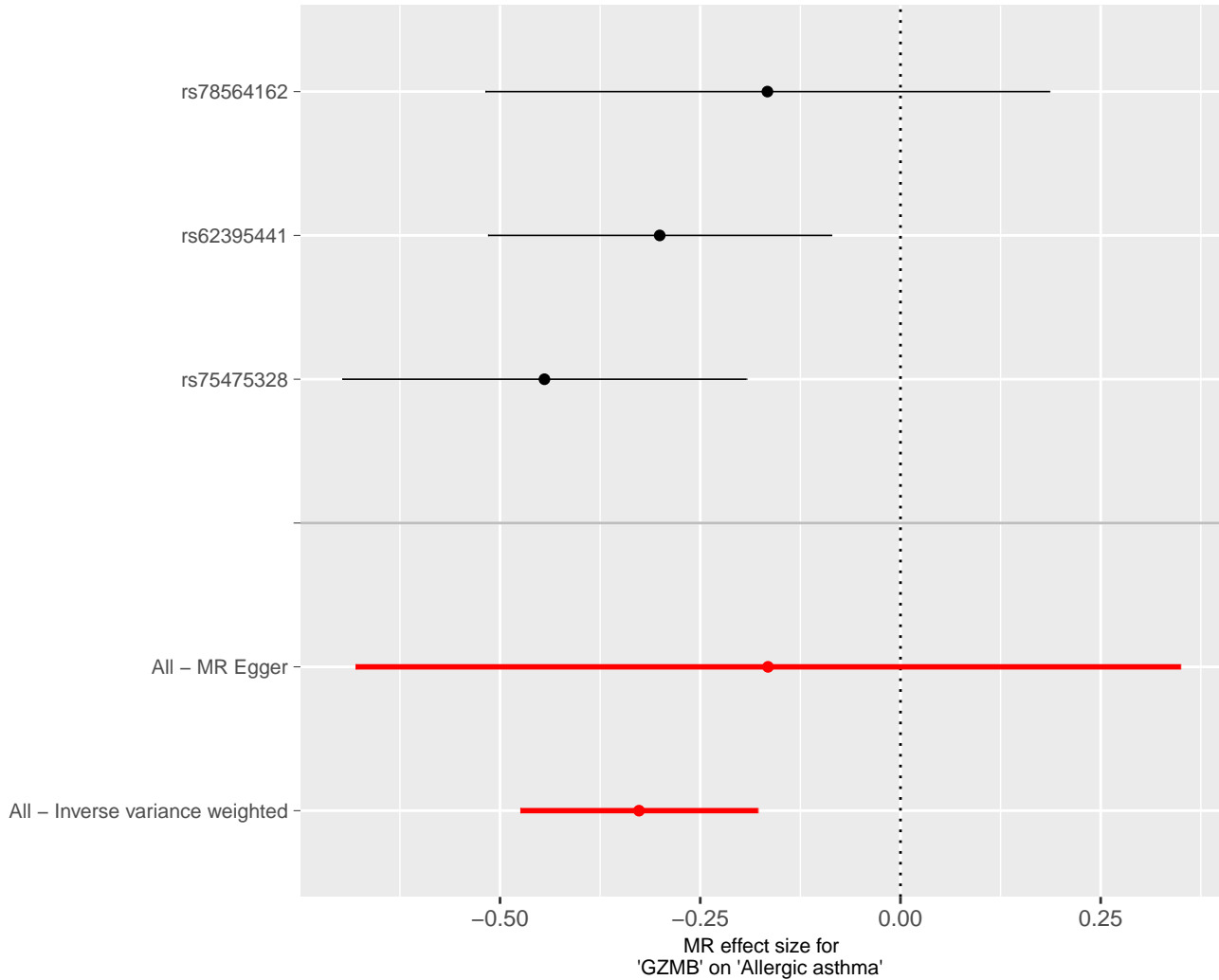

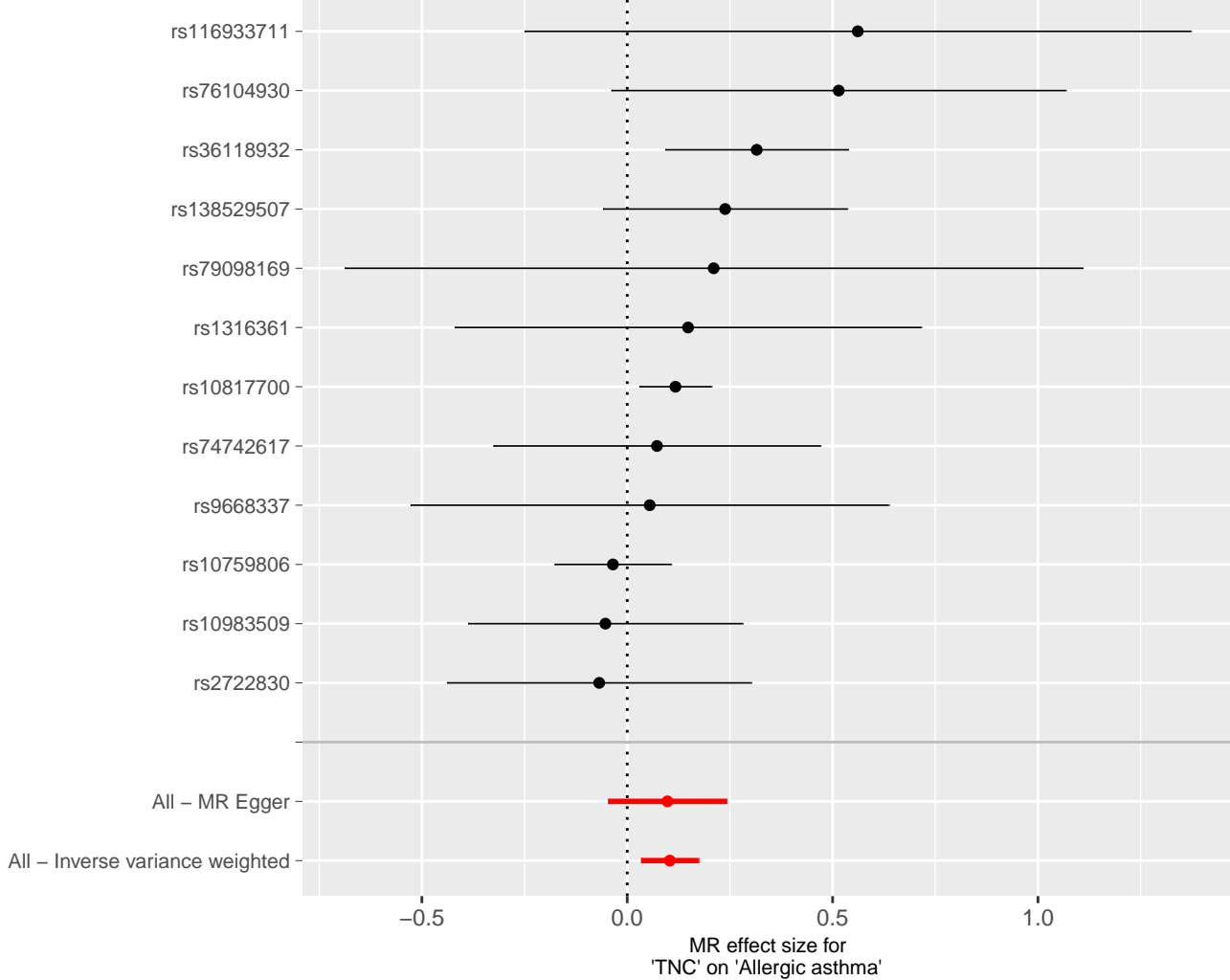

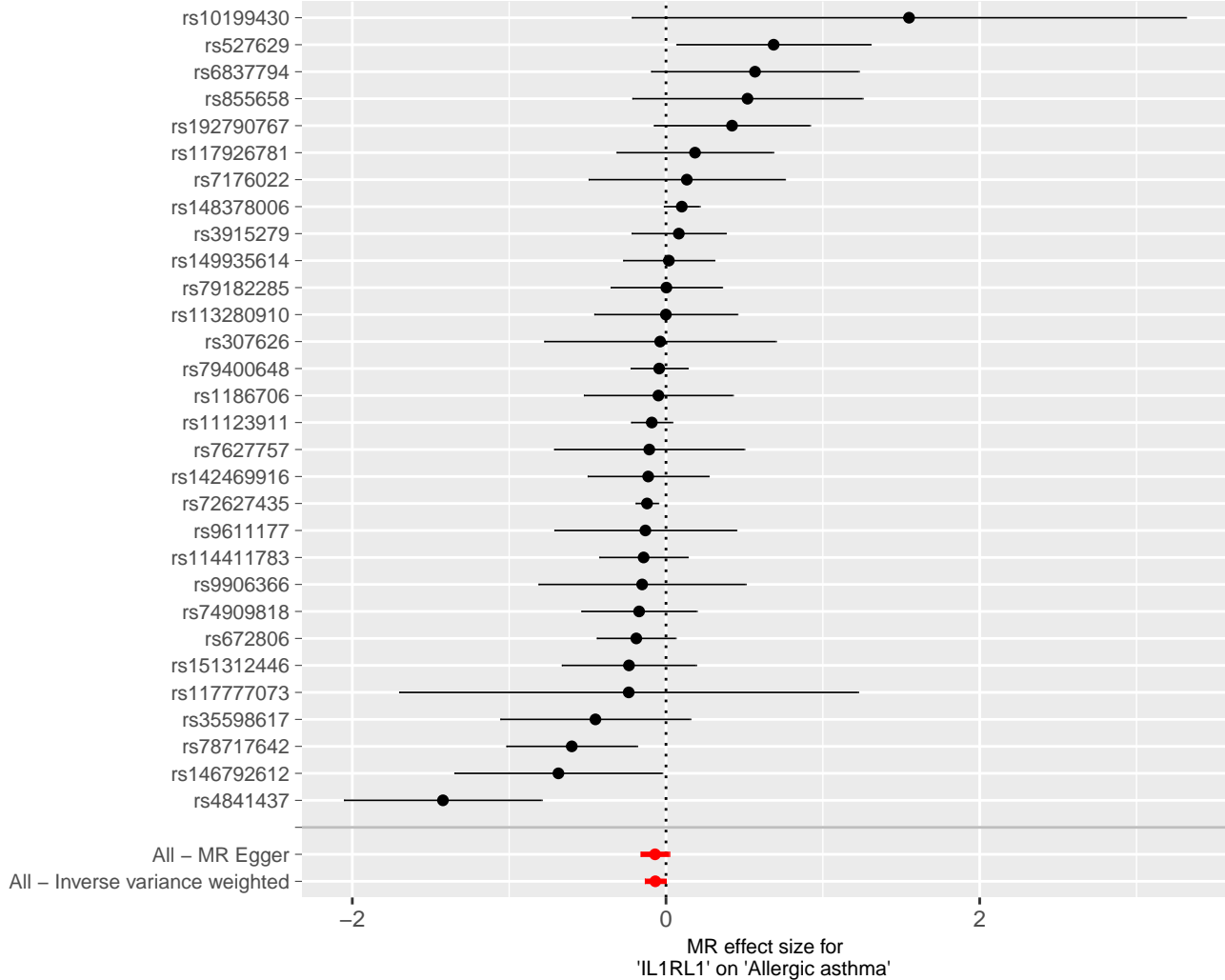

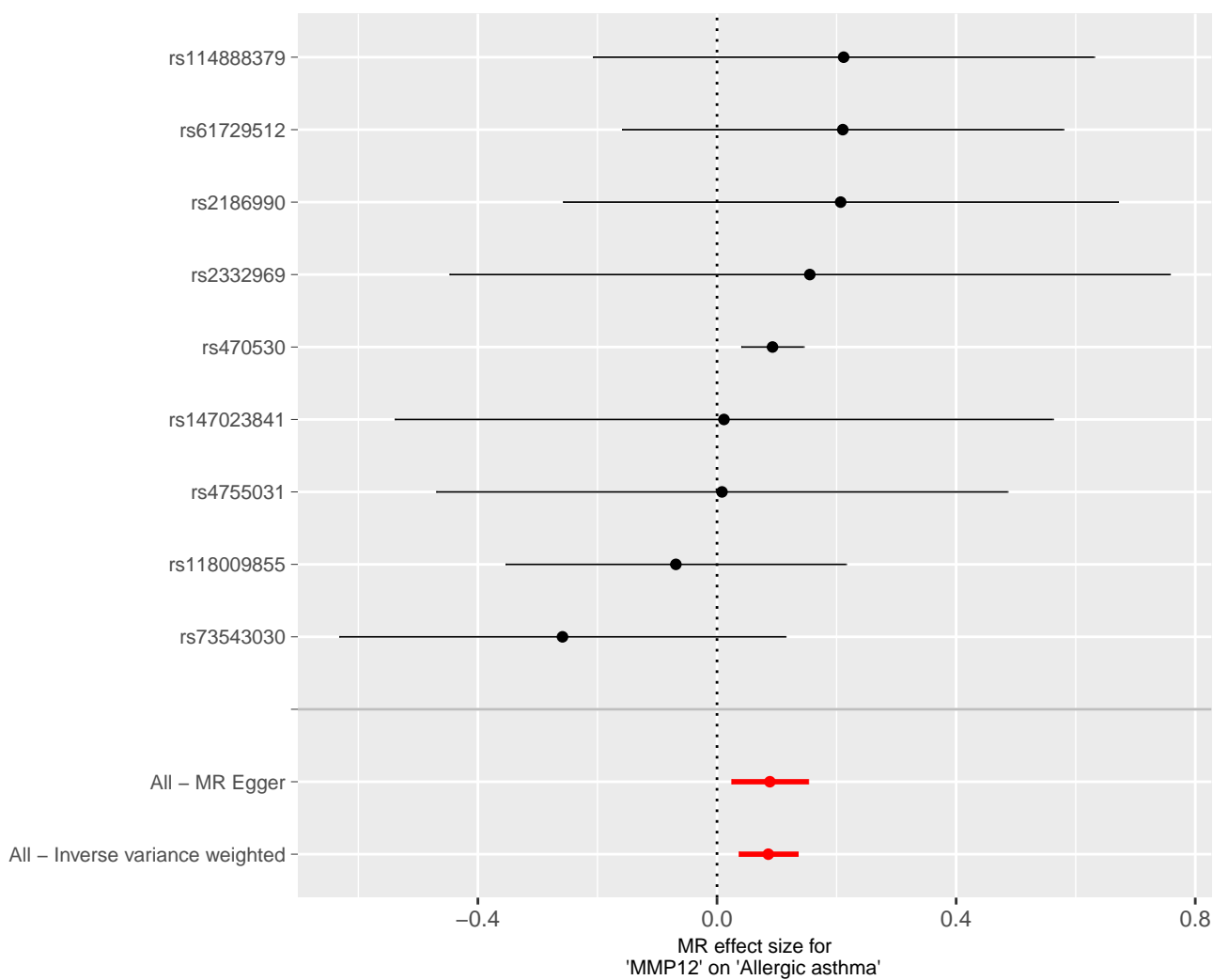

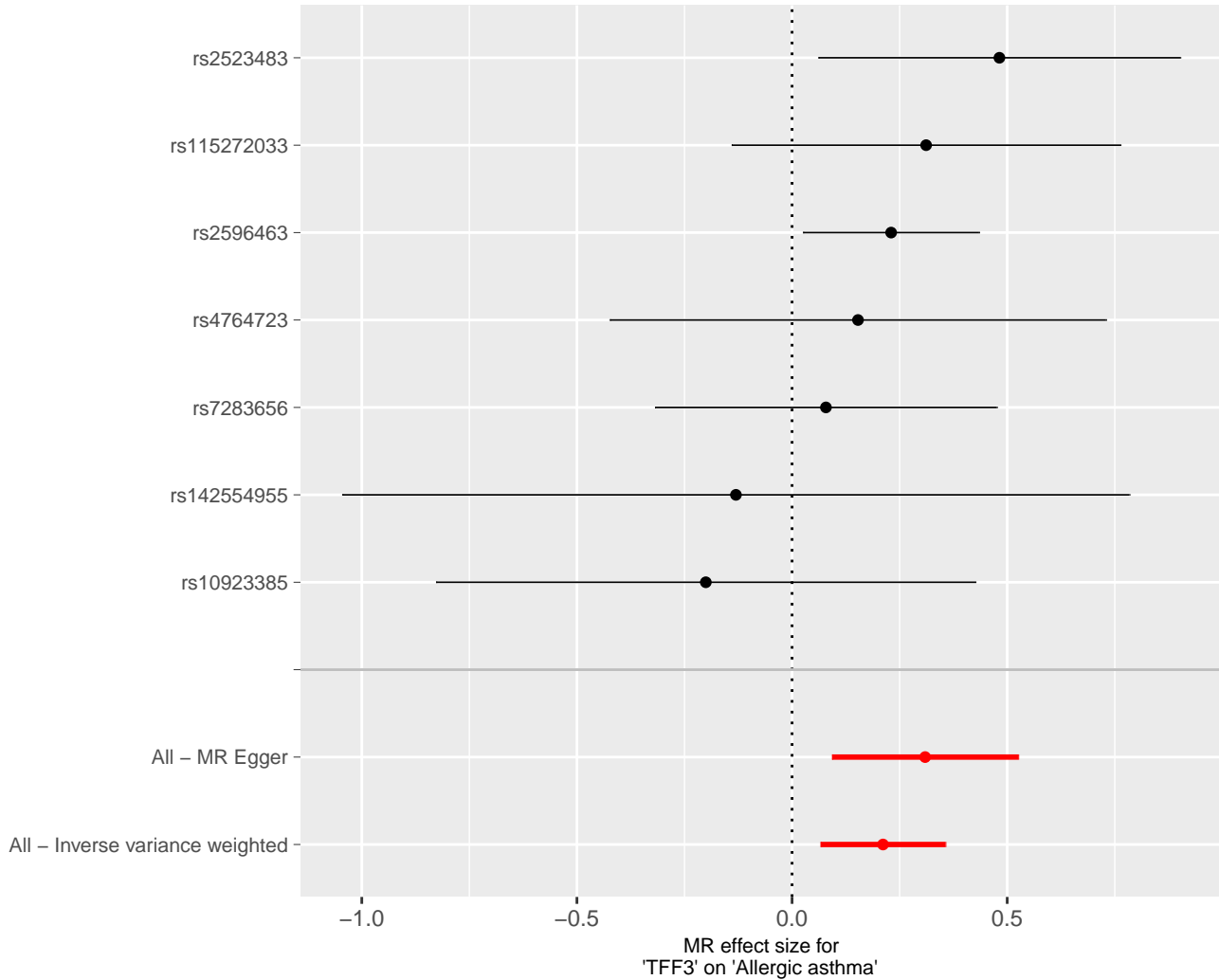

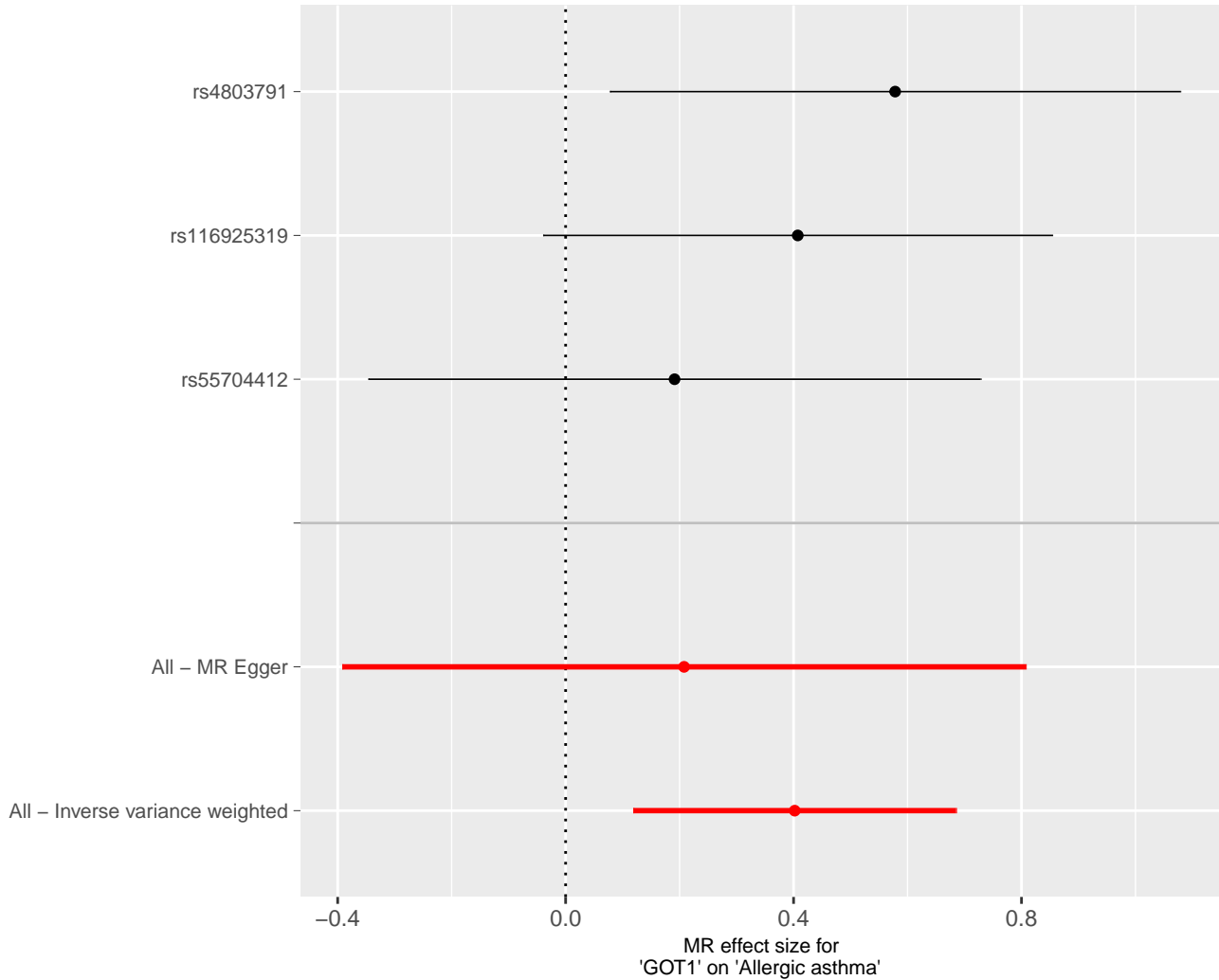

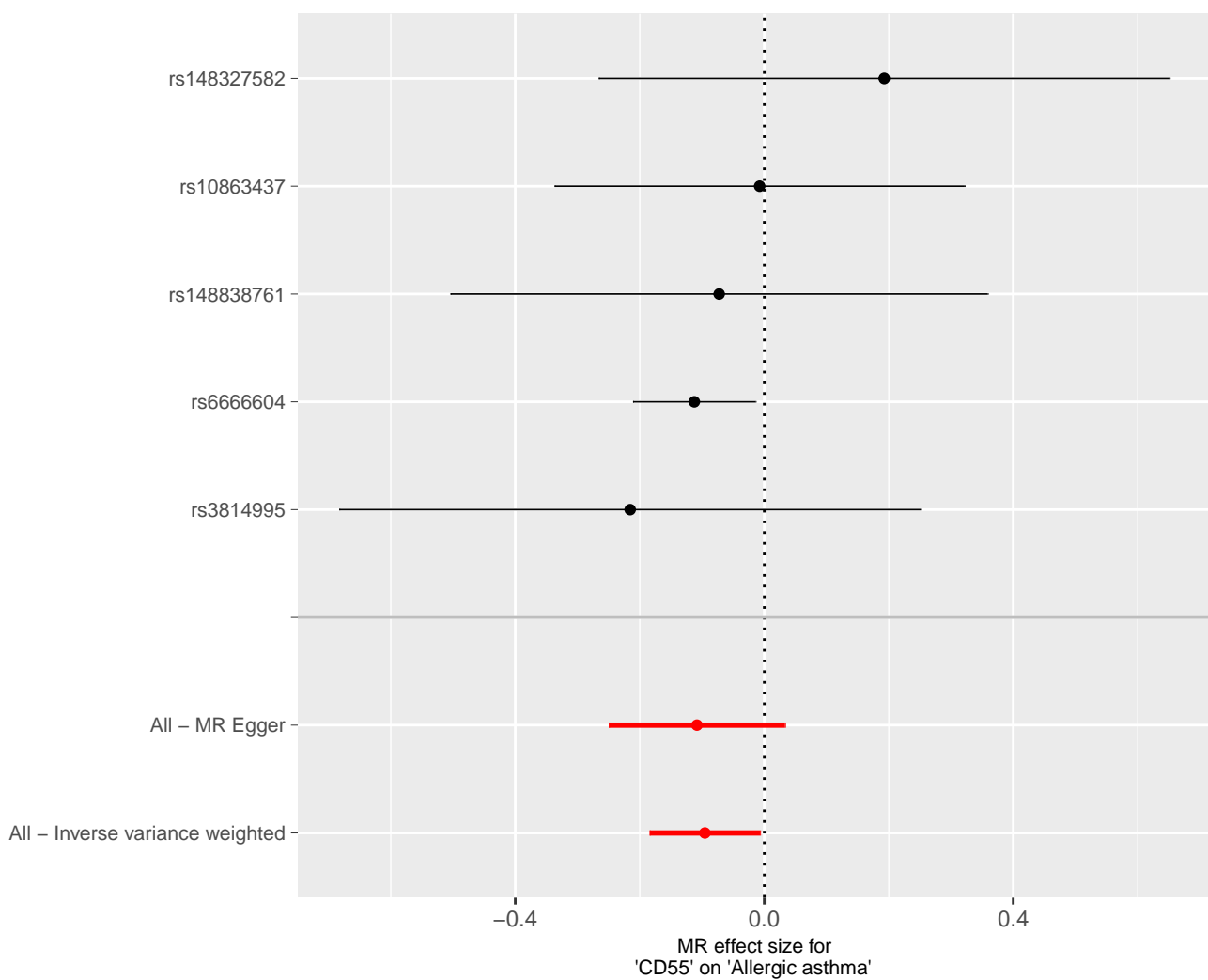

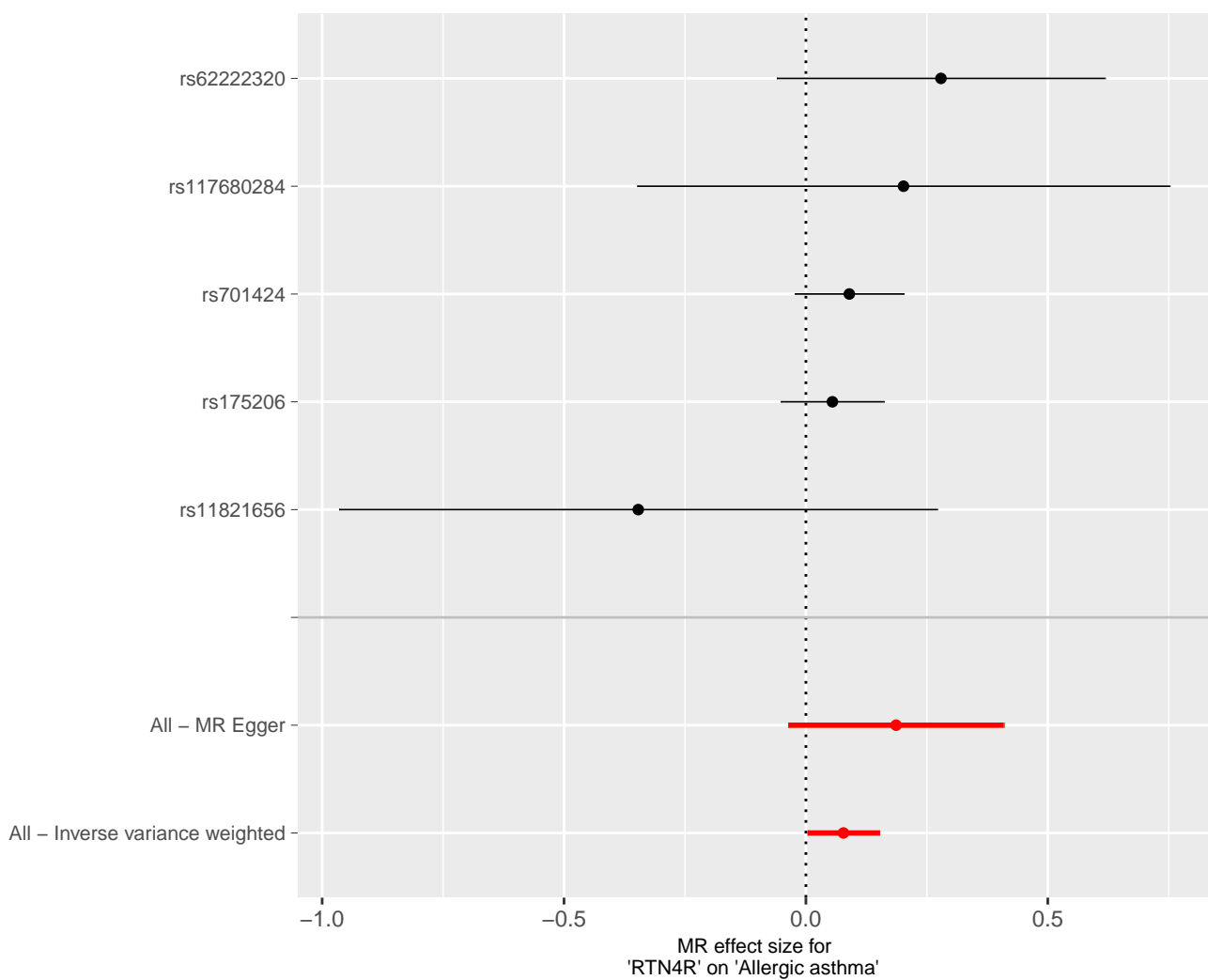

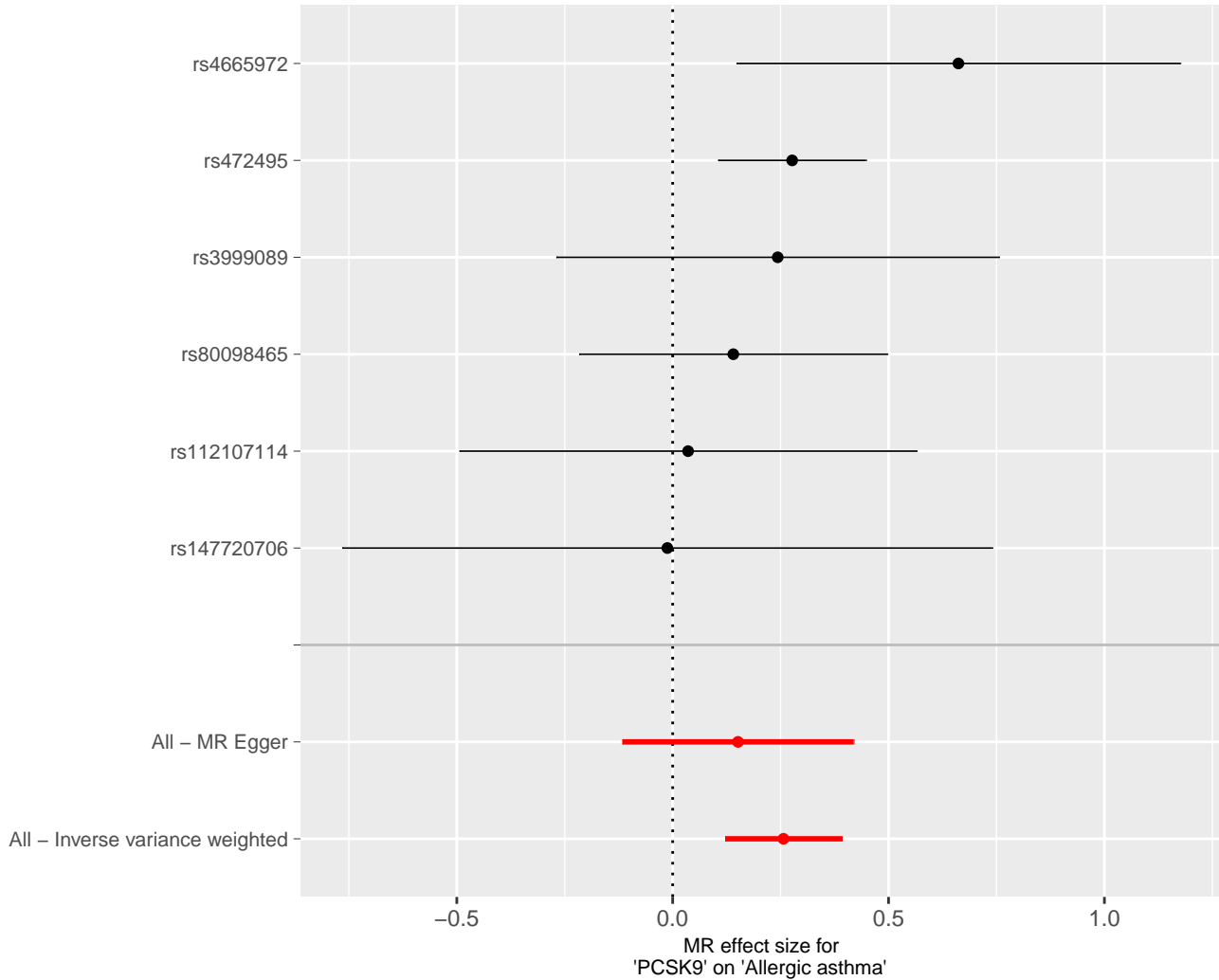

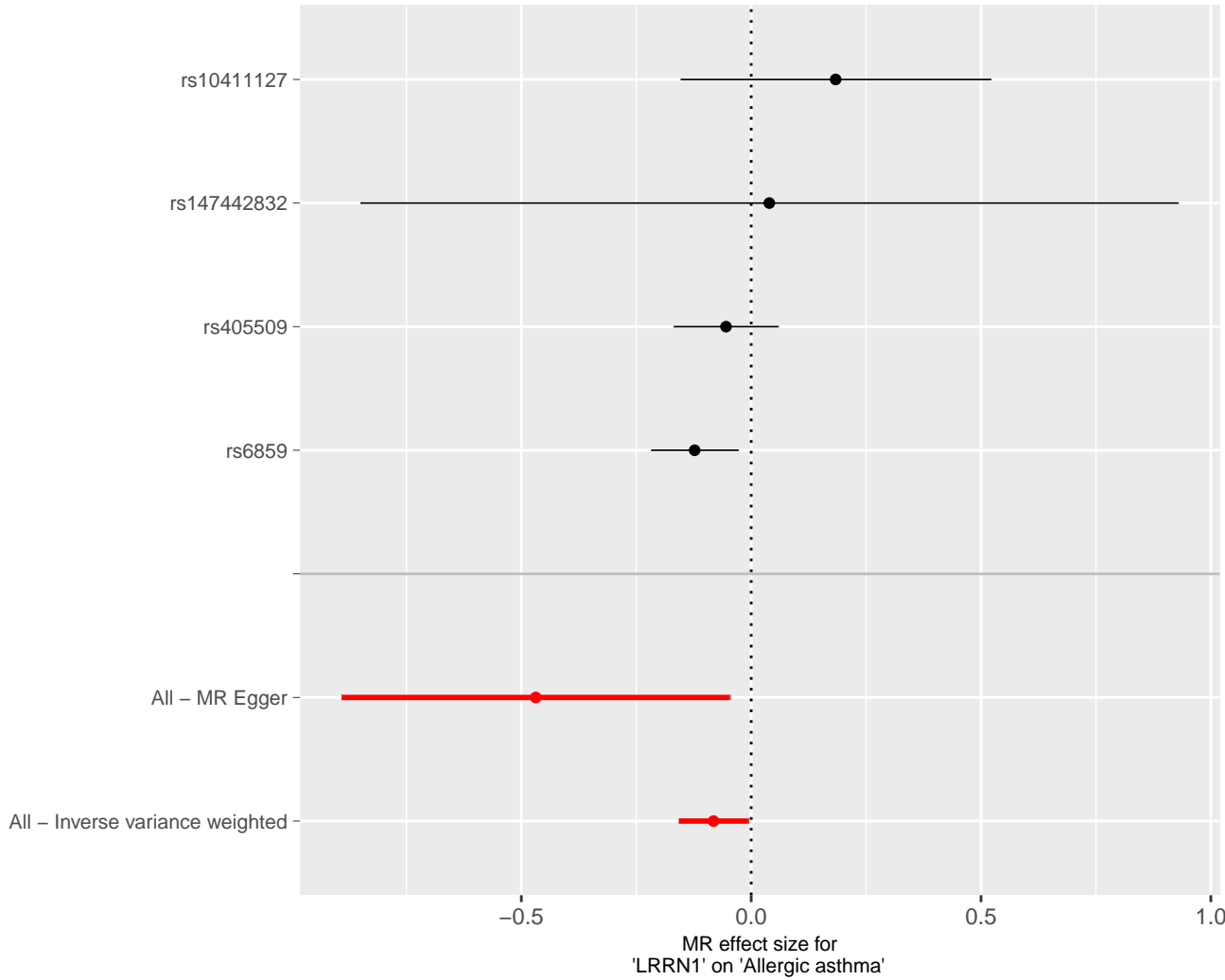

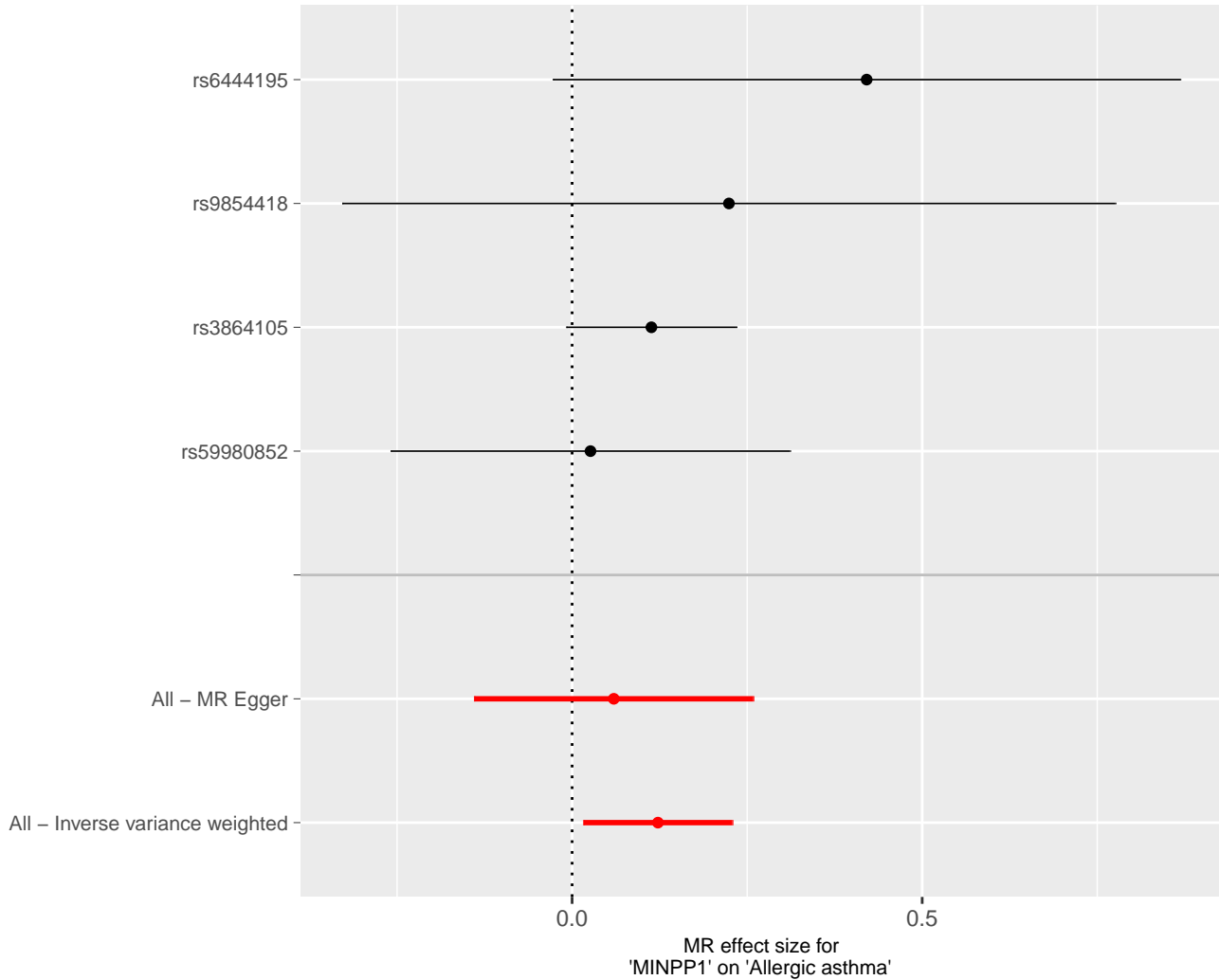

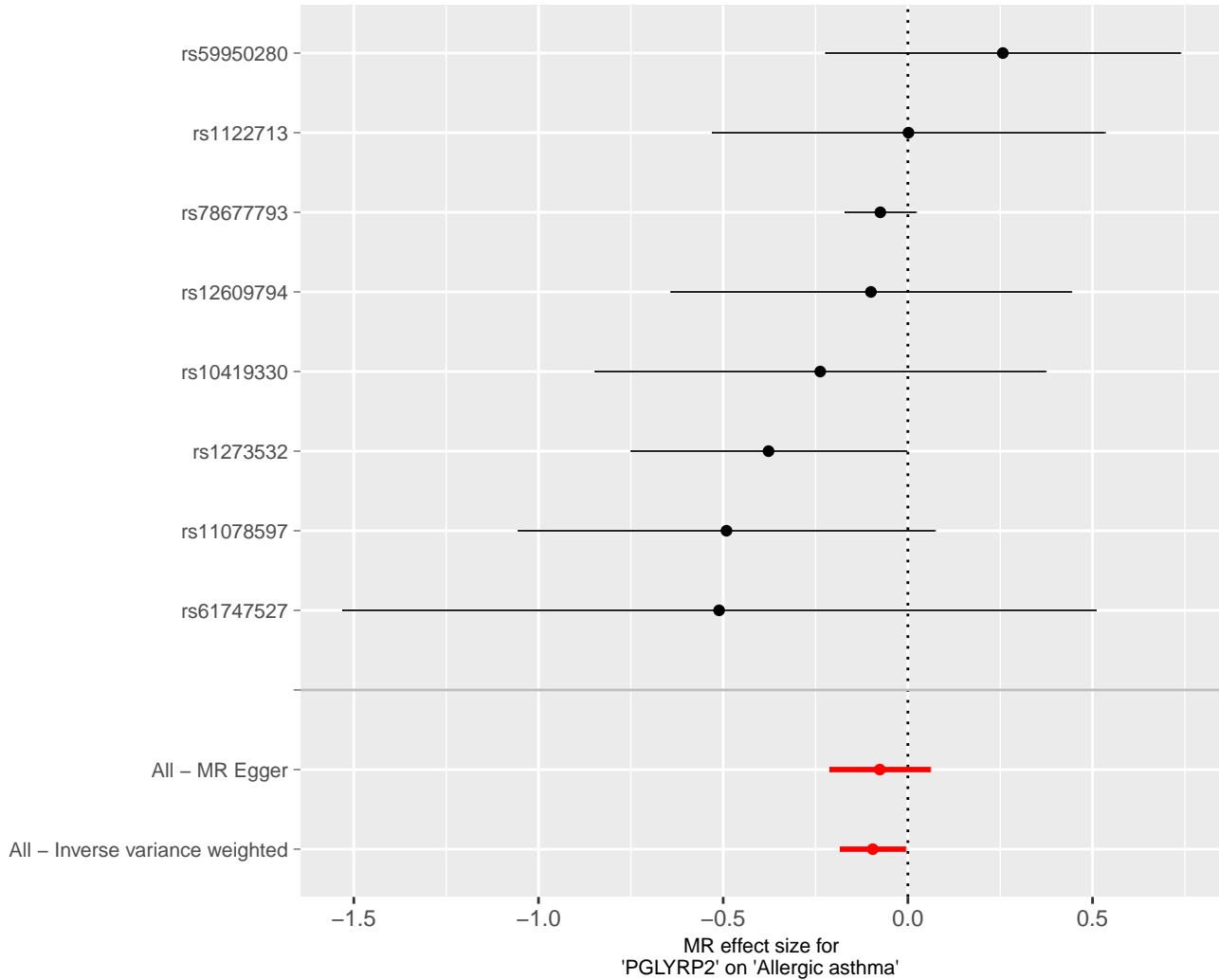

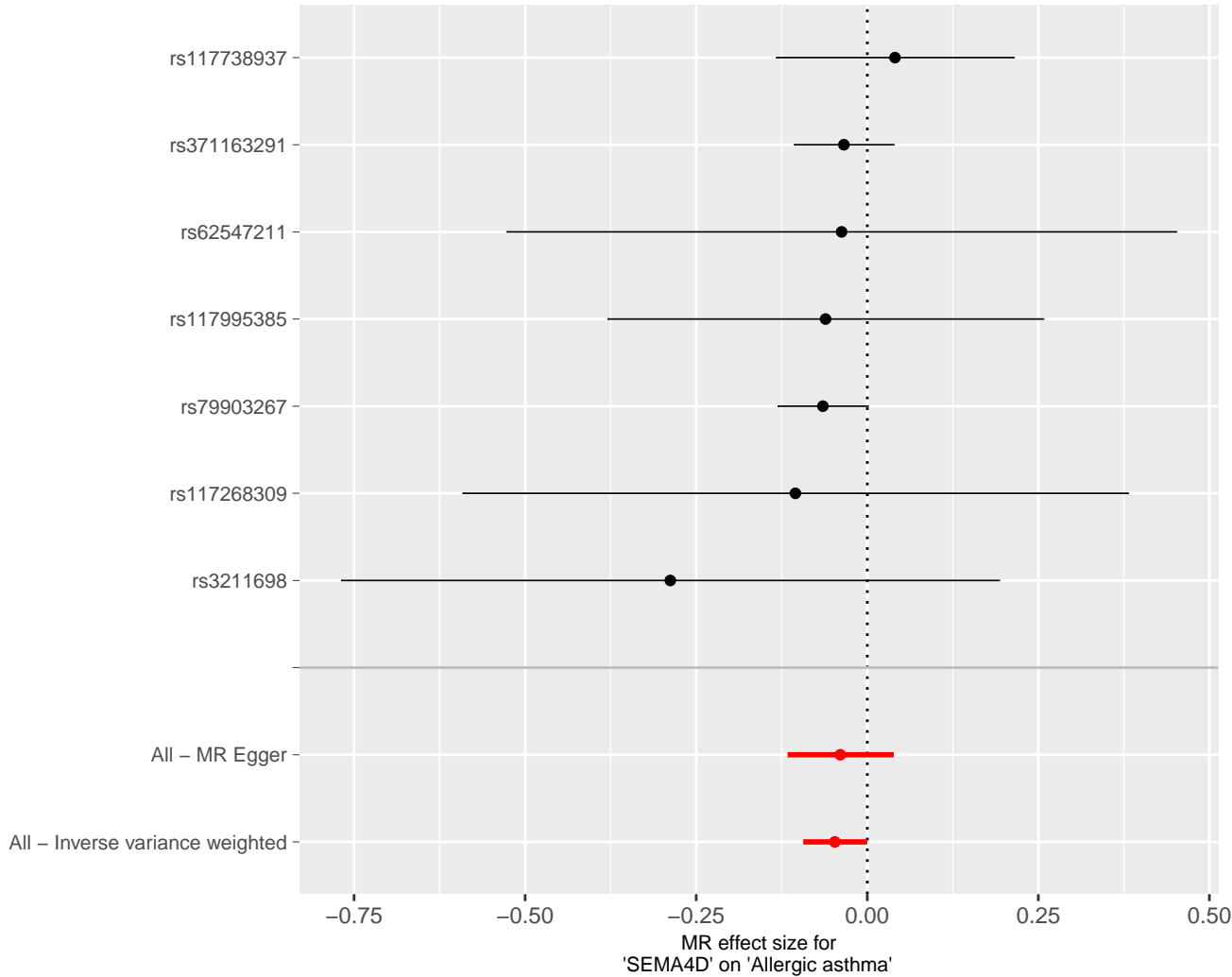

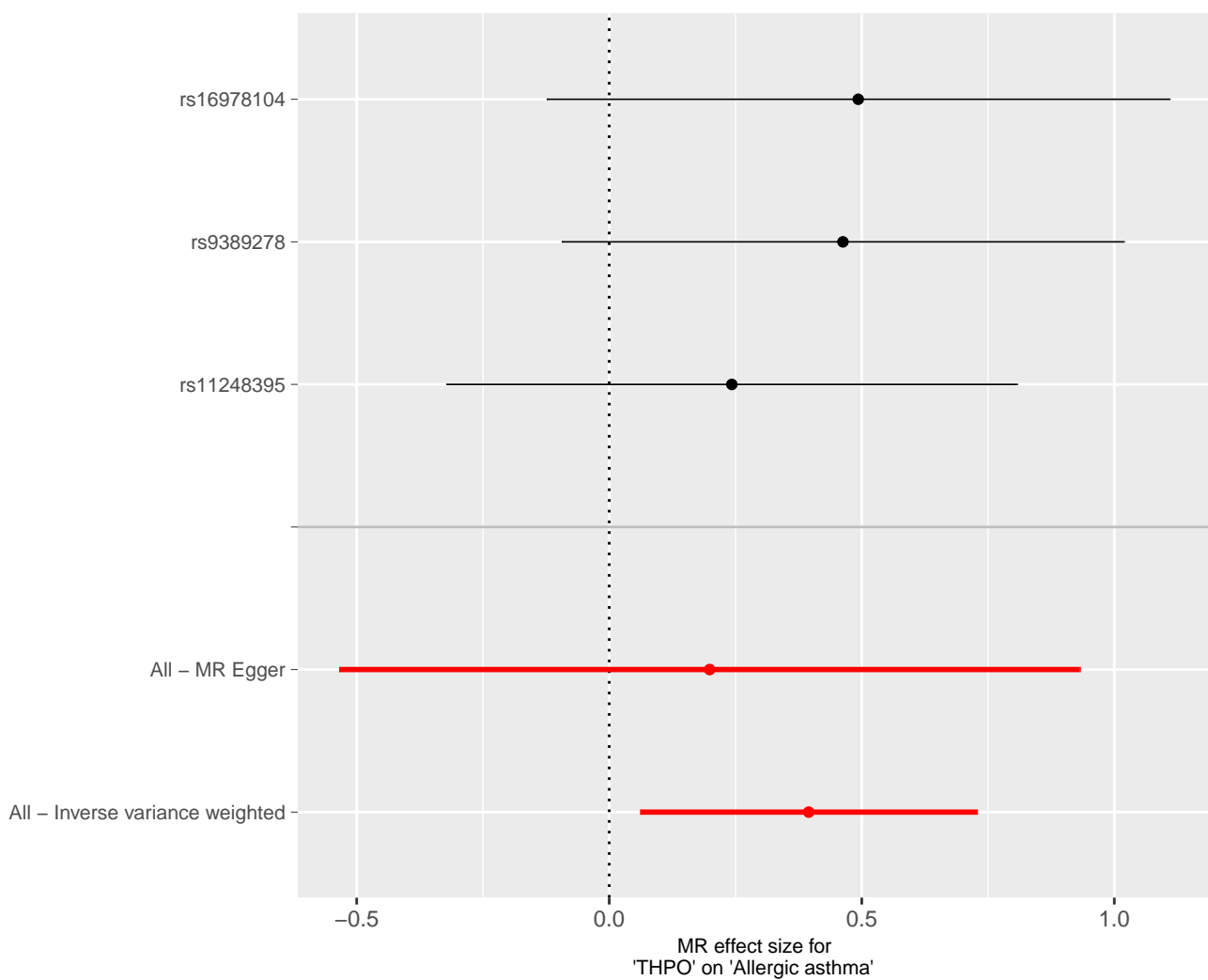

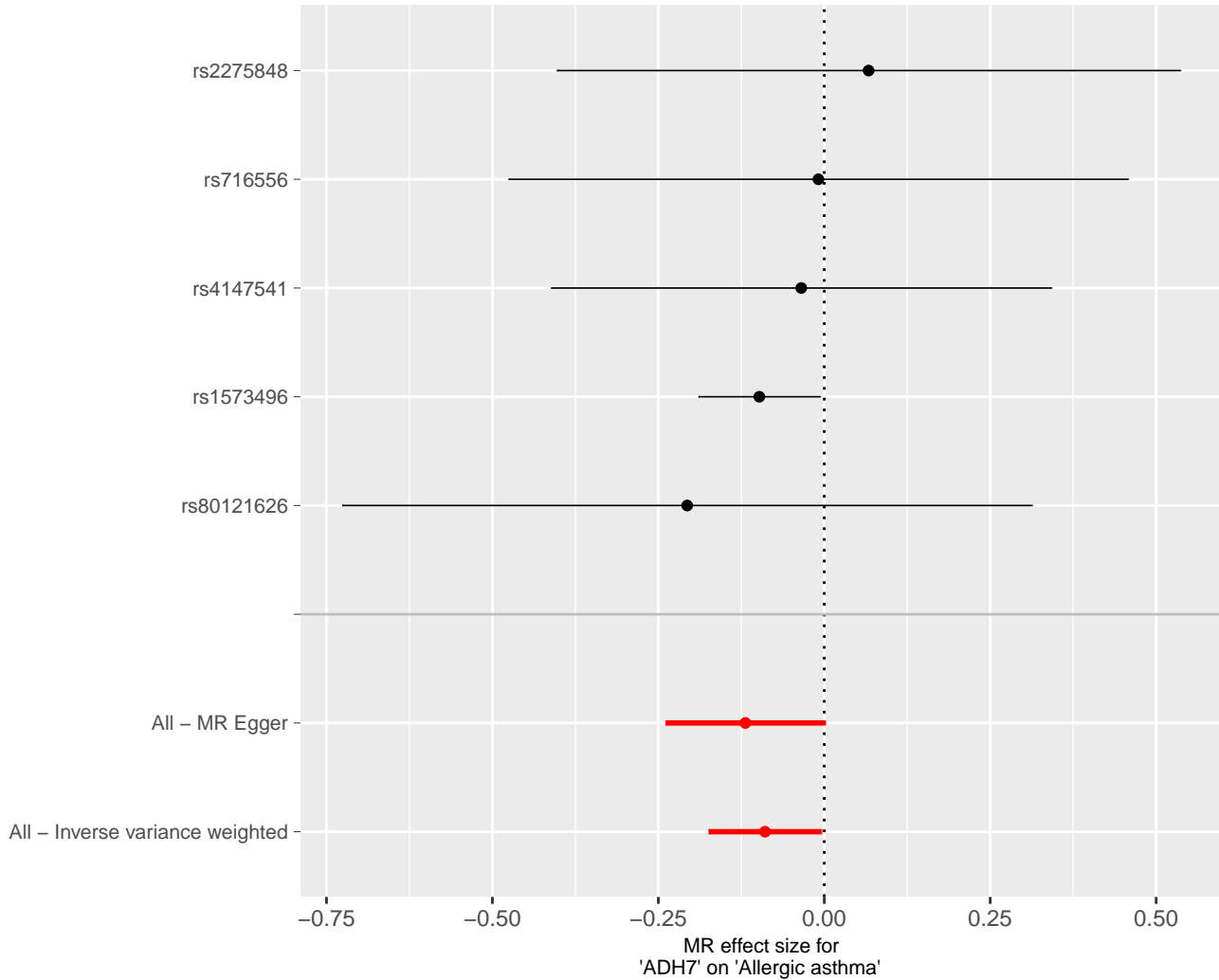

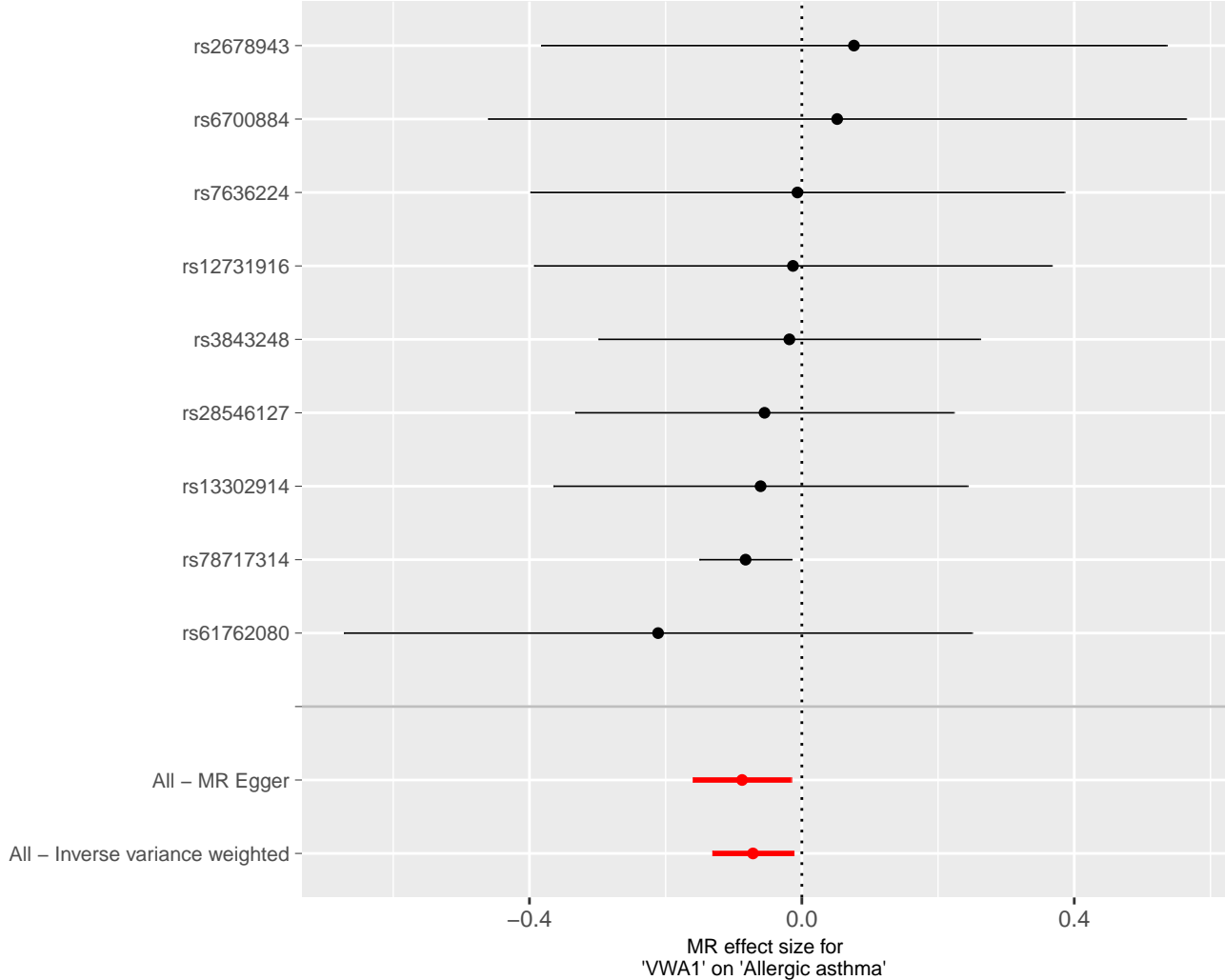

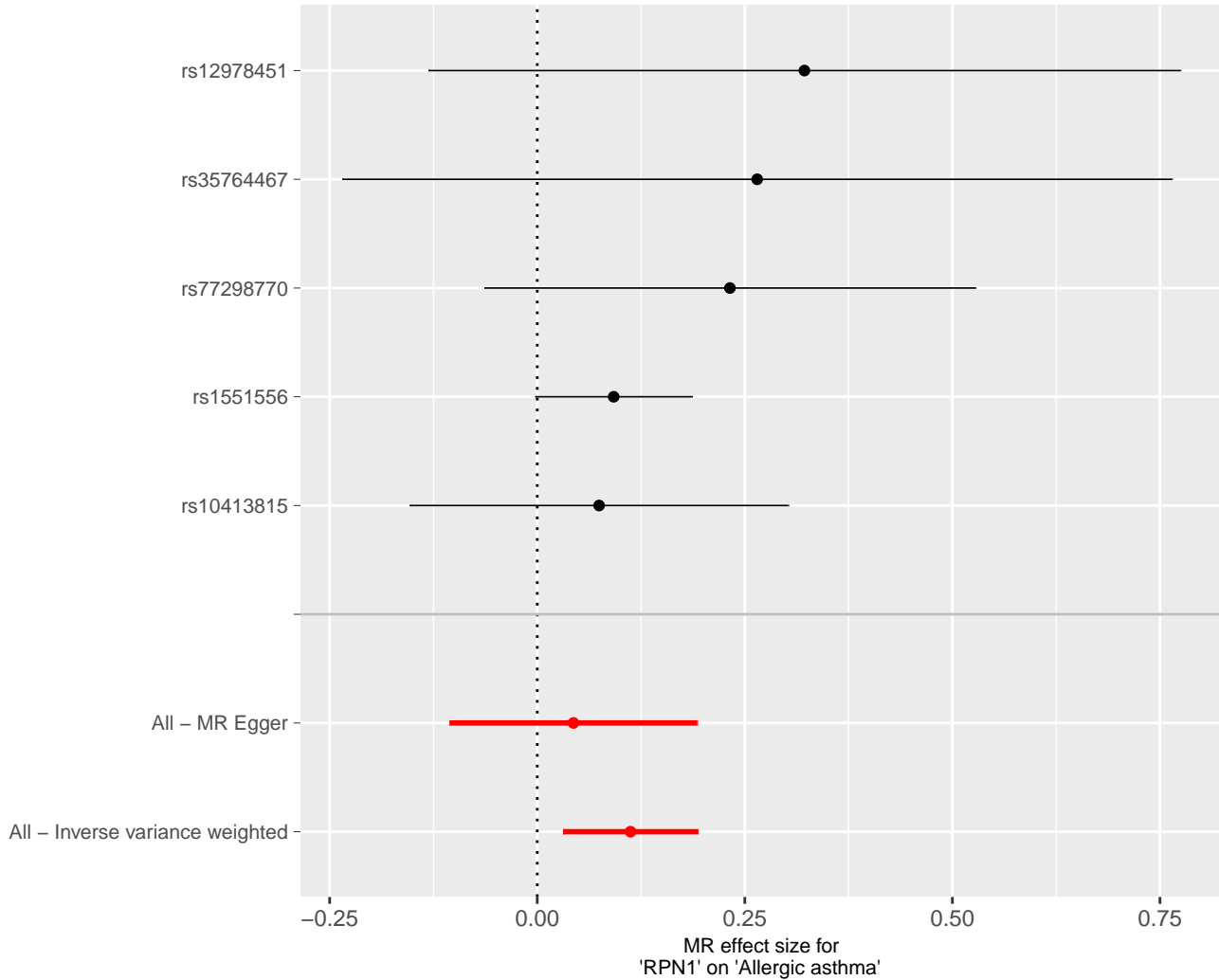

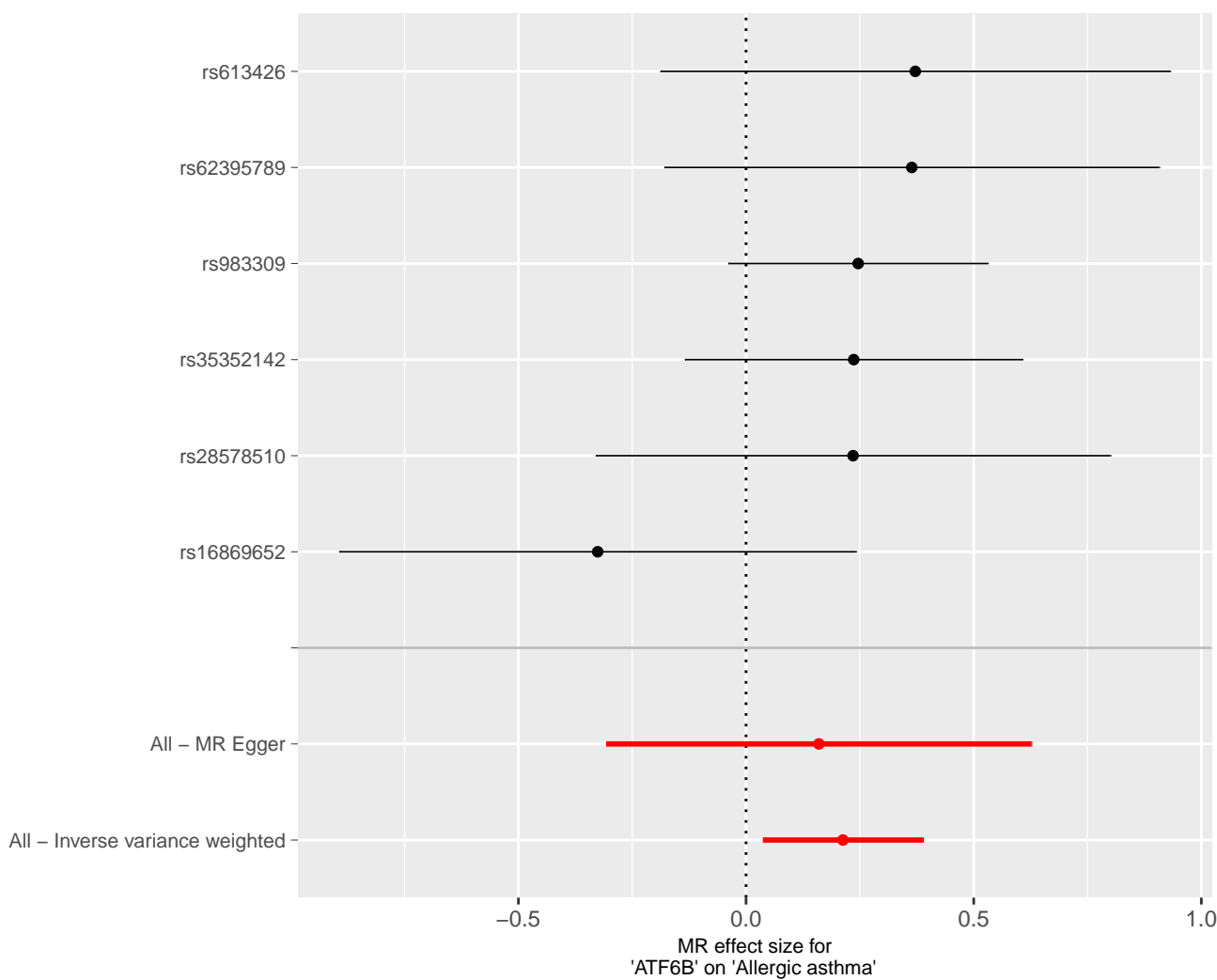

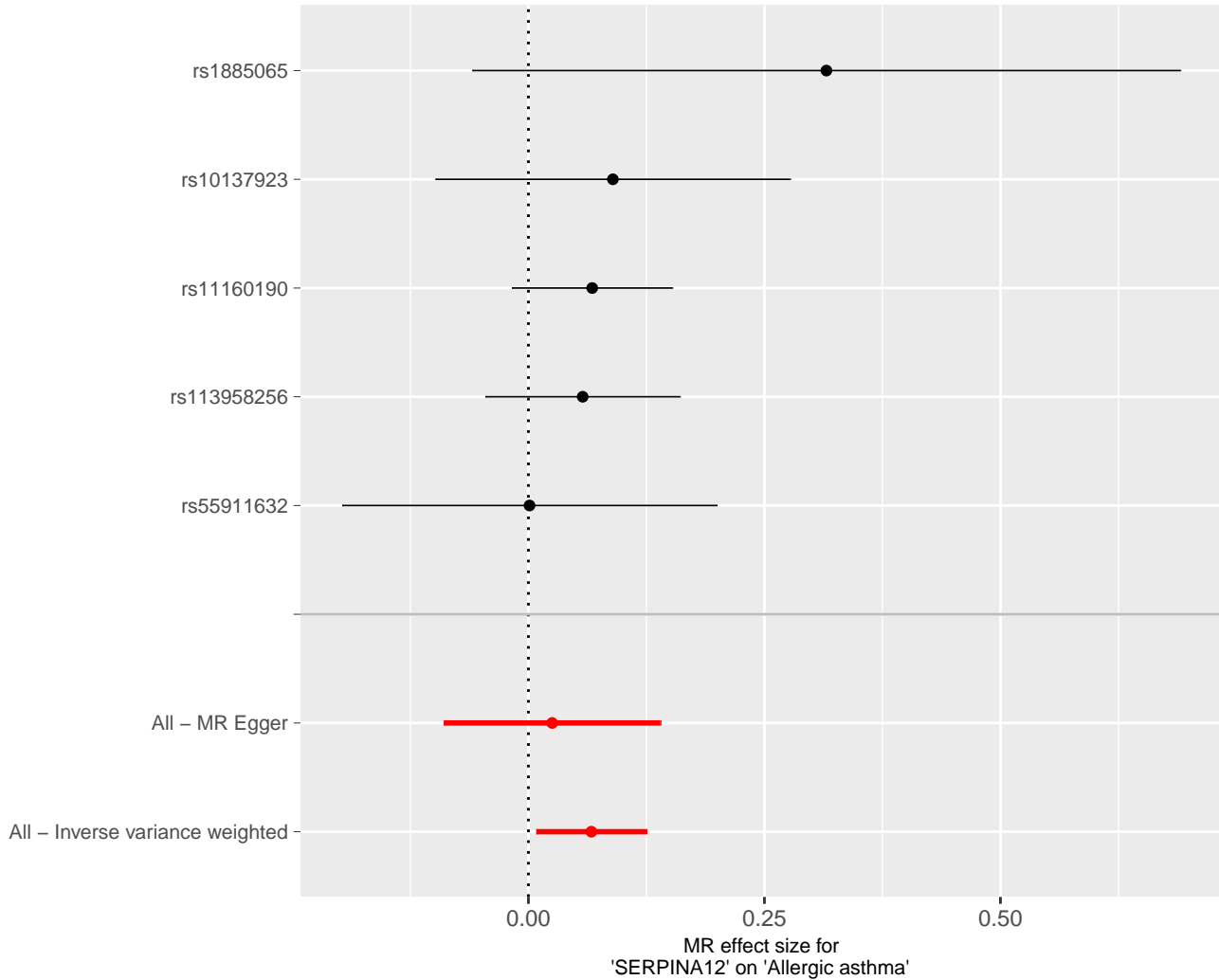

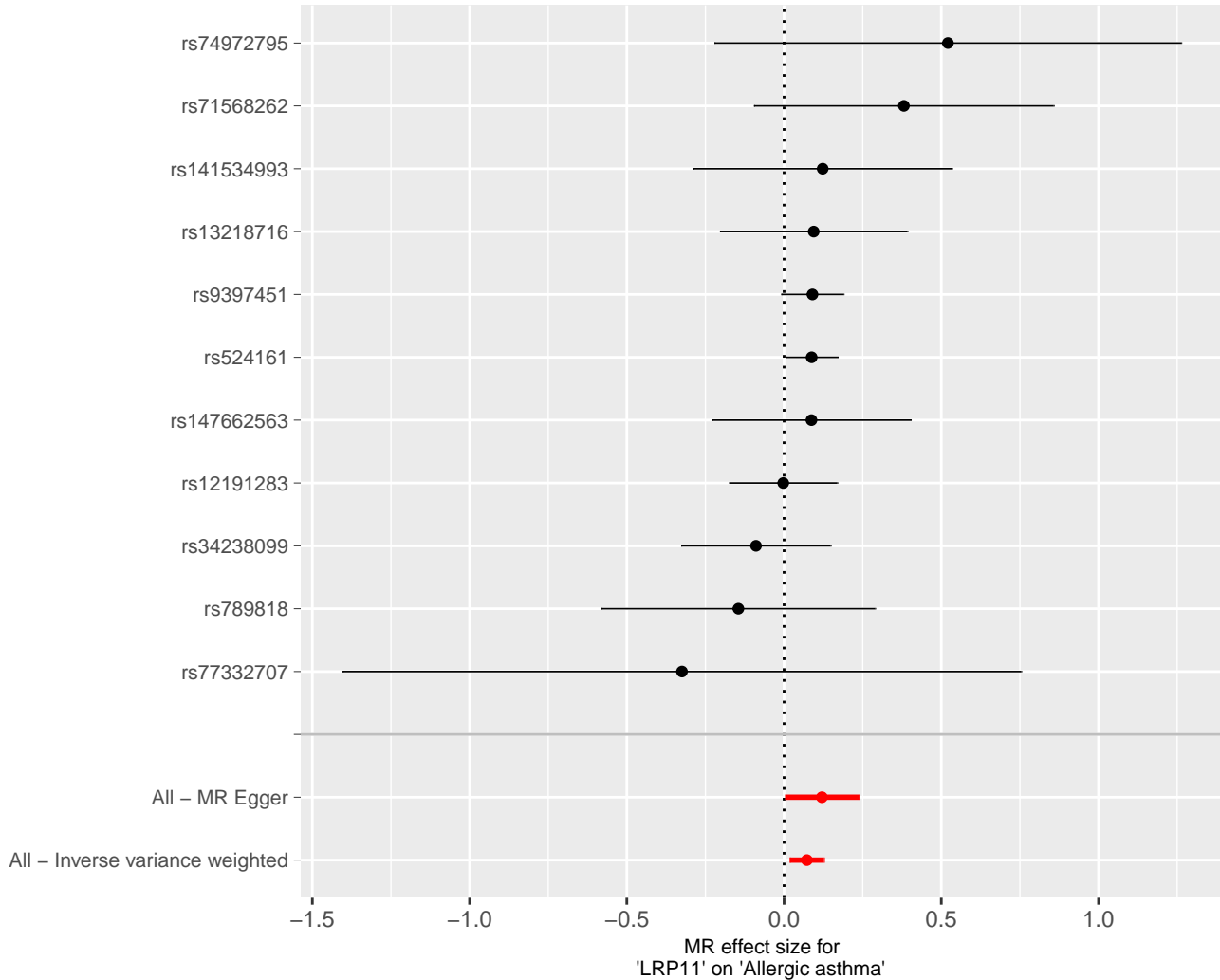

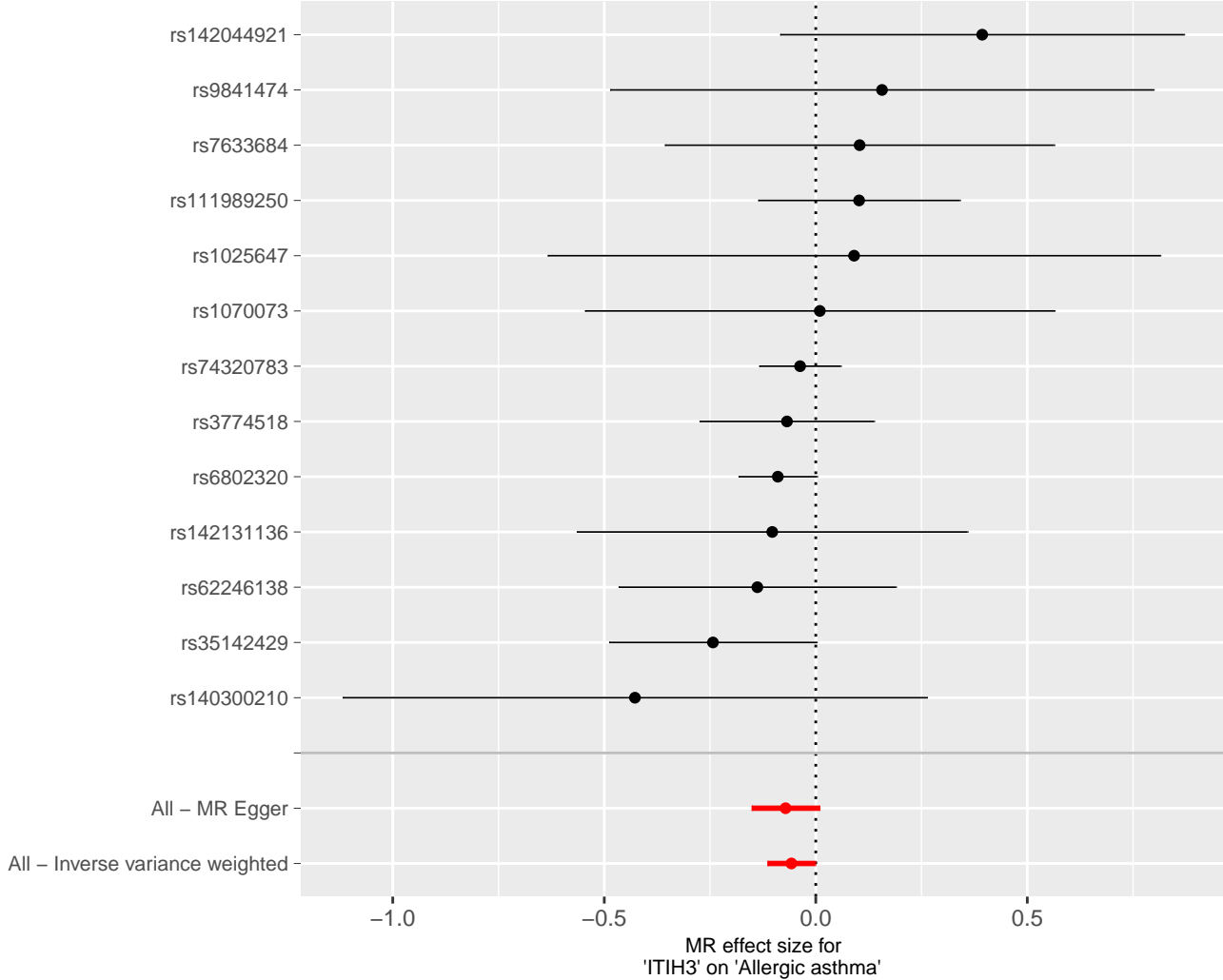

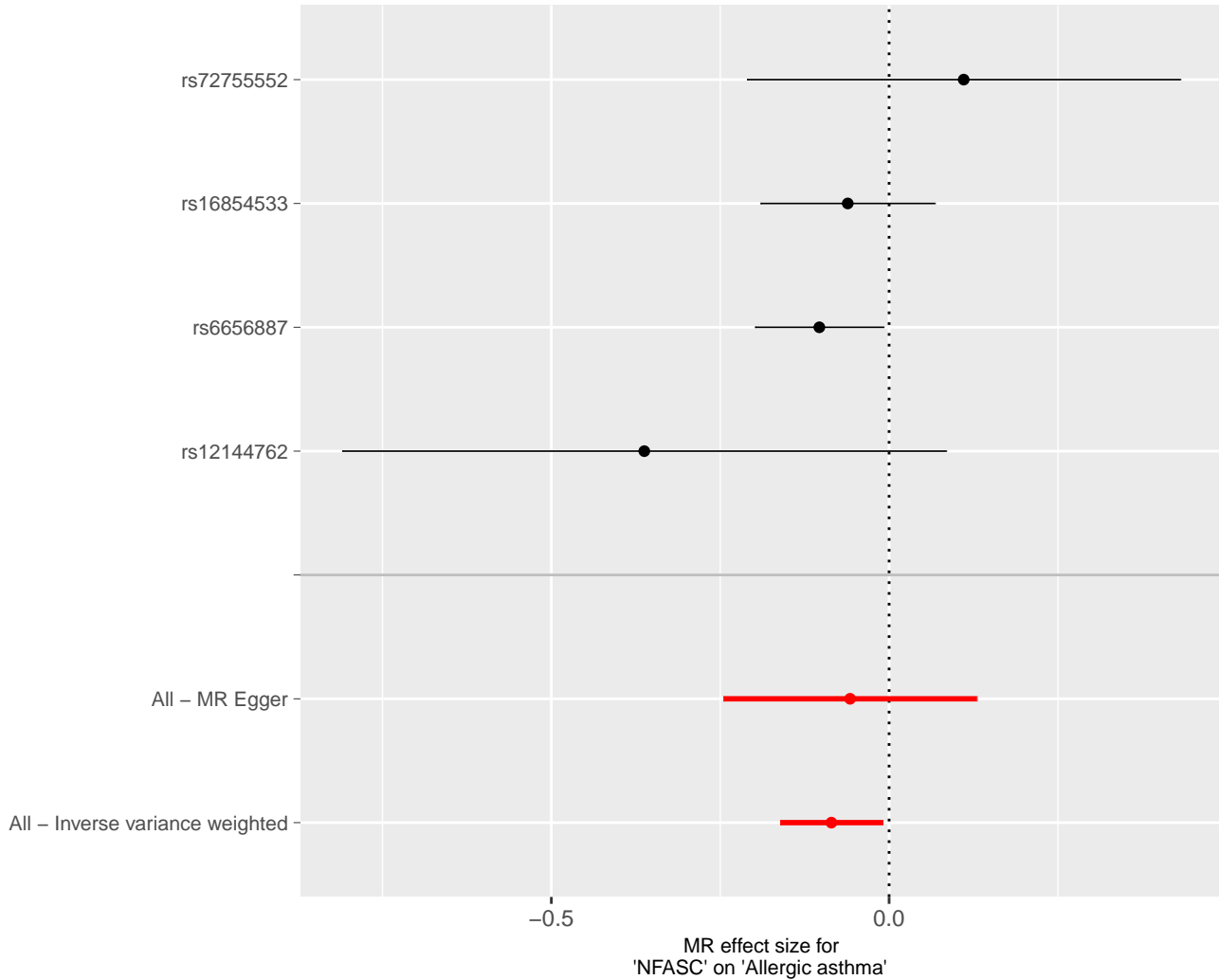

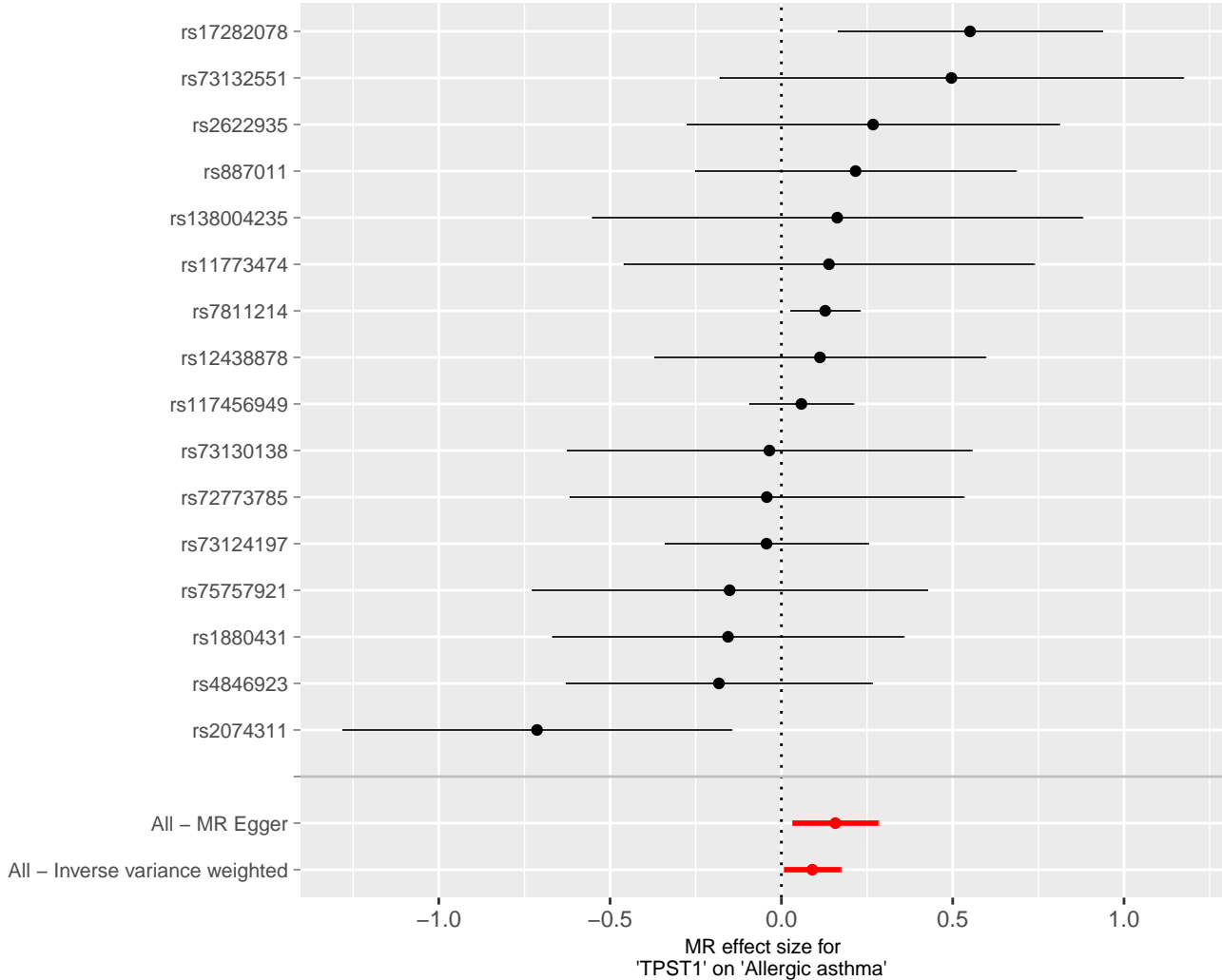

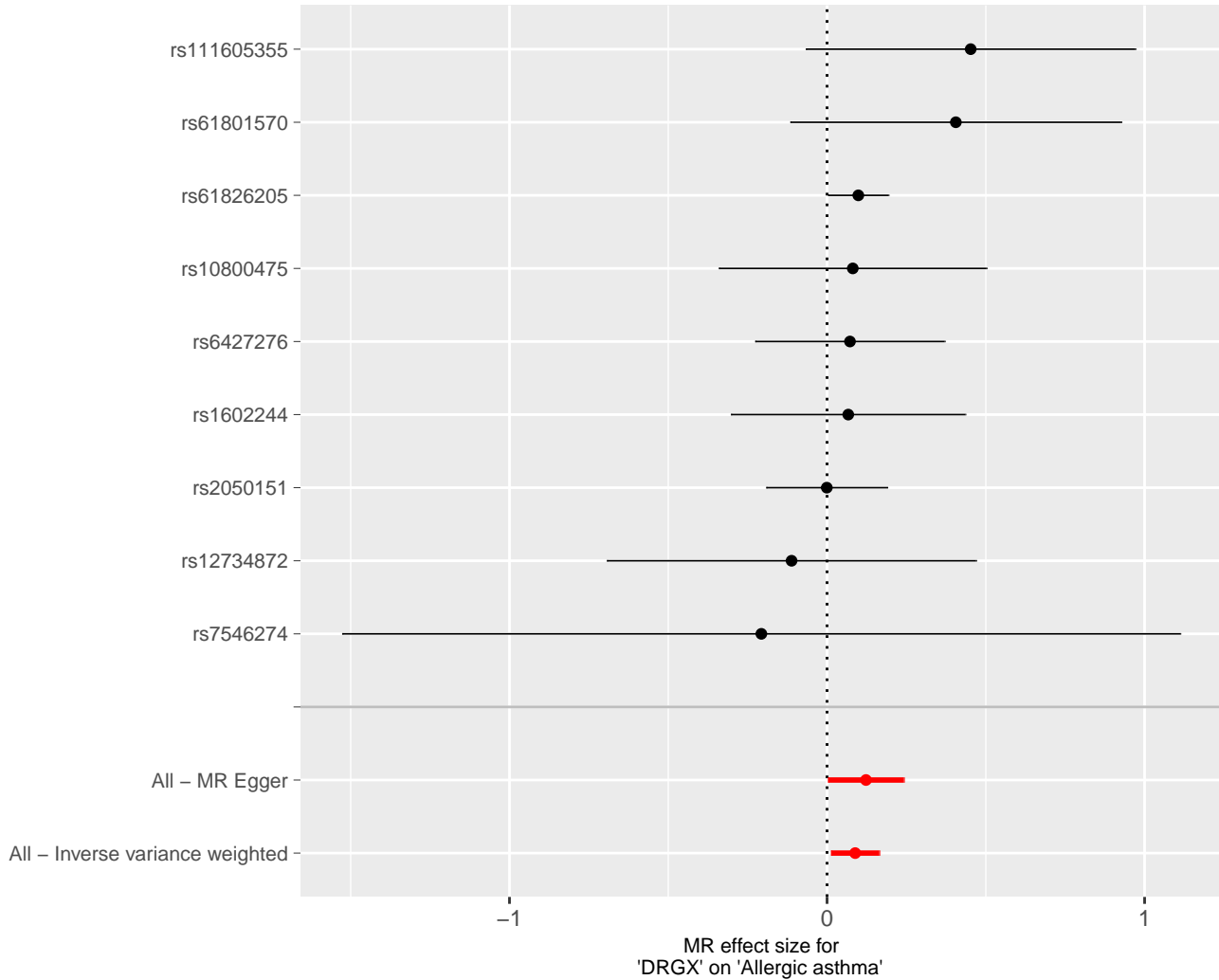

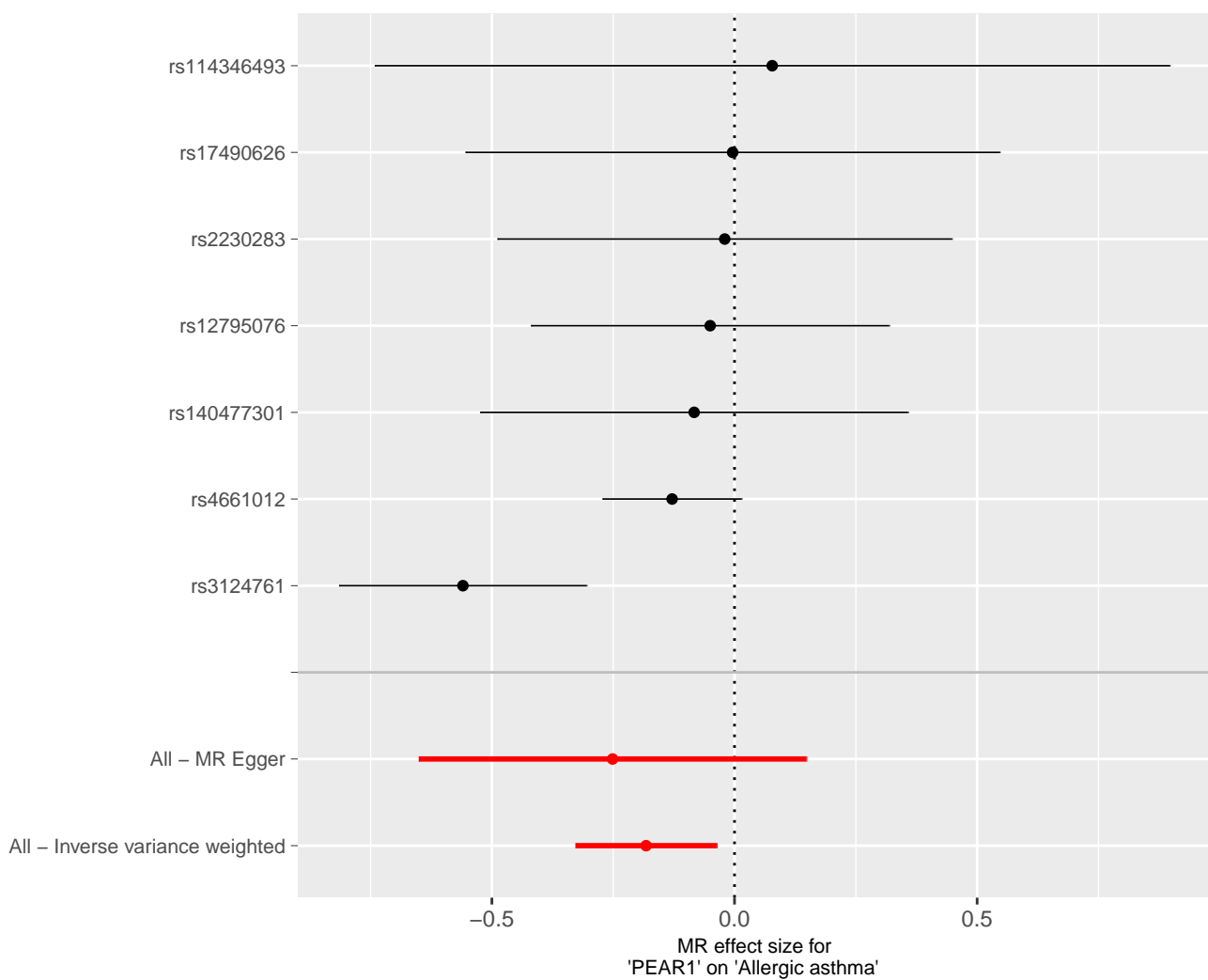

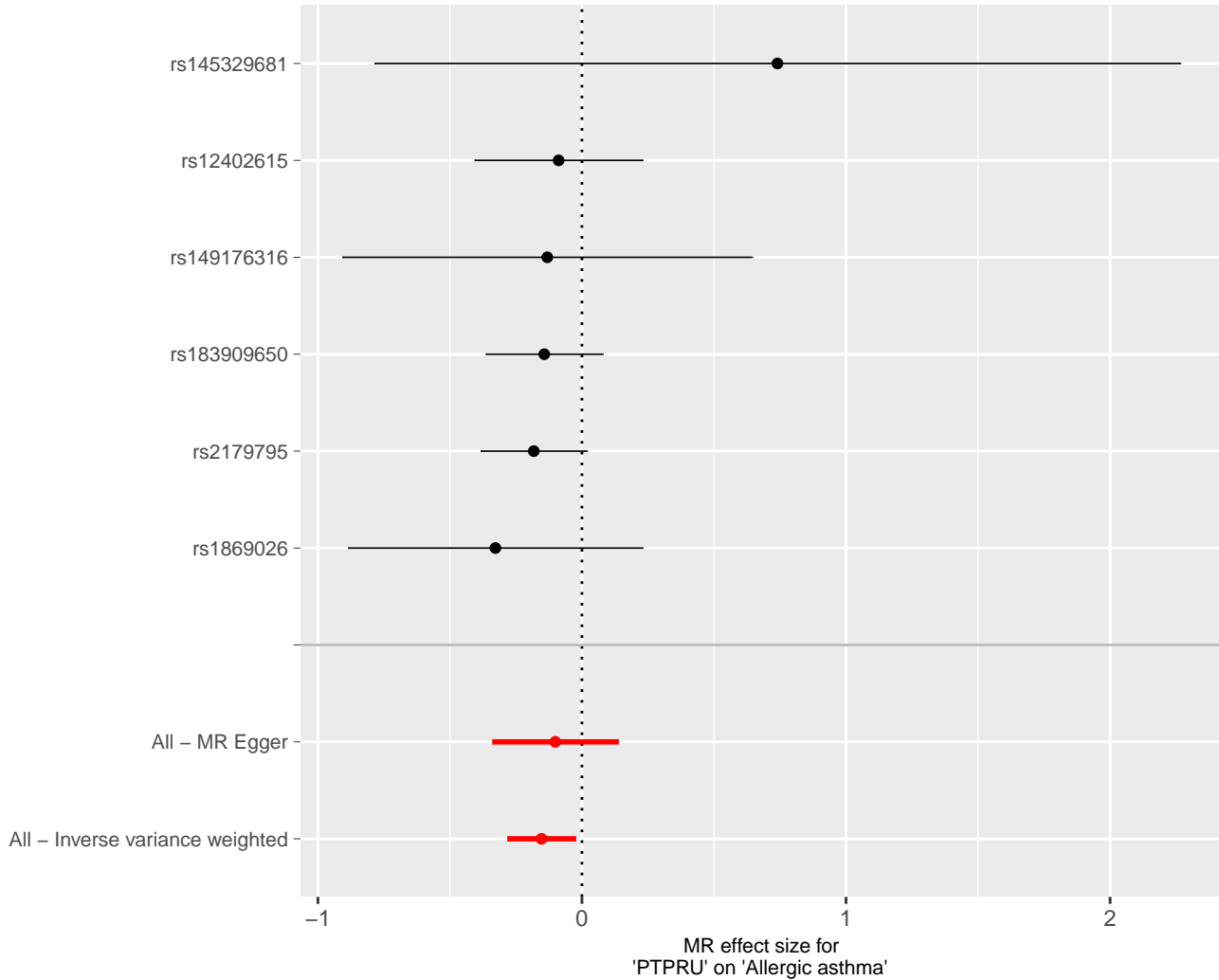

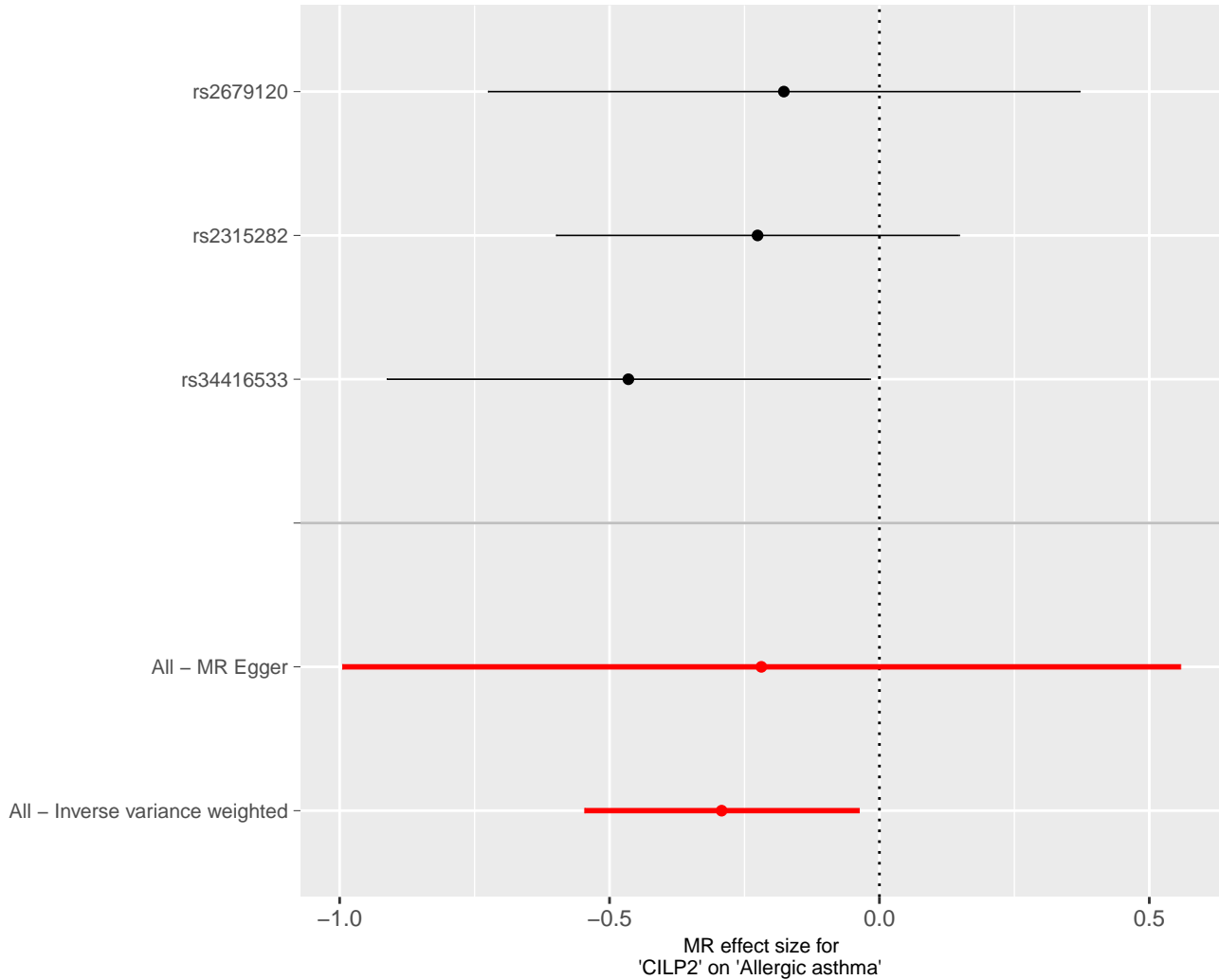

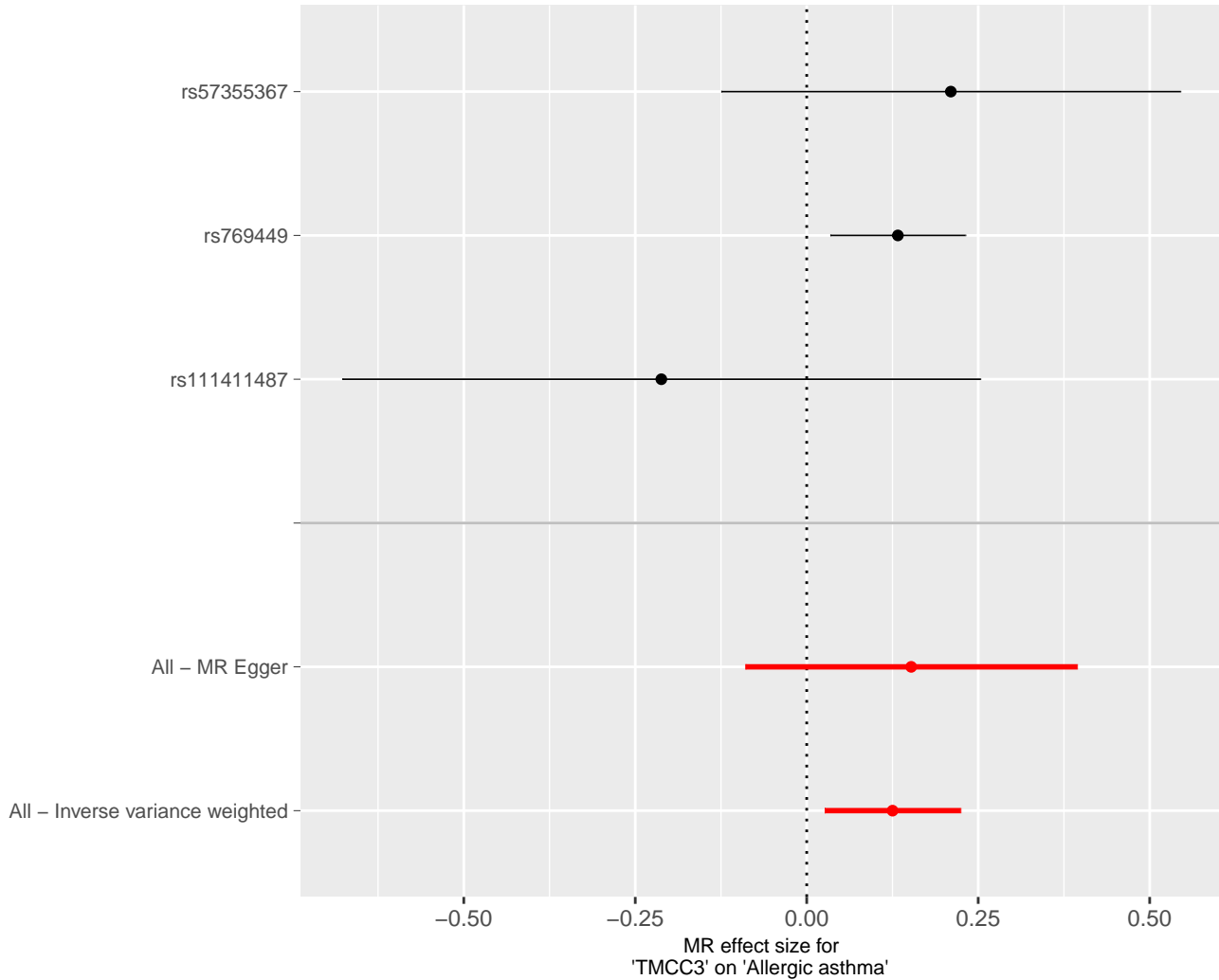

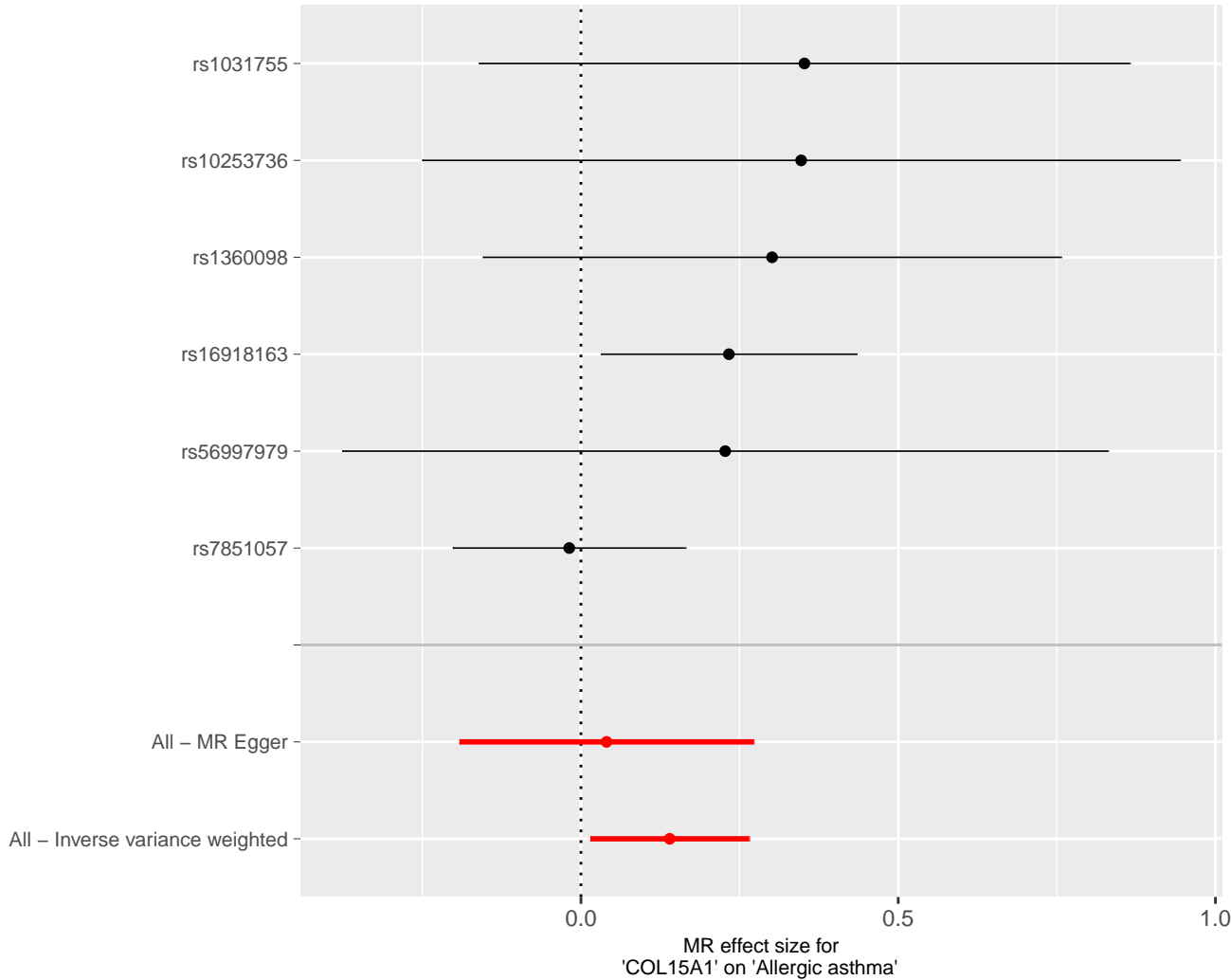

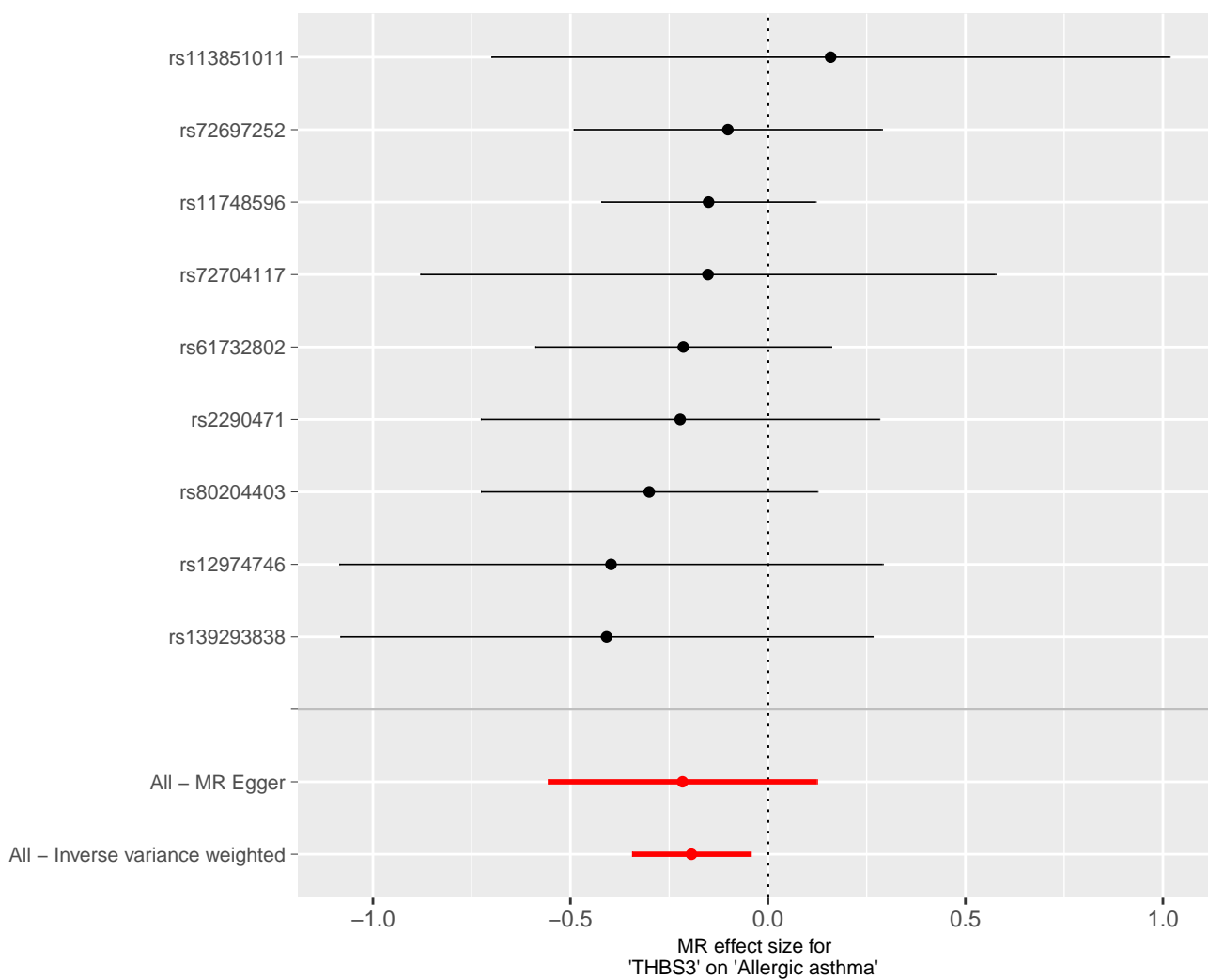

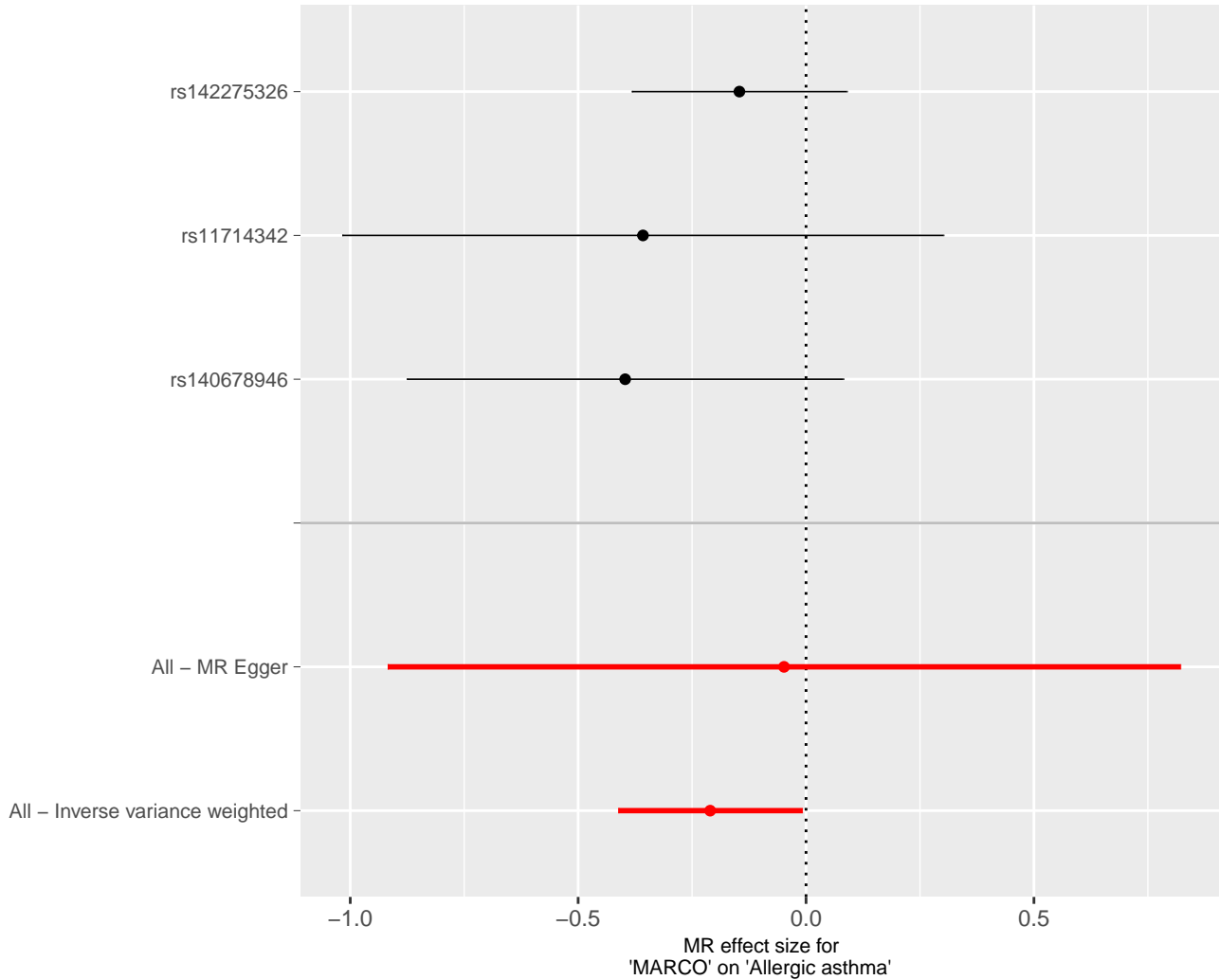

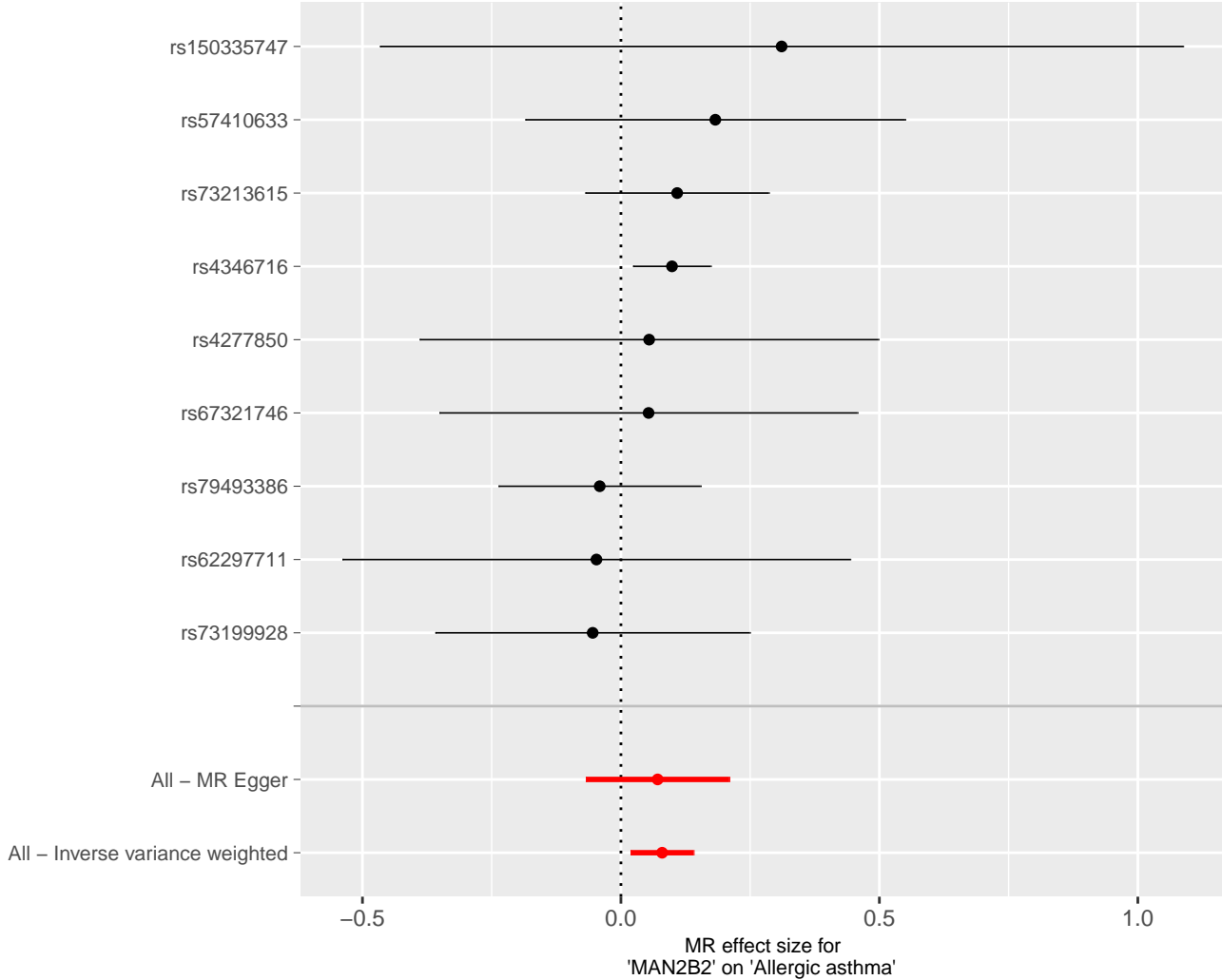

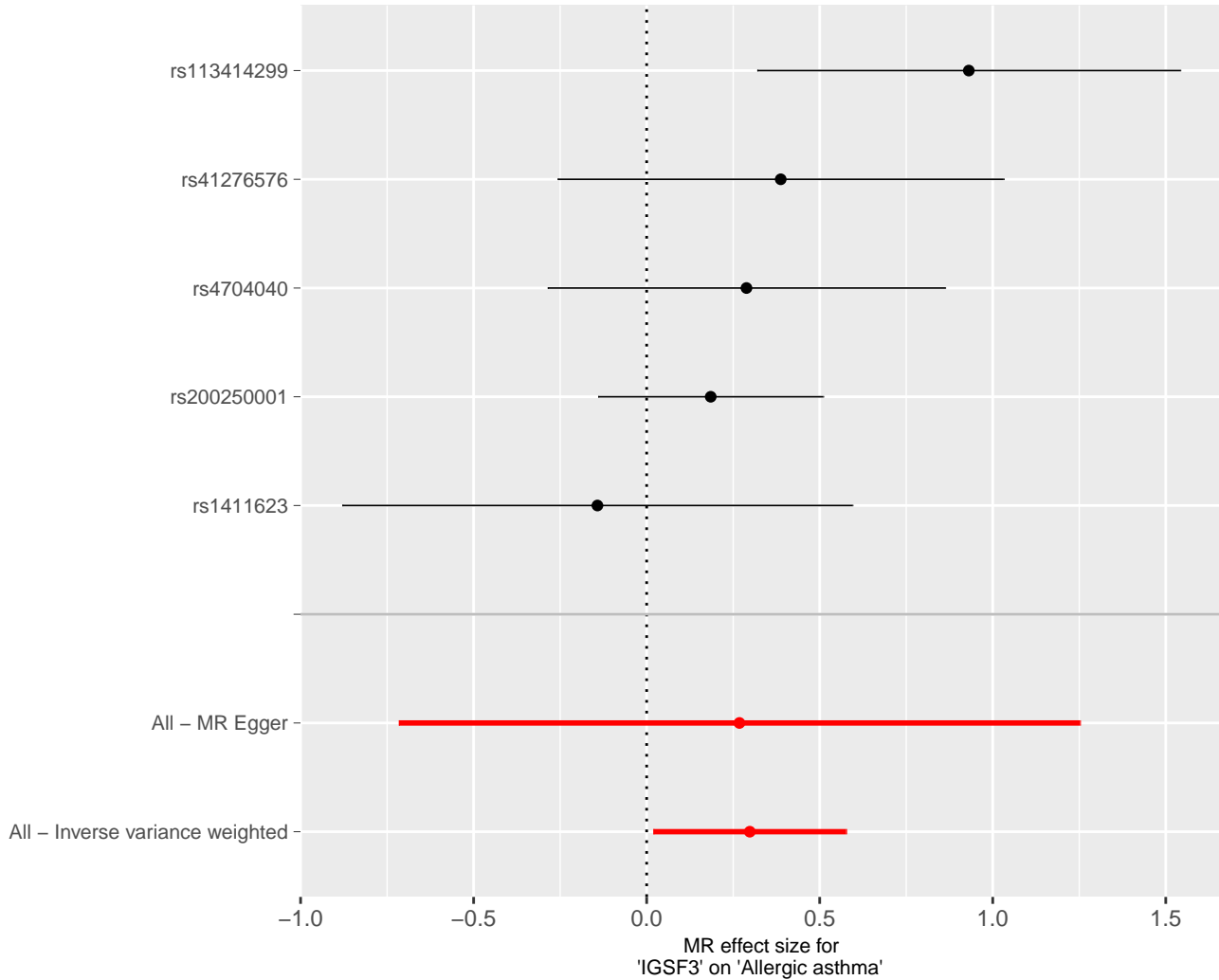

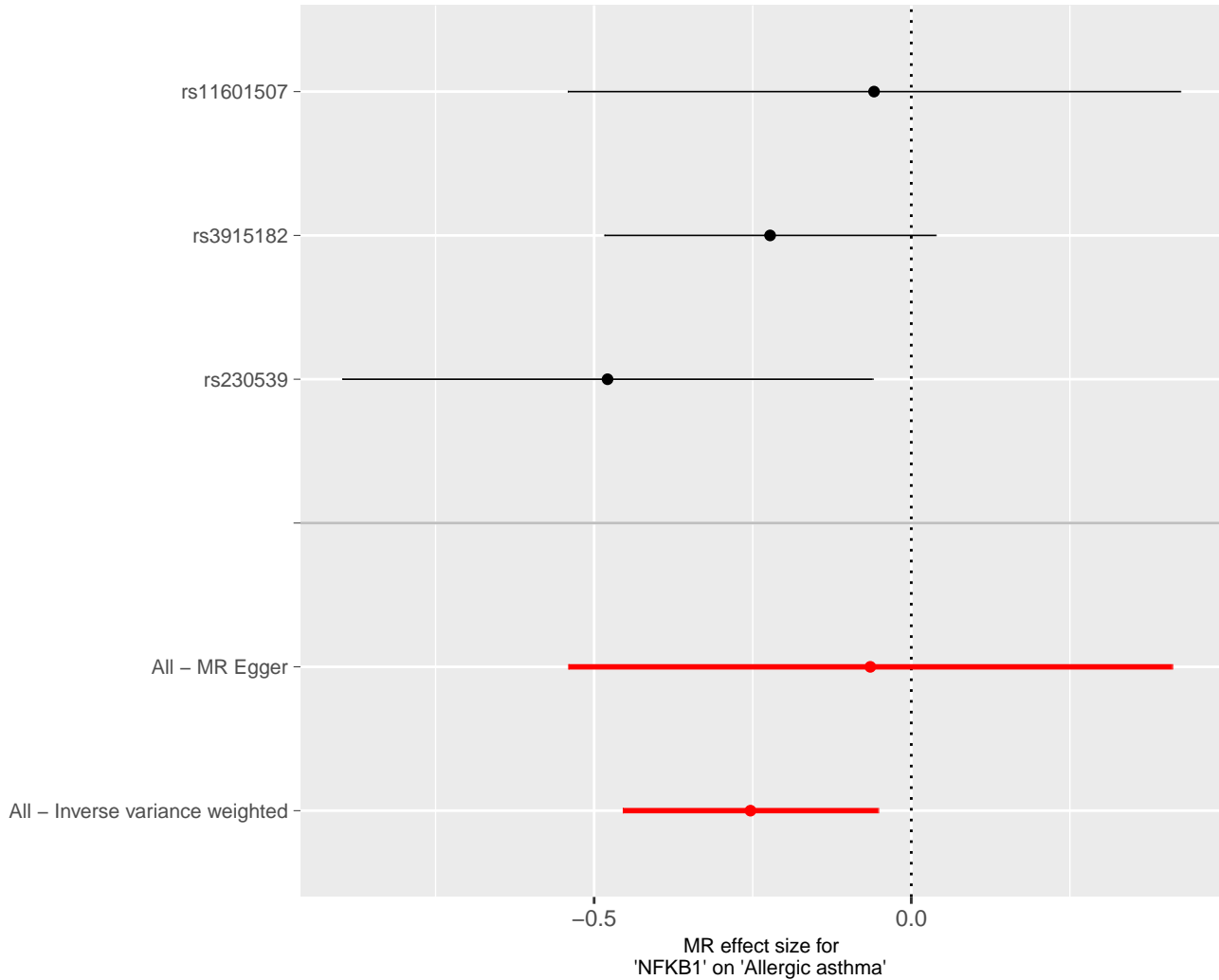

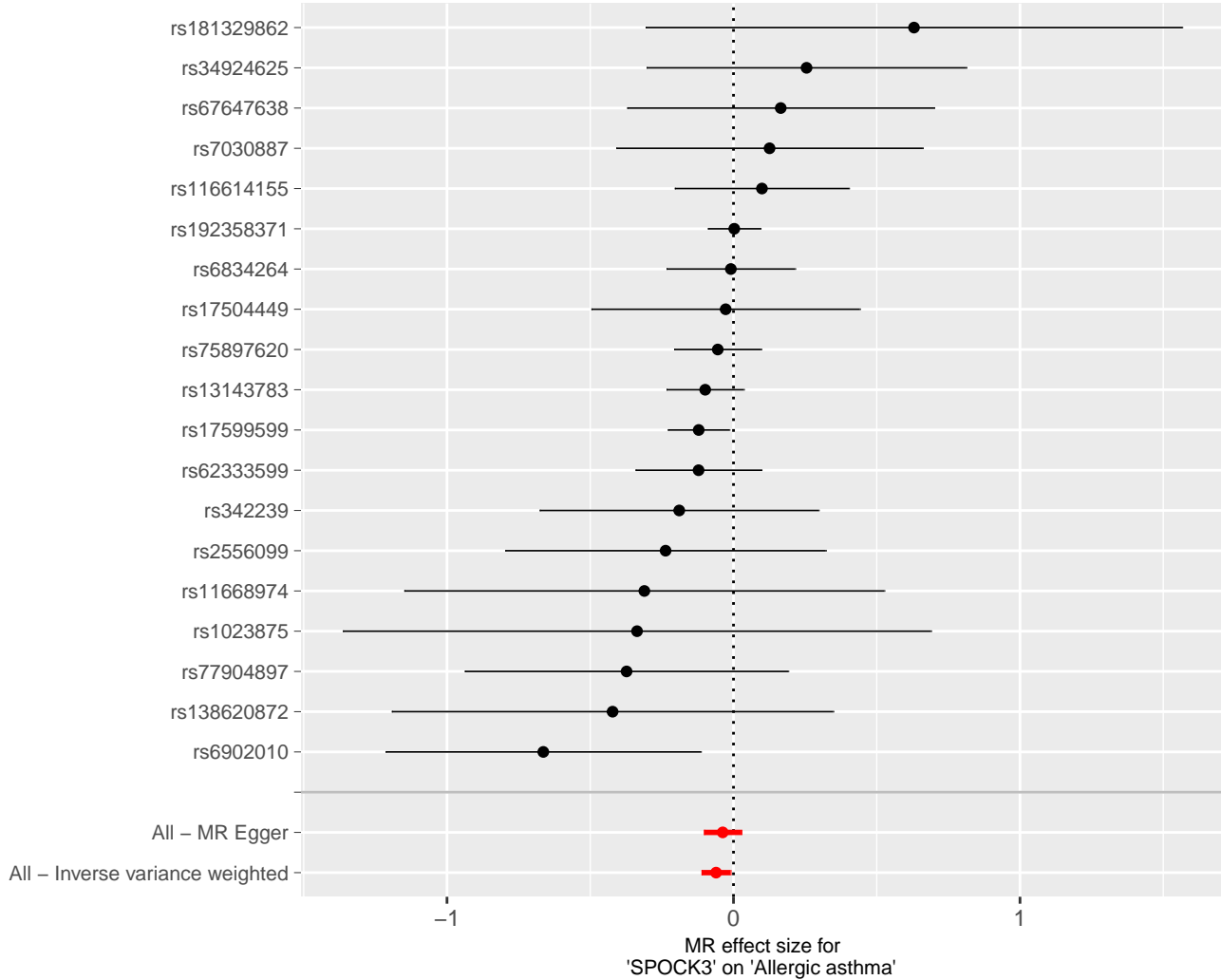

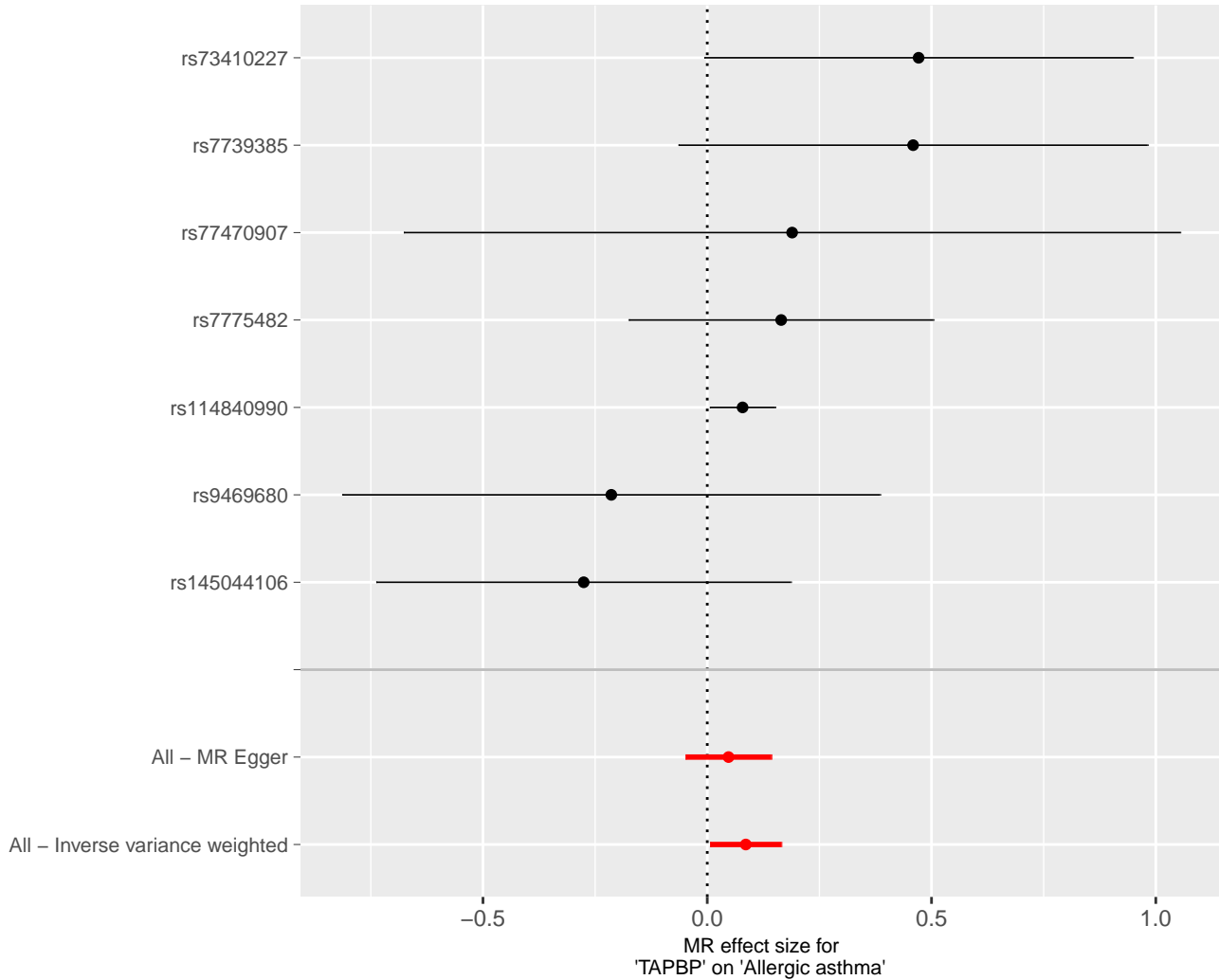

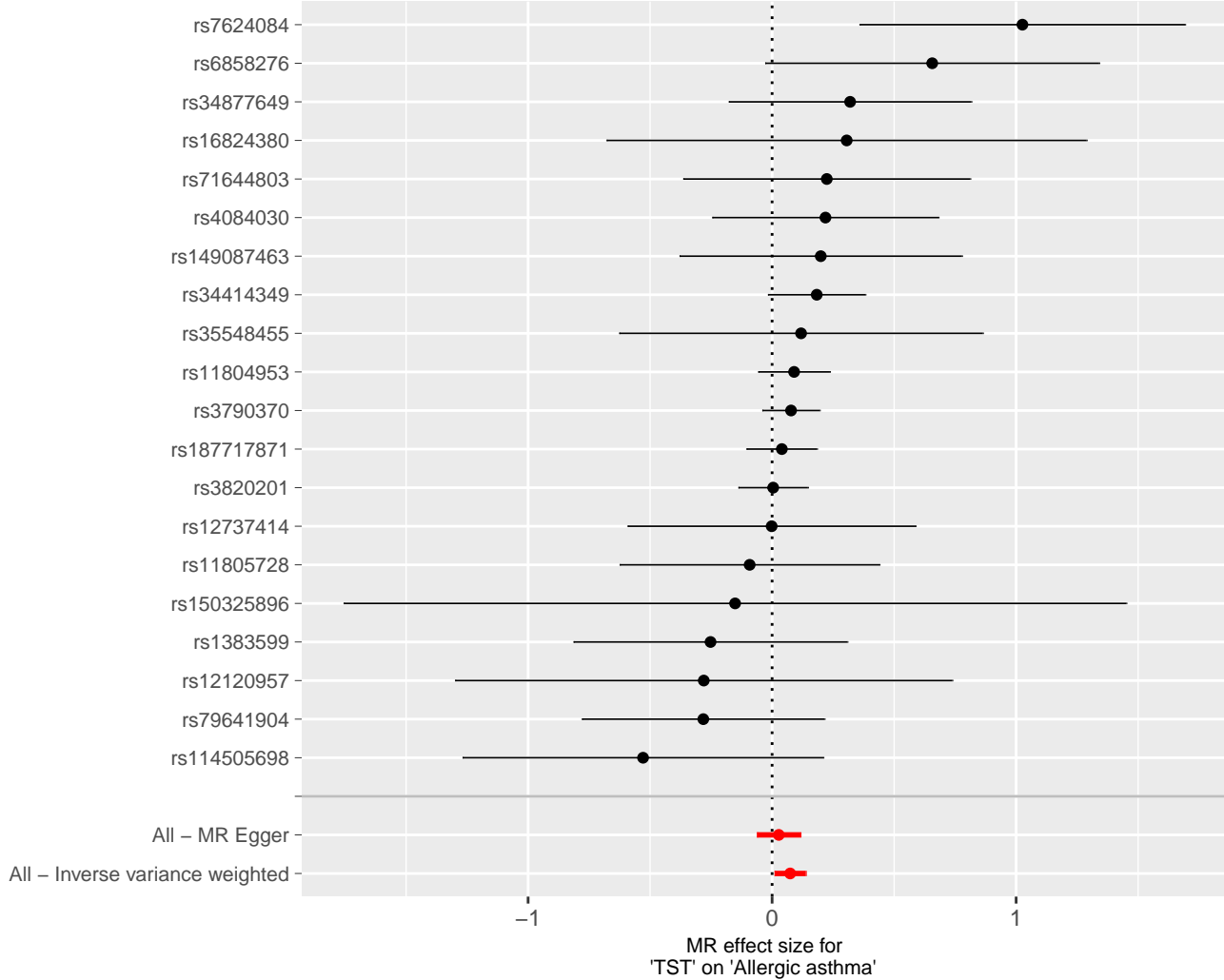

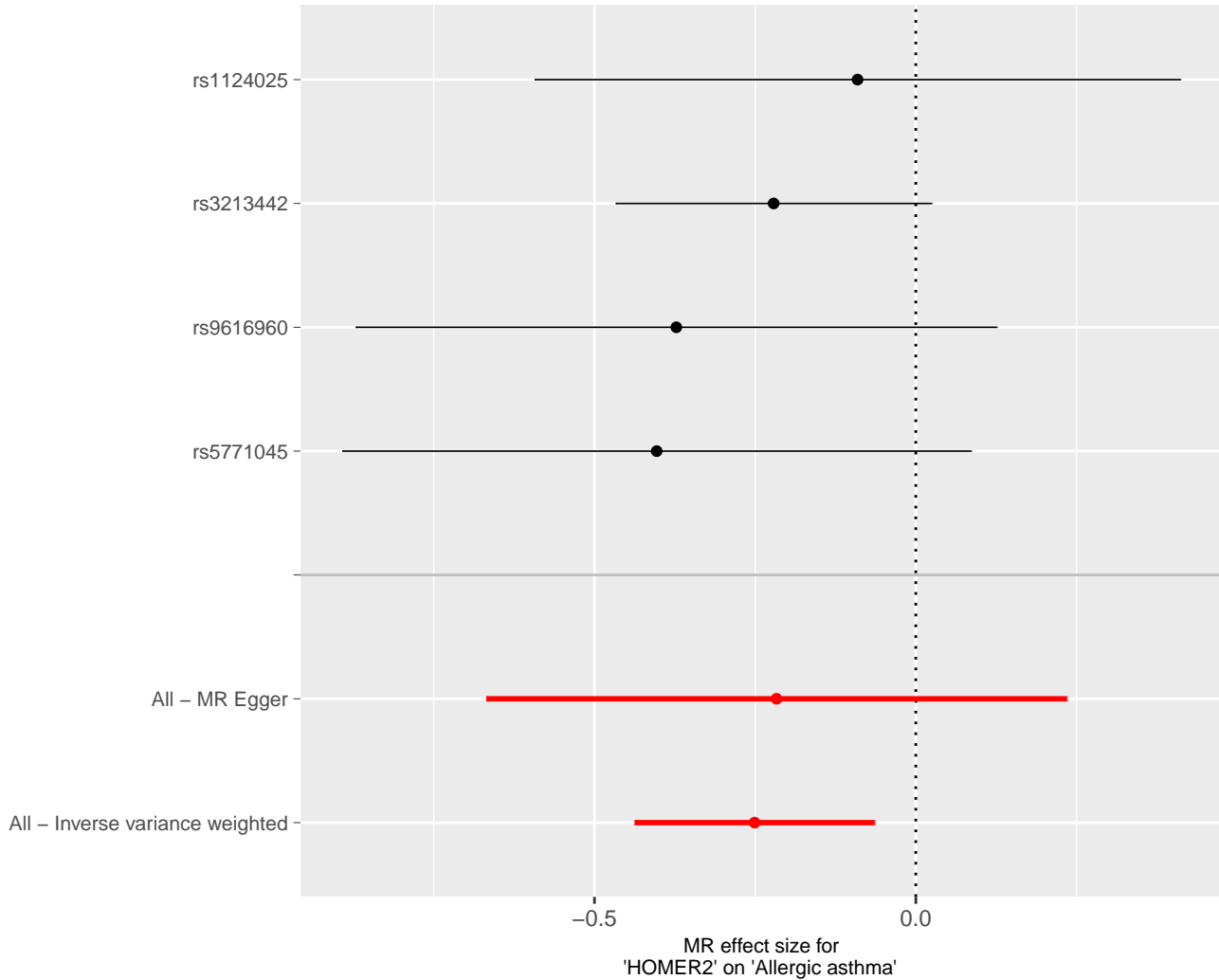

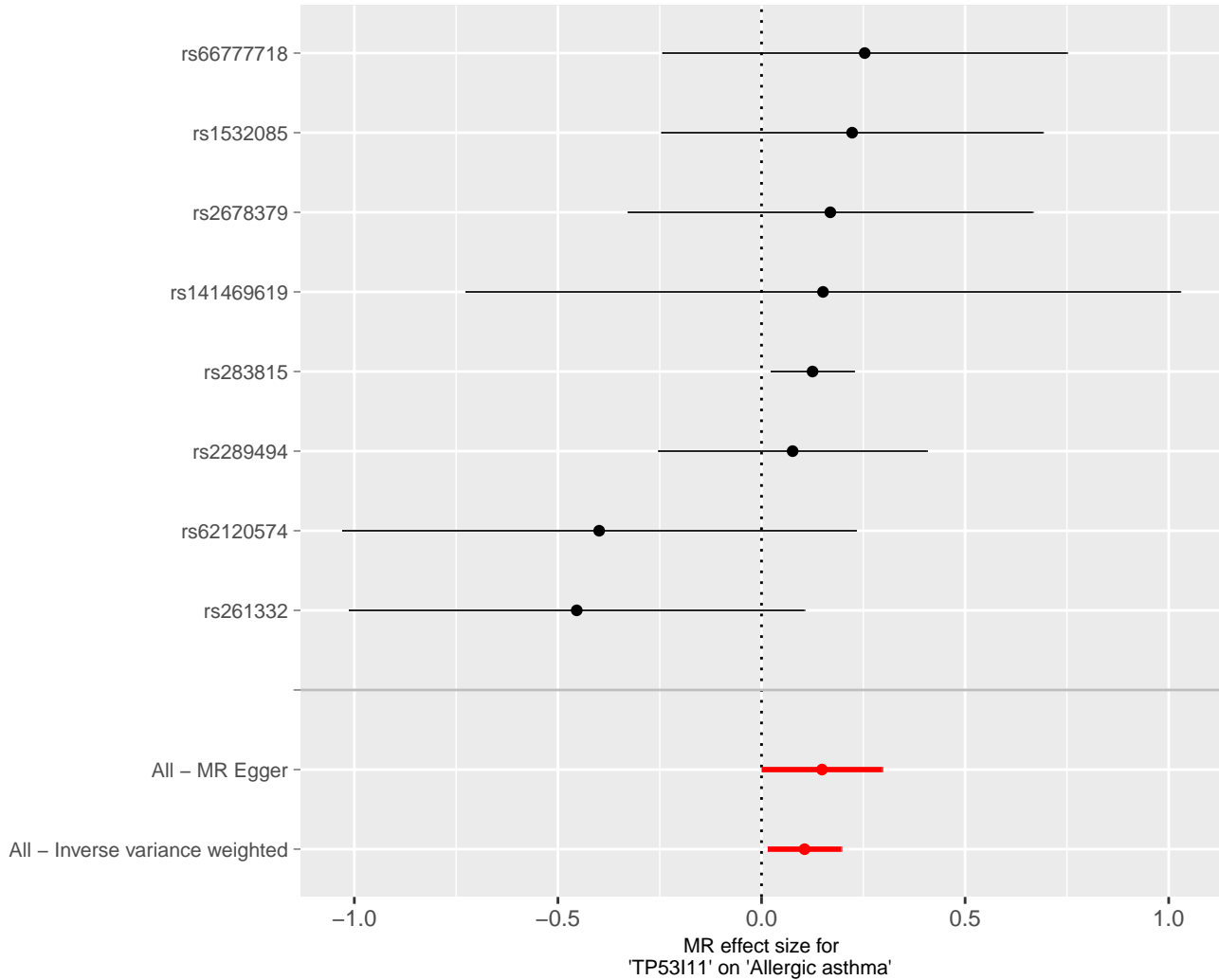

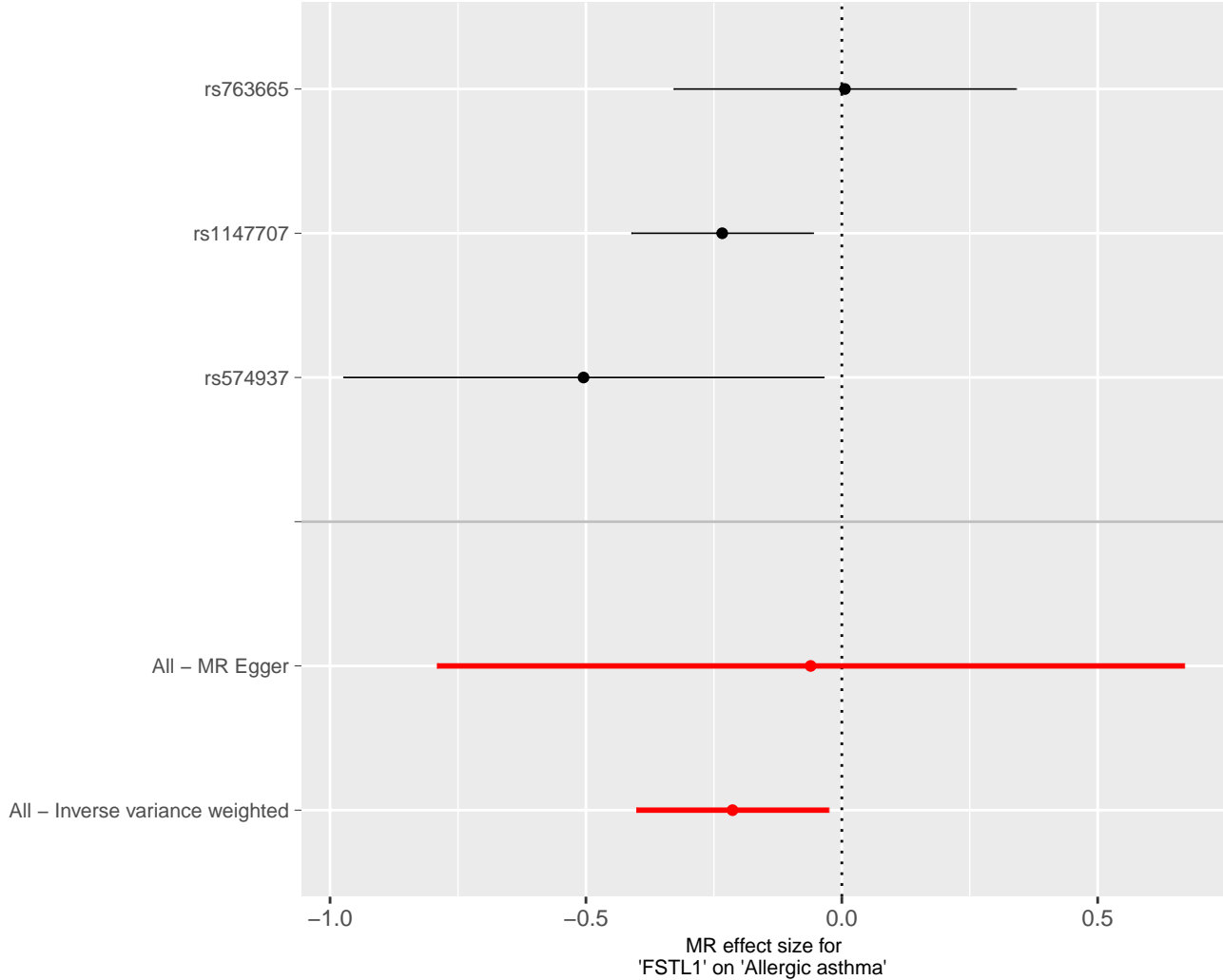

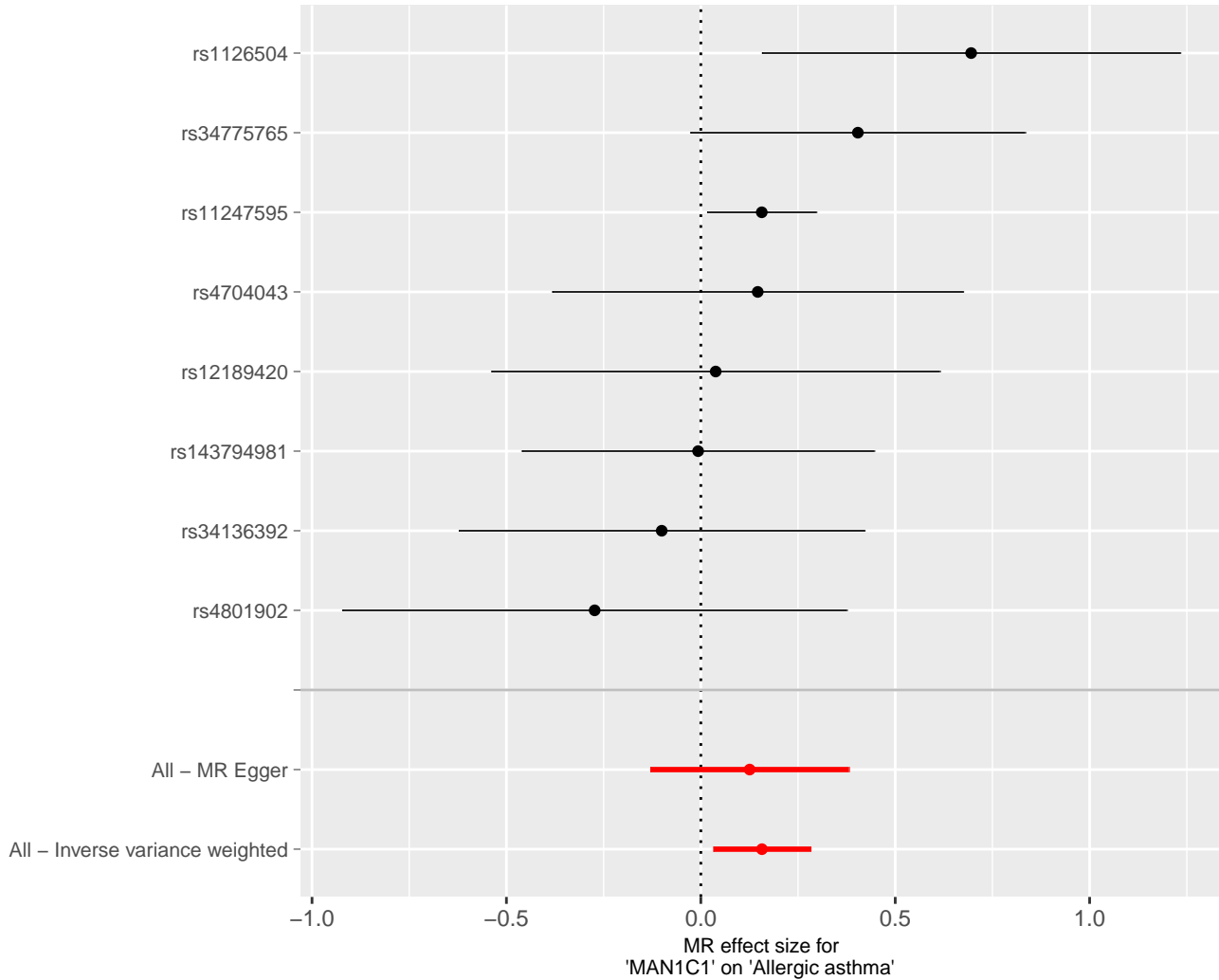

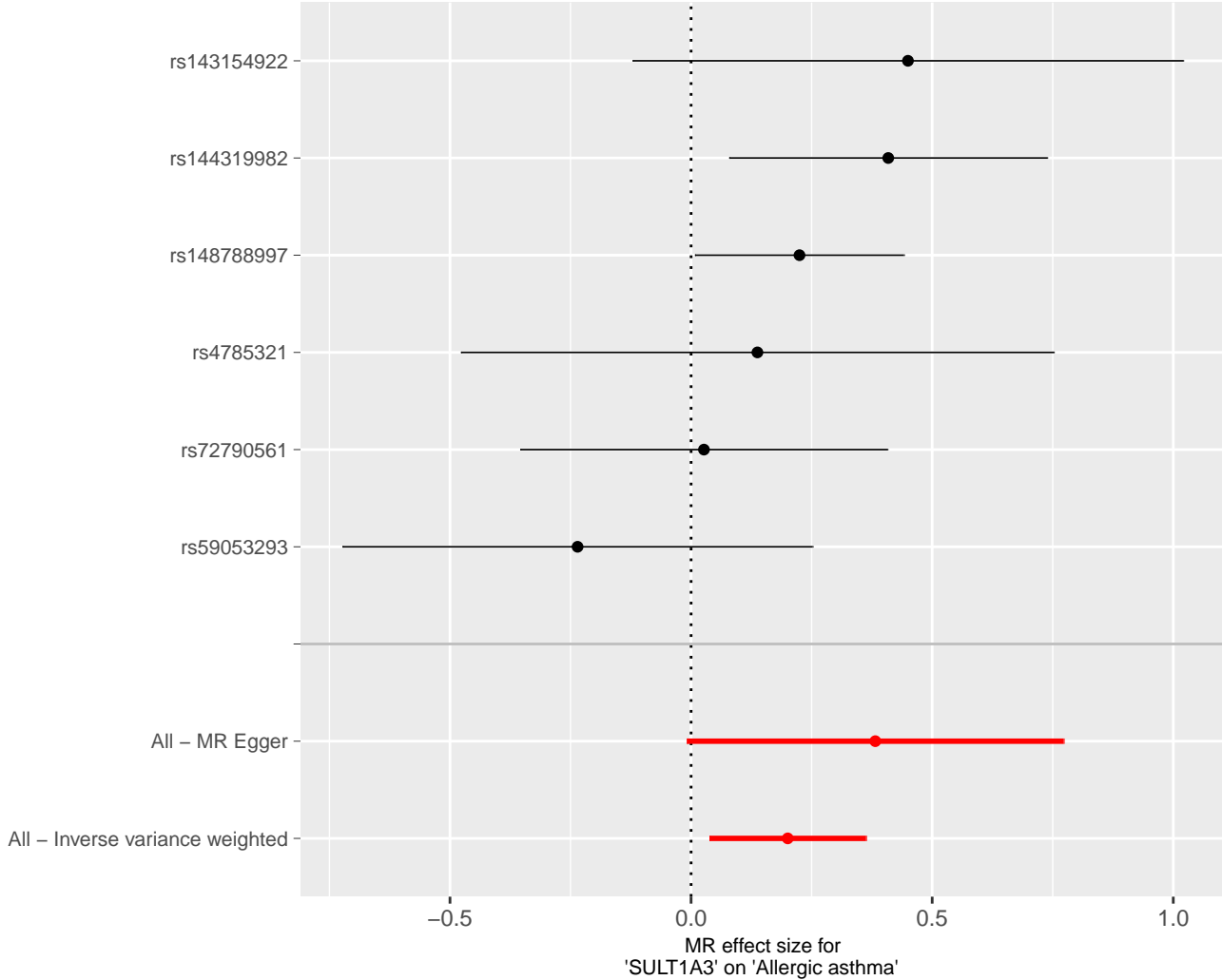

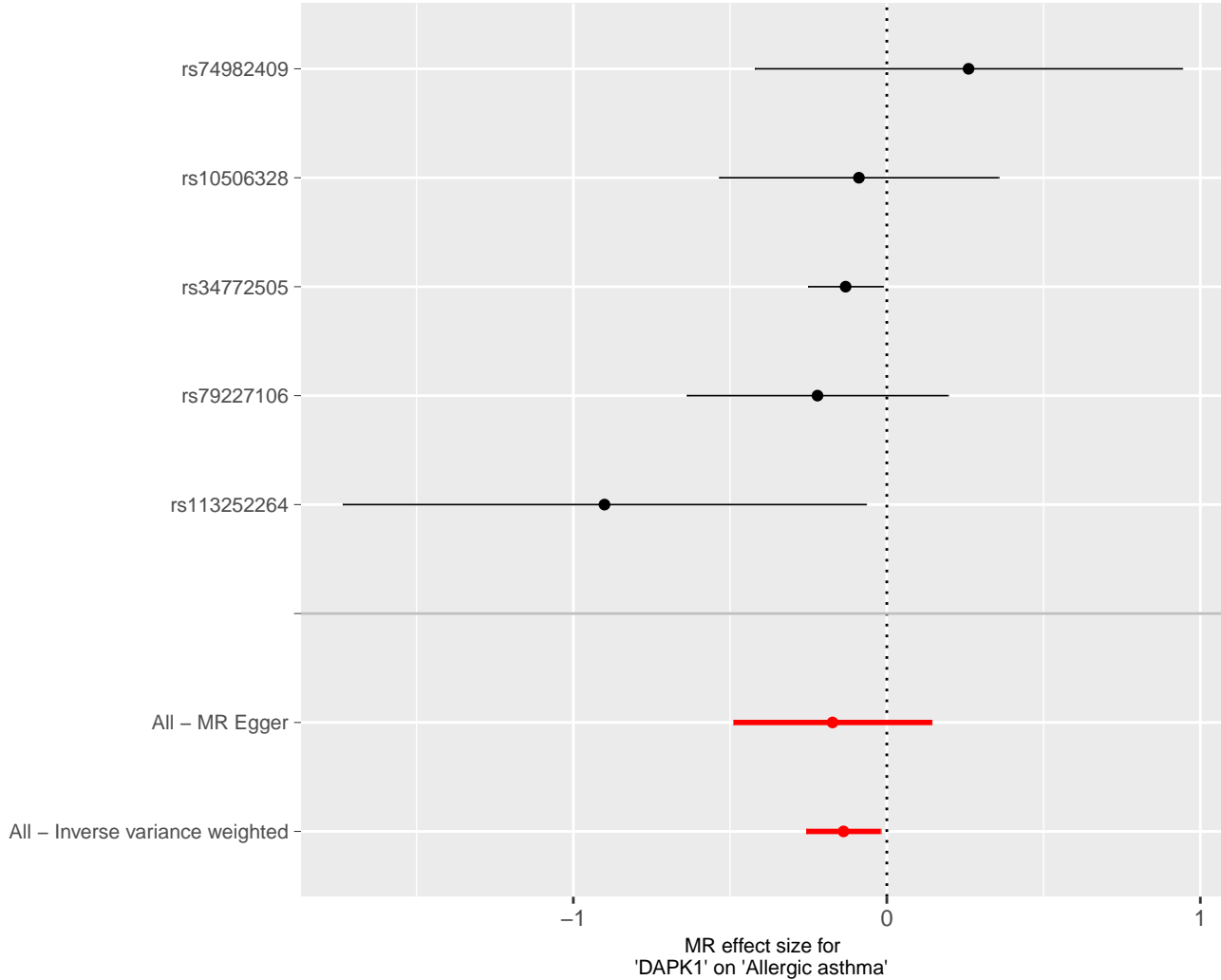

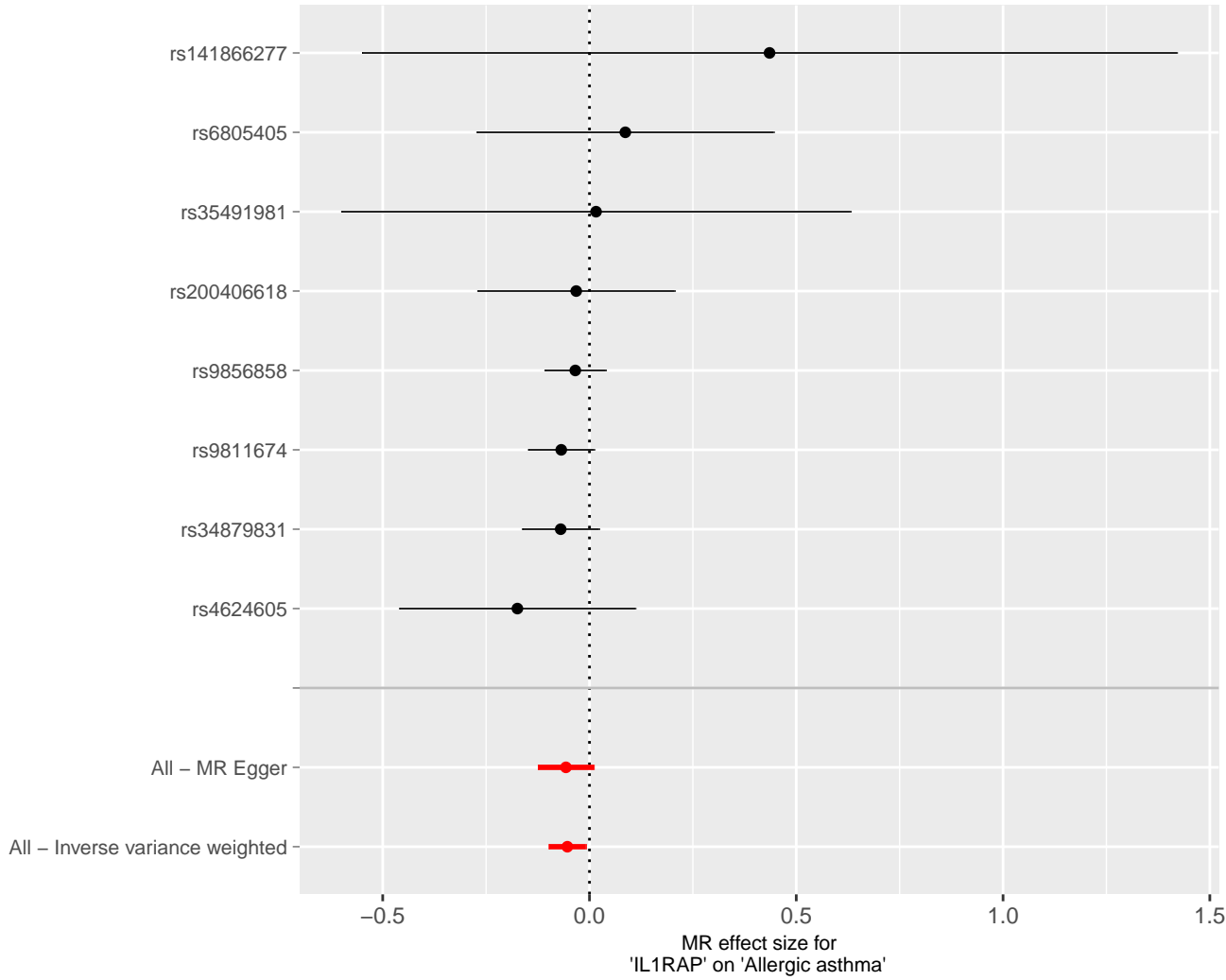

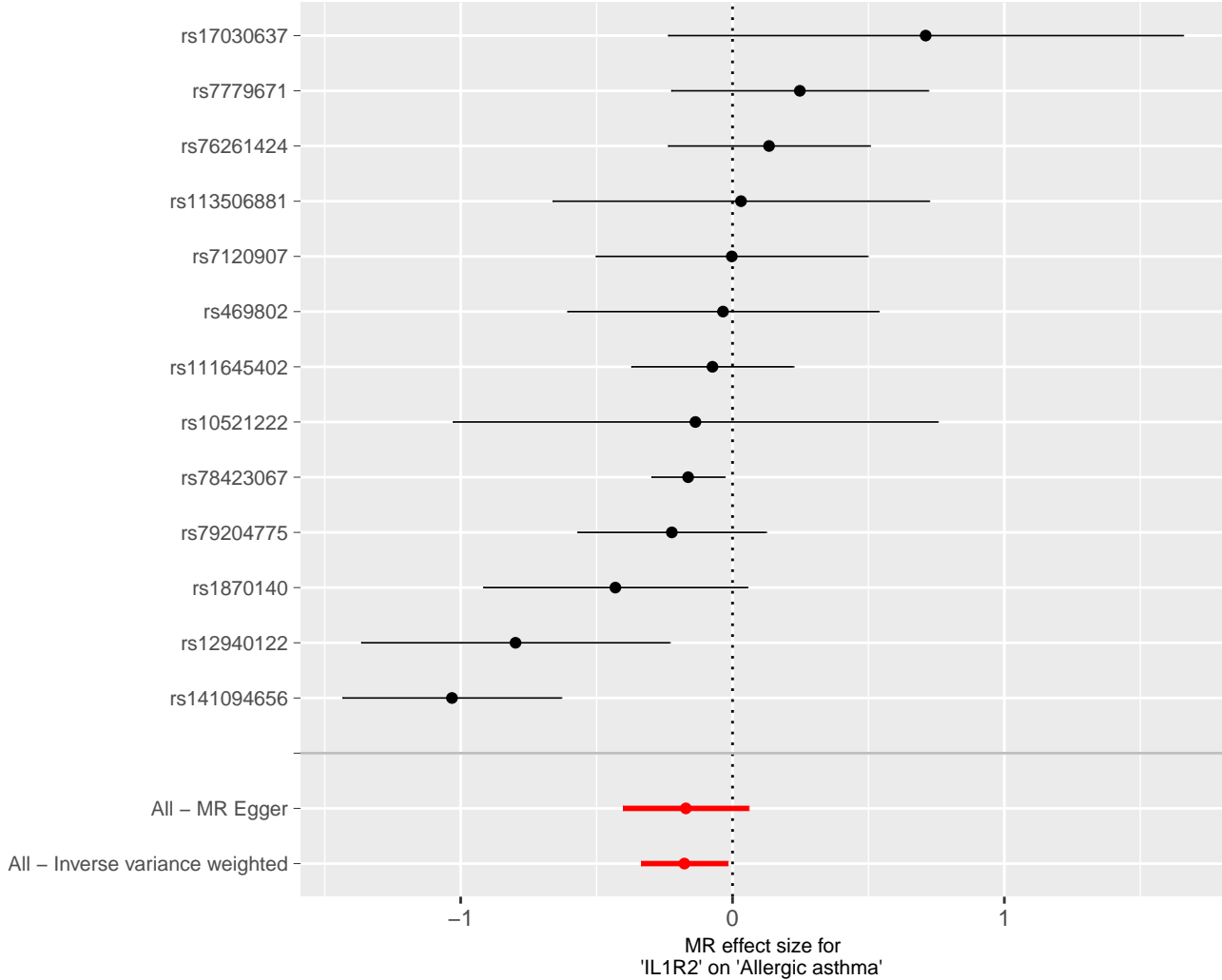

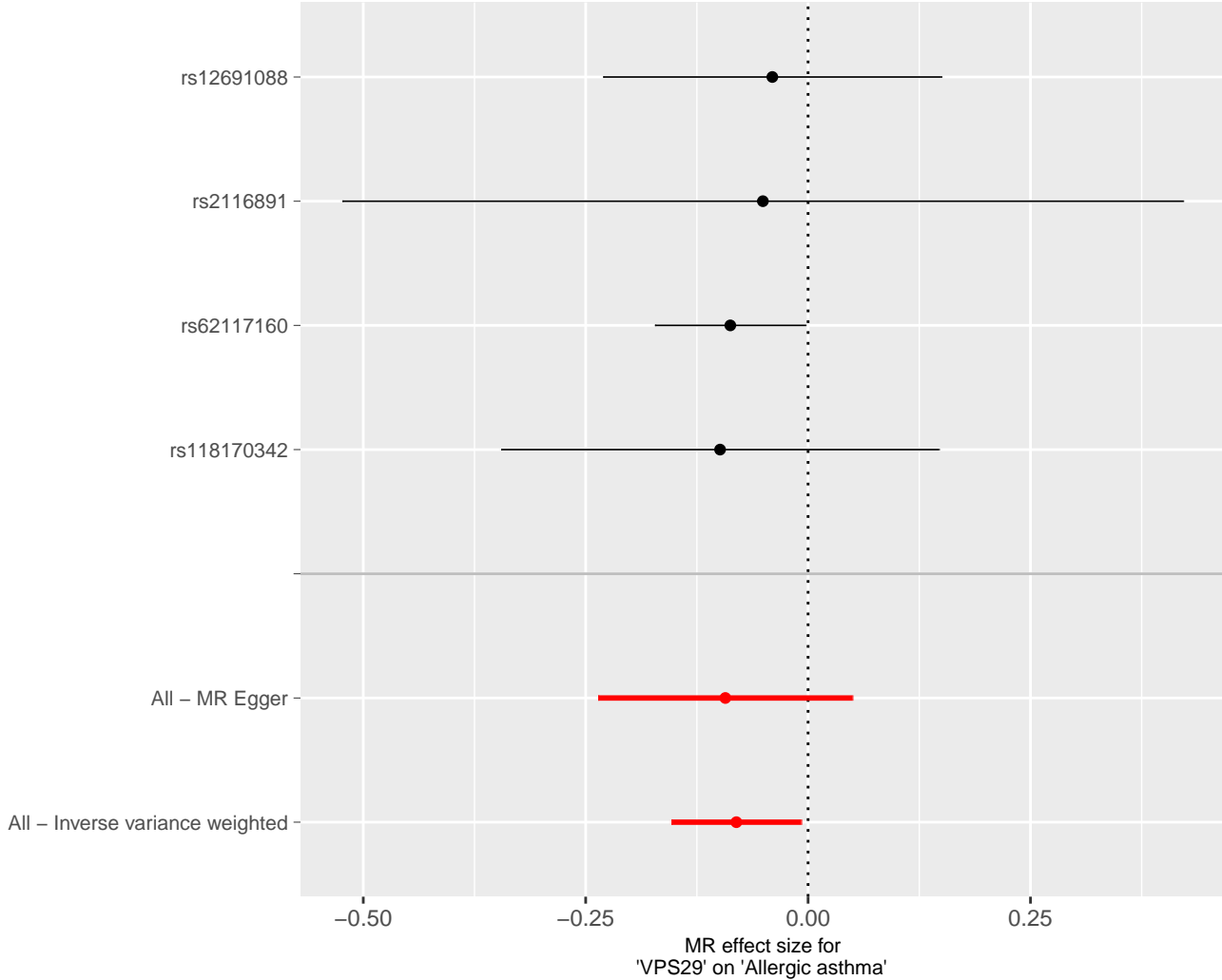

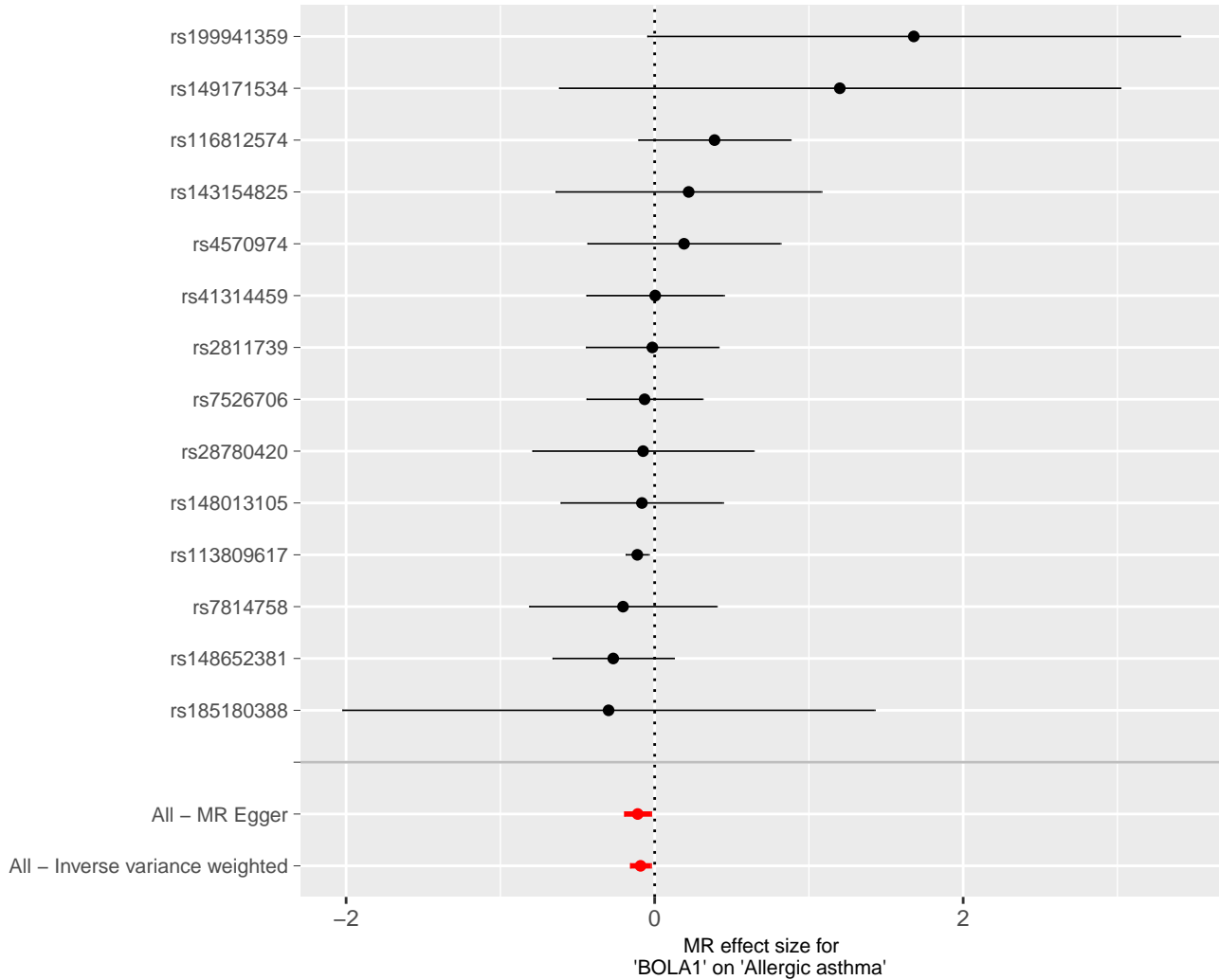

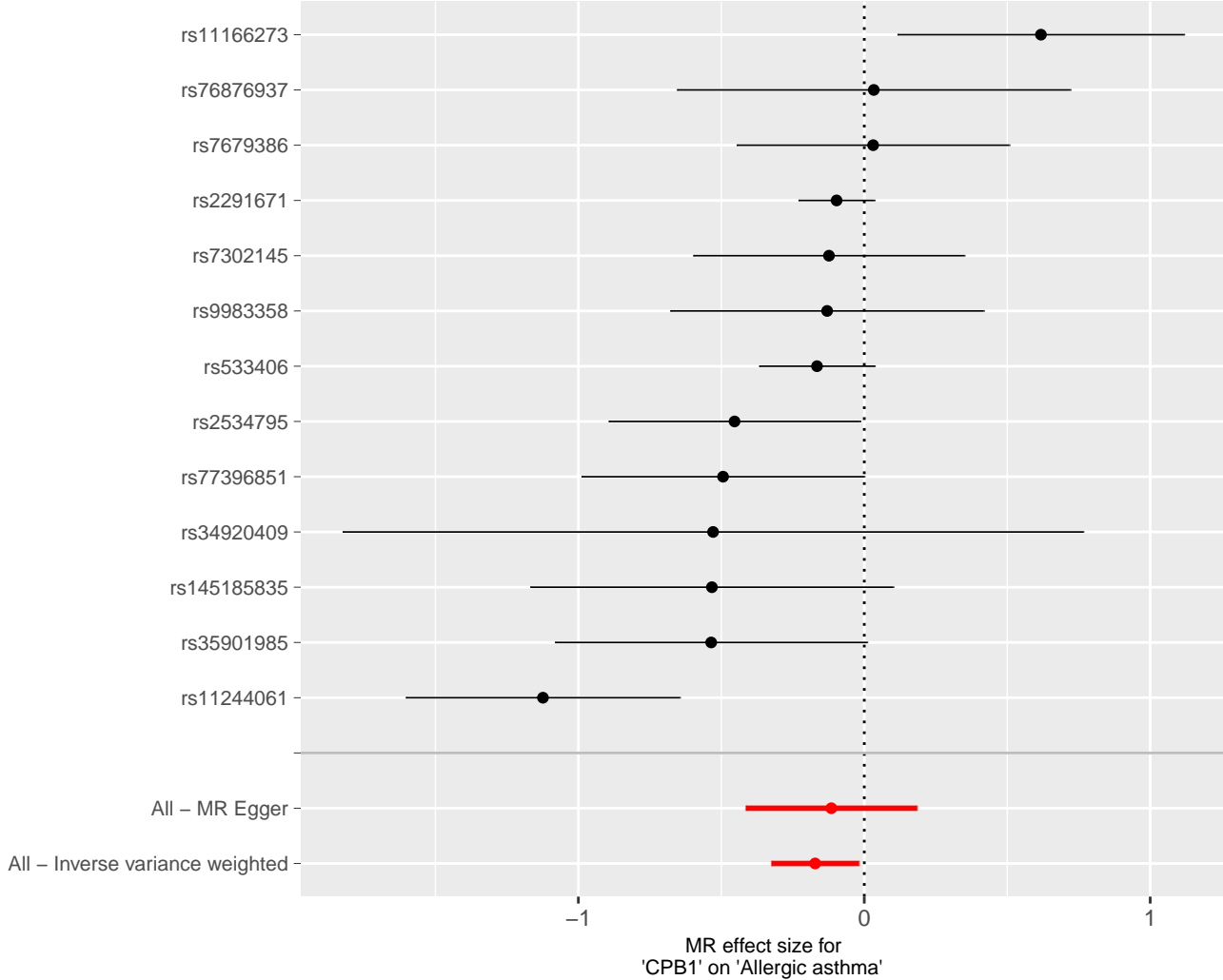

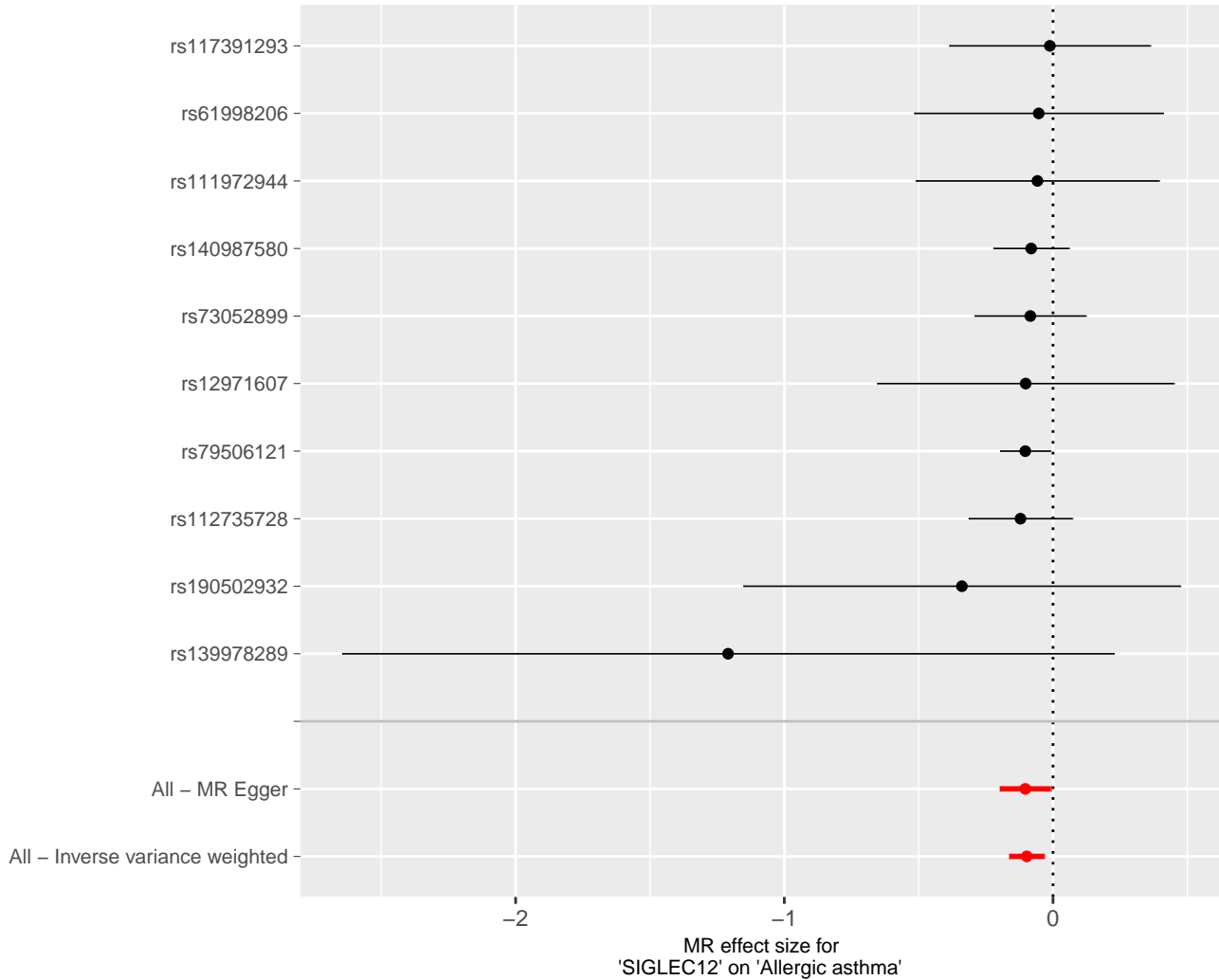

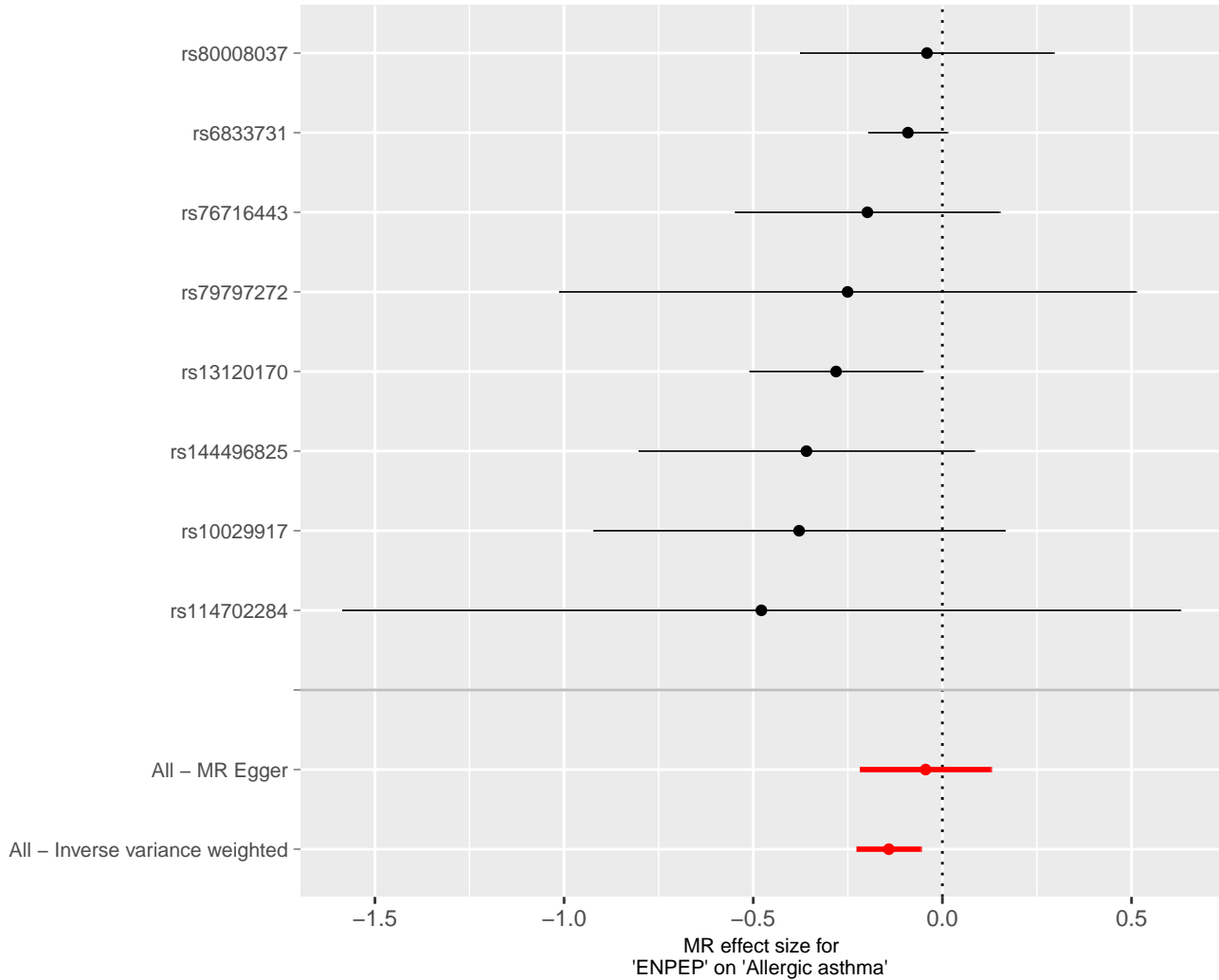

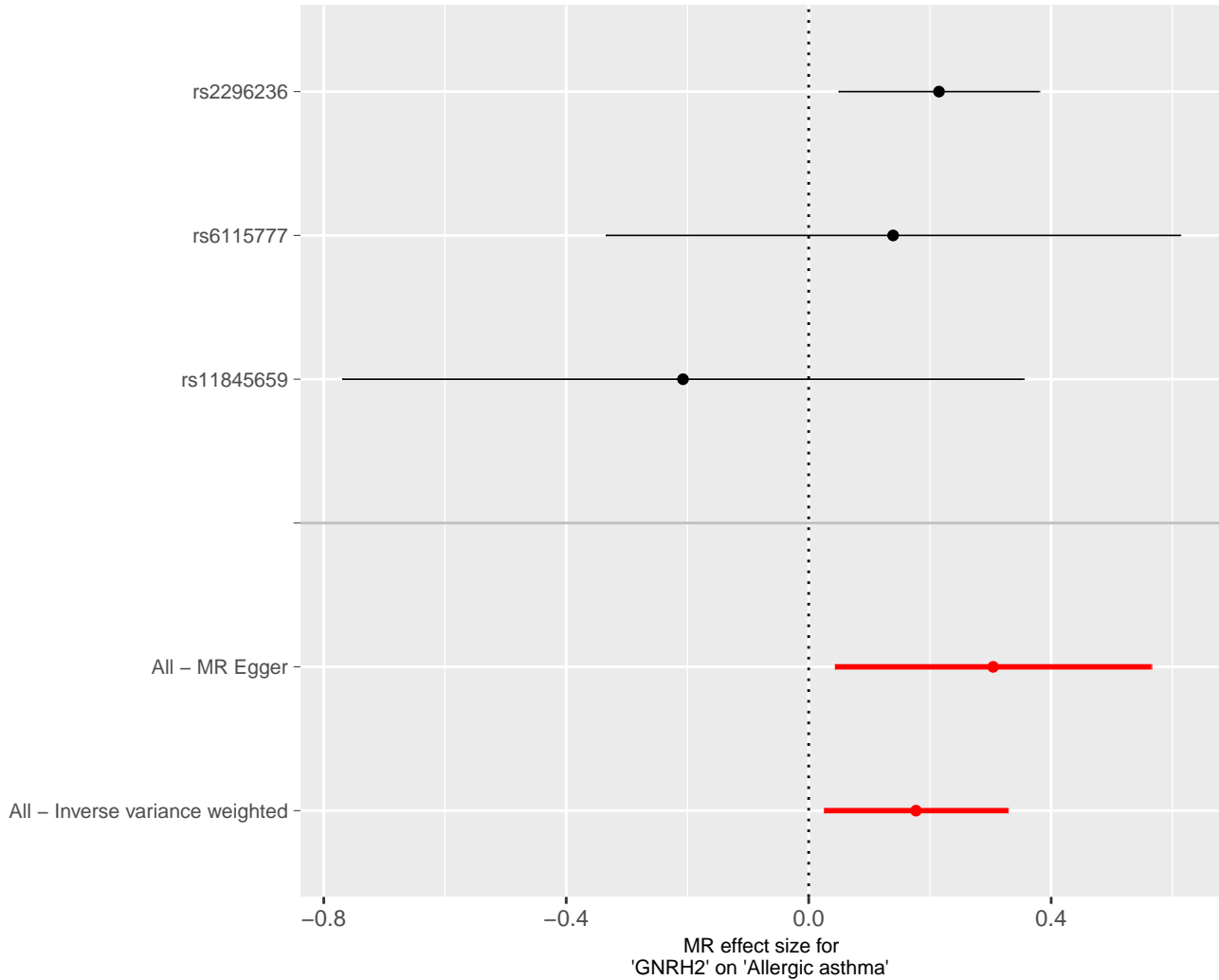

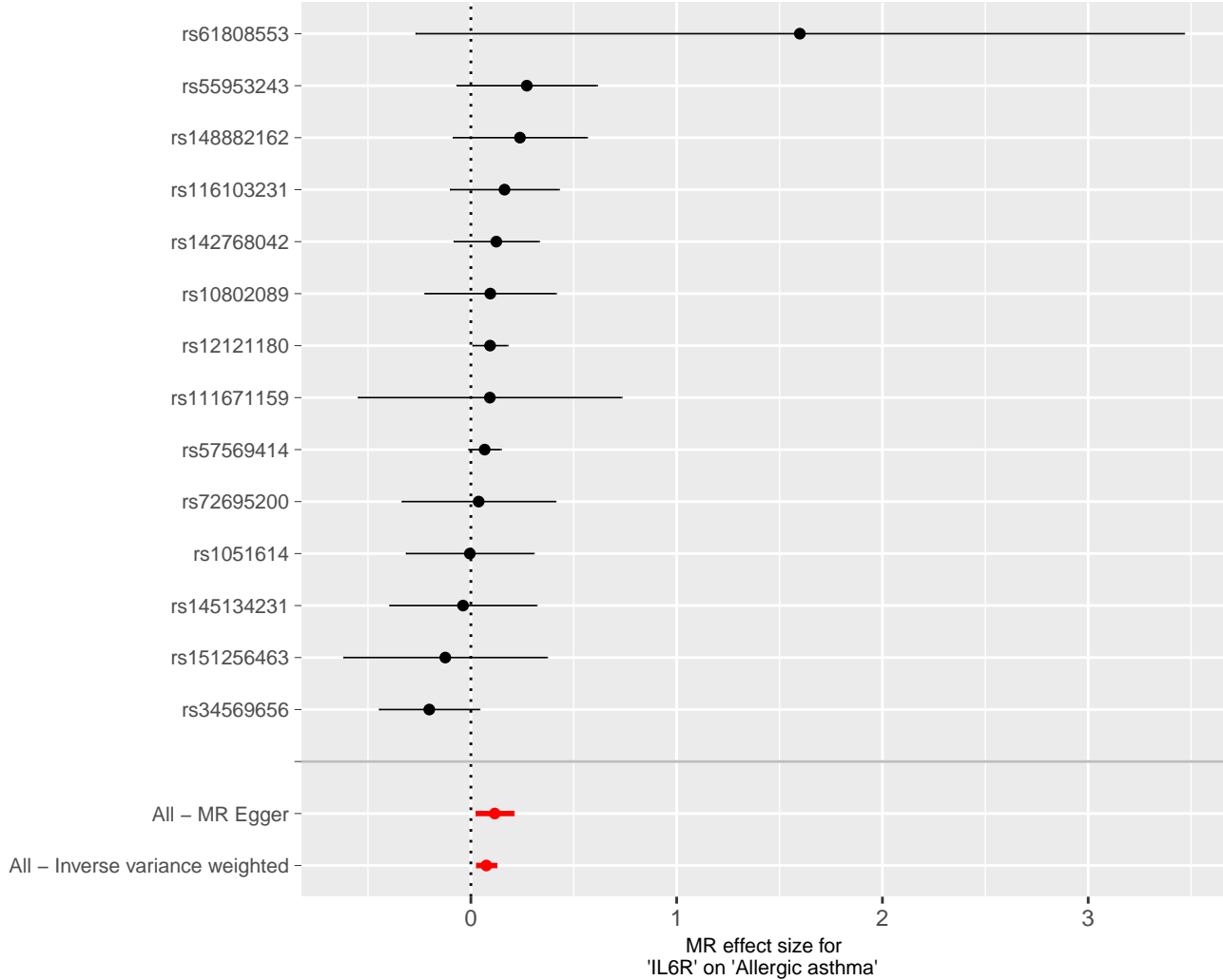

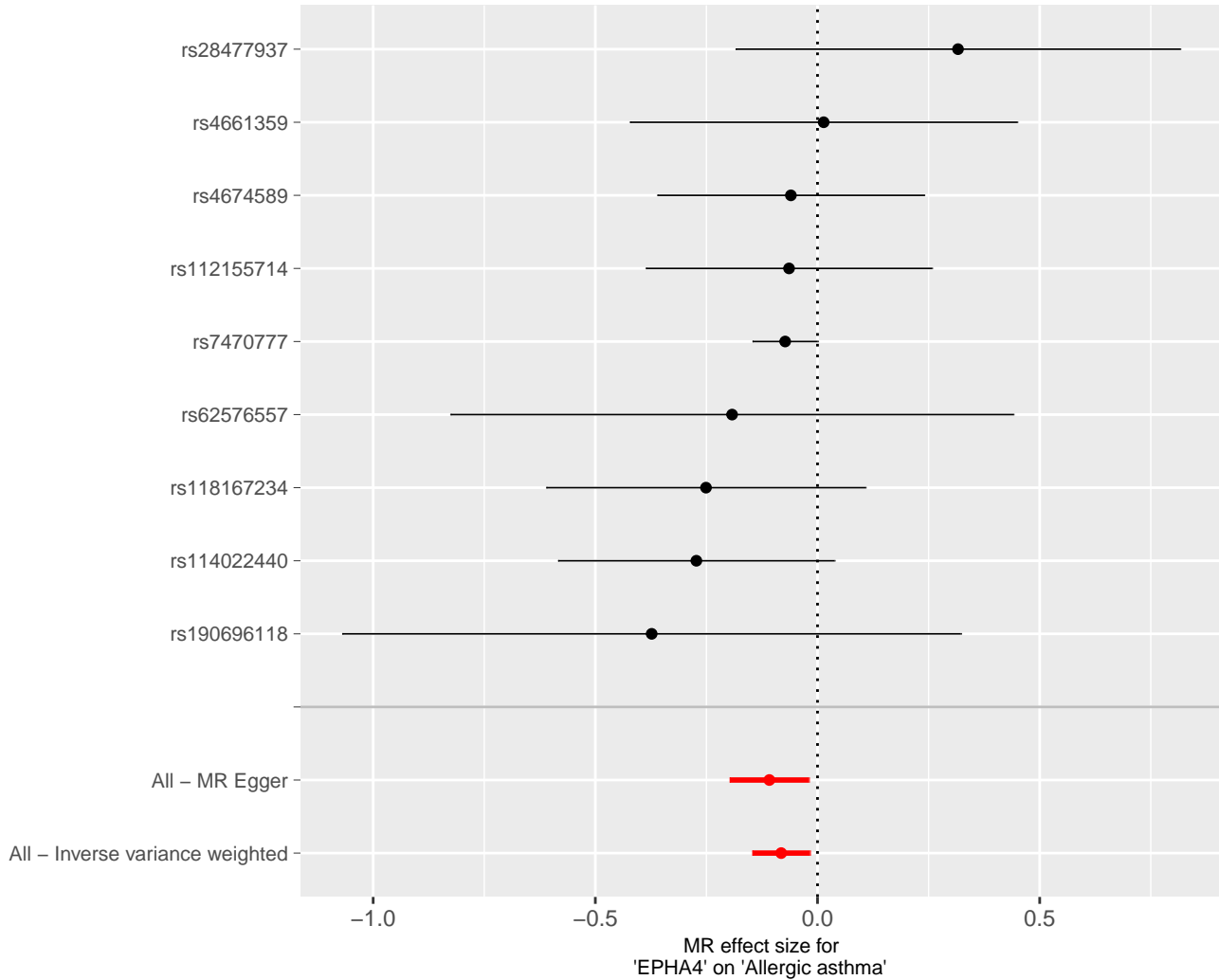

Supplement: Supplementary file 1 — Supplementary Material 1: Supplementary Materials 1. The MR results of BMI and allergic asthma. Supplementary Materials 2. The MR results of plasma proteins and allergic asthma. Supplementary Materials 3. The MR results of BMI and plasma proteins. Supplementary Materials 4. The MR results of mediation factor plasma proteins and allergic asthma. Supplementary Materials 5. The results of drug-targeted MR [file 41065_2025_376_MOESM1_ESM.zip › Supplementary Materials/S2 The MR results of plasma proteins and allergic asthma/S2 plasma proteins-Allergic asthma.forest.pdf]

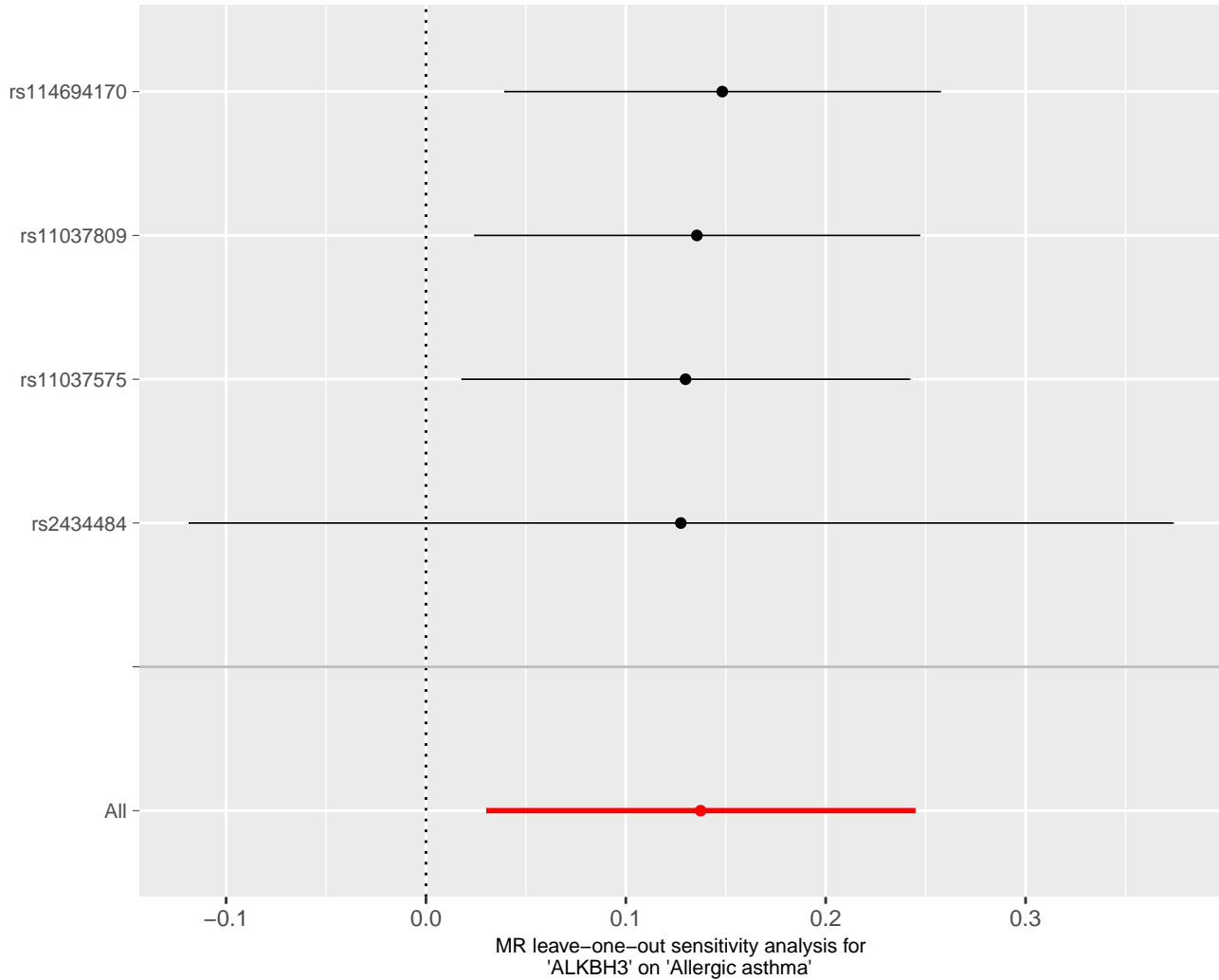

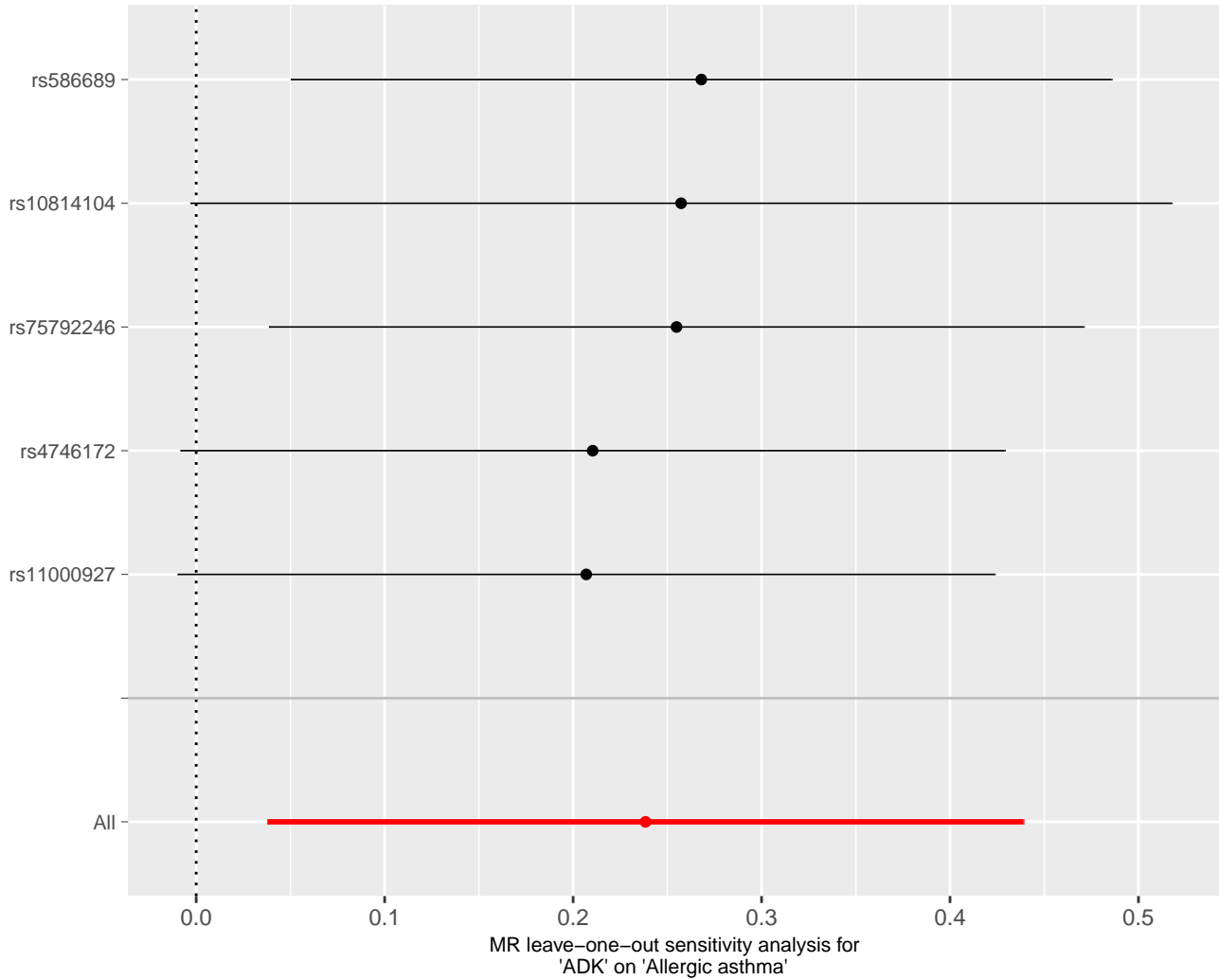

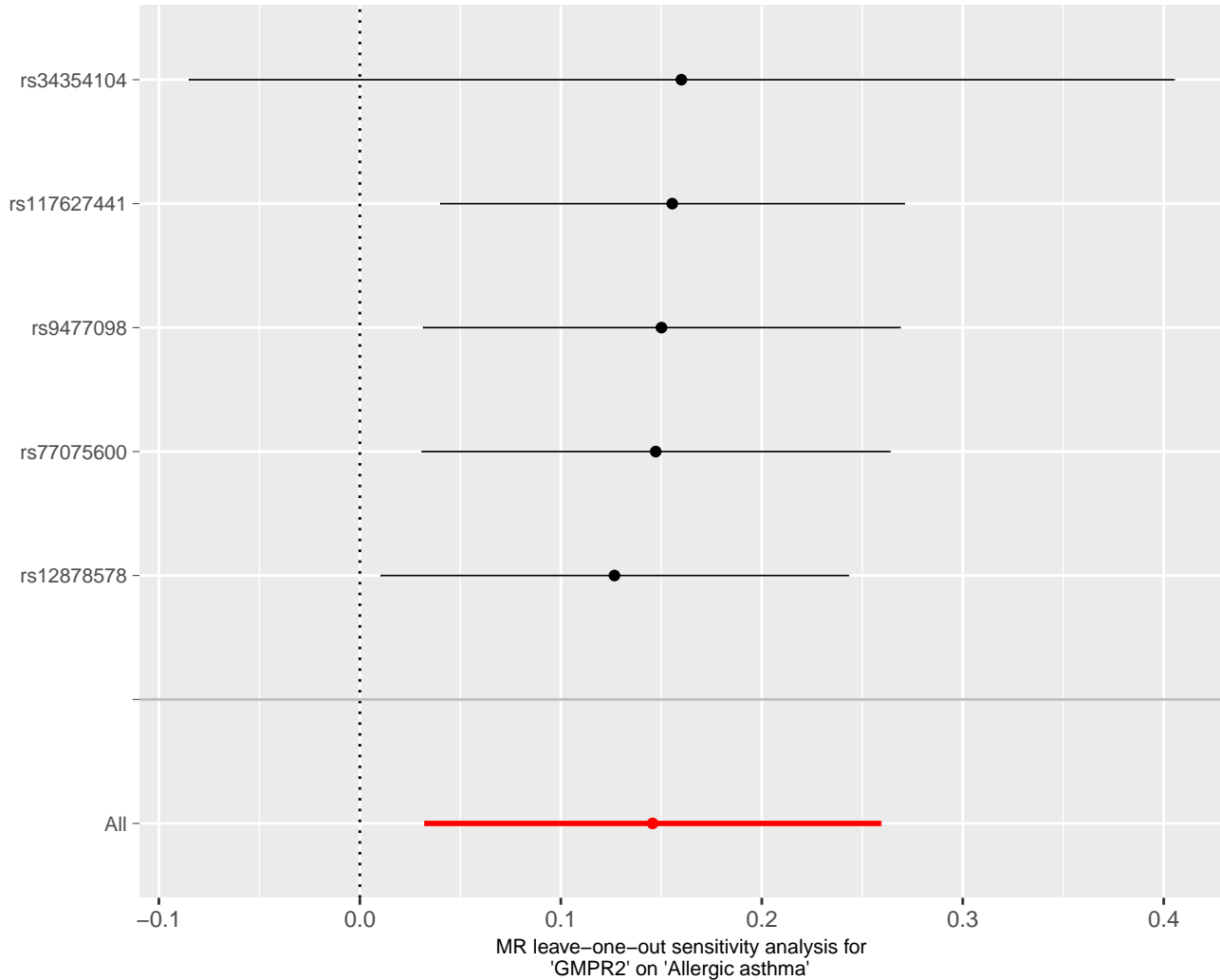

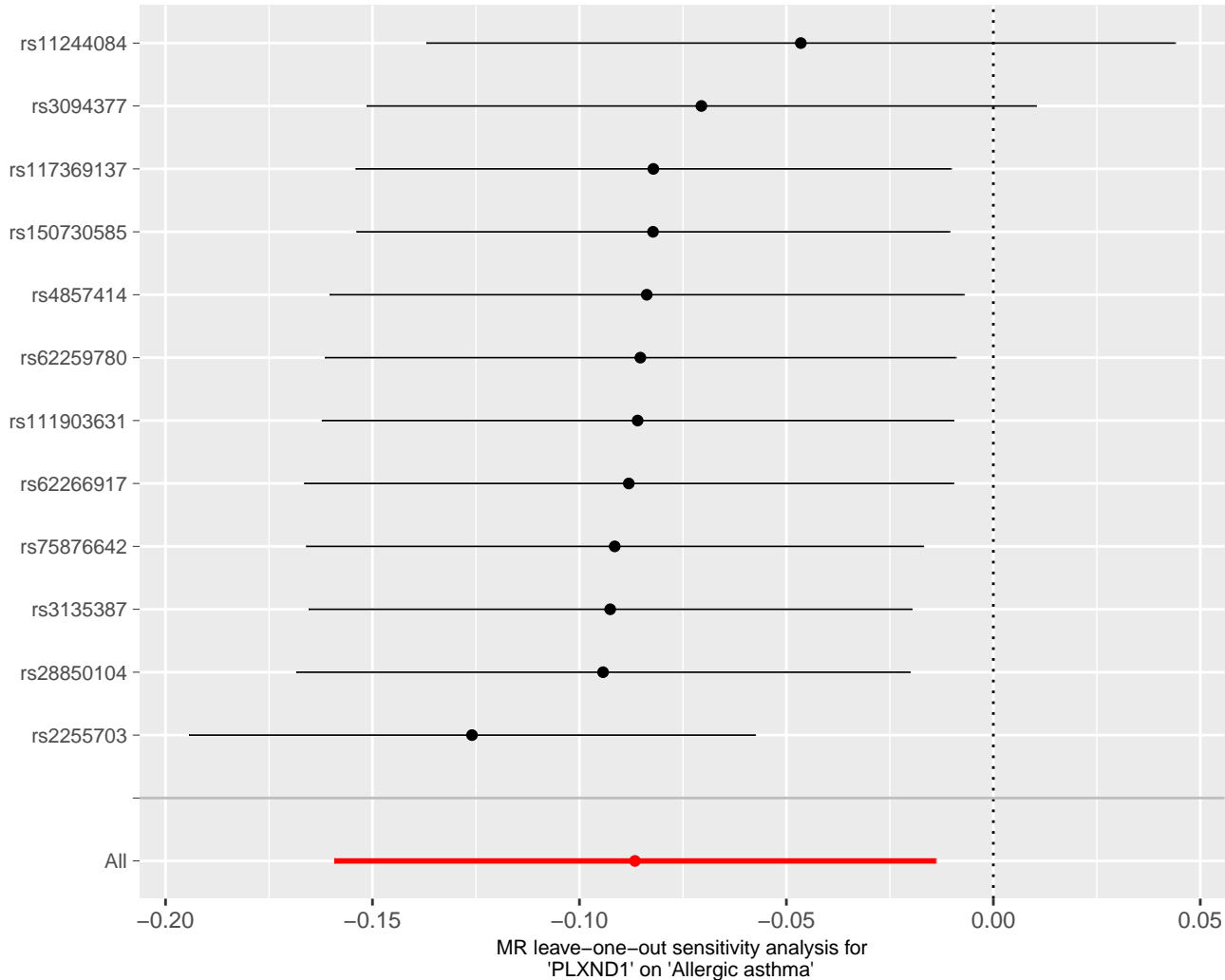

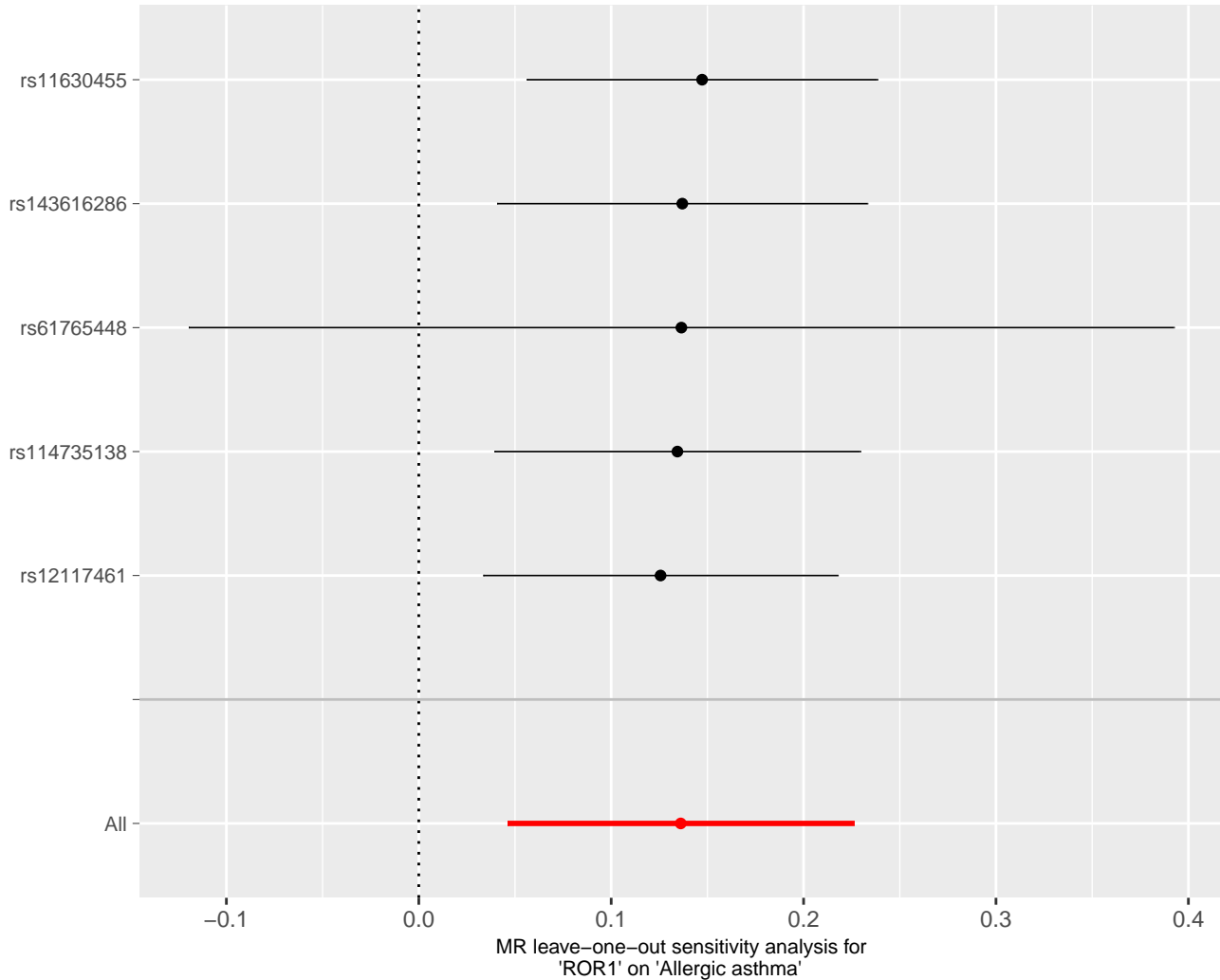

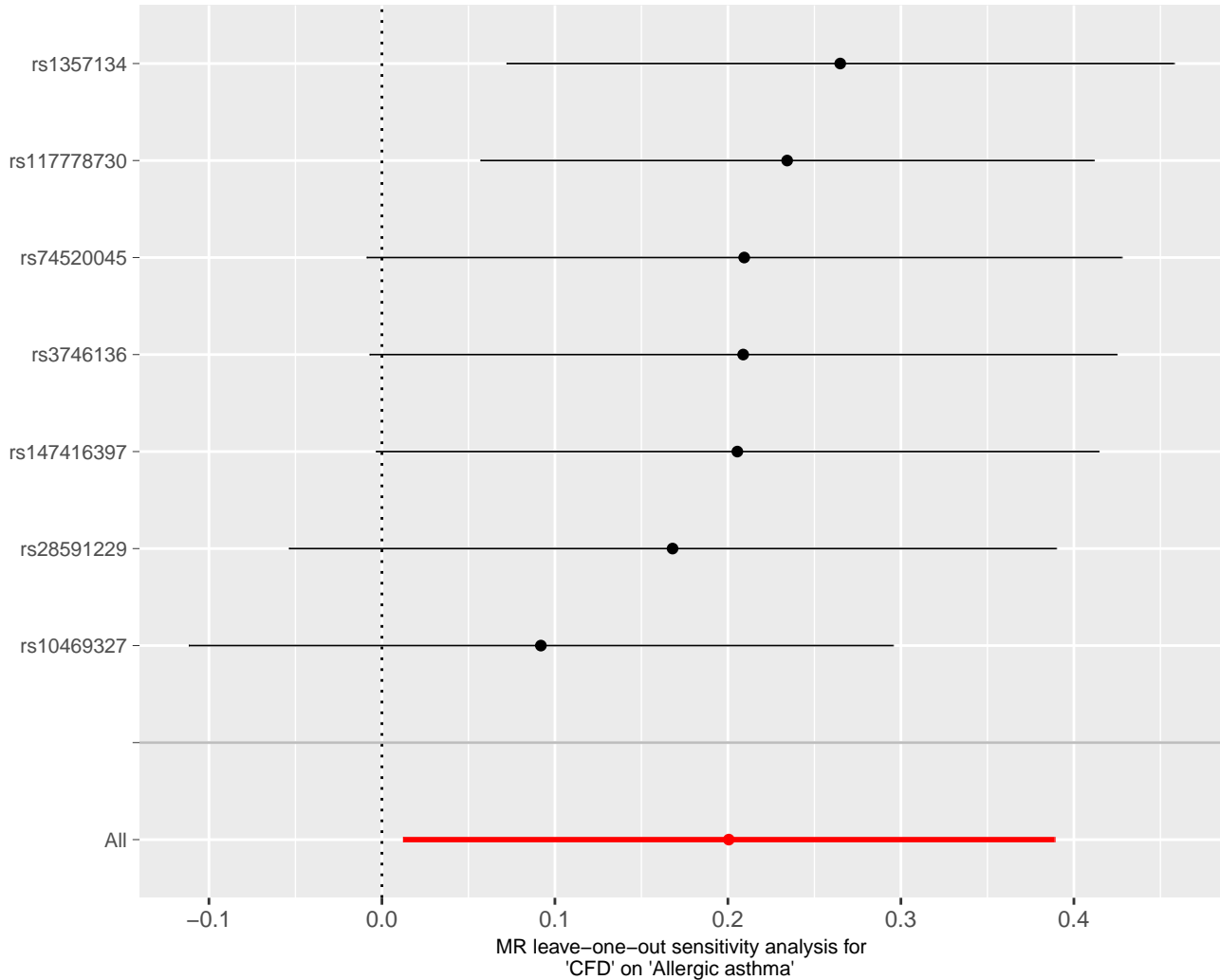

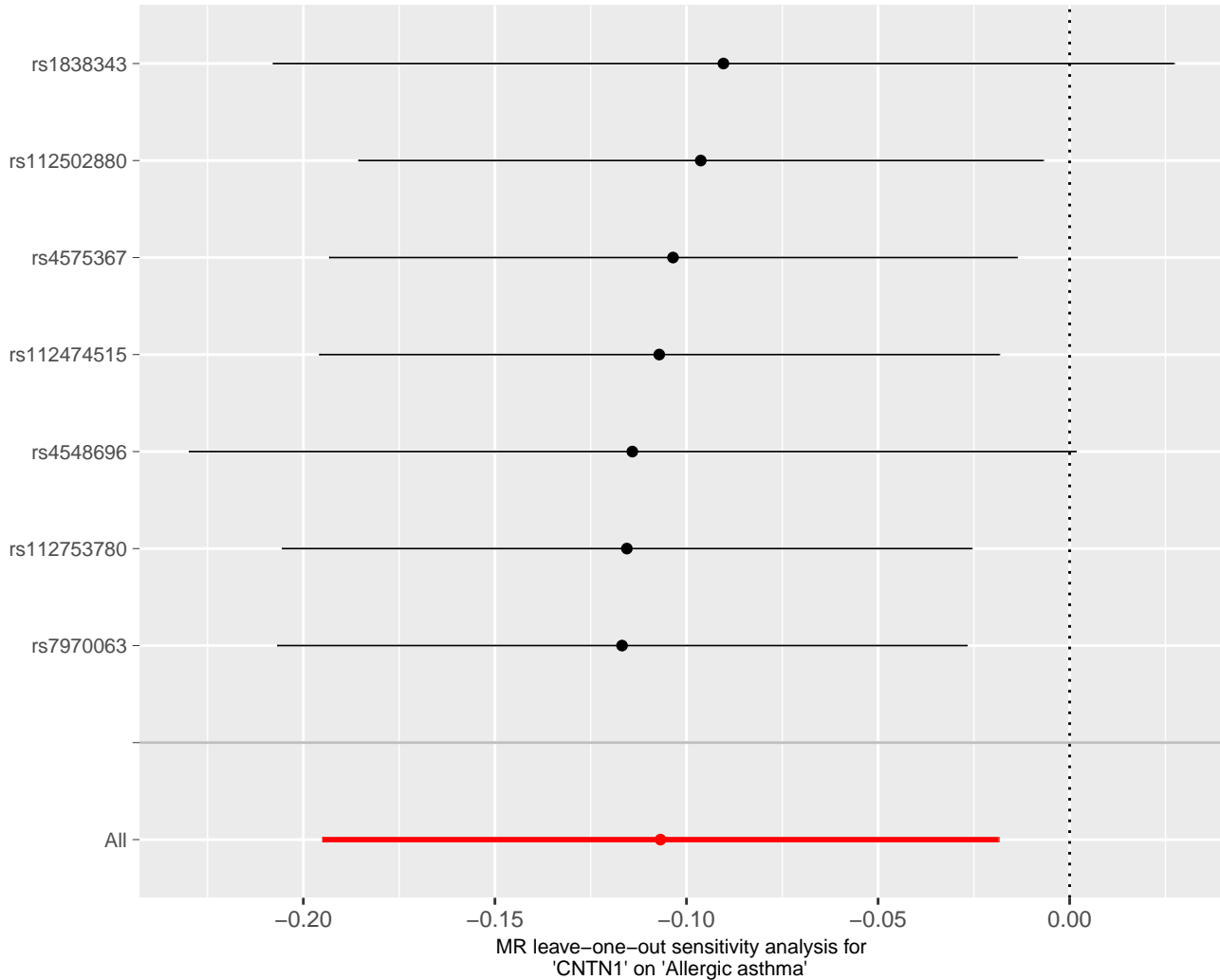

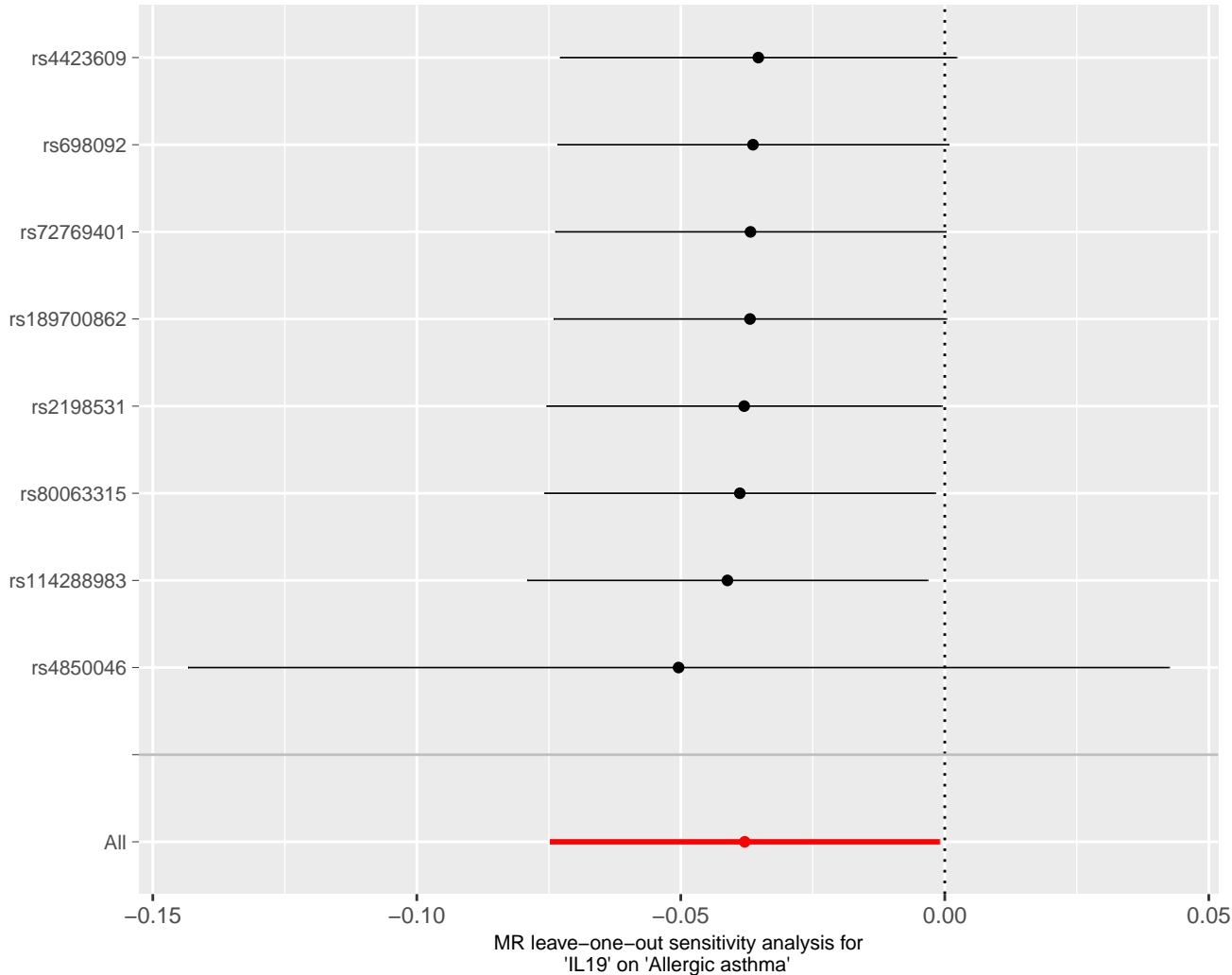

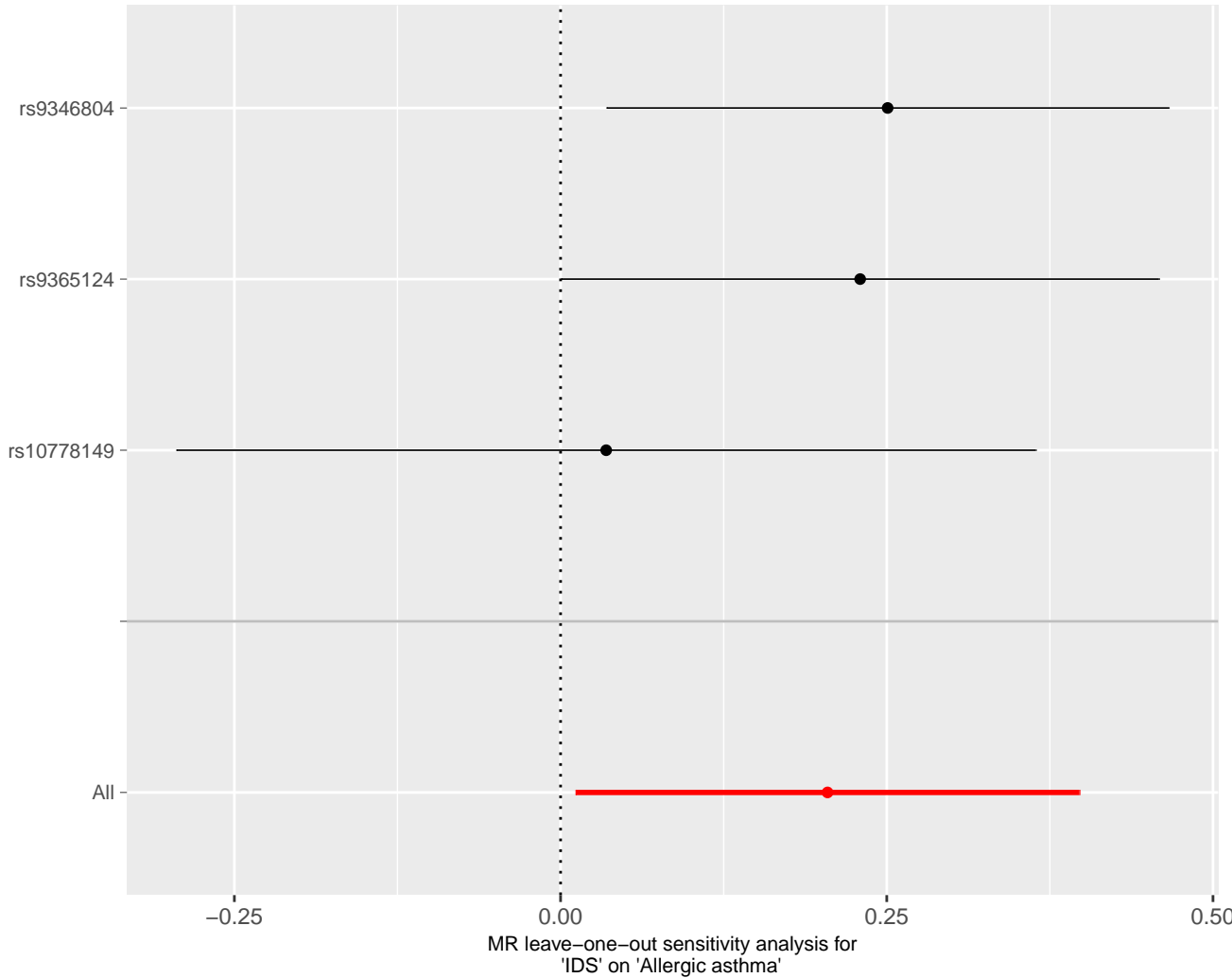

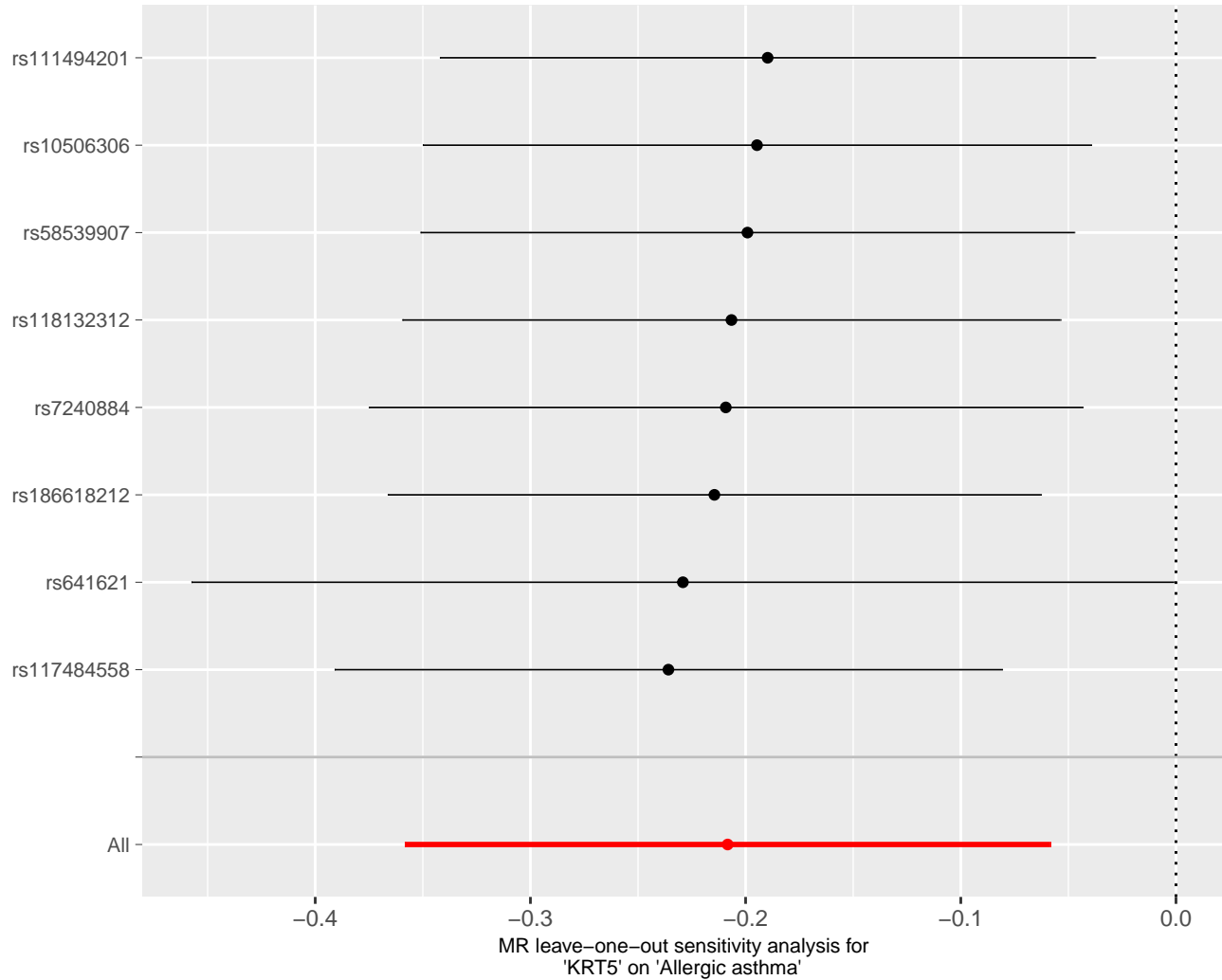

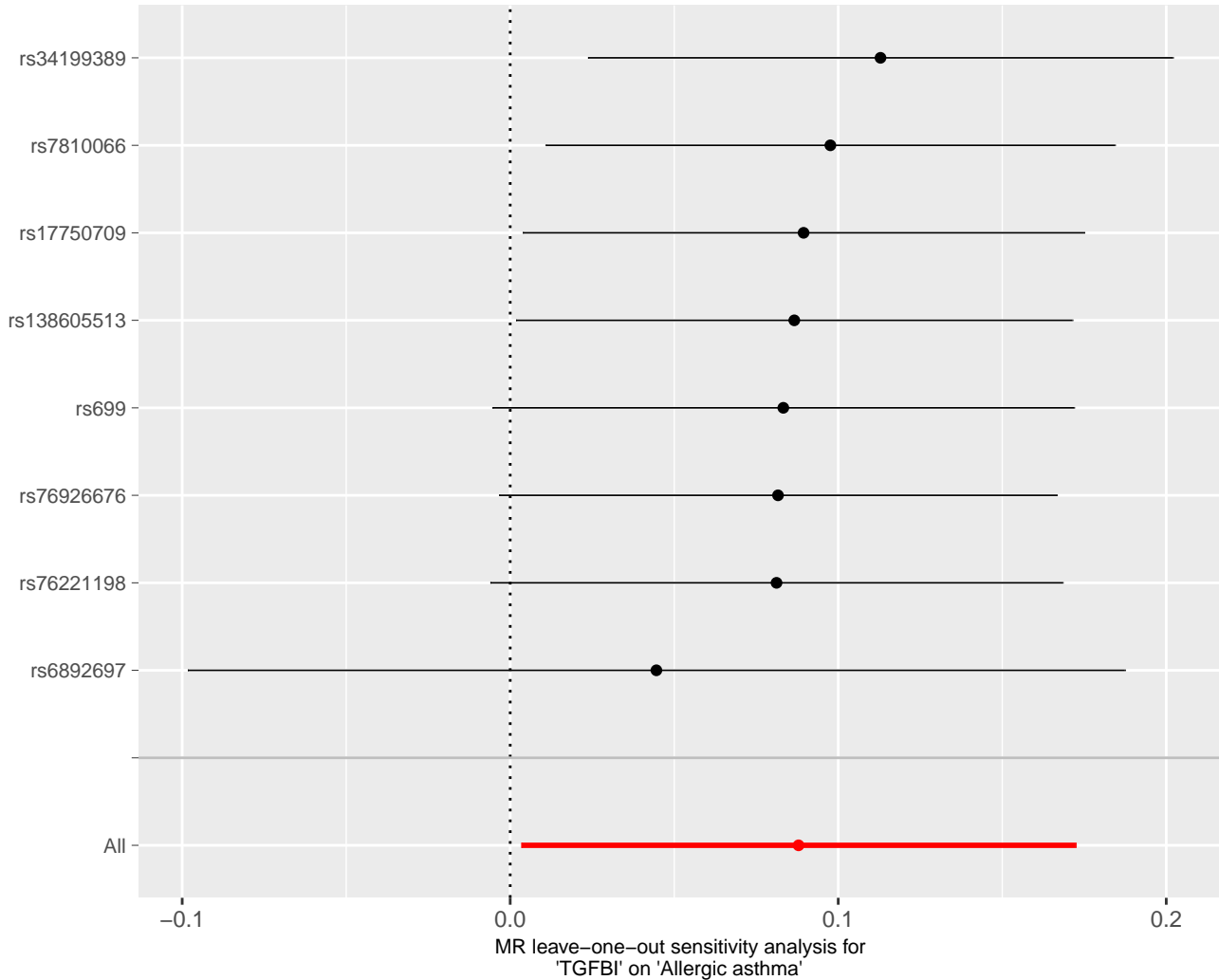

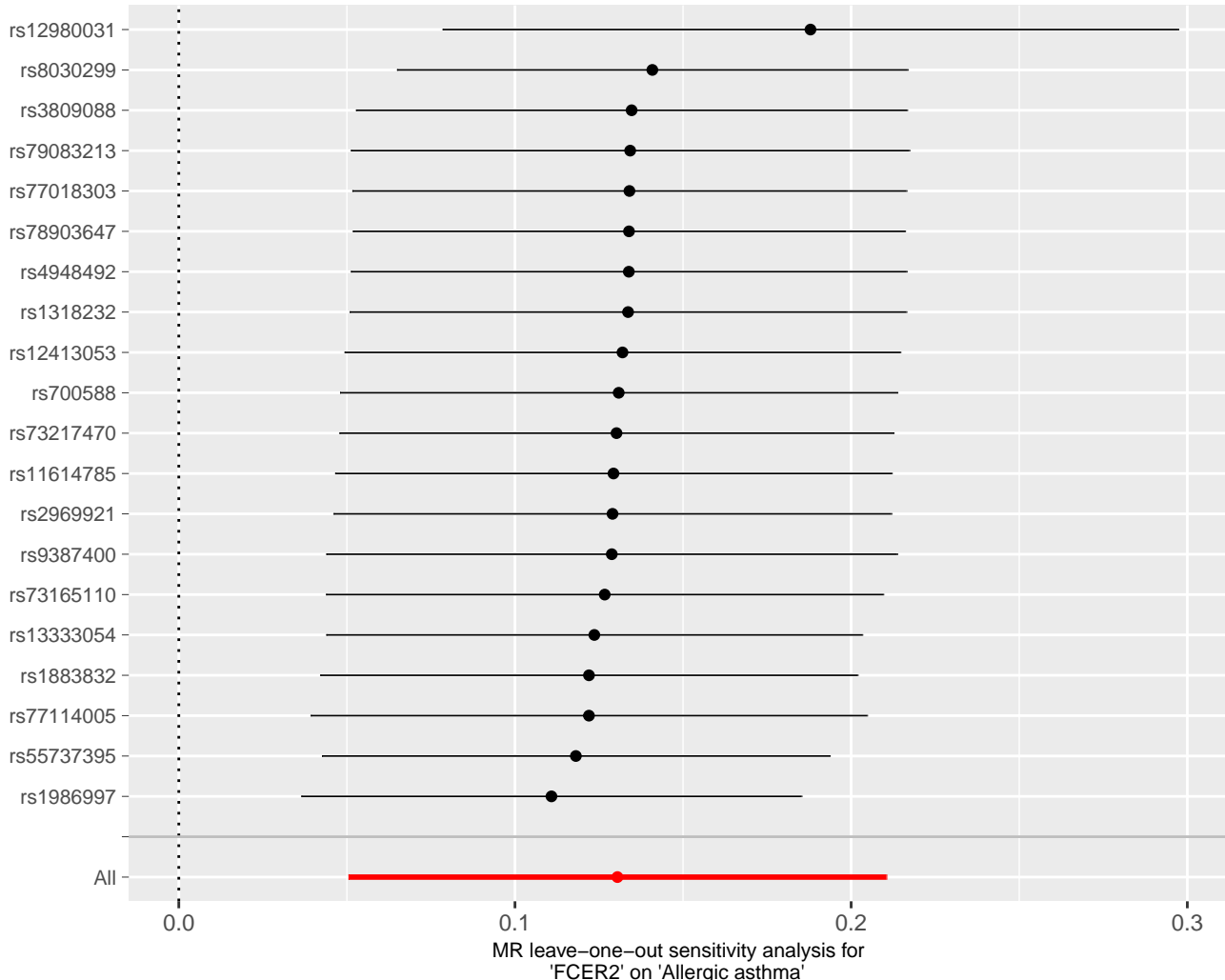

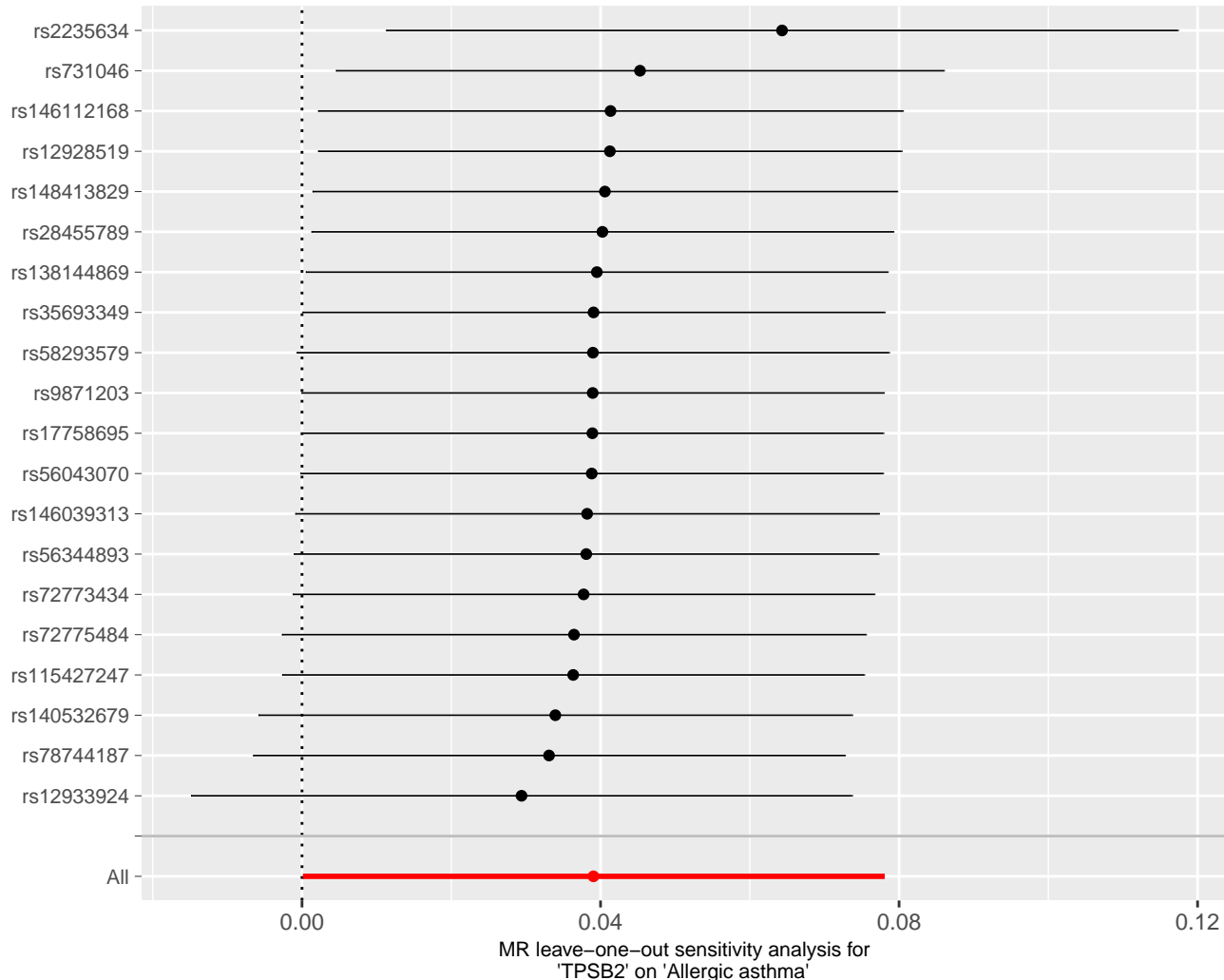

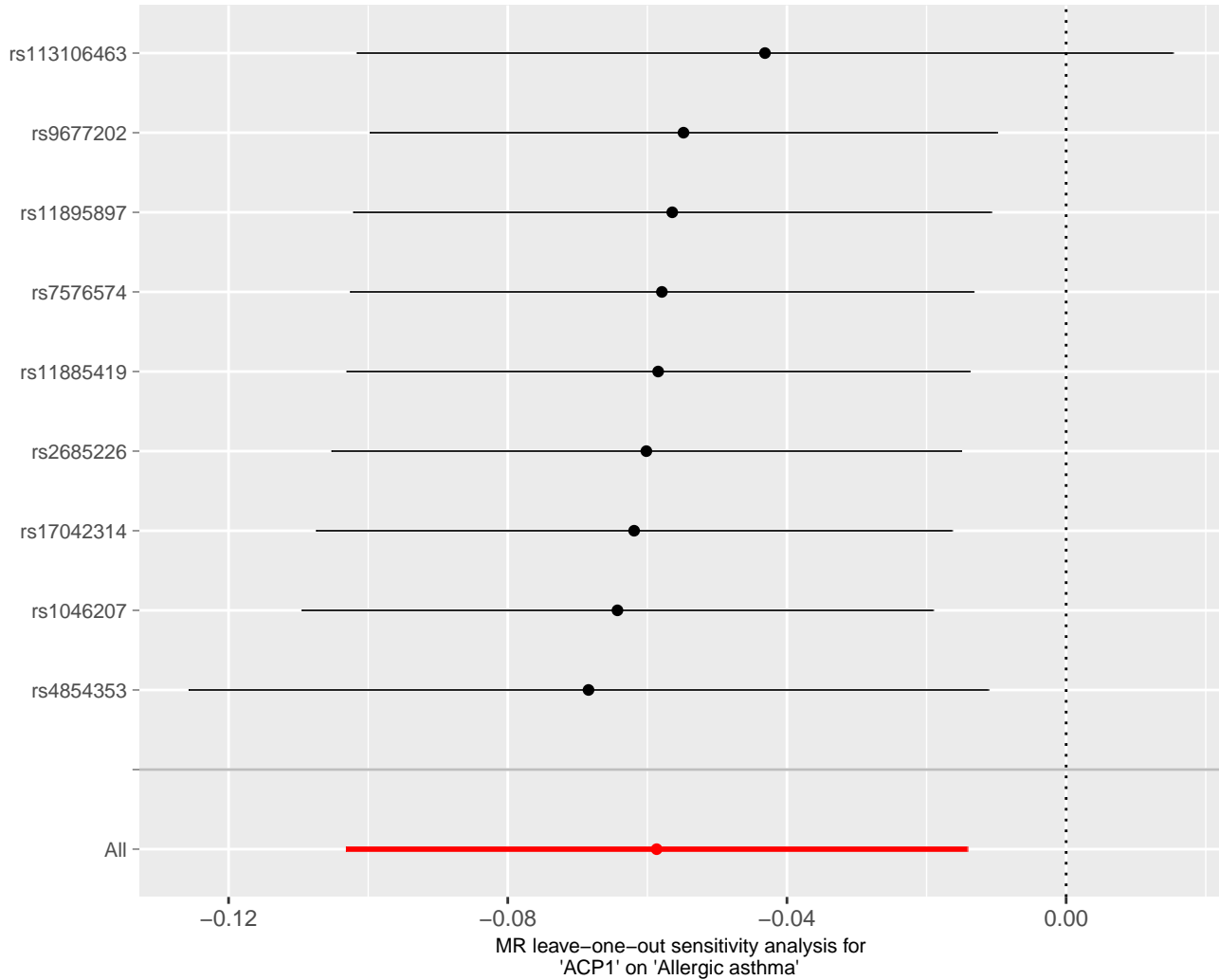

rs75475328

rs62395441

rs78564162

All

-0.6

-0.4

-0.2

0.0

MR leave-one-out sensitivity analysis for  
'GZMB' on 'Allergic asthma'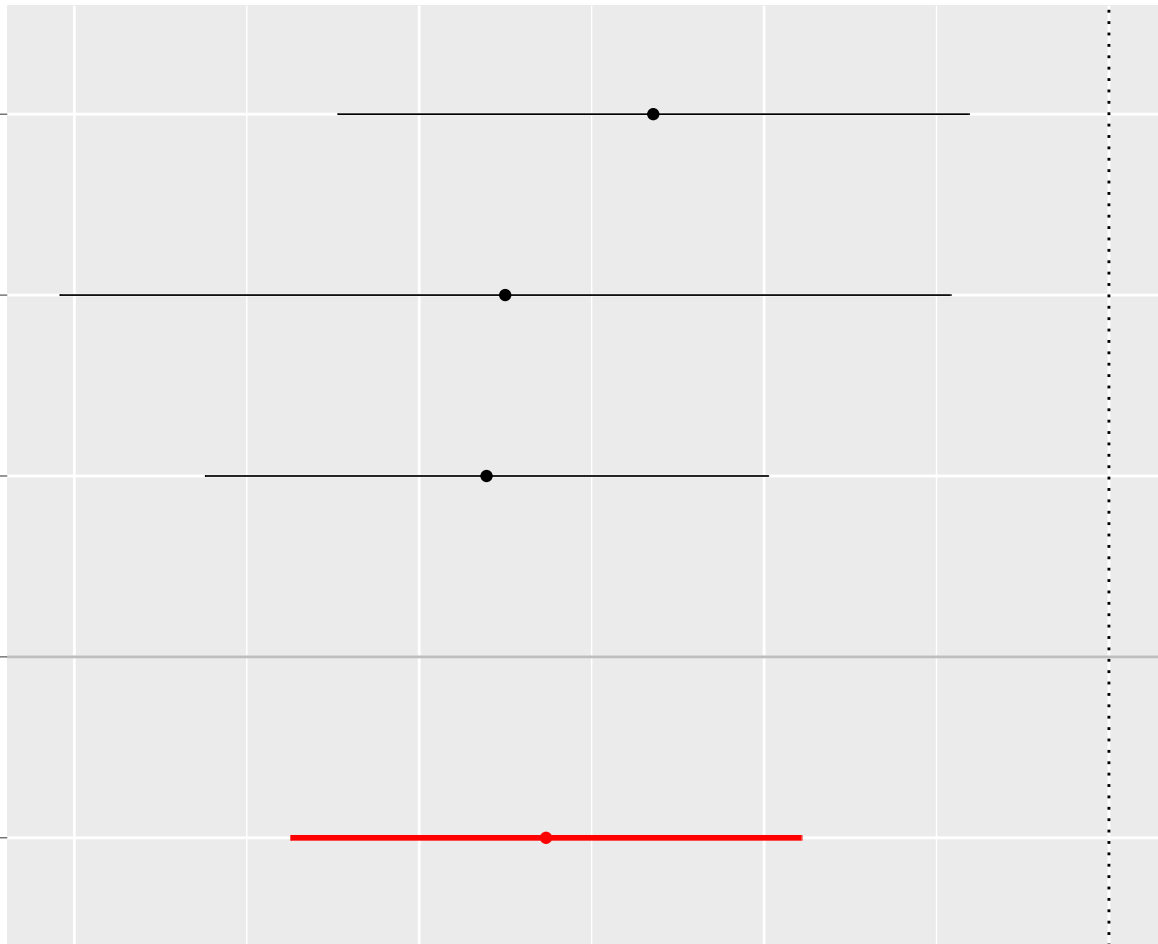

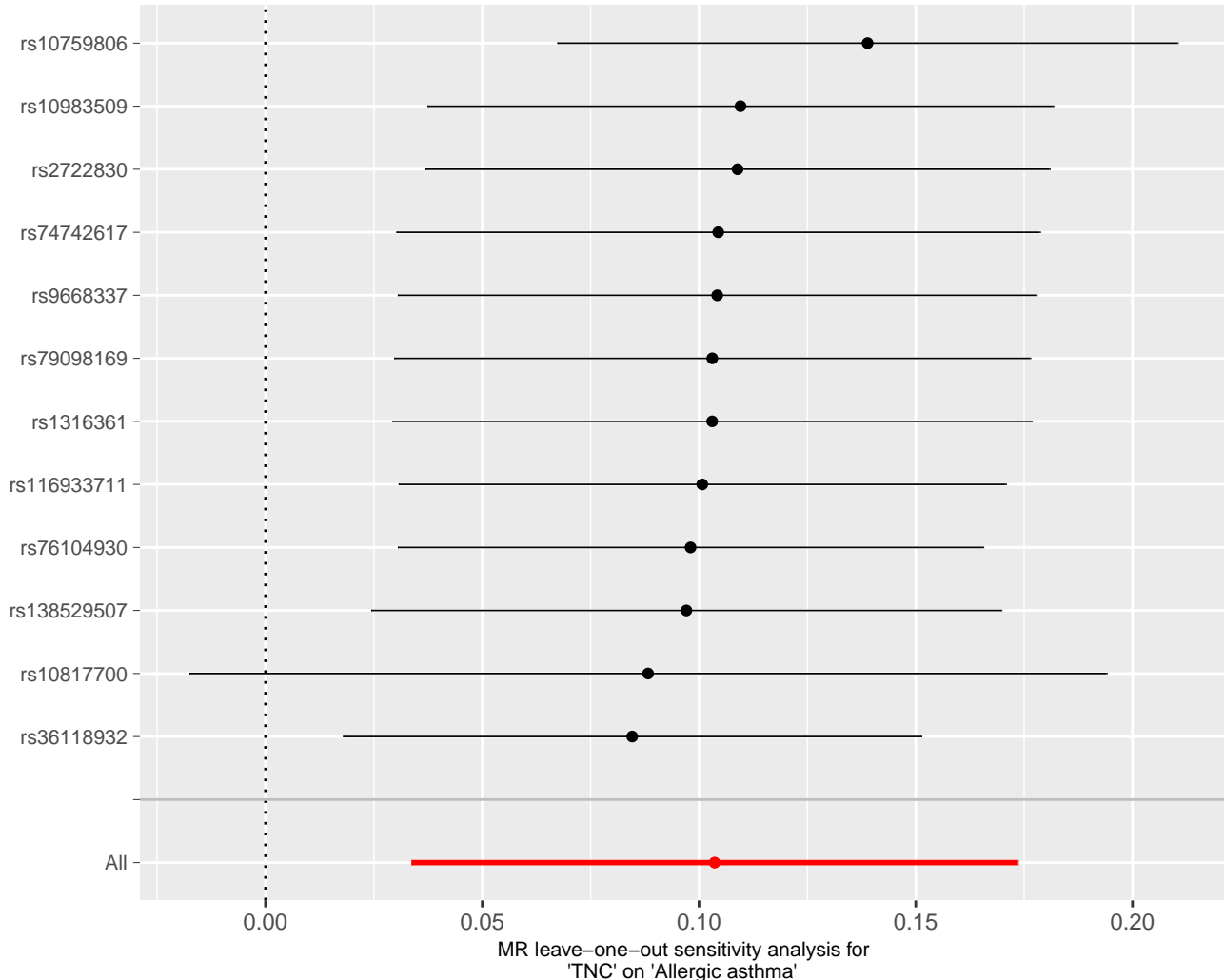

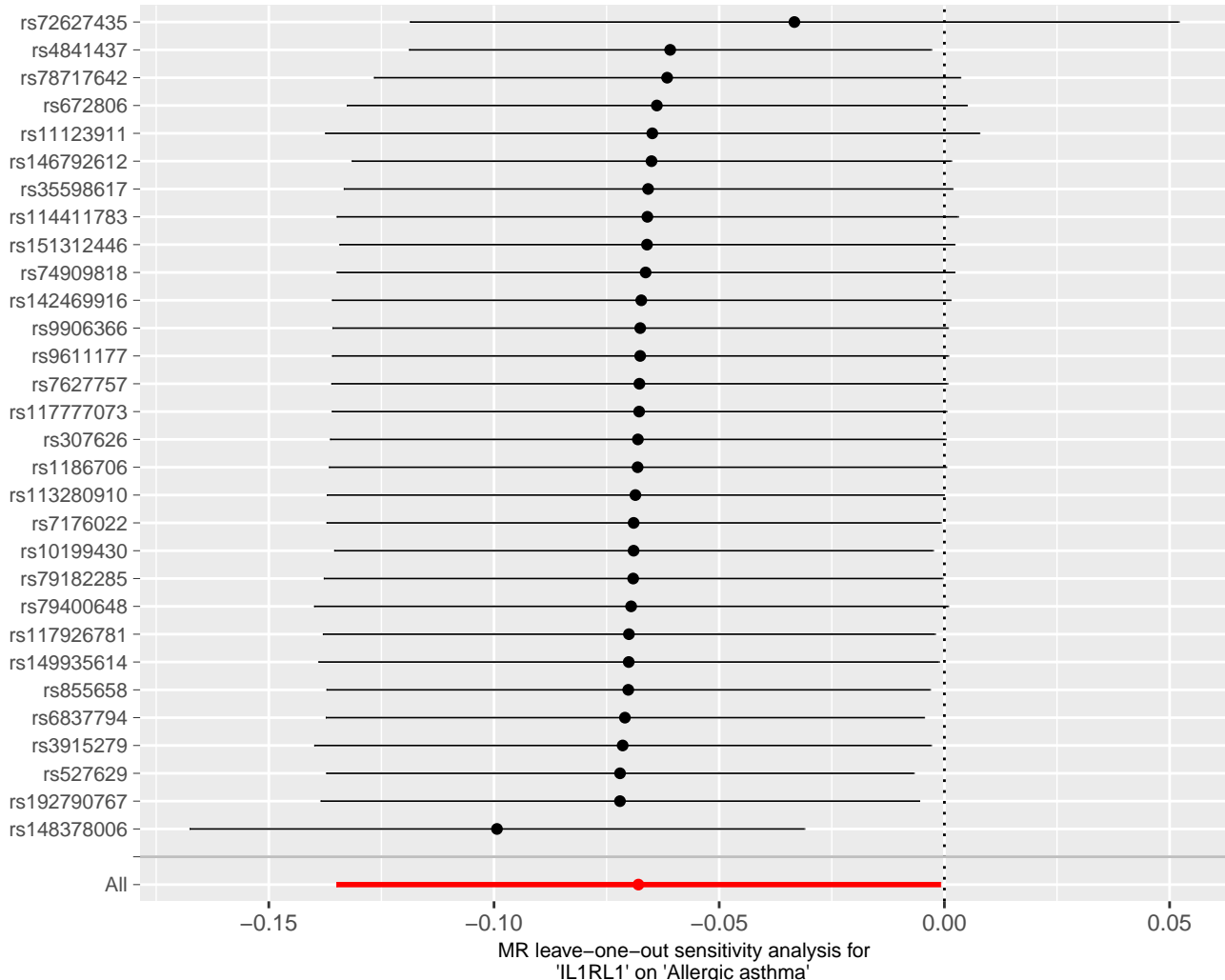

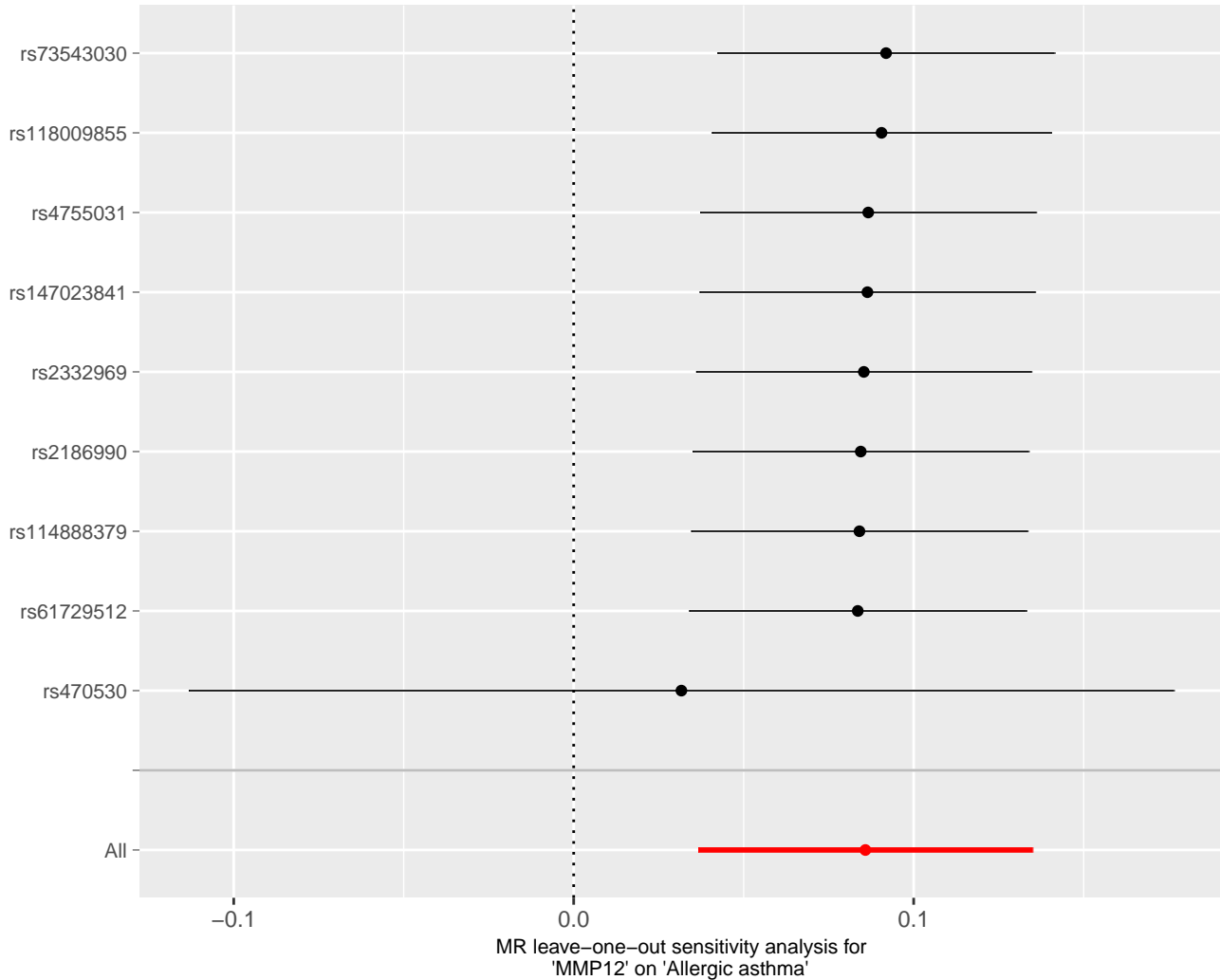

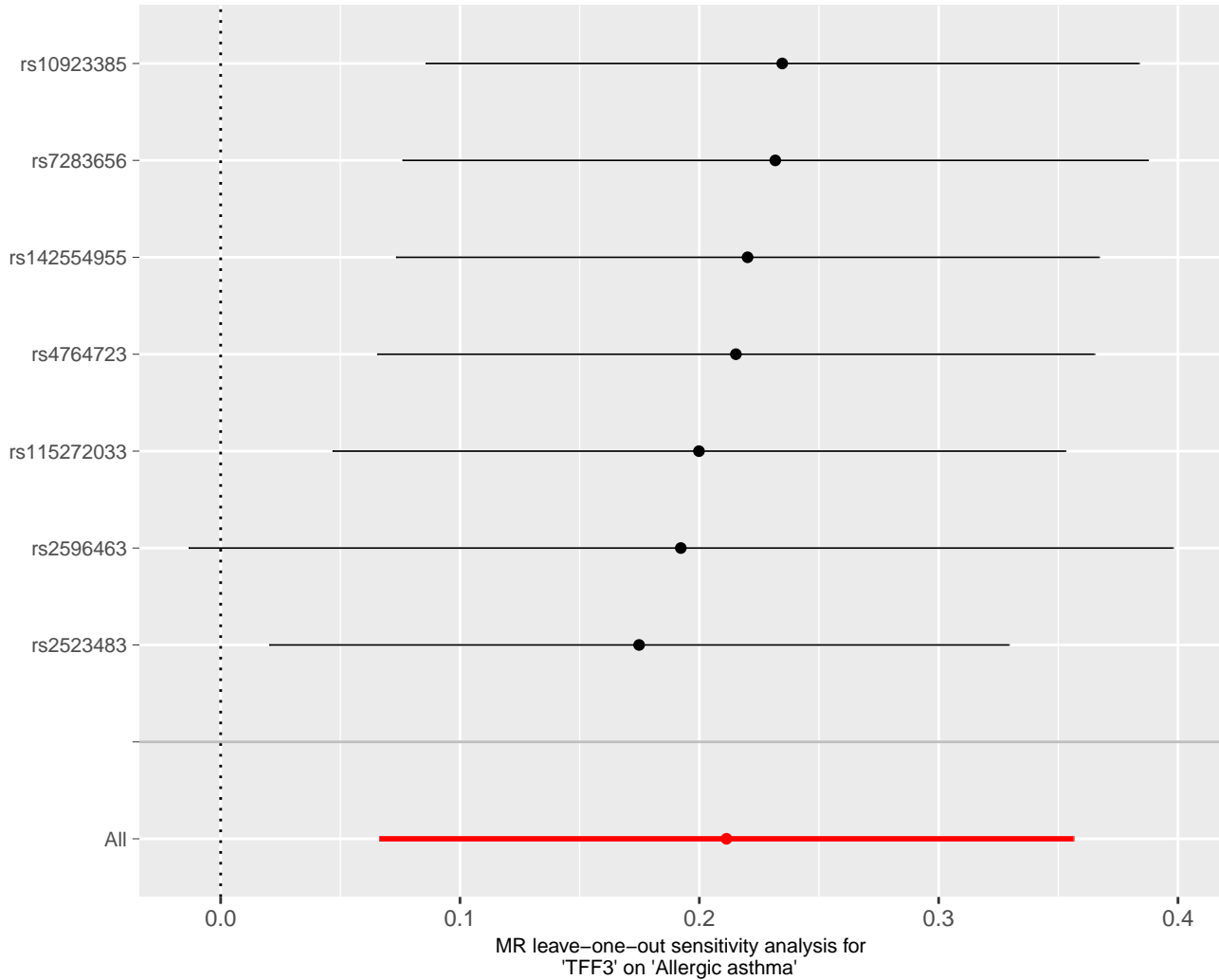

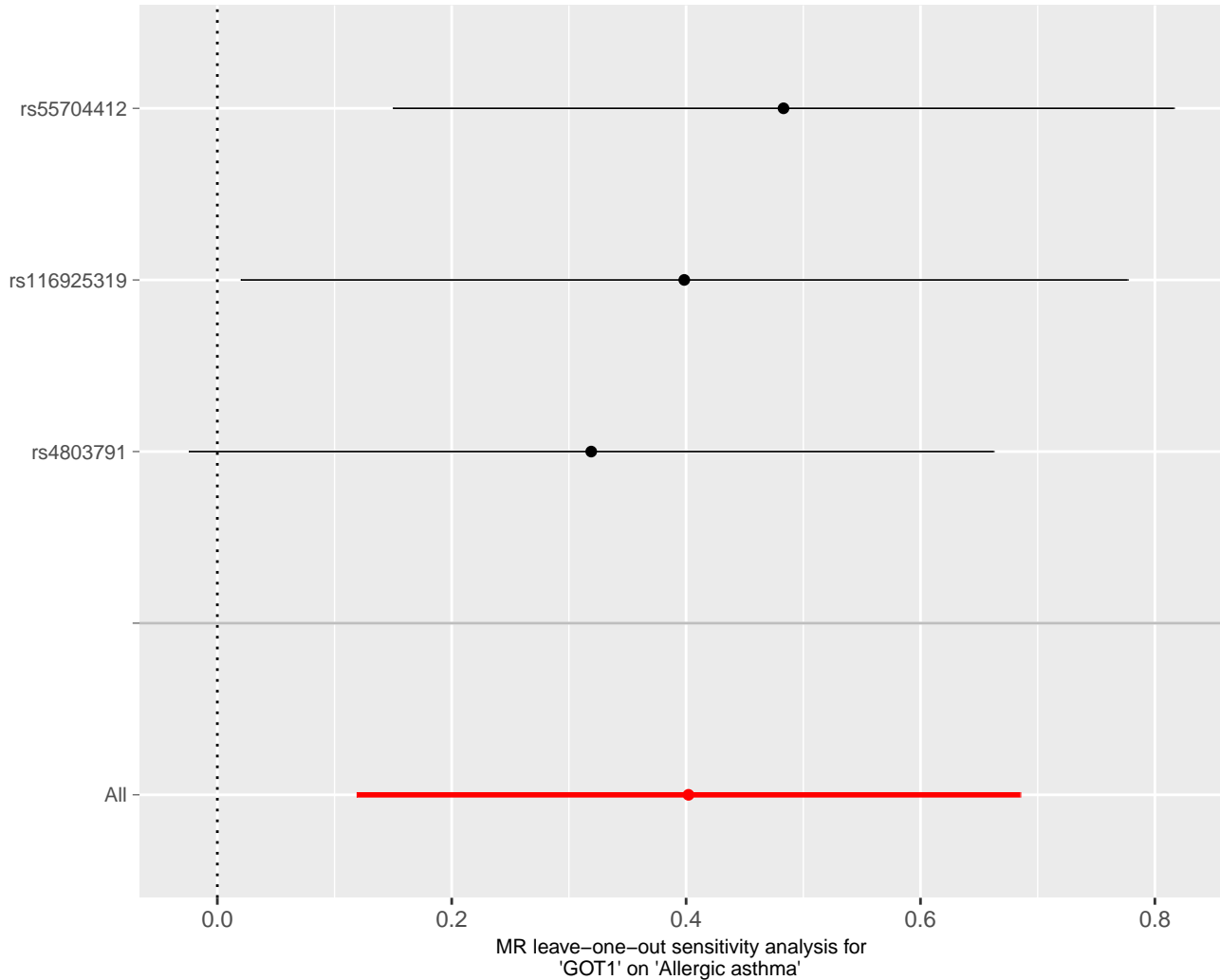

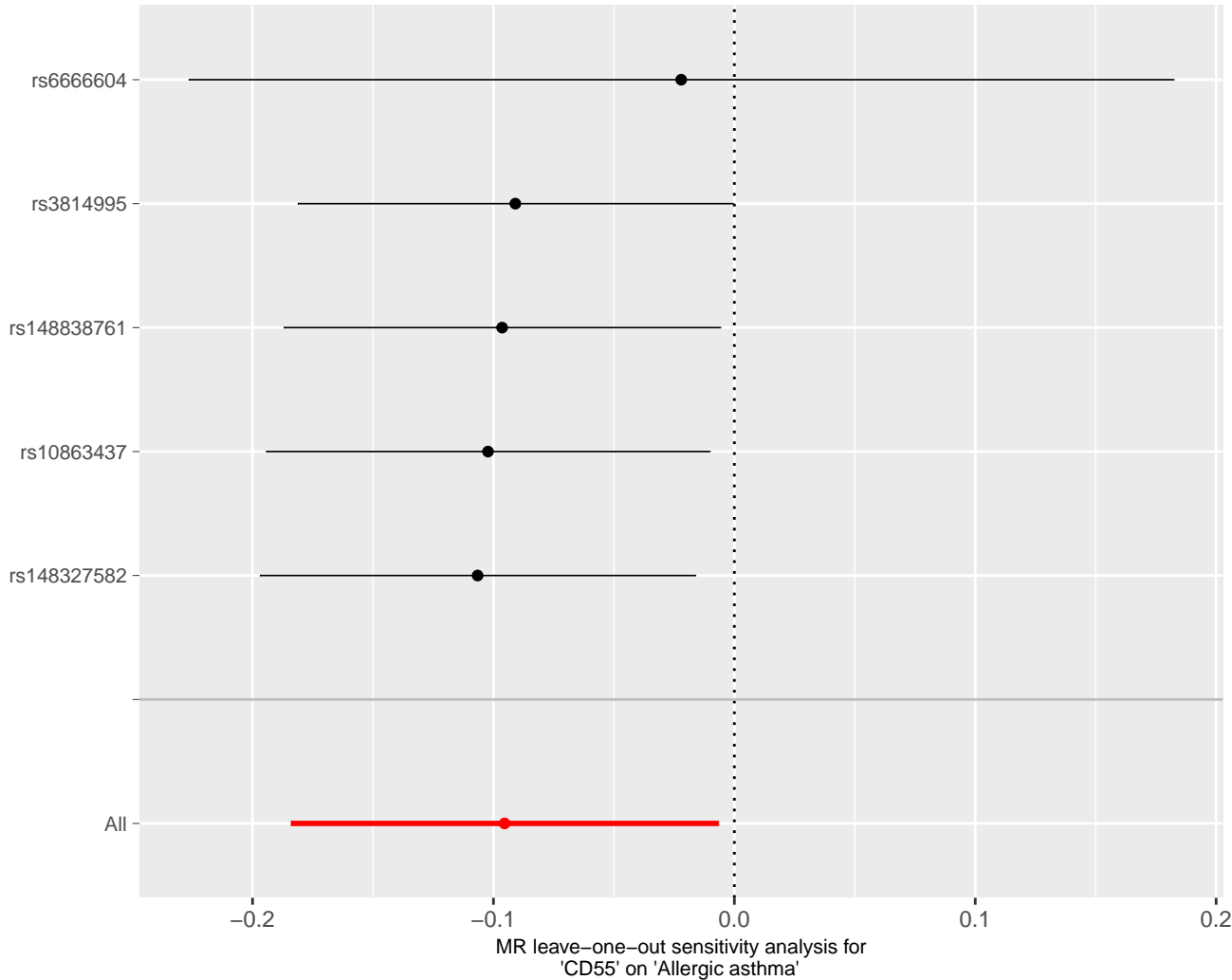

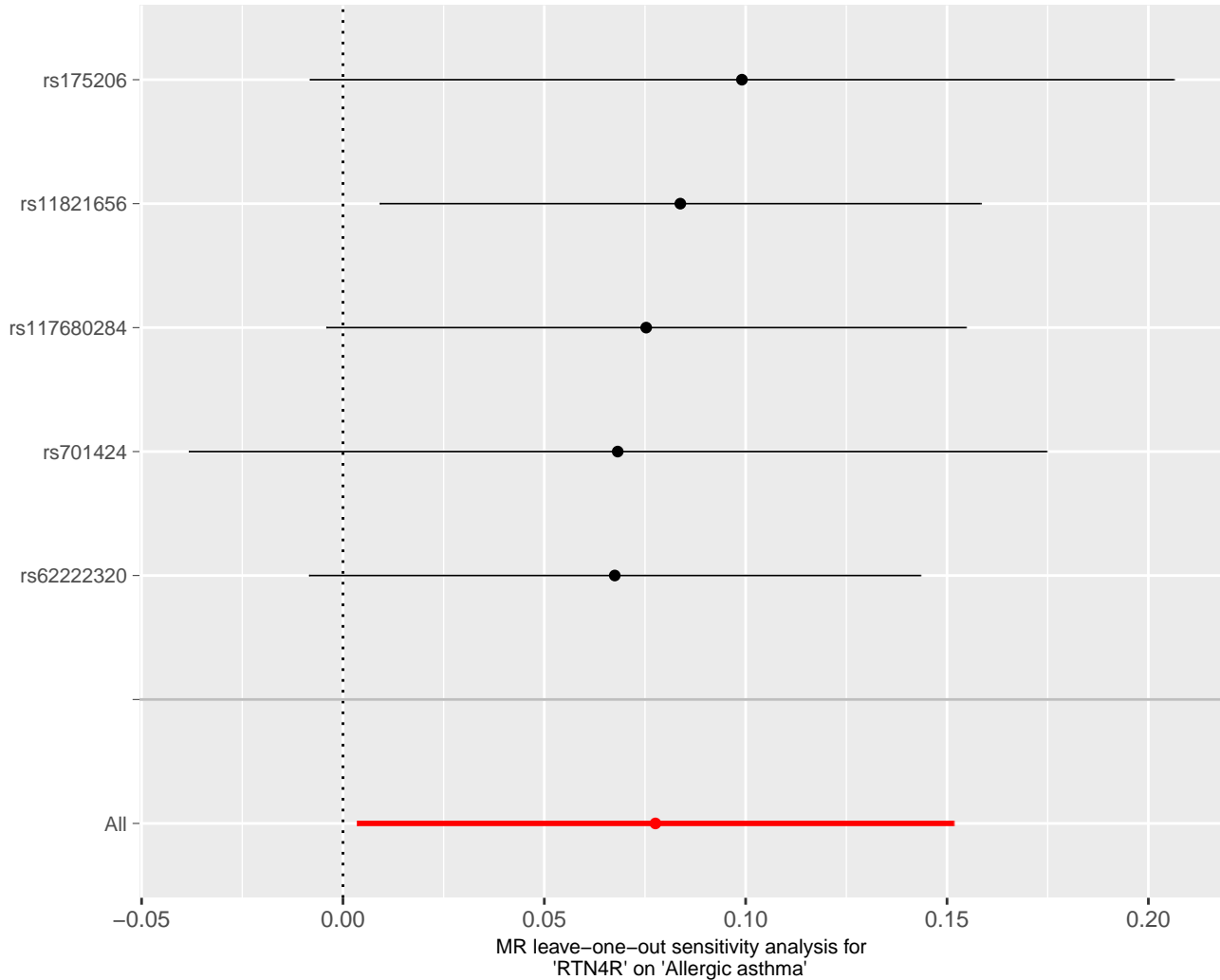

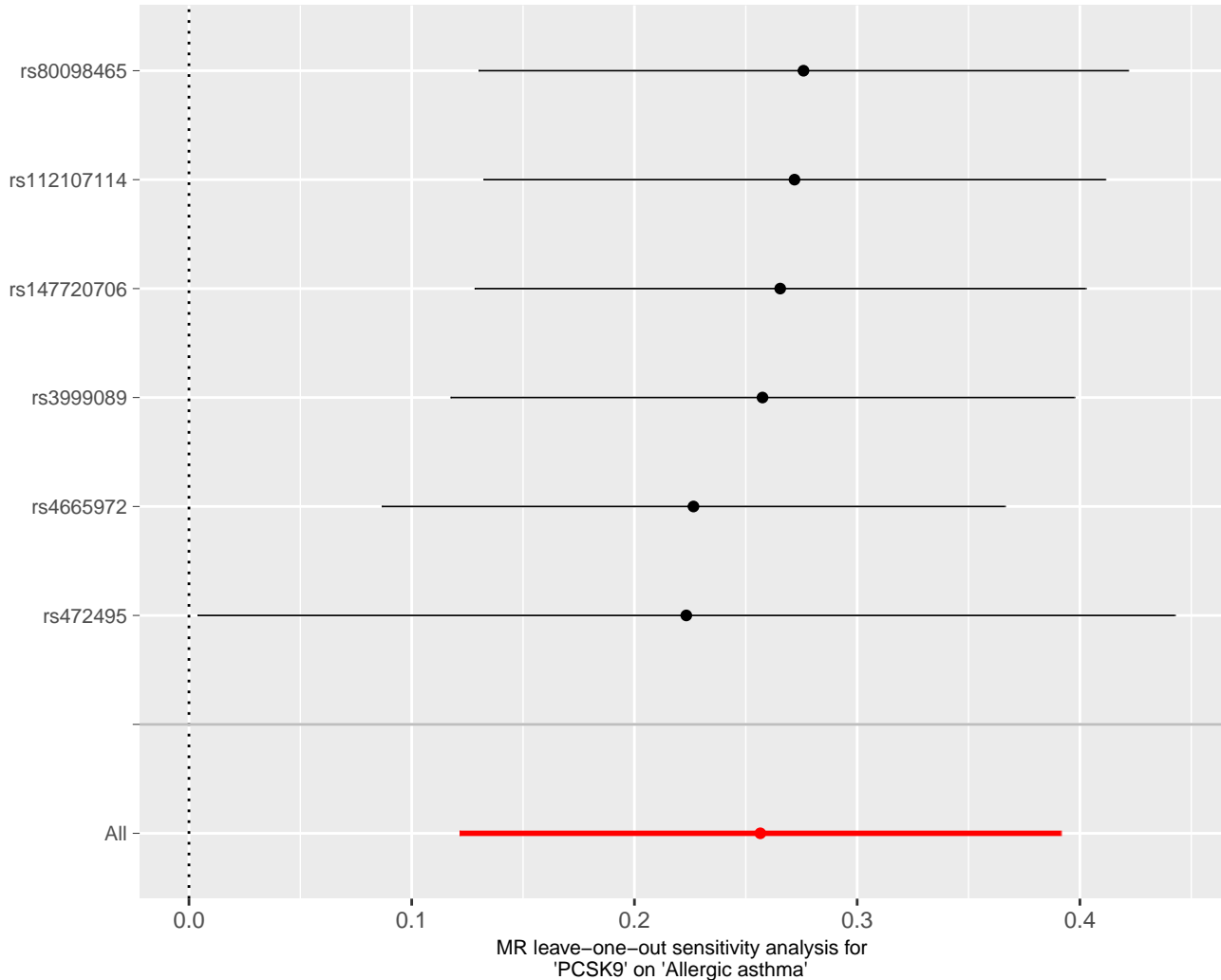

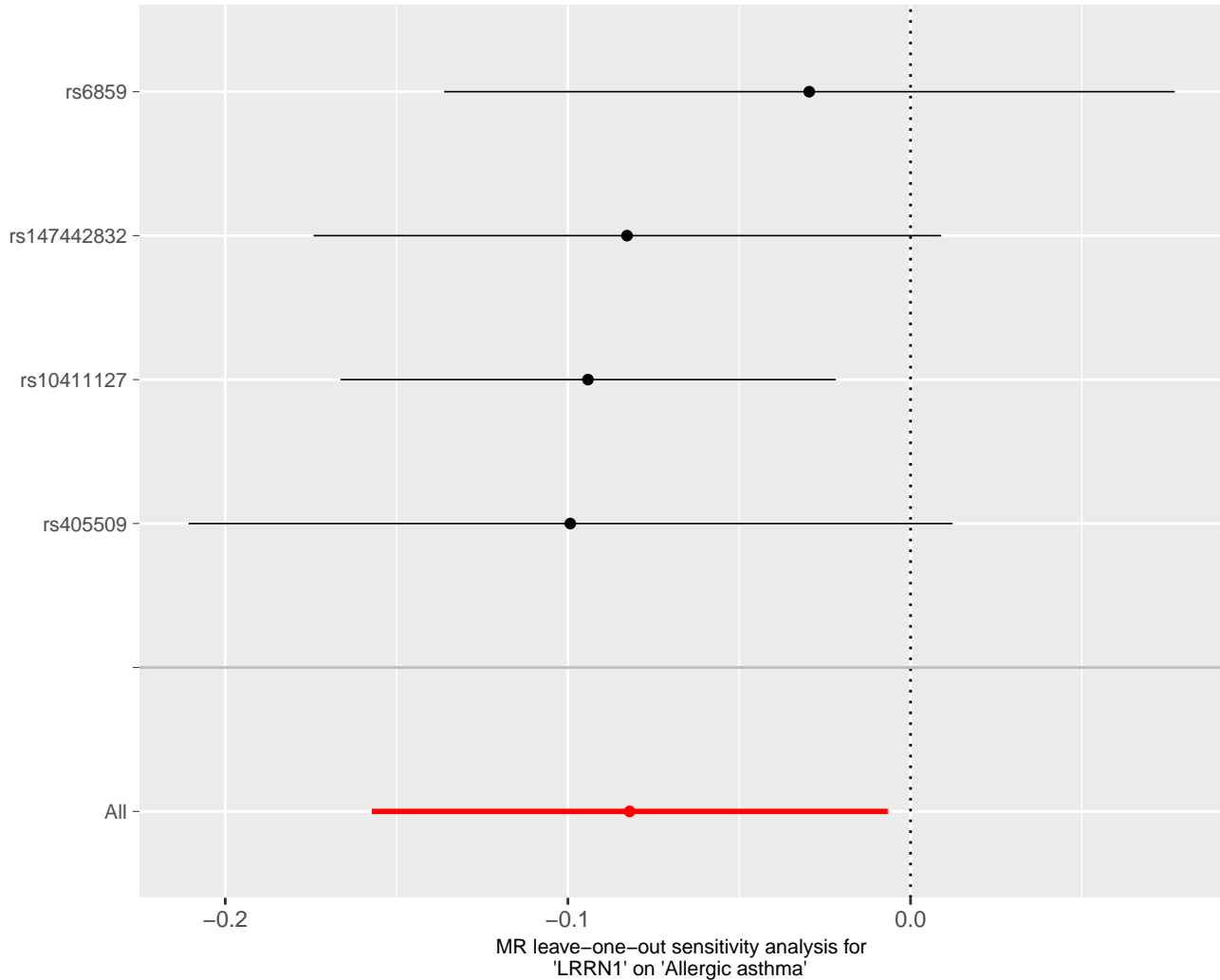

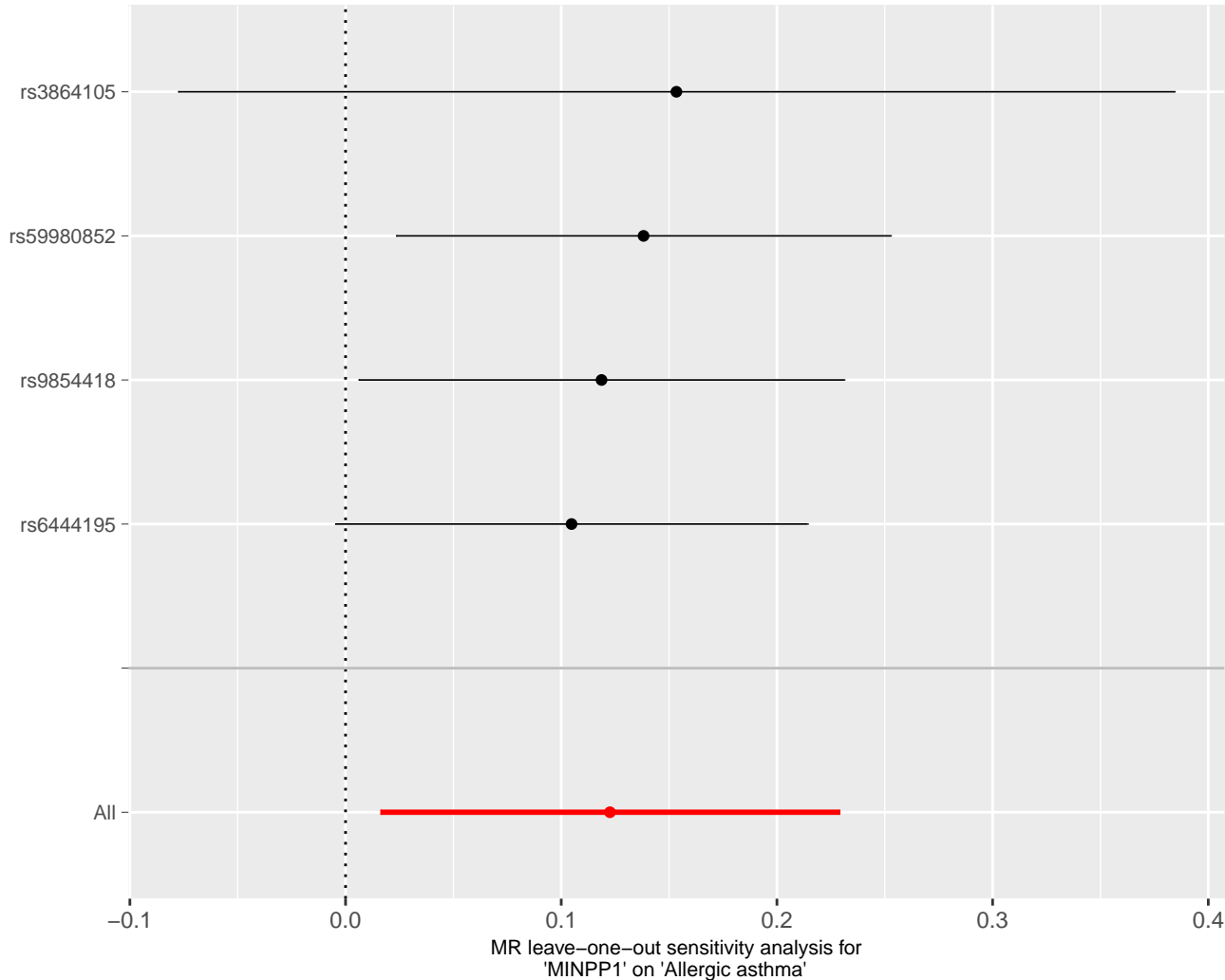

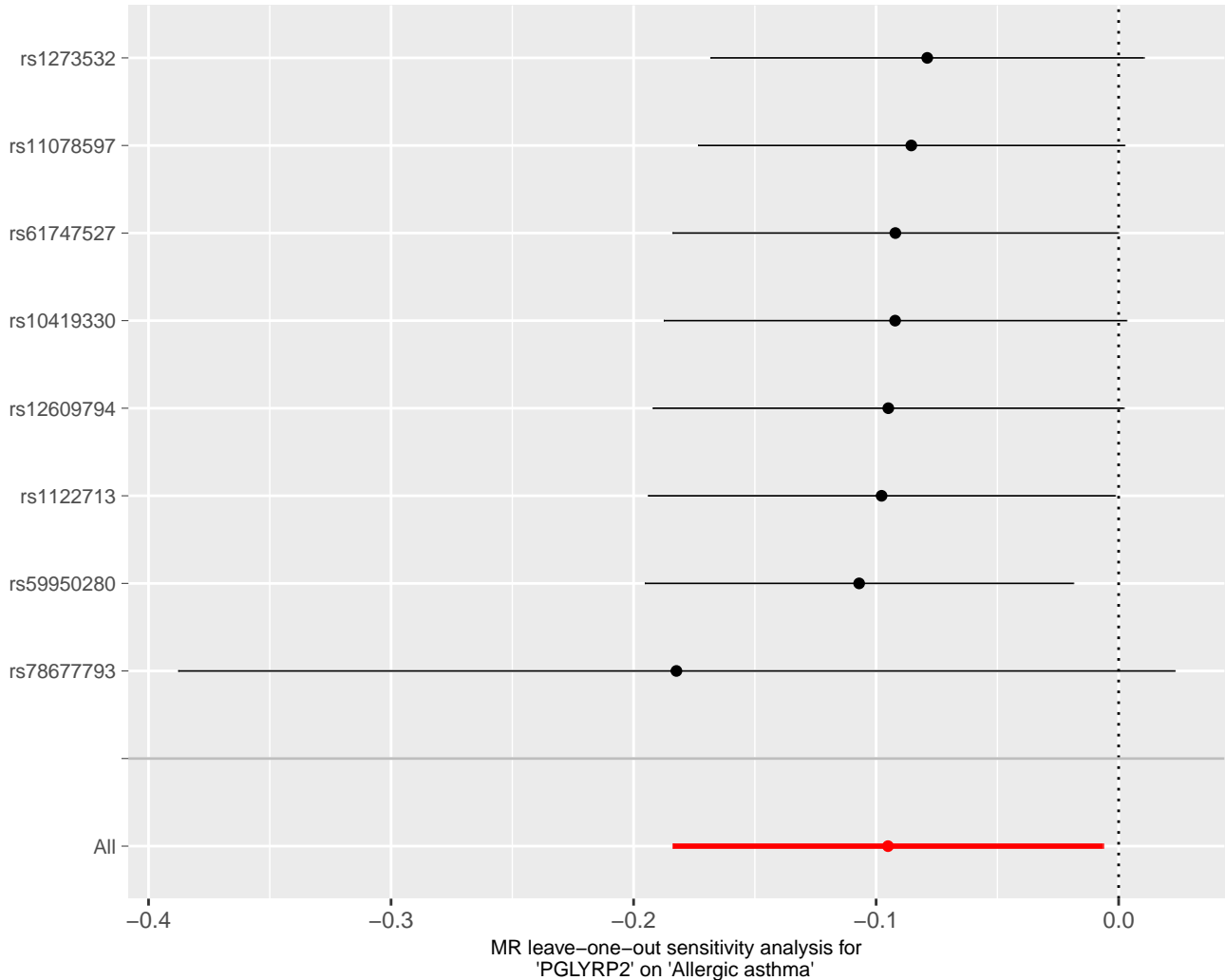

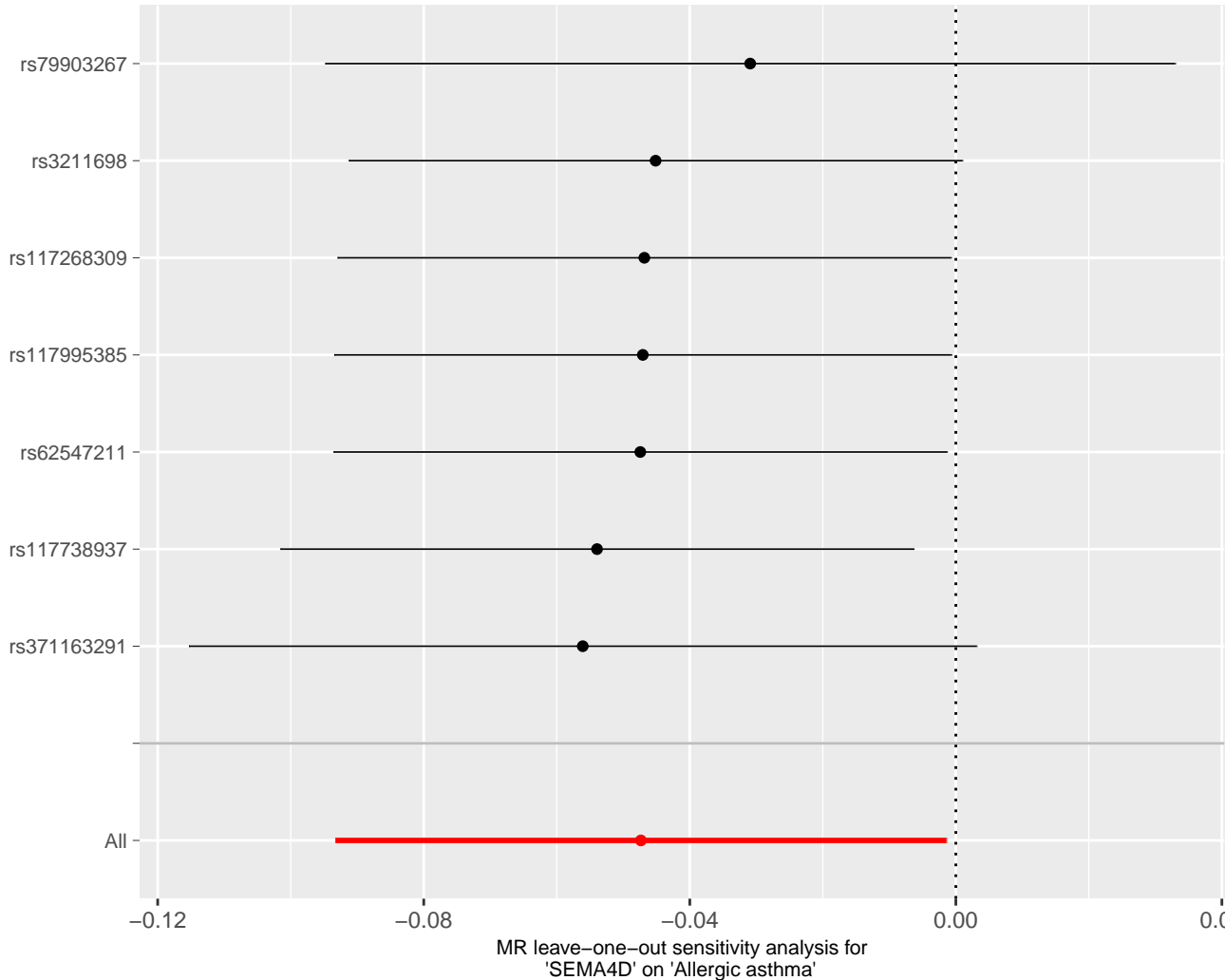

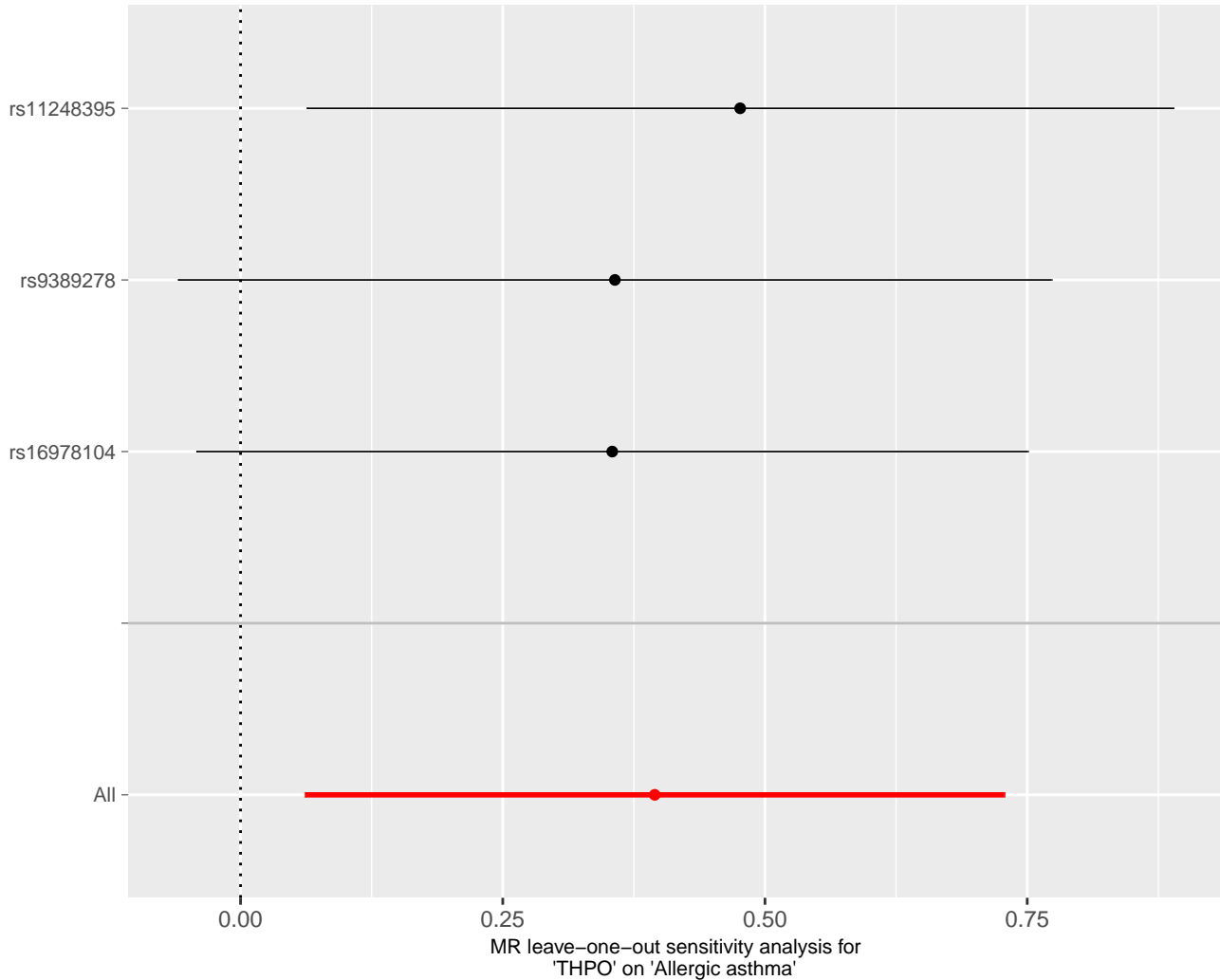

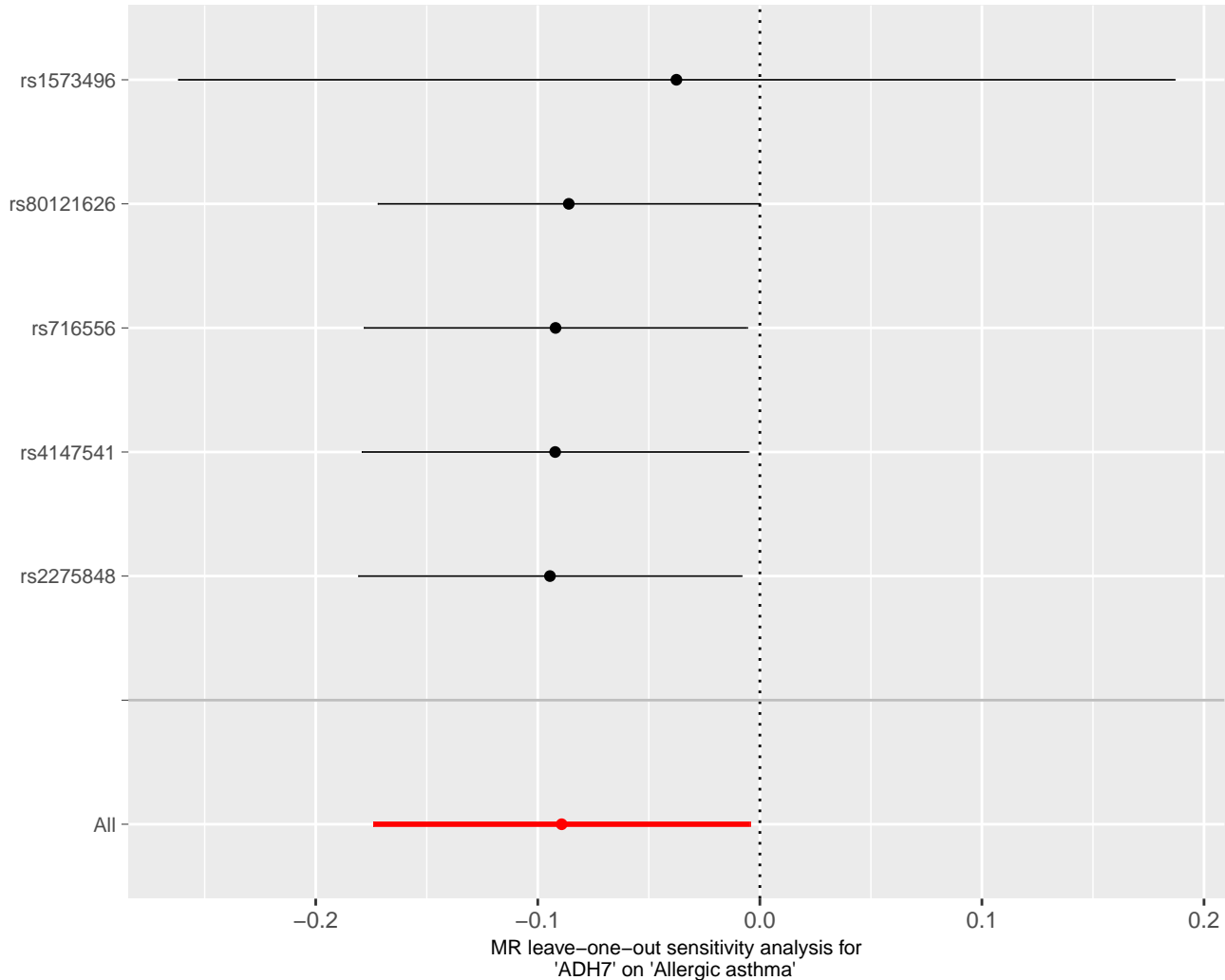

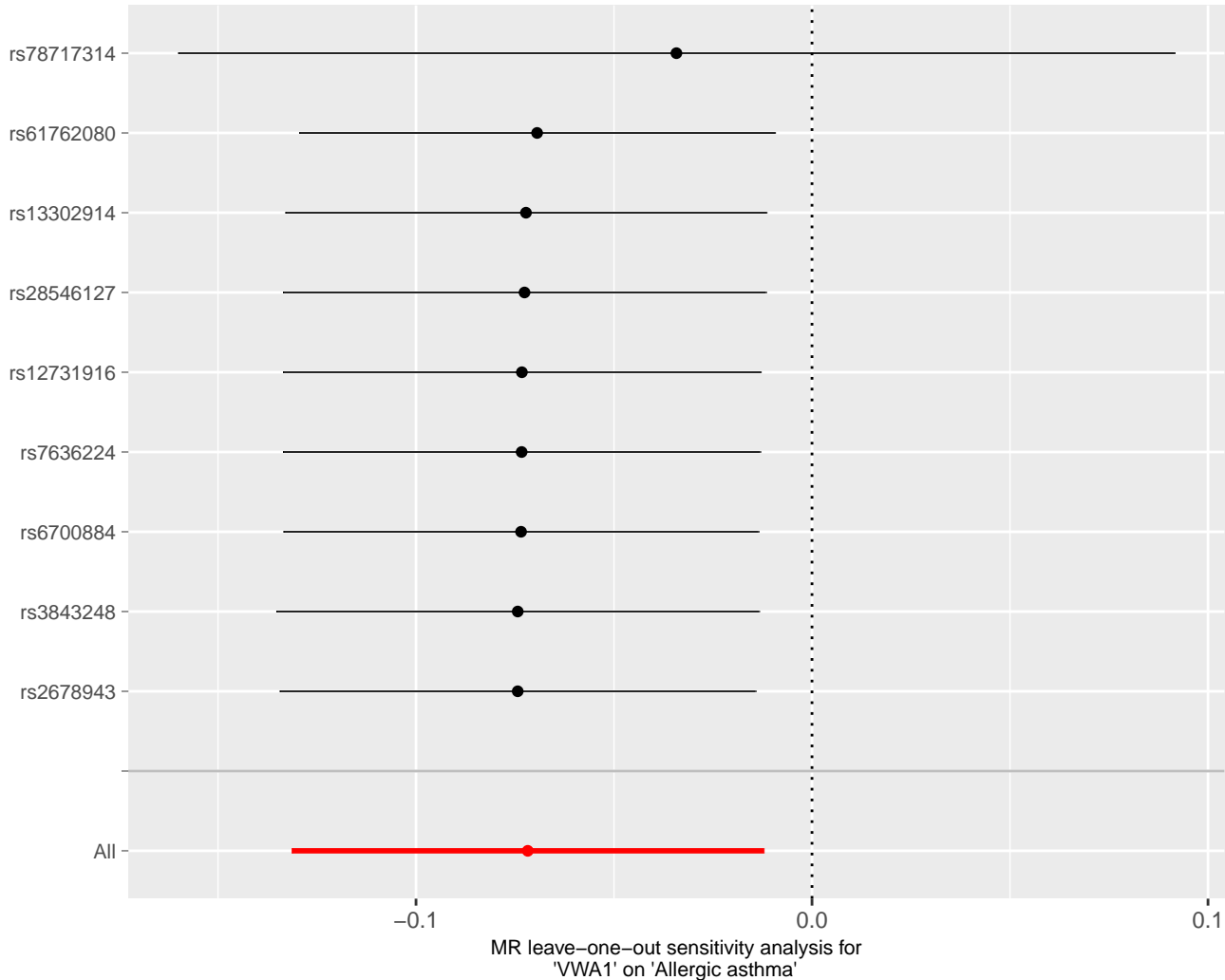

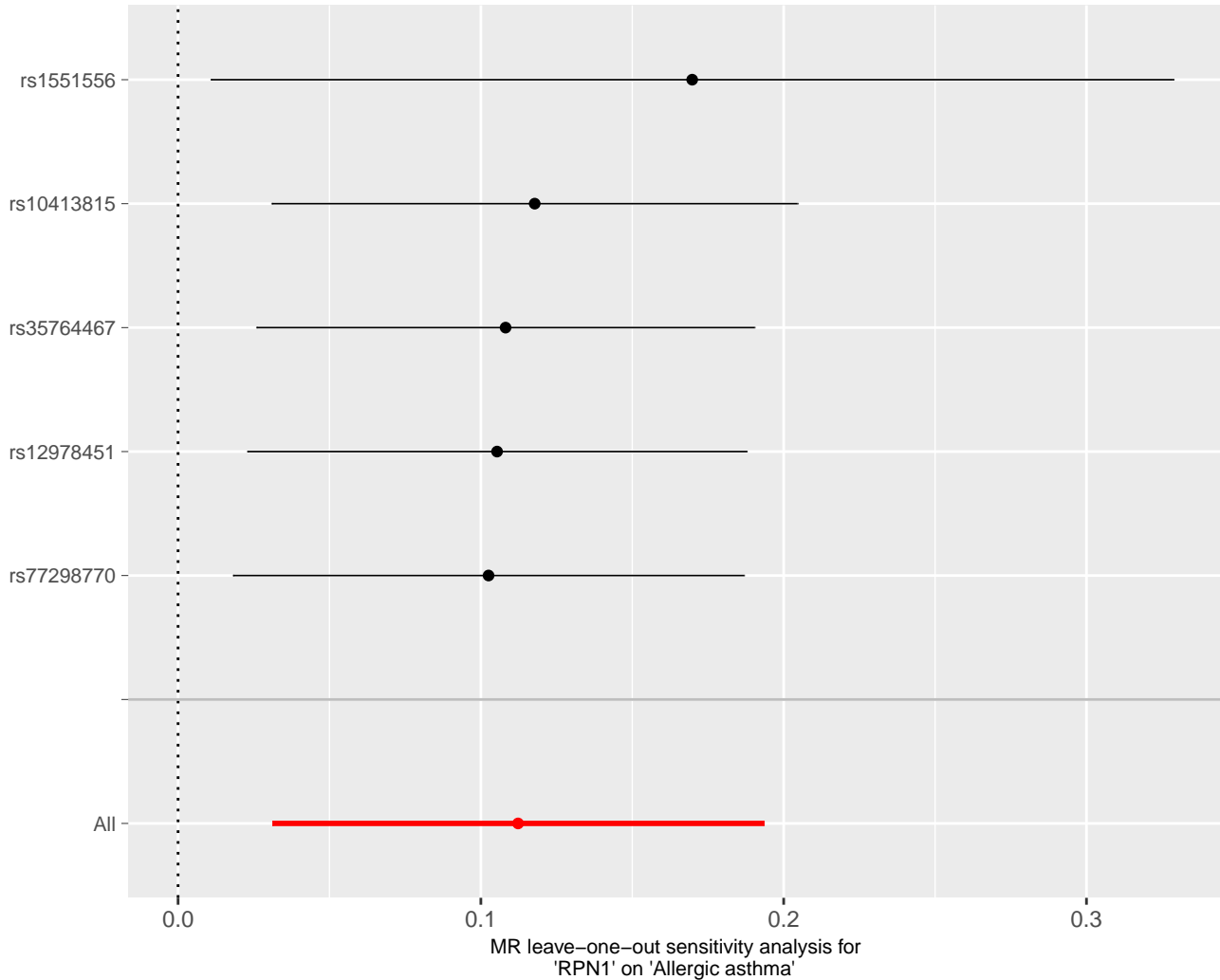

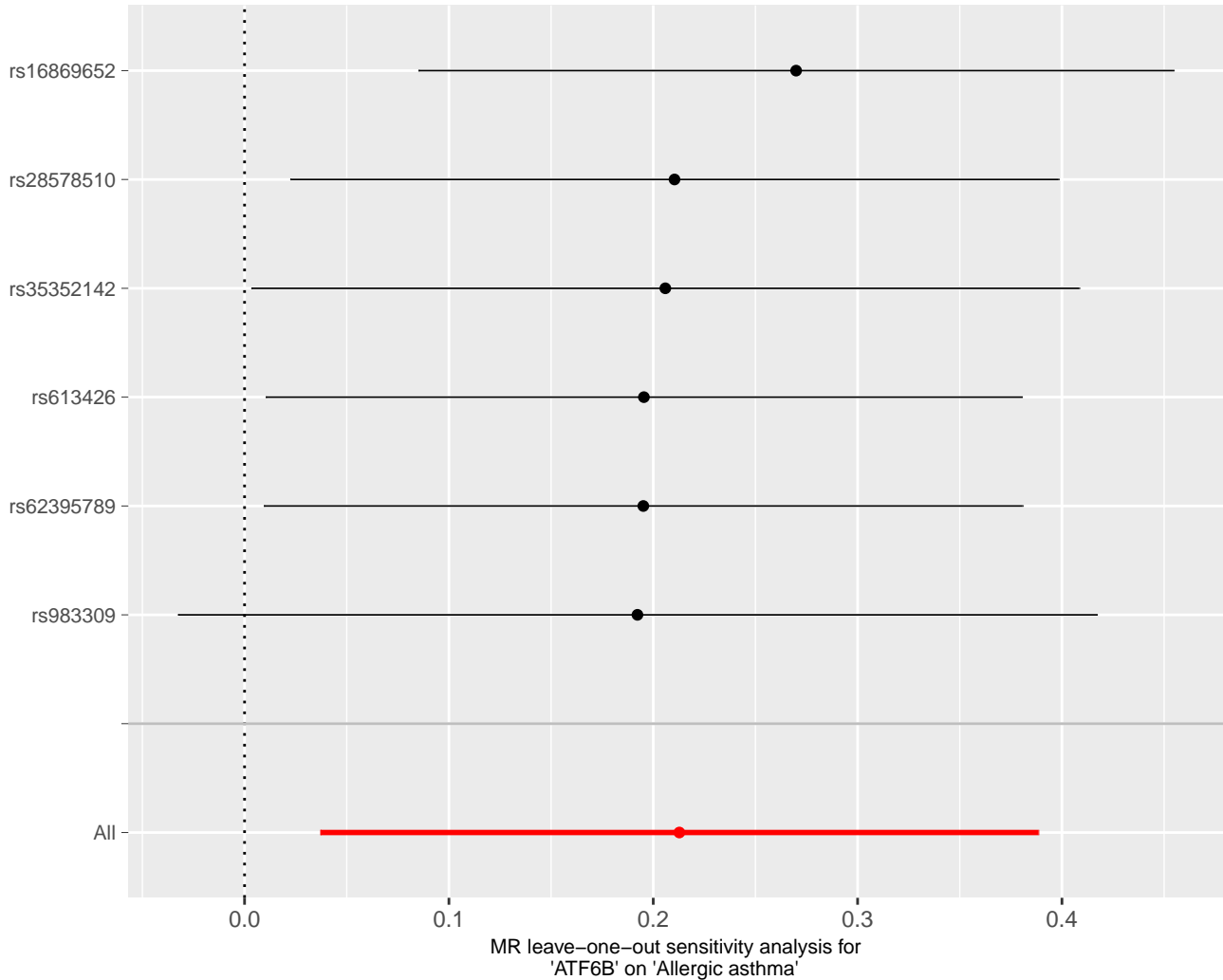

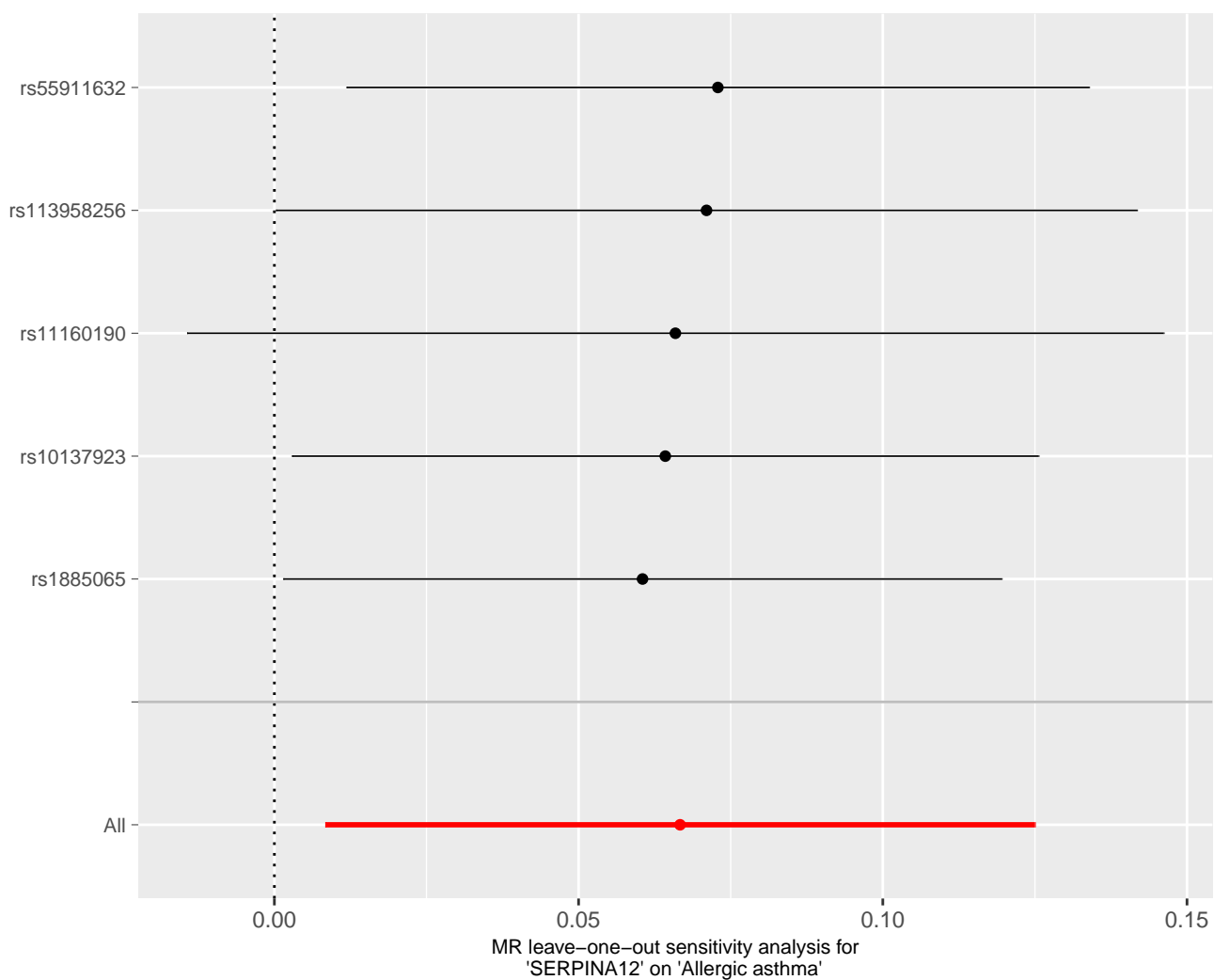

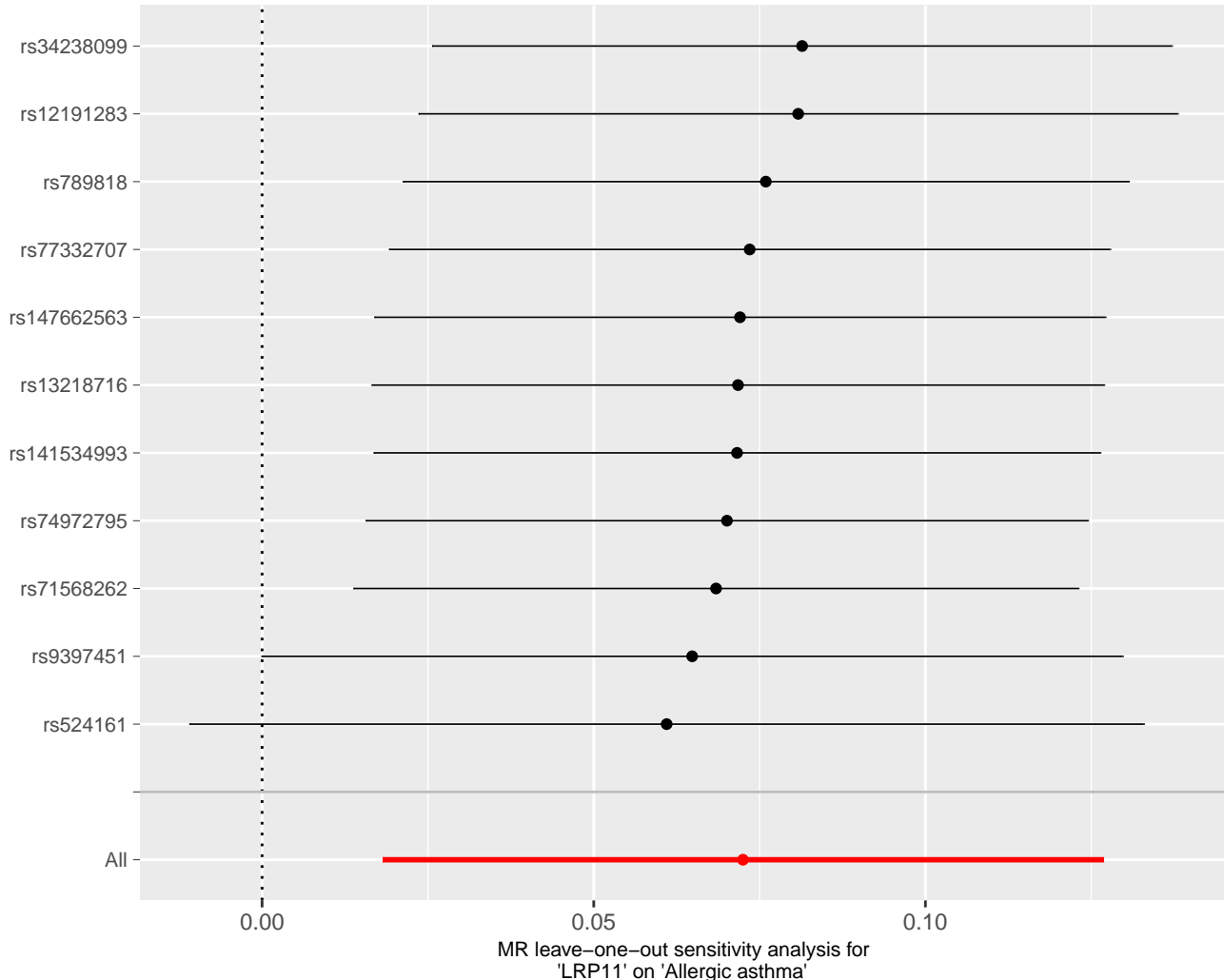

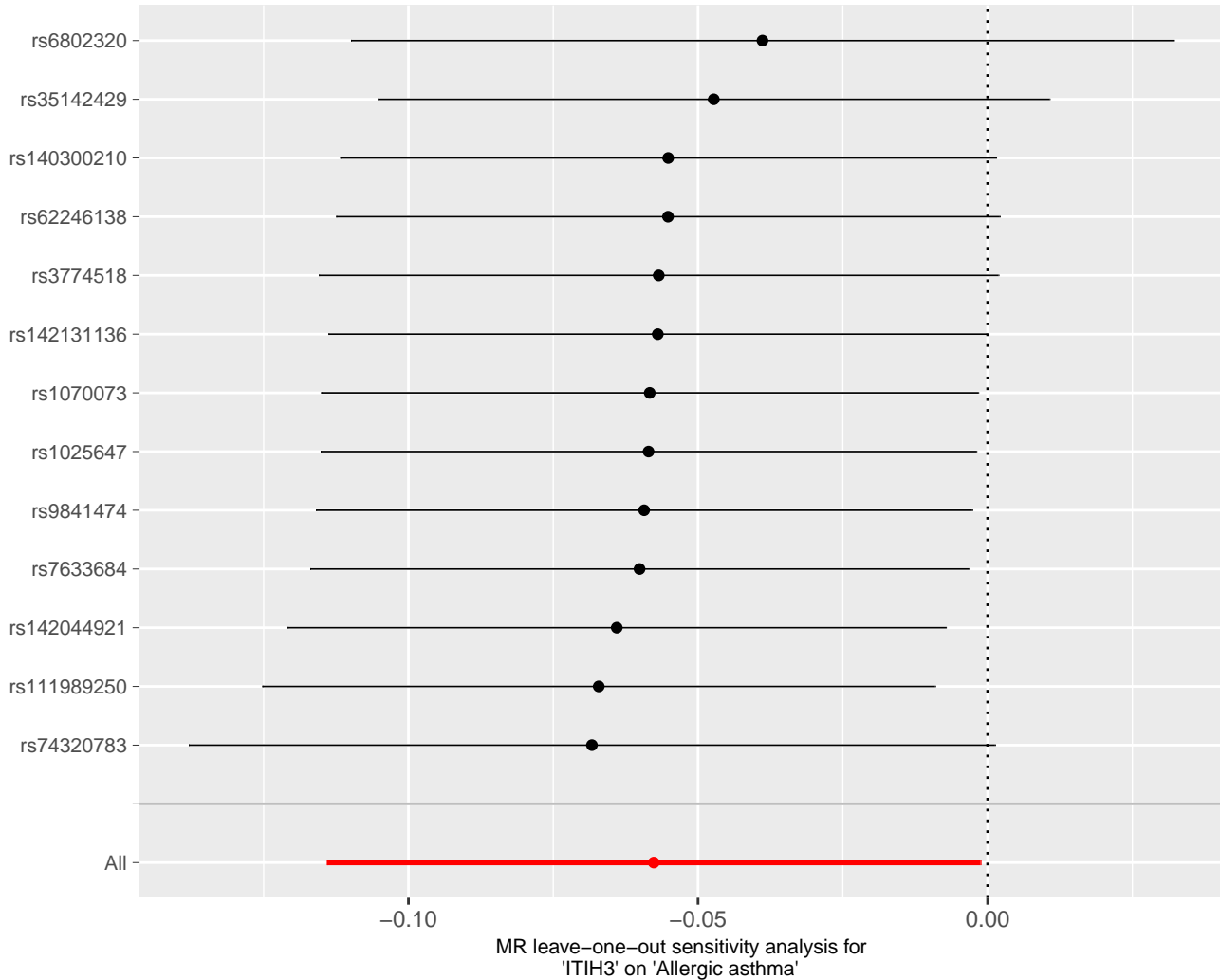

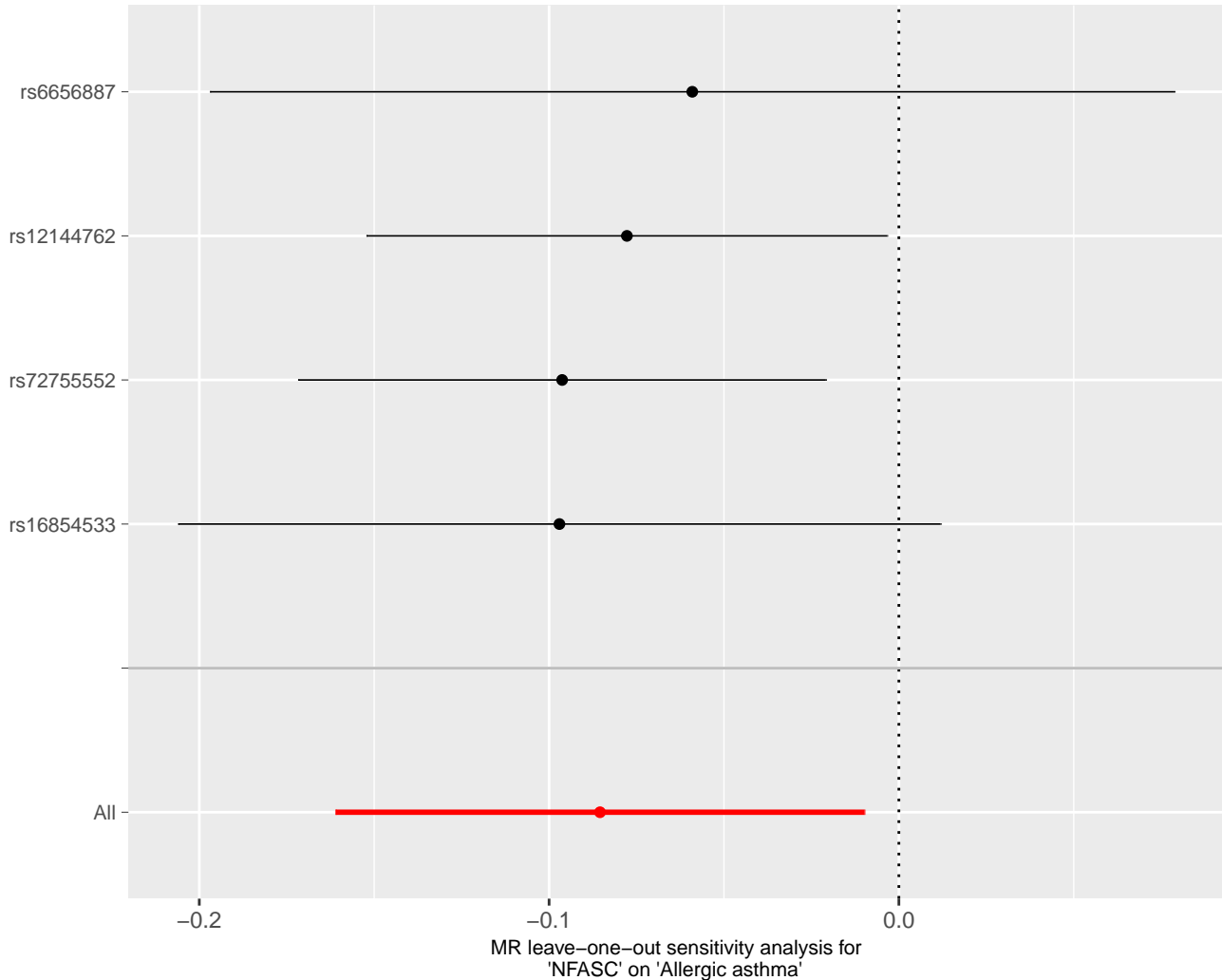

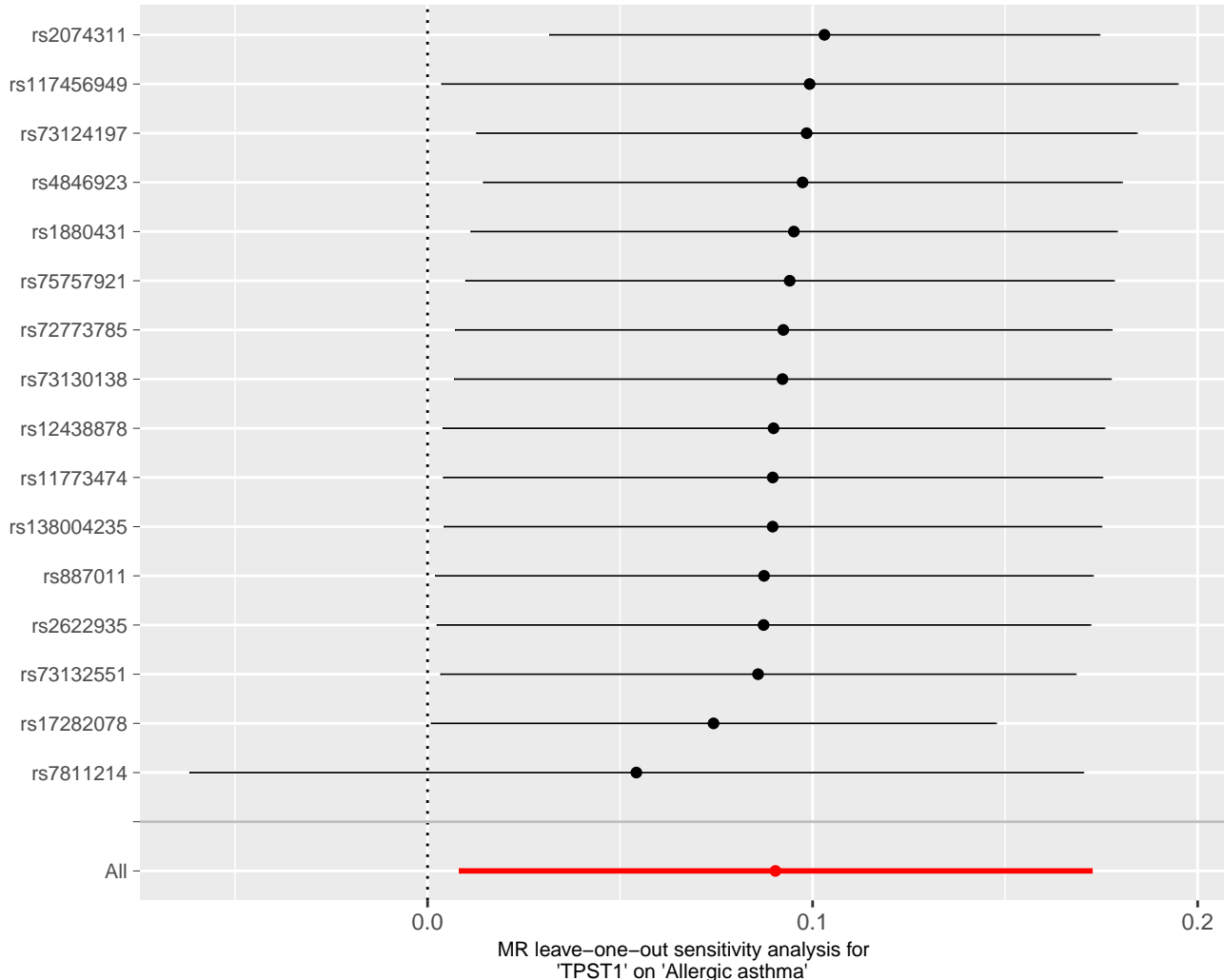

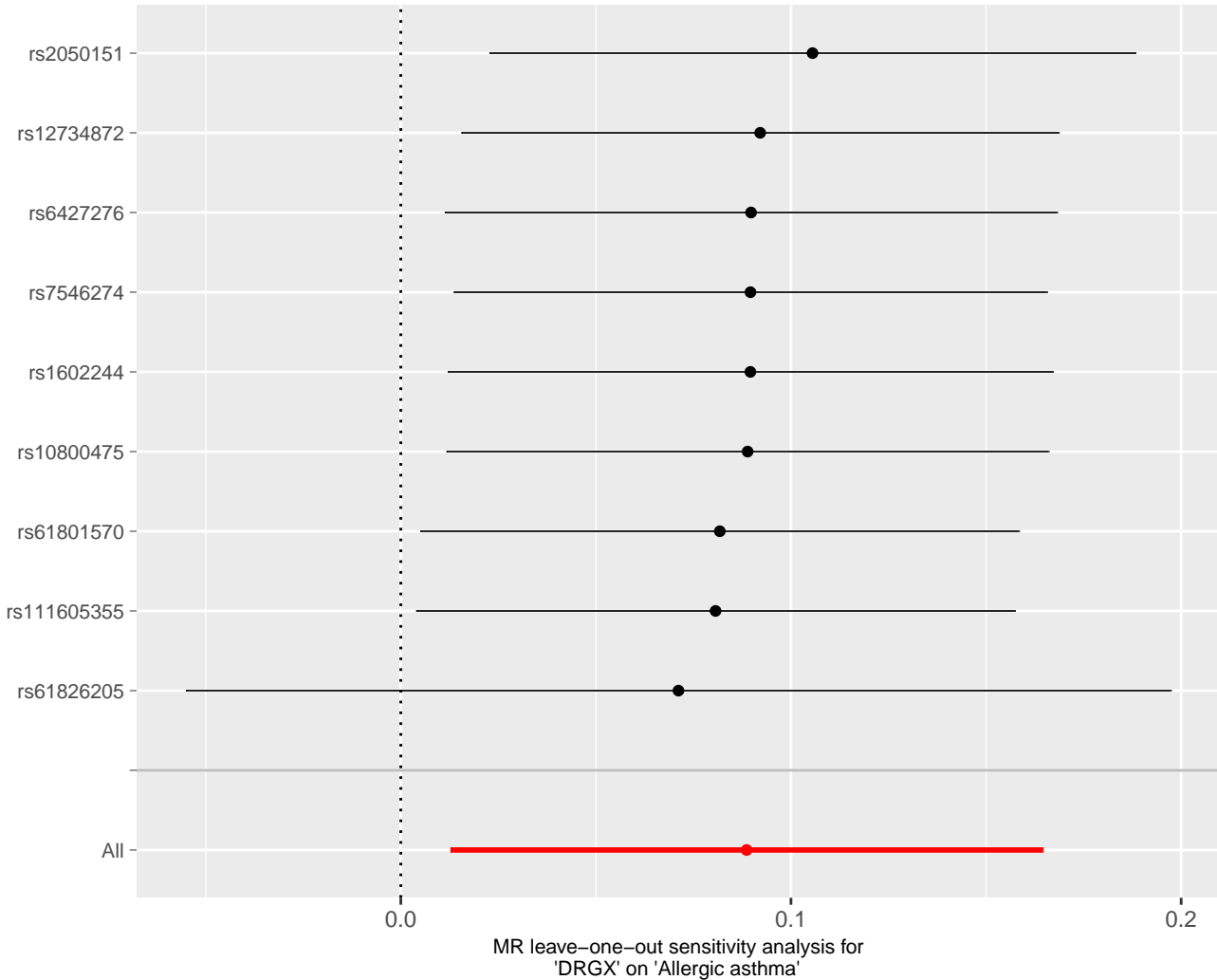

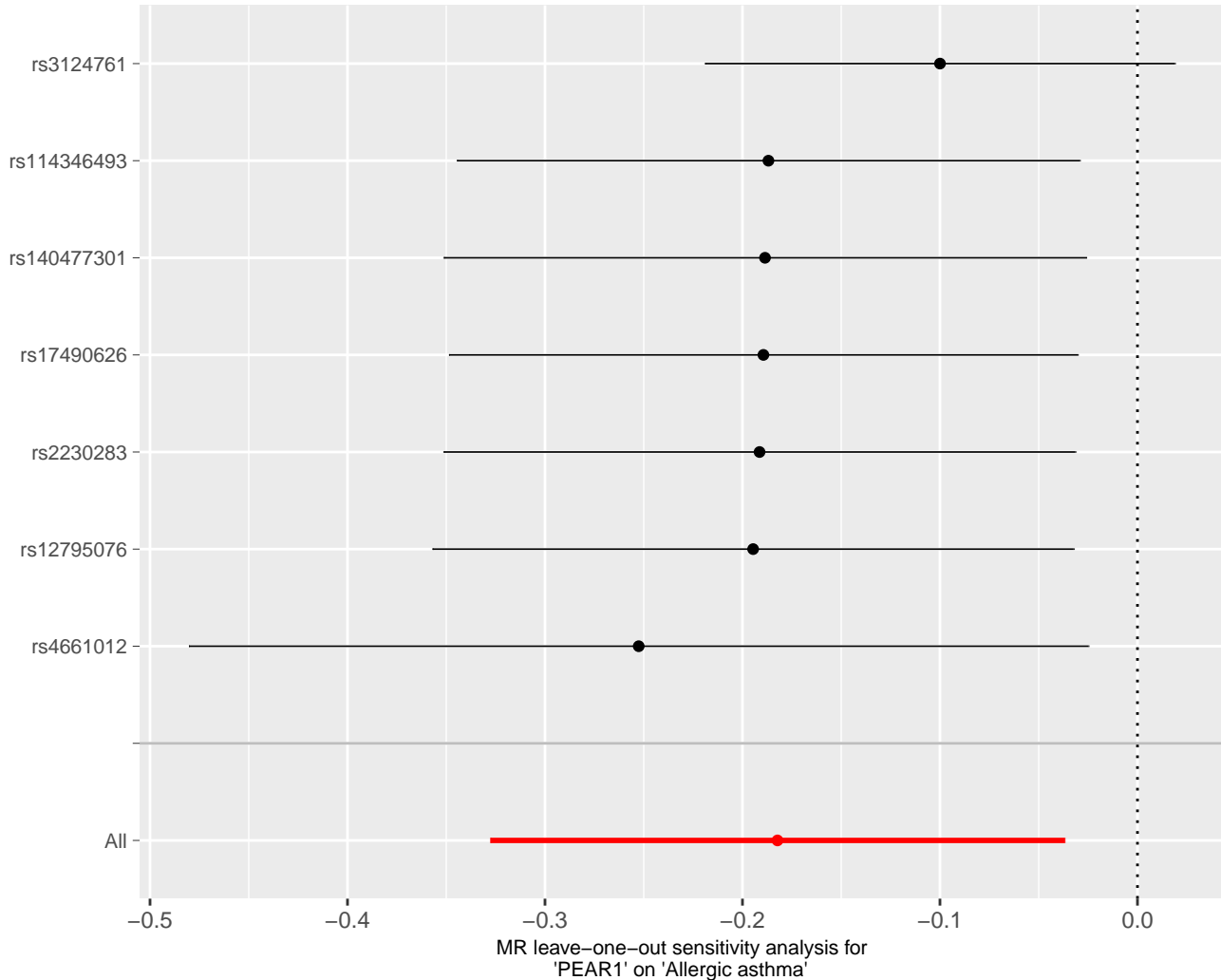

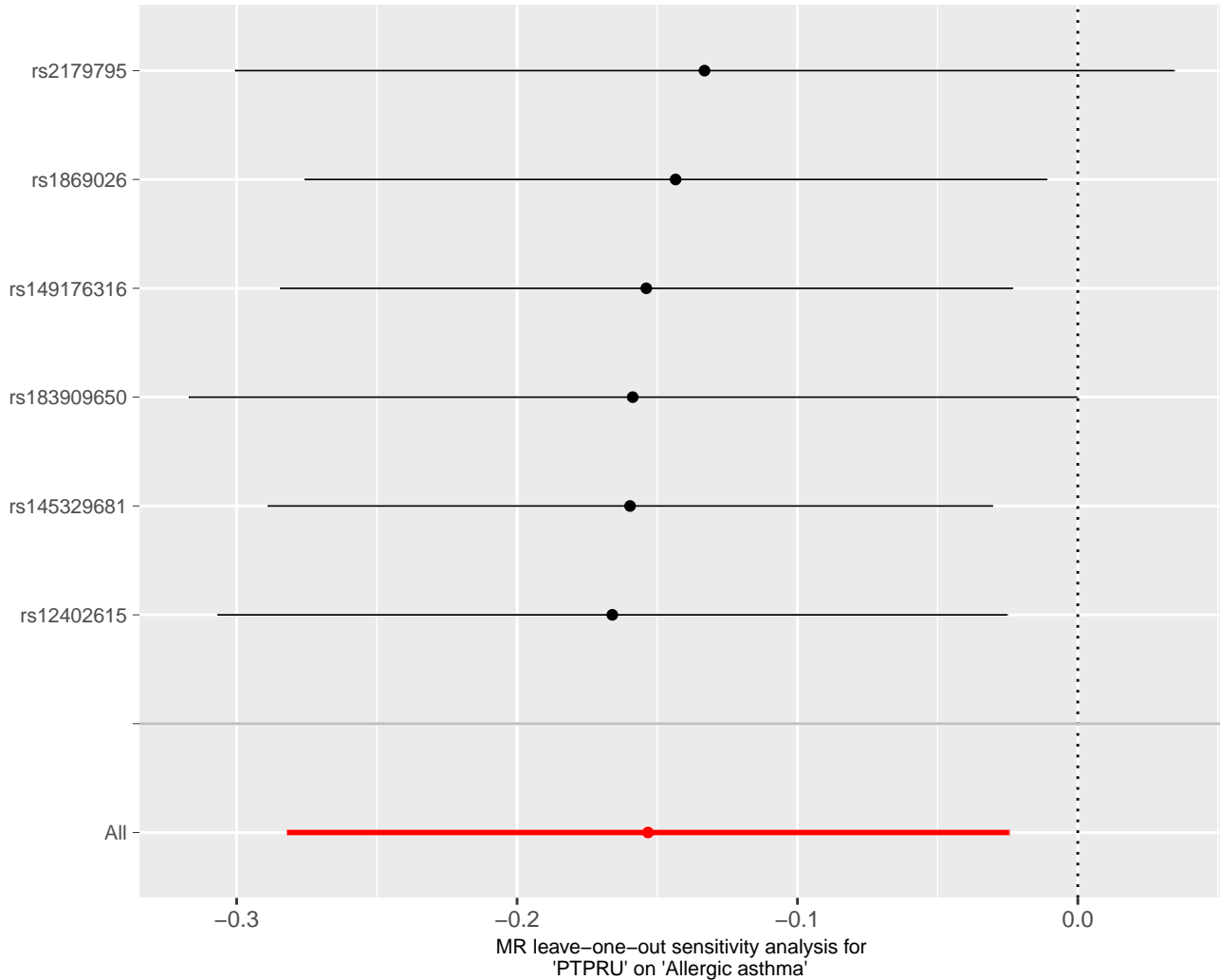

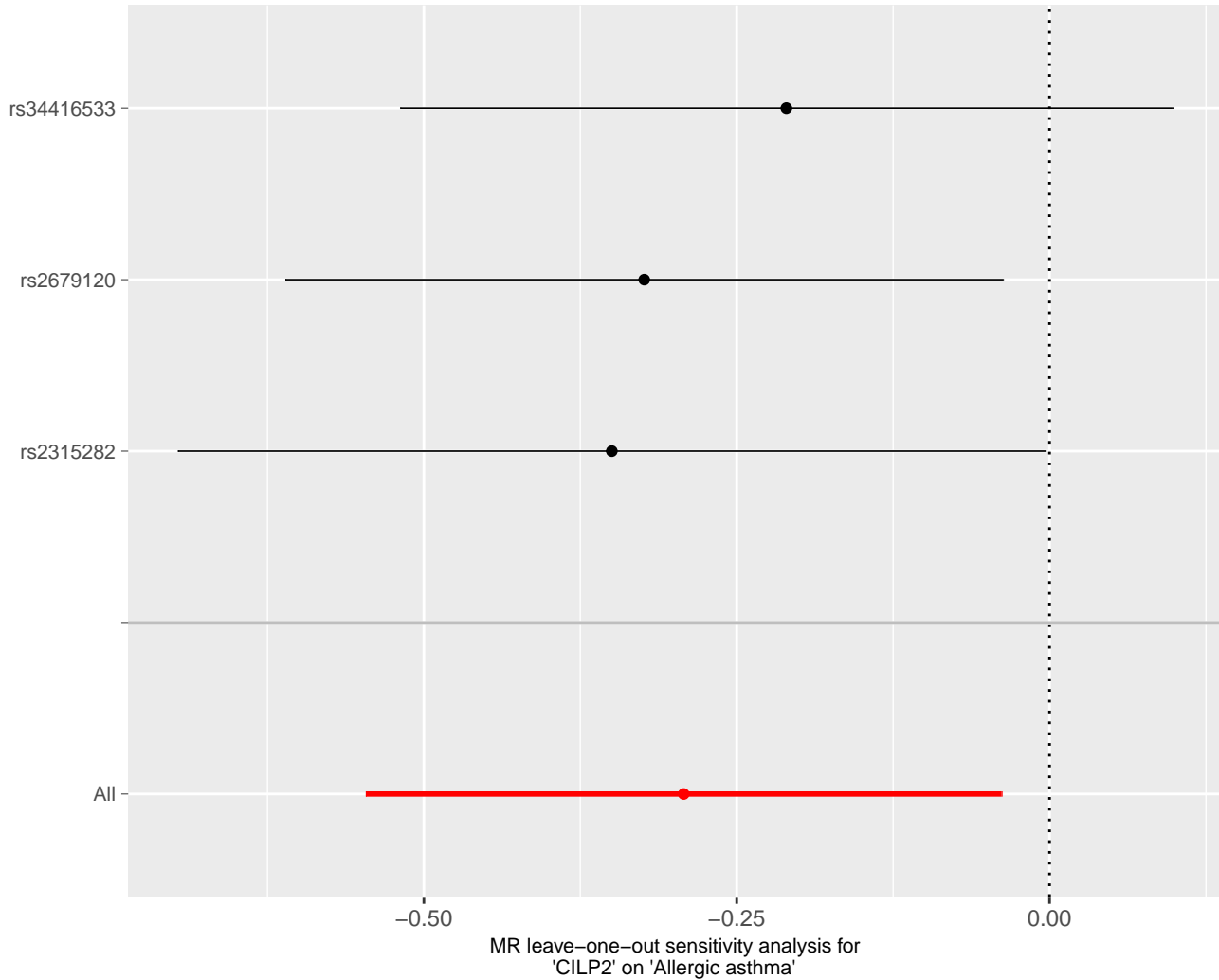

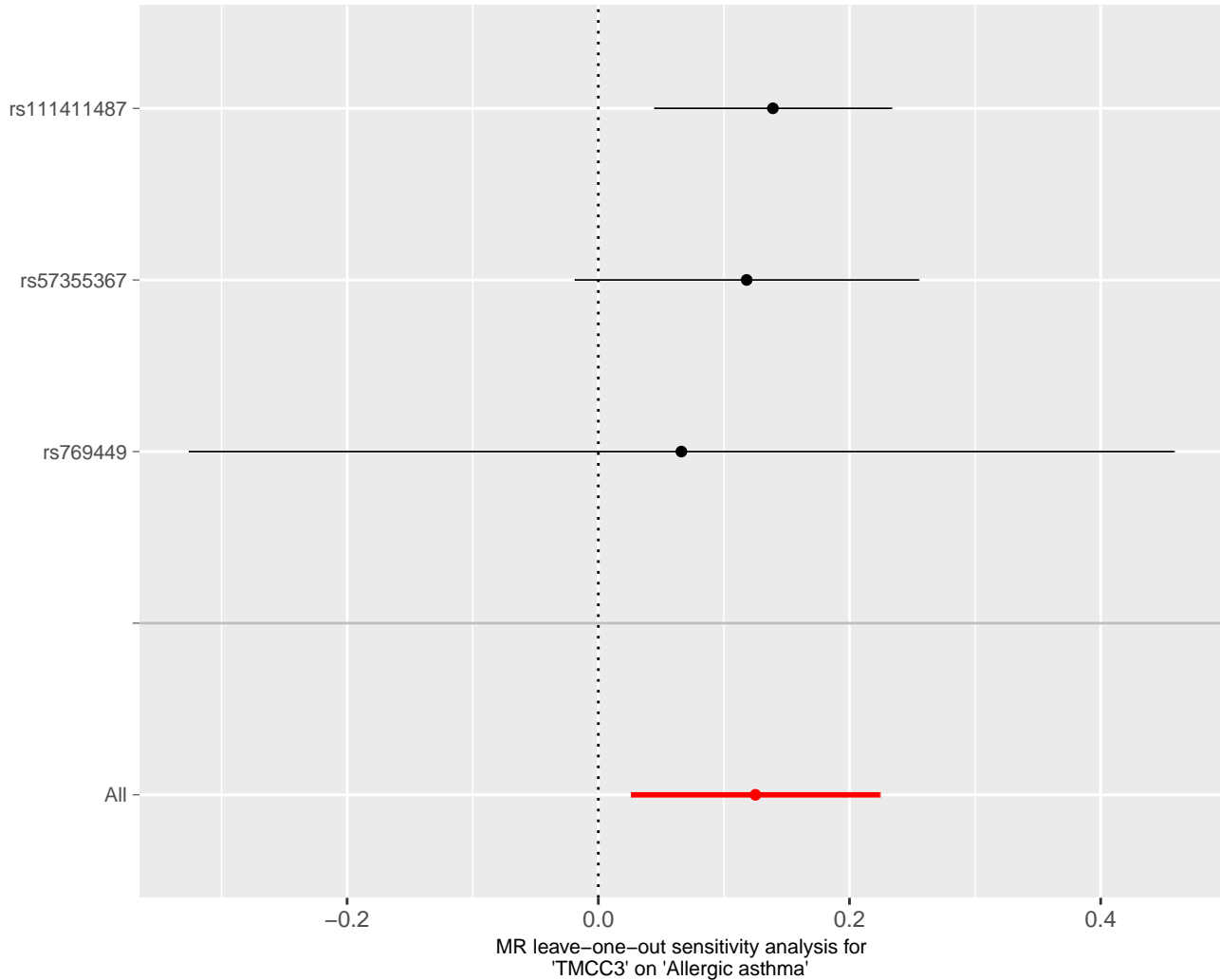

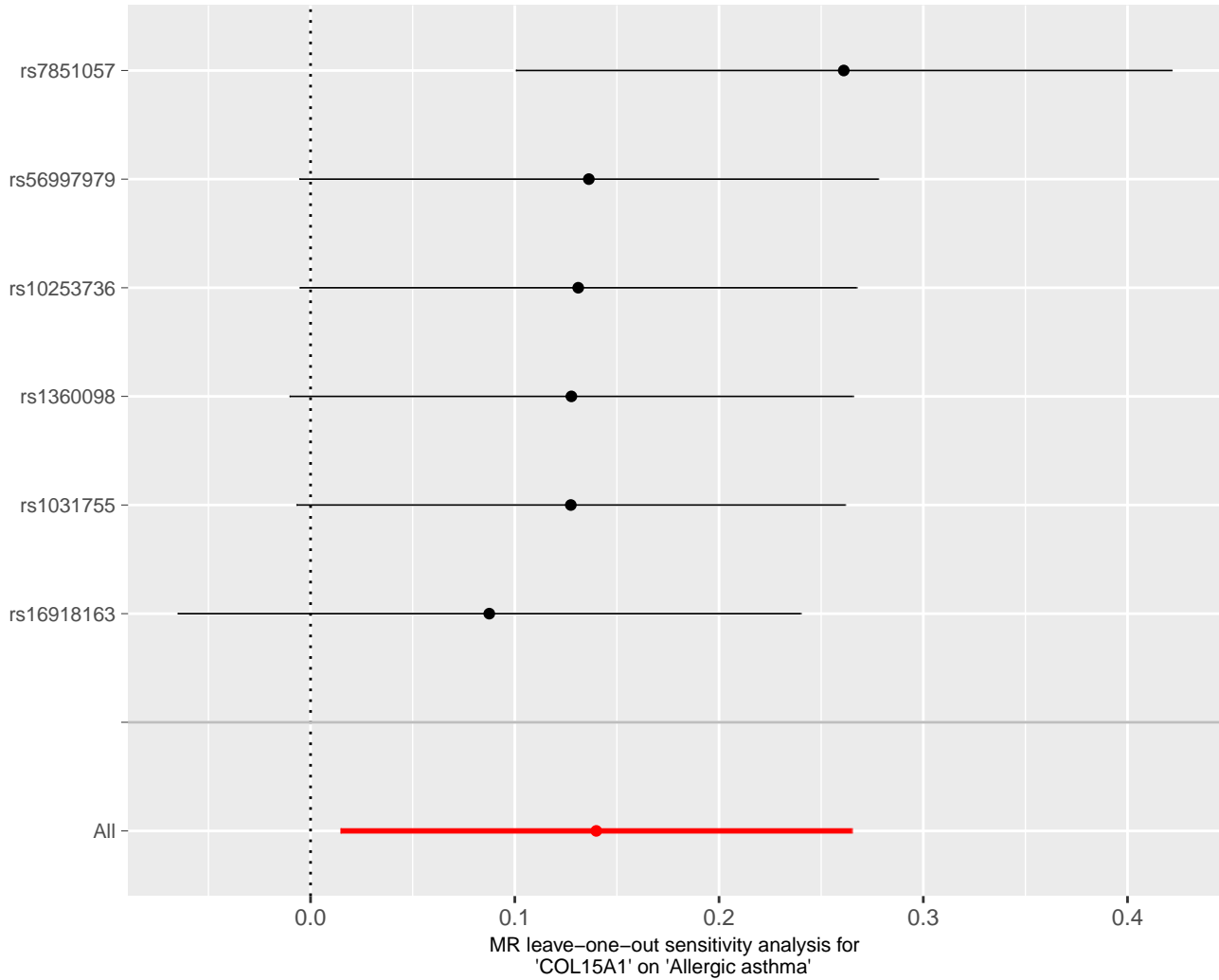

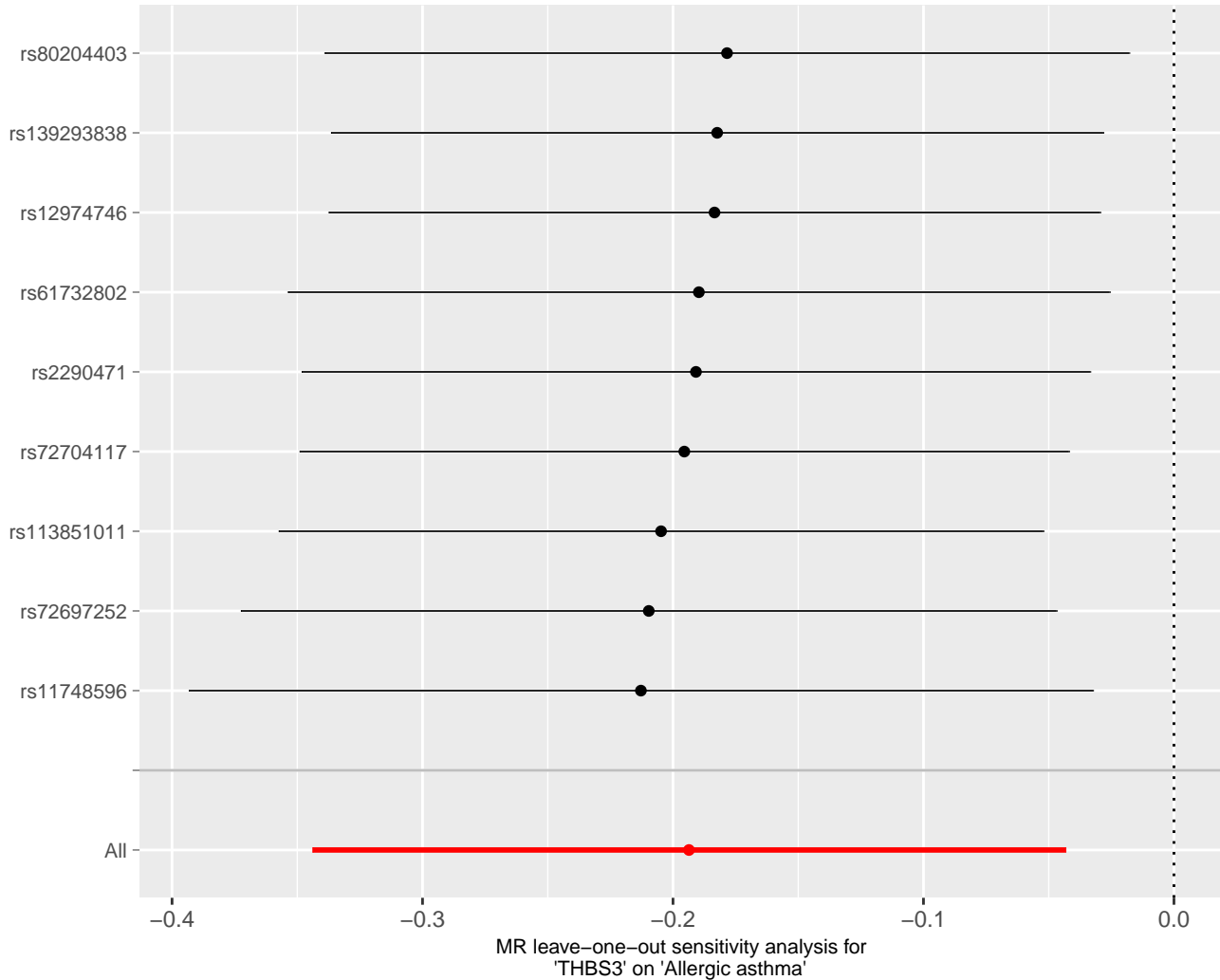

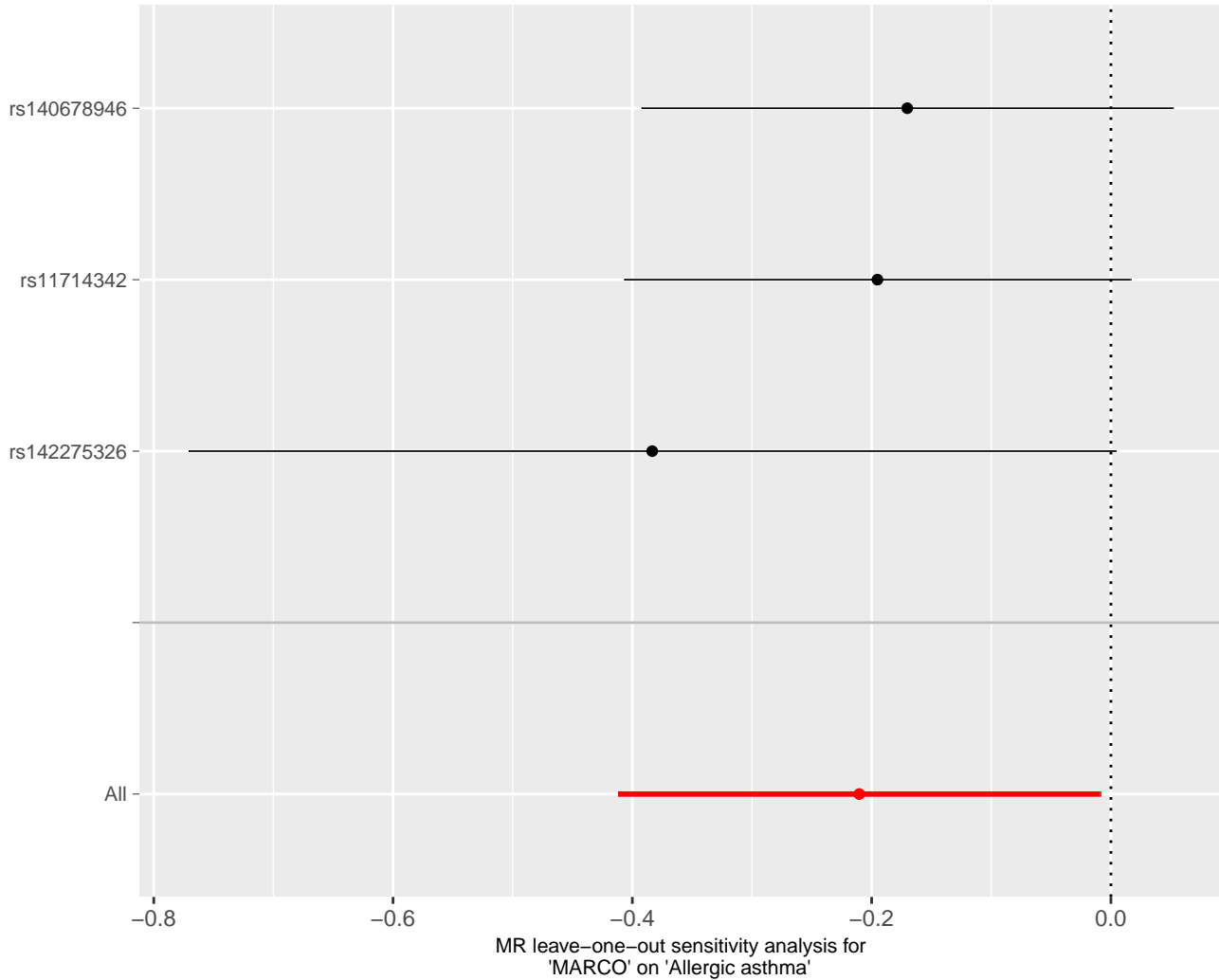

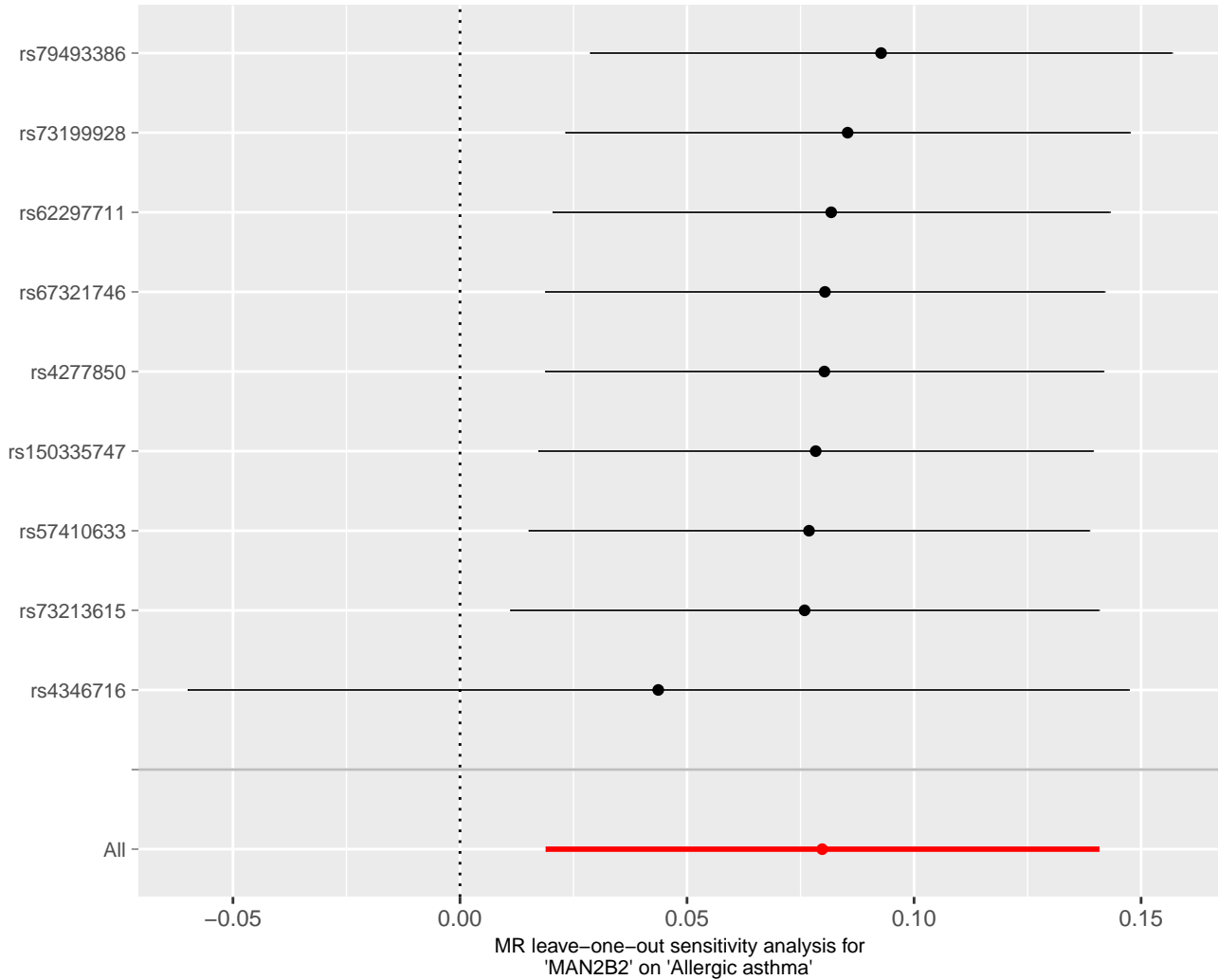

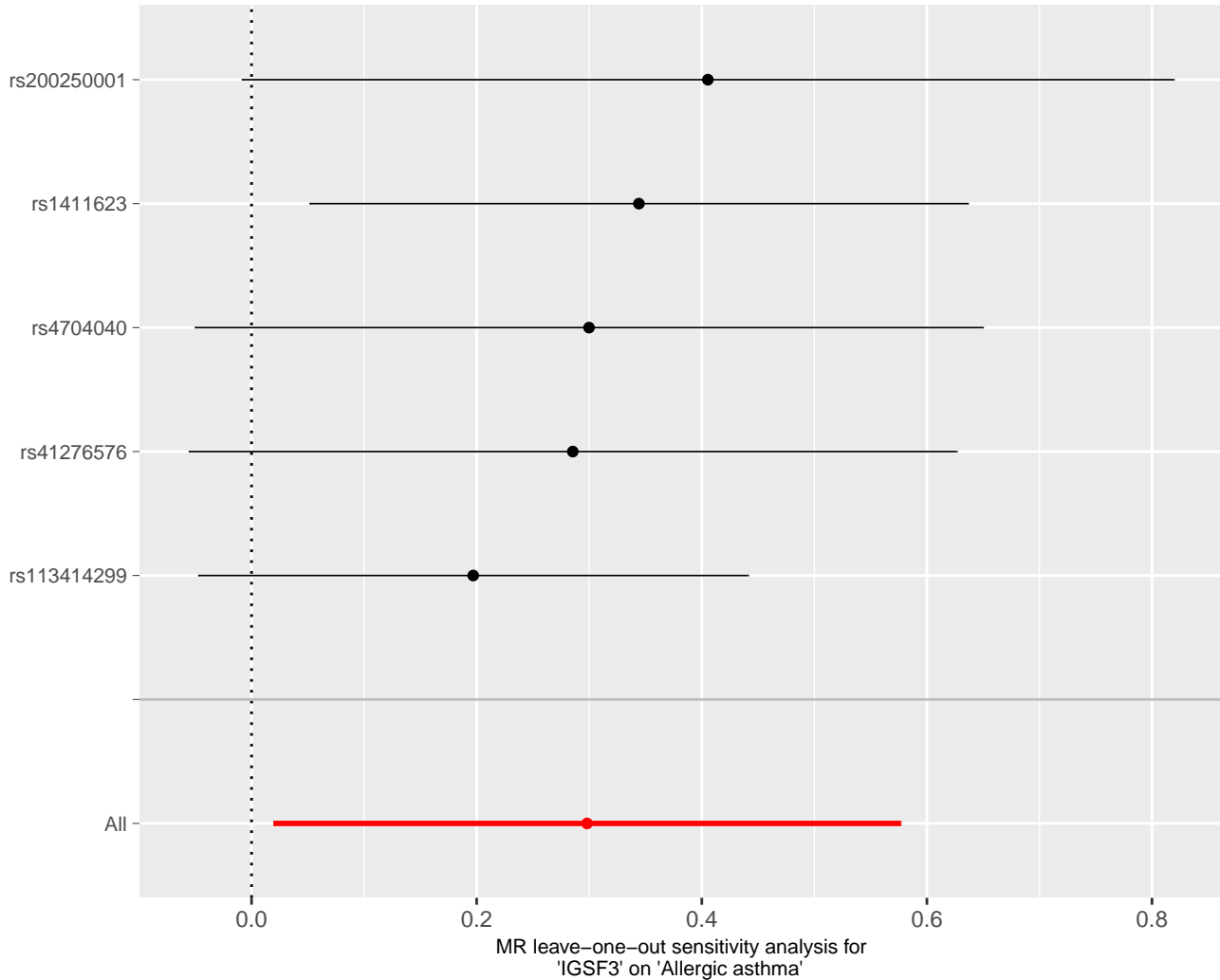

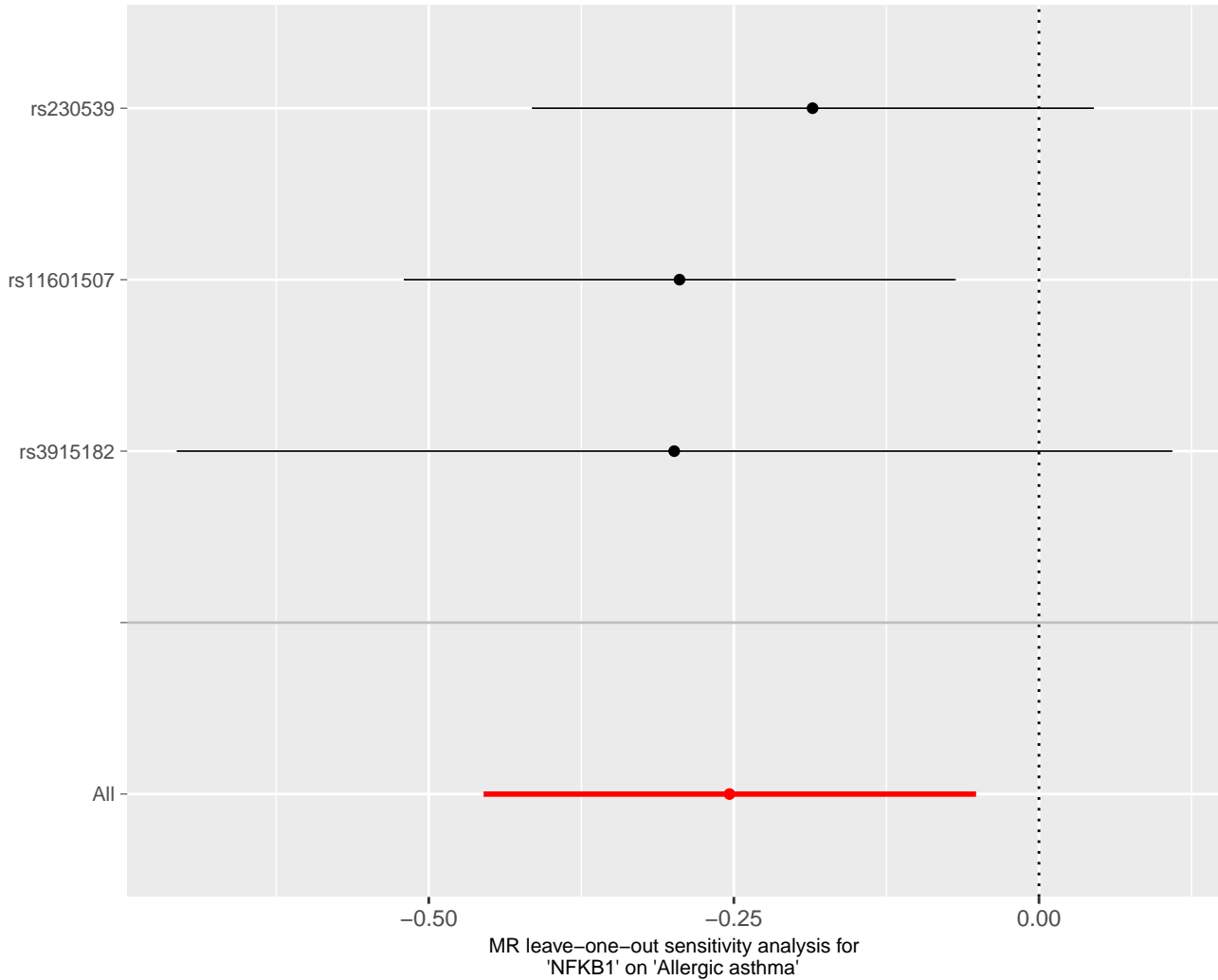

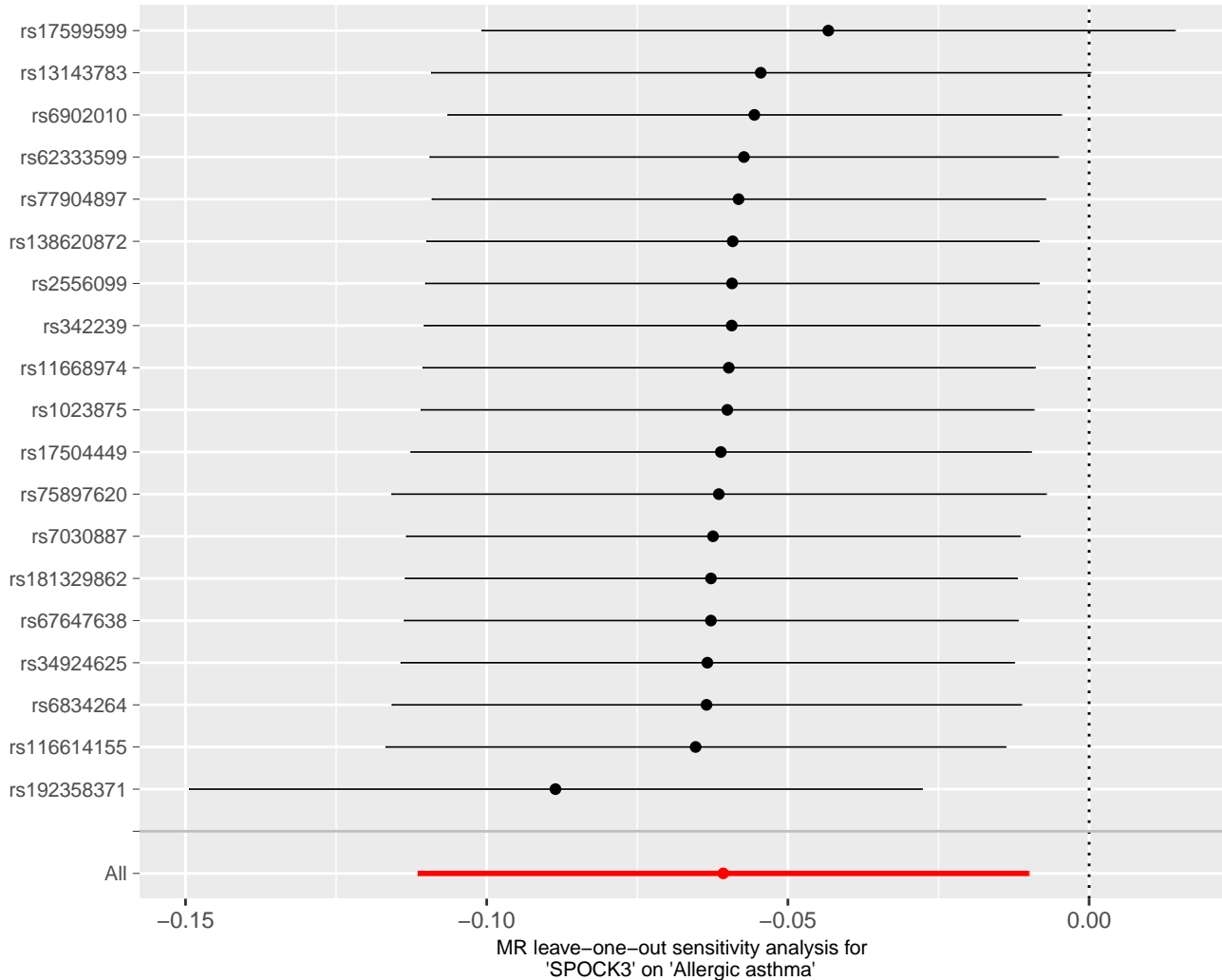

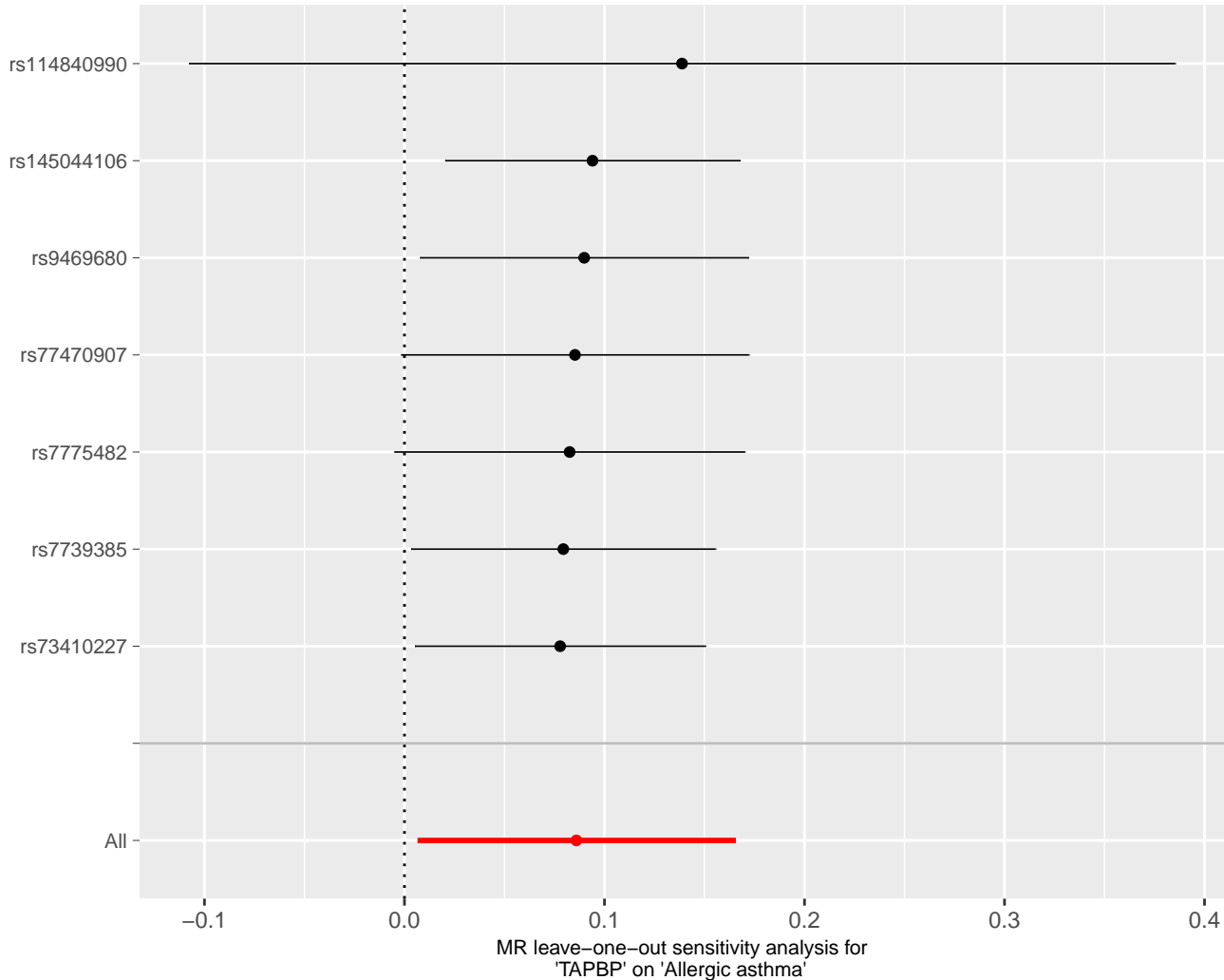

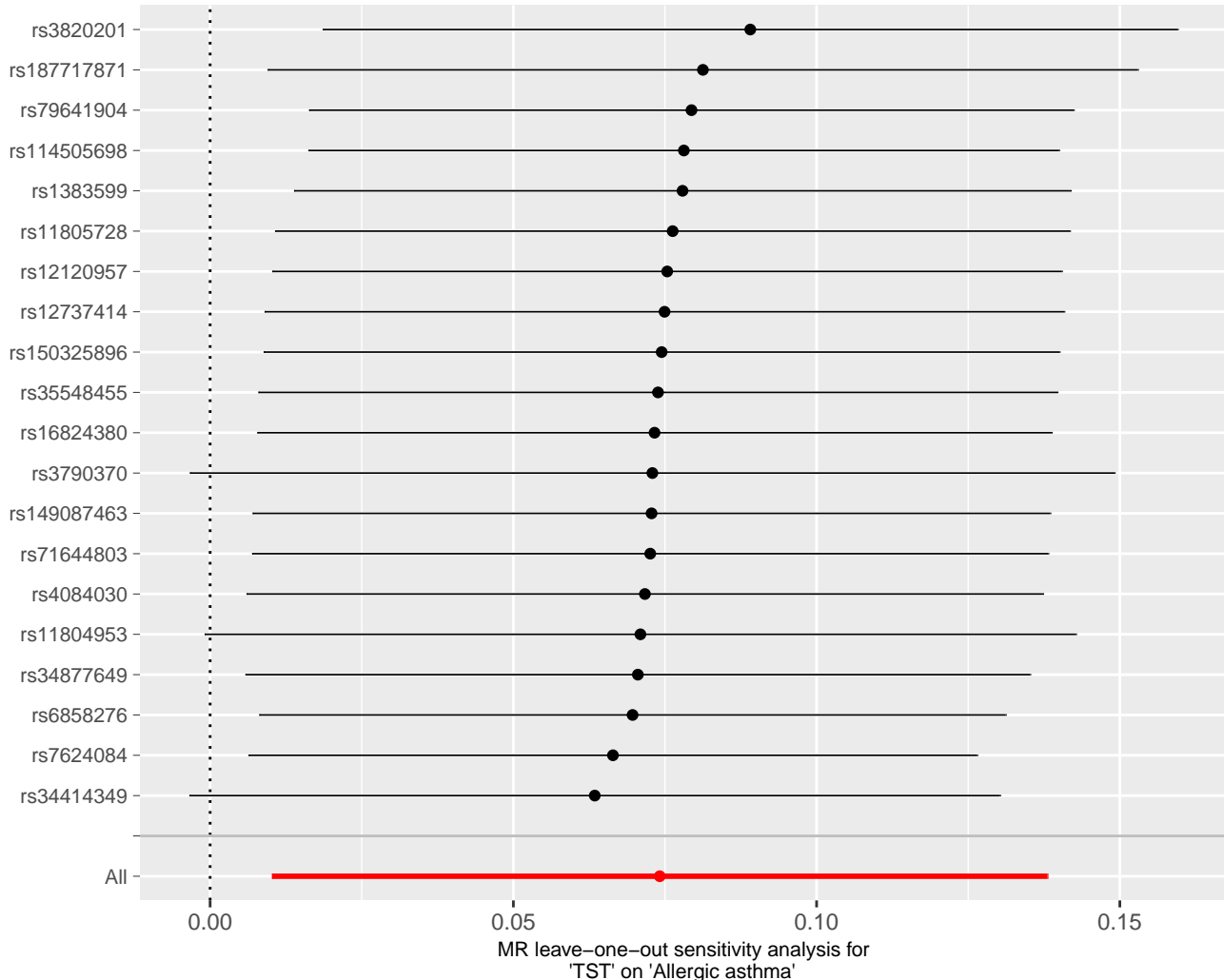

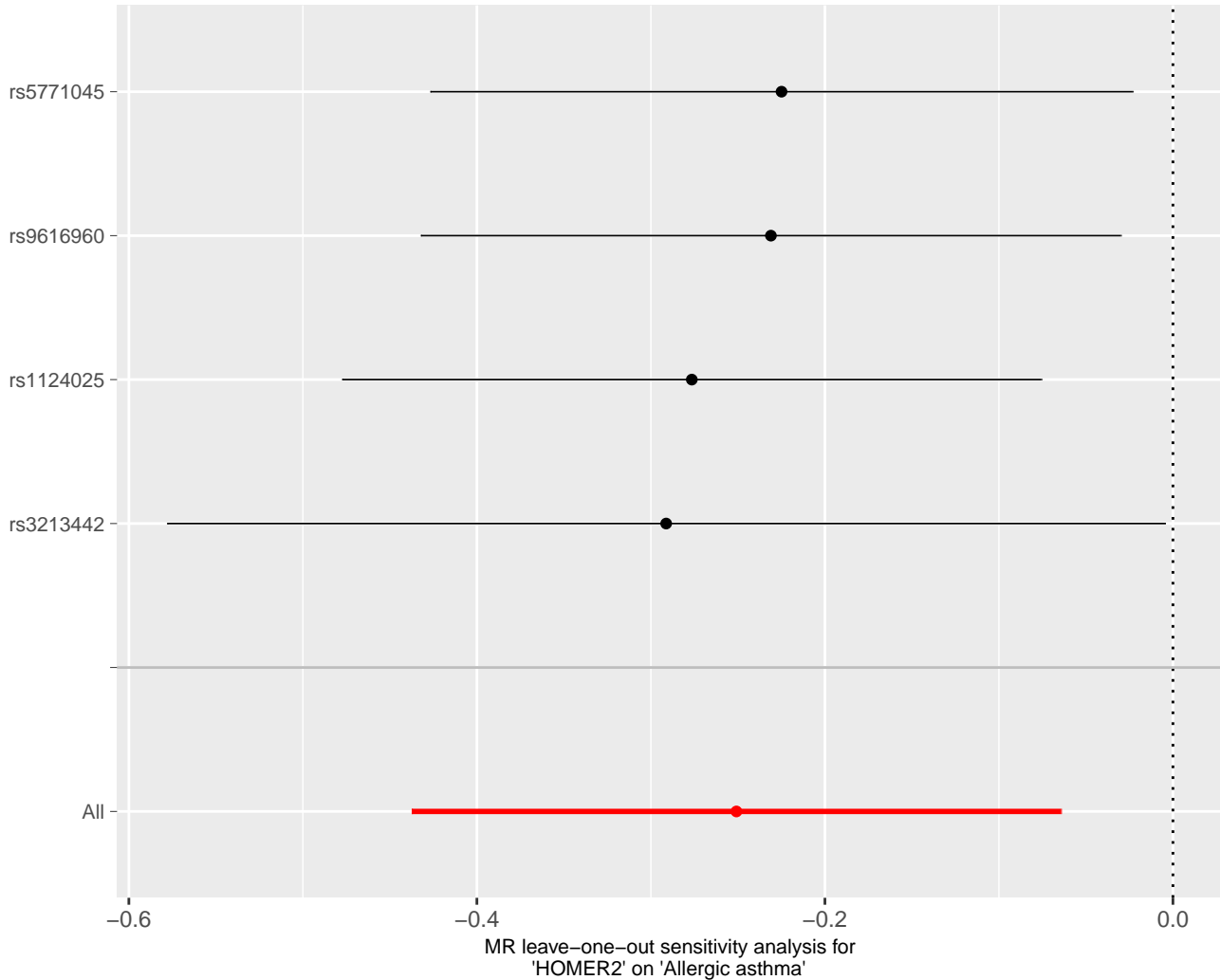

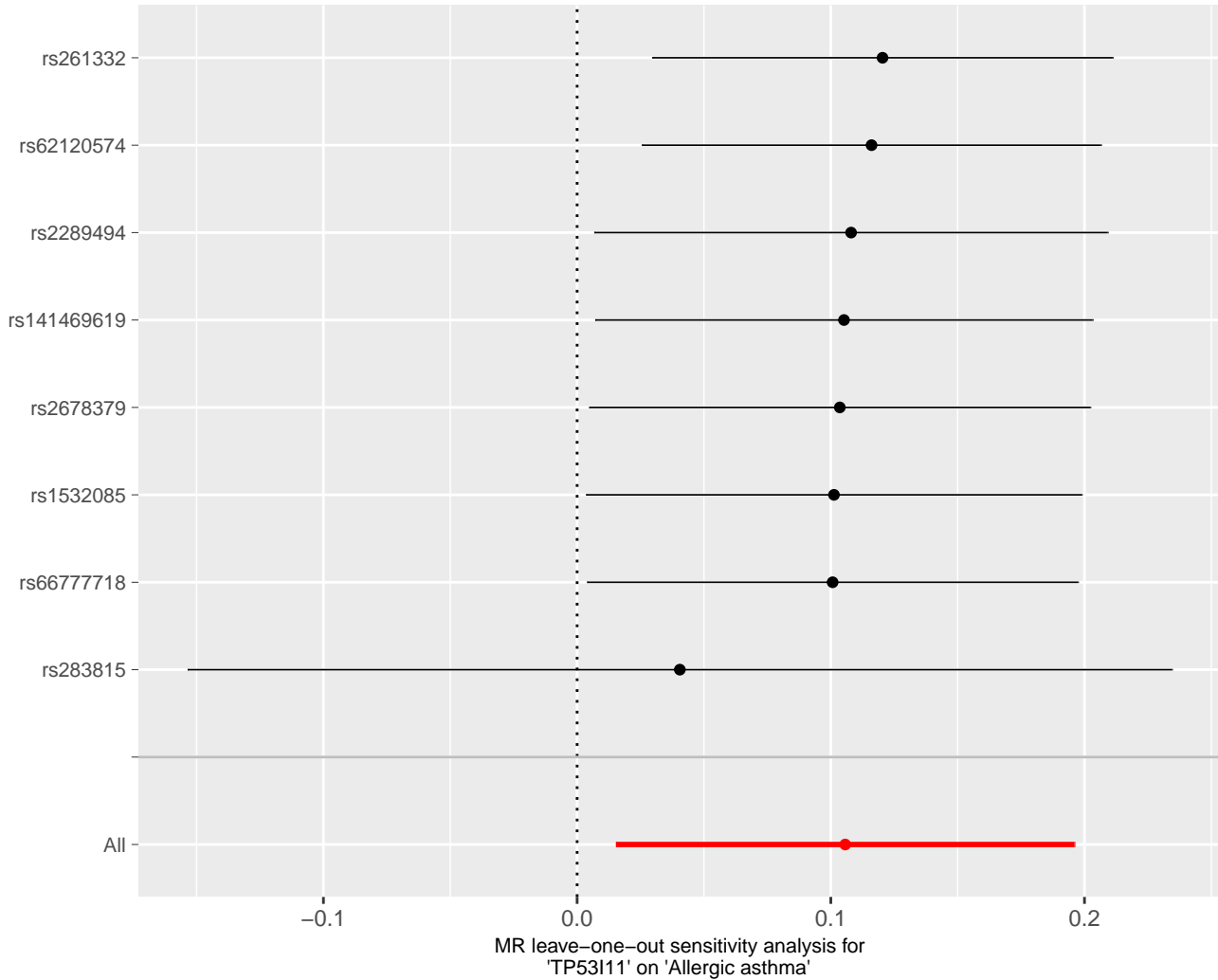

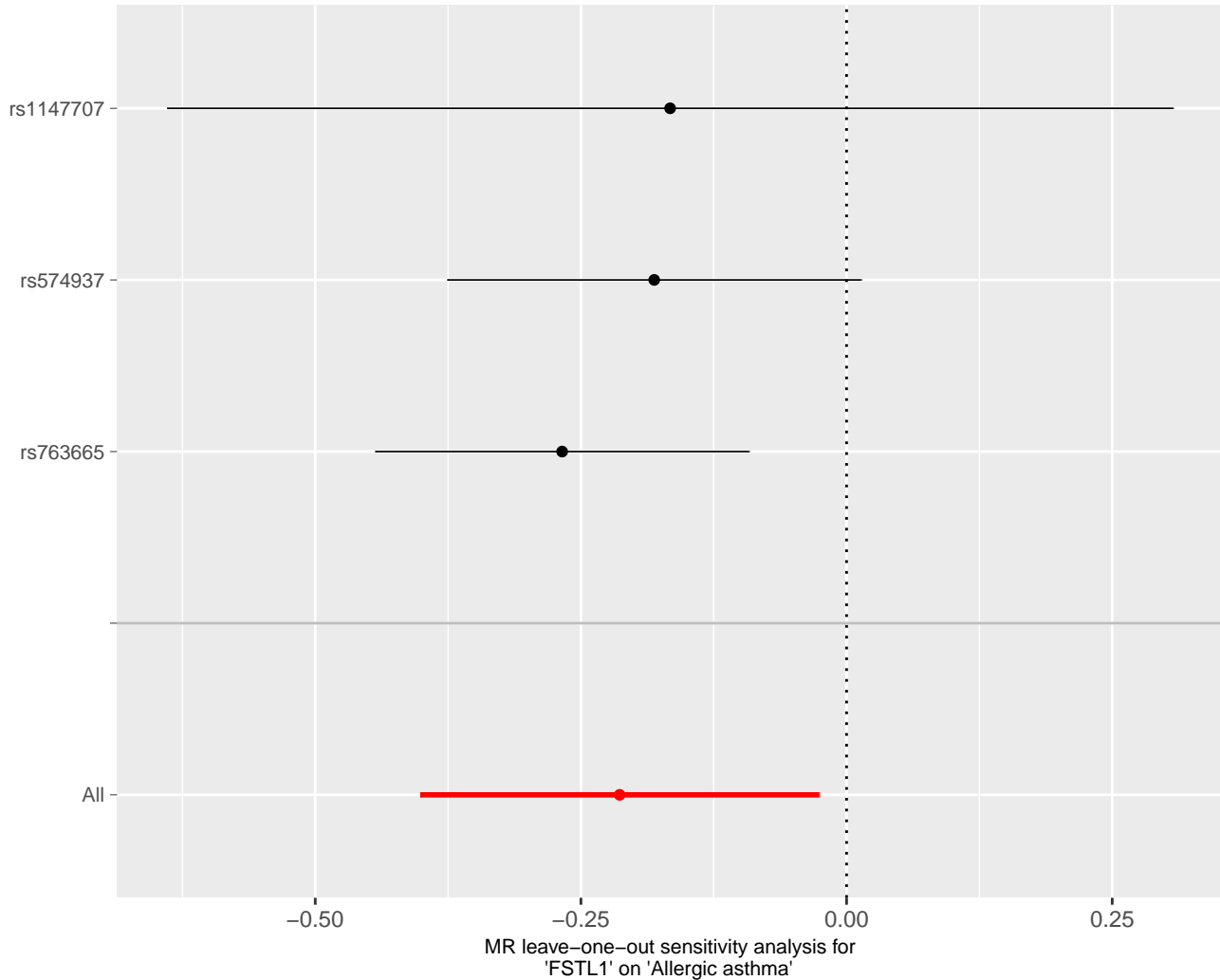

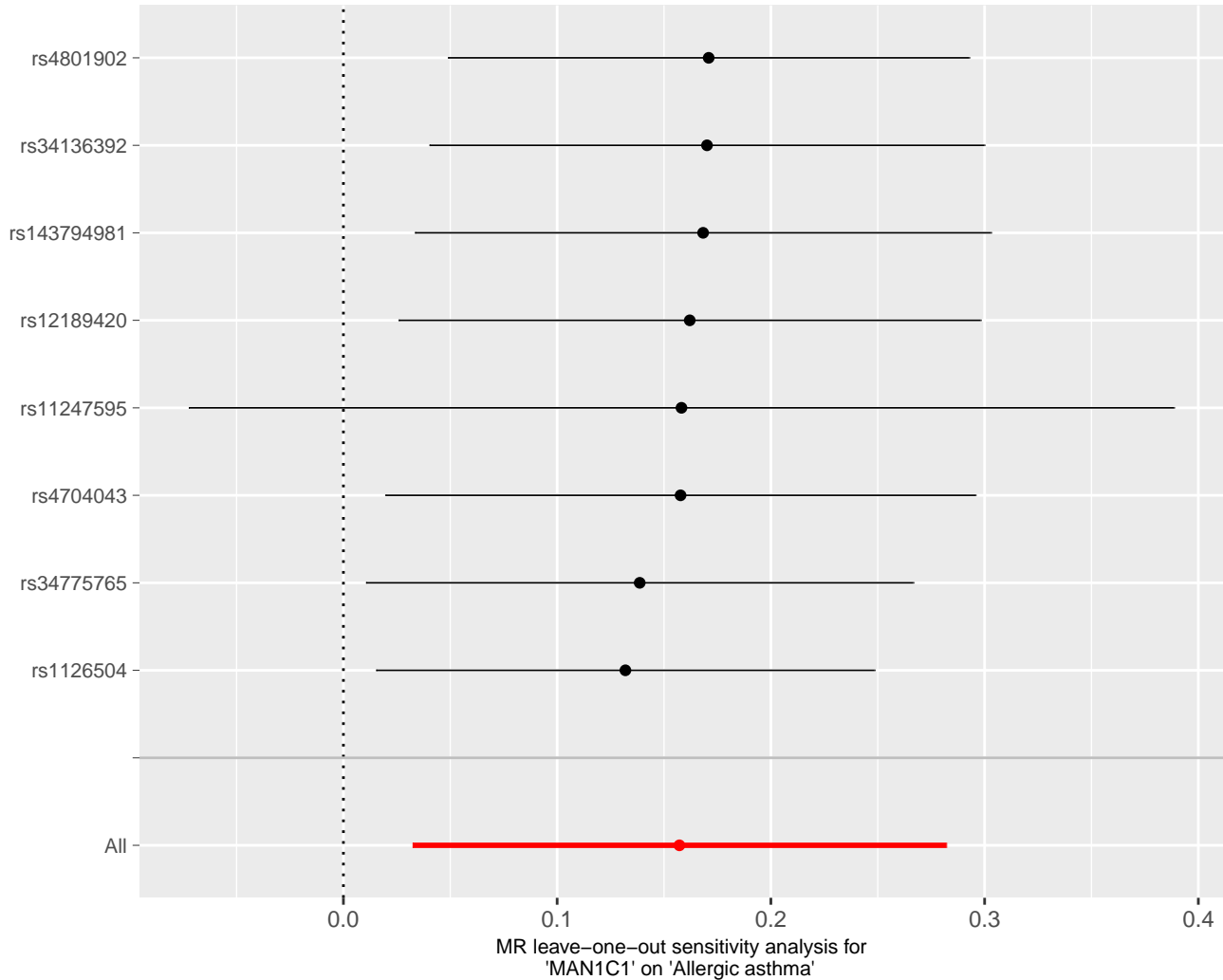

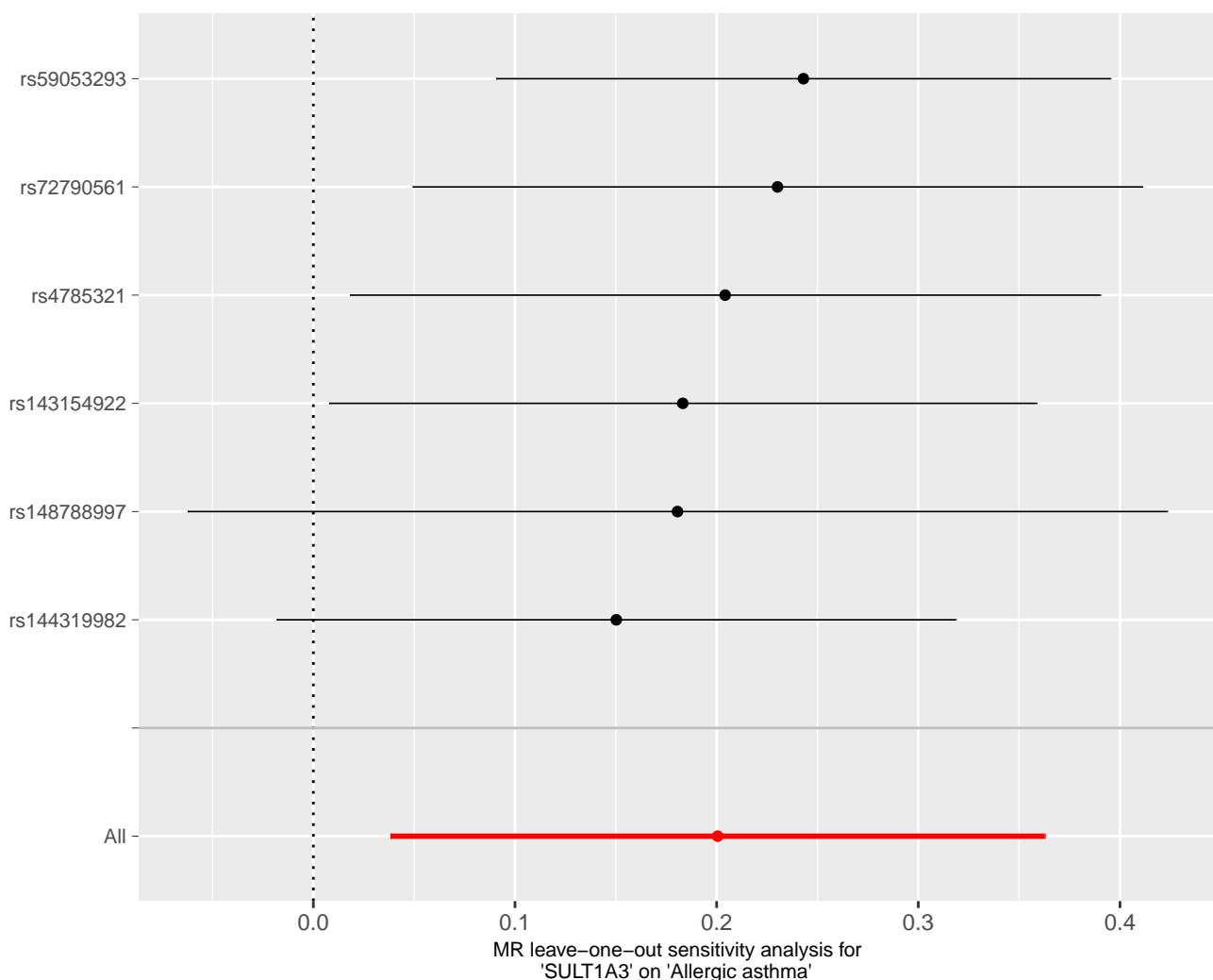

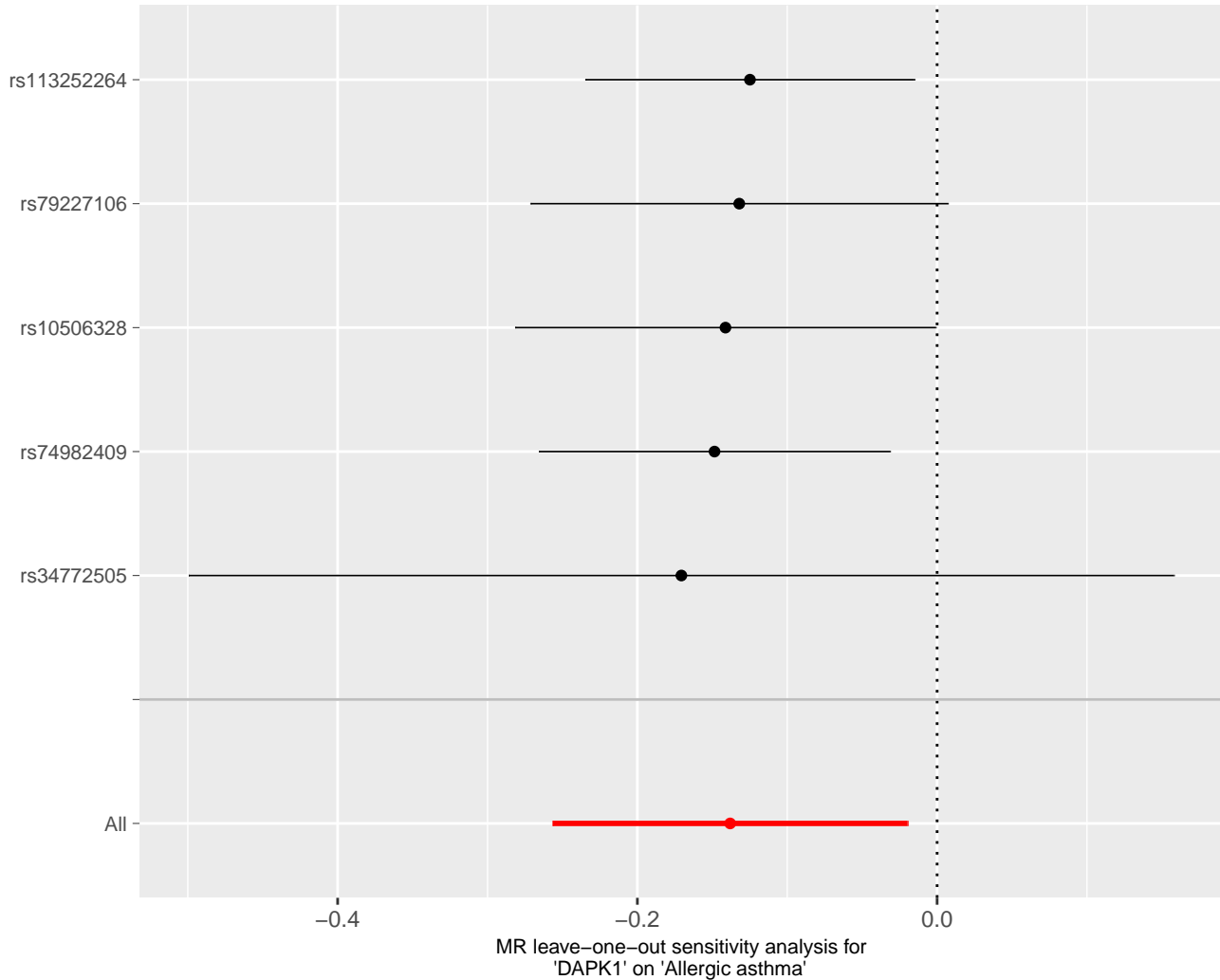

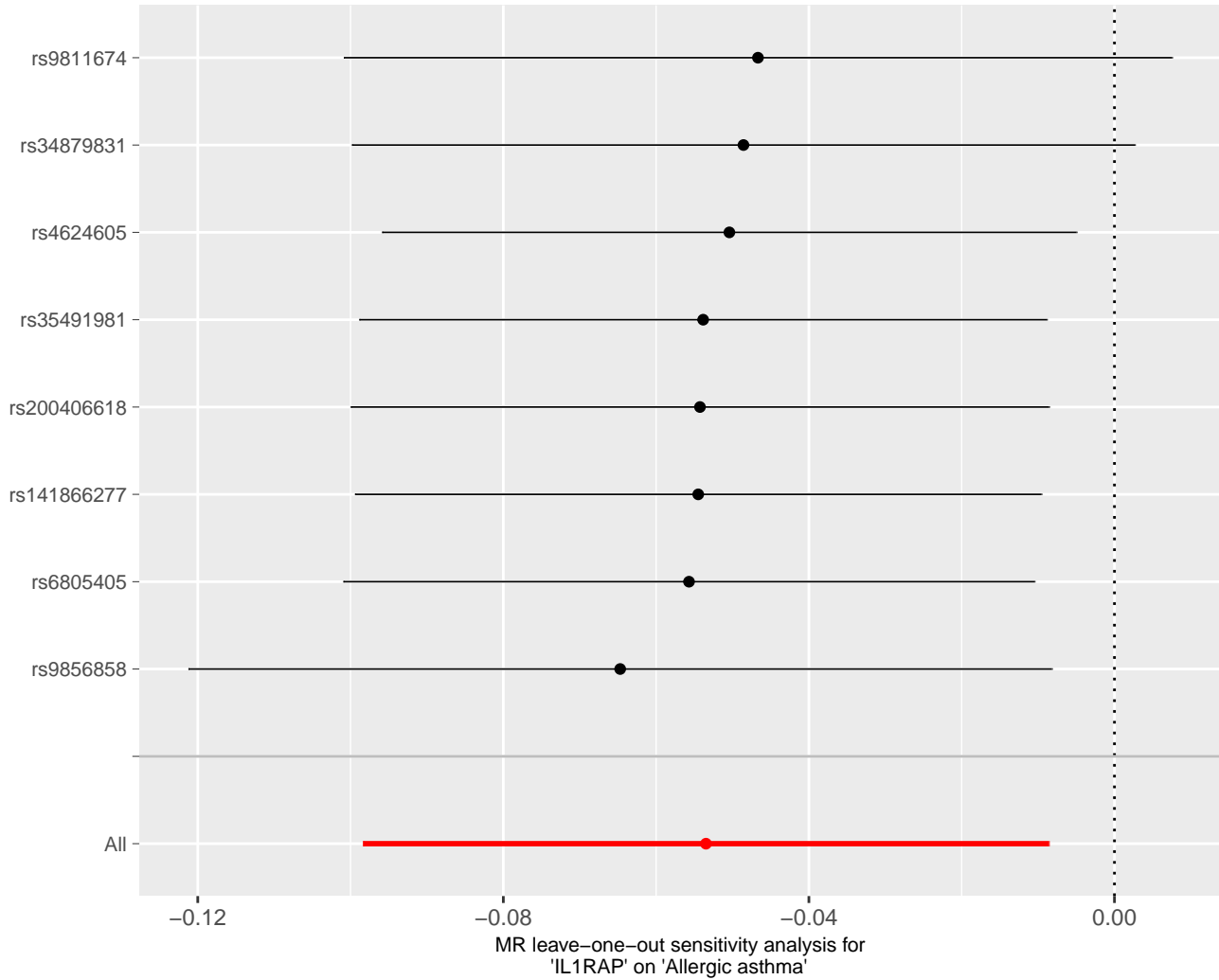

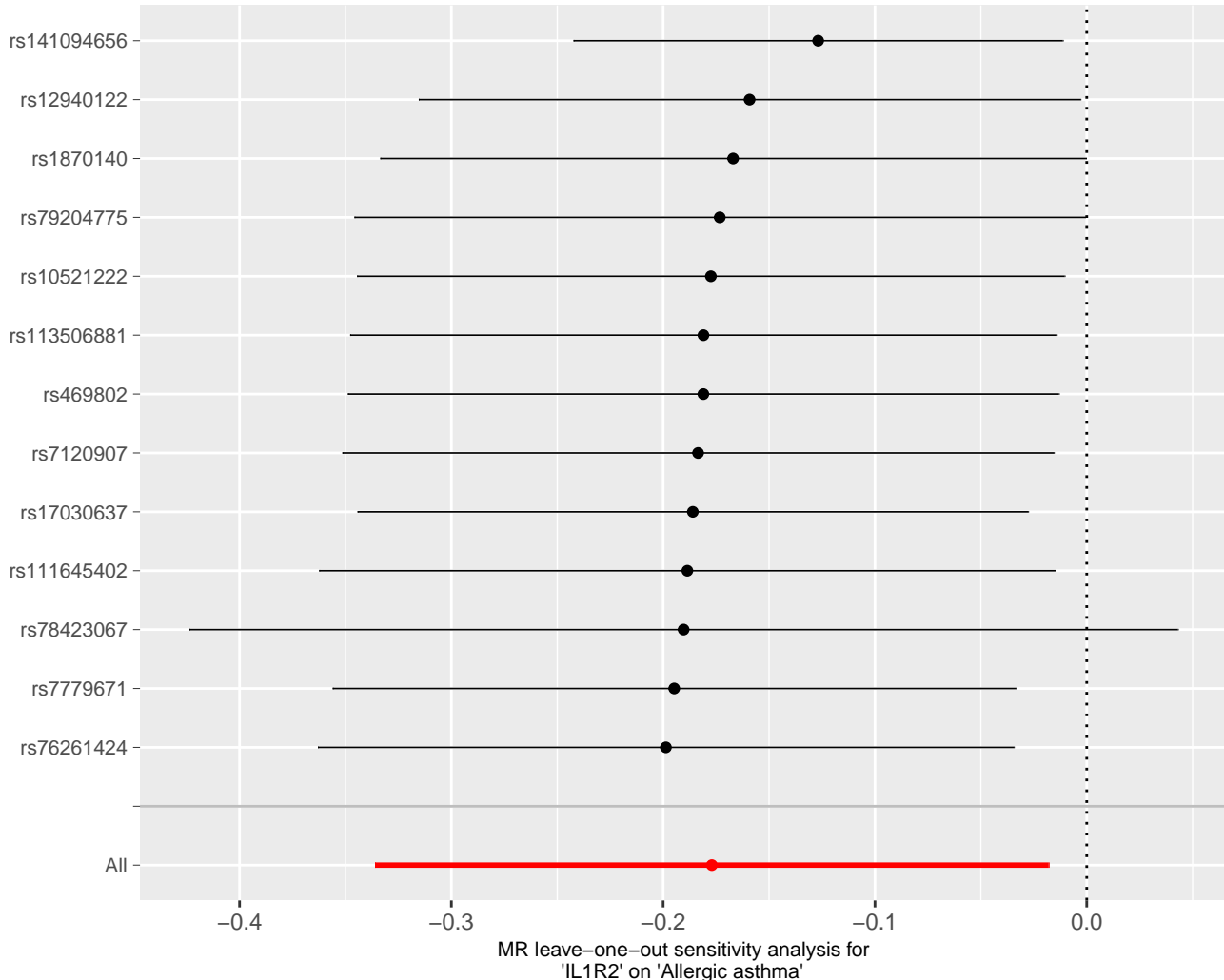

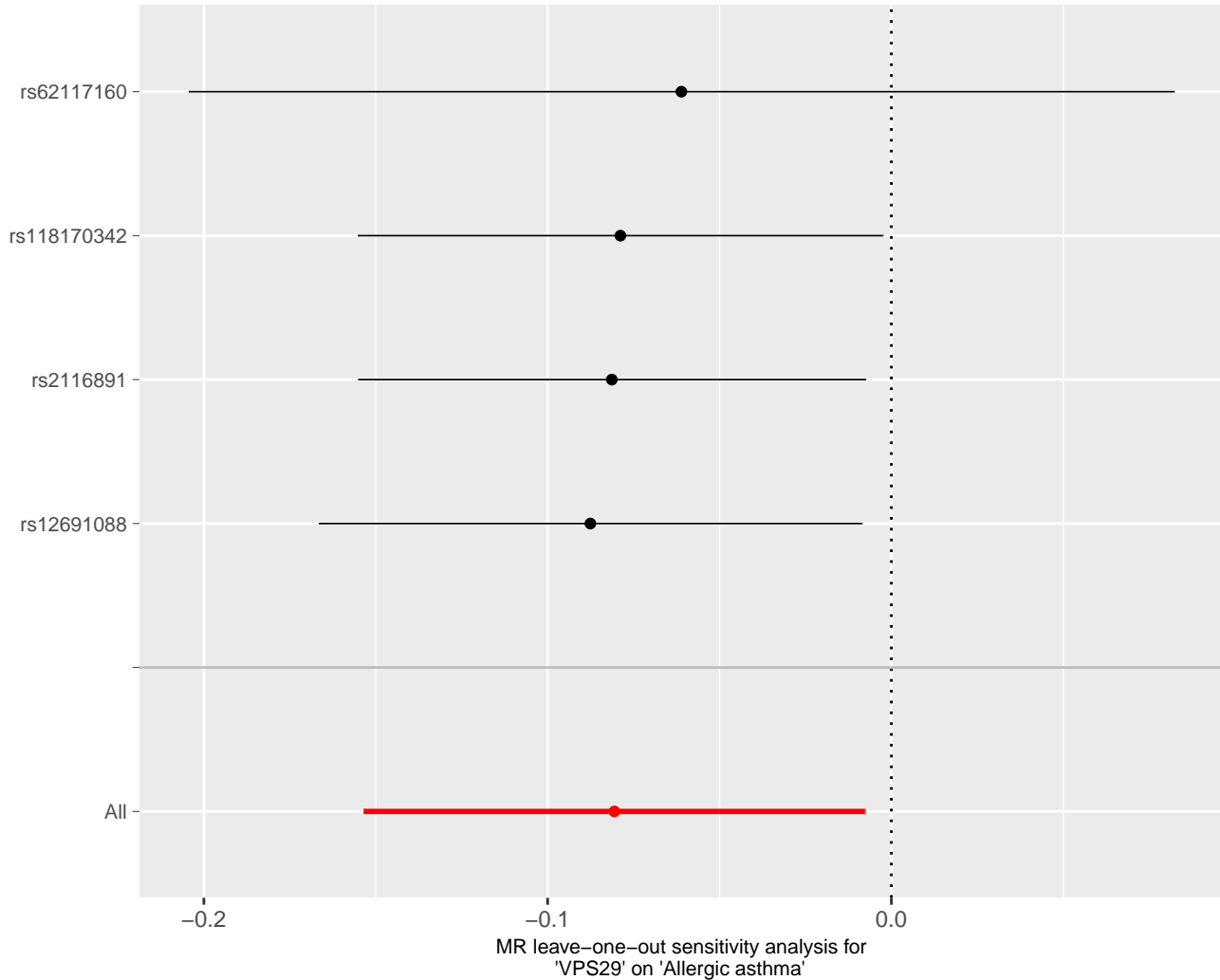

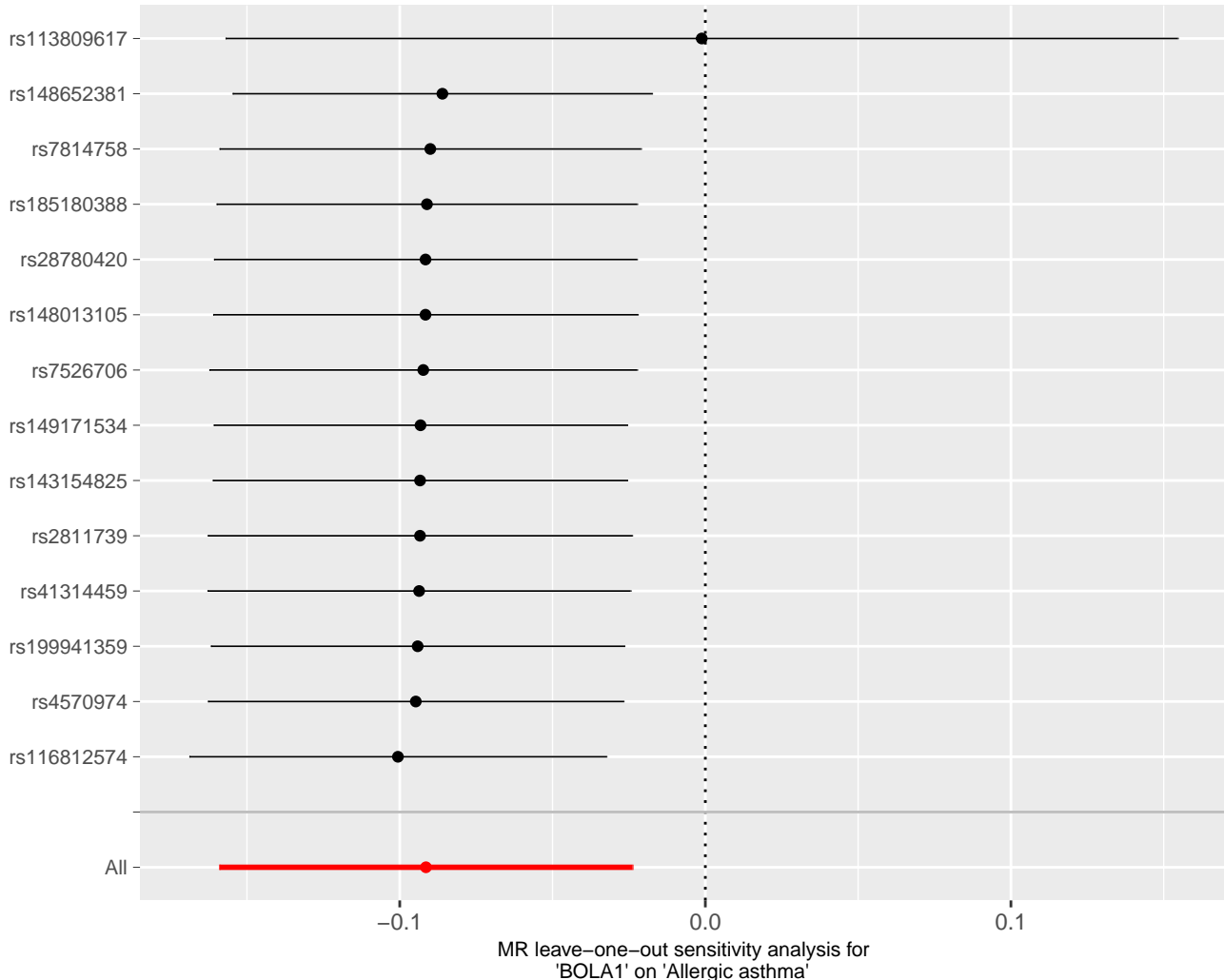

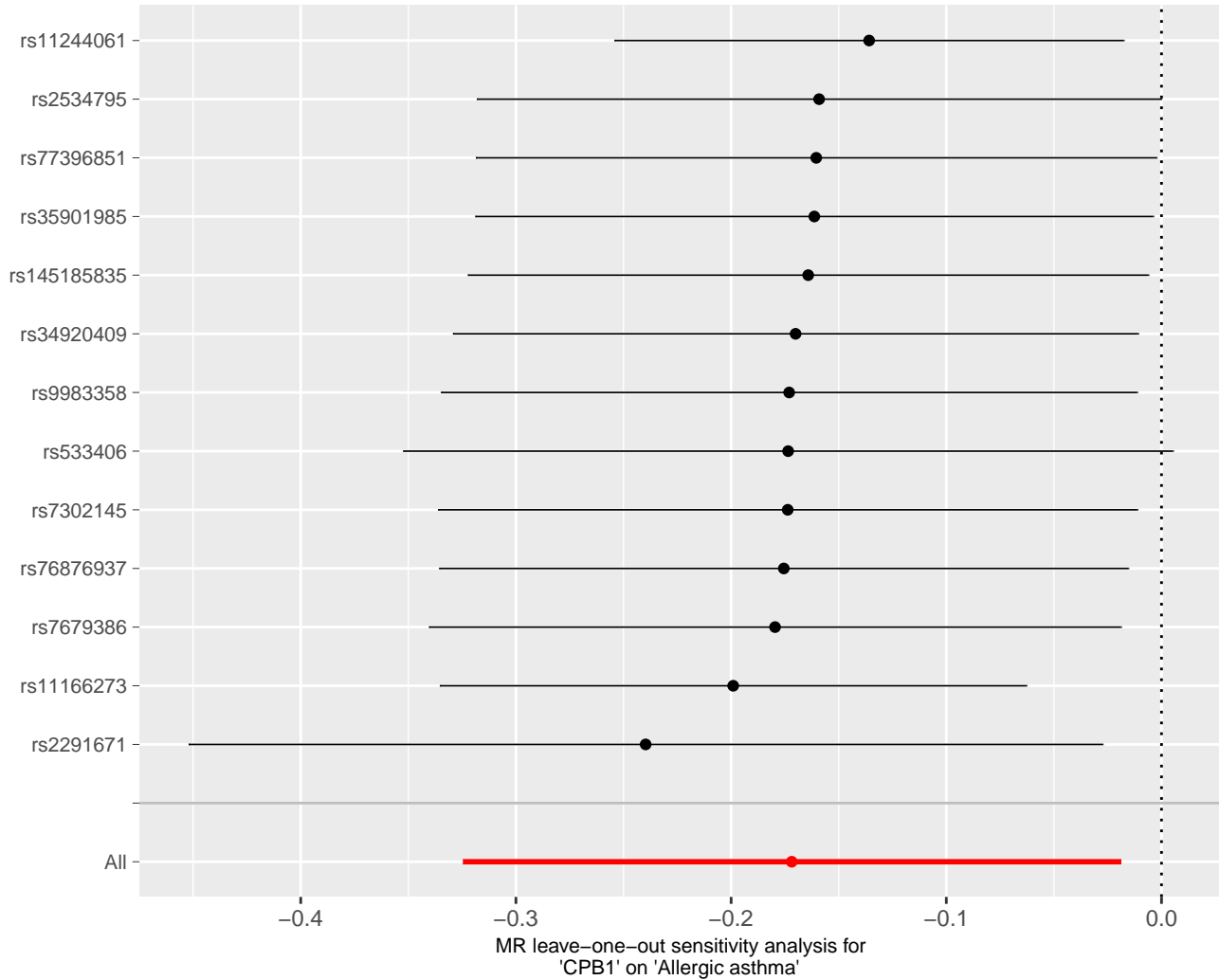

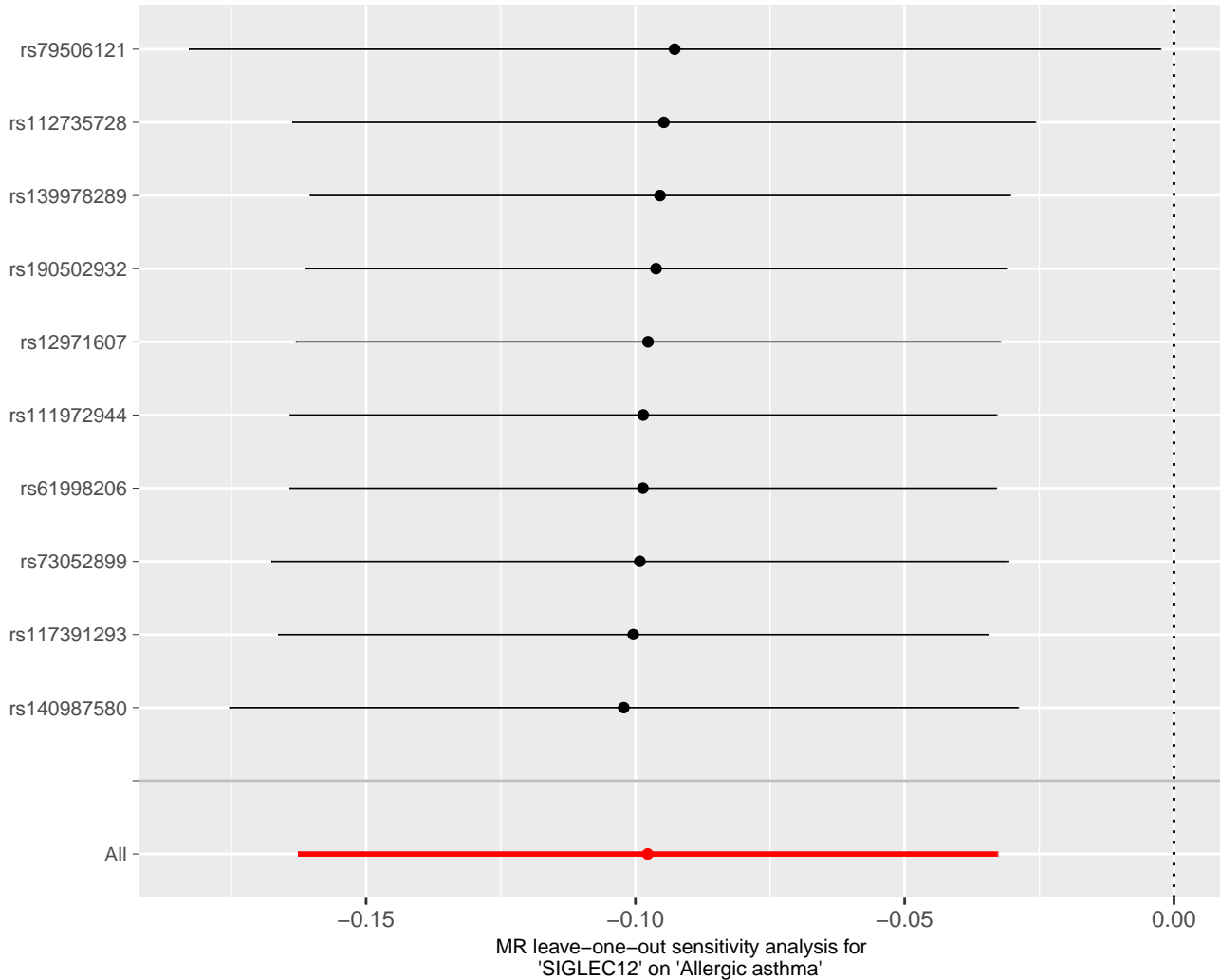

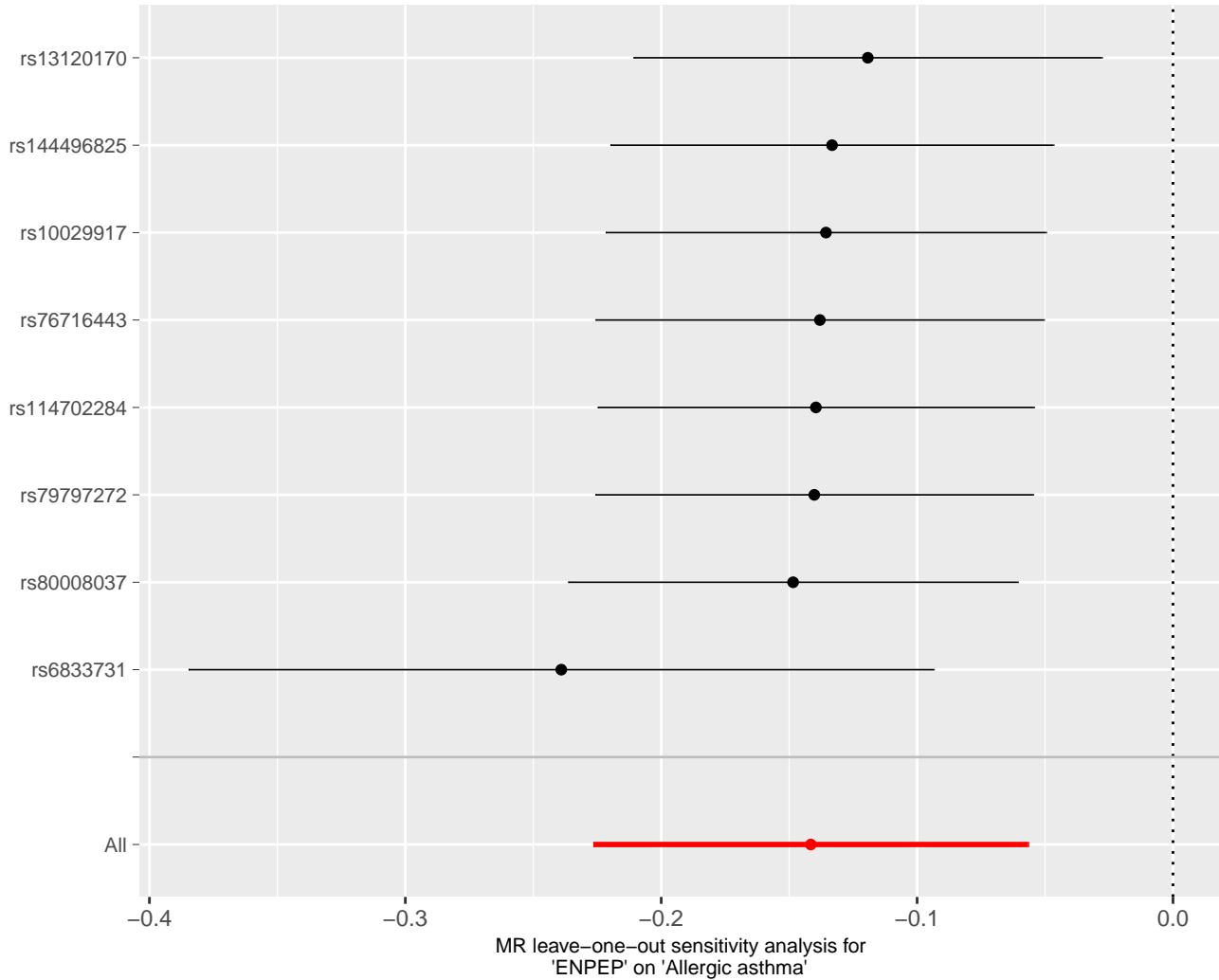

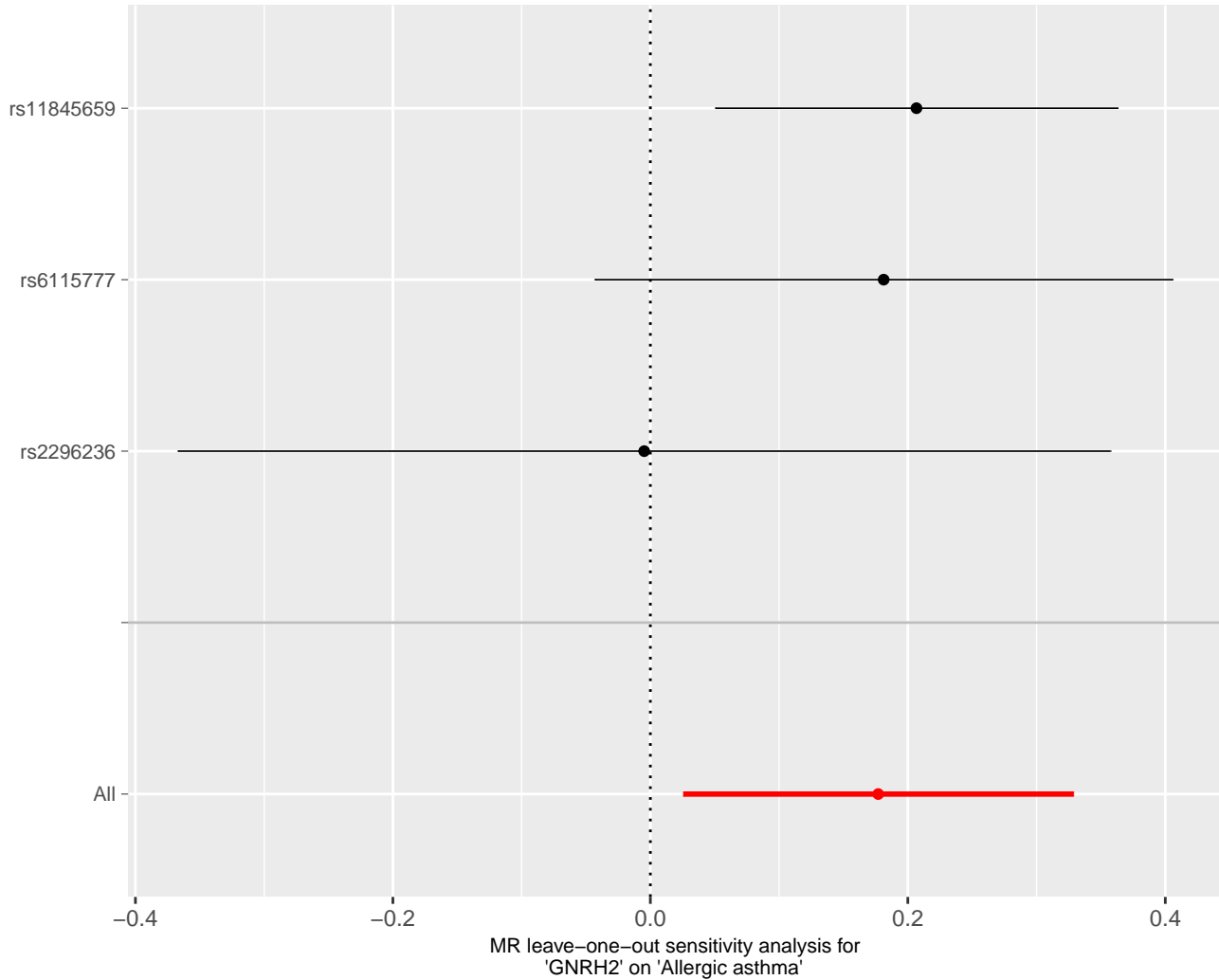

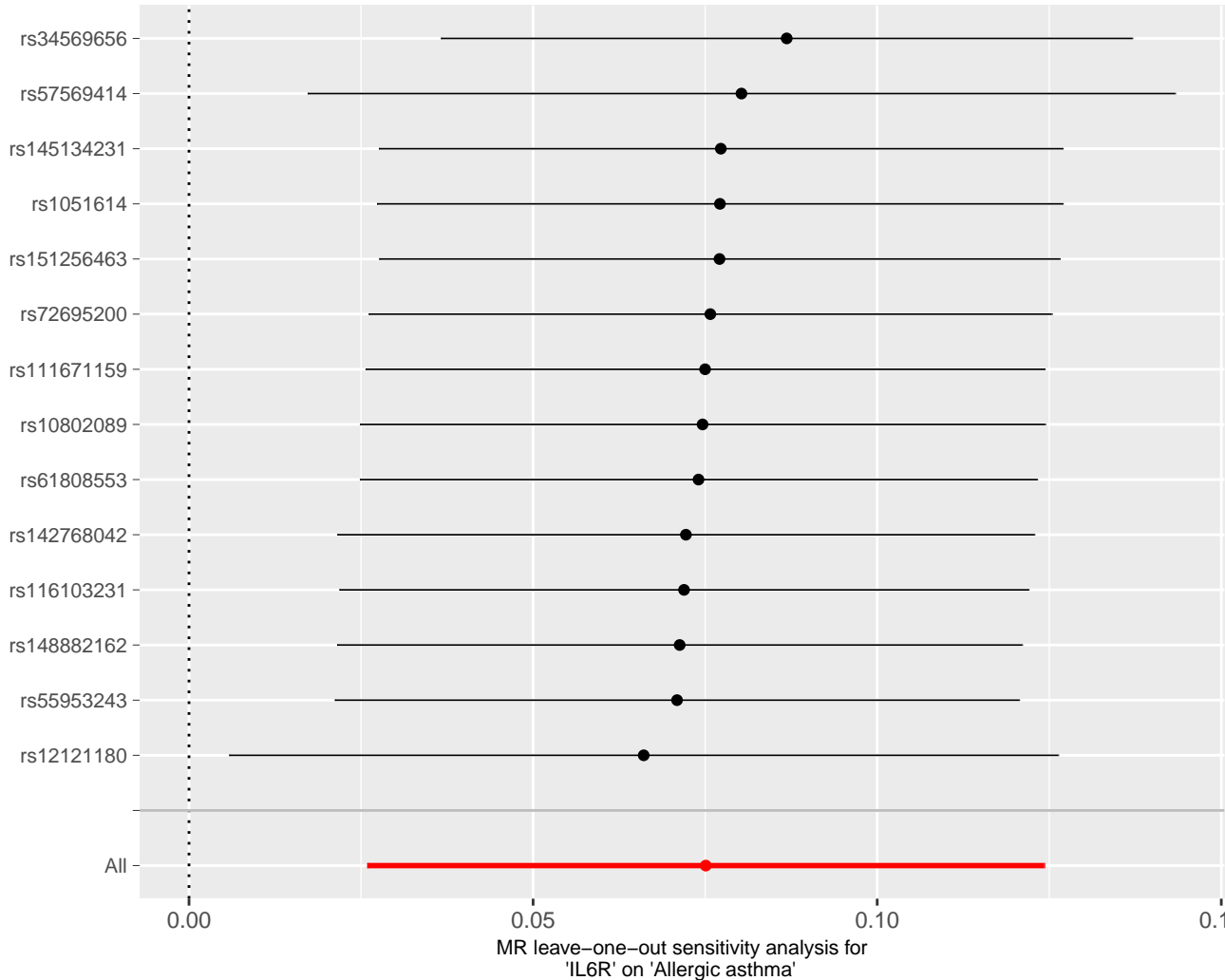

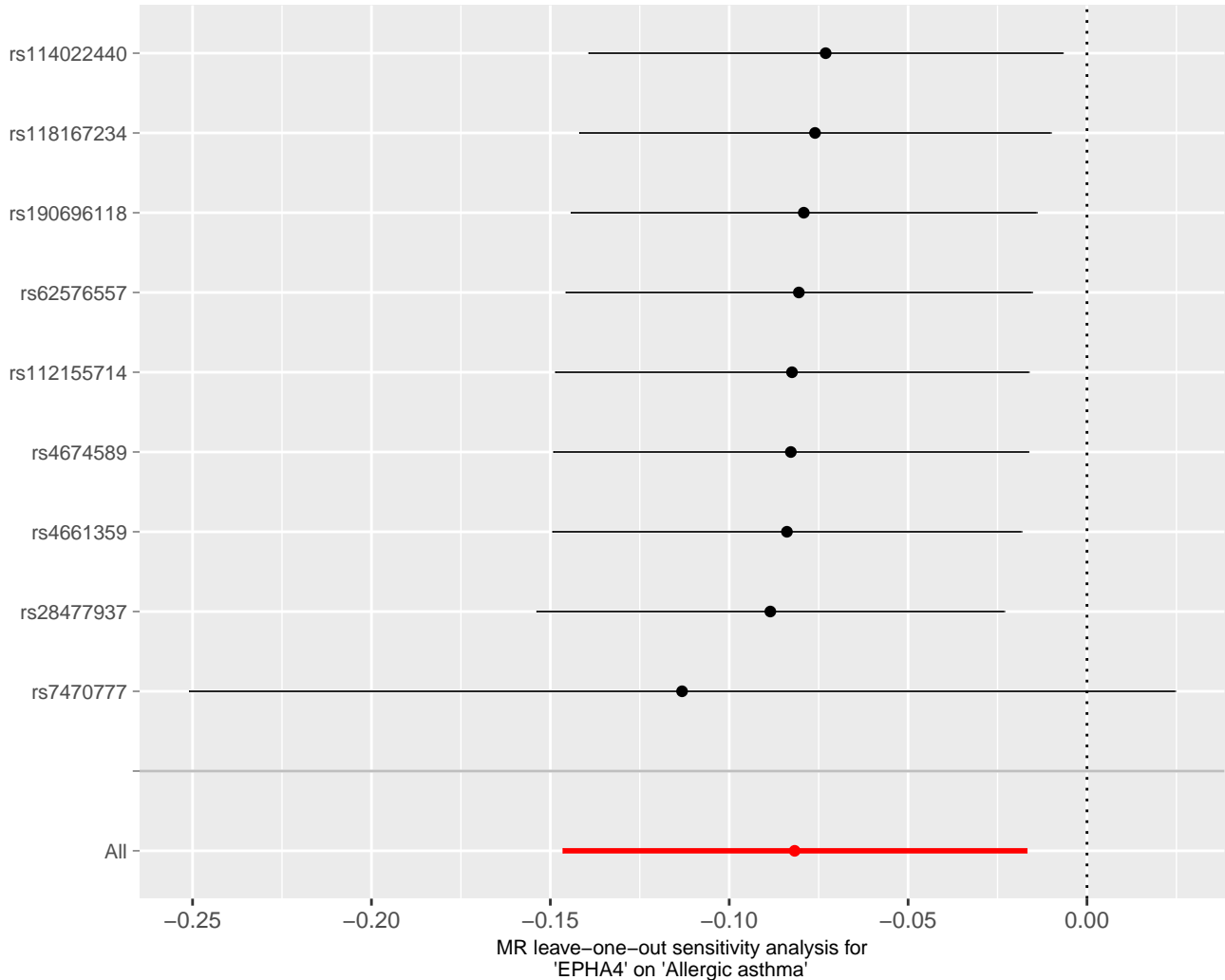

Supplement: Supplementary file 1 — Supplementary Material 1: Supplementary Materials 1. The MR results of BMI and allergic asthma. Supplementary Materials 2. The MR results of plasma proteins and allergic asthma. Supplementary Materials 3. The MR results of BMI and plasma proteins. Supplementary Materials 4. The MR results of mediation factor plasma proteins and allergic asthma. Supplementary Materials 5. The results of drug-targeted MR [file 41065_2025_376_MOESM1_ESM.zip › Supplementary Materials/S2 The MR results of plasma proteins and allergic asthma/S2 plasma proteins-Allergic asthma.leaveoneout.pdf]

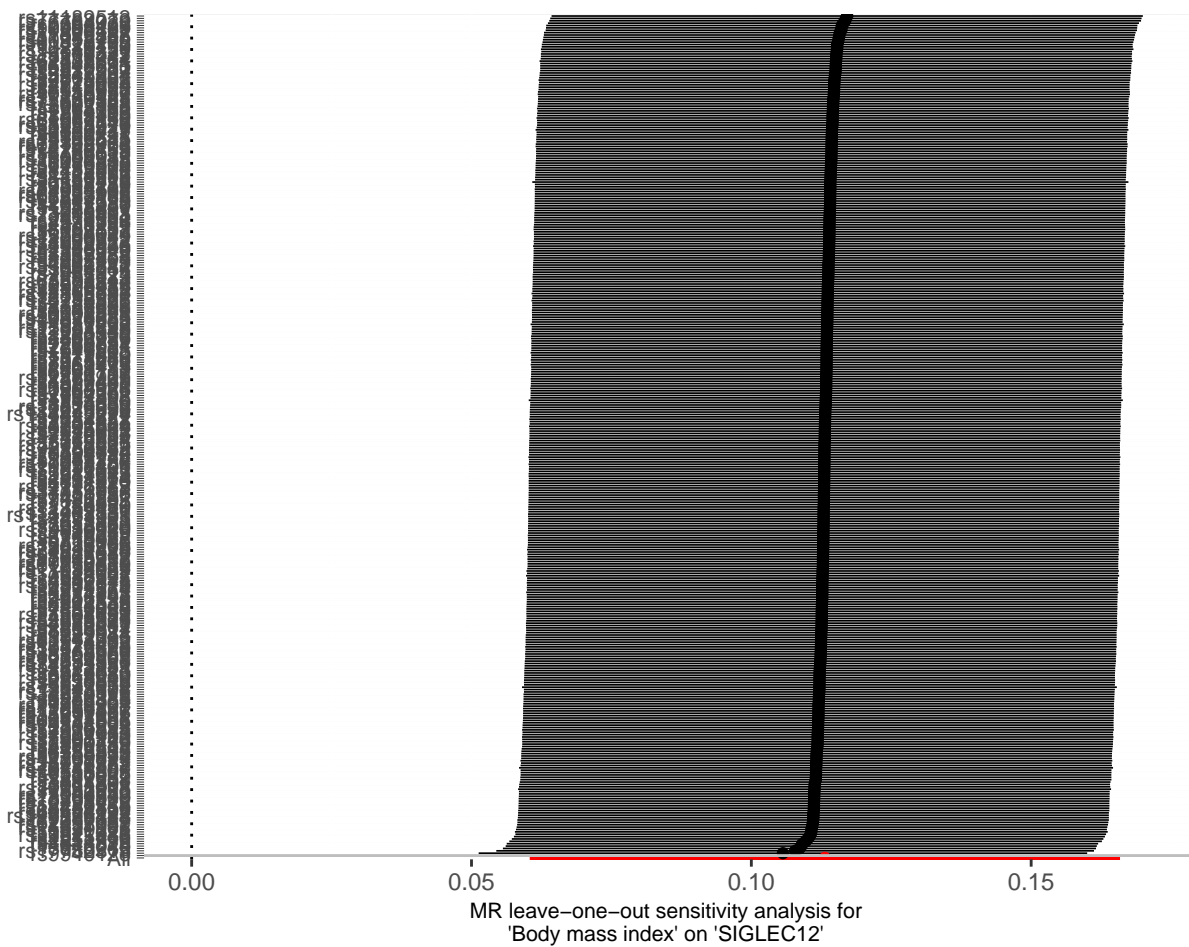

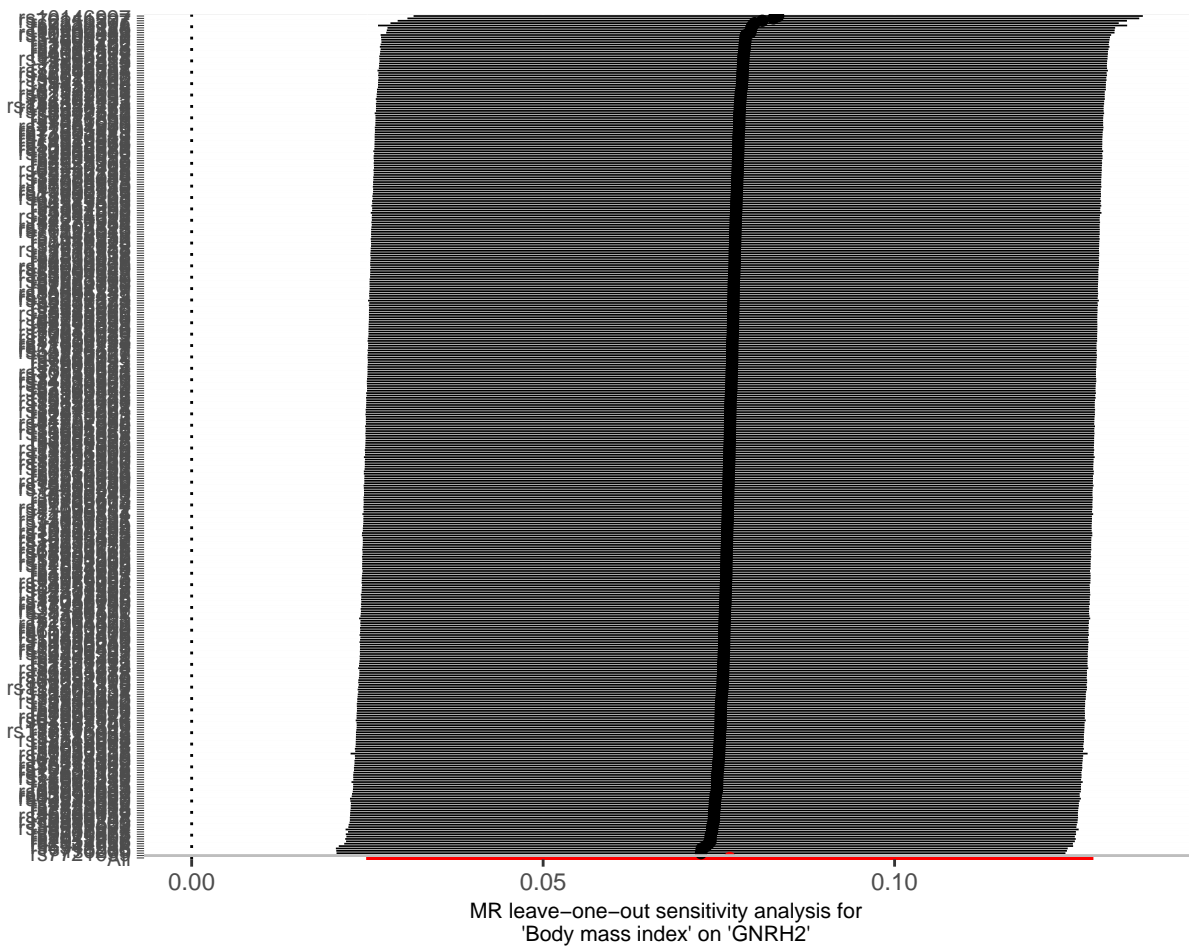

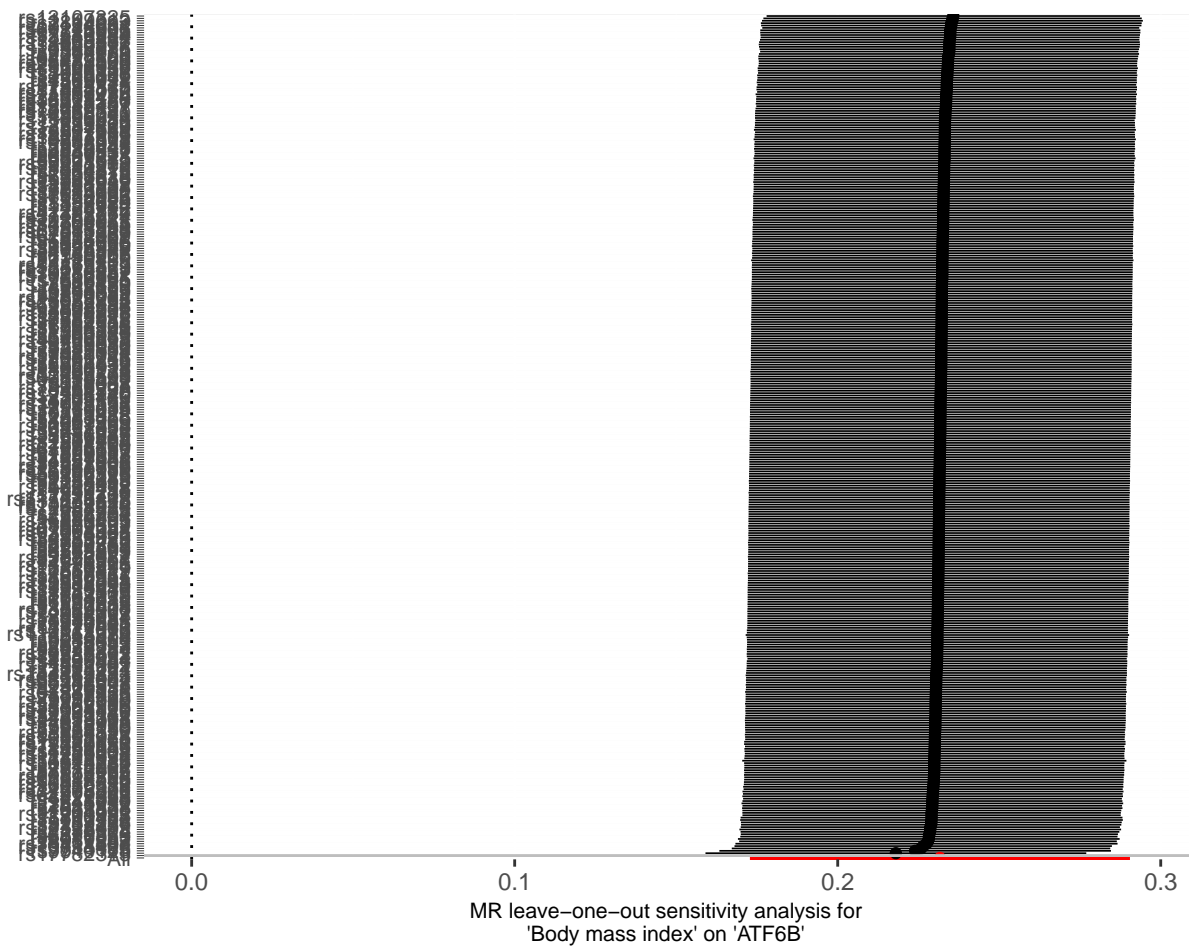

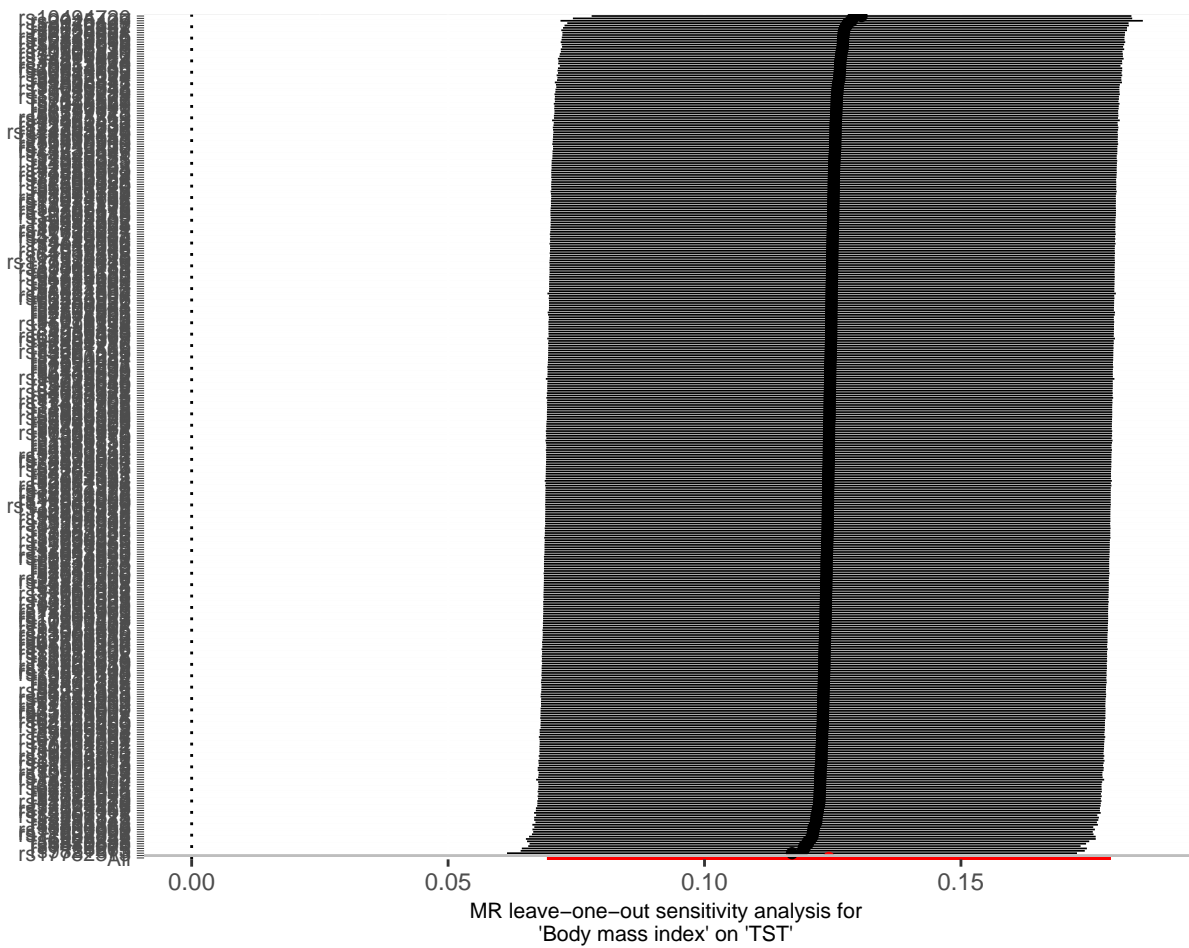

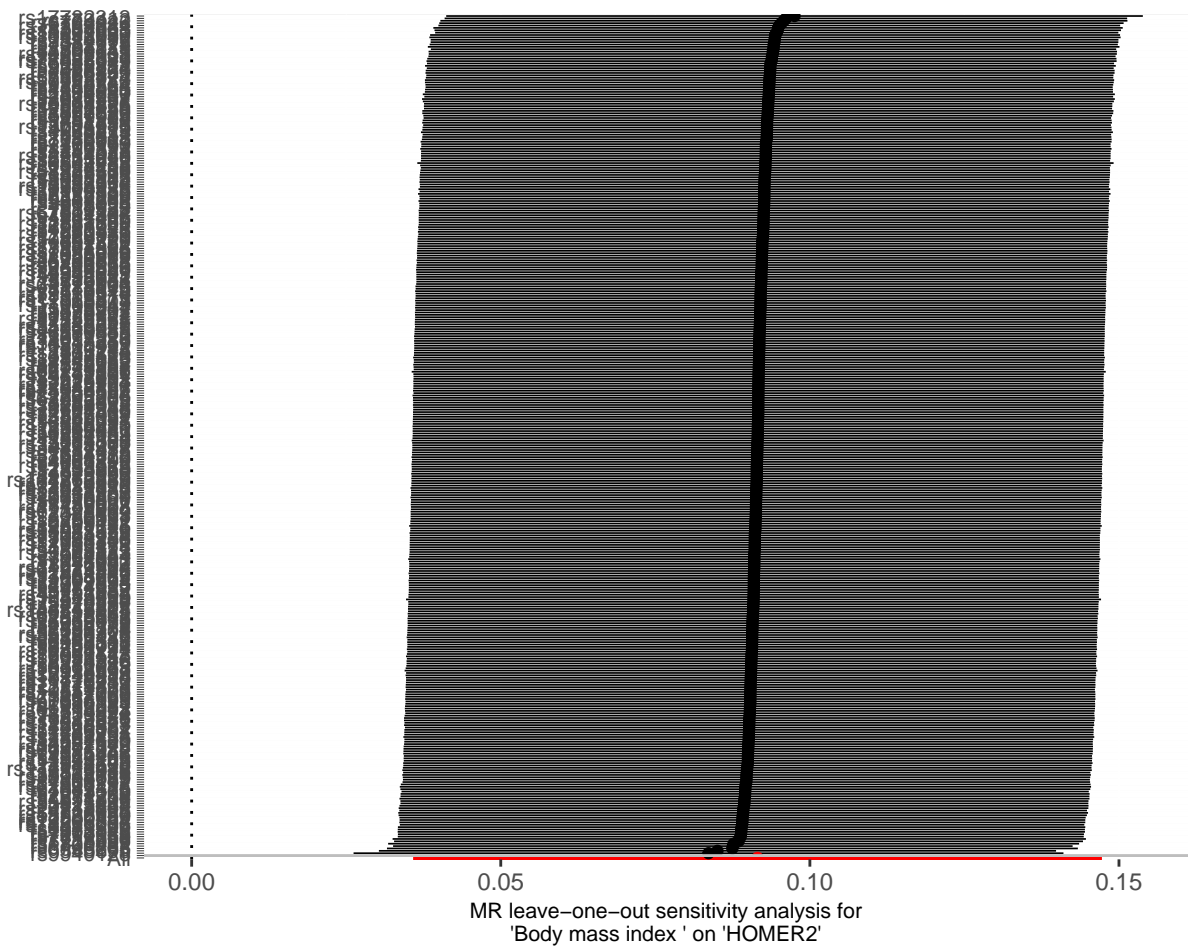

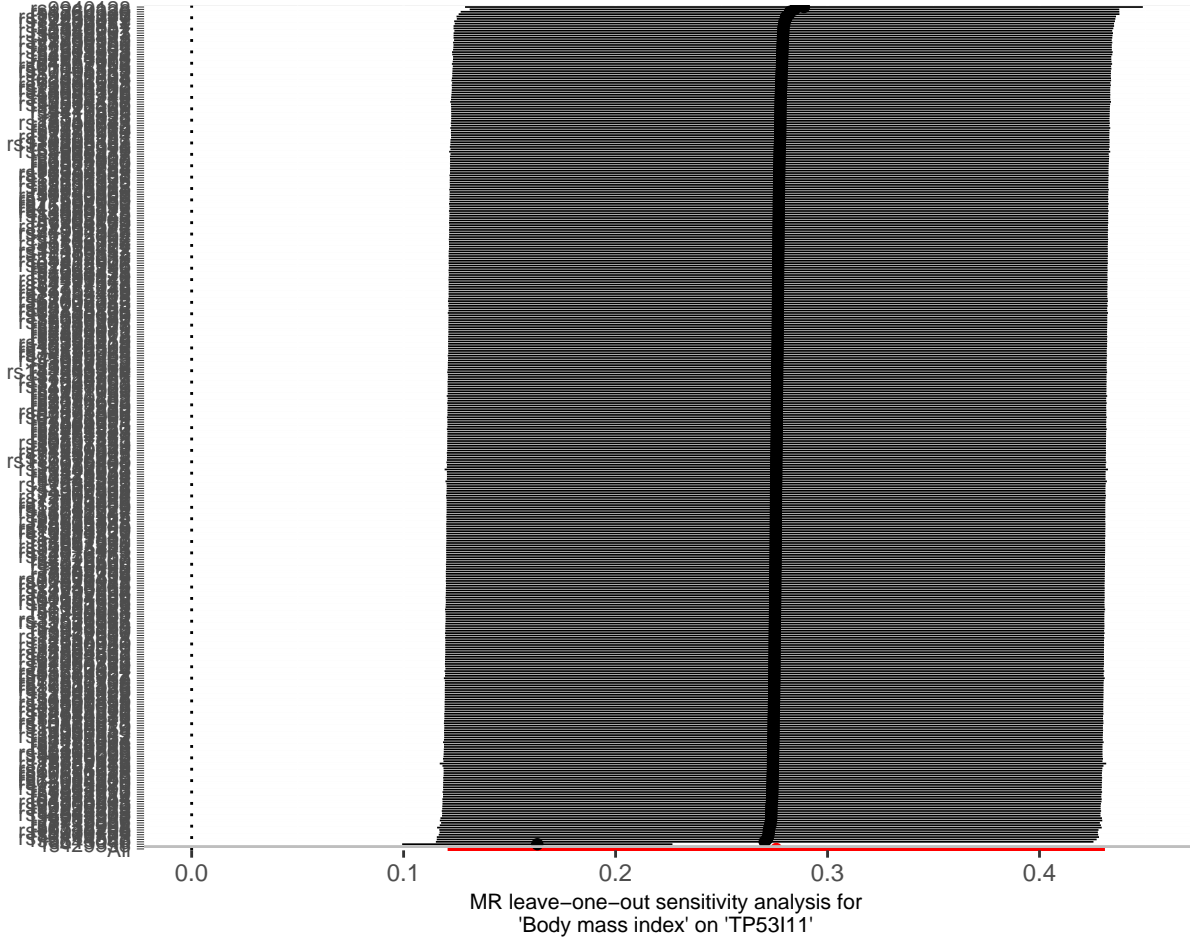

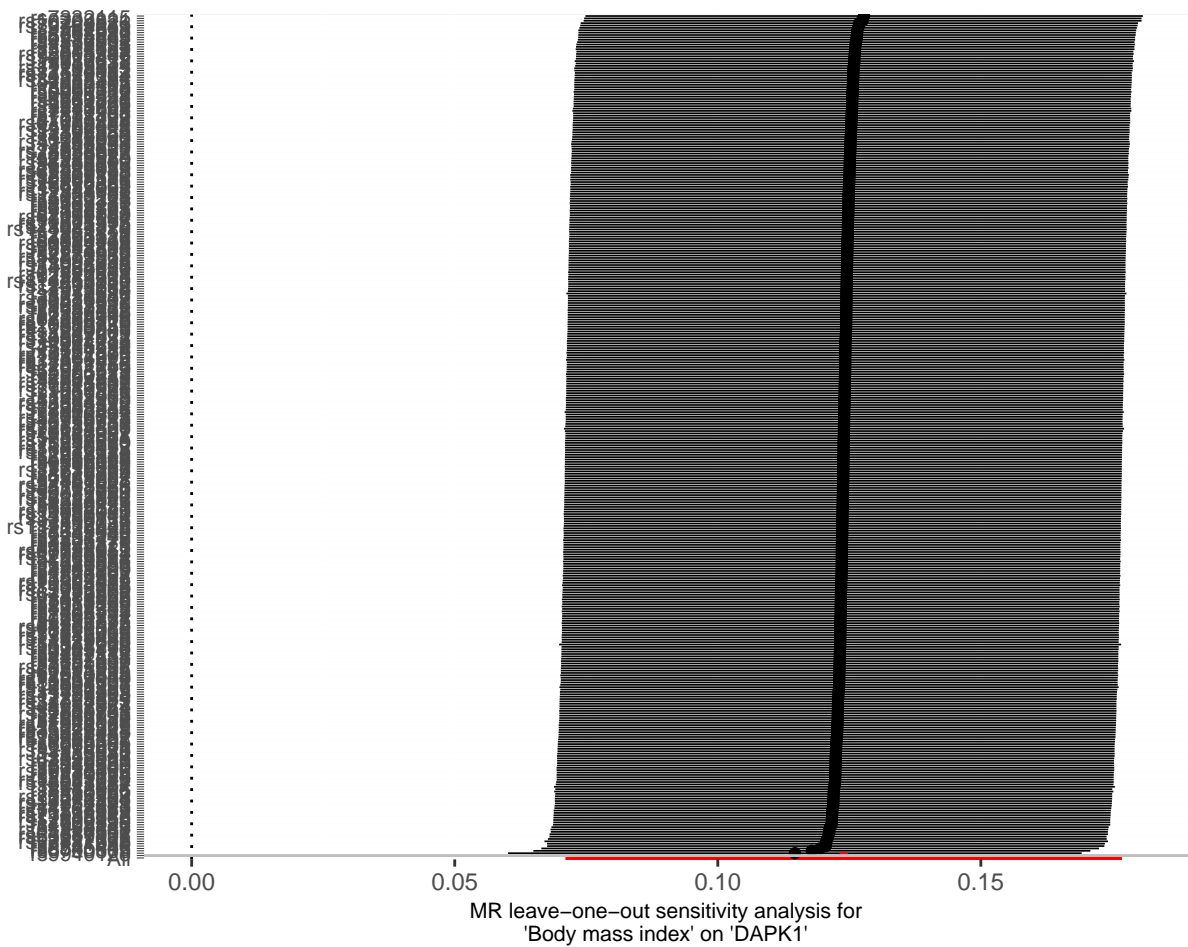

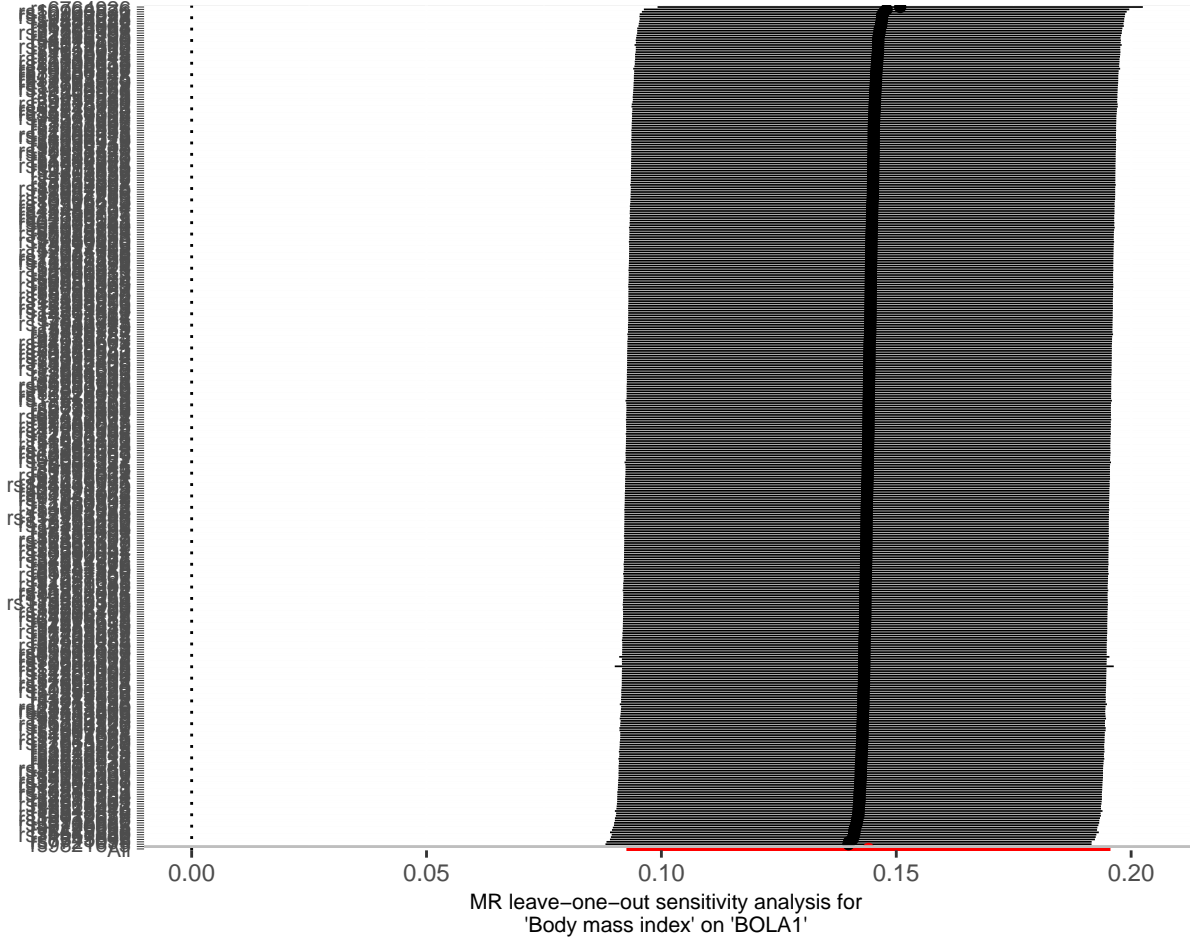

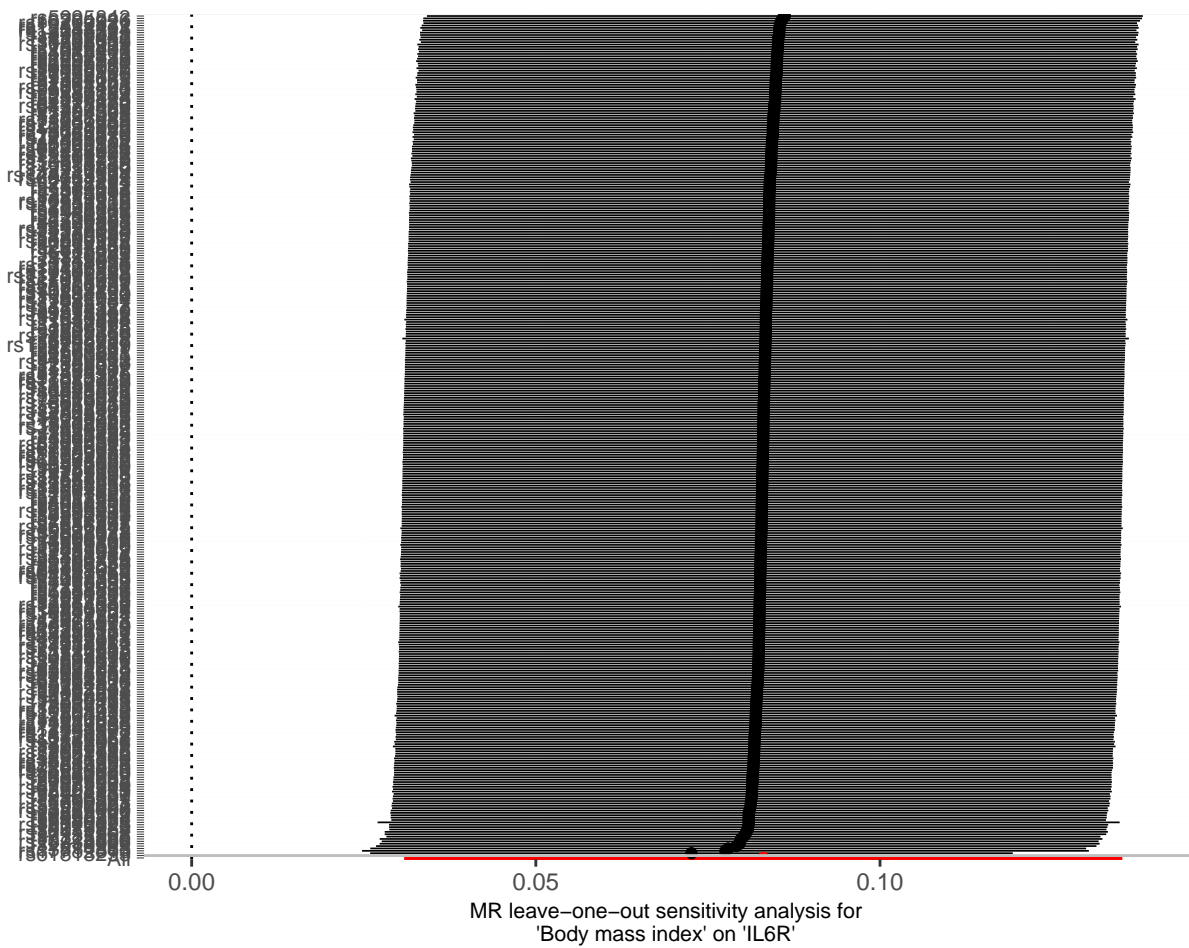

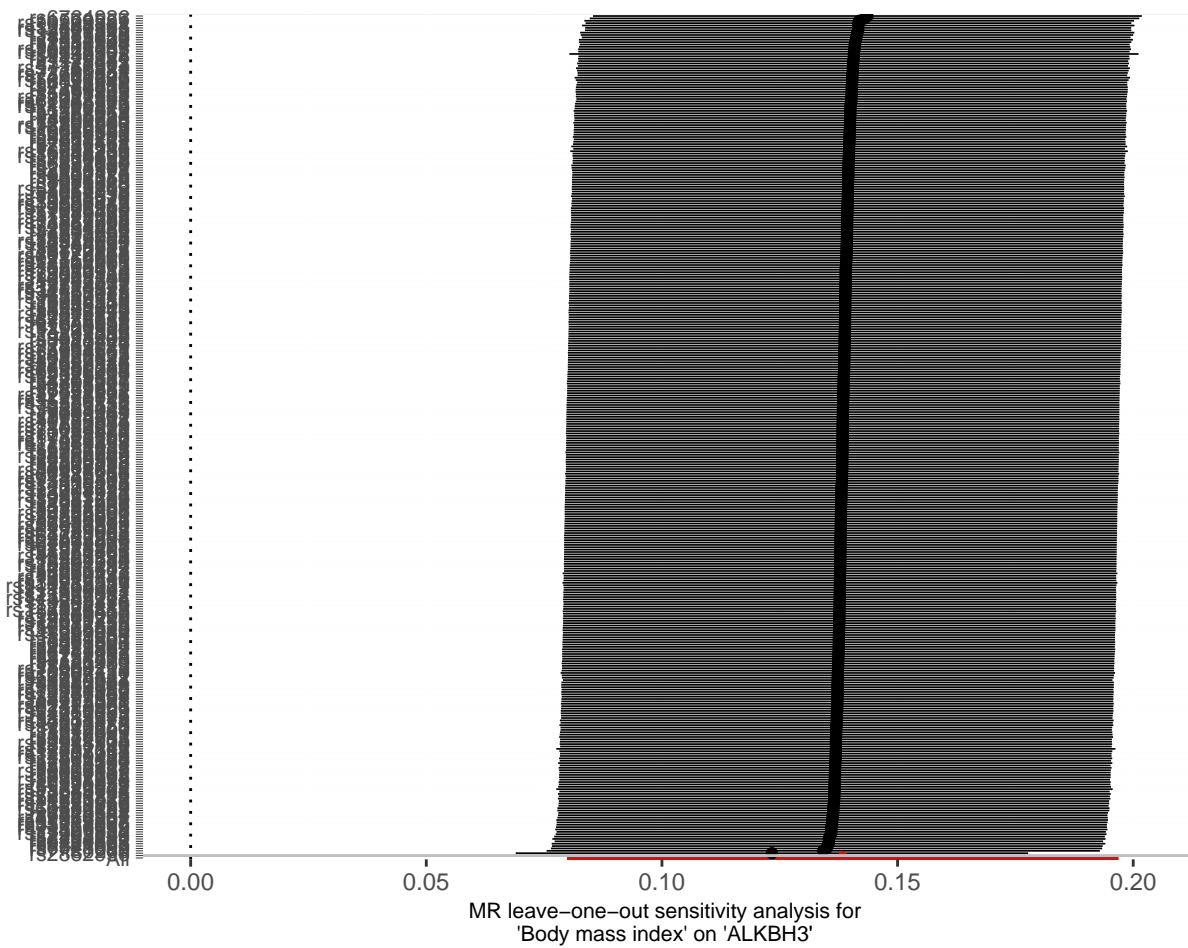

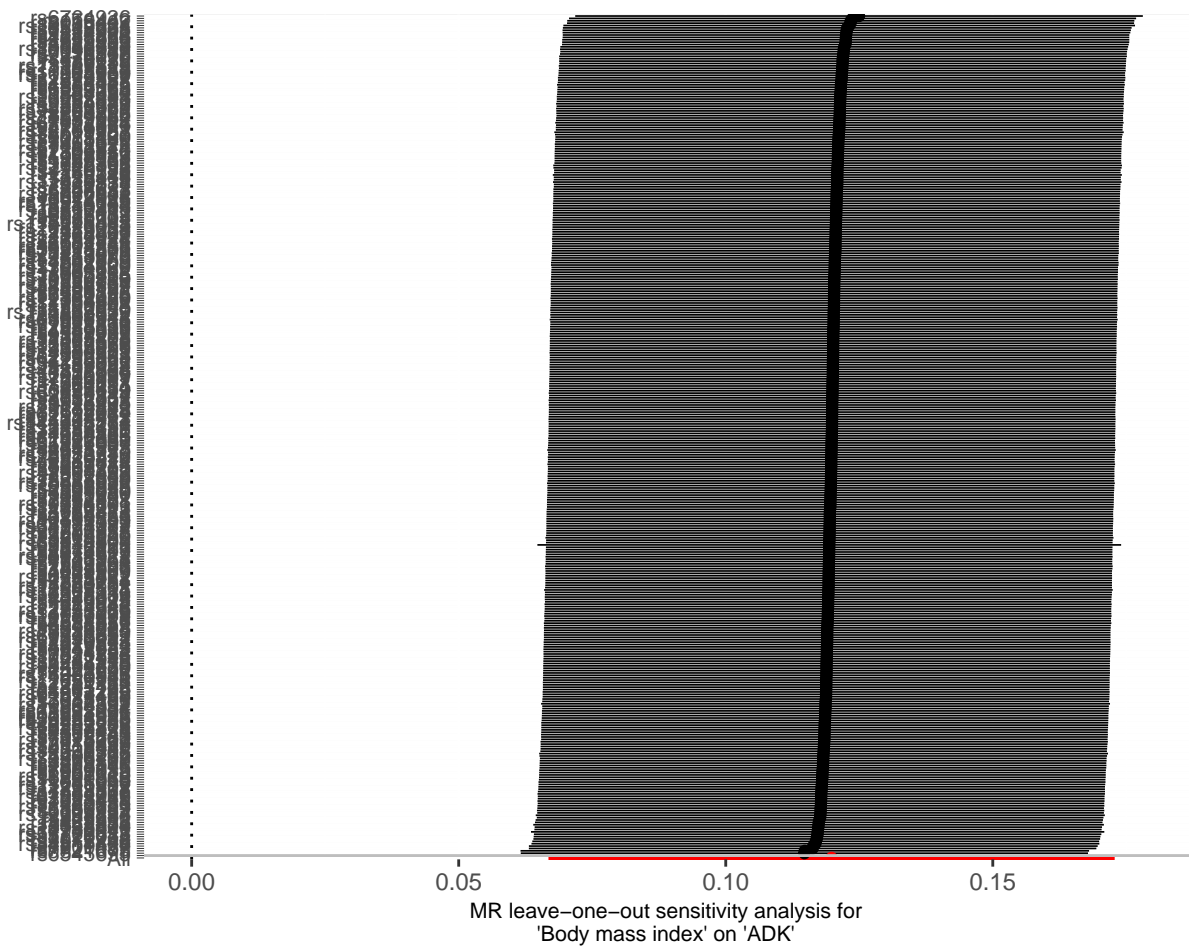

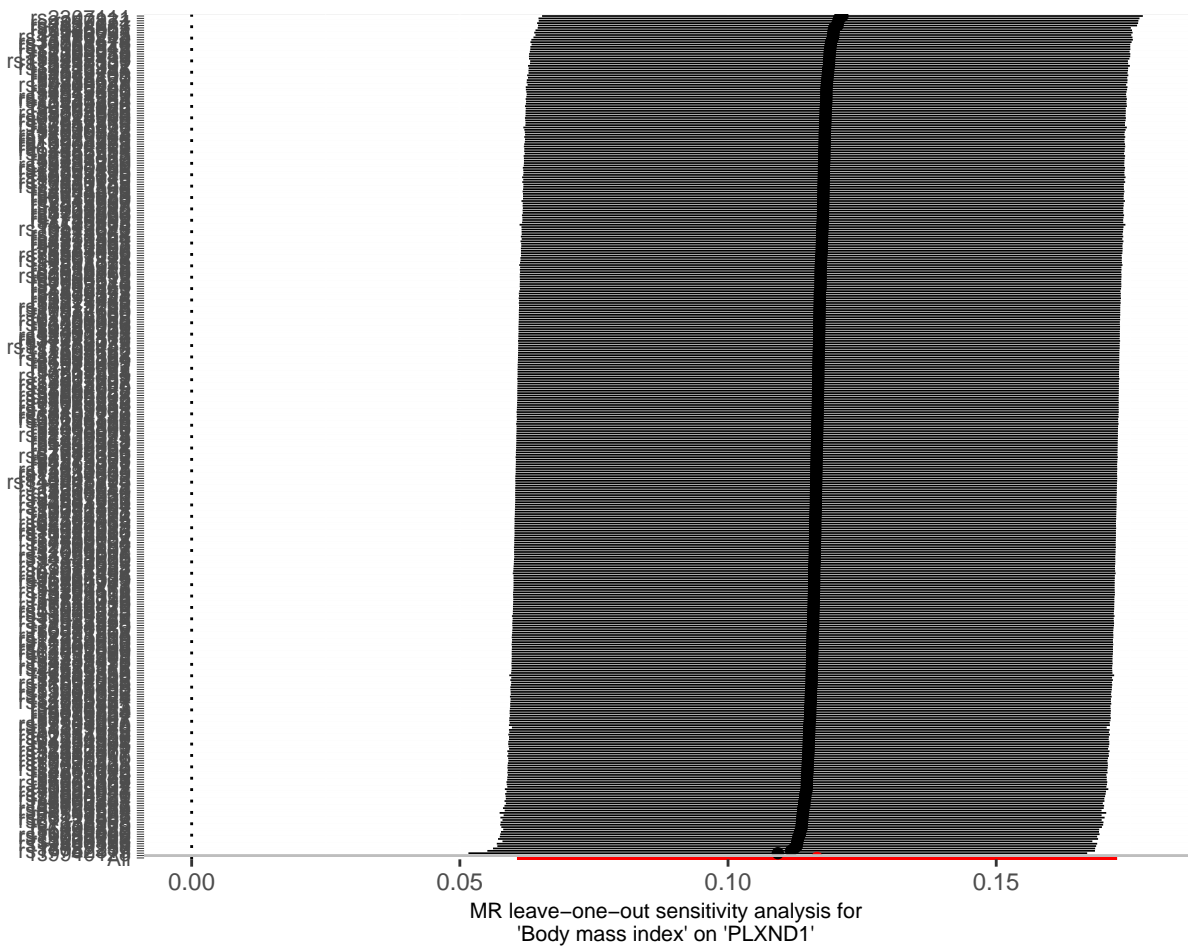

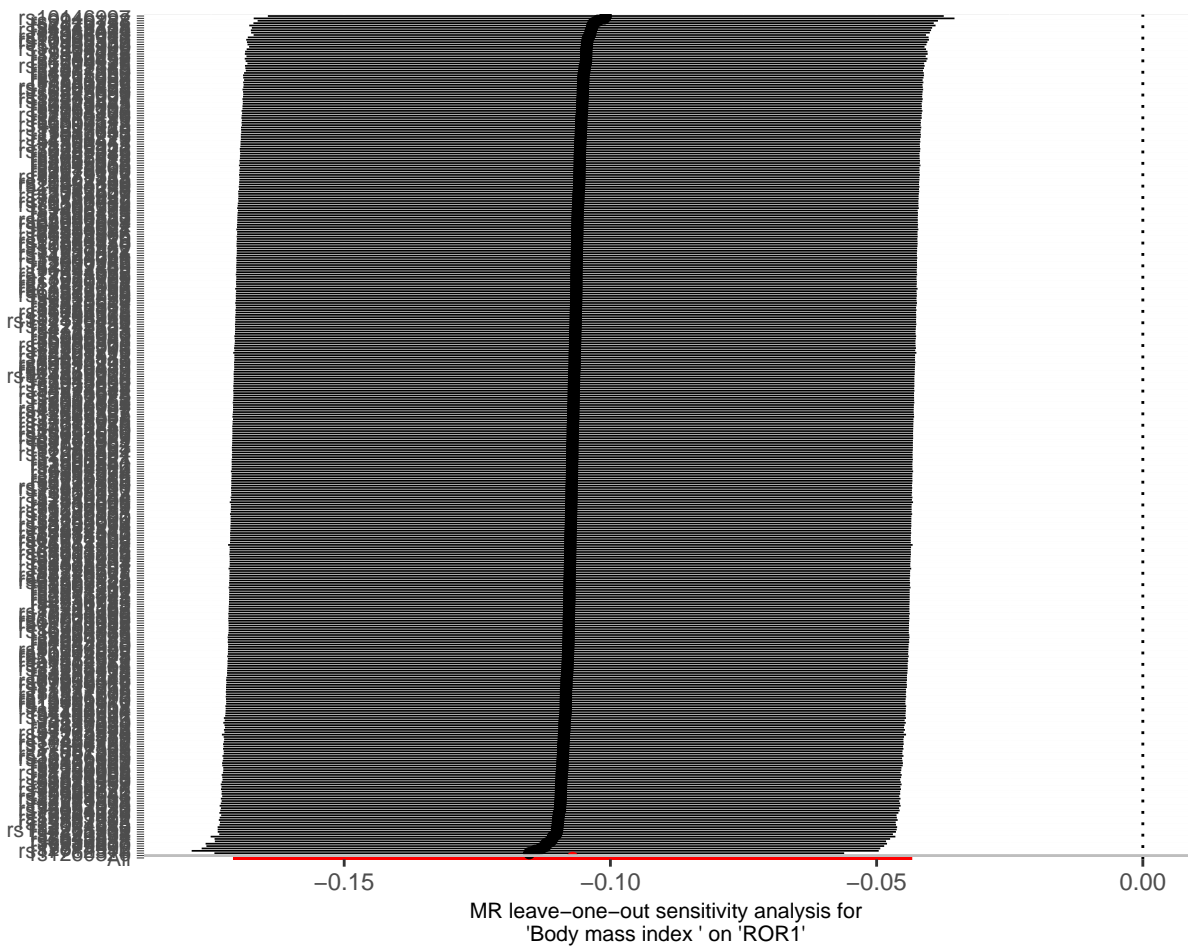

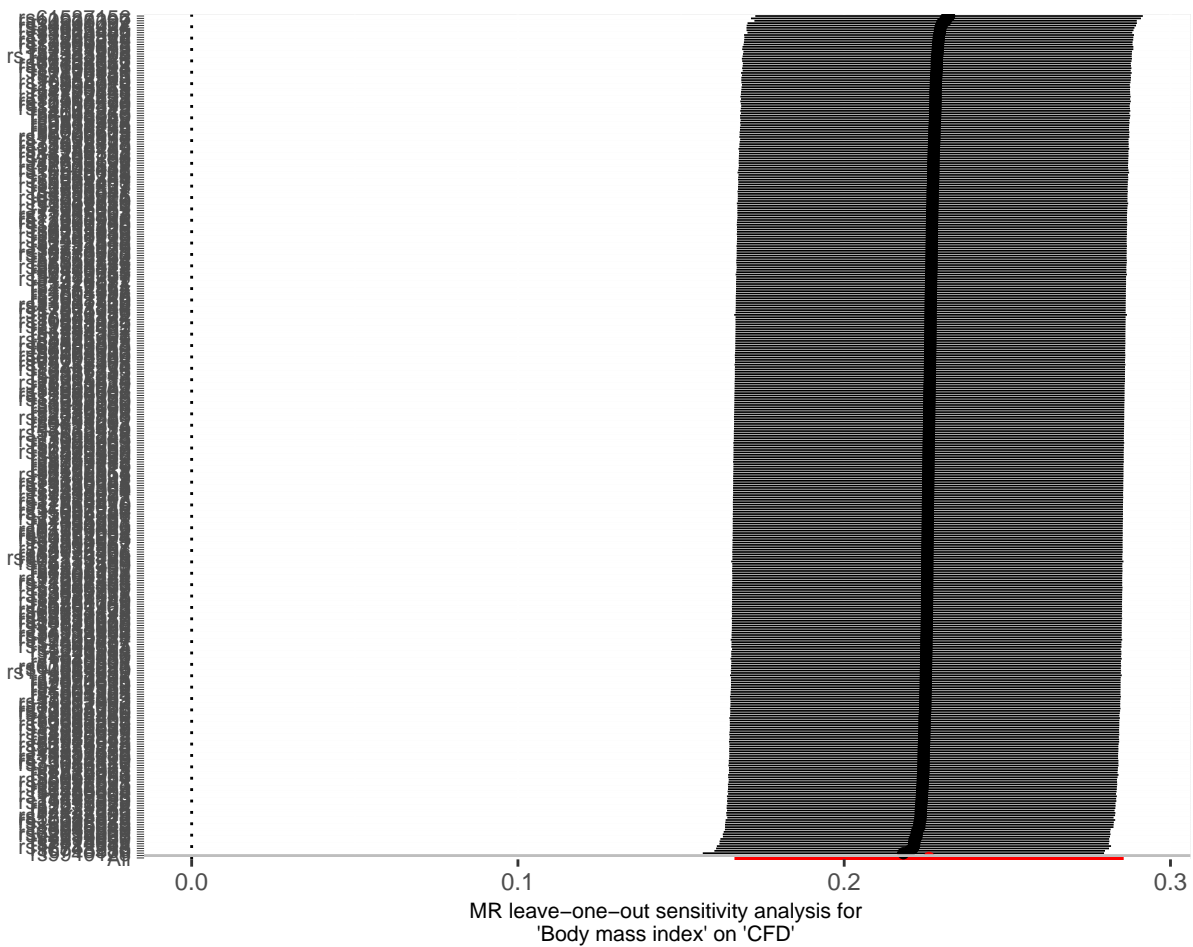

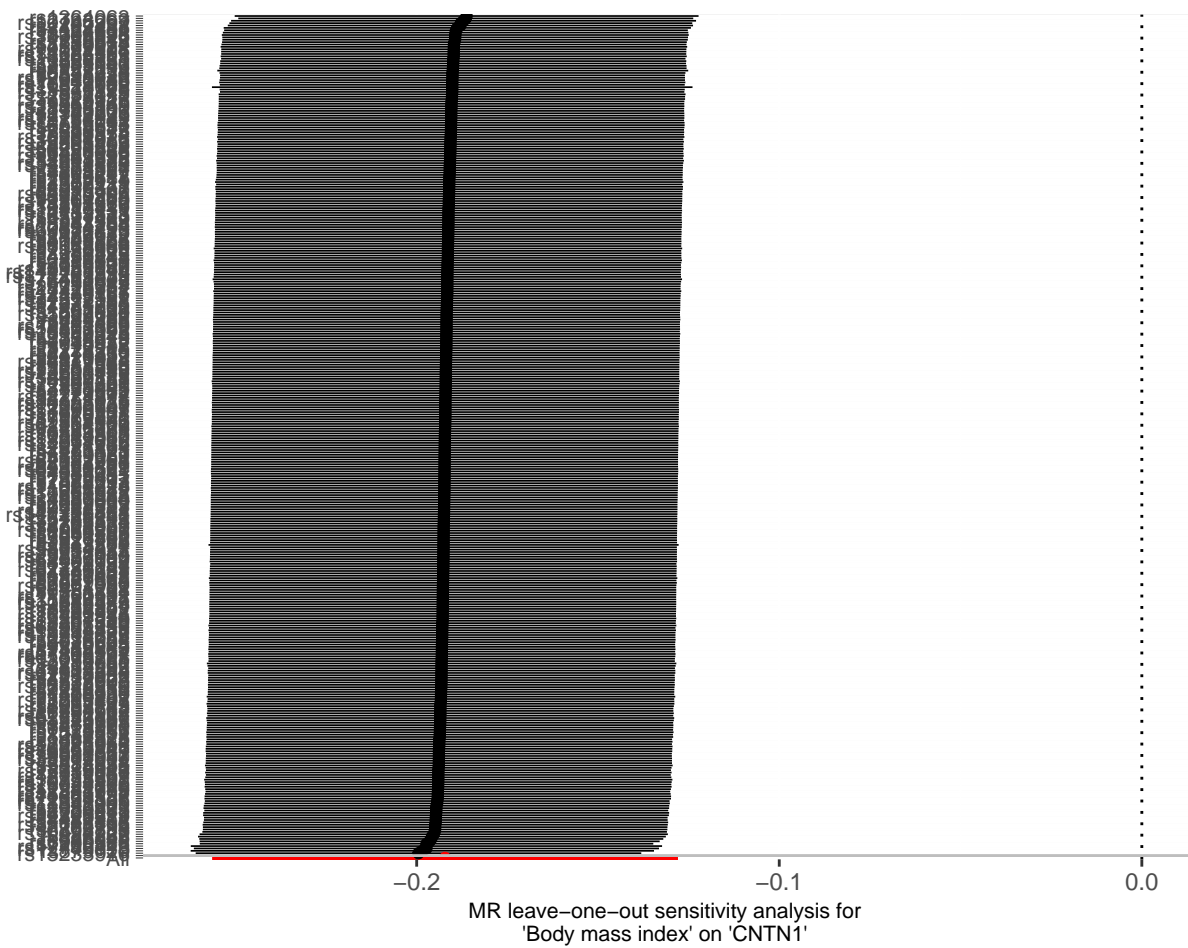

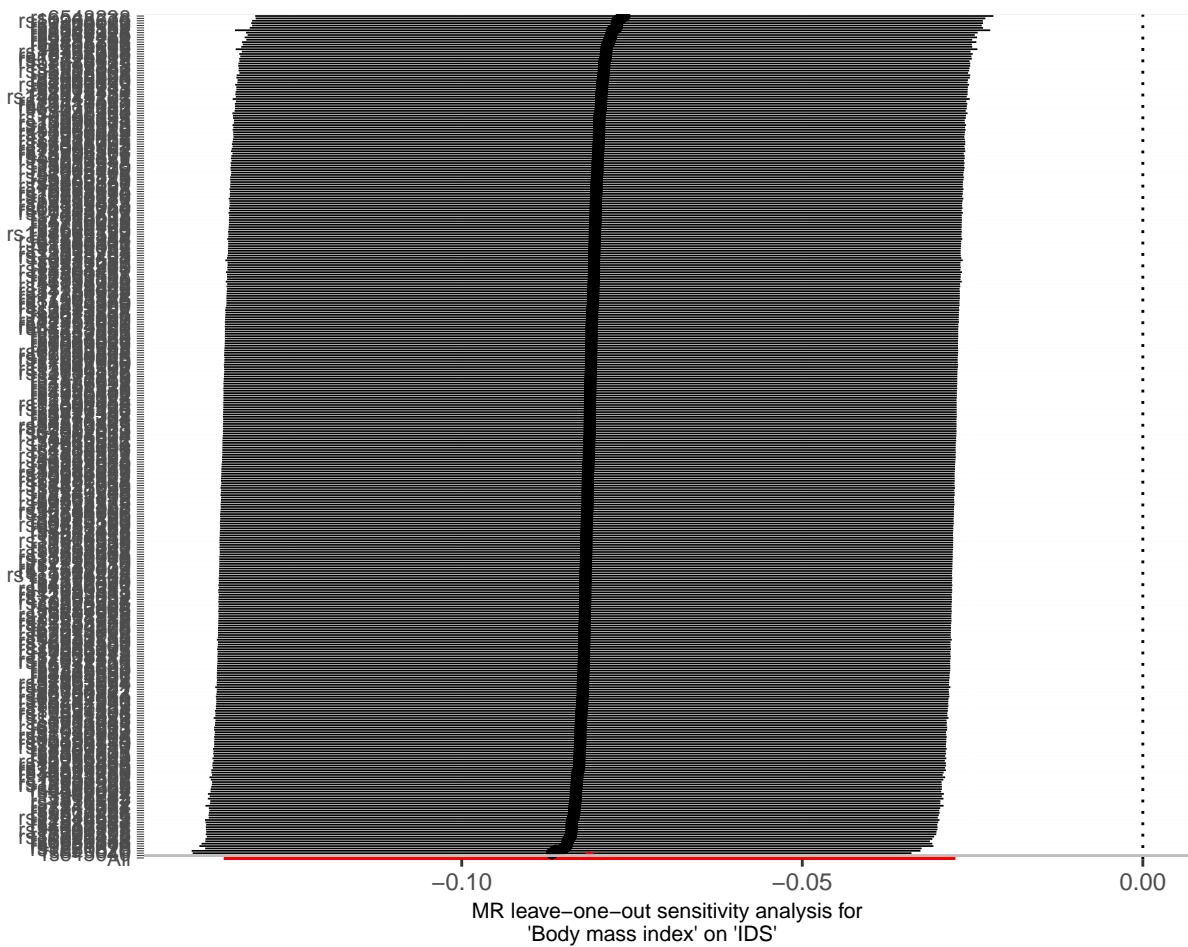

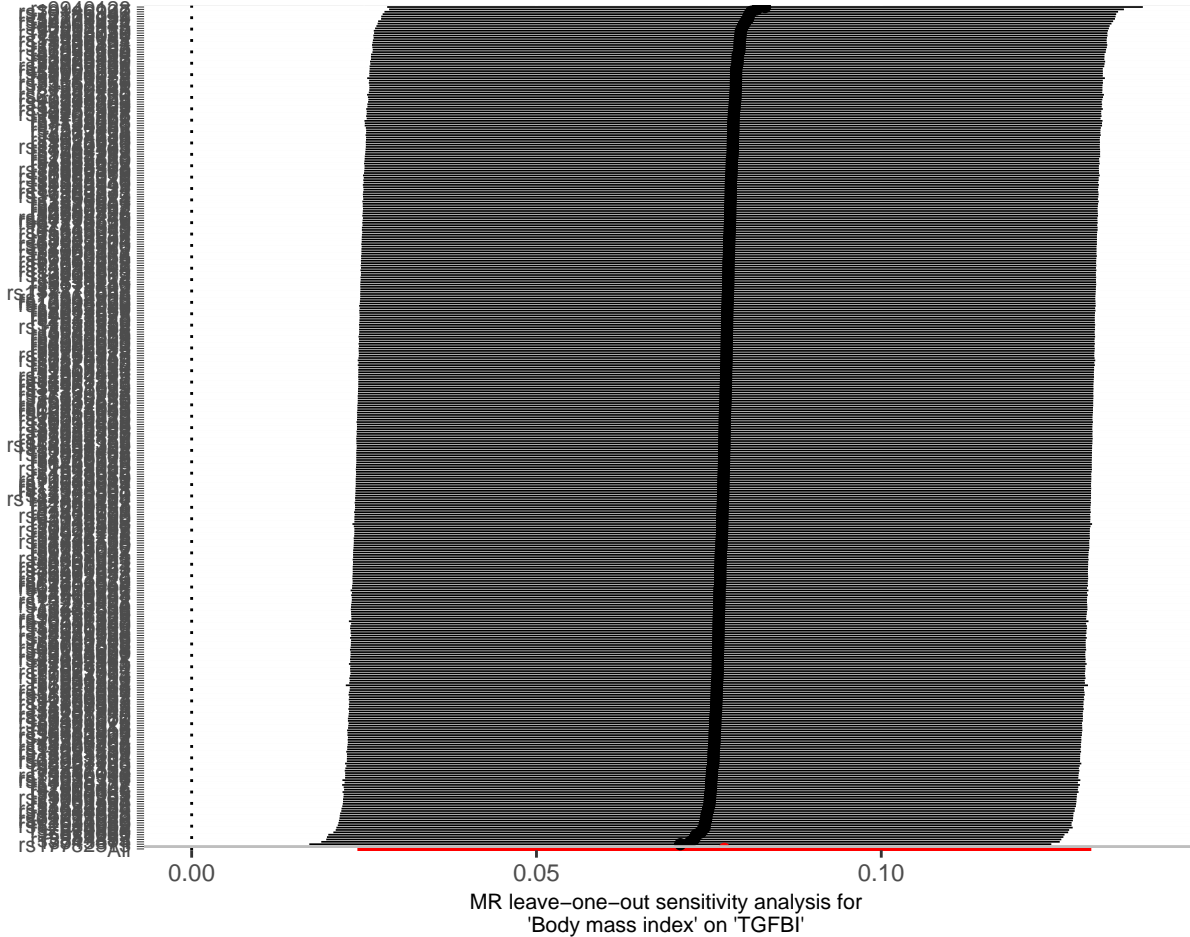

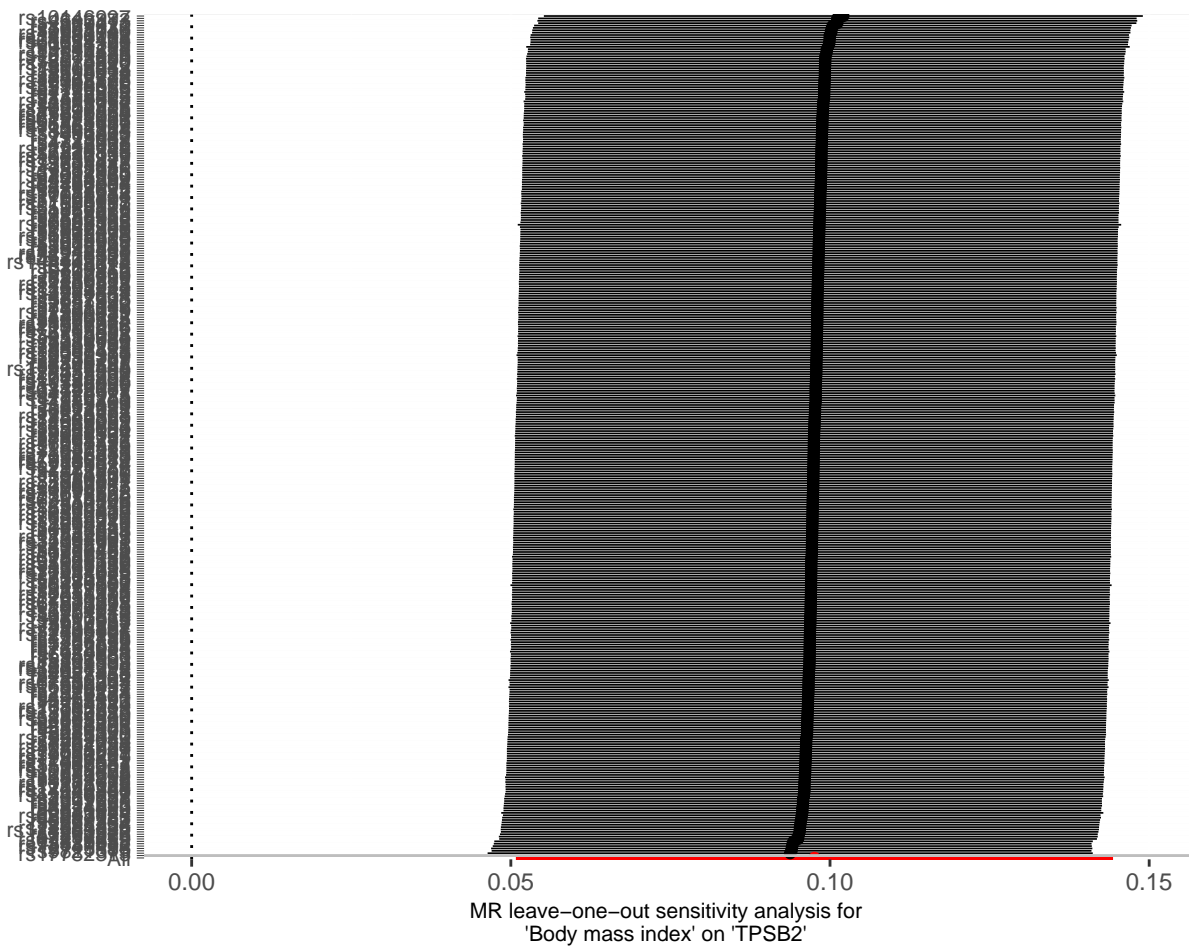

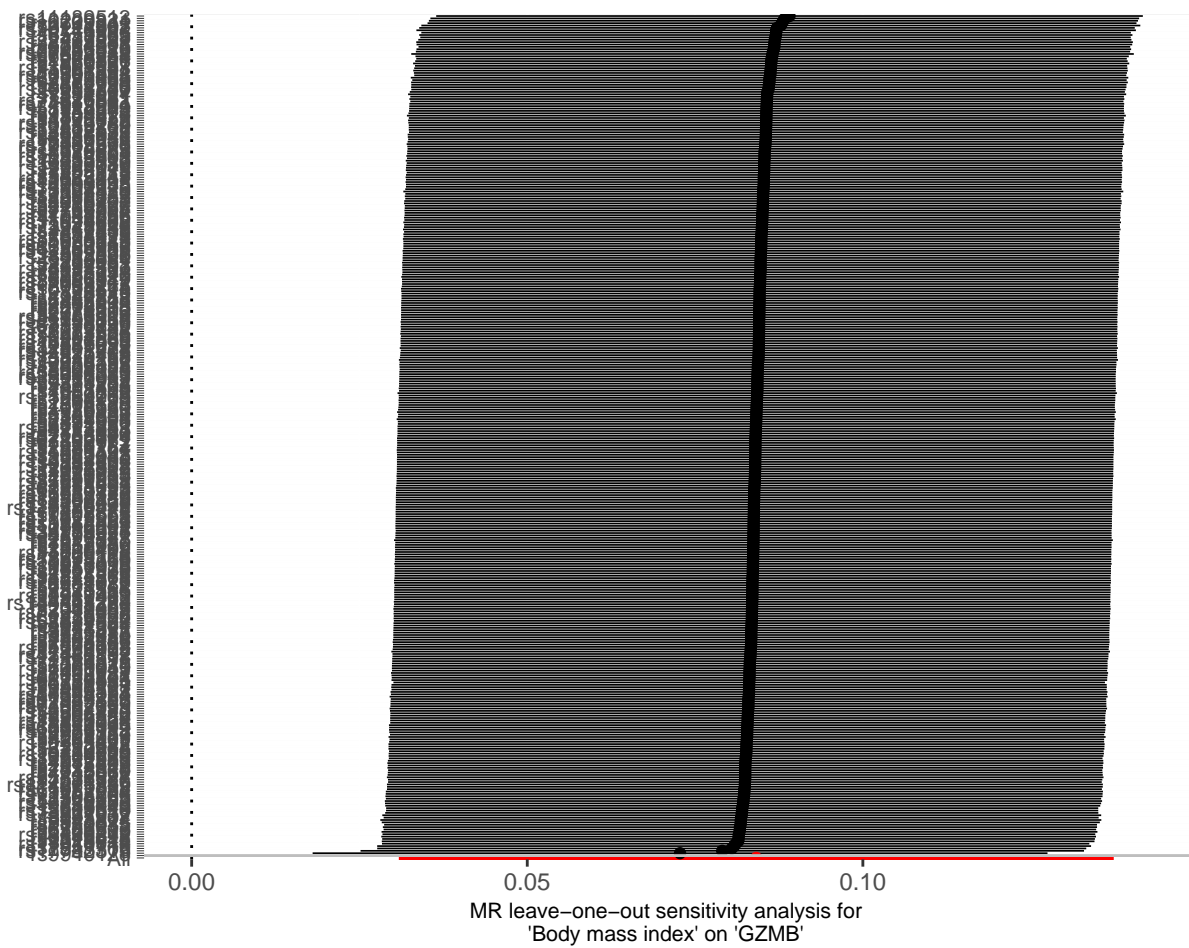

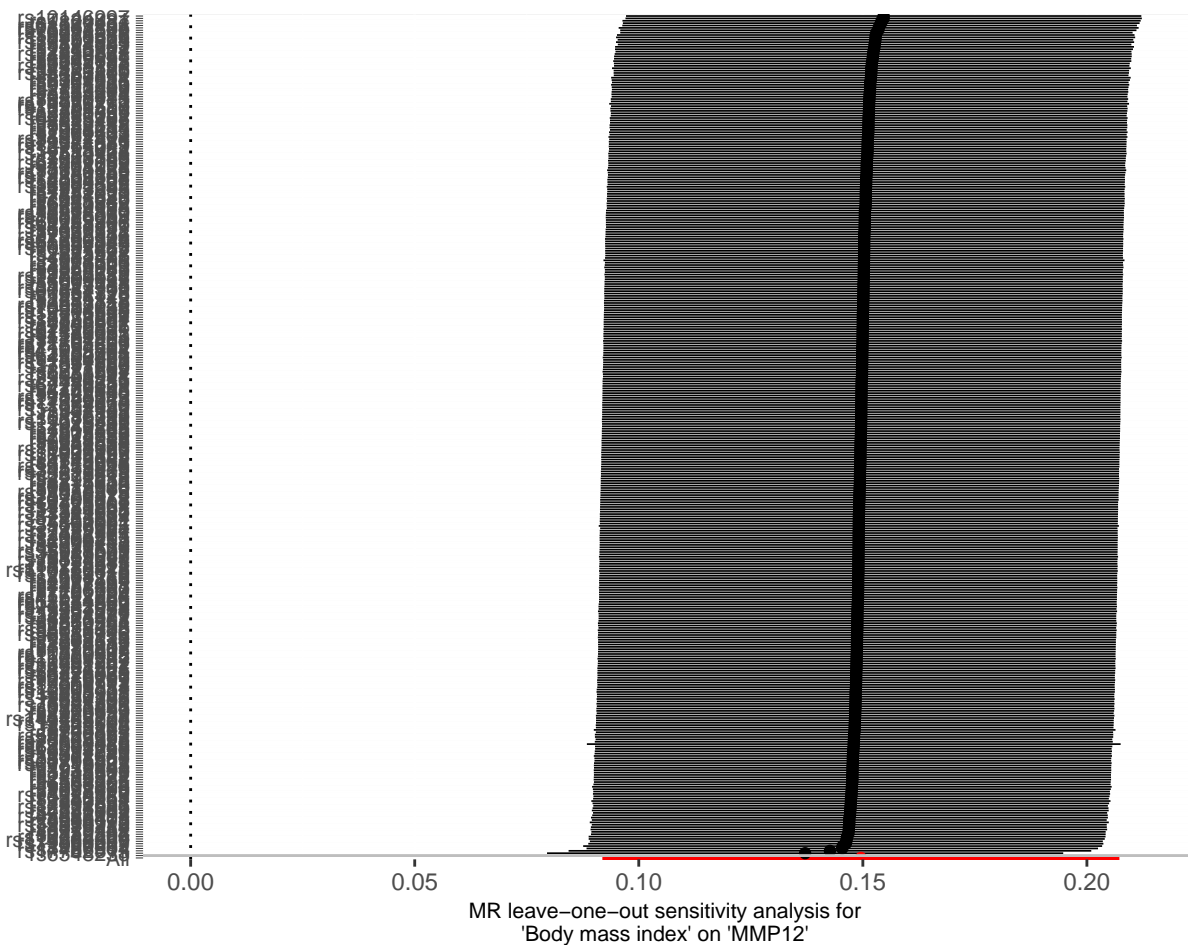

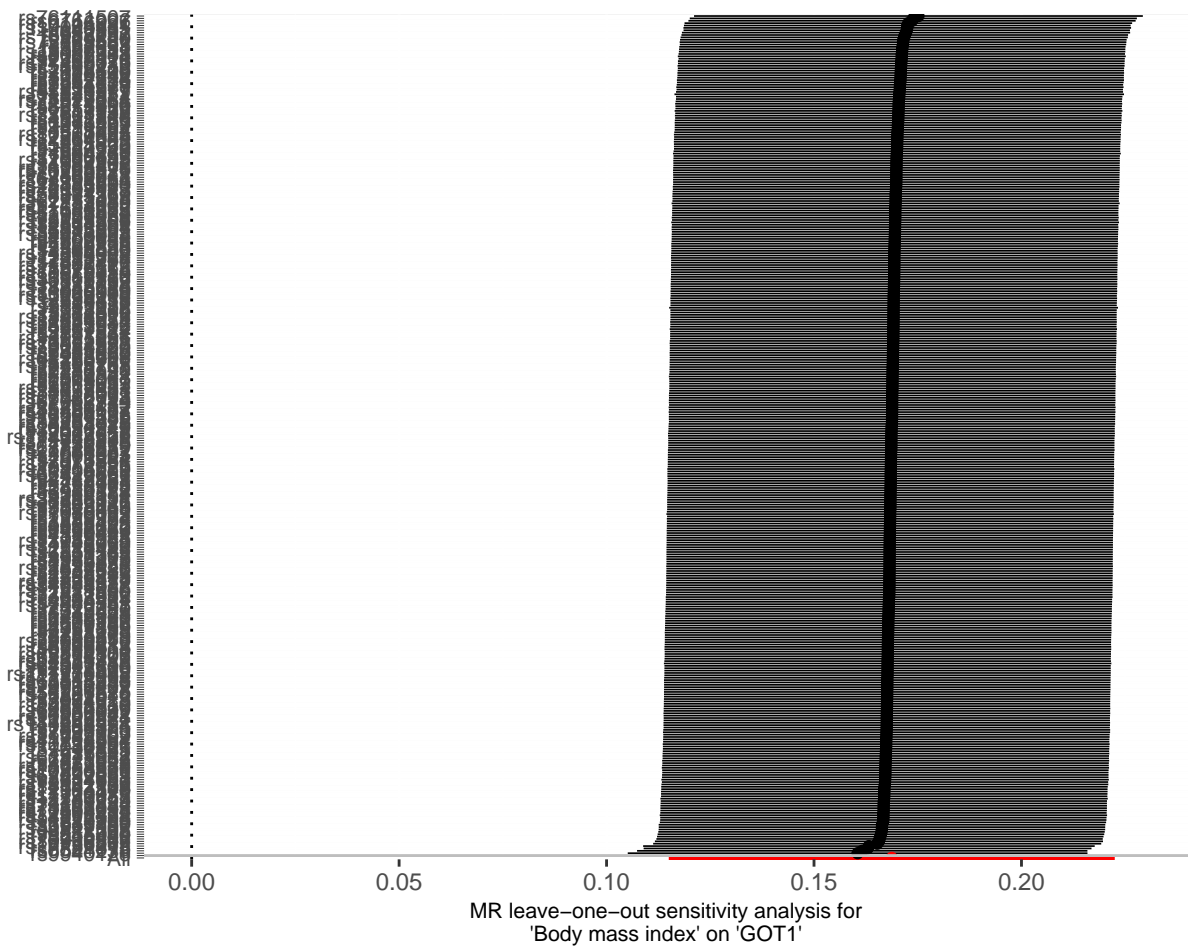

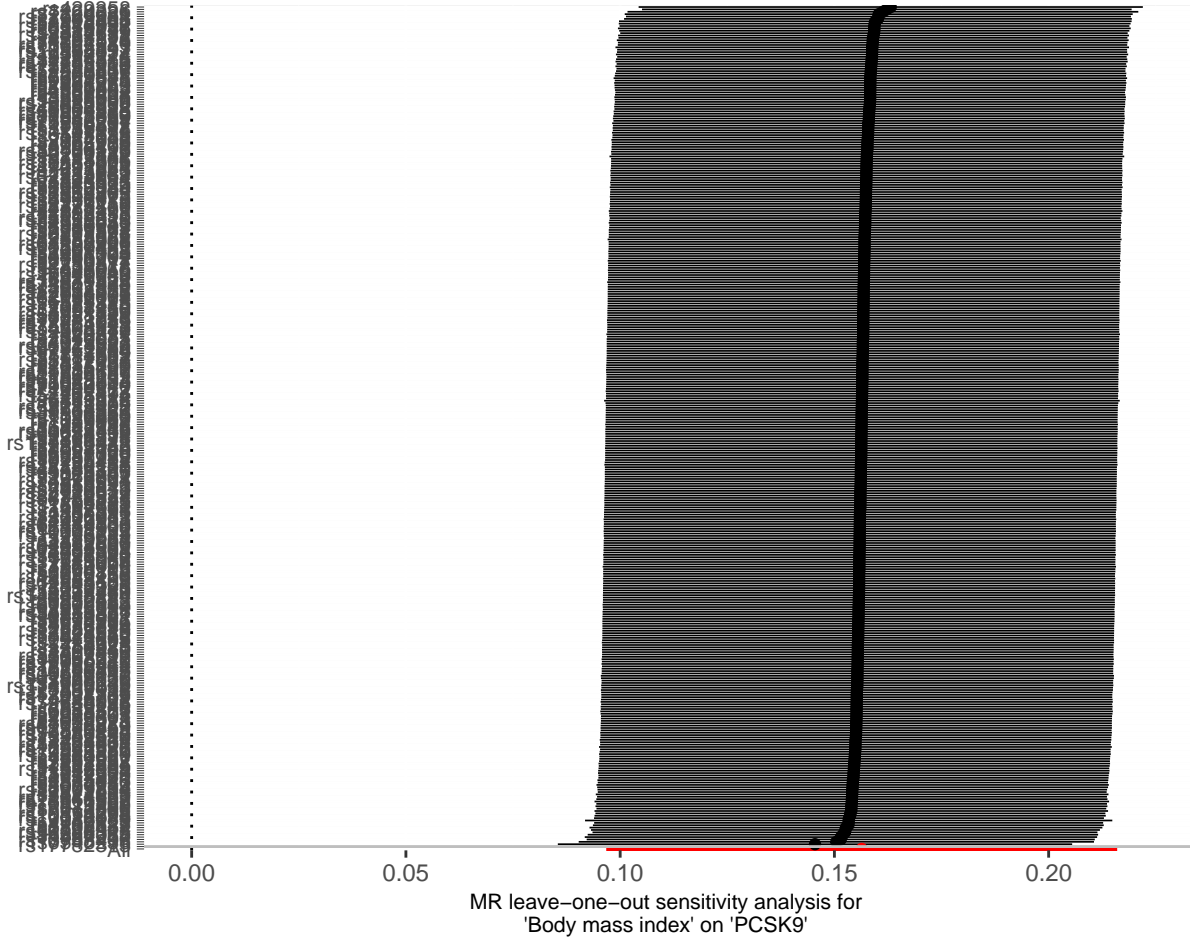

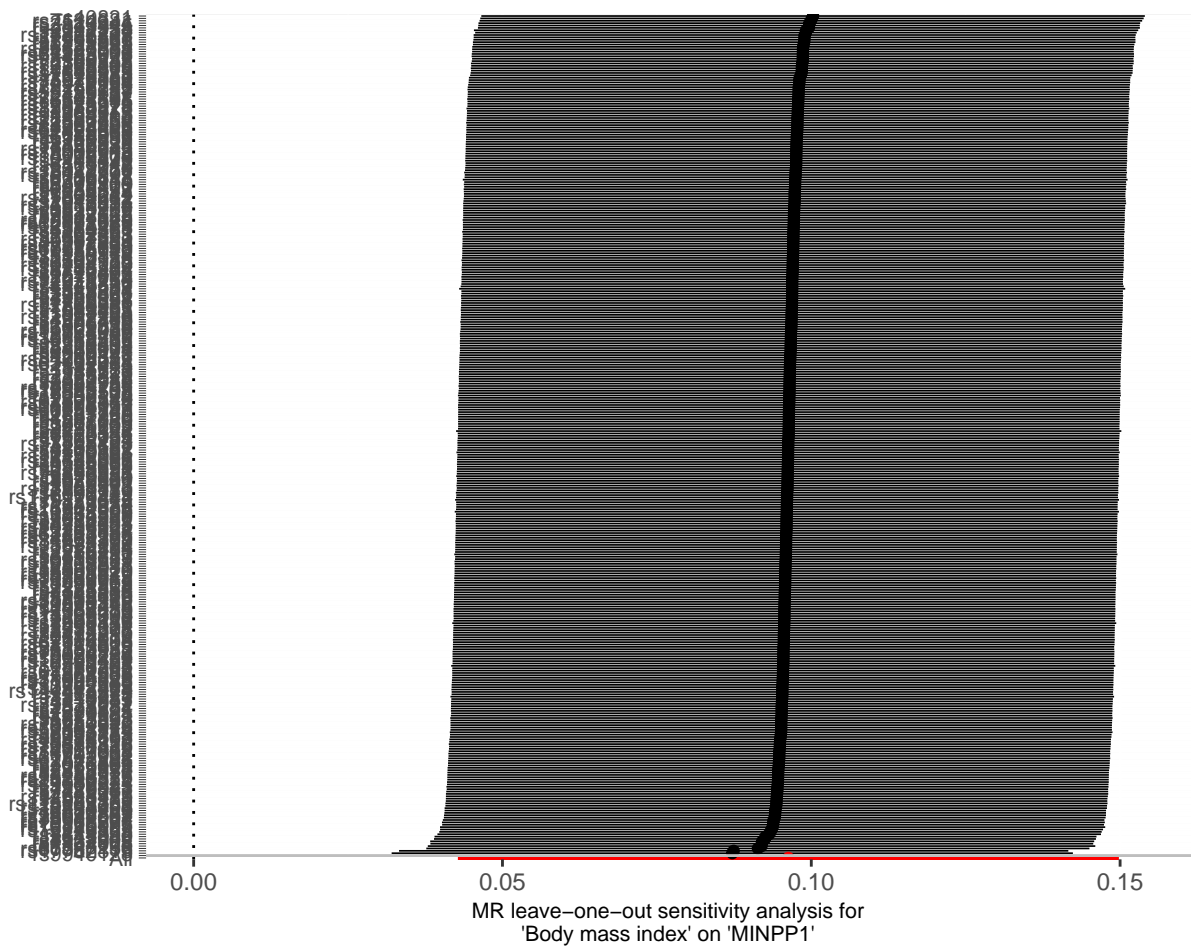

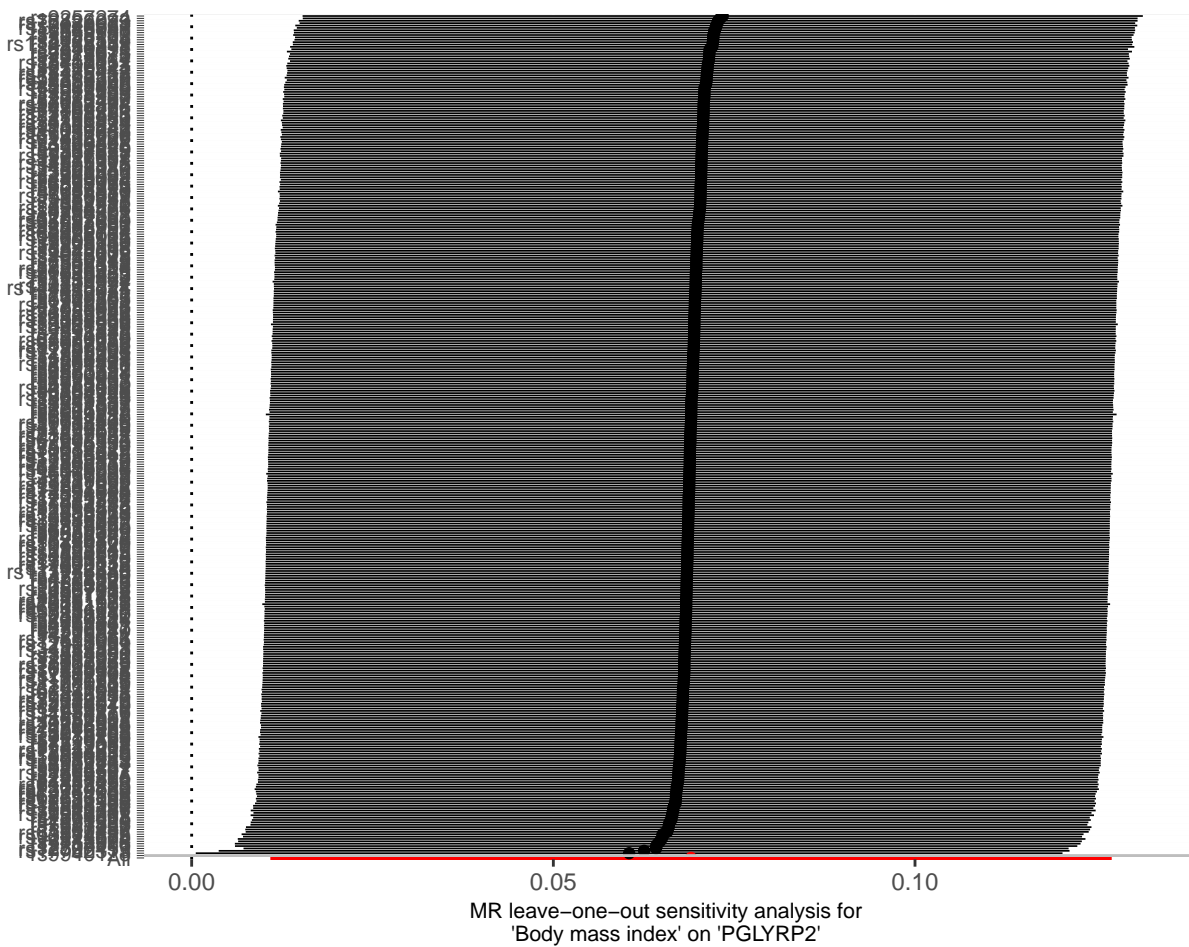

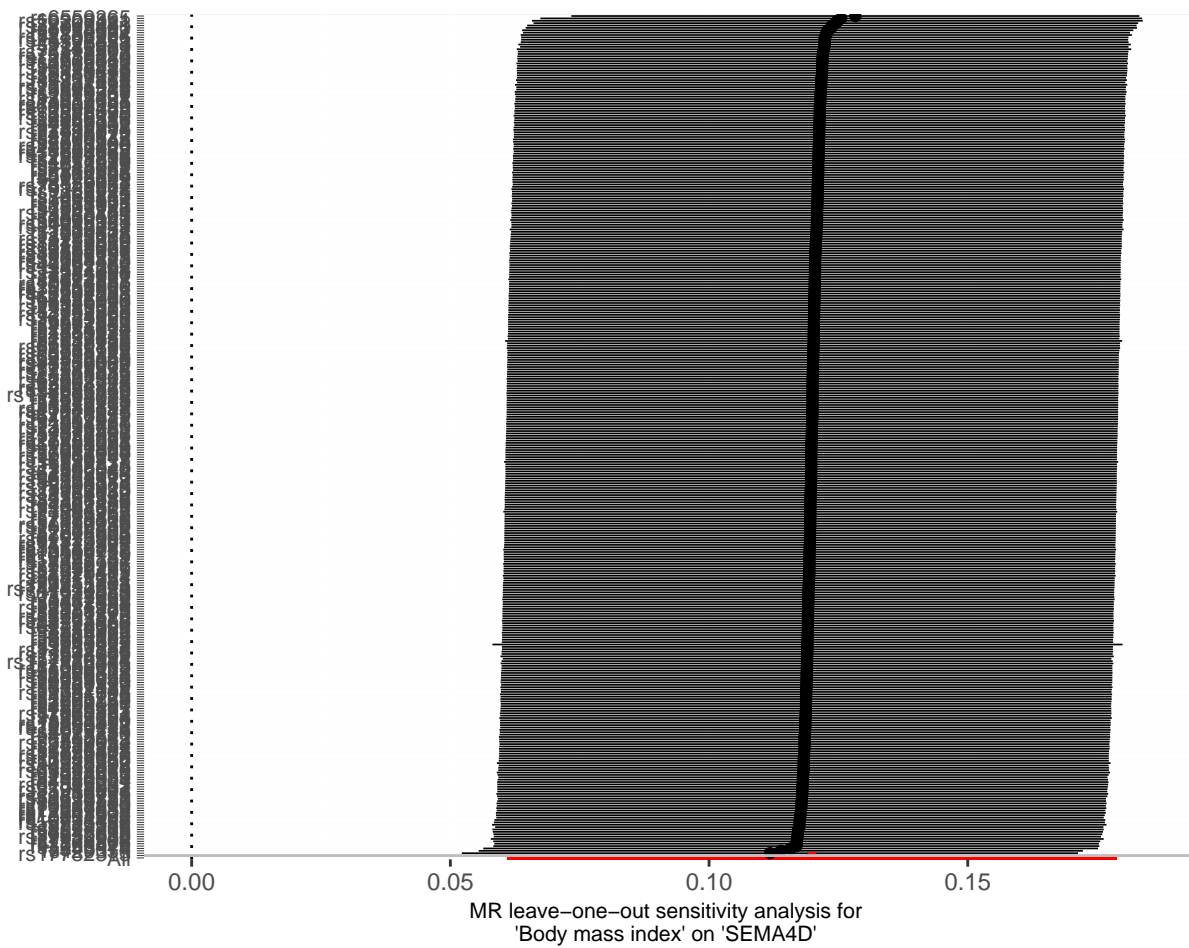

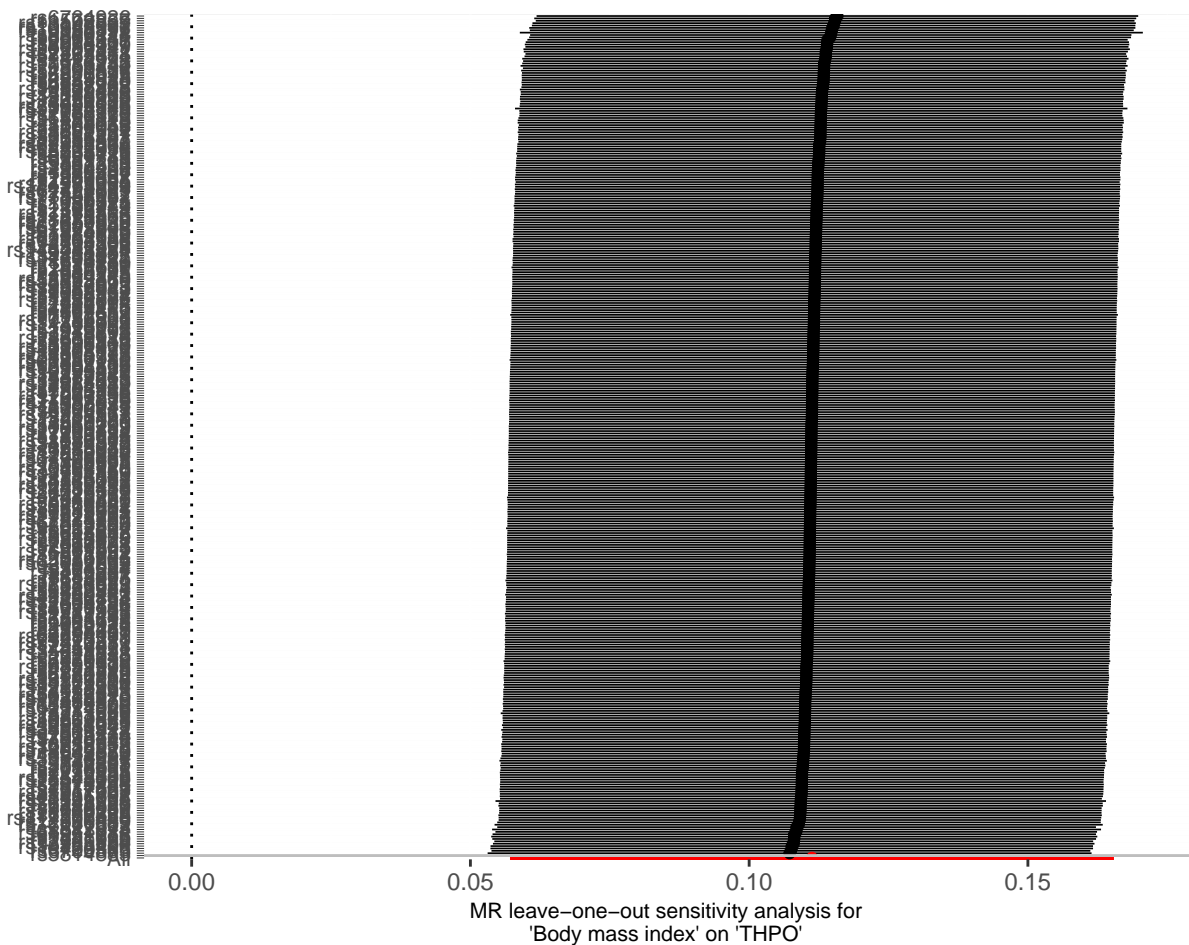

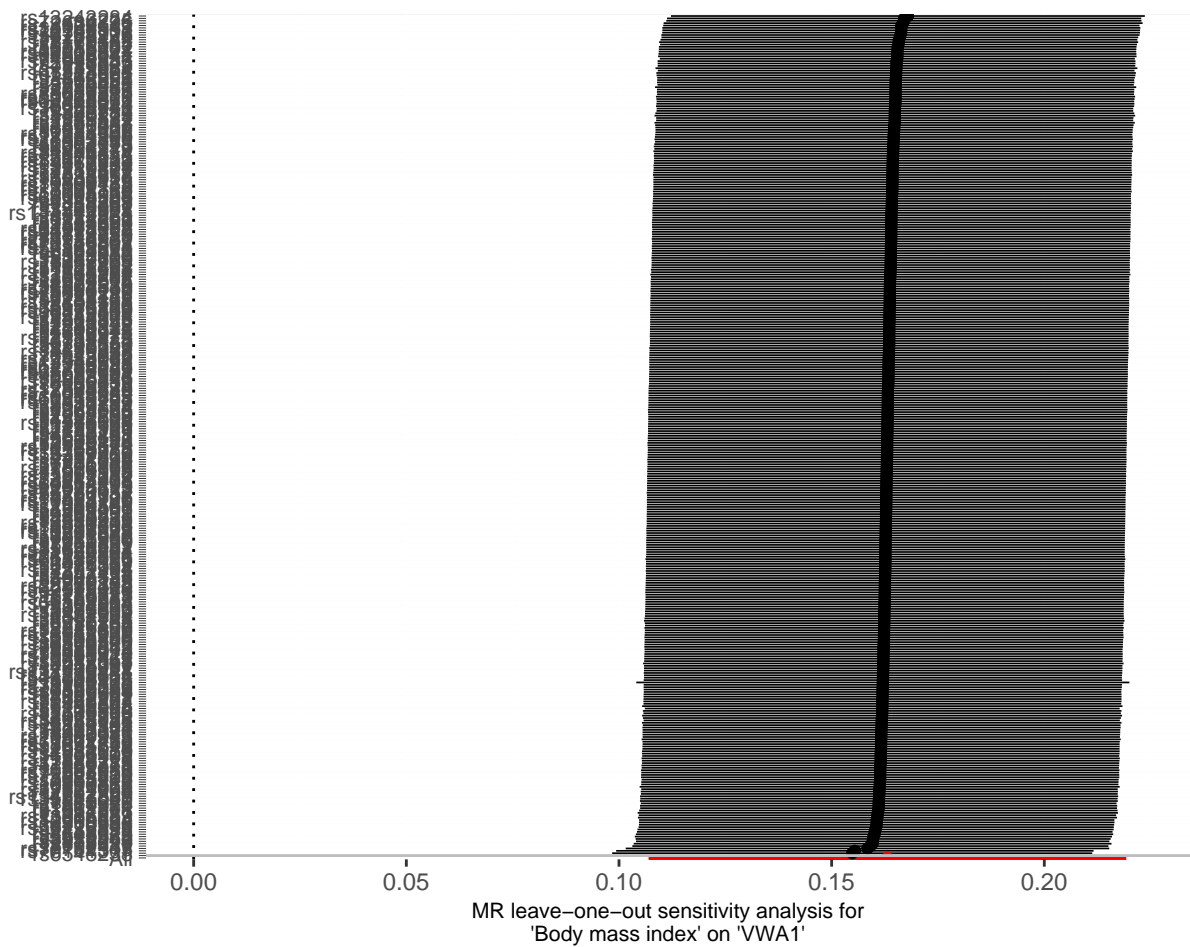

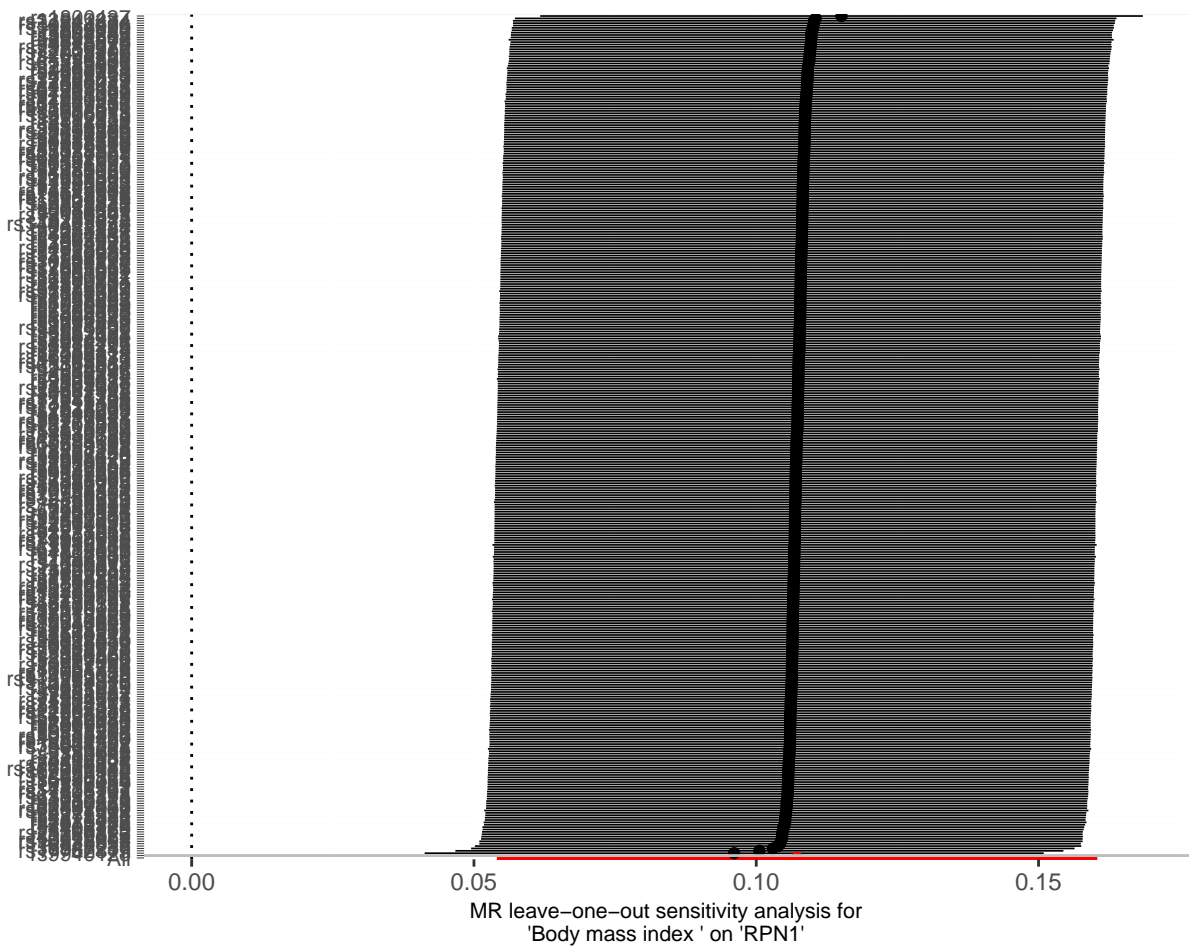

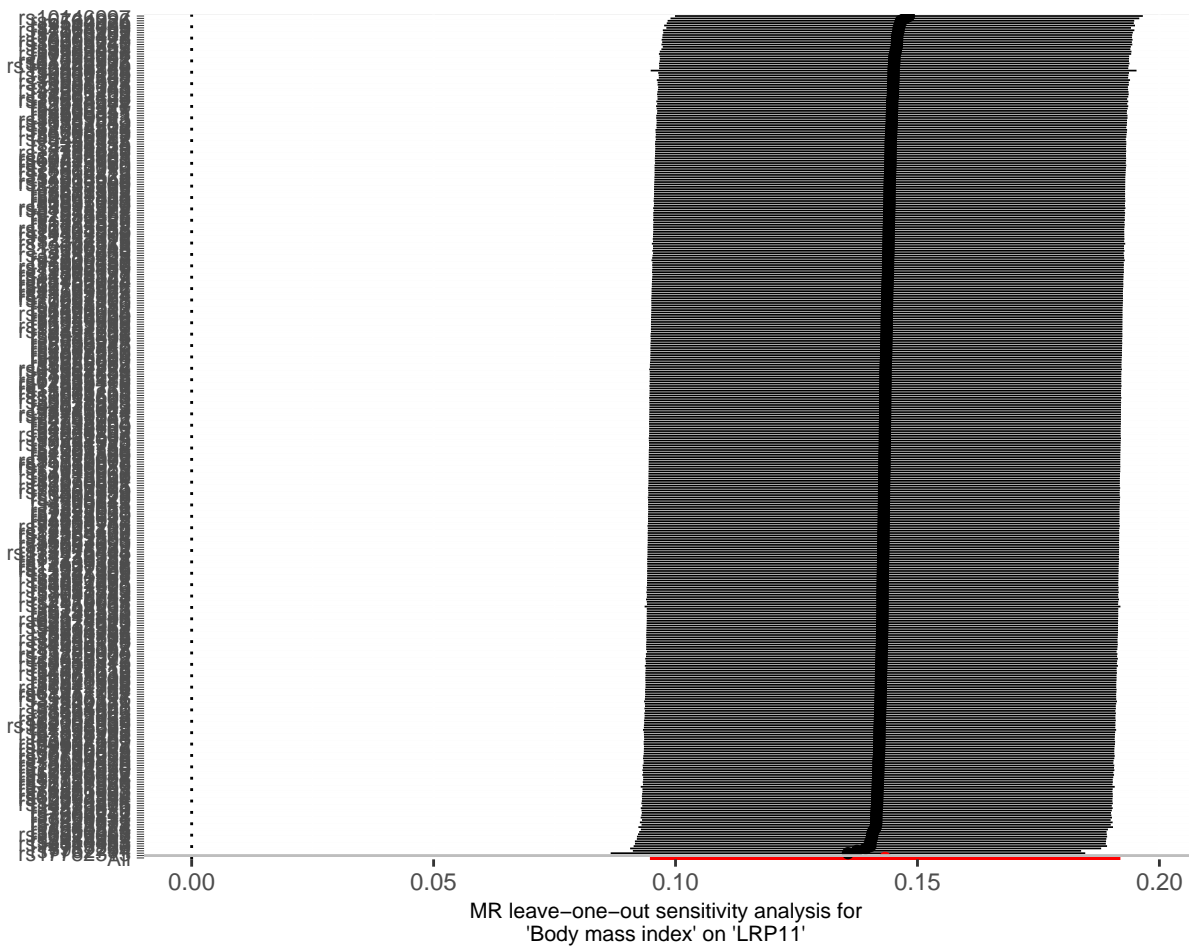

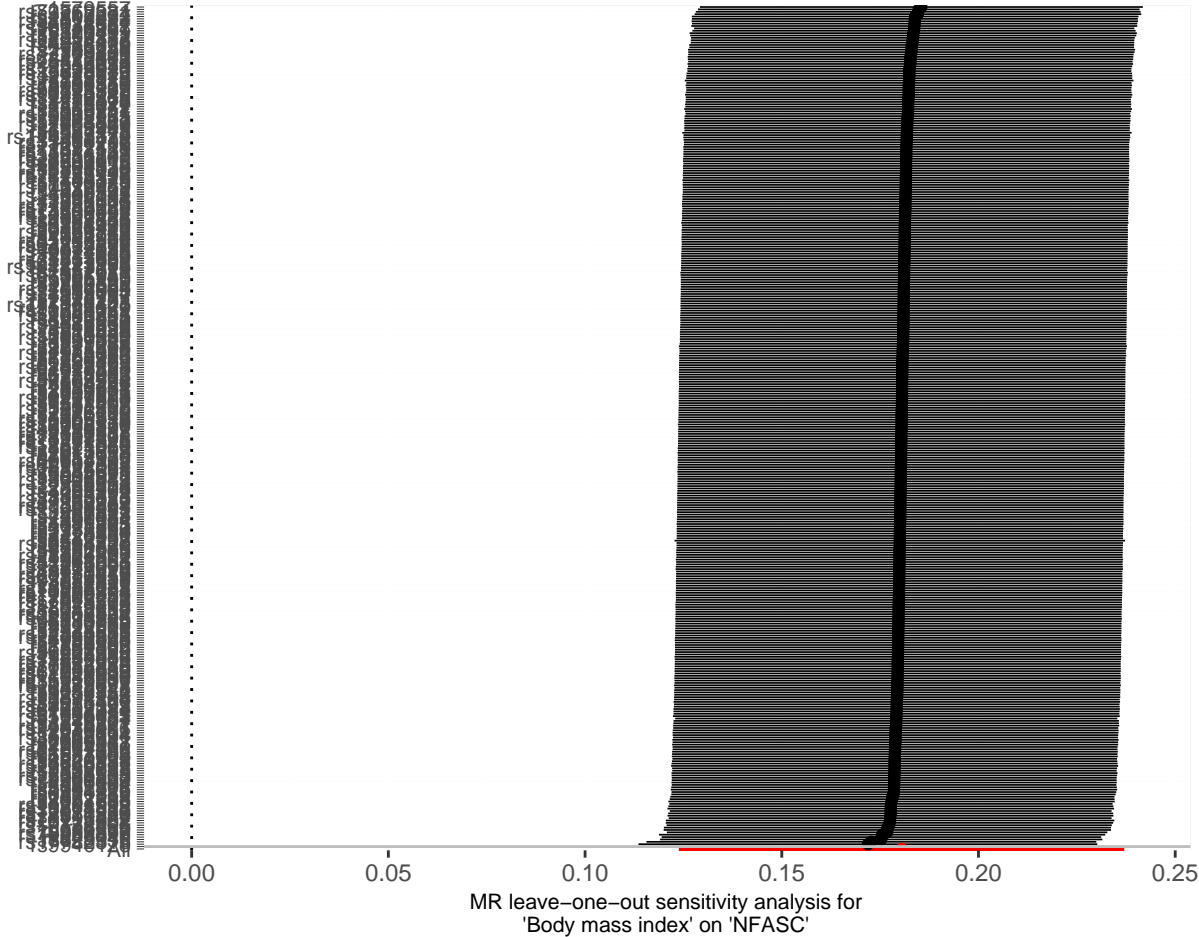

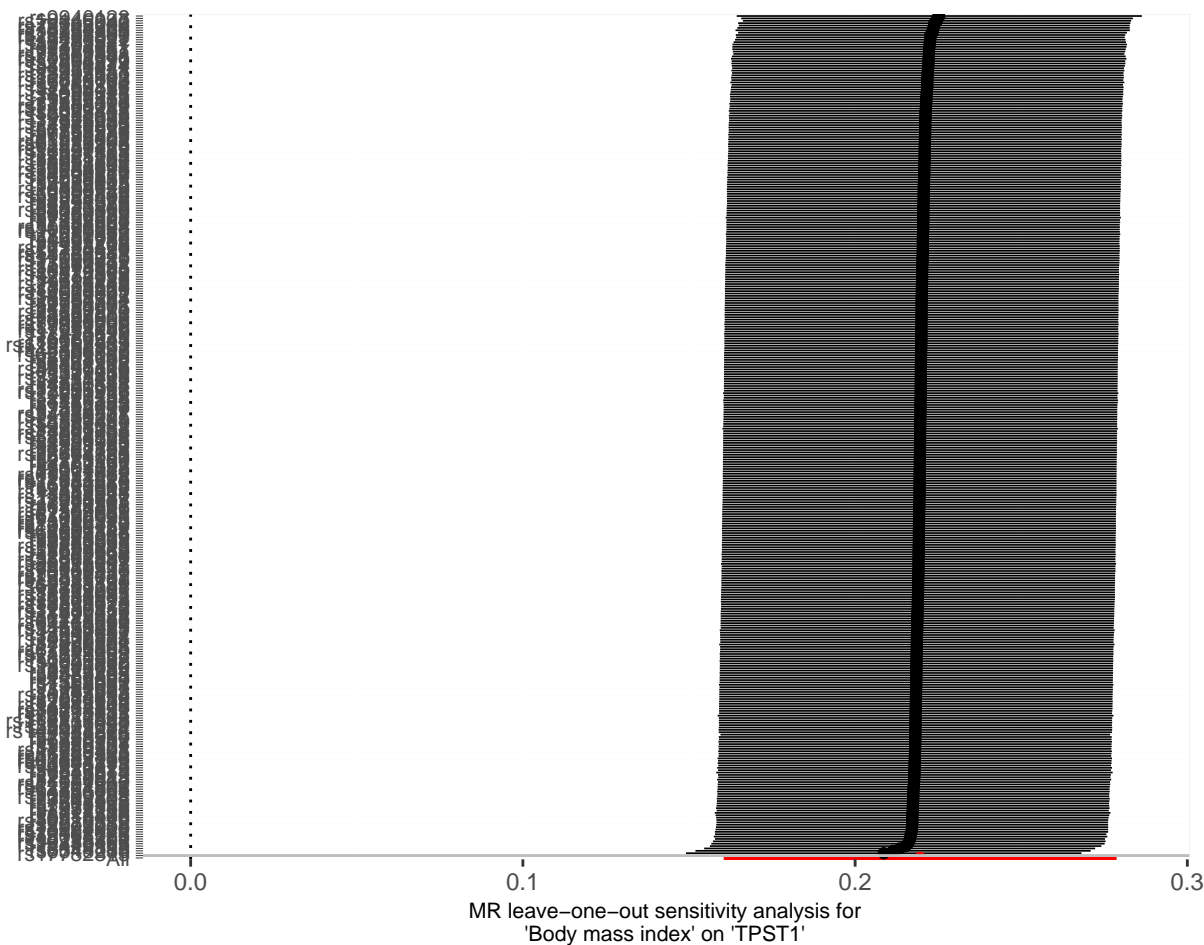

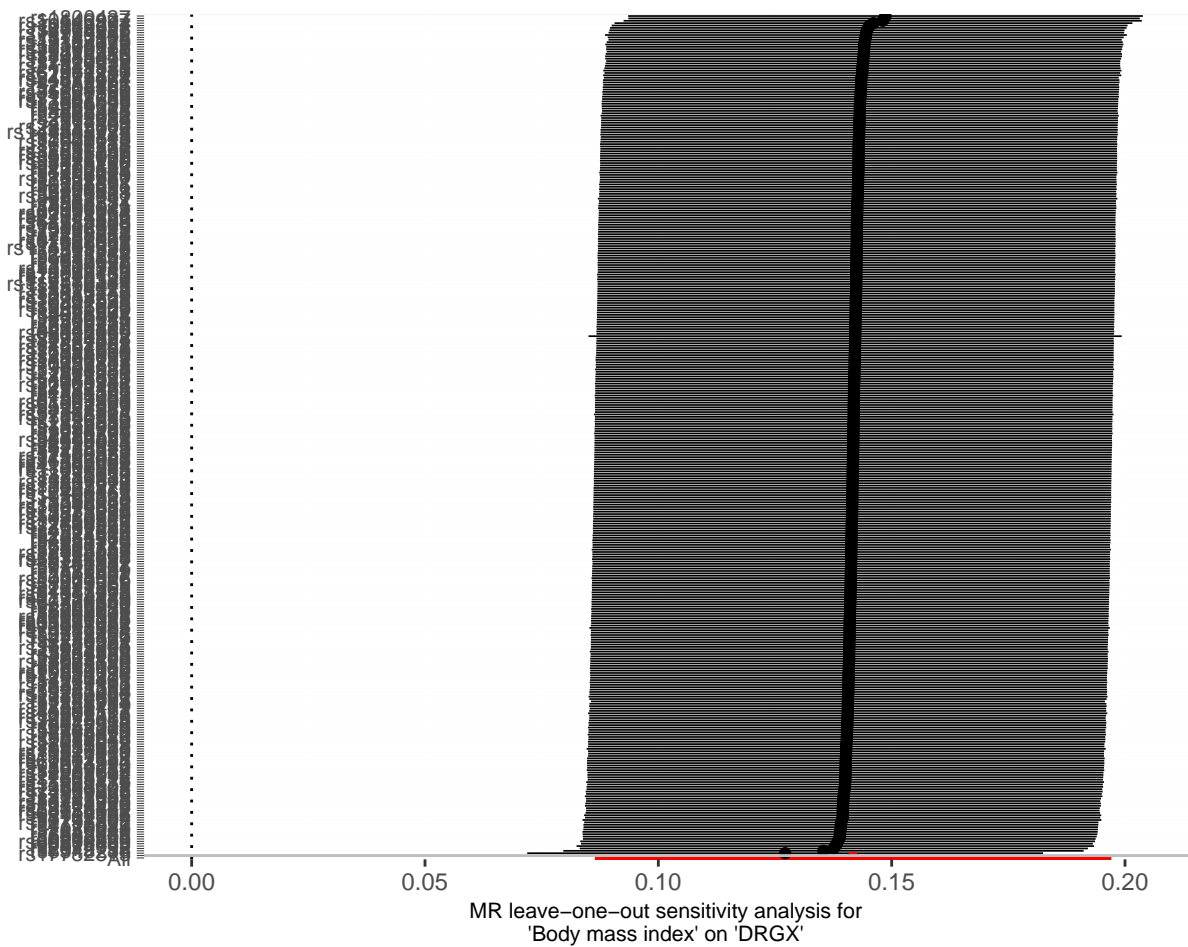

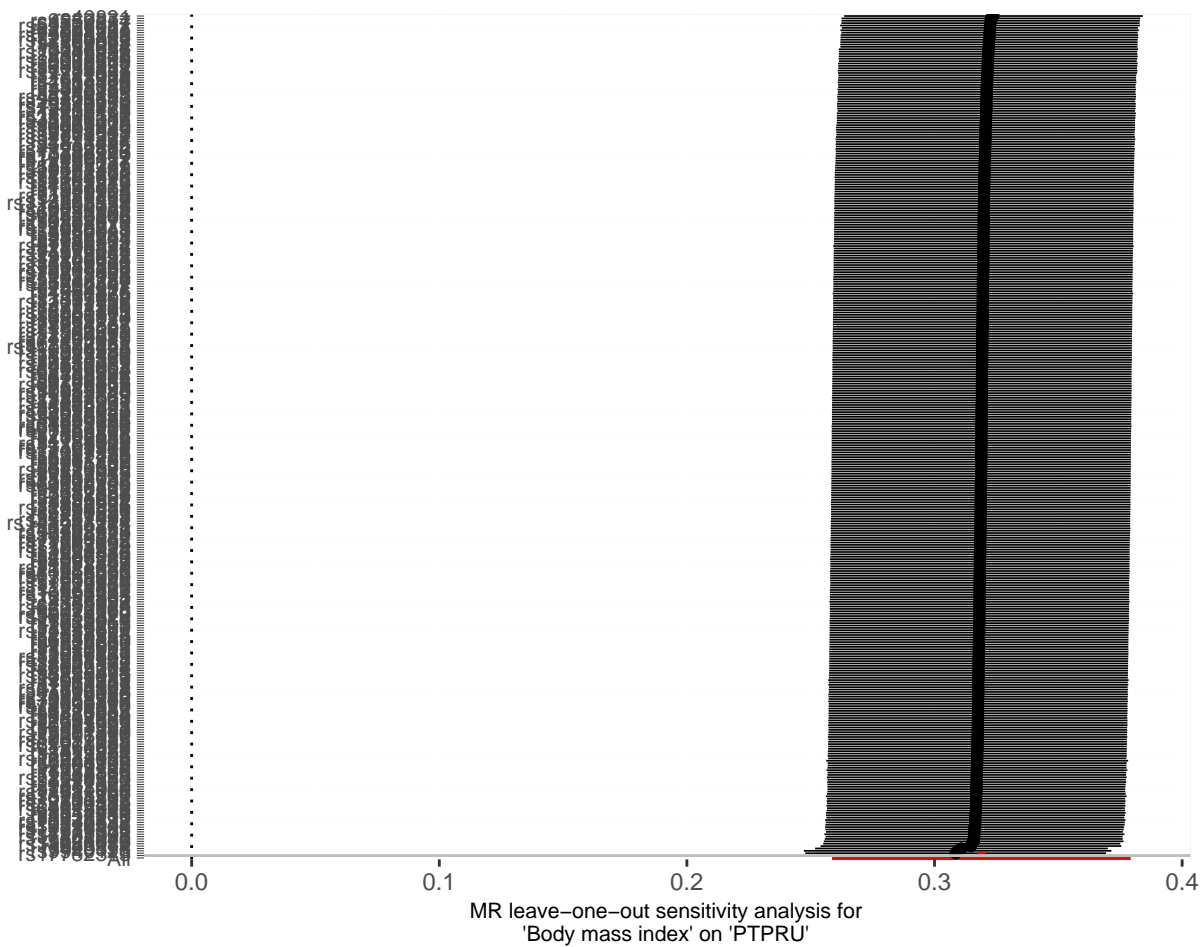

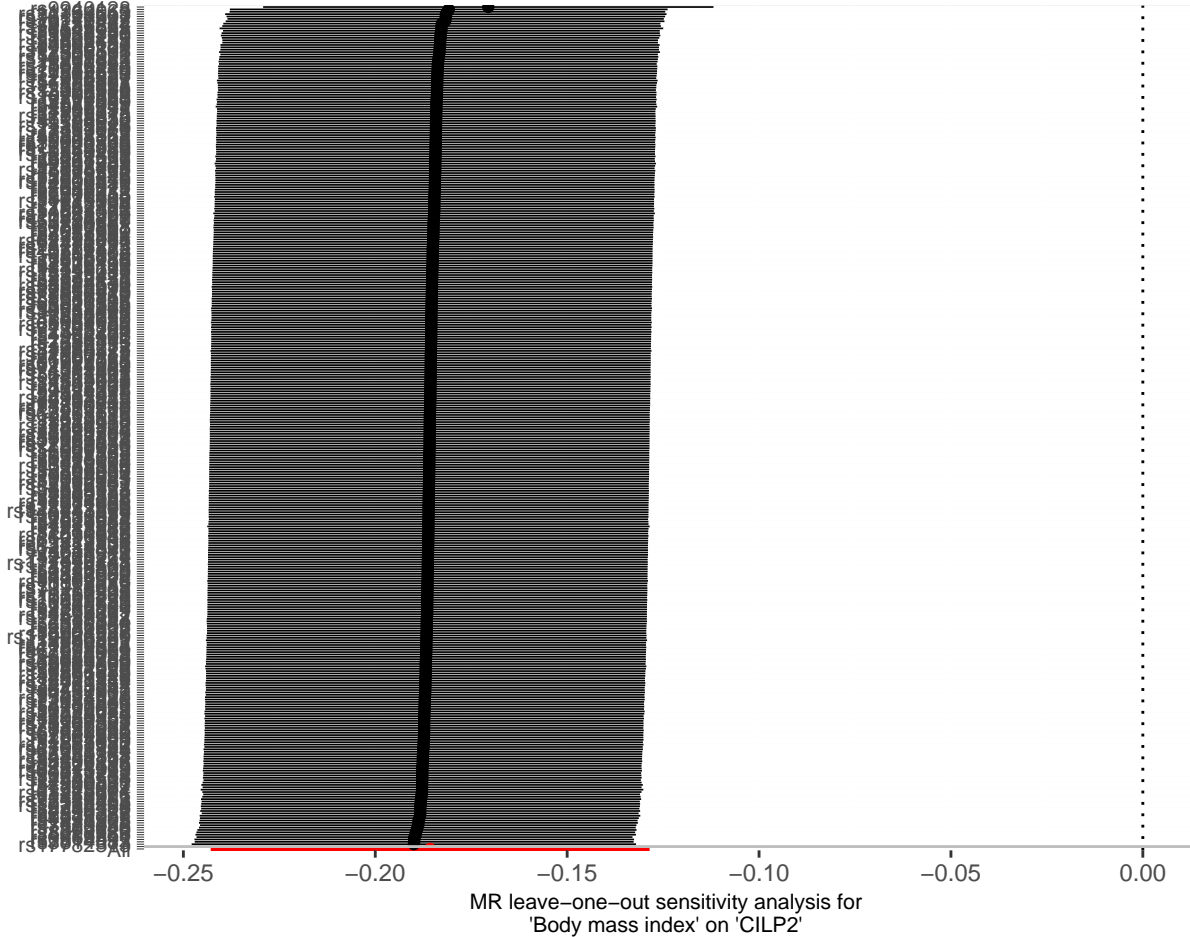

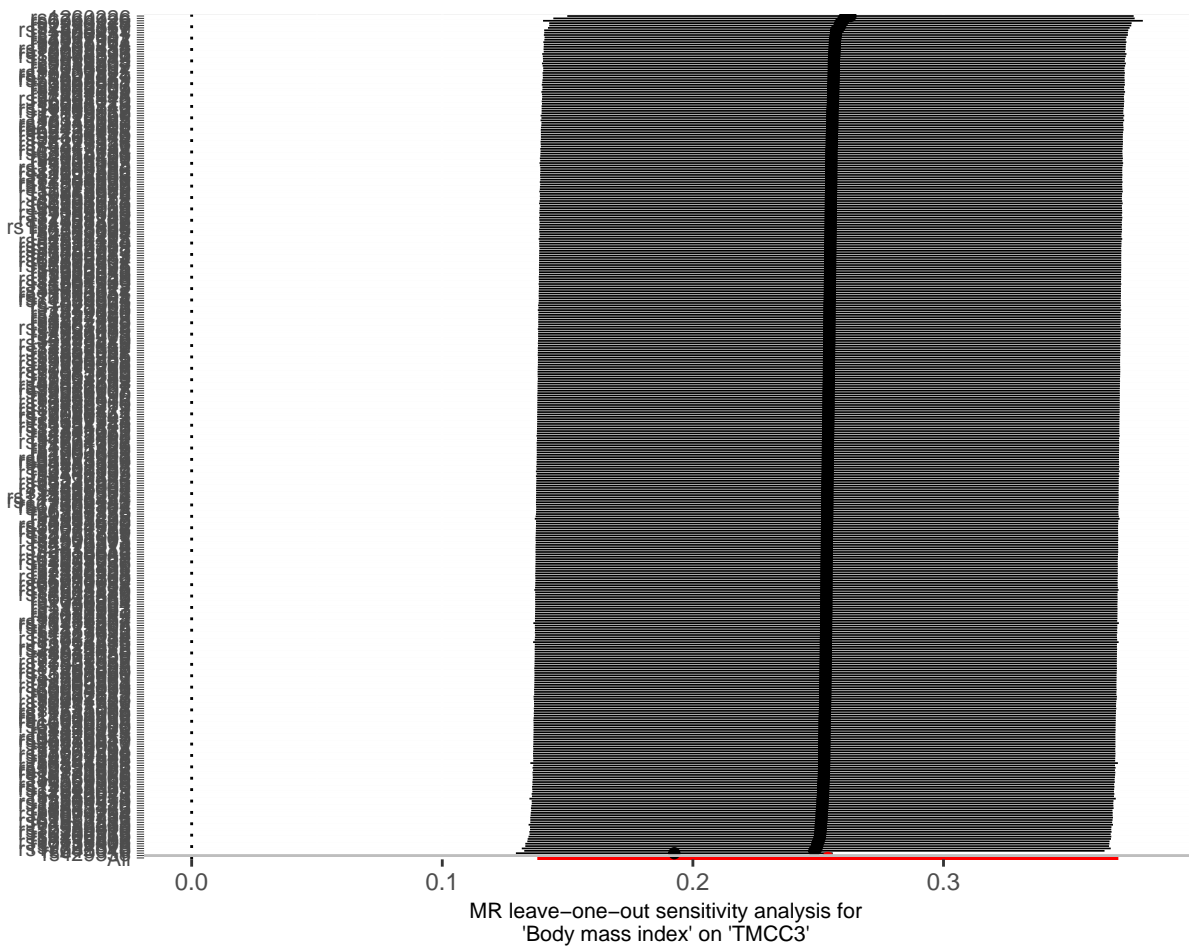

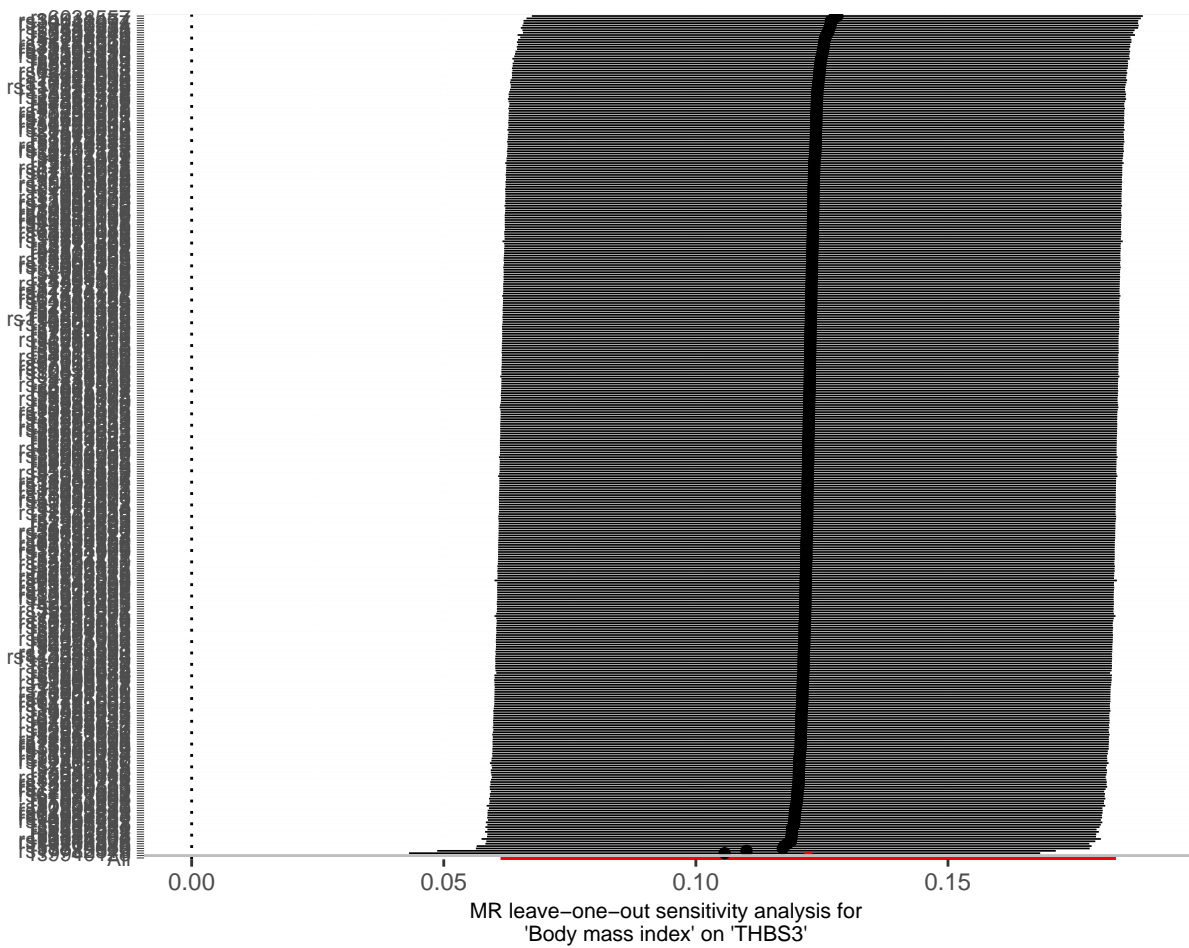

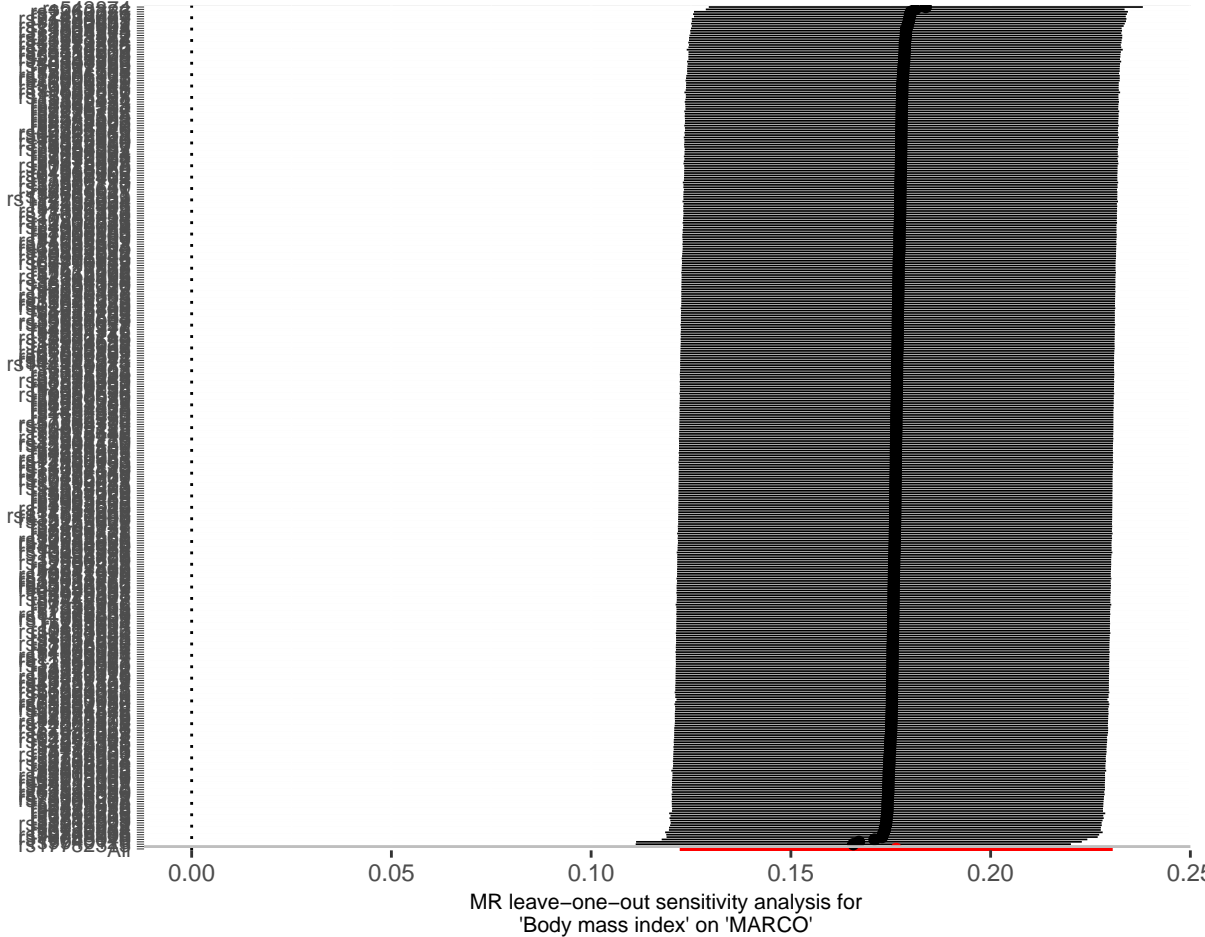

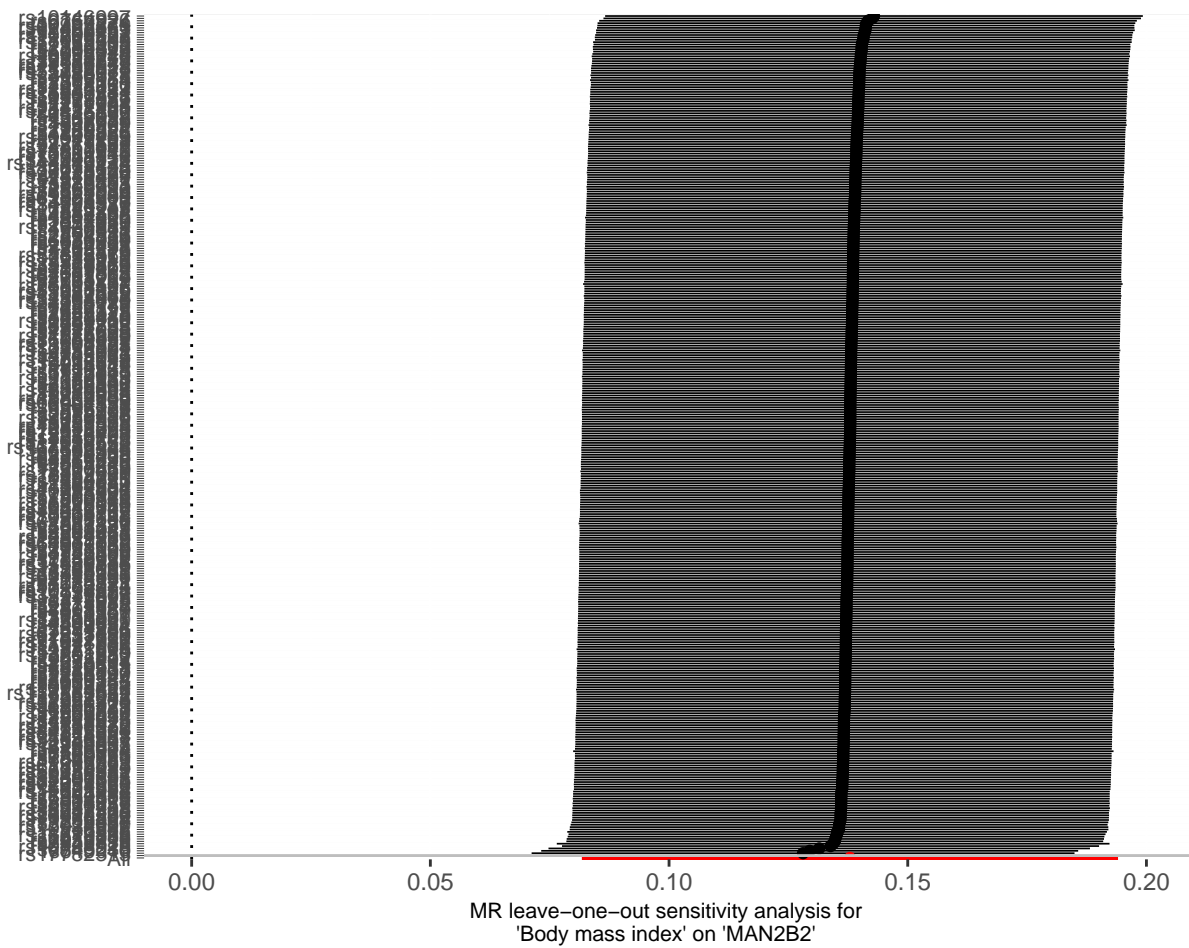

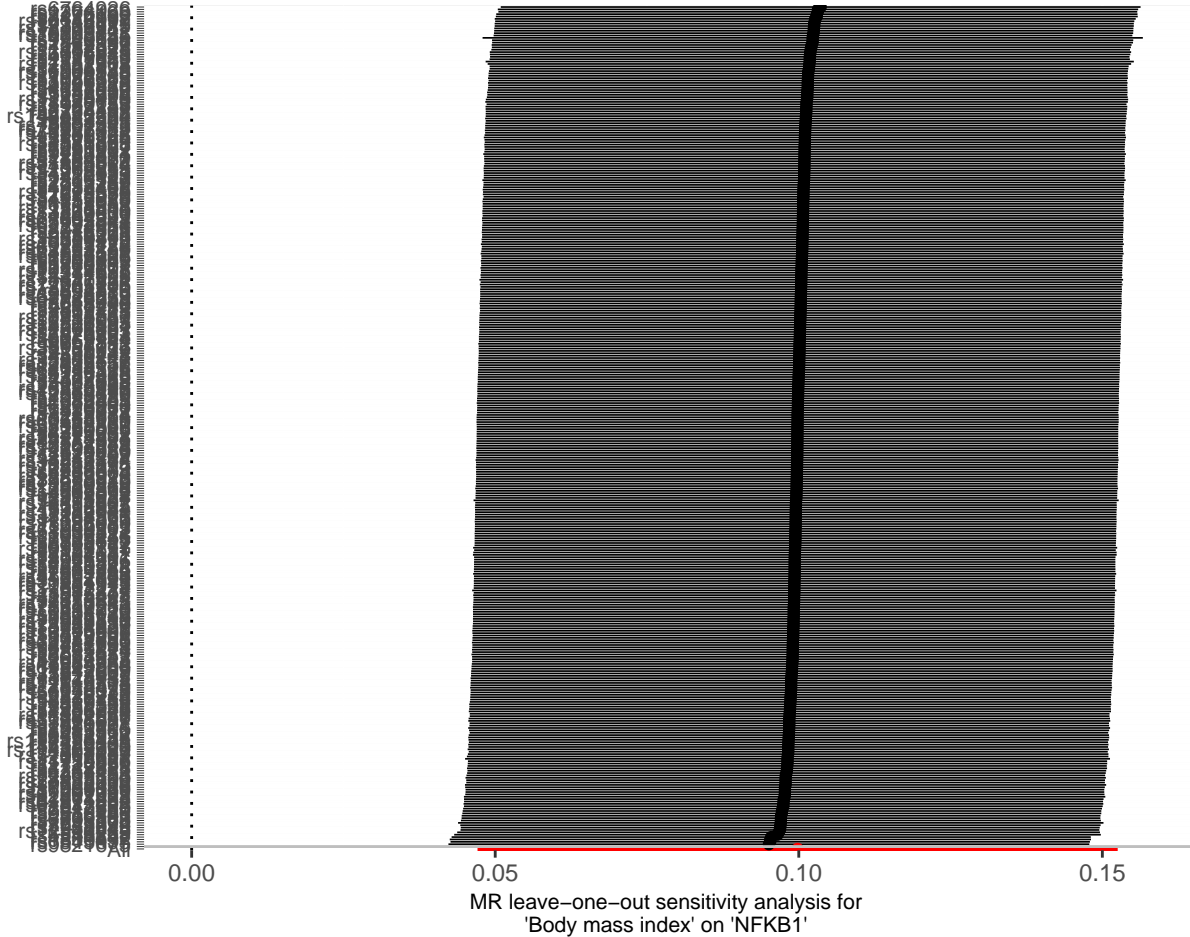

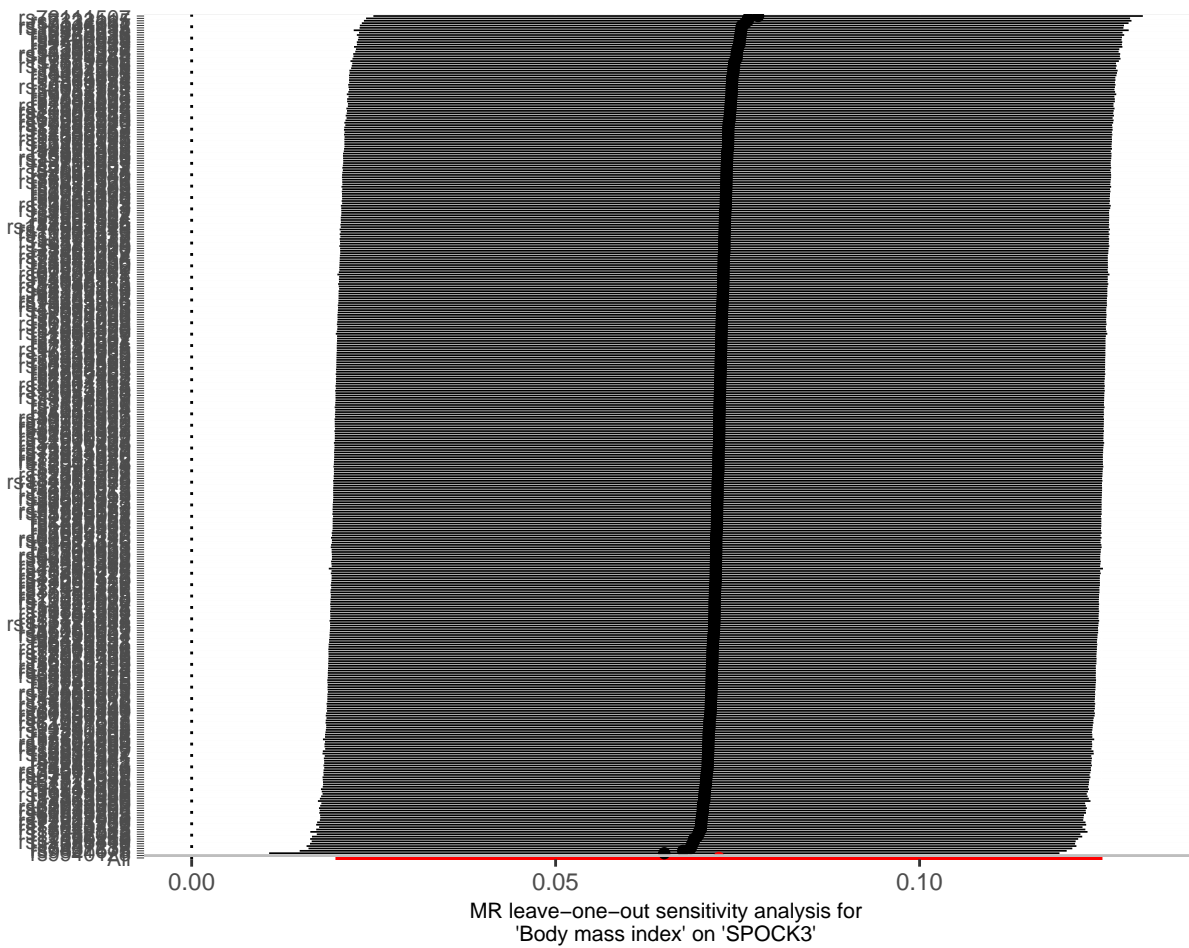

Supplement: Supplementary file 1 — Supplementary Material 1: Supplementary Materials 1. The MR results of BMI and allergic asthma. Supplementary Materials 2. The MR results of plasma proteins and allergic asthma. Supplementary Materials 3. The MR results of BMI and plasma proteins. Supplementary Materials 4. The MR results of mediation factor plasma proteins and allergic asthma. Supplementary Materials 5. The results of drug-targeted MR [file 41065_2025_376_MOESM1_ESM.zip › Supplementary Materials/S3 The MR results of BMI and plasma proteins/S3 BMI-plasma proteins.leaveoneout.pdf]

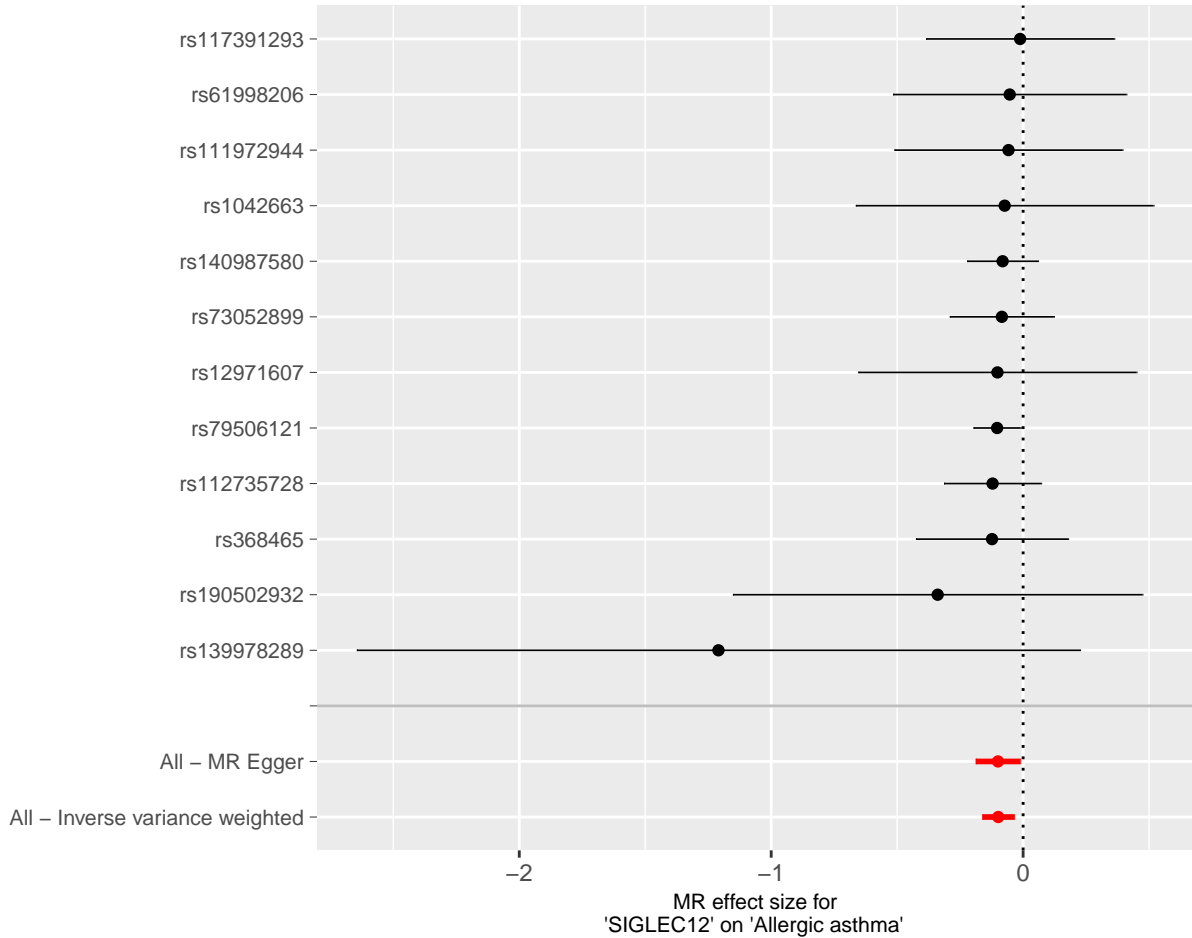

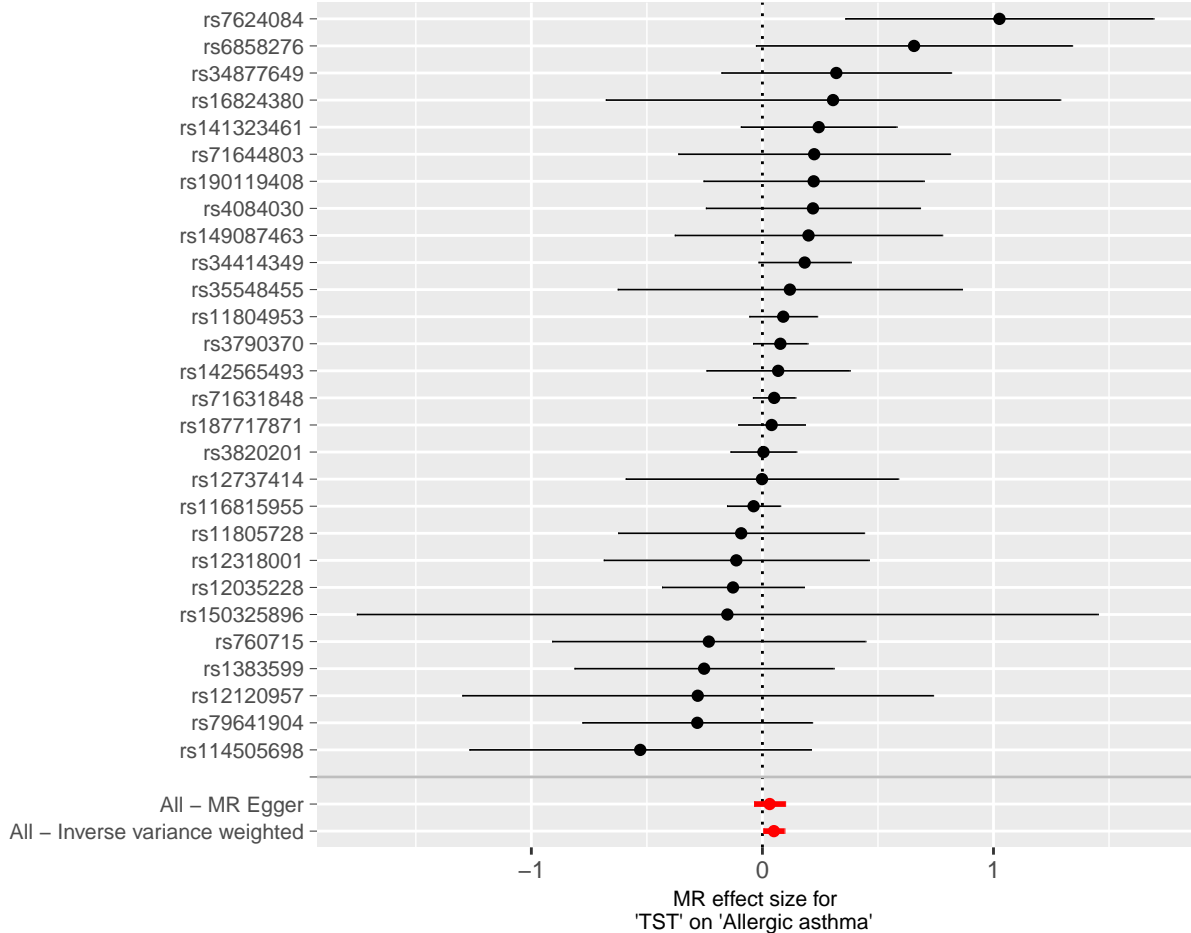

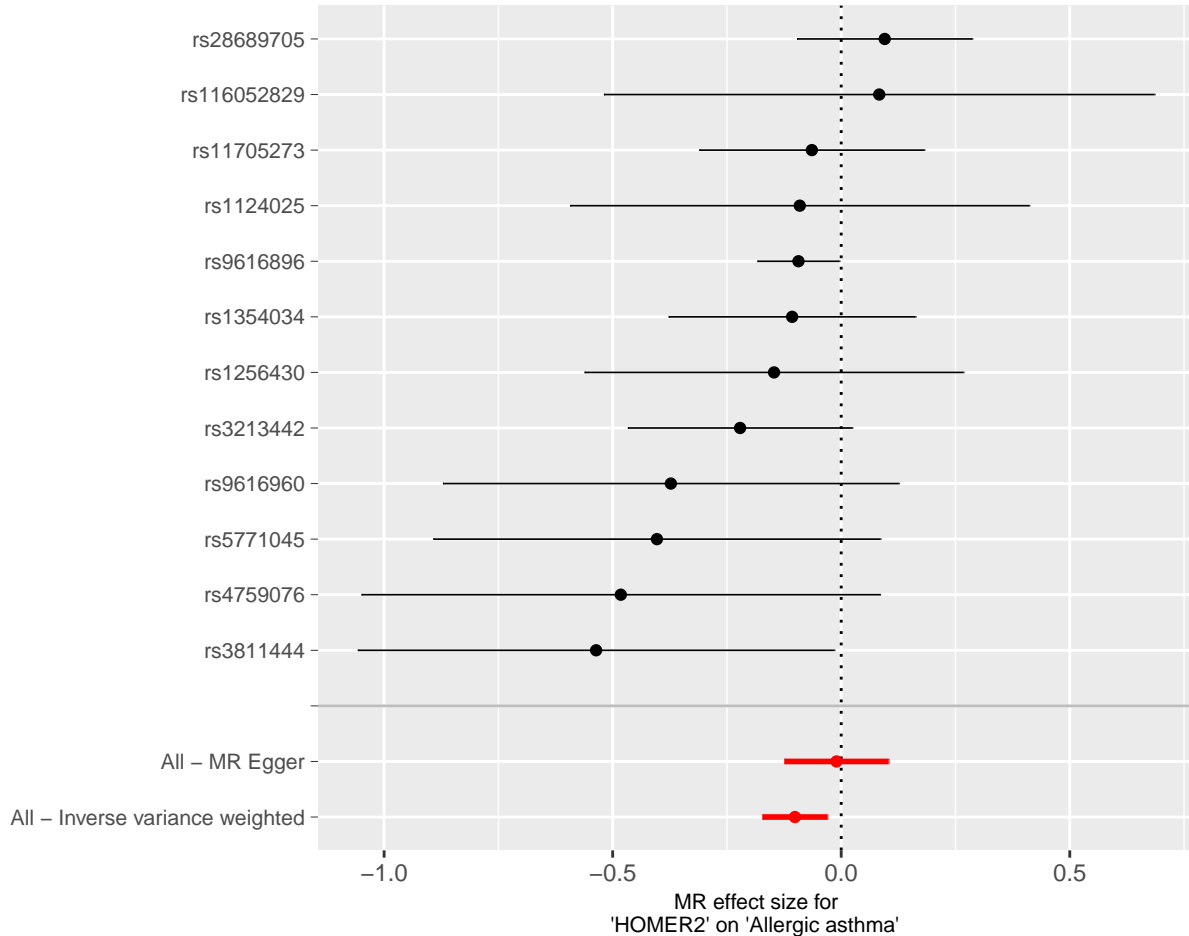

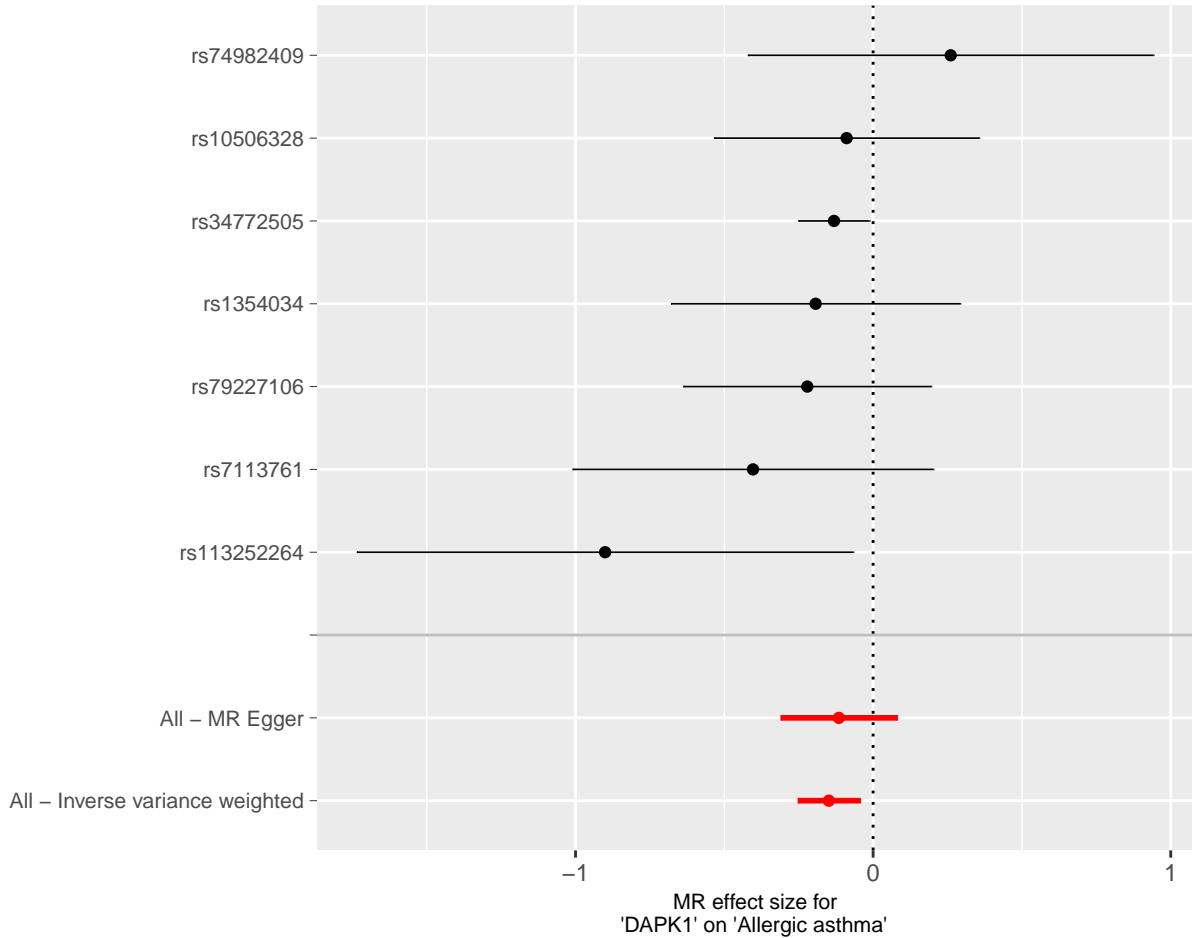

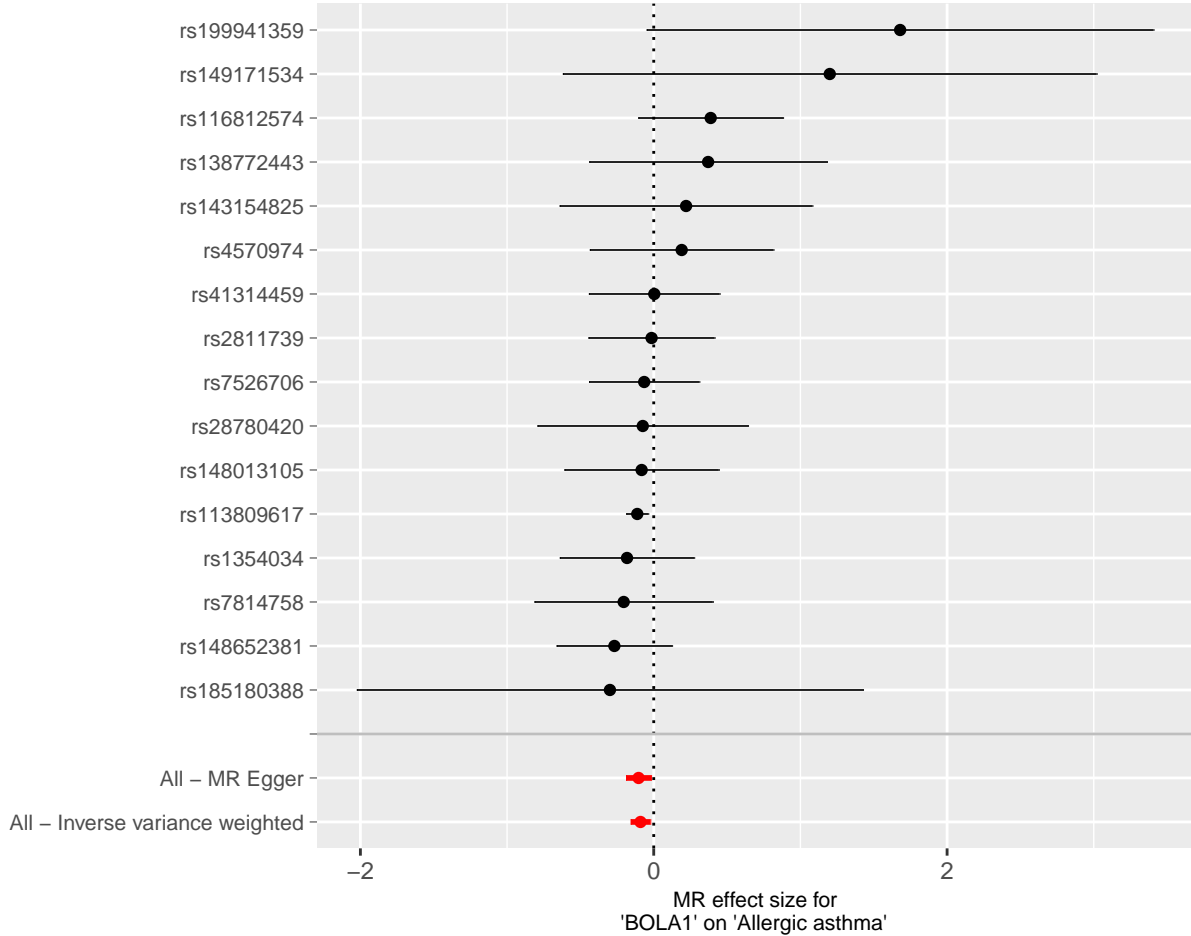

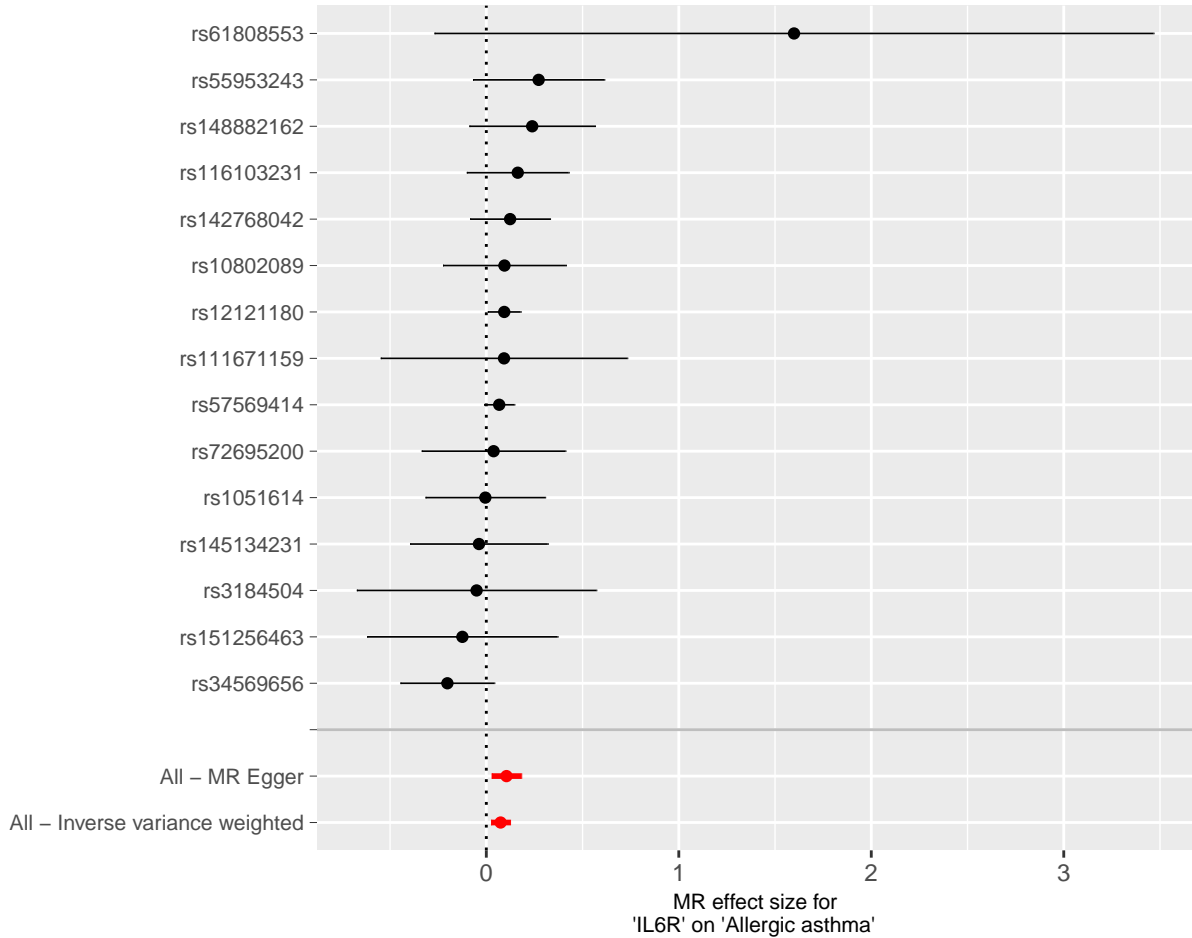

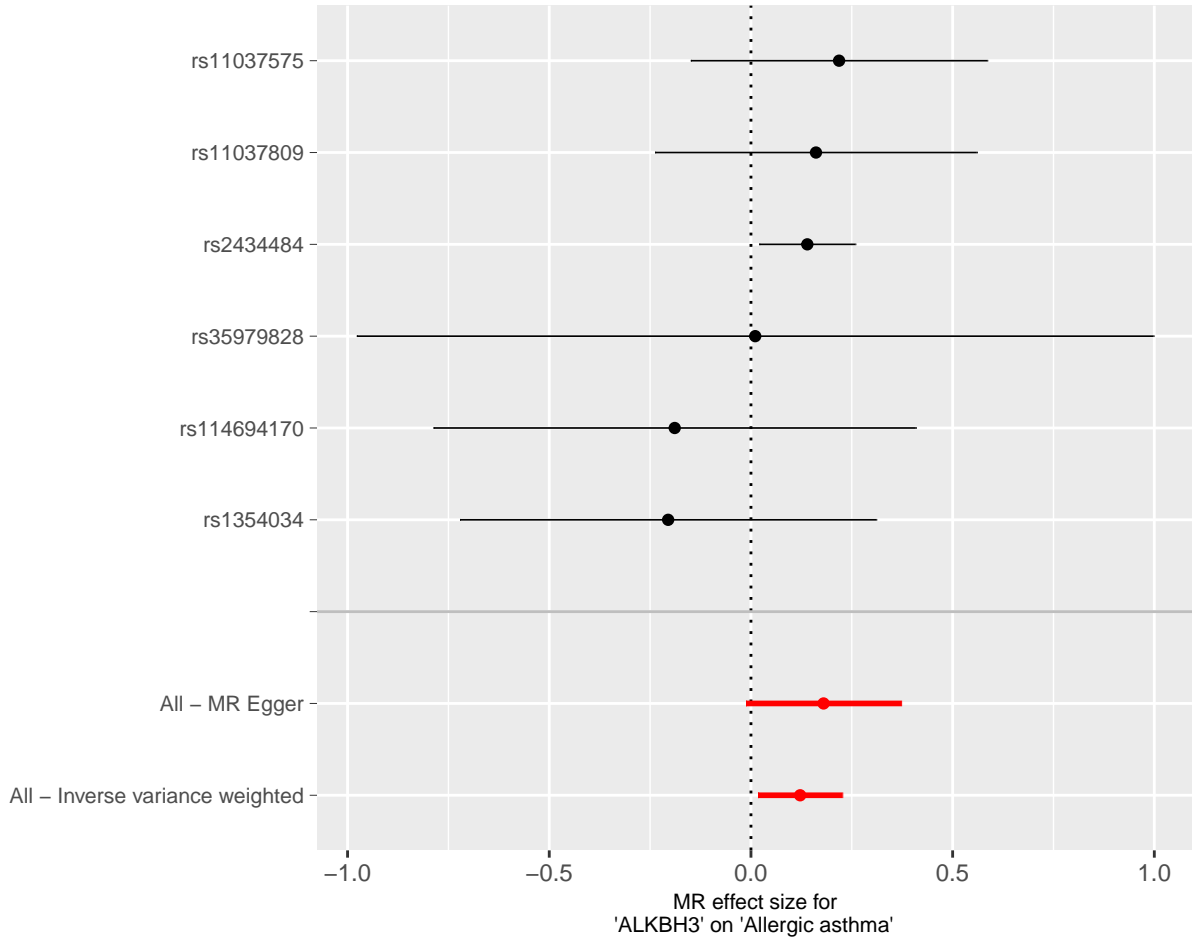

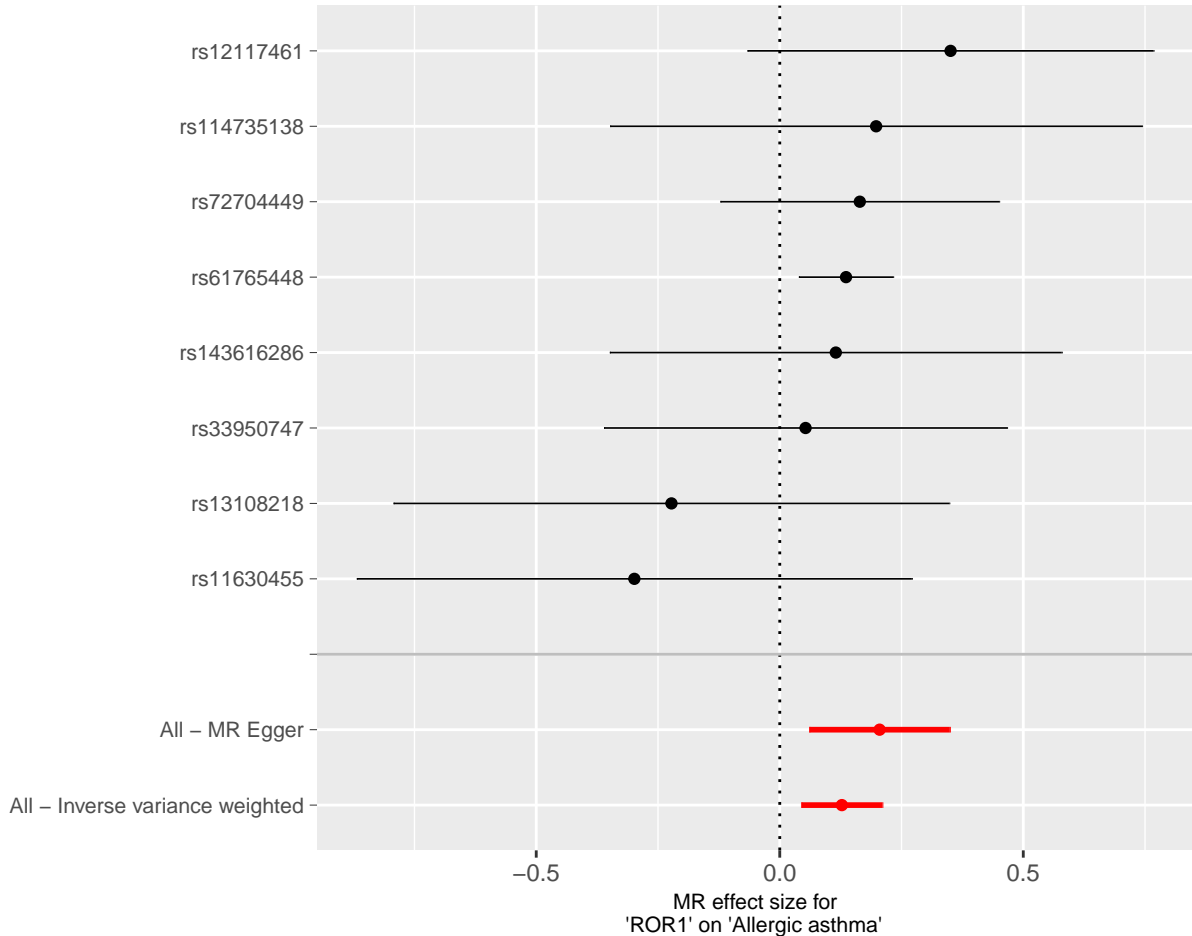

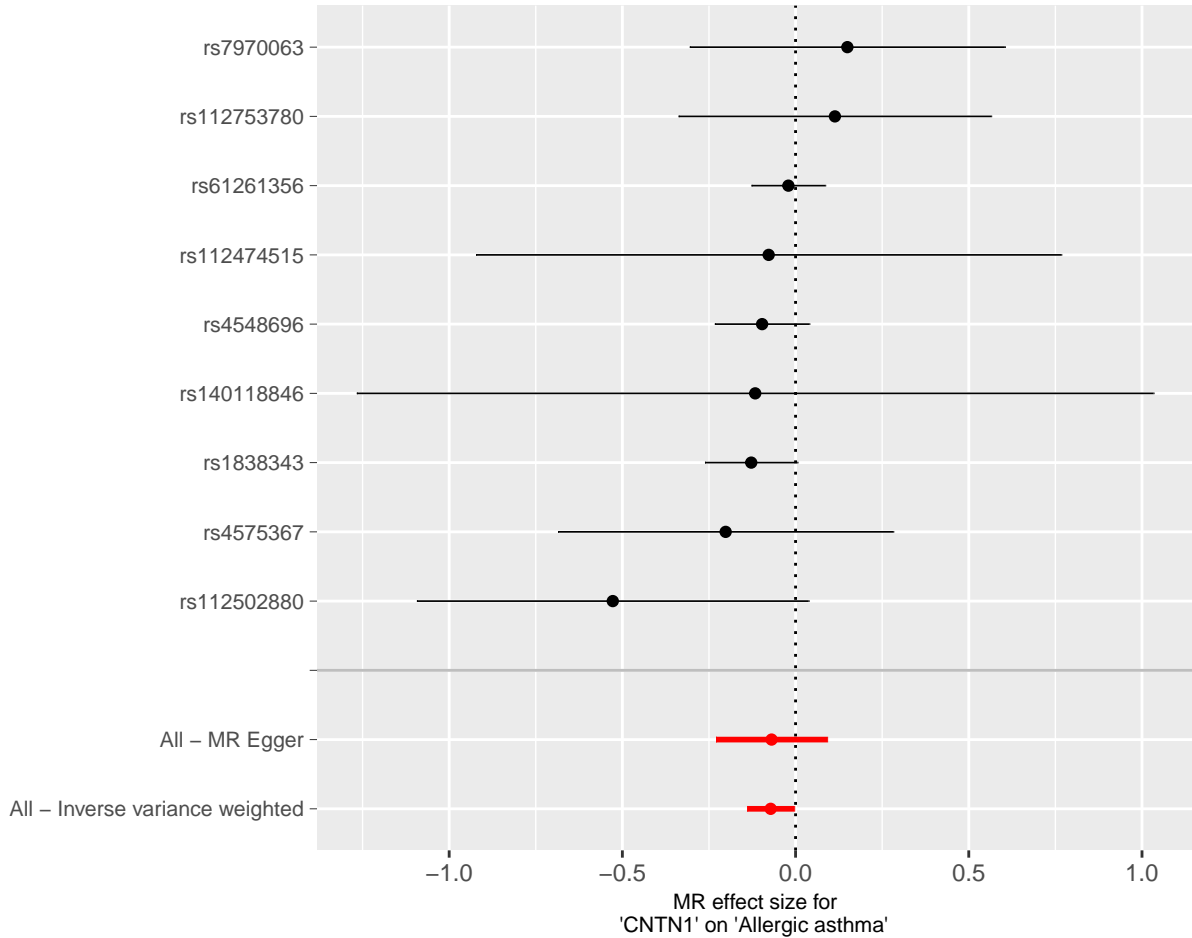

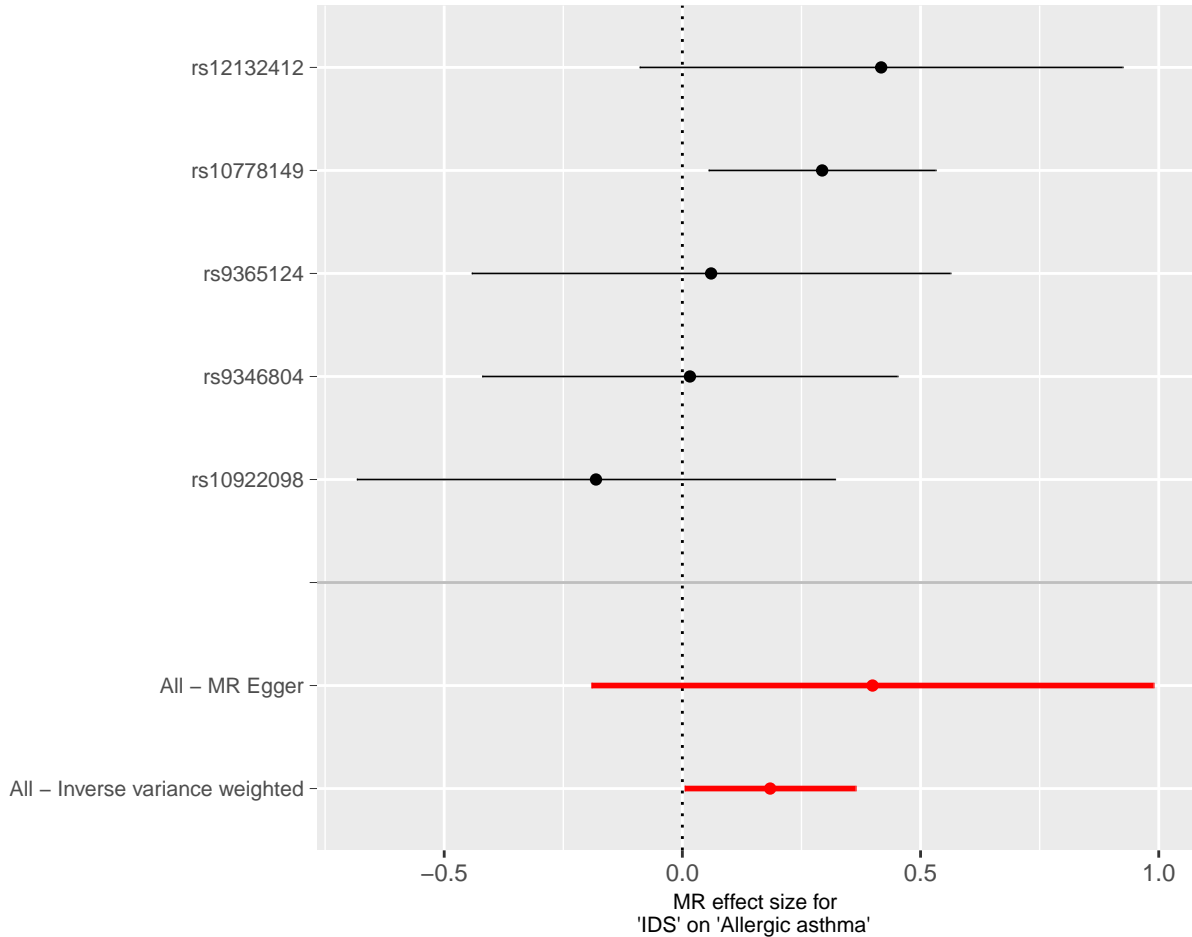

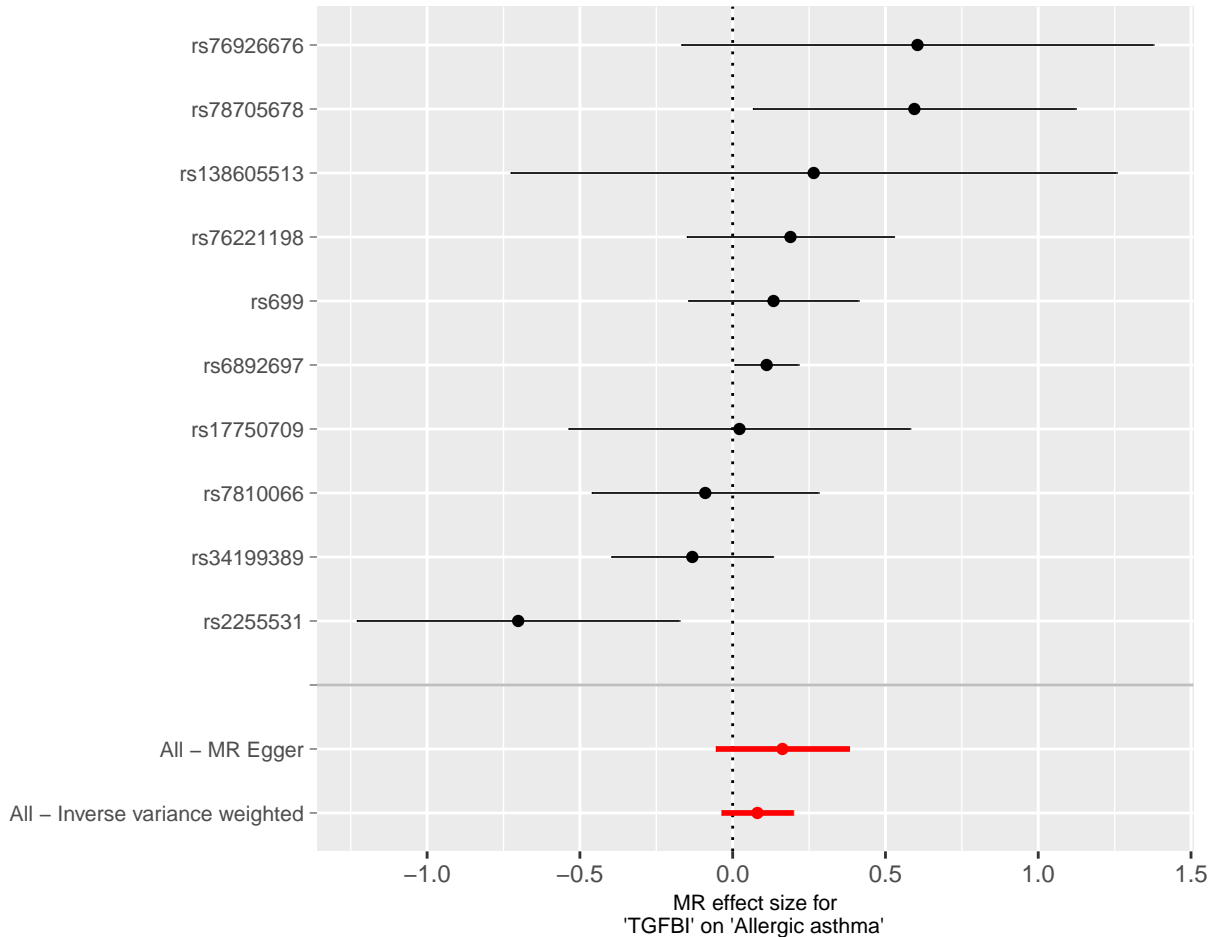

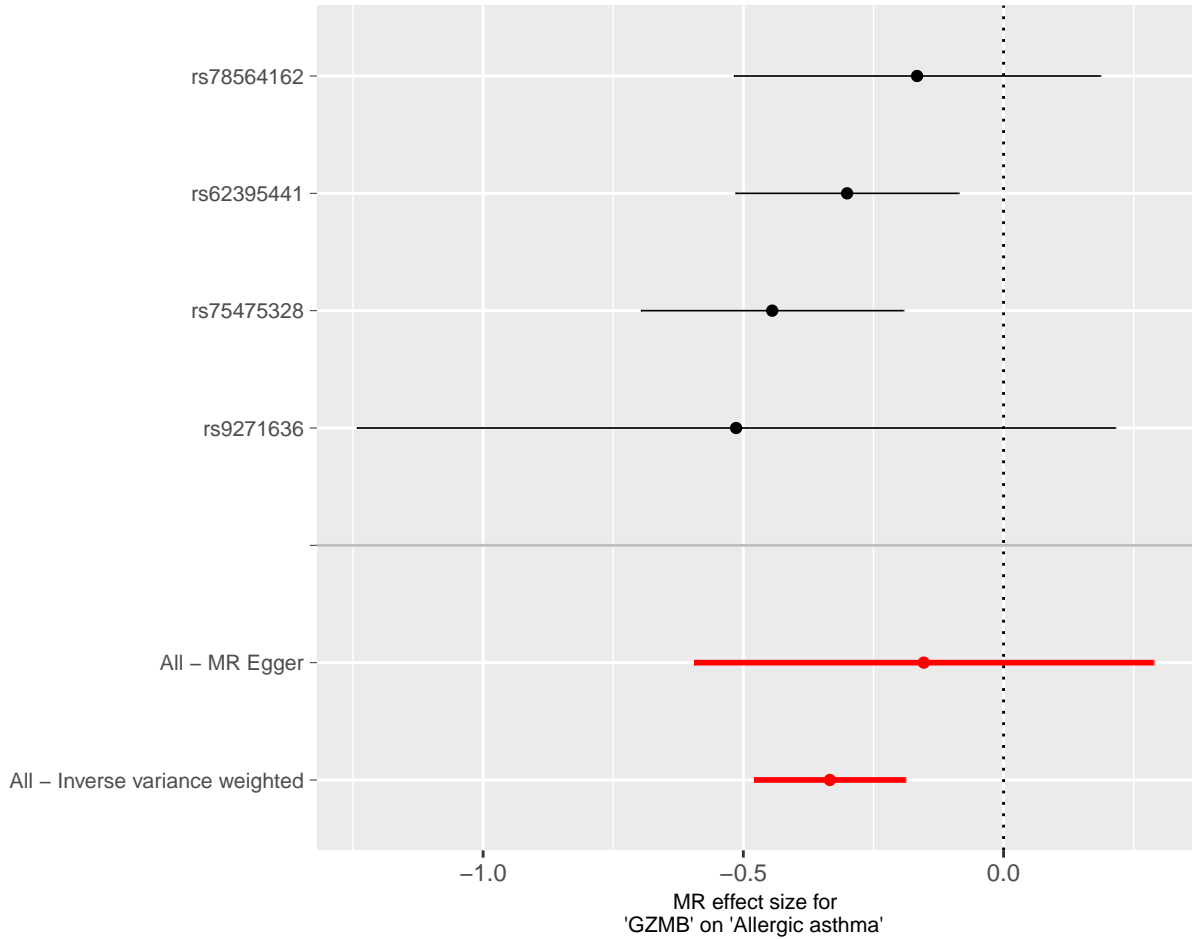

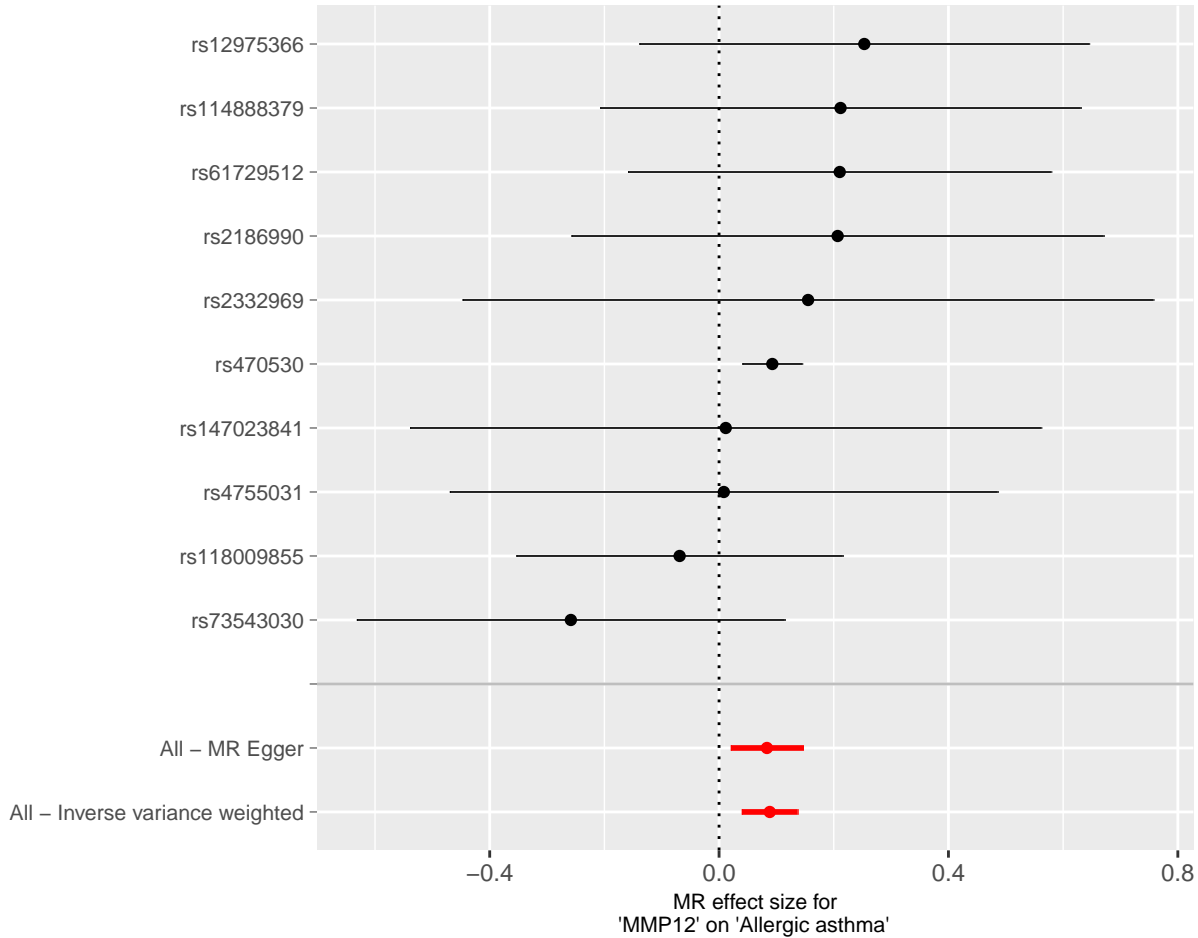

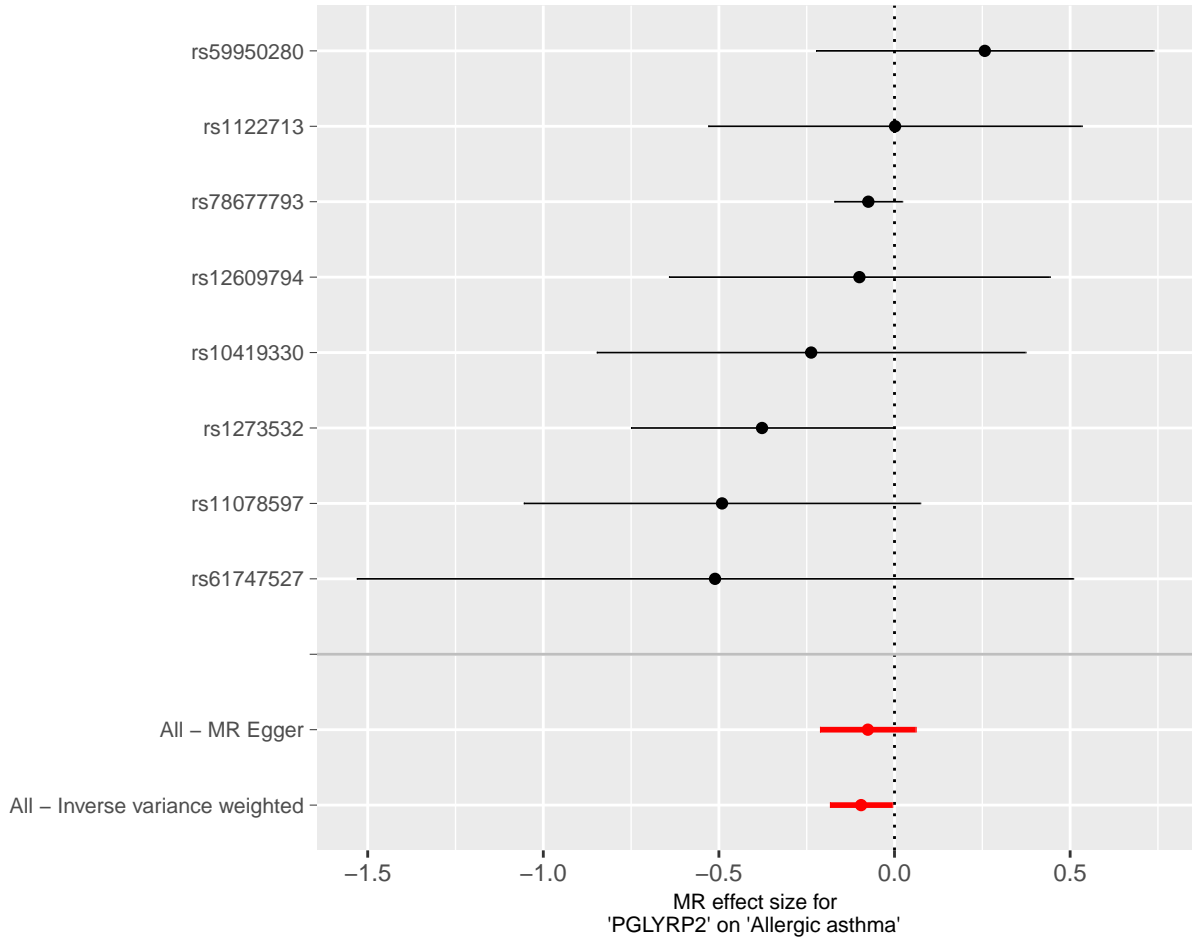

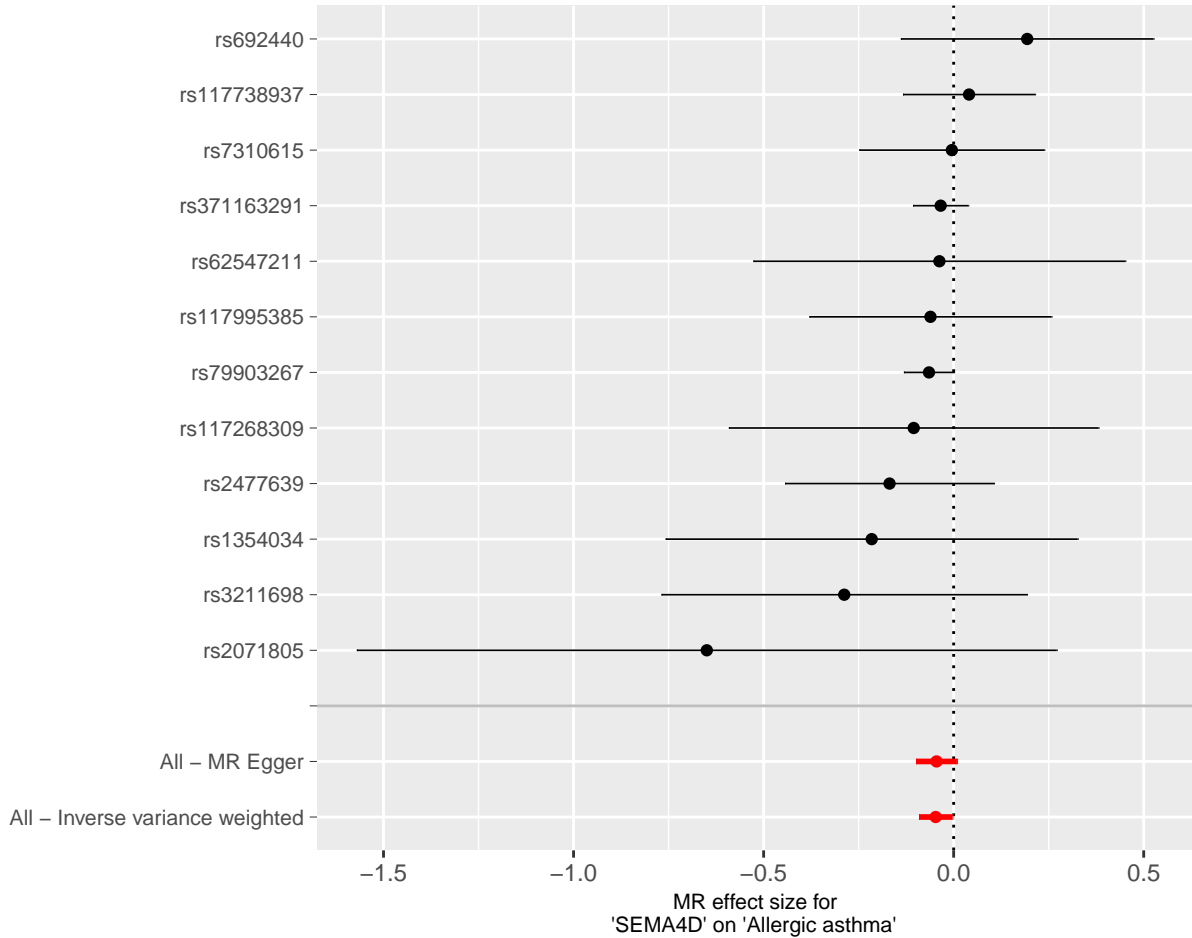

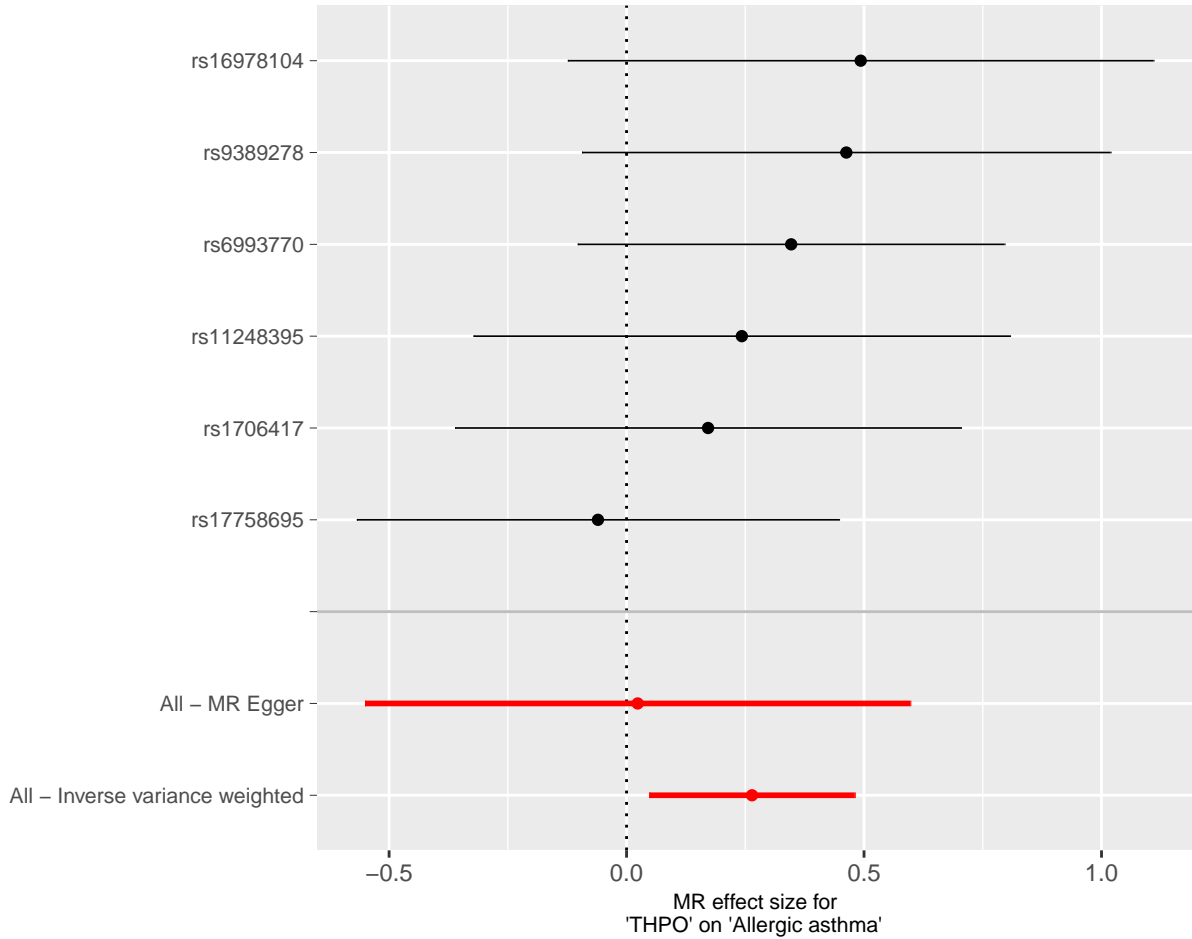

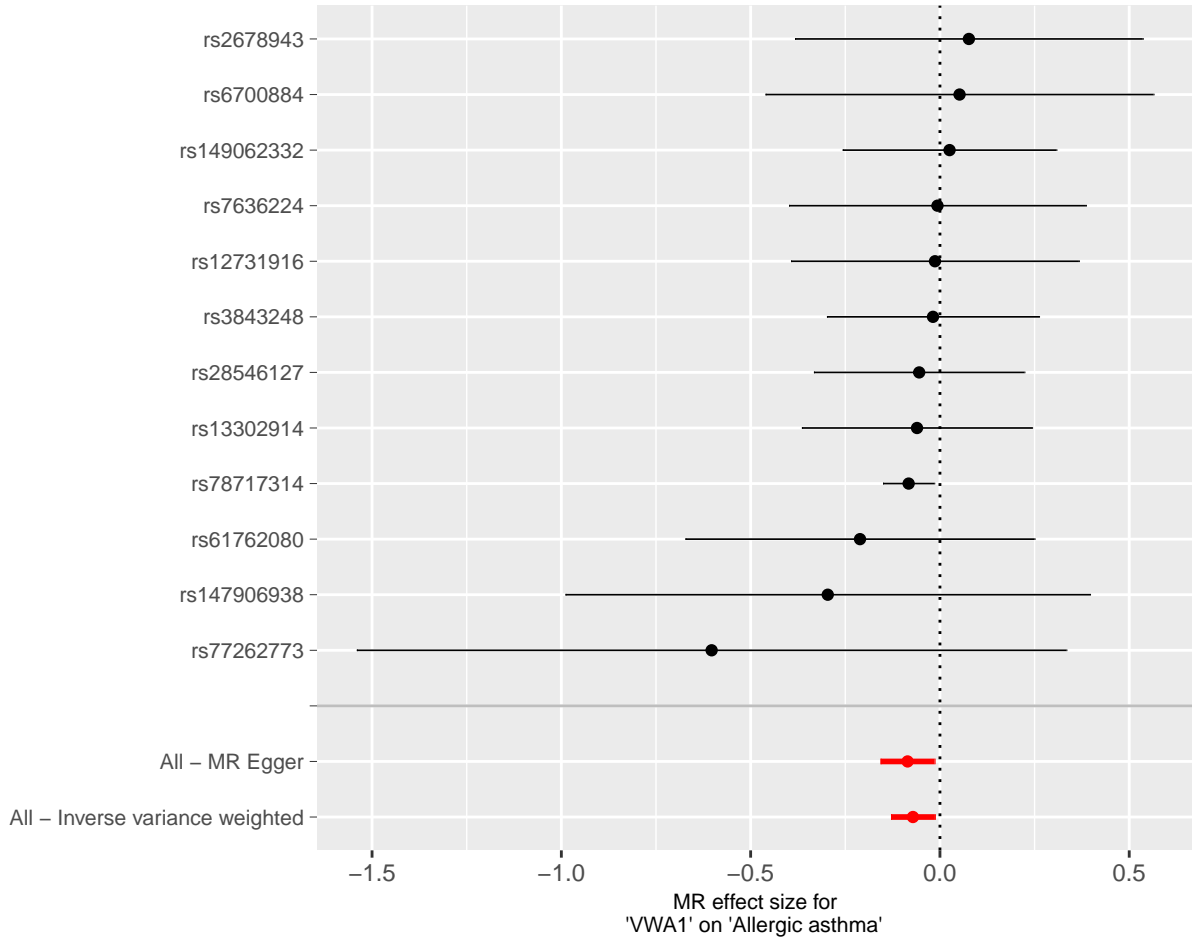

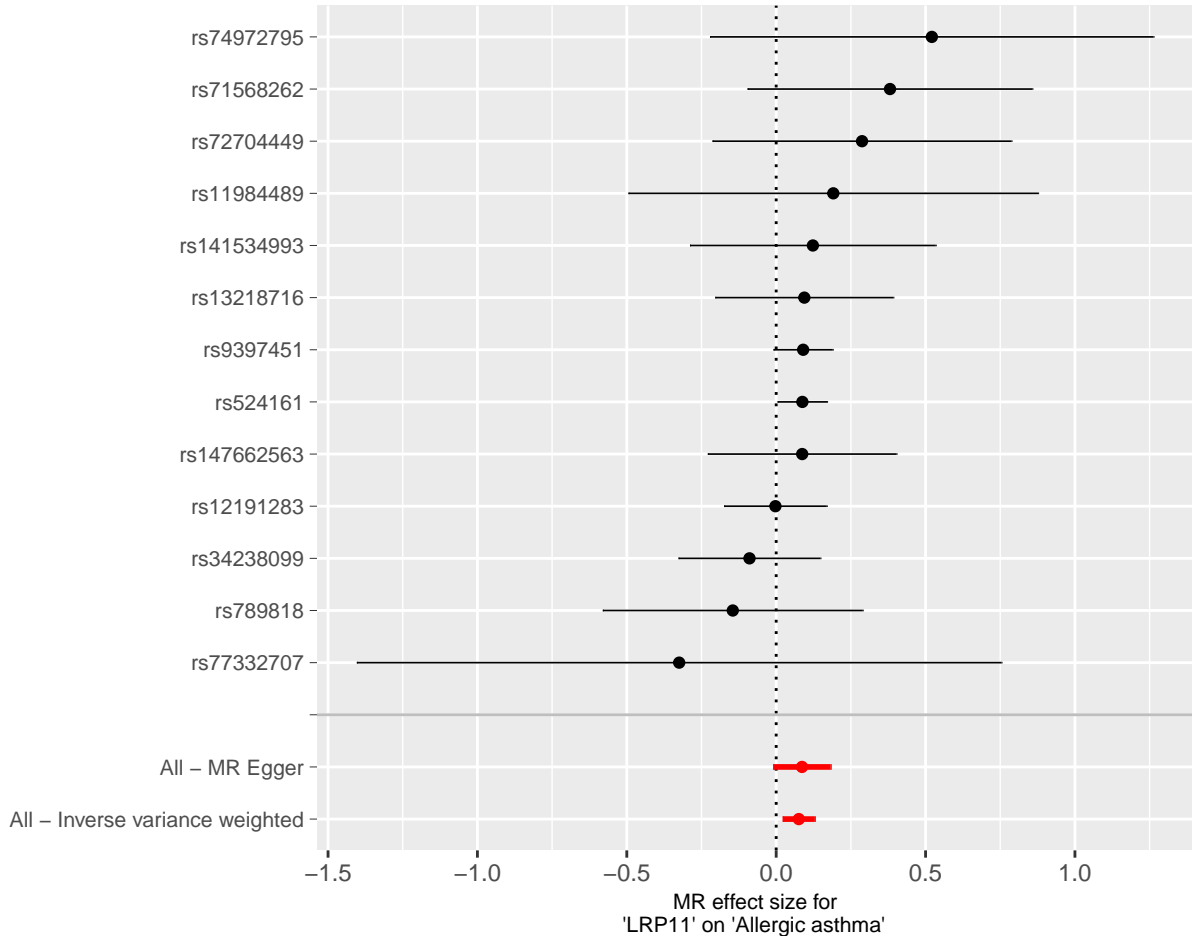

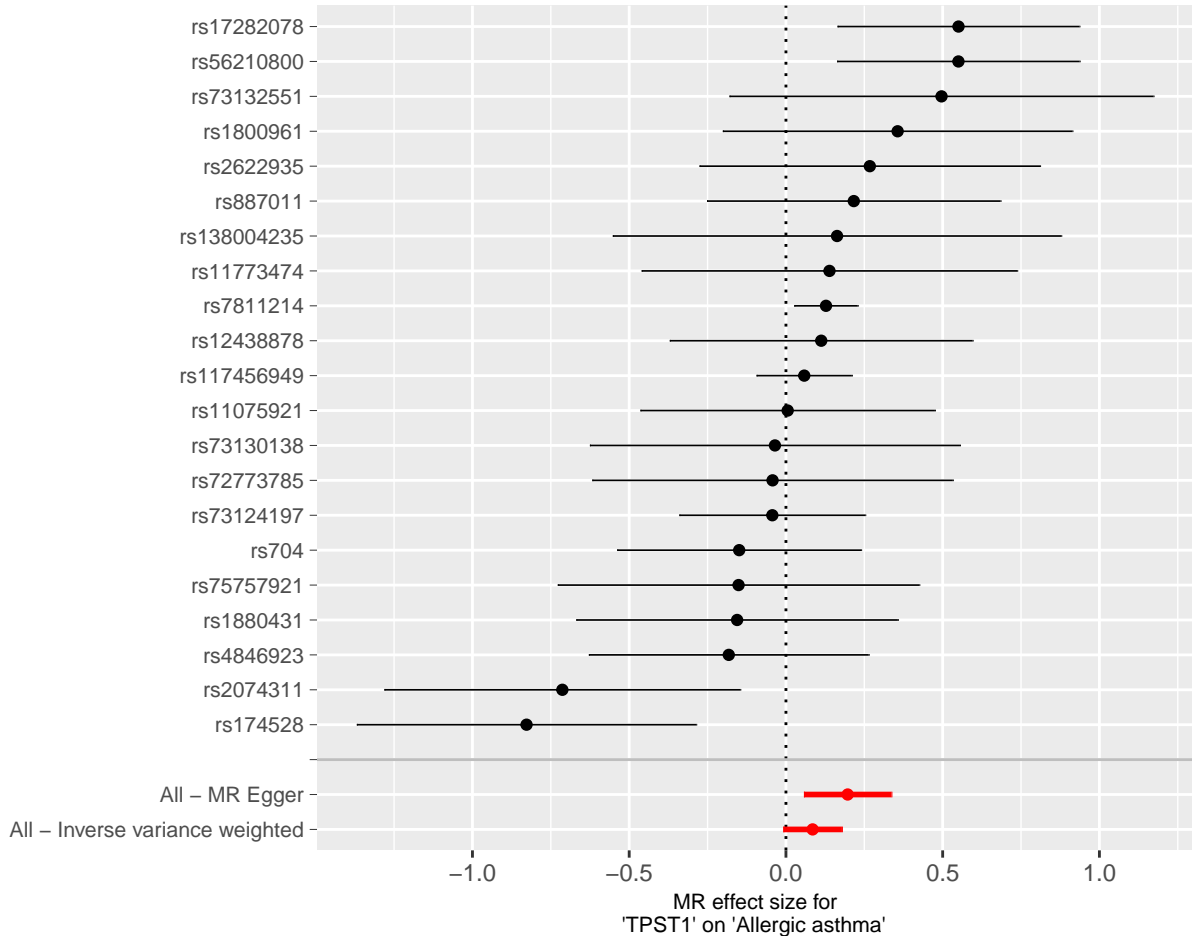

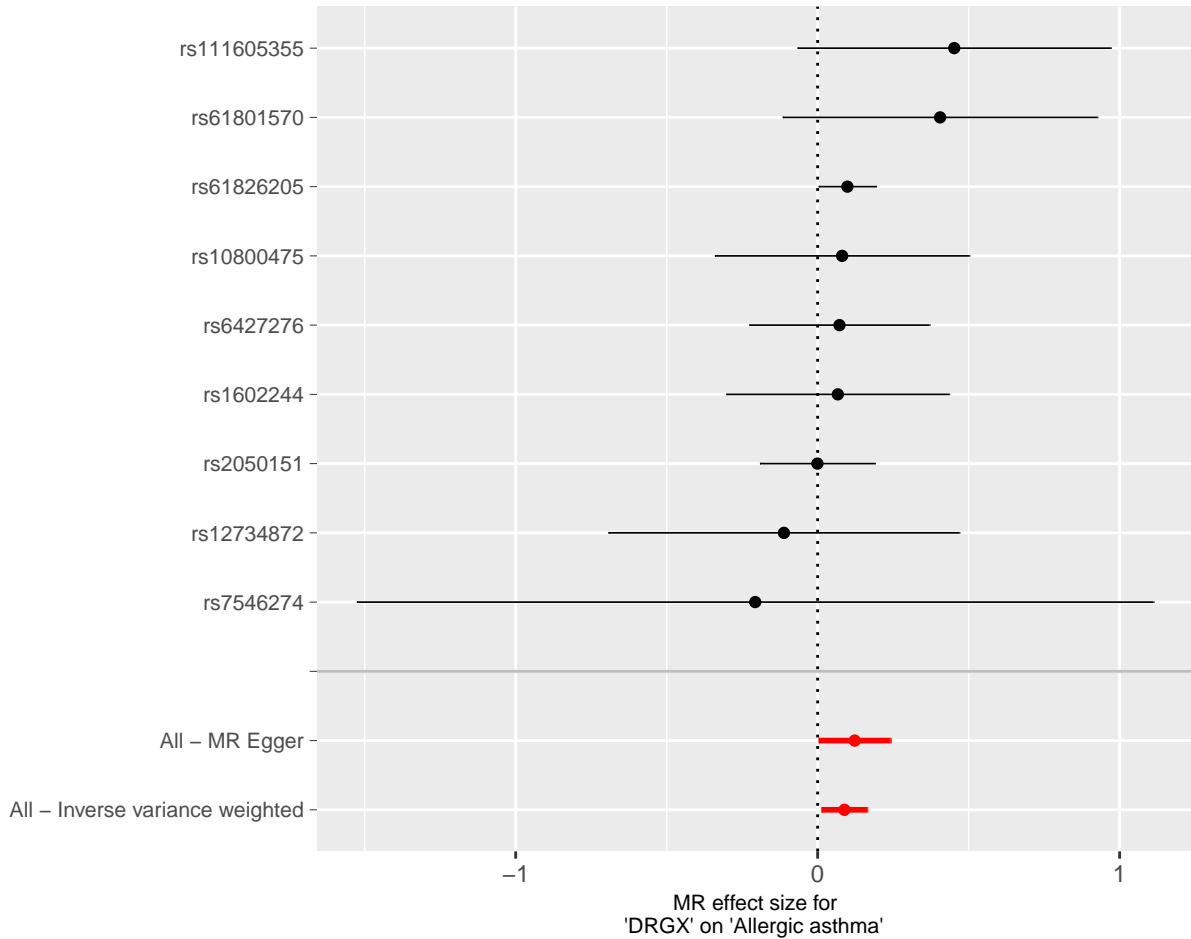

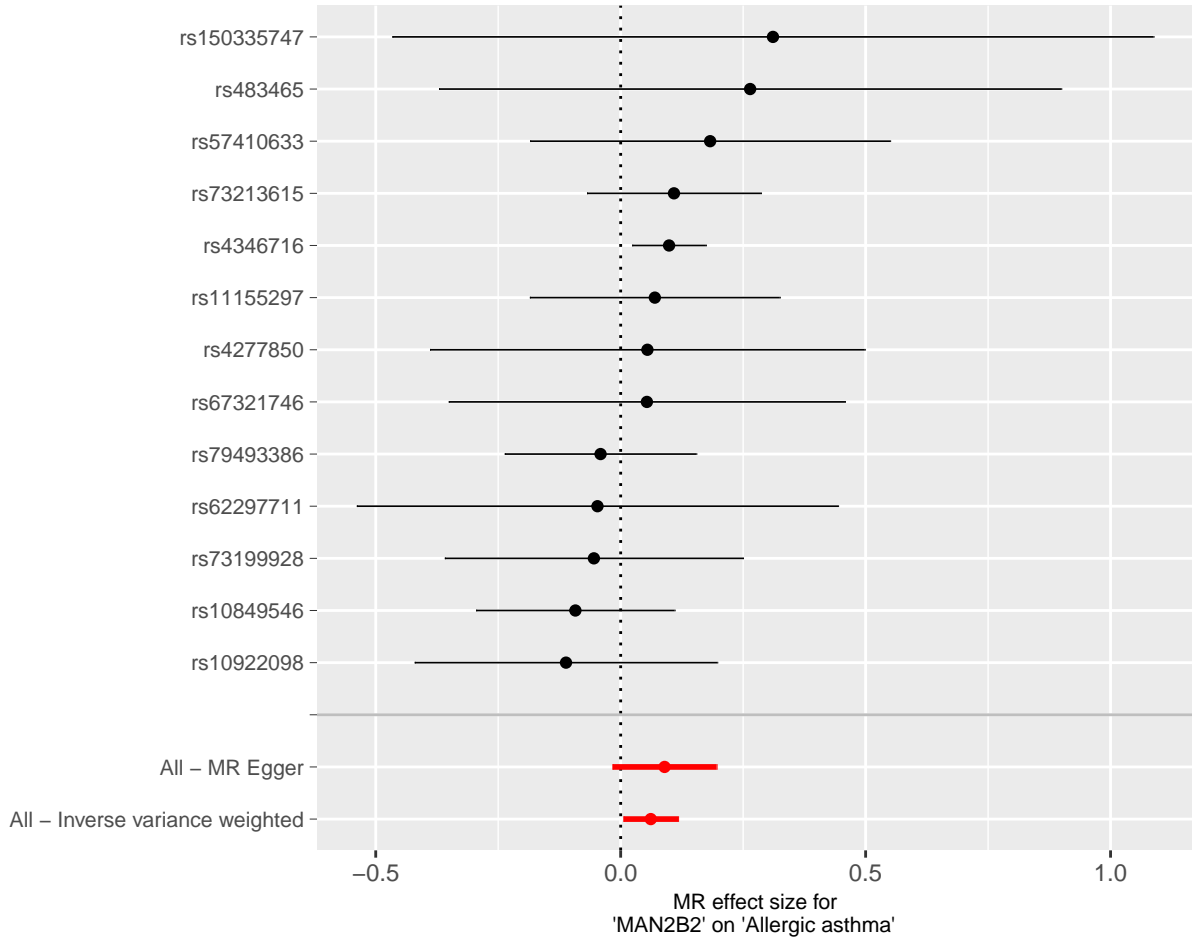

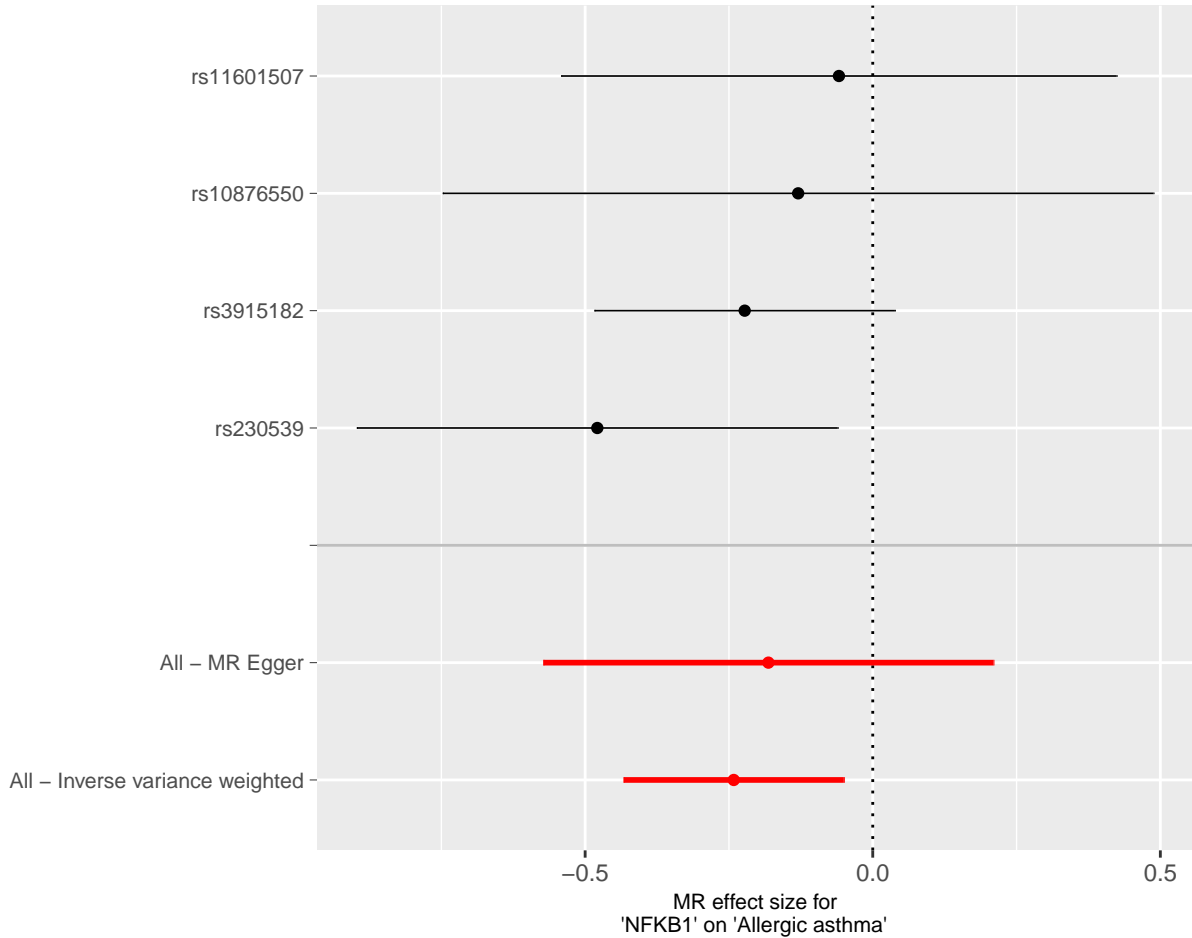

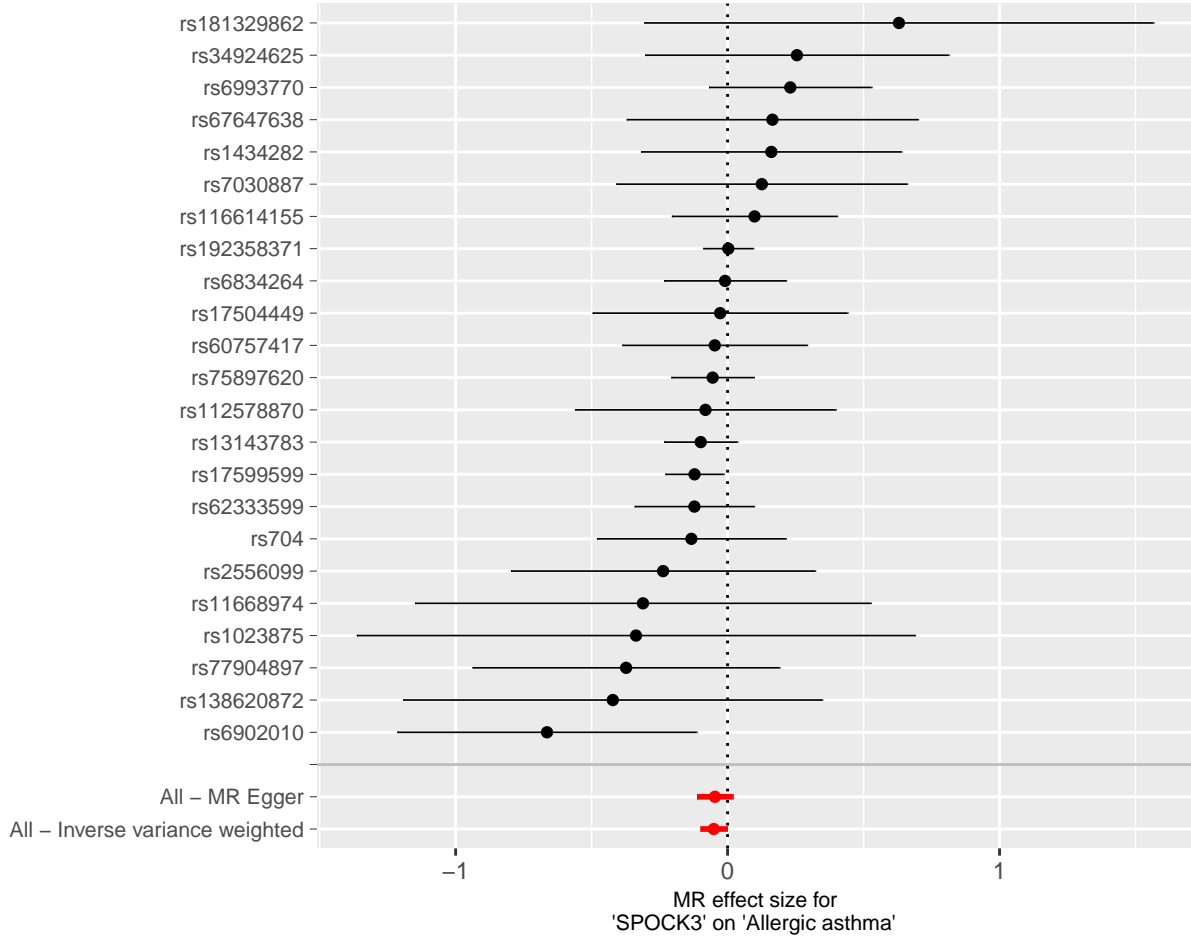

Supplement: Supplementary file 1 — Supplementary Material 1: Supplementary Materials 1. The MR results of BMI and allergic asthma. Supplementary Materials 2. The MR results of plasma proteins and allergic asthma. Supplementary Materials 3. The MR results of BMI and plasma proteins. Supplementary Materials 4. The MR results of mediation factor plasma proteins and allergic asthma. Supplementary Materials 5. The results of drug-targeted MR [file 41065_2025_376_MOESM1_ESM.zip › Supplementary Materials/S4 The MR results of mediation factor plasma proteins and allergic asthma/S4 plasma proteins-Allergic asthma.forest.pdf]

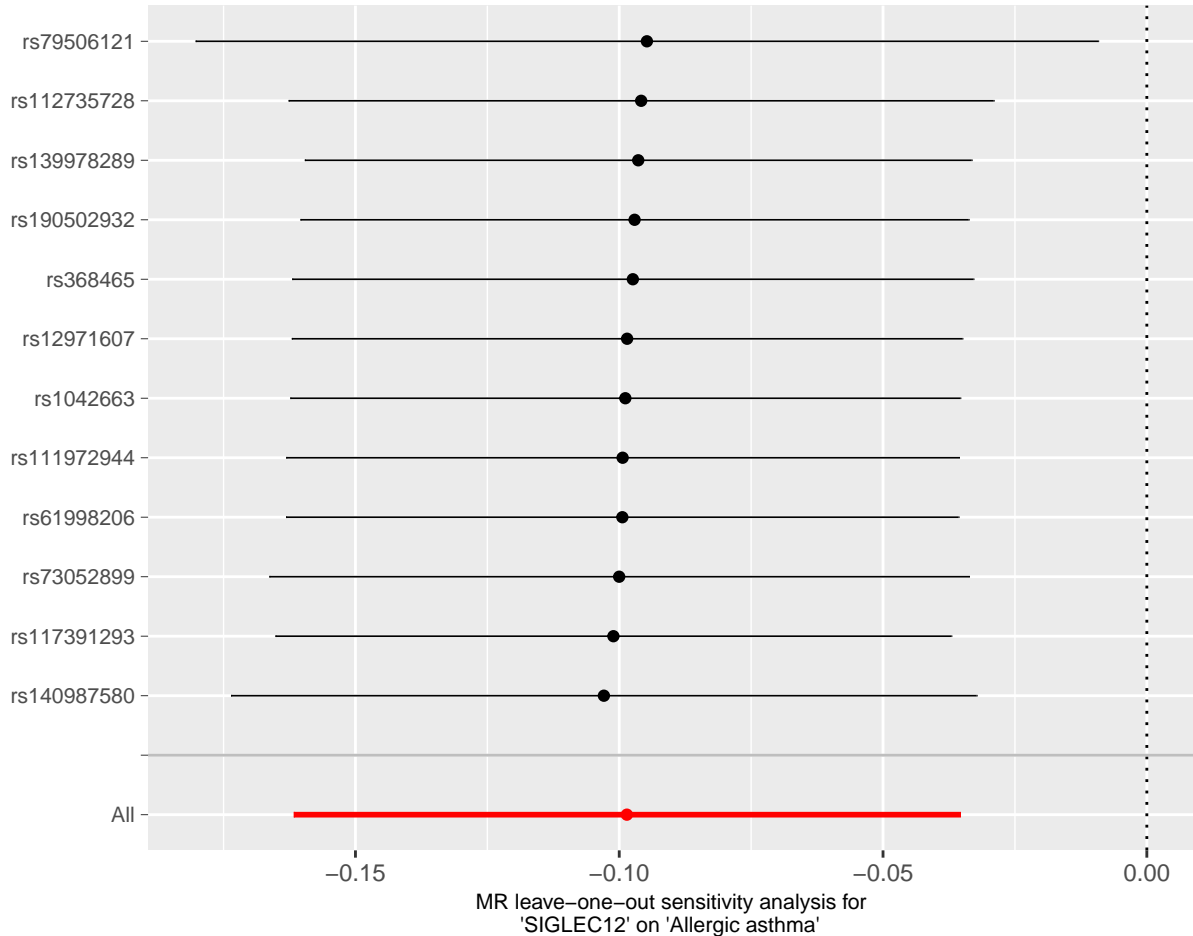

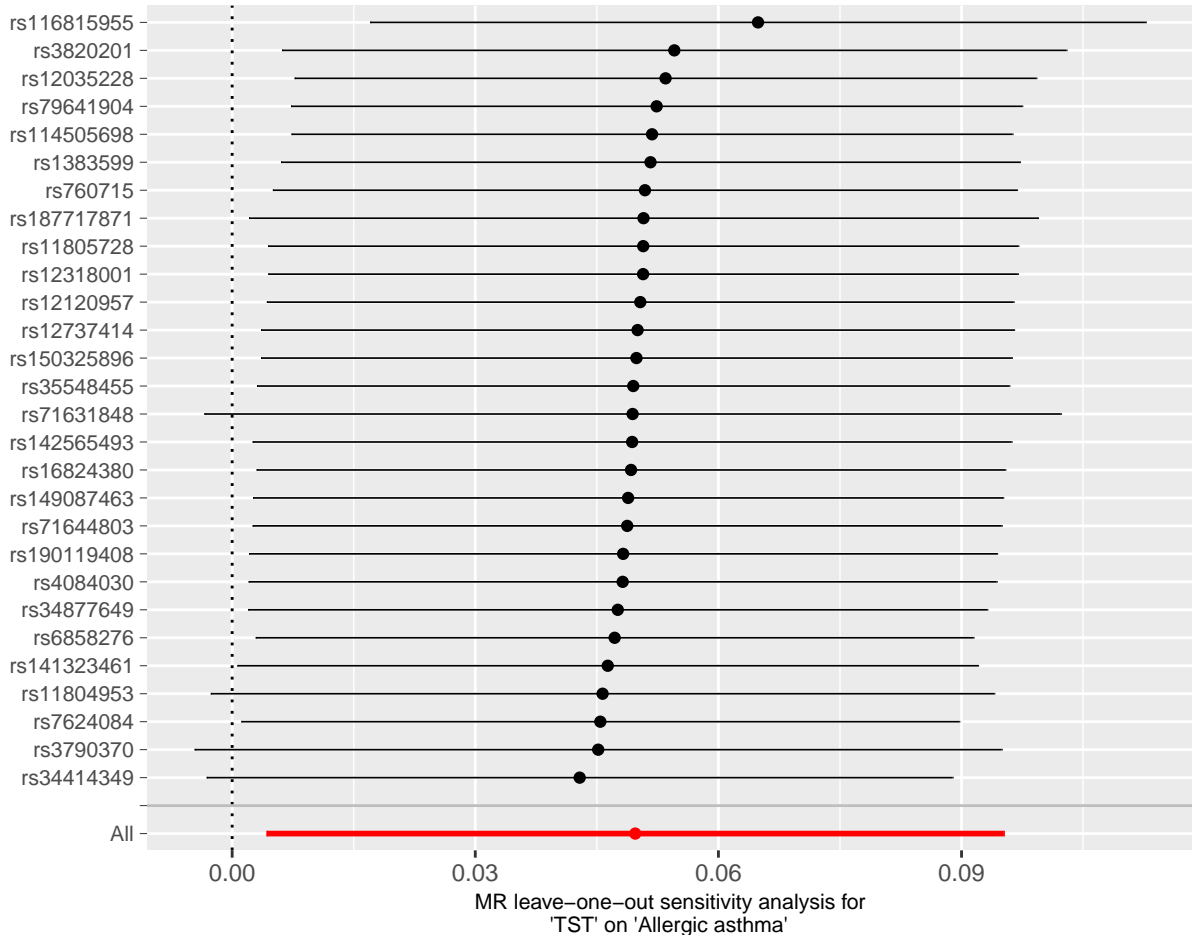

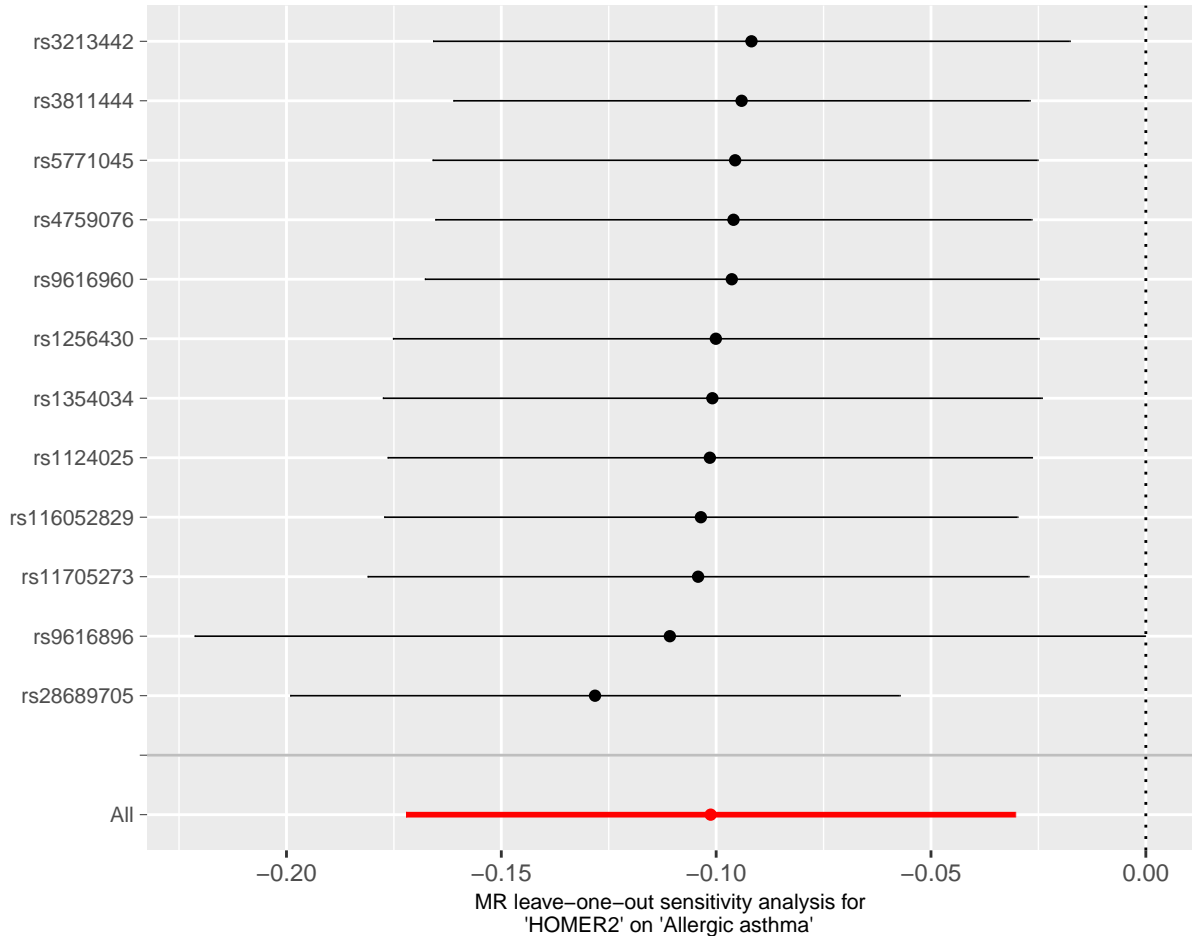

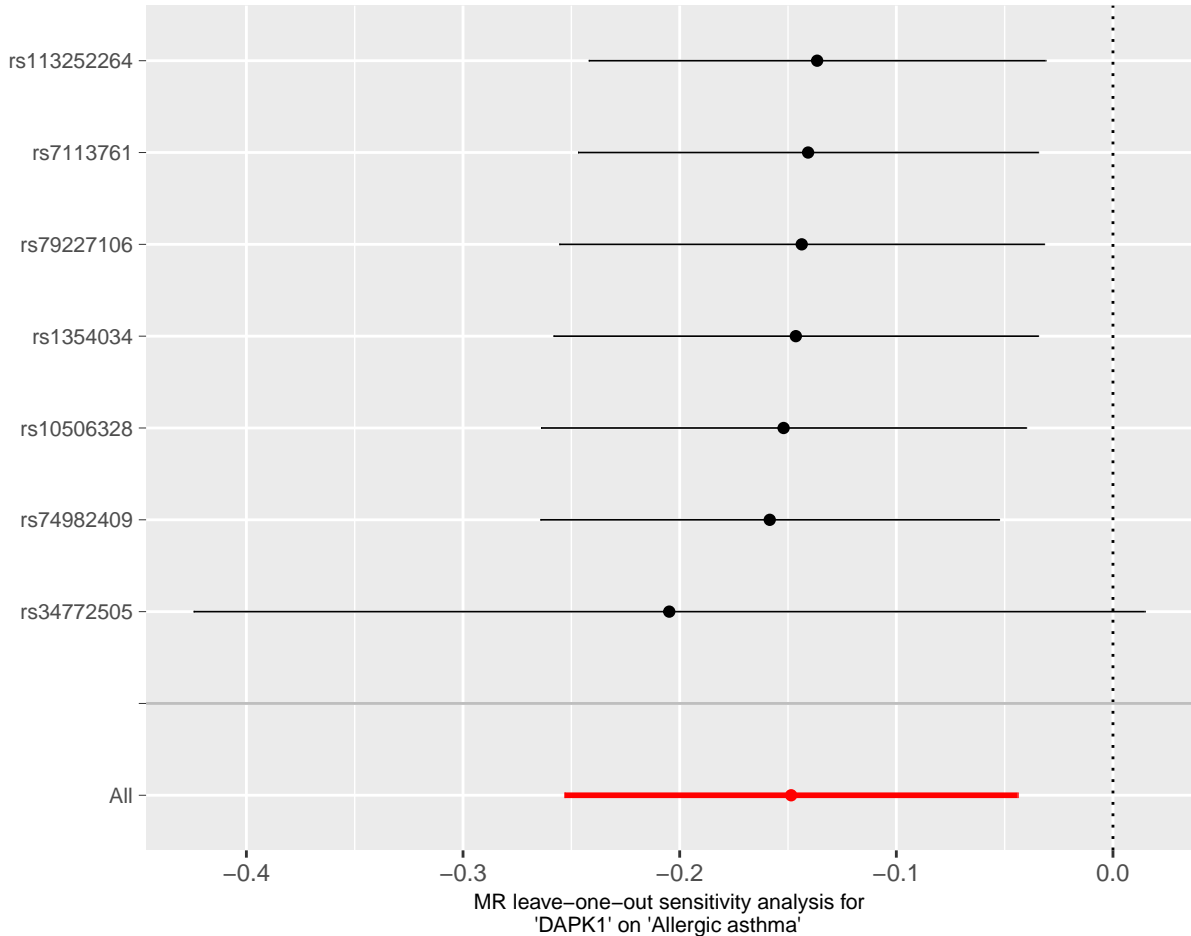

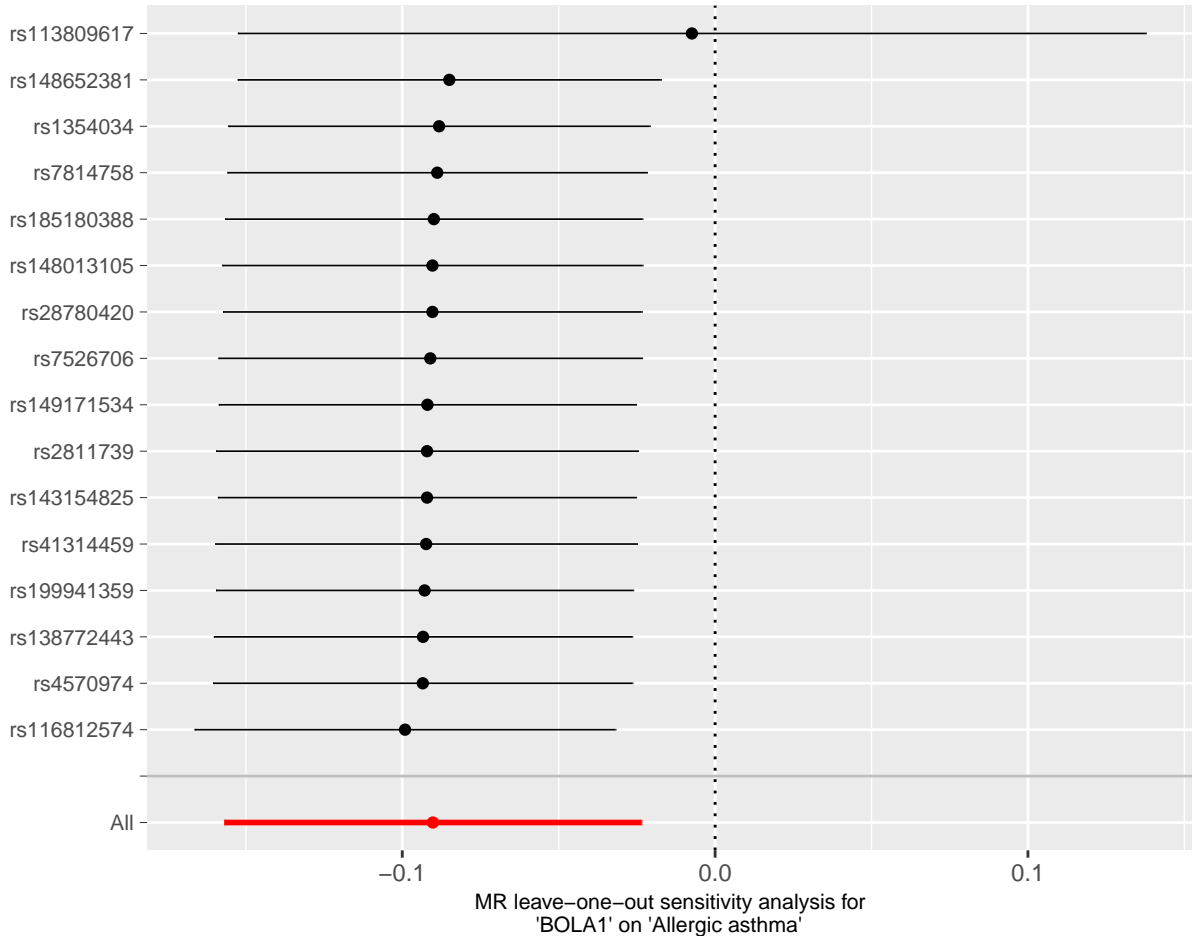

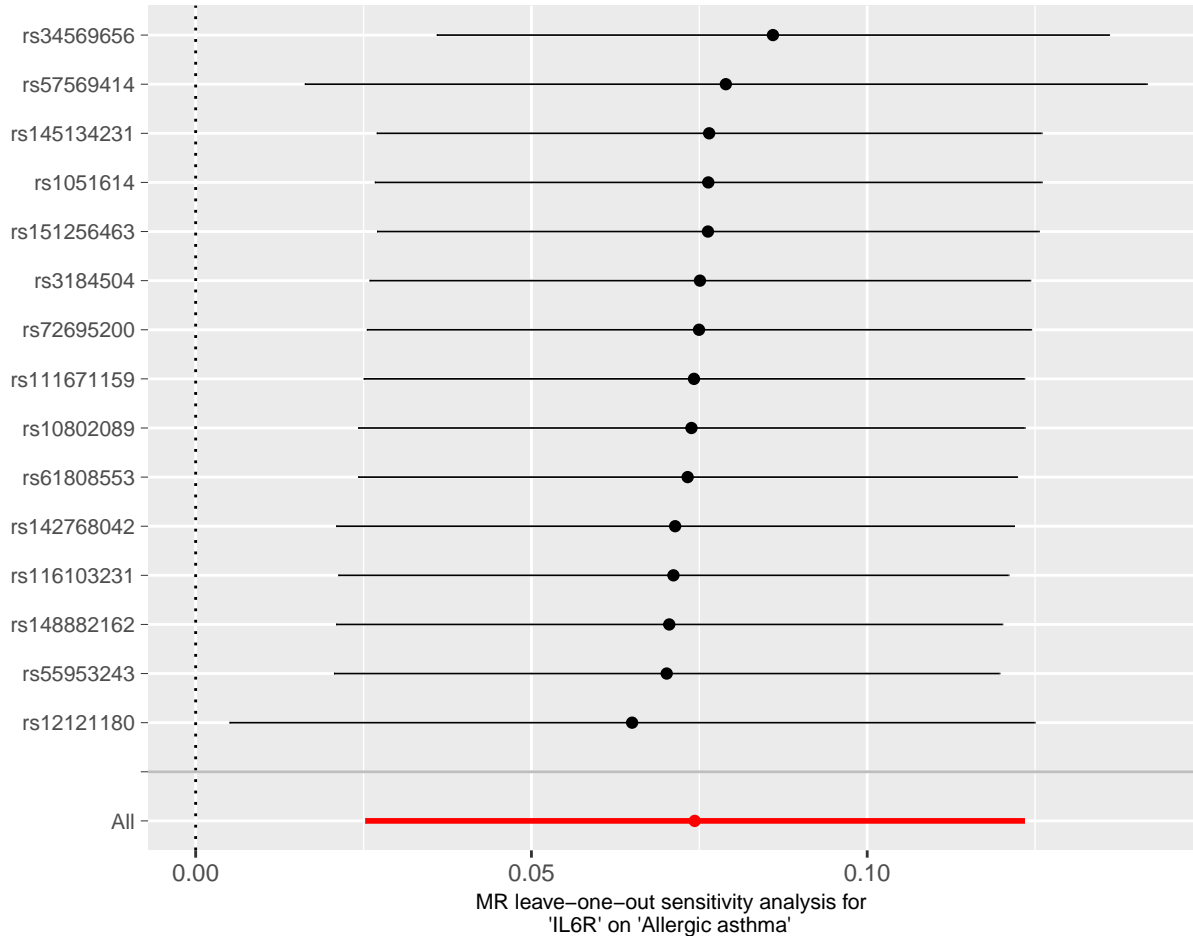

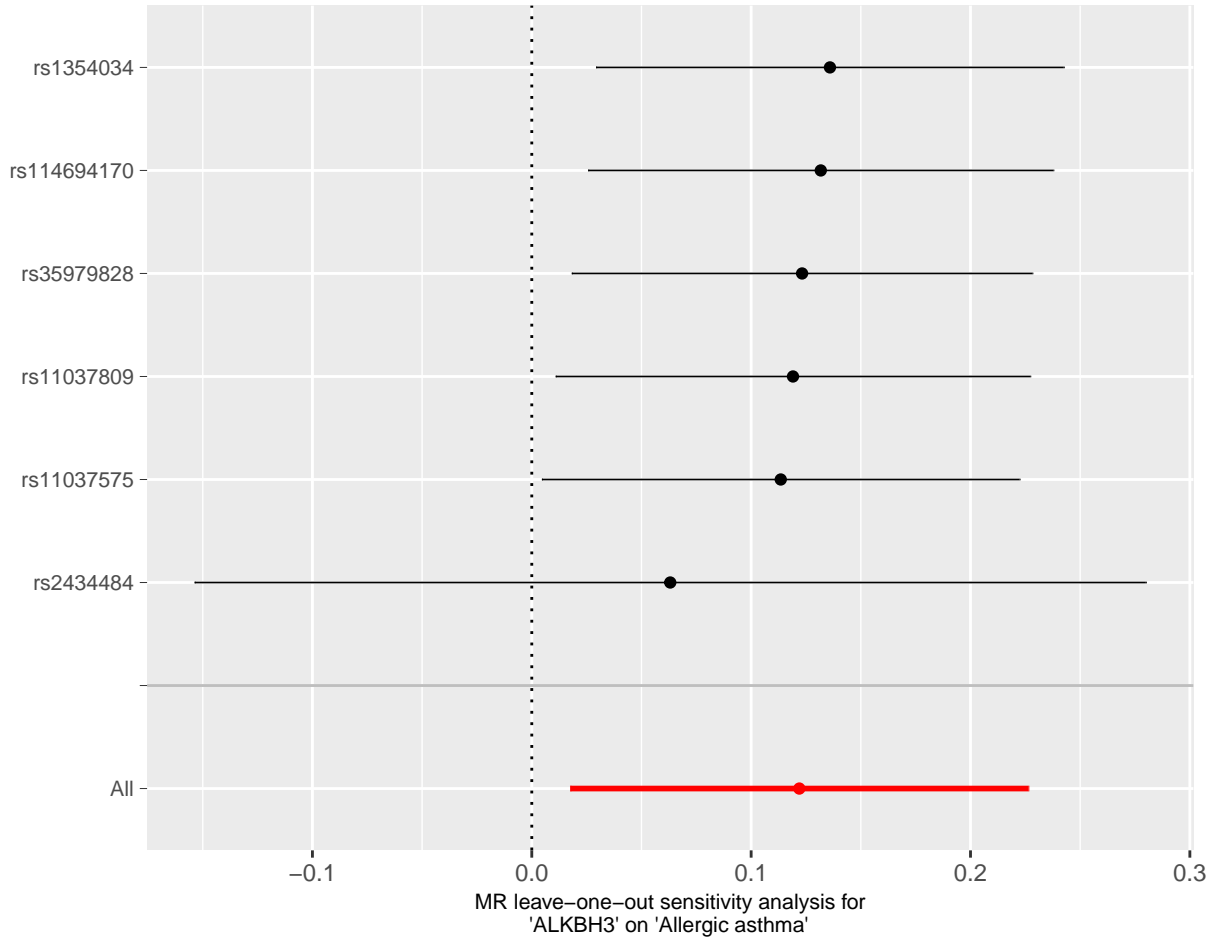

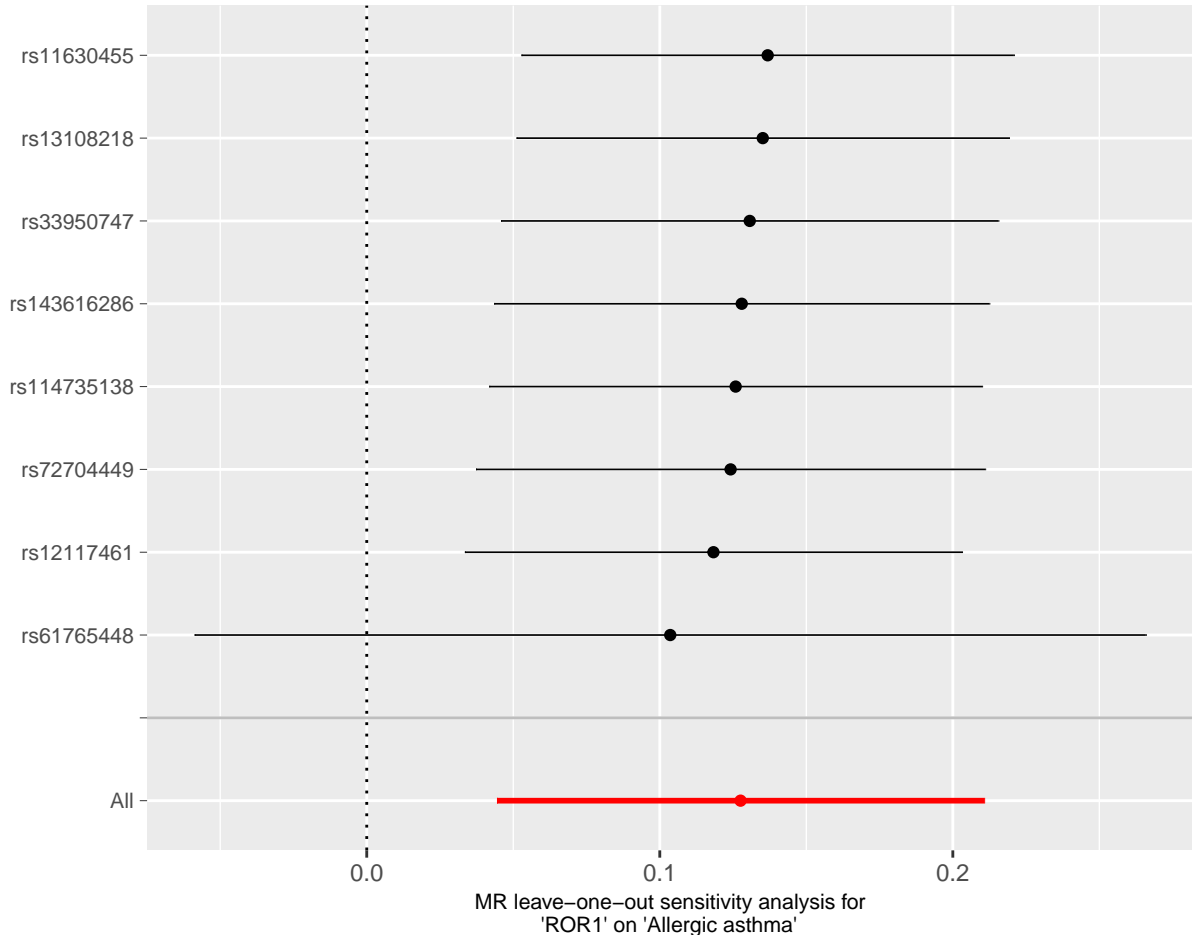

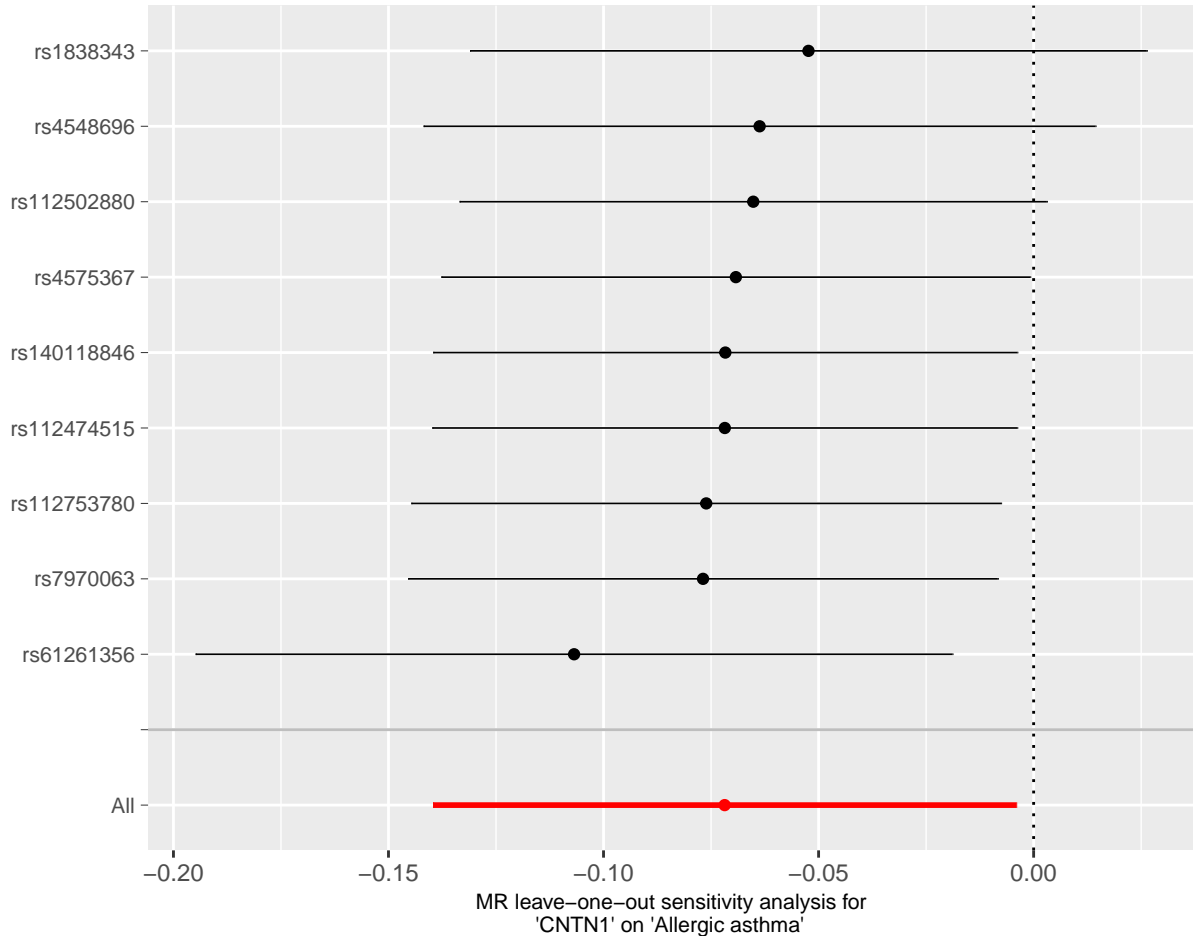

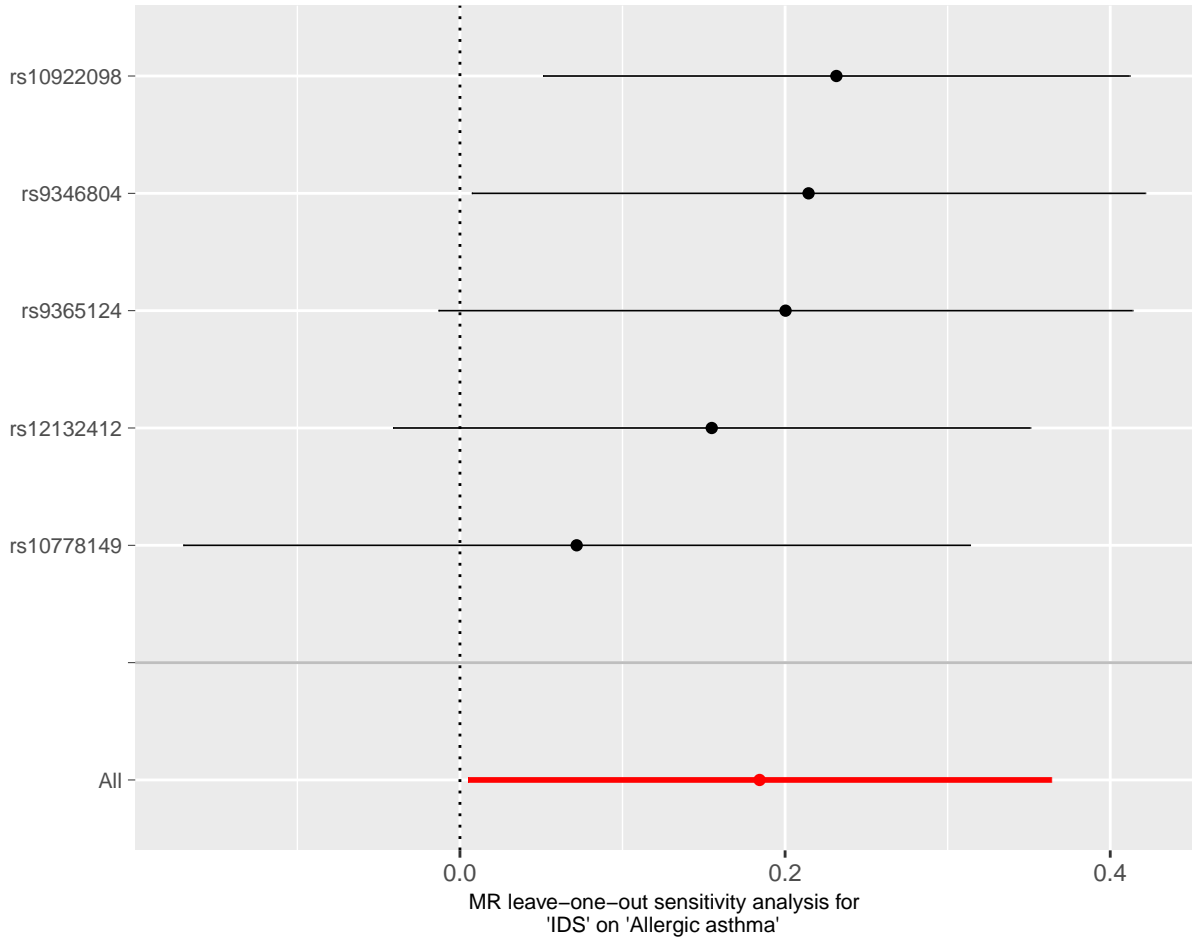

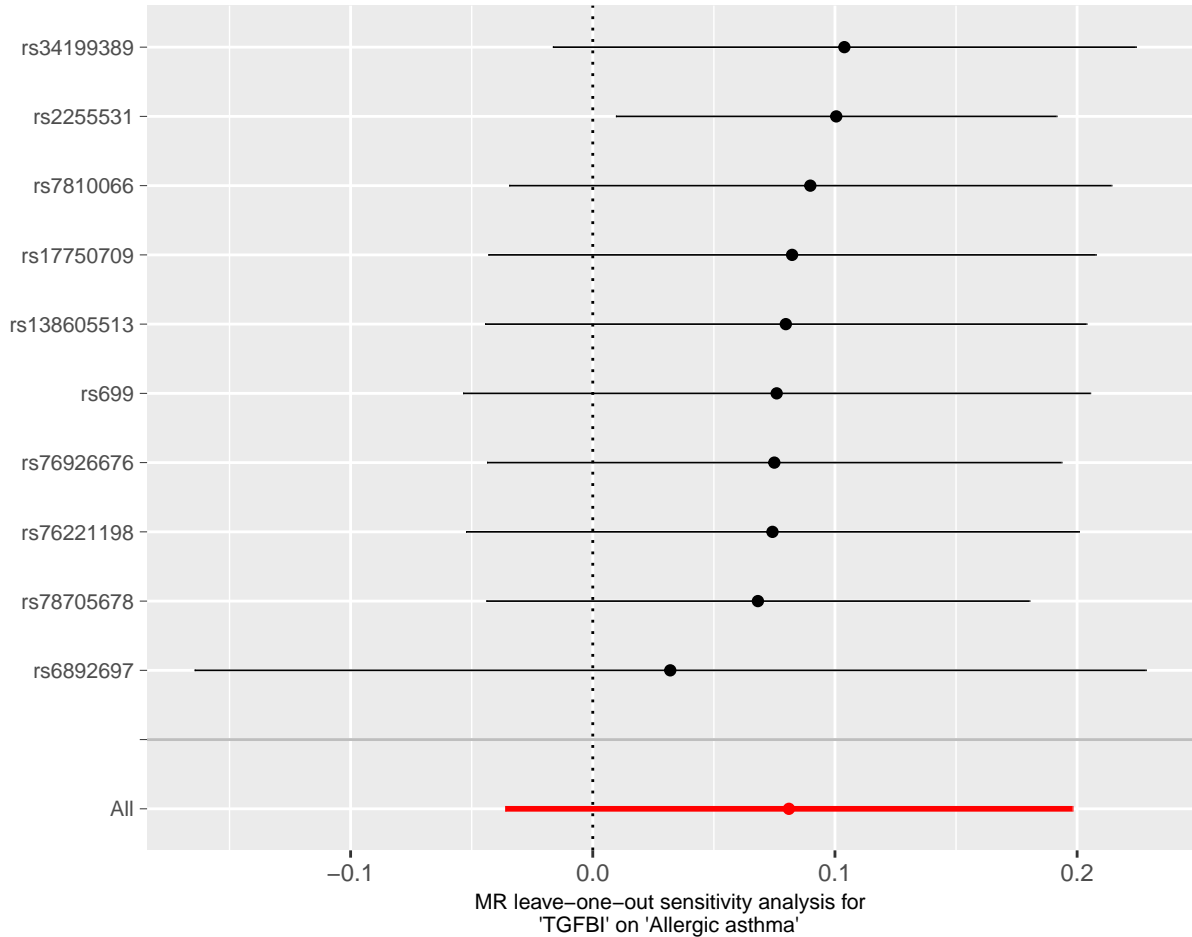

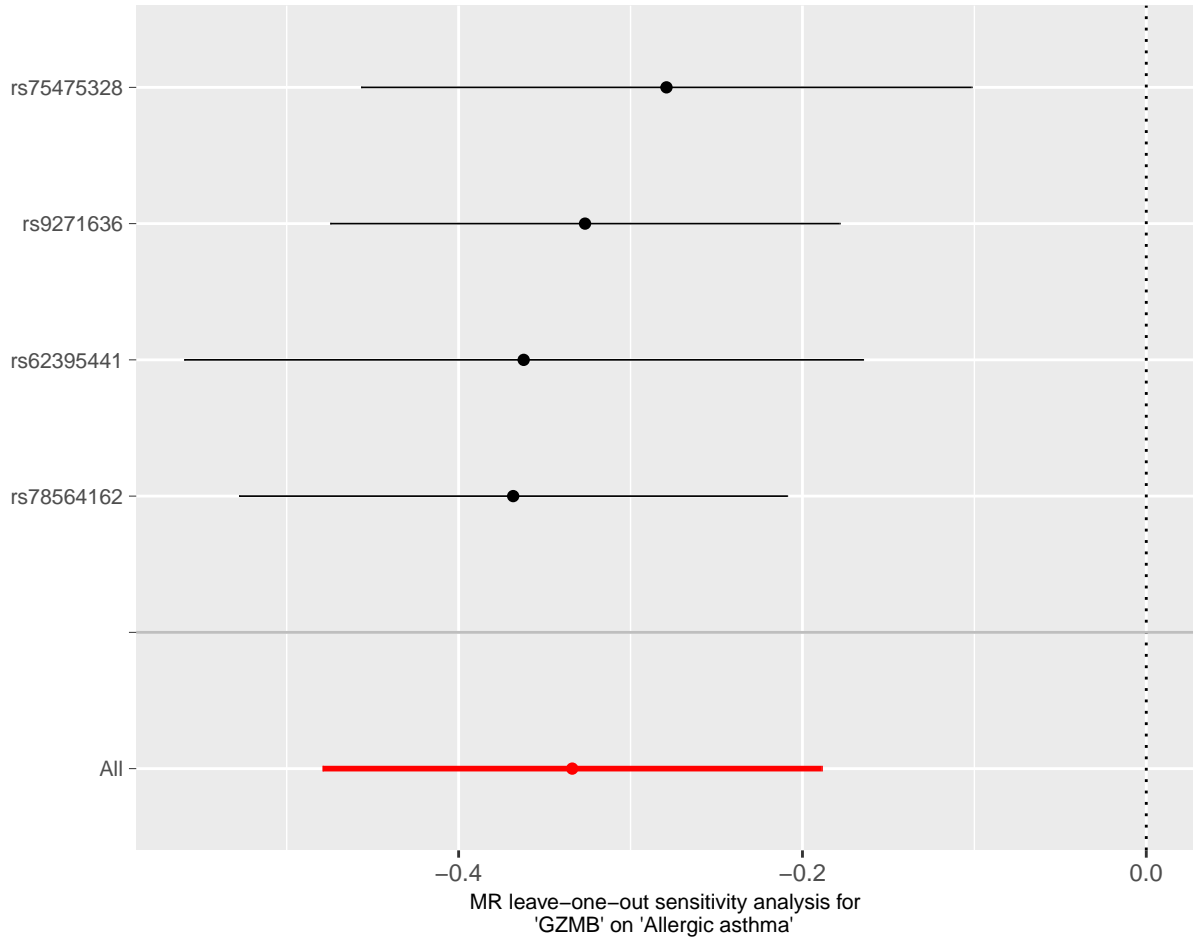

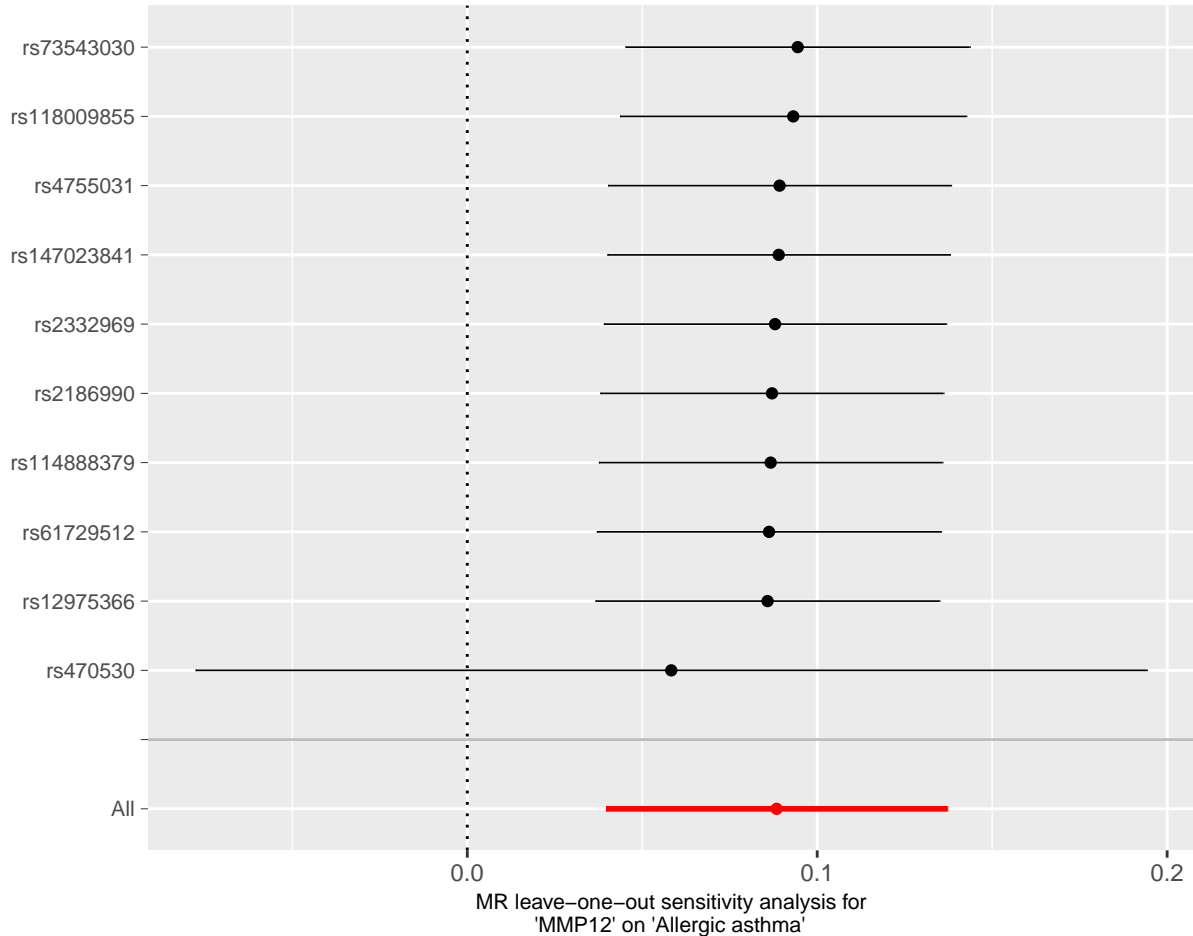

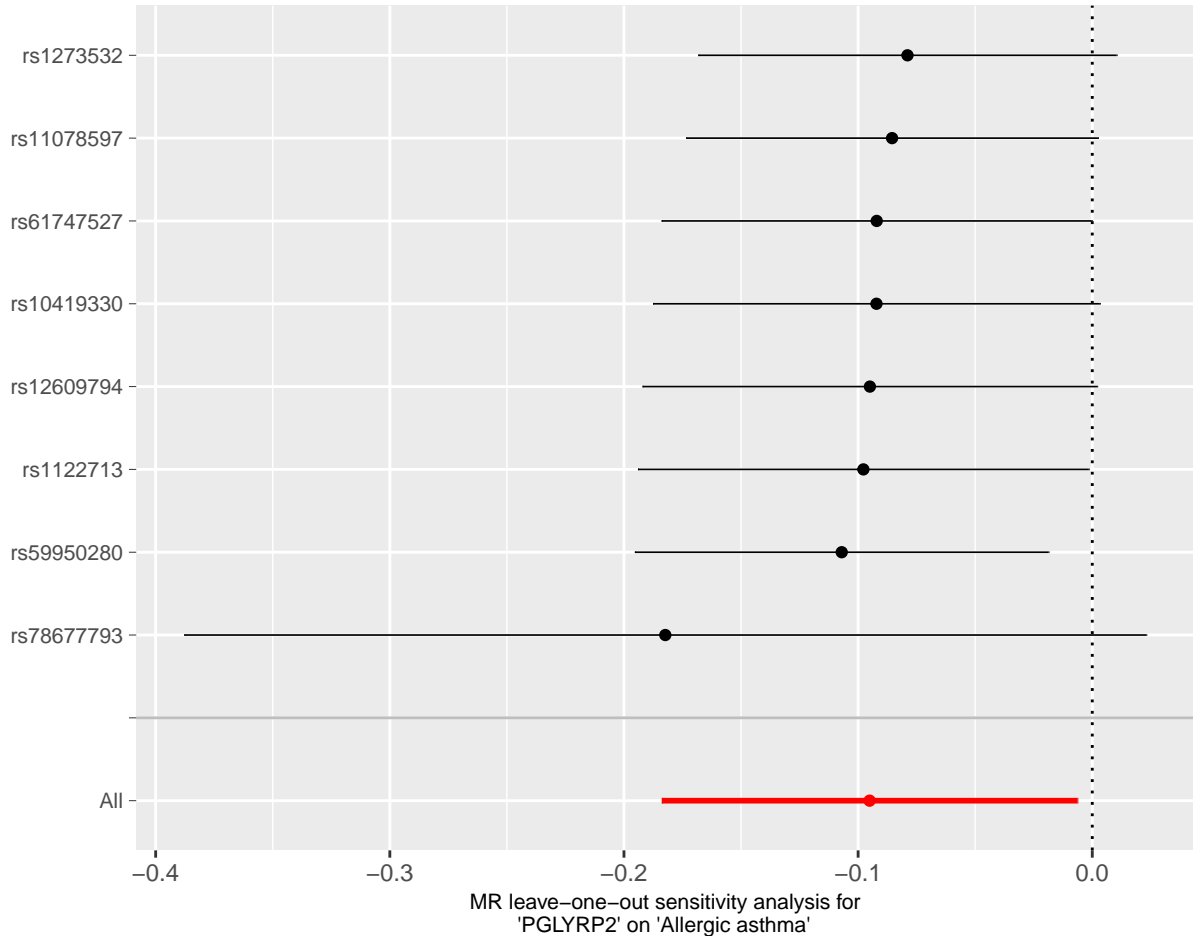

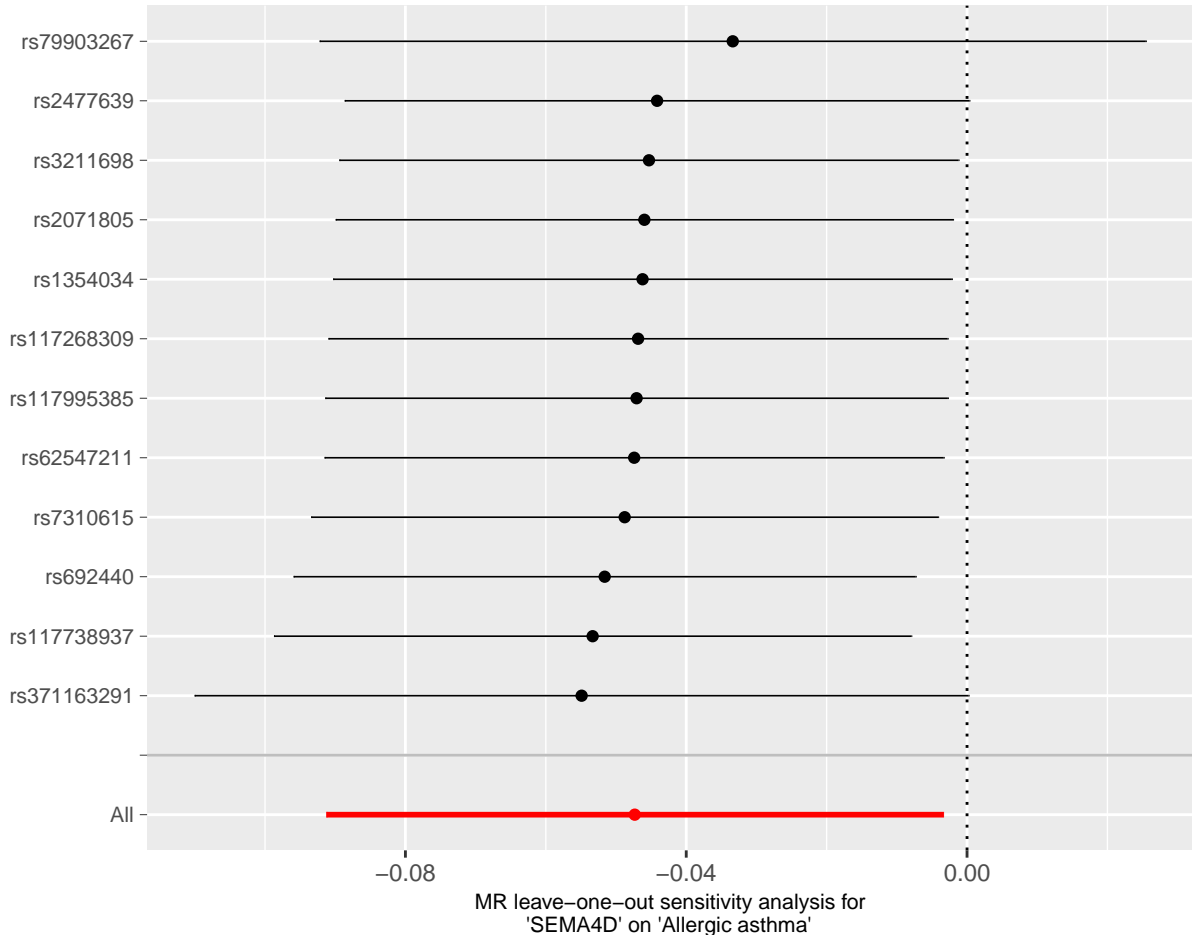

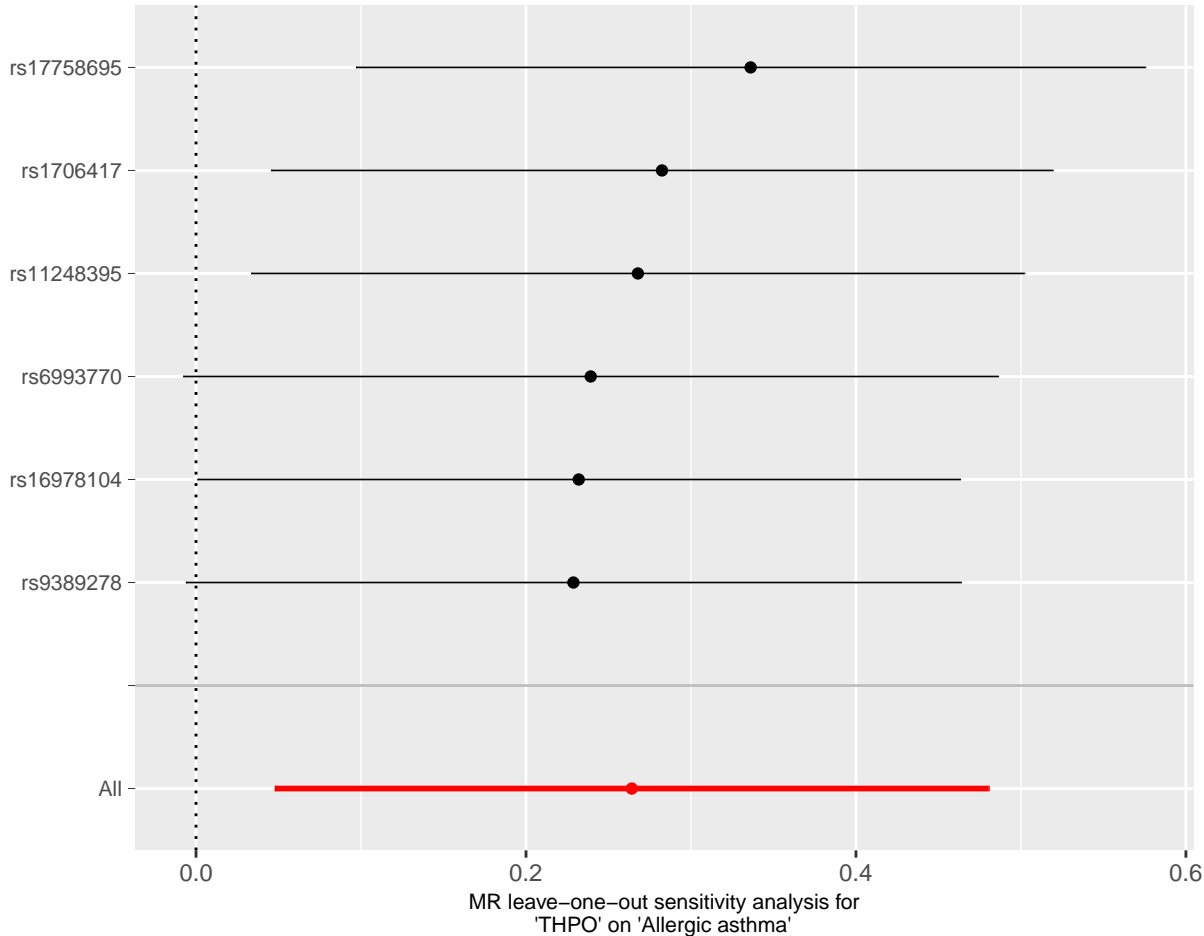

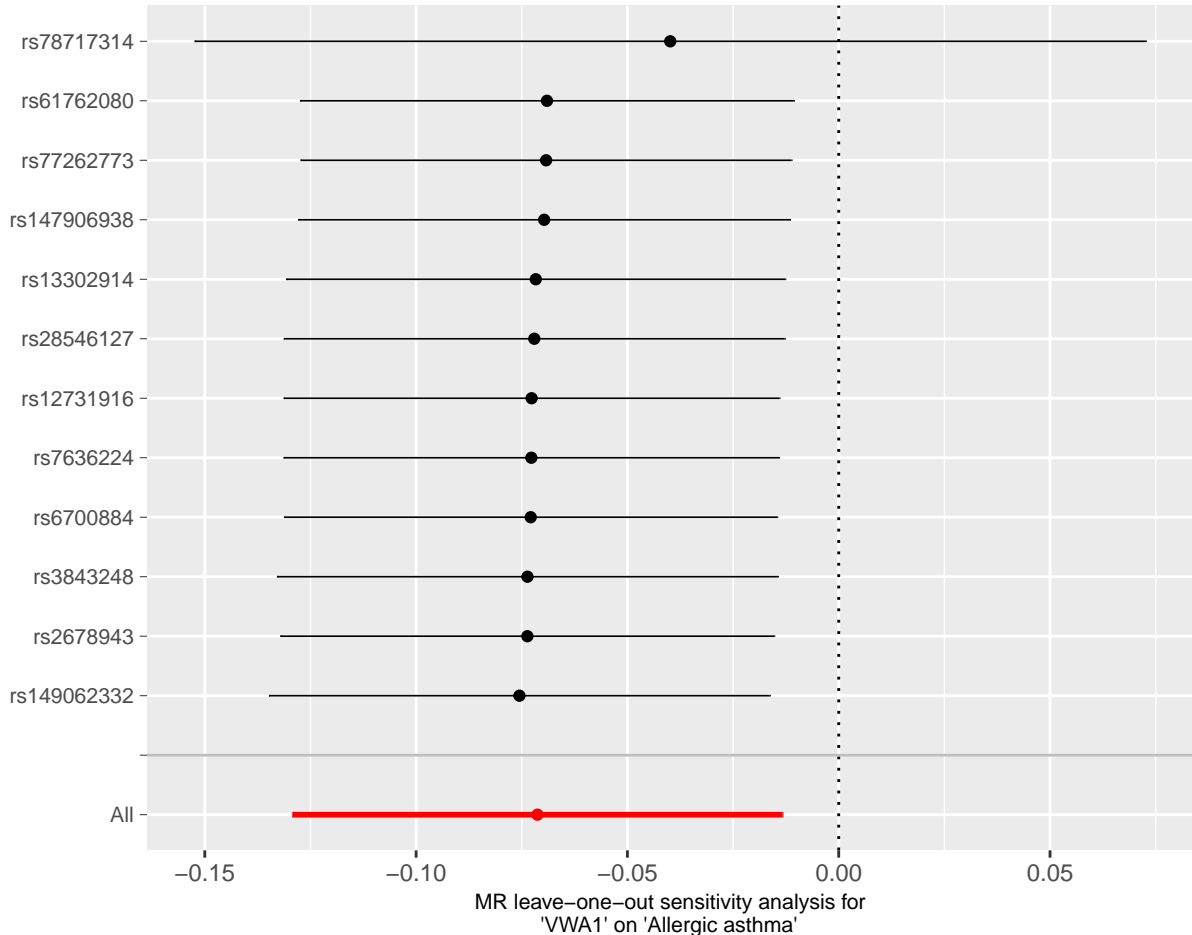

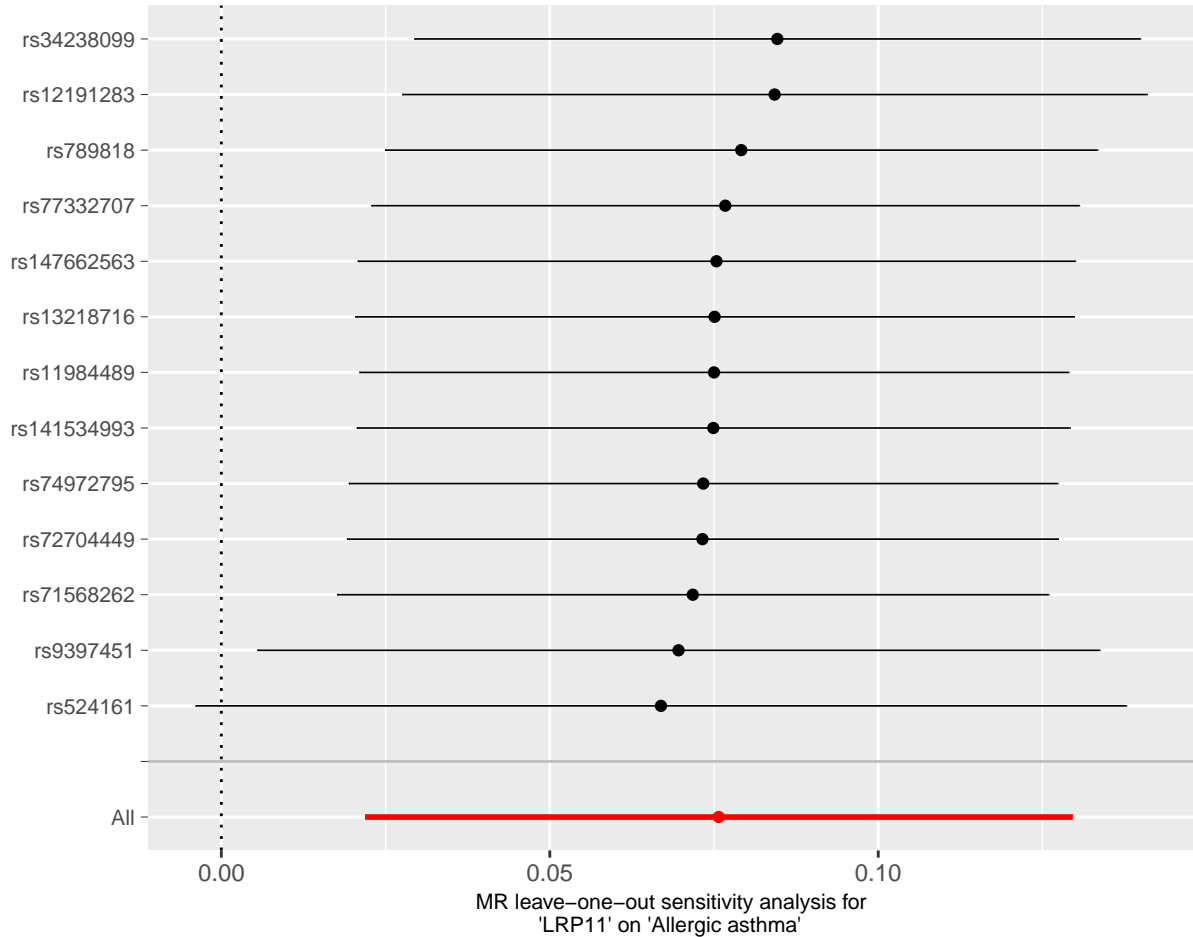

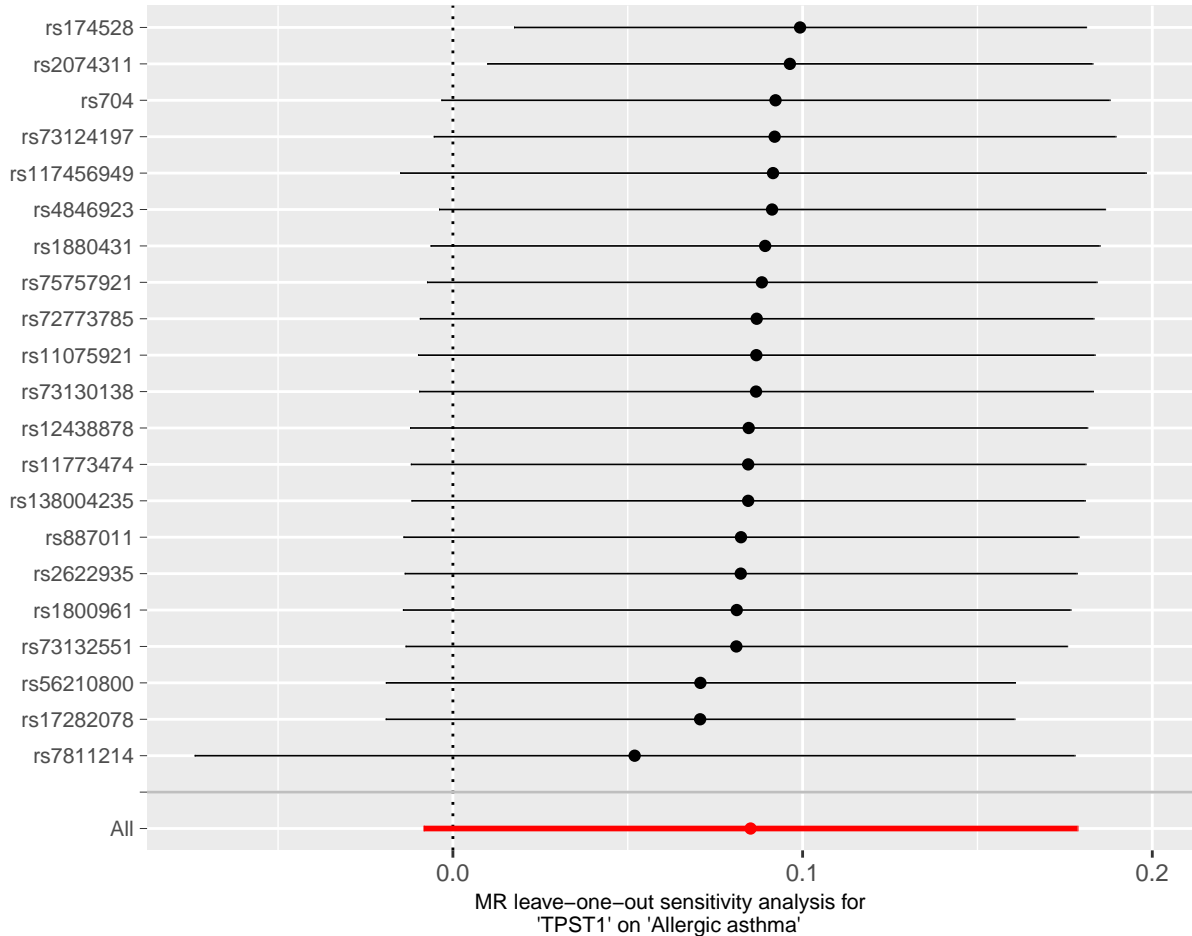

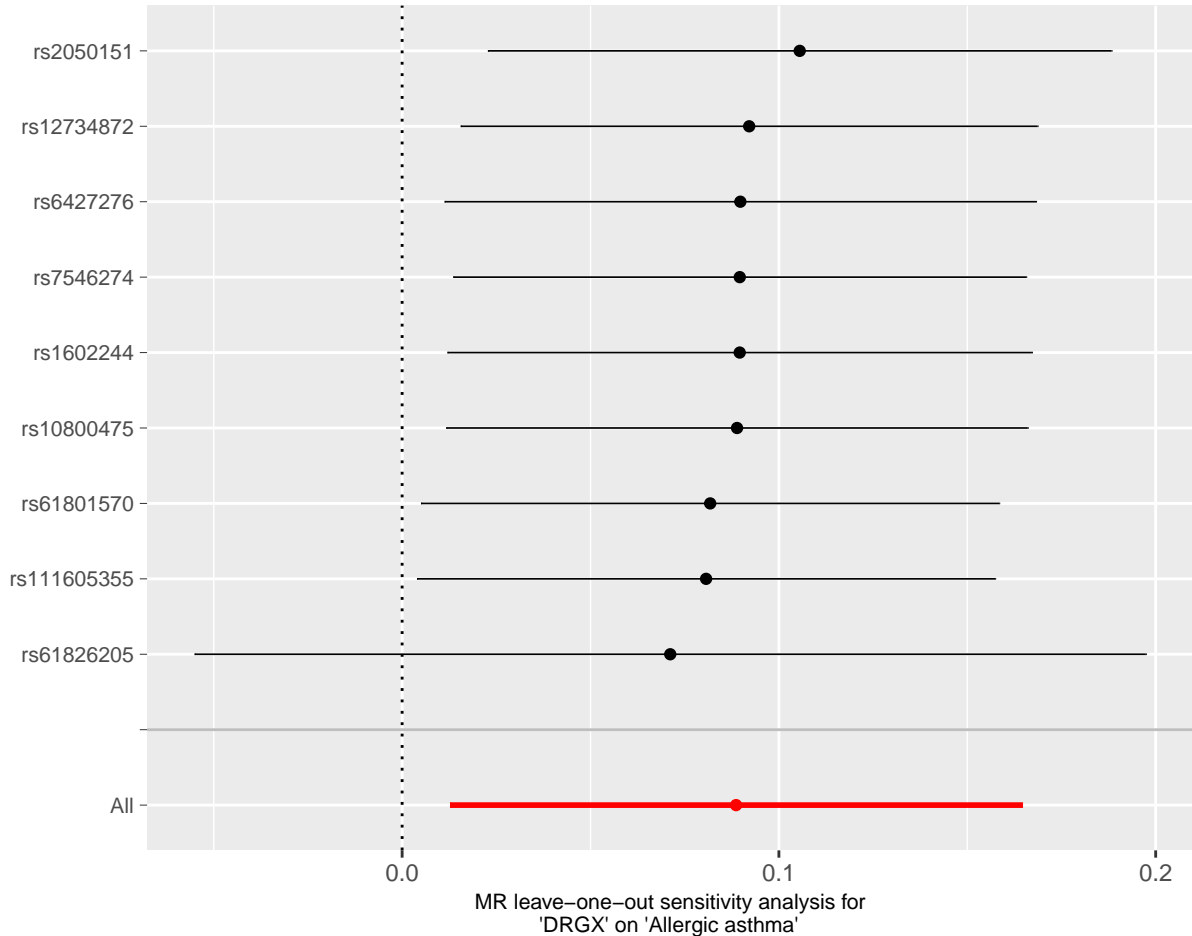

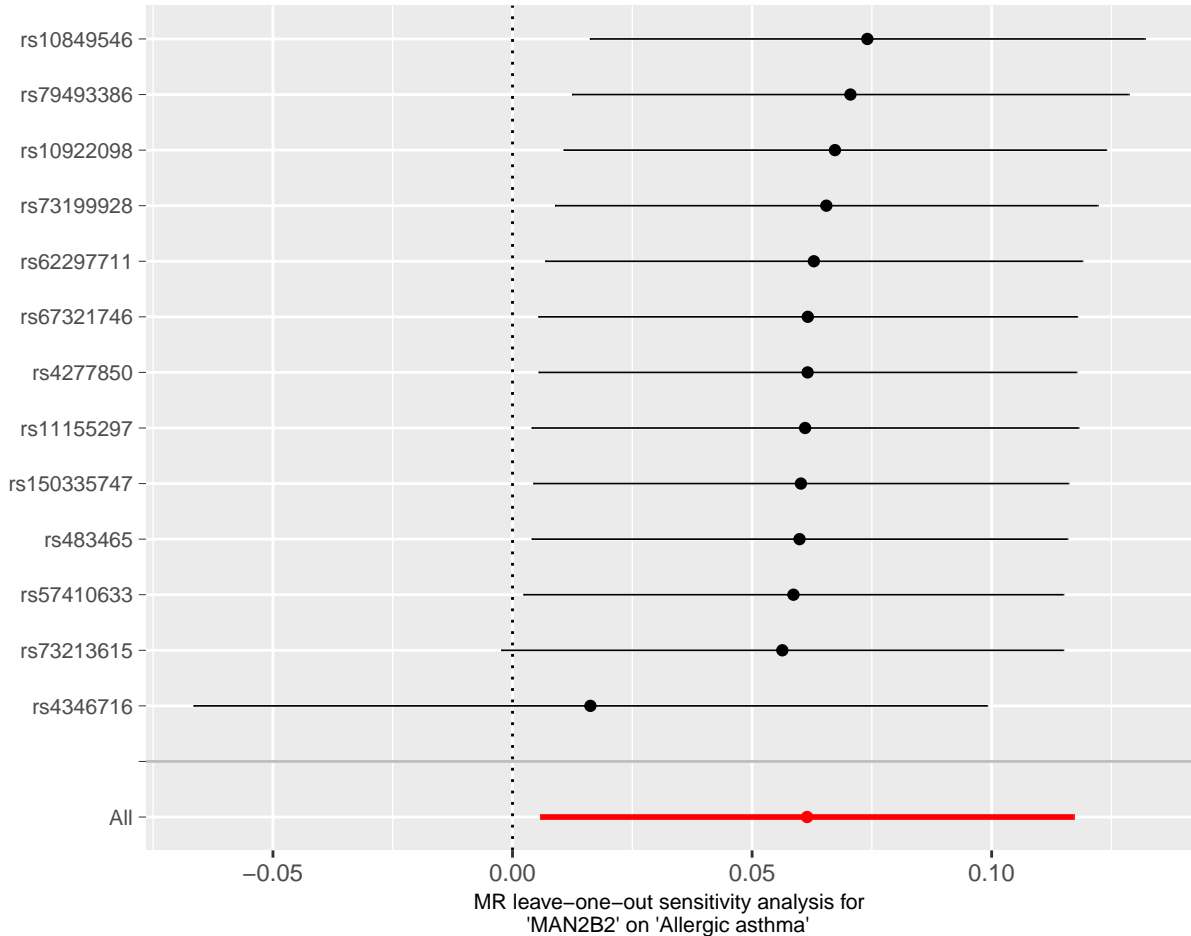

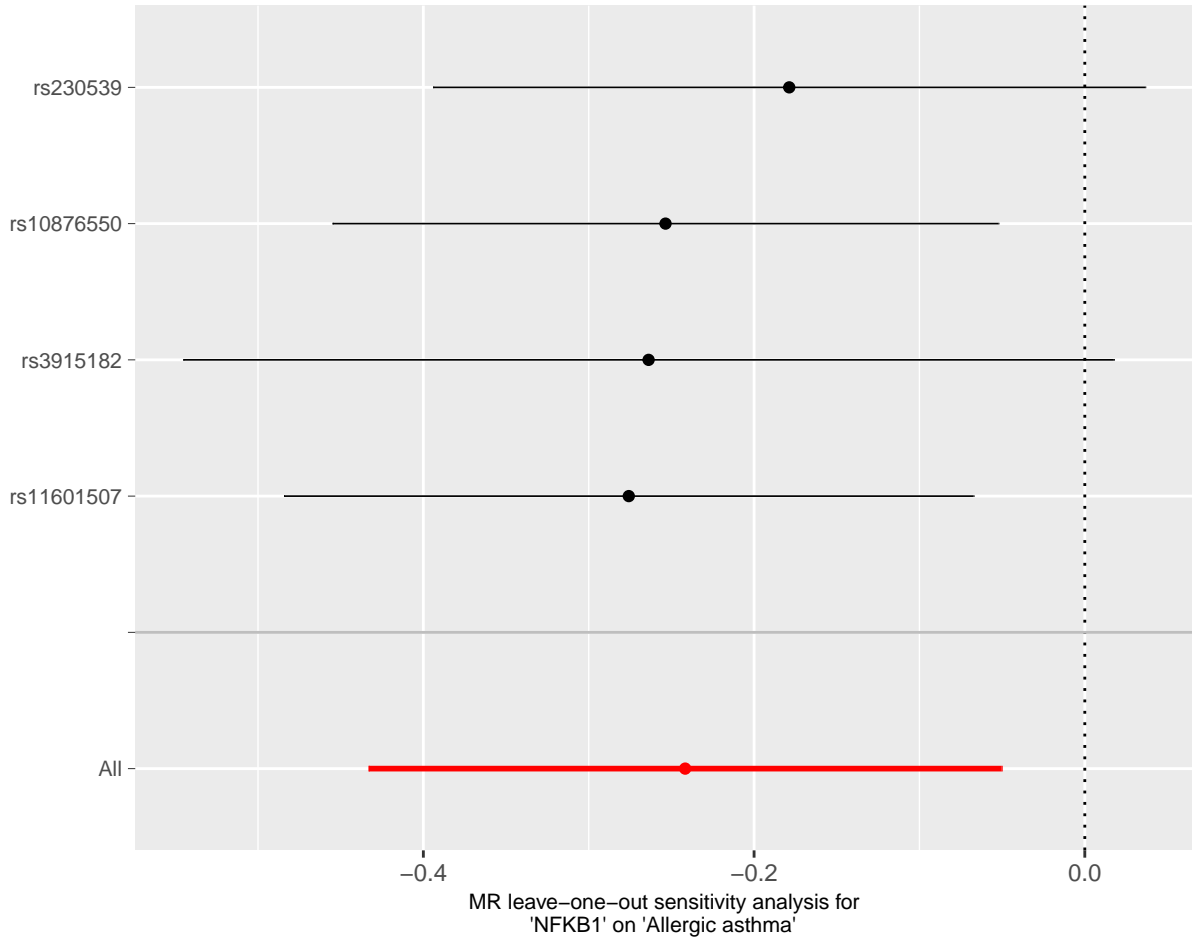

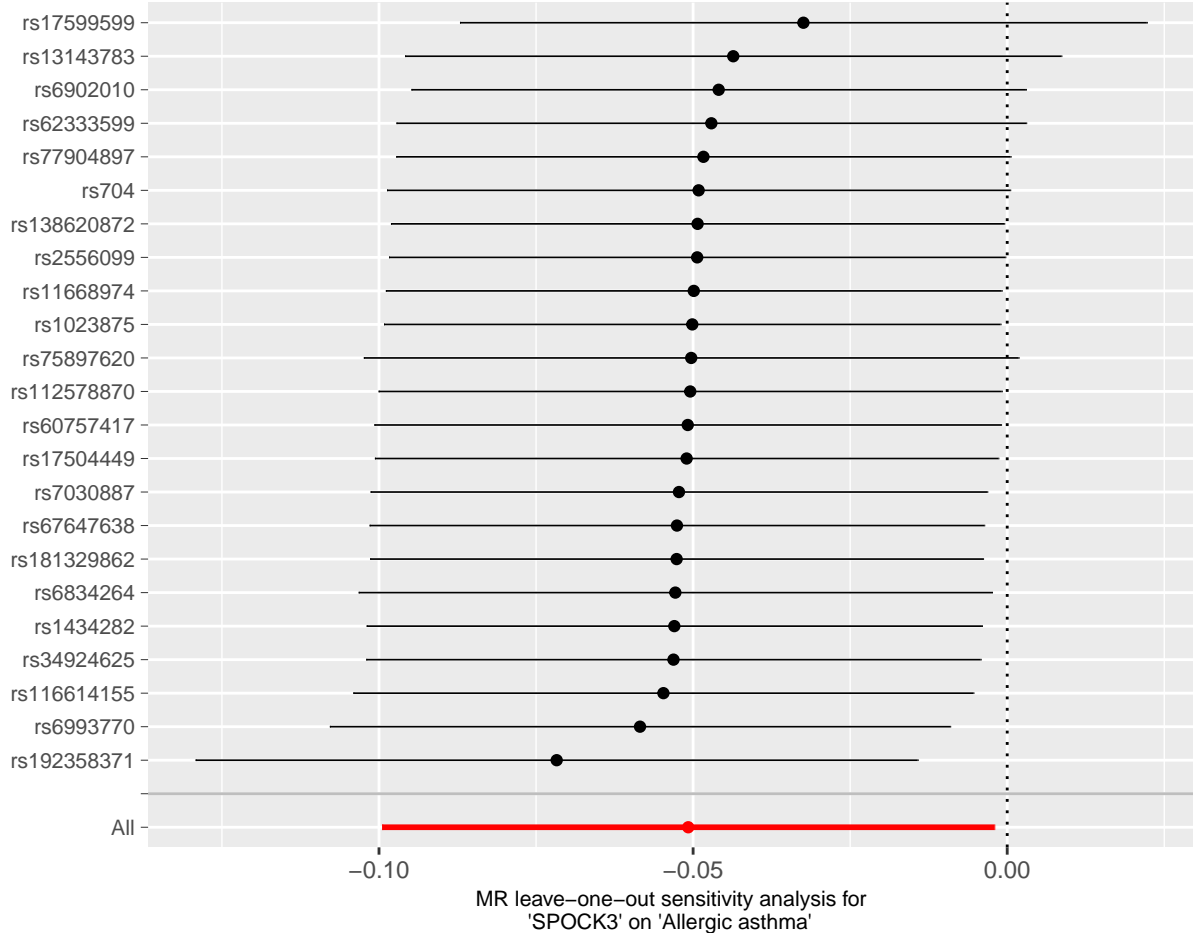

Supplement: Supplementary file 1 — Supplementary Material 1: Supplementary Materials 1. The MR results of BMI and allergic asthma. Supplementary Materials 2. The MR results of plasma proteins and allergic asthma. Supplementary Materials 3. The MR results of BMI and plasma proteins. Supplementary Materials 4. The MR results of mediation factor plasma proteins and allergic asthma. Supplementary Materials 5. The results of drug-targeted MR [file 41065_2025_376_MOESM1_ESM.zip › Supplementary Materials/S4 The MR results of mediation factor plasma proteins and allergic asthma/S4 plasma proteins-Allergic asthma.leaveoneout.pdf]

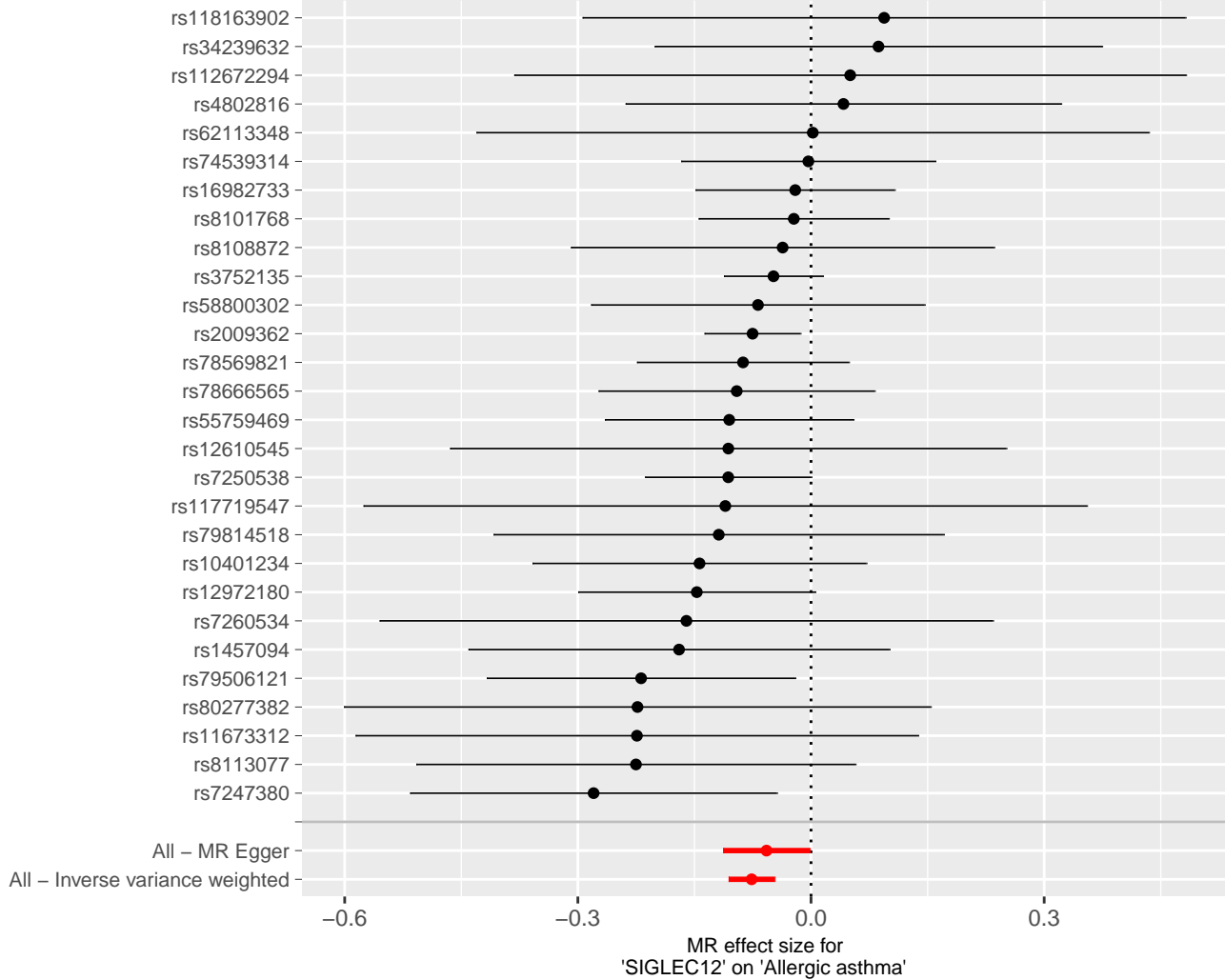

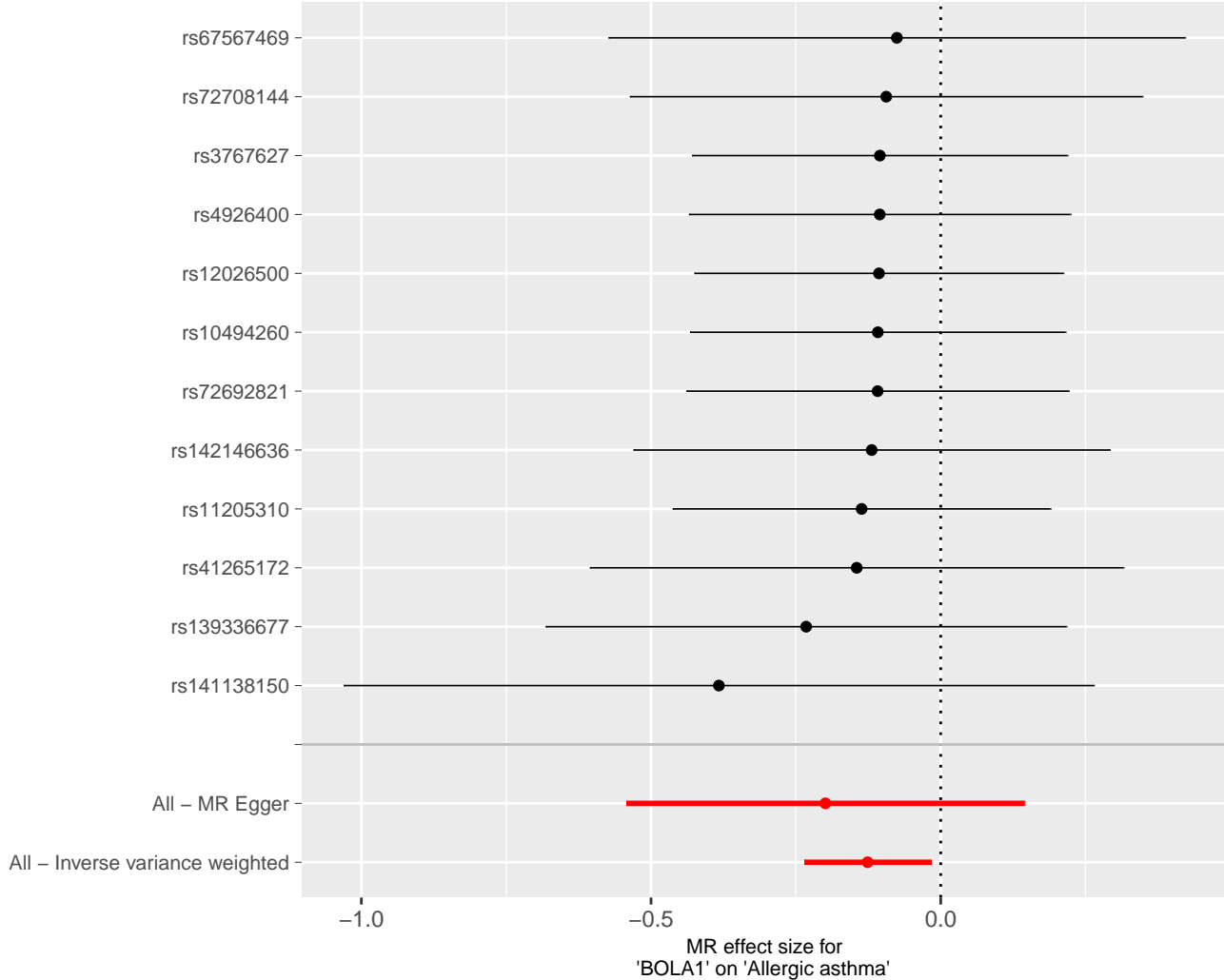

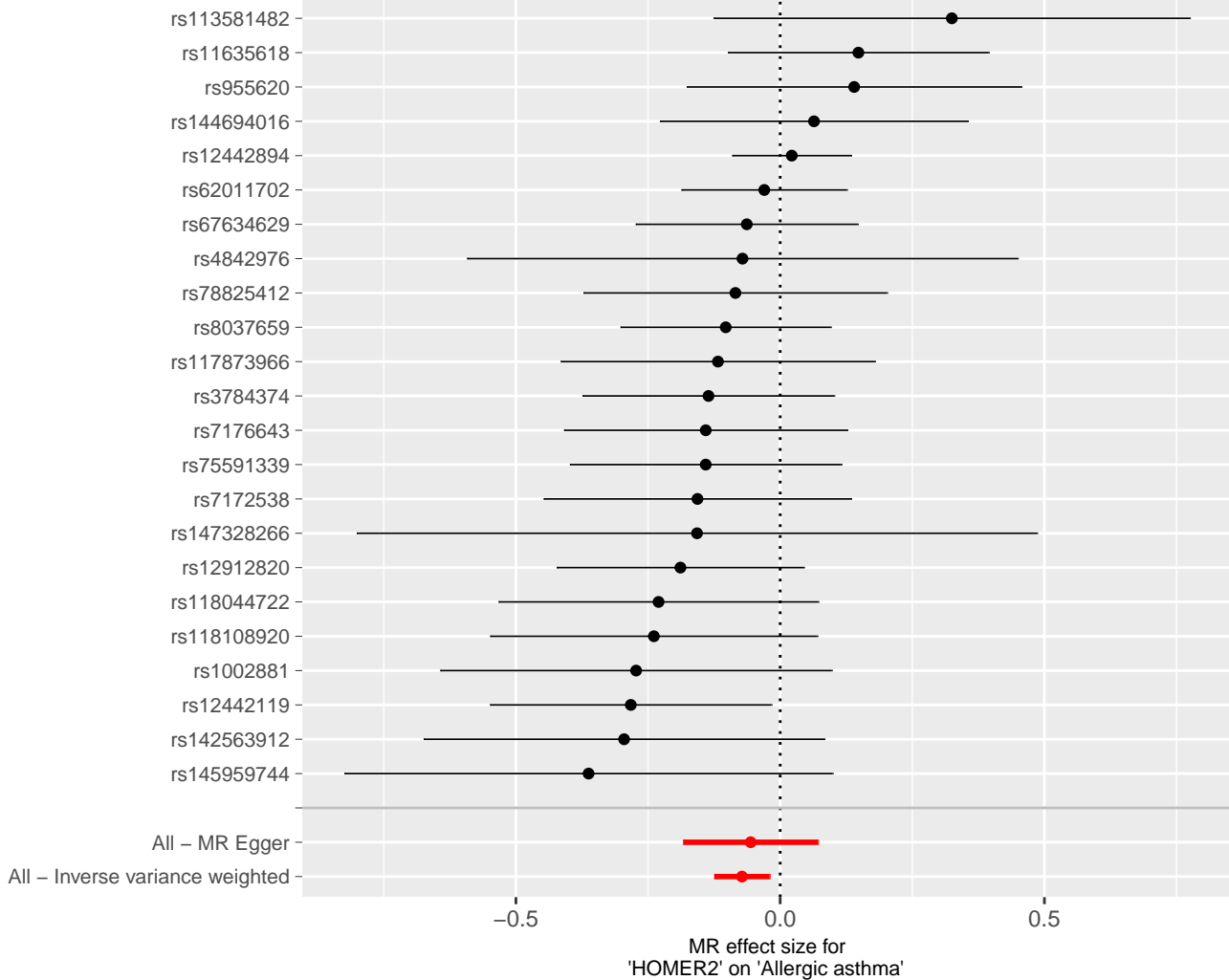

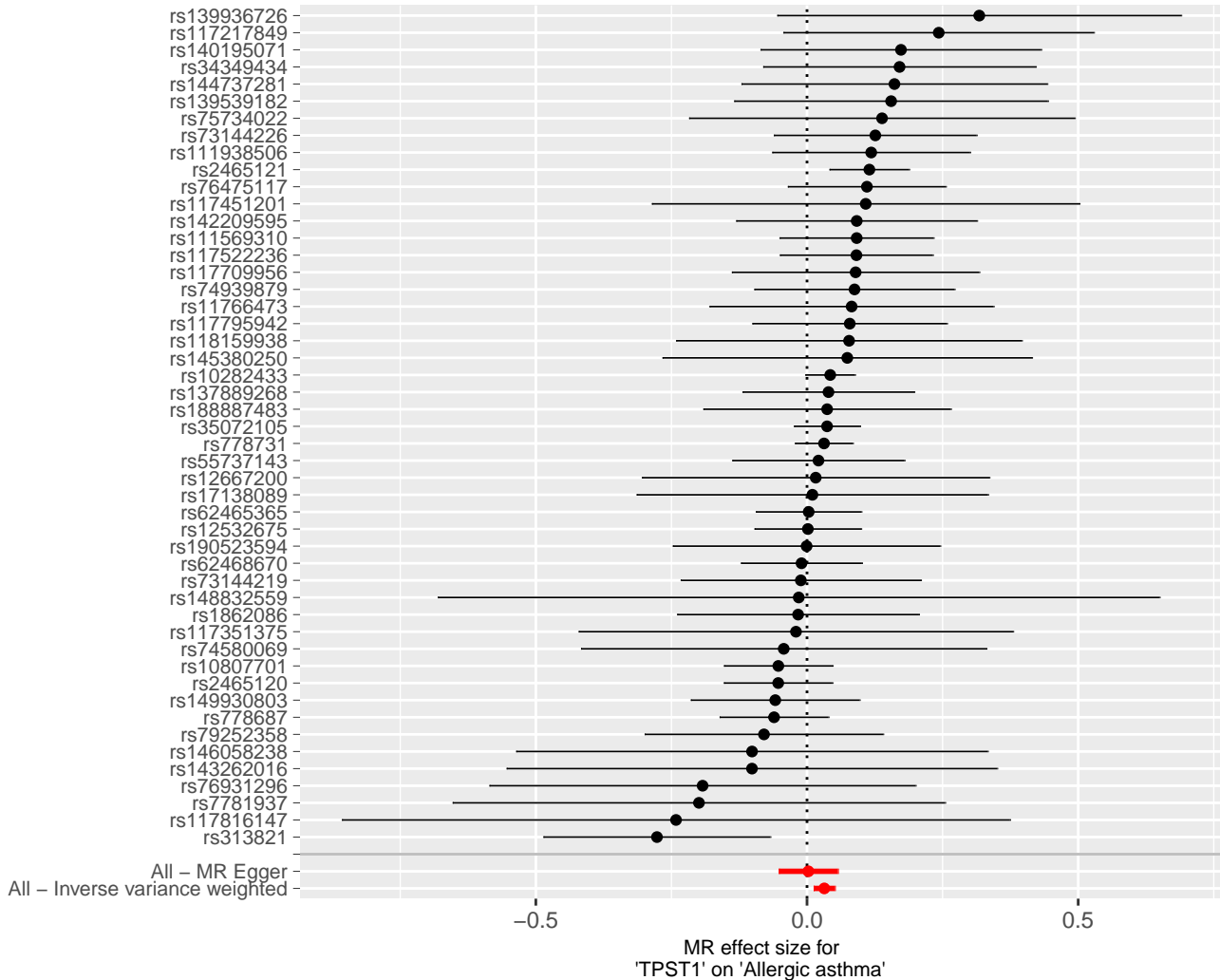

Supplement: Supplementary file 1 — Supplementary Material 1: Supplementary Materials 1. The MR results of BMI and allergic asthma. Supplementary Materials 2. The MR results of plasma proteins and allergic asthma. Supplementary Materials 3. The MR results of BMI and plasma proteins. Supplementary Materials 4. The MR results of mediation factor plasma proteins and allergic asthma. Supplementary Materials 5. The results of drug-targeted MR [file 41065_2025_376_MOESM1_ESM.zip › Supplementary Materials/S5 The results of drug-targeted MR/S5 The results of drug-targeted MR.forest.pdf]

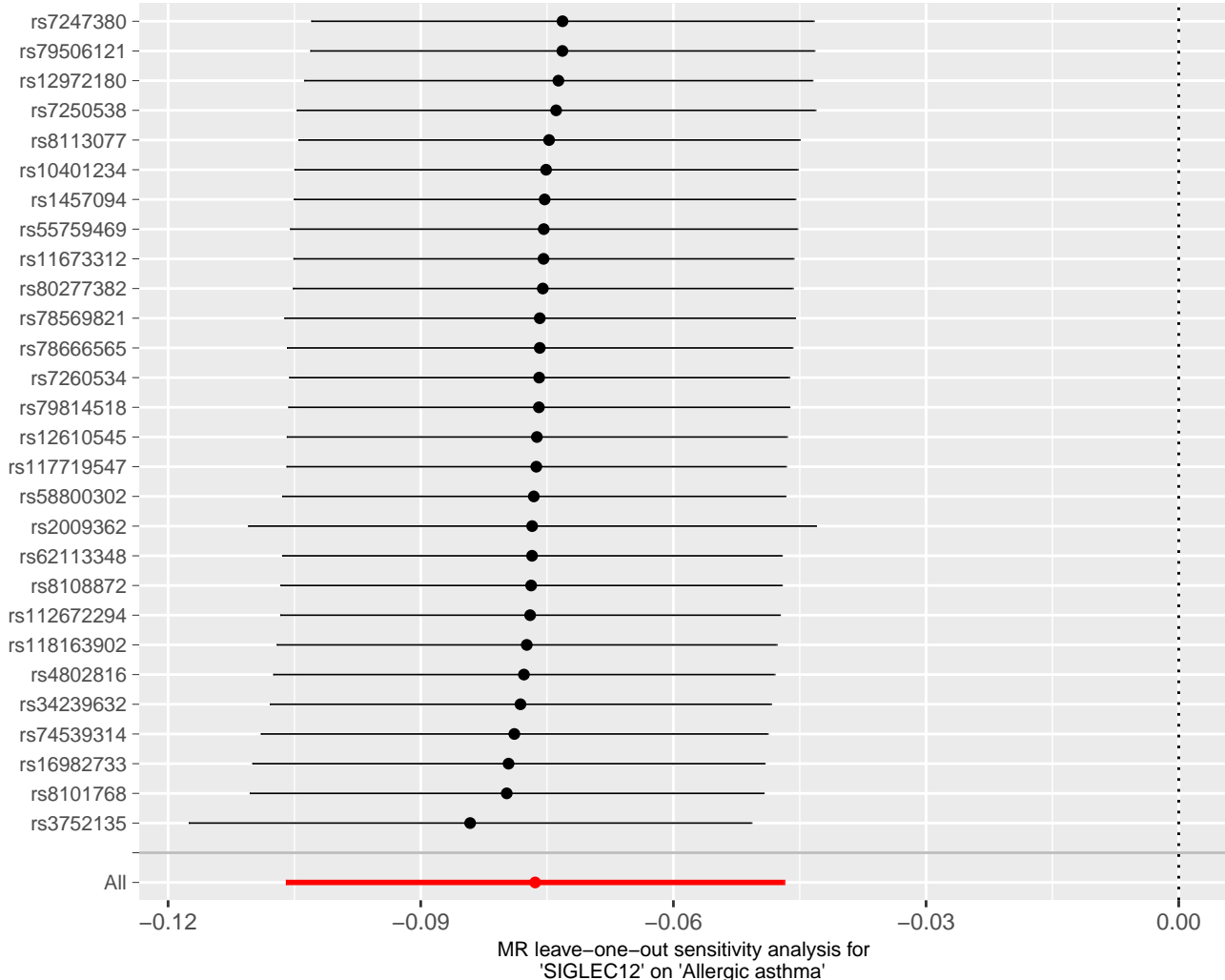

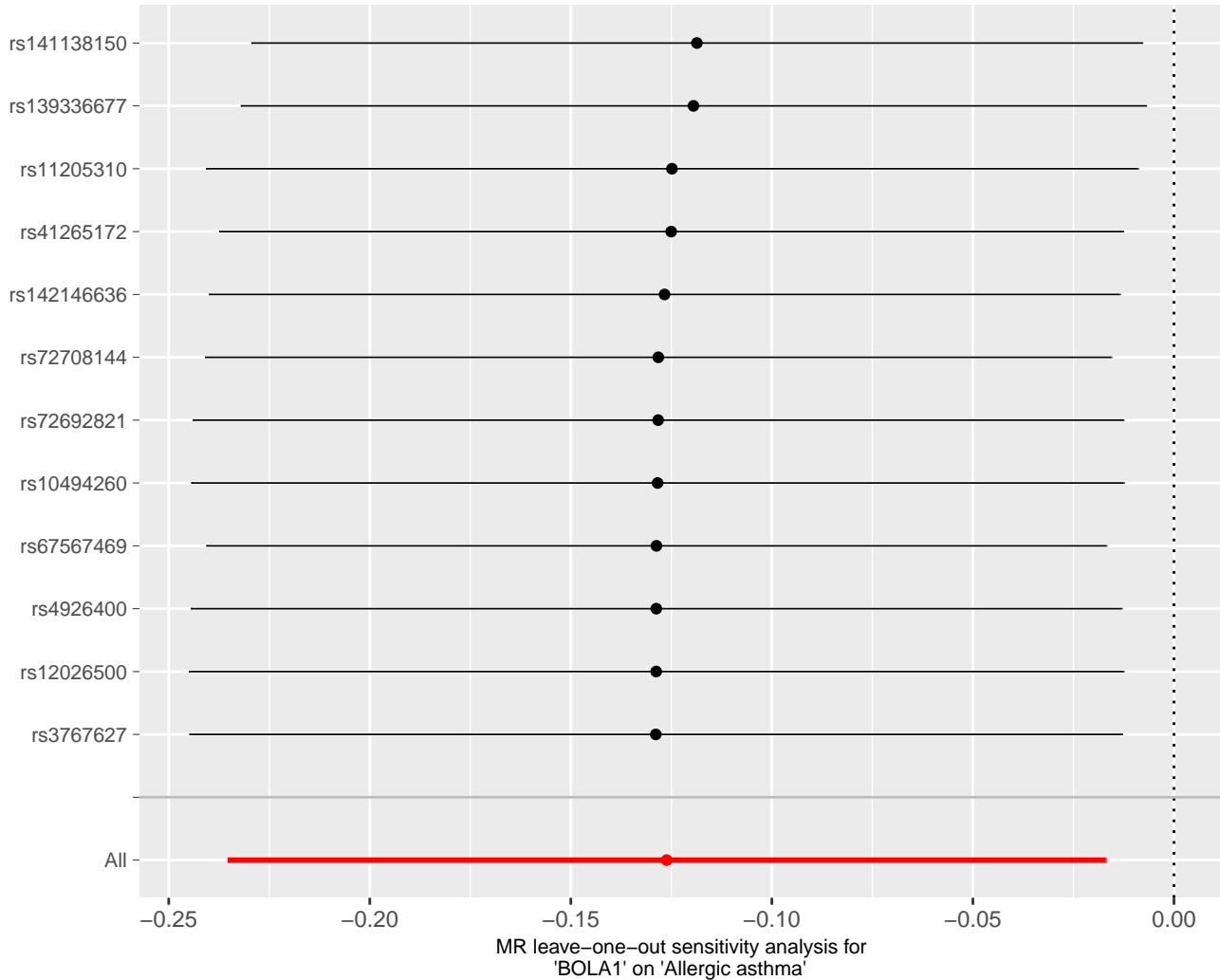

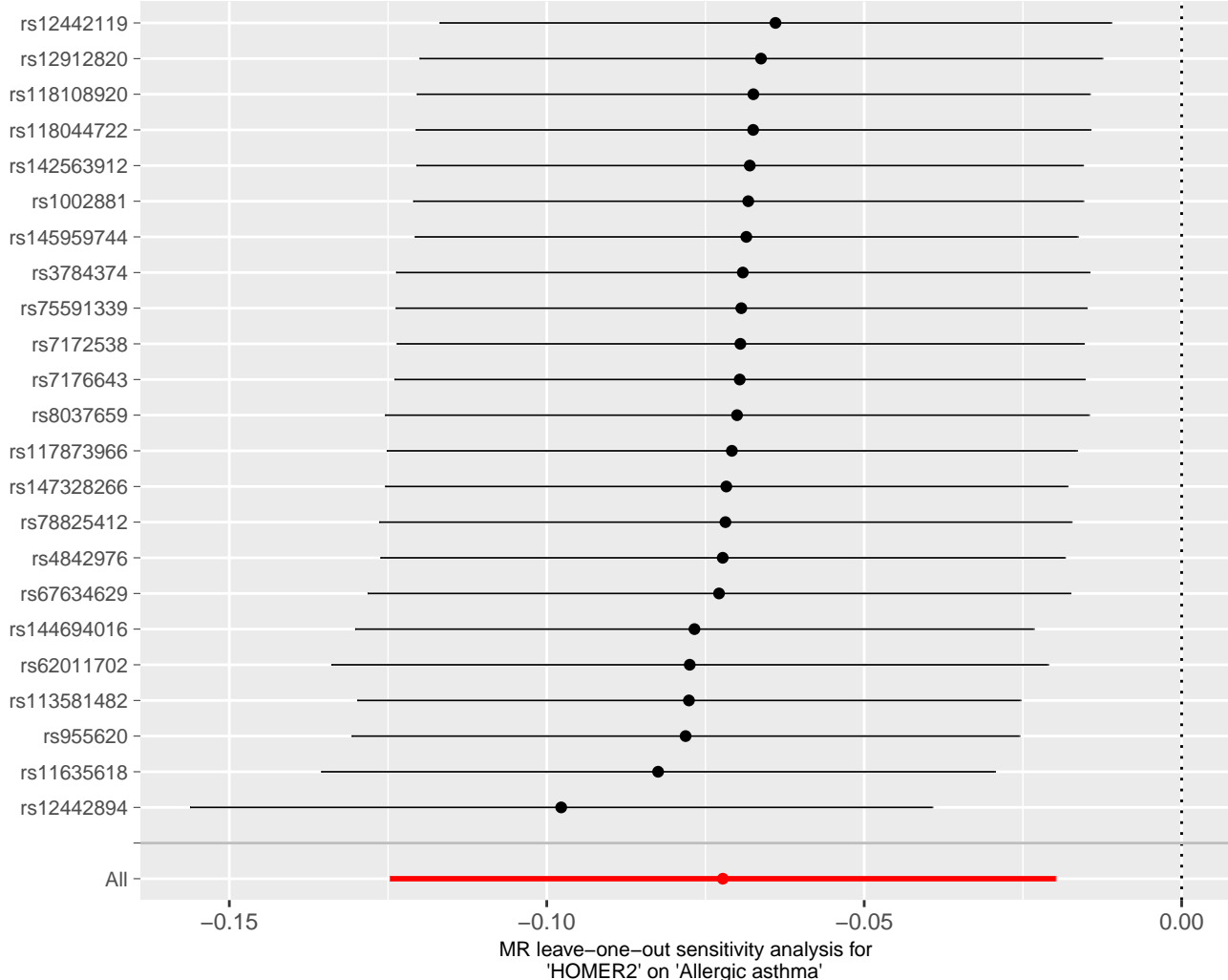

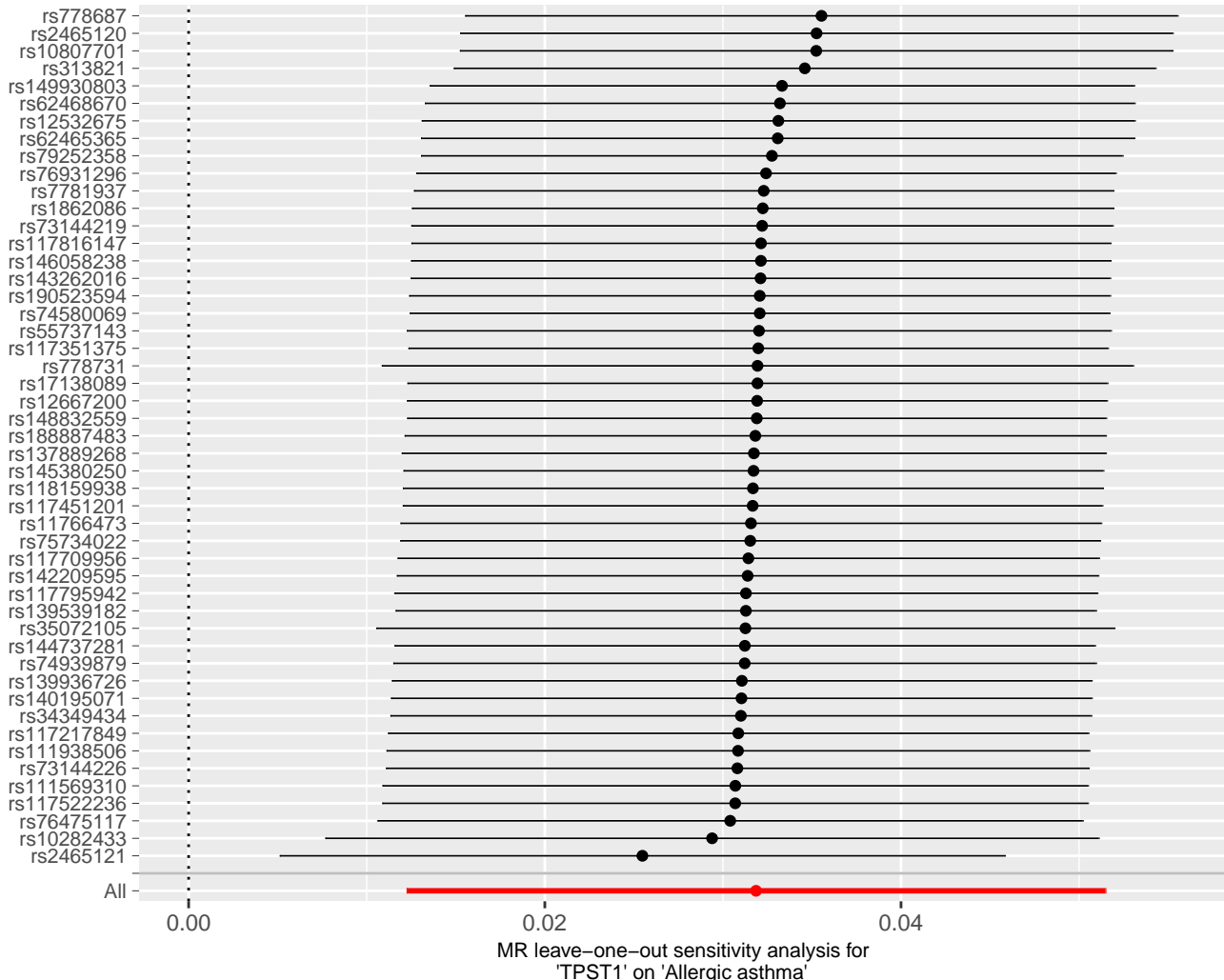

Supplement: Supplementary file 1 — Supplementary Material 1: Supplementary Materials 1. The MR results of BMI and allergic asthma. Supplementary Materials 2. The MR results of plasma proteins and allergic asthma. Supplementary Materials 3. The MR results of BMI and plasma proteins. Supplementary Materials 4. The MR results of mediation factor plasma proteins and allergic asthma. Supplementary Materials 5. The results of drug-targeted MR [file 41065_2025_376_MOESM1_ESM.zip › Supplementary Materials/S5 The results of drug-targeted MR/S5 The results of drug-targeted MR.leaveoneout.pdf]
